# Supplementary material for: Rapid syntheses of N-fused heterocycles via acyl-transfer in heteroaryl ketones
Source: Nat Commun. 2022 Jun 9;13:3337. doi: 10.1038/s41467-022-31063-3 (PMC9184603; doi:10.1038/s41467-022-31063-3)
Supplement: Supplementary file 1 — Supplementary Information [file 41467_2022_31063_MOESM1_ESM.pdf]

Supplementary Information for

Rapid Syntheses of *N*-Fused Heterocycles via Acyl-Transfer in Heteroaryl Ketones

Dan Ye,[1,‡] Hong Lu,[1,‡] Yi He,[1,2] Zhaojing Zheng,[2] Jinghao Wu[1] and Hao Wei[1,*]

[1]College of Chemistry & Materials Science, Northwest University, Xi'an 710069, China.

[2]College of Food Science and Technology, Northwest University, Xi'an 710069, China.

‡These authors contributed equally to this work.

*Correspondence: haow@nwu.edu.cn

Table of Contents

1. General Information 1

2. Supplementary Methods 2

2.1 Synthesis of Starting Materials 2

2.2. Optimization of Reaction Conditions 4

2.3 Experimental Procedures for the Expansion of the Substrate Scope 7

2.4 Supplementary Discussion 7

2.5 Synthetic Applications 12

3. Supplementary Spectra 14

3.1 X-Ray Crystallography 14

3.2 Characterization of Starting Materials 18

3.3 Characterization of Rearrangement Products 32

3.4 NMR Spectra 57

4. Supplementary References 220

1. General Information

All reagents were obtained commercially unless otherwise noted. Anhydrous dioxane (Adamas Reagent, Ltd.) and anhydrous tetrahydrofuran (THF) (Adamas Reagent, Ltd.) were used without further purification. Unless otherwise noted, all the rearrangement reactions were carried out under nitrogen atmosphere with a stir bar in a sealed vial. Reaction temperatures were reported as the temperatures of the bather surrounding the flasks or vials. Sensitive ligands and catalysts and solvents were transferred under nitrogen into a nitrogen-filled glovebox with standard techniques. Glass wares were heat-dried and cooled down under vacuum prior to use. Column chromatography was carried out on silica gel (300−400 mesh) using a forced flow of eluent at 0.3−0.5 bar pressure. Flash column chromatography was carried out using silica gel (200−300 mesh) at increased pressure. HRMS were performed on Bruker Daltonics MicroTof-Q II mass spectrometer. ^{1}H NMR, ^{13}C NMR and ^{19}F NMR spectra were recorded on a WNMR-I spectrometer (400 MHz ^{1}H and 100 MHz ^{13}C). The spectra were recorded in $CDCl_3$ as the solvent at room temperature. ^{1}H and ^{13}C chemical shifts are reported in ppm relative to either the residual solvent peak (^{13}C) or TMS (^{1}H) as an internal standard.

2. Supplementary Methods

2.1 Synthesis of Starting Materials

2.1.1 General procedure A for preparation of heteroaryl ketones

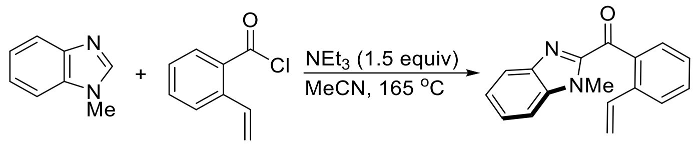

Step for heteroaryl ketones 1

The mixture of 1-methylbenzimidazole (2.7 mmol), triethylamine (4.0 mmol), acetonitrile (1.0 mL) and 2-vinylbenzoyl chloride (4.0 mmol) was added and heated at 165 °C for 1 h, then 10.0 mL acetone was added to it, and the solution was poured into 7% hydrochloric acid (40.0 mL) under stirring. The mixture was stirred and refluxed with charcoal for at least 30 minutes. The filtrate was alkalized with 20% sodium hydroxide to separate compound **1**. which was purified by column chromatography on silica gel to afford the desired product heteroaryl ketones **1**.

Steps for other heteroaryl ketones derived from (benzo)imidazoles

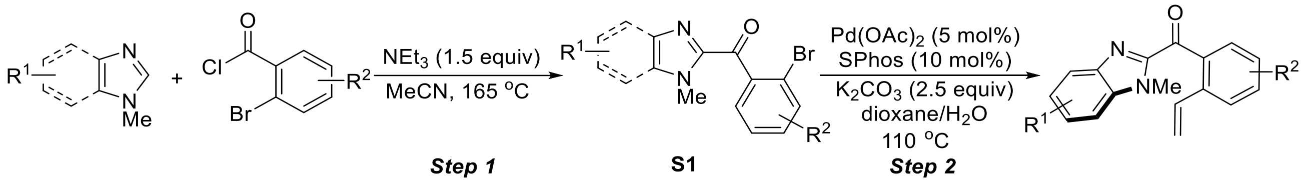

Step 1: the mixture of 1-methylbenzimidazole or 1-methylimidazole (14.0 mmol), acetonitrile (2.0 mL), triethylamine (21.0 mmol) and benzoyl chloride (21.0 mmol) was heated at 165 °C for 1 h, then 25.0 mL acetone was added to it, and the solution was poured into 7% hydrochloric acid (120.0 mL) under stirring. The mixture was stirred and refluxed with charcoal for at least 30 minutes. The filtrate was alkalized with 20% sodium hydroxide to separate compound **S1**.

Step 2: potassium vinyl trifluoroborate (3.75 mmol), K_2CO_3 (5.0 mmol), $Pd(OAc)_2$ (0.025 mmol, 5.61 mg), compound **S1** (2.5 mmol), 1,4-dioxane (2.5 mL) and H_2O (1.0 mL) were placed in a Schlenk flask. Then, the flask was evacuated and backfilled with N_2 for three times. The reaction mixture was stirred at 110 °C for 12 h and the reaction mixture was cooled down to room temperature. The aqueous layer was extracted with dichloromethane (10.0 mL) three times. The combined organic layer was dried over anhydrous Na_2SO_4 and evaporated under reduced pressure. The crude product was purified by column chromatography on silica gel to afford the desired product heteroaryl ketones.

2.1.2 General procedure B for preparation of heteroaryl ketones derived from (benzo)thiazoles

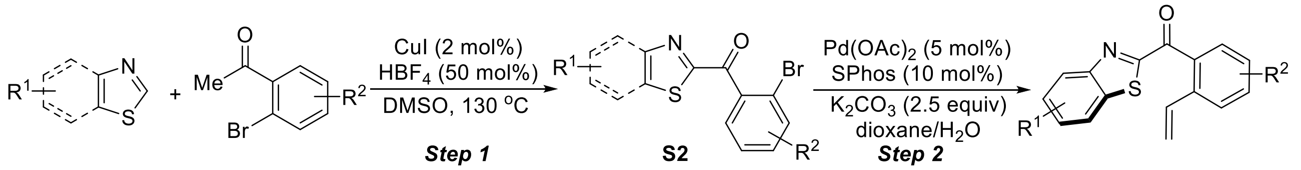

Step 1: add thiazole or benzothiazole (5.0 mmol), CuI (19.0 mg, 2 mol%), 1-(2-bromophenyl)ethanone (10.0 mmol), HBF_4 (2.5 mmol, 40% in aqueous) and DMSO (7.5 mL) to a sealed tube. Purge the resulting solution by N_2 and seal the tube. Stir the solution at 130 °C under N_2 for 9 hours (monitored by TLC). Upon completion of the reaction, add ethyl acetate (20.0 mL) to the solution. Wash the organic layer with saturate $NaHCO_3$ solution, brine. Extract the combined aqueous layers with ethyl acetate. Dry the combined organic layers over anhydrous Na_2SO_4. Remove the solvent through rotary evaporator. Purify **S2** by flash chromatography.

Step 2: potassium vinyl trifluoroborate (3.75 mmol), K_2CO_3 (5.0 mmol), $Pd(OAc)_2$ (0.025 mmol, 5.61 mg), compound **S2** (2.5 mmol), 1,4-dioxane (2.5 mL) and H_2O (1.0 mL) were placed in a Schlenk flask. Then, the flask was evacuated and backfilled with N_2 for three times. The reaction mixture was stirred at 110 °C for 12 h and the reaction mixture was cooled down to room temperature. The aqueous layer was extracted with dichloromethane (10.0 mL) three times. The combined organic layer was dried over anhydrous Na_2SO_4 and evaporated under reduced pressure. The crude product was purified by column chromatography on silica gel to afford the desired heteroaryl ketones.

2.1.3 General procedure C for preparation of heteroaryl ketones derived from (benzo)oxazoles

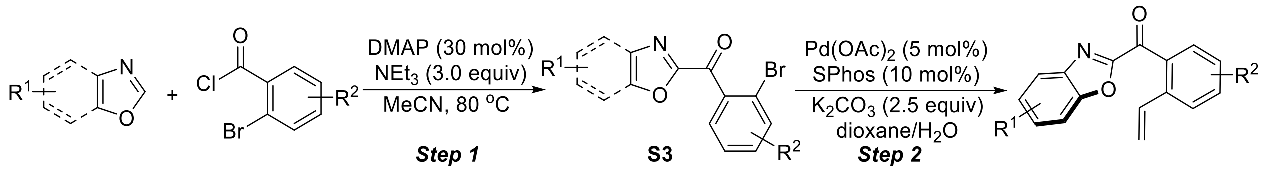

Step 1: add *N*, *N*-dimethyl-4-aminopyridine (370.0 mg, 30.0 mol%), benzoyl chloride (20.0 mmol) and benzoxazole (10.0 mmol) in a sealable tube. Flush the tube with a stream of N_2. Acetonitrile (20.0 mL) was added to the tube, followed by triethylamine (4.2 mL). The reaction mixture was stirred at 80 °C for 24 hours. Cool the mixture to room temperature. Dilute the mixture with ethyl acetate and saturated $NaHCO_3$ aqueous solution. Extract the mixture with ethyl acetate. Wash the combined organic layers with brine. The combined organic layers were dried on Na_2SO_4. The combined organic layers are concentrated in vacuum. The product **S3** was purified by rapid column chromatography.

Step 2: potassium vinyl trifluoroborate (3.75 mmol), K_2CO_3 (5.0 mmol), $Pd(OAc)_2$ (0.025 mmol, 5.61 mg), compound **S3** (2.5 mmol), 1,4-dioxane (2.5 mL) and H_2O (1.0 mL) were placed in a Schlenk flask. Then, the flask was evacuated and backfilled with N_2 for three times. The reaction mixture was stirred at 110 °C for 12 h and the reaction mixture was cooled down to room temperature. The aqueous layer was extracted with dichloromethane (10.0 mL) three times. The combined organic layer was dried over anhydrous Na_2SO_4 and evaporated under reduced pressure. The crude product was purified by column chromatography on silica gel to afford the desired heteroaryl ketones.

2.1.4 General procedure D for preparation of difluoroalkyl bromide derivatives

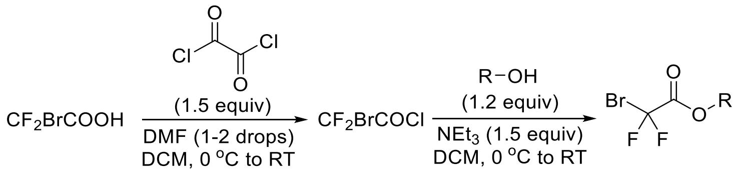

Bromodifluoroacetic acid (10.0 mmol) was added to a round bottomed flask at 0 °C, drying CH_2Cl_2 (30.0 mL), oxalyl chloride (15.0 mmol) and 1-2 drops of DMF were added to the reaction mixture, and the reaction was carried out at room temperature for 3 h. The reaction was then transferred to 0 °C, alcohol (12.0 mmol) and Et_3N (15.0 mmol) was added to the reaction mixture. The reaction mixture was stirred at this temperature for 10 min. The reaction mixture was stirred at room temperature for about 14 h. Quench the reaction mixture with HCl (1.0 M, 5.0 mL). The aqueous phase was extracted with CH_2Cl_2. Wash the combined organic phases with brine. Dry the combined organic phases on anhydrous Na_2SO_4. Purify the residue by rapid column chromatography to obtain the target product.

2.1.5 General procedure E for preparation of difluoroalkyl bromide derivatives

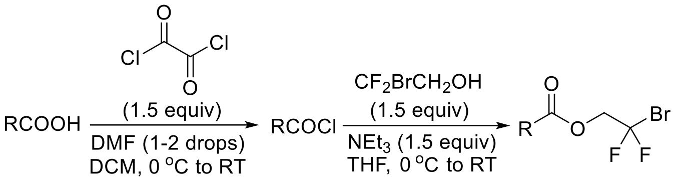

Acid (10.0 mmol) was added to a round bottomed flask at 0 °C, drying CH_2Cl_2 (30.0 mL), oxalyl chloride (15.0 mmol) and 1-2 drops of DMF were added to the reaction mixture, and the reaction was carried out at room temperature for 3 h. After concentration, dissolve in THF (30.0 mL), transfer the reaction to 0 °C, add alcohol (15.0 mmol) to the mixture, stir the reaction mixture at this temperature for 10 minutes, and add Et_3N (15mmol) to the reaction mixture. Stir the reaction mixture at room temperature for about 14 h. Quench the reaction mixture with HCl (1.0 M, 5.0 mL). Extract the aqueous phase with CH_2Cl_2. Wash the combined organic phases with brine. Dry the combined organic phases on anhydrous Na_2SO_4. Purify the residue by rapid column chromatography to obtain the target product.

2.2. Optimization of Reaction Conditions

2.2.1 General procedure for optimization

In an N_2-filled glovebox, an oven-dried 10 mL sealed tube equipped with a Teflon-coated magnetic stir bar was charged successively with ketone **1** (0.1 mmol), brominated precursors (0.15 mmol, 1.5 equiv), [Pd] precatalyst (0.01 mmol, 10 mol%), ligand (0.012mmol, 12 mol%), base (0.1 mmol, 1.0 equiv) and solvent (1.0 mL). The tube then was sealed with a Teflon screw cap, moved out of the glovebox, and placed on a hotplate pre-heated to 130 °C with vigorous stirring. After 24 h, cool the reaction to room temperature and purify it by silica gel rapid chromatography.

Supplementary Table 1. Screening for Catalysts[a]

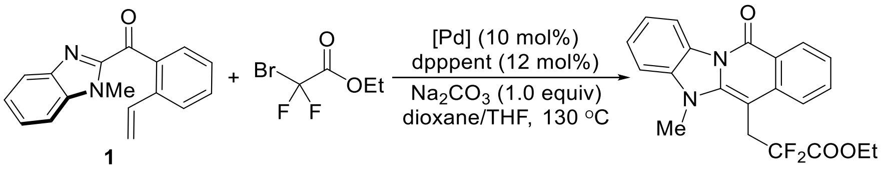

Entry	[Pd] precatalyst	Yiled (%)[b]
1	$PdCl_2$	90
2	$Pd_2(bda)_3$	51
3	$Pd[P(o\text{-}Tol)_3]_2$	55
4	$Pd(OAc)_2$	54
5	$Pd(PPh_3)_2Cl_2$	59
6	$Pd(PPh_3)_4$	57
7	Pd-PEPPS-Ipr	37
8	$PdCl_2$(dppf)	72
9	-	n.r.

[a] Unless otherwise specified, all reactions were carried out using **1** (0.1 mmol) and difluorobromoethyl ester (0.15 mmol) with 10 mol% [Pd] precatalyst, 10 mol% dpppent and Na_2CO_3 (1.0 equiv) in dioxane/THF (1:2) at 130 °C for 24 h. [b] Isolated yields after chromatography.

Supplementary Table 2. Screening for Solvents[a]

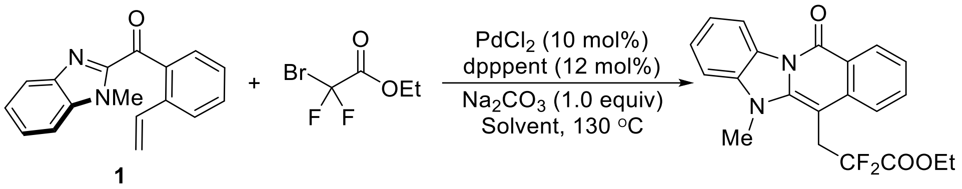

Entry	Ligand	Yiled (%)[b]
1	MeCN	57
2	THF	68
3	1,4-dioxane	76
4	DCM	68
5	Toluene	trace
6	DMF	2
7	DCE	66
8	PhCl	52
9	1,4-dioxane /THF (1:1)	72
10	1,4-dioxane /THF (1:2)	90
11	1,4-dioxane /THF (2:1)	69

[a] Unless otherwise specified, all reactions were carried out using **1** (0.1 mmol) and difluorobromoethyl ester (0.15 mmol), with 10 mol% $PdCl_2$, 12 mol% dpppent and Na_2CO_3 (1.0 equiv) in solvent at 130 °C for 24 h. [b] Isolated yields after chromatography.

Supplementary Table 3. Screening for Bases[a]

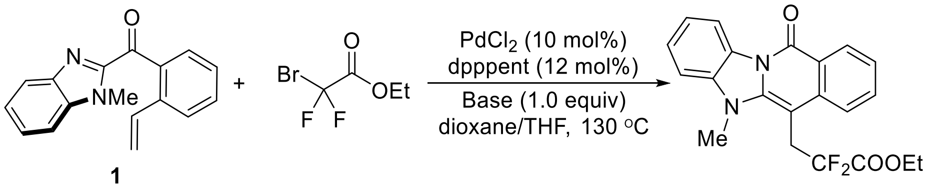

Entry	Base	Yiled (%)[b]
1	K_2CO_3	58
2	Na_2CO_3	90
3	$NaHCO_3$	4
4	K_3PO_4	48
5	Na_2HPO_4	18
6	$KHCO_3$	48
7	CH_3COONa	20
8	KF	15
9	Et_3N	9
10	-	12

[a] Unless otherwise specified, all reactions were carried out using **1** (0.1 mmol) and difluorobromoethyl ester (0.15 mmol), with 10 mol% $PdCl_2$, 12 mol% dpppent and base (1.0 equiv) in dioxane/THF (1:2) at 130 °C for 24 h. [b] Isolated yields after chromatography.

Supplementary Table 4. Screening for Ligands of (Benzo)thiazole[a]

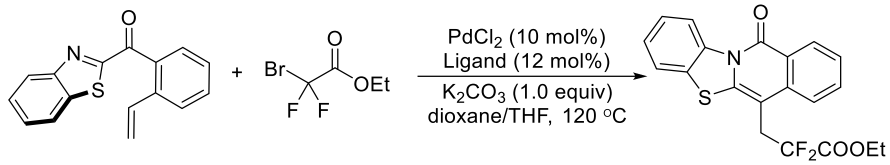

Entry	Ligand	Yiled (%)[b]
1	[1,1'-Bis(diphenylphosphino)ferrocene]dichloropalladium(II)<br>dppf	78
2	1,2-Bis(diphenylphosphino)ethane<br>dppe	58
3	1,3-Bis(diphenylphosphino) propane<br>dppp	50
4	1,4-Bis(diphenylphosphino)butane<br>dppb	51
5	1,1-Bis(diphenylphosphino)pentane<br>dpppent	59
6	1,6-Bis(diphenylphosphino)hexane<br>dppph	54
7	Tris(4-fluorophenyl)phosphine	55
8	Tri(2-furyl)phosphine	36
9	Cyclohexylhydrazinehydrochloride	40

[a] Unless otherwise specified, all reactions were carried out using benzothiazole ketone (0.1 mmol) and difluorobromoethyl ester (0.15 mmol), with 10 mol% $PdCl_2$, 12 mol% ligand and K_2CO_3 (1.0 equiv) in dioxane/THF (1:1) at 120 °C for 24 h. [b] Isolated yields after chromatography.

Supplementary Table 5. Screening for Bases of (Benzo)thiazole[a]

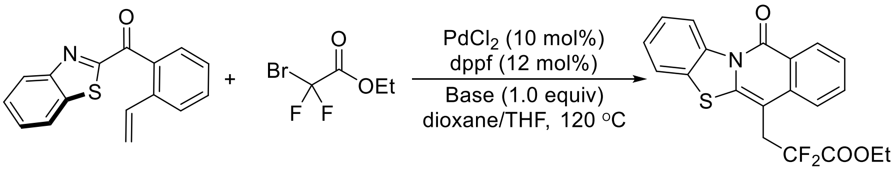

Entry	Base	Yiled (%)[b]
1	K_2CO_3	78
2	Na_2CO_3	66
3	$NaHCO_3$	38
4	K_3PO_4	trace
5	Na_2HPO_4	trace
6	$KHCO_3$	18
7	CH_3COONa	n.r.
8	KF	23
9	Et_3N	n.r.
10	-	trace

[a] Unless otherwise specified, all reactions were carried out using benzothiazole ketone (0.1 mmol) and difluorobromoethyl ester (0.15 mmol), with 10 mol% $PdCl_2$, 12 mol% dppf and base (1.0 equiv) in dioxane/THF (1:1) at 120 °C for 24 h. [b] Isolated yields after chromatography.

2.3 Experimental Procedures for the Expansion of the Substrate Scope

2.3.1 General procedure F for transfer-annulation of heteroaryl ketones derived from (benzo)imidazoles.

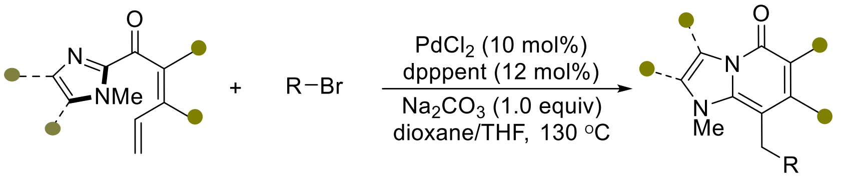

In a nitrogen-filled glovebox, an oven-dried 10 mL sealed tube equipped with a Teflon-coated magnetic stir bar was charged successively with heteroaryl ketones (0.1 mmol), brominated precursors (0.15 mmol, 1.5 equiv), $PdCl_2$ (0.01 mmol, 10 mol%), dpppent (0.012 mmol, 12 mol%), Na_2CO_3 (0.1 mmol, 1.0 equiv) and dioxane/THF (1.0 mL, 1:2). The tube then was sealed with a Teflon screw cap, moved out of the glovebox, and placed on a hotplate pre-heated to 130 °C for 24-36 h. After completion of the reaction, the mixture was filtered through a thin pad of silica gel. The filter cake was washed with ethyl acetate and the combined filtrate was concentrated under vacuum. The residue was purified via silica gel chromatography.

2.3.2 General procedure G for transfer-annulation of heteroaryl ketones derived from (benzo)thiazoles and (benzo)oxazoles

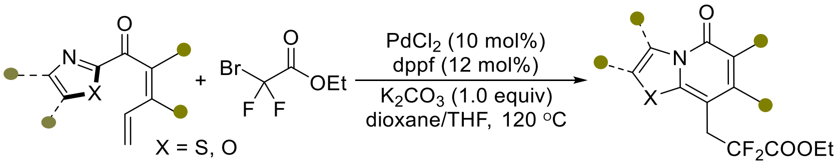

In a nitrogen-filled glovebox, an oven-dried 10 mL sealed tube equipped with a Teflon-coated magnetic stir bar was charged successively with heteroaryl ketone (0.1 mmol), difluorobromoethyl ester (0.15 mmol, 1.5 equiv), $PdCl_2$ (0.01 mmol, 10 mol%), dppf (0.012 mmol, 12 mol%), K_2CO_3 (0.1 mmol, 1.0 equiv) and dioxane/THF (1.0 mL, 1:1). The tube then was sealed with a Teflon screw cap, moved out of the glovebox, and placed on a hotplate pre-heated to 120 °C for 24 h. After completion of the reaction, the mixture was filtered through a thin pad of silica gel. The filter cake was washed with ethyl acetate and the combined filtrate was concentrated under vacuum. The residue was purified via silica gel chromatography.

2.4 Supplementary Discussion

2.4.1 Radical inhibition experiments

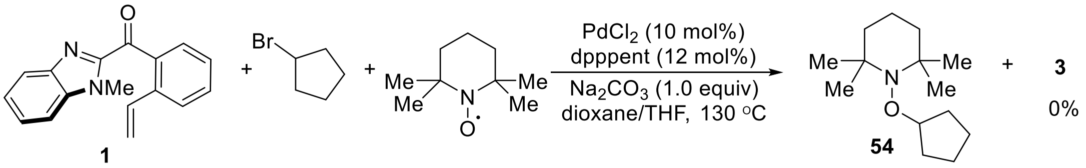

calcd for $[C_{14}H_{28}NO]^+$: 226.2165
observed for $[C_{14}H_{28}NO]^+$: 226.2172

In an N_2-filled glovebox, an oven-dried 10 mL sealed tube equipped with a Teflon-coated magnetic stir bar was charged successively with ketone **1** (0.1 mmol), bromocyclopentane (0.15 mmol, 1.5 equiv), TEMPO (0.2 mmol, 2.0 equiv), $PdCl_2$ (0.01 mmol, 10 mol%), dpppent (0.012 mmol, 12 mol%), Na_2CO_3 (0.1 mmol, 1.0 equiv) and dioxane/THF (1.0 mL, 1:2). The tube then was sealed with a Teflon screw cap, moved out of the glovebox, and placed on a hotplate pre-

heated to 130 °C with vigorous stirring. After 24 h, cool the reaction to room temperature and diluted with CH_2Cl_2 (2.0 mL). The resulting reaction solution was analyzed by HRMS, no desired product **3** was detected, and a TEMPO added product **54** was observed.

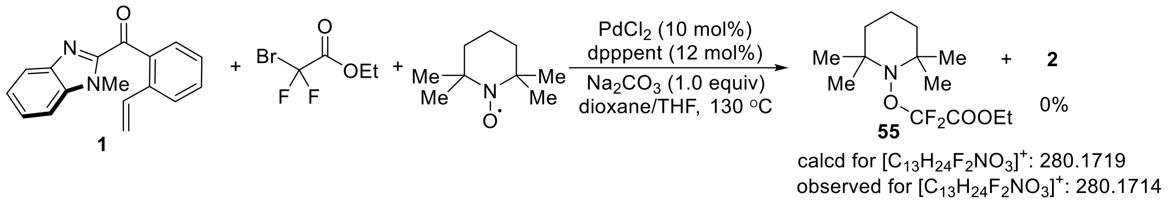

In an N_2-filled glovebox, an oven-dried 10 mL sealed tube equipped with a Teflon-coated magnetic stir bar was charged successively with ketone **1** (0.1 mmol), difluorobromoethyl ester (0.15 mmol, 1.5 equiv), TEMPO (0.2 mmol, 2.0 equiv), $PdCl_2$ (0.01 mmol, 10 mol%), dpppent (0.012 mmol, 12 mol%), Na_2CO_3 (0.1 mmol, 1.0 equiv) and dioxane/THF (1.0 mL, 1:2). The tube then was sealed with a Teflon screw cap, moved out of the glovebox, and placed on a hotplate pre-heated to 130 °C with vigorous stirring. After 24 h, cool the reaction to room temperature and diluted with CH_2Cl_2 (2.0 mL). The resulting reaction solution was analyzed by HRMS, no desired product **2** was detected, and a TEMPO added product **55** was observed.

2.4.2 Electron paramagnetic resonance (EPR) experiments

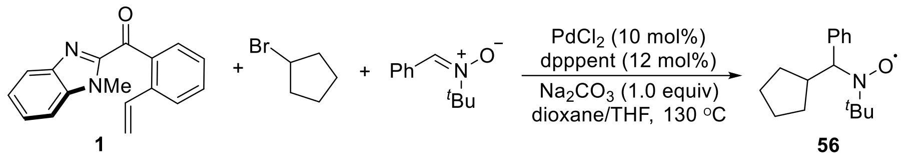

In an N_2-filled glovebox, an oven-dried 10 mL sealed tube equipped with a Teflon-coated magnetic stir bar was charged successively with ketone **1** (0.1 mmol), bromocyclopentane (0.15 mmol, 1.5 equiv), PBN (0.15 mmol, 1.5 equiv), $PdCl_2$ (0.01 mmol, 10 mol%), dpppent (0.012 mmol, 12 mol%), Na_2CO_3 (0.1 mmol, 1.0 equiv) and dioxane/THF (1.0 mL, 1:2). The tube then was sealed with a Teflon screw cap, moved out of the glovebox, and placed on a hotplate pre-heated to 130 °C with vigorous stirring for 40 min. Then the resulting reaction solution was analyzed by EPR at 130 °C. As shown in Figure 1, a strong EPR signal of **56** was observed.

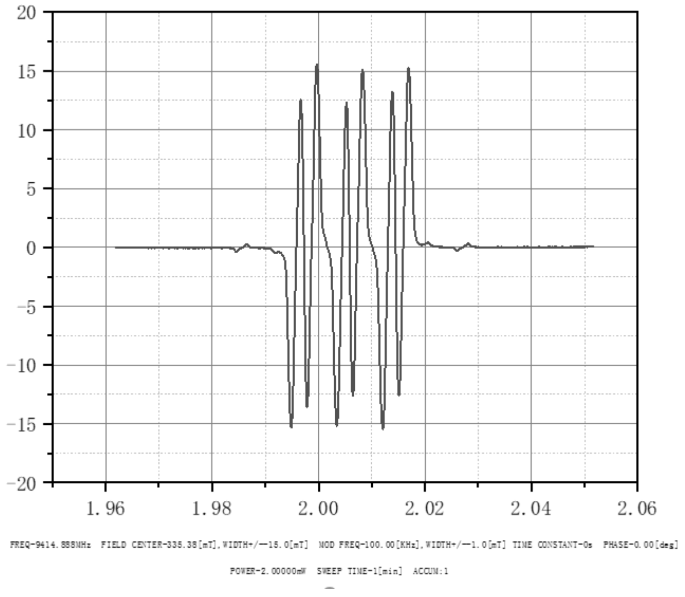

Supplementary Figure 1. The Electron Paramagnetic Resonance (EPR) Spectrum of a Mixture of ketone **1**, bromocyclopentane, PBN, $PdCl_2$, dpppent and Na_2CO_3.

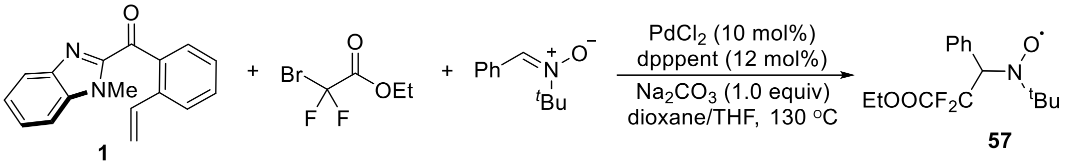

In an N_2-filled glovebox, an oven-dried 10 mL sealed tube equipped with a Teflon-coated magnetic stir bar was charged successively with ketone **1** (0.1 mmol), difluorobromoethyl ester (0.15 mmol, 1.5 equiv), PBN (0.15 mmol, 1.5 equiv), $PdCl_2$ (0.01 mmol, 10 mol%), dpppent (0.012 mmol, 12 mol%), Na_2CO_3 (0.1 mmol, 1.0 equiv) and dioxane/THF (1.0 mL, 1:2). The tube then was sealed with a Teflon screw cap, moved out of the glovebox, and placed on a hotplate pre-heated to 130 °C with vigorous stirring for 40 min. Then the resulting reaction solution was analyzed by EPR at 130 °C. As shown in Figure 2, a strong EPR signal of **57** was observed.

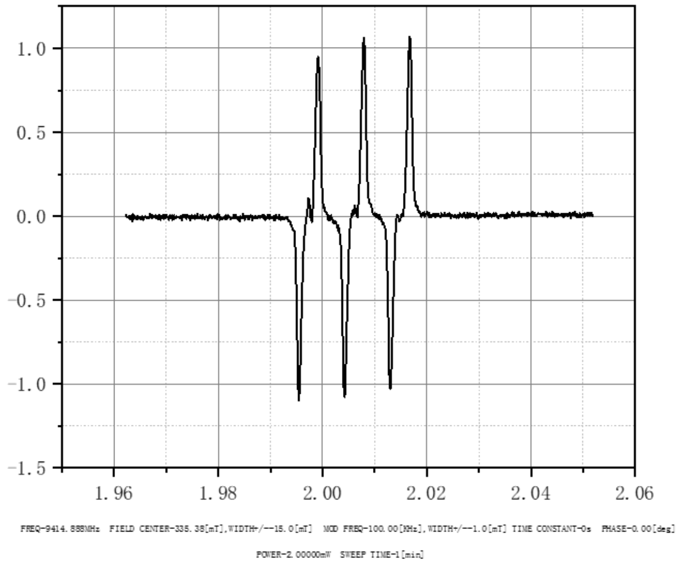

Supplementary Figure 2. The Electron Paramagnetic Resonance (EPR) Spectrum of a Mixture of ketone **1**, difluorobromoethyl ester, PBN, $PdCl_2$, dpppent and Na_2CO_3.

2.4.3 Deuterium labeling experiments

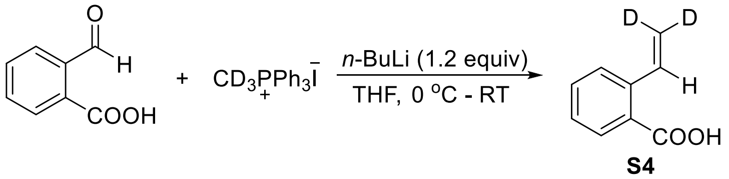

The mixture of d-methyltriphenylphosphonium iodide (2.44 g, 6.0 mmol), THF (5.0 mL) was added to a 25 mL three-neck flask under nitrogen atmosphere. *n*-BuLi (6.0 mmol, 2.5mol/L in hexane) was added in one portion to the reaction mixture at 0 °C and stirred for 30 min at this temperature. 2-Formylbenzoic acid (0.75 g, 5.0 mmol) in THF (5.0 mL) was added dropwise to the reaction mixture. The resulting mixture was warmed to room temperature and stirred for additional 2 h. Then the reaction was quenched by water and THF was removed under reduced pressure. The residue was filtered and extracted with petroleum ether. The combined organic layer was washed with brine and dried over anhydrous Na_2SO_4. After removing the solvent under reduced pressure, the crude product was purified by flash column chromatography on silica gel and eluted with petroleum ether to afford 2-(vinyl-2,2-d2)benzoic acid **S4**.

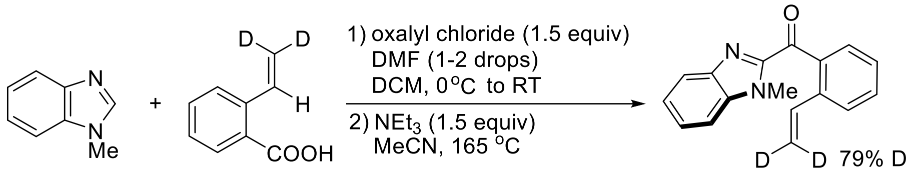

S4 **D-1**

S4 (4.0 mmol) was added to a round bottomed flask at 0 °C, drying CH_2Cl_2 (12.0 mL), oxalyl chloride (6.0 mmol) and 1-2 drops of DMF were added to the reaction mixture, and the reaction was carried out at room temperature for 6 h. After concentration, 1-methylbenzimidazole (2.7 mmol), triethylamine (4.0 mmol), acetonitrile (1.0 mL) was added and heated at 165 °C for 1 h, then 10.0 mL acetone was added to it, and the solution was poured into 7% hydrochloric acid (40.0 mL) under stirring. The mixture was stirred and refluxed with charcoal for at least 30 minutes. The filtrate was alkalized with 20% sodium hydroxide to separate compound **D-1**. The ^{1}H NMR analysis revealed that 79% D was incorporated into the molecule.

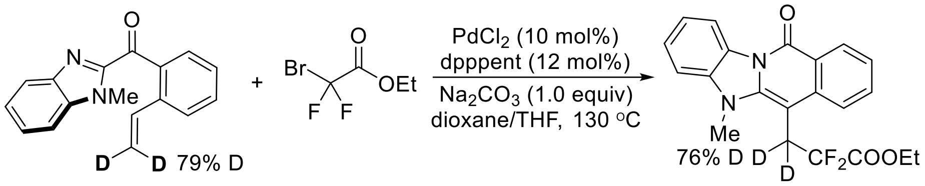

D-1 **D-2**, 72%

In an N_2-filled glovebox, an oven-dried 10 mL sealed tube equipped with a Teflon-coated magnetic stir bar was charged successively with deuterium labeling heteroaryl ketones **D-1** (0.1 mmol), difluorobromoethyl ester (0.15 mmol, 1.5 equiv), $PdCl_2$ (0.01 mmol, 10 mol%), dpppent (0.012 mmol, 12 mol%), Na_2CO_3 (0.1 mmol, 1.0 equiv) and dioxane/THF (1.0 mL, 1:2). The tube then was sealed with a Teflon screw cap, moved out of the glovebox, and placed on a hotplate pre-heated to 130 °C with vigorous stirring. After 24 h, cool the reaction to room temperature and purify **D-2** product by silica gel rapid chromatography.

2.4.4 Reaction using [Ph(PPh$_3$)$_2$PdBr] as a catalyst

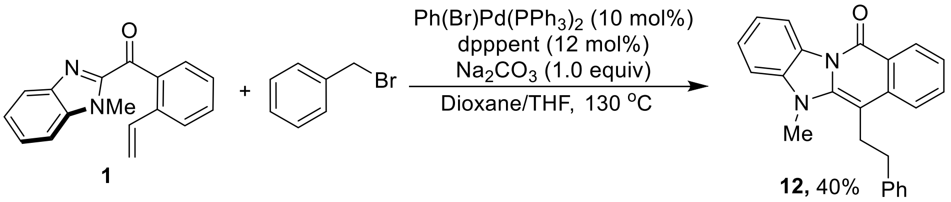

1 **12,** 40%

In an N_2-filled glovebox, an oven-dried 10 mL sealed tube equipped with a Teflon-coated magnetic stir bar was charged successively with deuterium labeling heteroaryl ketones **1** (0.1 mmol), benzyl bromide (0.15 mmol, 1.5 equiv), $Ph(PPh_3)_2PdBr$[1] (0.01 mmol, 10 mol%), dpppent (0.012 mmol, 12 mol%), Na_2CO_3 (0.1 mmol, 1.0 equiv) and dioxane/THF (1.0 mL, 1:2). The tube then was sealed with a Teflon screw cap, moved out of the glovebox, and placed on a hotplate pre-heated to 130 °C with vigorous stirring. After 36 h, cool the reaction to room temperature and purify by silica gel rapid chromatography.

2.4.5 X-ray photoelectron spectroscopy (XPS) experiments

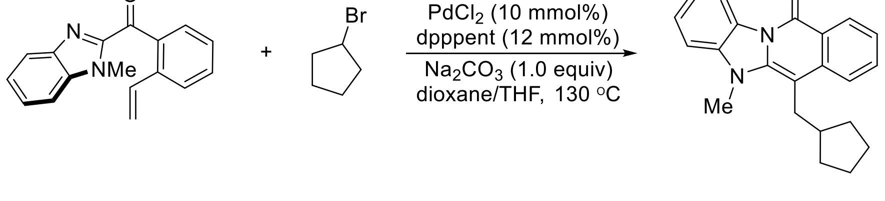

In an N_2-filled glovebox, an oven-dried 10 mL sealed tube equipped with a Teflon-coated magnetic stir bar was charged successively with ketone **1** (0.1 mmol), bromocyclopentane (0.15 mmol, 1.5 equiv), $PdCl_2$ (0.01 mmol, 10 mol%), dpppent (0.012 mmol, 12 mol%), Na_2CO_3 (0.1 mmol, 1.0 equiv) and dioxane/THF (1.0 ml, 1:2). The tube then was sealed with a Teflon screw cap, moved out of the glovebox, and placed on a hotplate pre-heated to 130 °C with vigorous stirring for 12 h. After 12 h, the reaction was concentrated under Ar, then the resulting powder was analyzed by the following procedure.

Experiment

The XPS spectra of the samples were measured with ULVAC-PHI, PHI5000VersaProbeIII. Stick the dried sample onto the sample, drag it into intro, and prepump it for about 12 hours under ultra-high vacuum. Then, move the sample into the analysis room. Use monochrome Al Kα (HV = 1486.6 eV) source, at 100 μ Spectra were obtained at 25w15kv. For the full spectrum (survey), 280 eV energy is used, for the narrow spectrum (narrow), 112 ev is used, and the time per step is 50 ms. the surface charge of the sample is neutralized by electron neutralization gun and argon ion gun at the same time. Mutipeak is used for mapping, and all spectra adopt Shirley type background and La (2.5, 2.5100) peak fitting is performed for the linetype, which is convoluted by symmetric Lorentz curve and Gaussian curve.

Results[2]

The Pd 3d spectra indicate the presence of three distinct Pd oxidation states, namely Pd(0), Pd(I) and Pd(II). (According to Alexander V. Naumkin, Anna Kraut-Vass, Stephen W. Gaarenstroom, Cedric J. Powell, NIST Standard Reference Database 20, Version 4.1 (http:/srdata.nist.gov/xps/), 2012.) This is also the case for their counterparts, which arise from spin-orbit-splitting and are shifted by ~5.3 eV towards higher binding energy. With 52.6 at.%, Pd(II) makes up the highest proportion of the three species. The other oxidation states, Pd(0) and Pd(I), make up 29.7 at.% and 17.7 at.%, respectively (Figure 3).

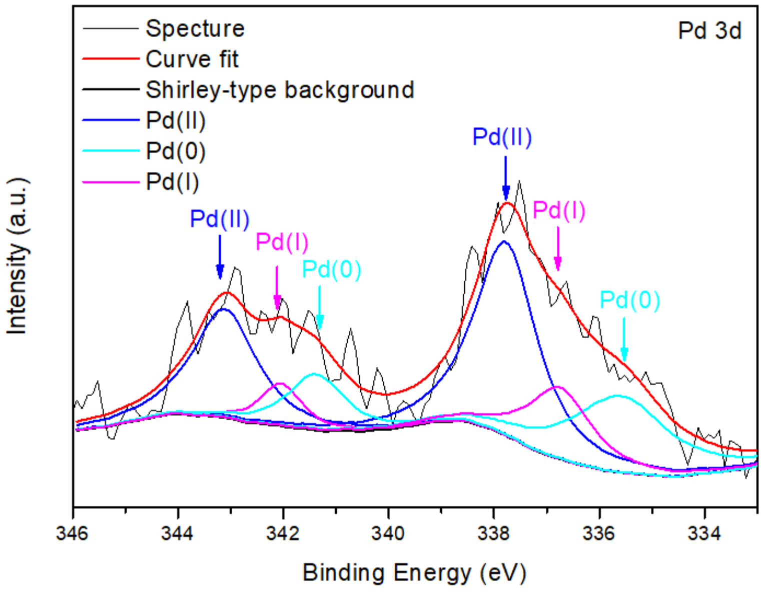

Species	At.%
Pd(0)	29.7
Pd(I)	17.7
Pd(II)	52.6

Supplementary Figure S3. Three distinct oxidation states of Pd(II), Pd(0), Pd(I).

2.5 Synthetic Applications

2.5.1 General procedure H for late-stage modification of matural products and drug derivatives

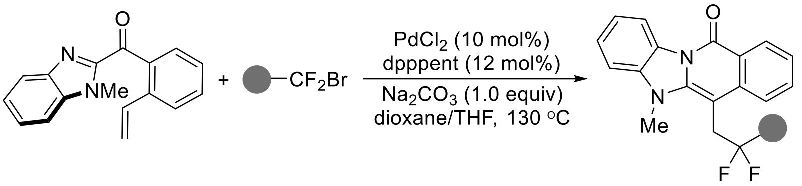

In an N_2-filled glovebox, an oven-dried 10 mL sealed tube equipped with a Teflon-coated magnetic stir bar was charged successively with heteroaryl ketones (0.1 mmol), difluoroalkyl bromide derived from natural products and drugs (0.15 mmol, 1.5 equiv), $PdCl_2$ (0.01 mmol, 10 mol%), dpppent (0.012 mmol, 12 mol%), Na_2CO_3 (0.1 mmol, 1.0 equiv) and dioxane/THF (1.0 mL, 1:2). The tube then was sealed with a Teflon screw cap, moved out of the glovebox, and placed on a hotplate pre-heated to 130 °C with vigorous stirring. After 24 h, cool the reaction to room temperature and purify by silica gel rapid chromatography.

2.5.2 Procedure for gram-scale synthesis

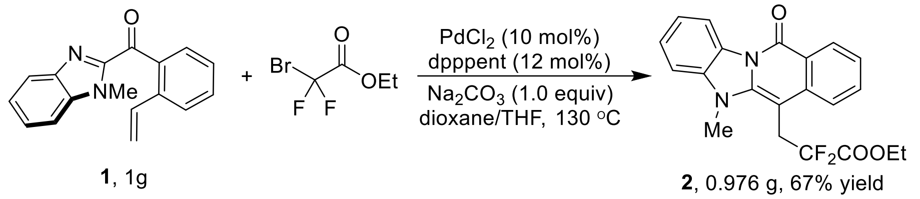

In an N_2-filled glovebox, an oven-dried 100 mL sealed tube equipped with a Teflon-coated magnetic stir bar was charged successively with heteroaryl ketones (1.0 g, 3.8 mmol), difluorobromoethyl ester (5.7 mmol, 1.5 equiv), $PdCl_2$ (0.38 mmol, 10 mol%), dpppent (0.456 mmol, 12 mol%), Na_2CO_3 (3.8 mmol, 1.0 equiv) and dioxane/THF (38.0 mL, 1:2). The tube then was sealed with a Teflon screw cap, moved out of the glovebox, and placed on a hotplate pre-heated to 130 °C with vigorous stirring. After 24 h, cool the reaction to room temperature and purify by silica gel rapid chromatography.

2.5.3 Procedure for derivatization of product

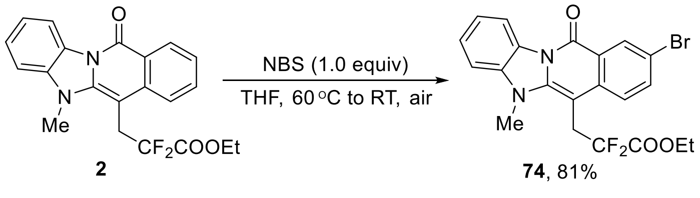

Product **2** (0.1 mmol), NBS (0.1 mmol) and THF (0.3 mL) was added to the sealed tube with magneton. After stirring at 60 °C for 4 h, the reaction was transferred to room temperature and reacted overnight. The crude reaction mixture was loaded directly onto a silica gel column and purification with petroleum ether and ethyl acetate to afford compound **74** in 81% yield.[3]

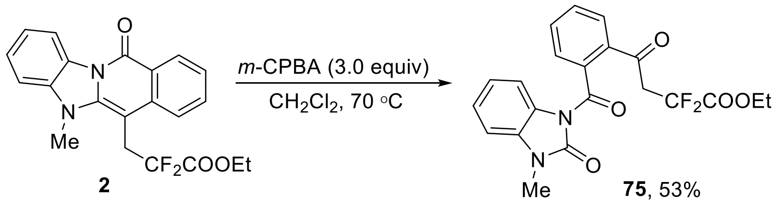

Product **2** (0.1 mmol), *m*-CPBA (0.3 mmol) and CH_2Cl_2 was added to the sealed tube with magneton at 70 °C. After 3 h,

The crude reaction mixture was loaded directly onto a silica gel column and purification with petroleum ether and ethyl acetate to afford compound **75** in 53% yield.

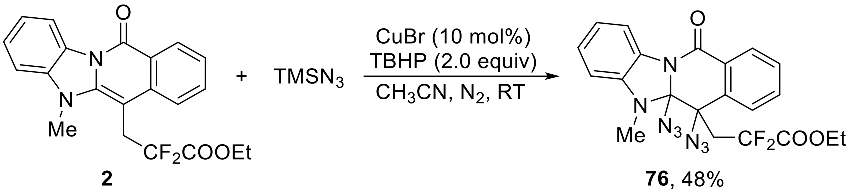

Product **2** (0.1 mmol) and CuBr (0.01 mmol, 10 mol%) was added to the sealed tube with magneton, under nitrogen atmosphere added $TMSN_3$ (0.2 mmol), TBHP (70% solution in water, 0.2 mmol), MeCN (1.0 mL). The solution was stirred at room temperature. After 2 h, the crude reaction mixture was loaded directly onto a silica gel column and purification with petroleum ether and ethyl acetate to afford compound **76** in 48% yield.[4]

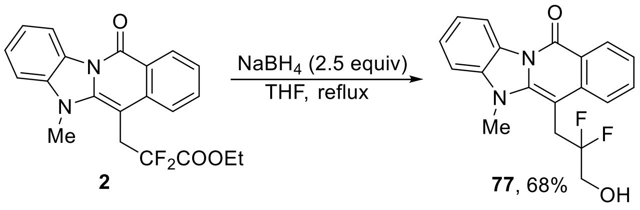

Product **2** (0.2 mmol) and THF (4.0 mL) was added to round bottomed flask, added $NaBH_4$ (0.5 mmol) and reflux. After 3 h, the crude reaction mixture was loaded directly onto a silica gel column and purification with petroleum ether and ethyl acetate to afford compound **77** in 68% yield.

3. Supplementary Spectra

3.1 X-Ray Crystallography

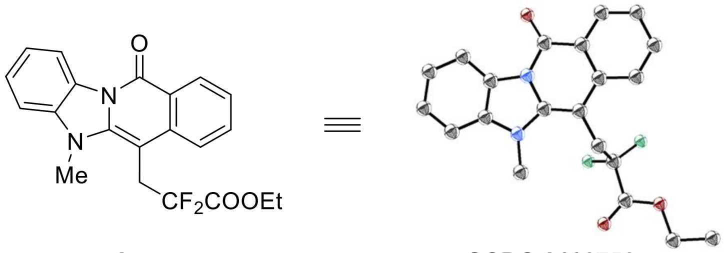

2 CCDC 2116750

Supplementary Table 6 Crystal data and structure refinement for 2

Empirical formula	$C_{21}H_{18}F_2N_2O_3$
Formula weight	384.37
Temperature/K	150(2)
Crystal system	Triclinic
Space group	P -1
a/Å	8.3124(5)
b/Å	9.7560(6)
c/Å	11.7303(7)
α/°	74.635(2)
β/°	76.747(2)
γ/°	77.293(2)
Volume/$Å^3$	879.91(9)
Z	2
Density (calculated) g/cm^3	1.451
μ/mm^{-1}	0.601
F(000)	400.0
Crystal size/mm^3	0.32 × 0.25 × 0.17
Radiation	Ga Kα (λ = 1.34139)
2Θ range for data collection	3.46 to 54.90°
Index ranges	-9 ≤ h ≤ 10, -11 ≤ k ≤ 11, -14≤ l ≤14
Reflections collected	16977
Independent reflections	3332 [R_{int} = 0.0304]
Data/restraints/parameters	3332/0/255
Goodness-of-fit on F^2	1.090
Final R indexes [I>=2σ (I)]	R_1 = 0.0330, wR_2 = 0.0927
Final R indexes [all data]	R_1 = 0.0349, wR_2 = 0.0941
Largest diff. peak/hole / e $Å^{-3}$	0.217/-0.200

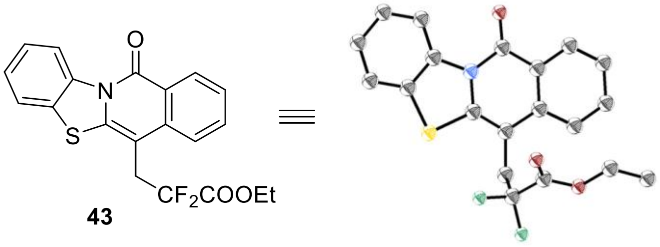

43

CCDC 2116753

Supplementary Table 7 Crystal data and structure refinement for 43

Empirical formula	$C_{20}H_{15}FNO_3S$
Formula weight	387.39
Temperature/K	150(2)
Crystal system	Triclinic
Space group	P -1
a/Å	5.8181(3)
b/Å	10.0244(4)
c/Å	14.4406(6)
α/°	84.4850(10)
β/°	87.3400(10)
γ/°	83.0880(10)
Volume/$Å^3$	831.71(6)
Z	2
Density (calculated) g/cm^3	1.547
μ/mm^{-1}	1.388
F(000)	400.0
Crystal size/mm^3	0.25 × 0.19 × 0.15
Radiation	Ga Kα (λ = 1.34139)
2Θ range for data collection	3.881 to 54.938°
Index ranges	$-6 \le h \le 7$, $-12 \le k \le 12$, $-17 \le l \le 17$
Reflections collected	13058
Independent reflections	3131 [R_{int} = 0.0357]
Data/restraints/parameters	3131/0/245
Goodness-of-fit on F^2	1.000
Final R indexes [I>=2σ (I)]	R_1 = 0.0297, wR_2 = 0.0824
Final R indexes [all data]	R_1 = 0.0318, wR_2 = 0.0839
Largest diff. peak/hole / e $Å^{-3}$	0.302/-0.272

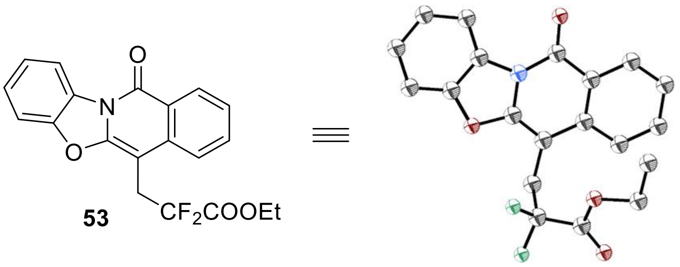

CCDC 2116752

Supplementary Table 8 Crystal data and structure refinement for 53

Empirical formula	$C_{20}H_{15}F_2NO_4$
Formula weight	371.33
Temperature/K	200(2)
Crystal system	Triclinic
Space group	P -1
a/Å	8.9667(11)
b/Å	11.4541(14)
c/Å	17.087(2)
α/°	99.994(4)
β/°	98.027(4)
γ/°	100.974(4)
Volume/$Å^3$	1669.5(4)
Z	4
Density (calculated) g/cm^3	1.477
μ/mm^{-1}	0.64
F(000)	768
Crystal size/mm^3	0.28 × 0.26 × 0.21
Radiation	Ga Kα (λ = 1.34139)
2Θ range for data collection	5.01 to 54.99°
Index ranges	$-10 \le h \le 10$, $-13 \le k \le 13$, $-20 \le l \le 20$
Reflections collected	20191
Independent reflections	6122 [R_{int} = 0.0591]
Data/restraints/parameters	6122/0/490
Goodness-of-fit on F^2	1.052
Final R indexes [I>=2σ (I)]	R_1 = 0.1413, wR_2 = 0.3398
Final R indexes [all data]	R_1 = 0.1448, wR_2 = 0.3493
Largest diff. peak/hole / e $Å^{-3}$	0.684/-0.943

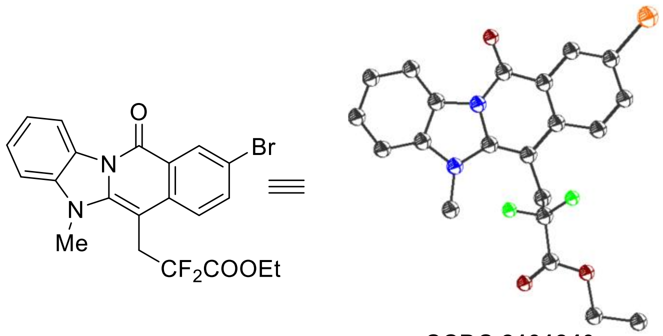

CCDC 2131840

Supplementary Table 9 Crystal data and structure refinement for 74

Empirical formula	$C_{22}H_{17}BrF_2N_2O_3$
Formula weight	463.27
Temperature/K	140.0
Crystal system	Triclinic
Space group	P -1
a/Å	8.2847(13)
b/Å	9.8931(14)
c/Å	12.3085(19)
α/°	71.557(5)
β/°	80.756(6)
γ/°	77.848(5)
Volume/Å^3	930.7(2)
Z	2
Density (calculated) g/cm^3	1.650
μ/mm^{-1}	2.253
F(000)	468
Crystal size/mm^3	0.5 × 0.5 × 0.1
Radiation	Mo Kα (λ = 0.71073)
2Θ range for data collection	1.753 to 25.681°
Index ranges	$-10 \le h \le 10$, $-12 \le k \le 11$, $-14 \le l \le 14$
Reflections collected	15543
Independent reflections	3496 [R_{int} = 0.0549]
Data/restraints/parameters	3496/0/264
Goodness-of-fit on F^2	1.064
Final R indexes [I>=2σ (I)]	R_1 = 0.0624, wR_2 = 0.1684
Final R indexes [all data]	R_1 = 0.0804, wR_2 = 0.1827
Largest diff. peak/hole / e Å^{-3}	2.300/-1.121

3.2 Characterization of Starting Materials

(1-methyl-1*H*-benzo[*d*]imidazol-2-yl)(2-vinylphenyl)methanone

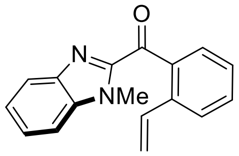

Prepared from **GPA**. Afforded the desired product as a White solid, Mp = 126–127 °C. ^{1}H NMR (400 MHz, $CDCl_3$) δ = 7.88–7.85 (td, *J* = 1.2 Hz, *J* = 8.4 Hz, 1H), 7.80–7.78 (m, 1H), 7.65–7.62 (dd, *J* = 1.2 Hz, *J* = 8.0 Hz, 1H), 7.51–7.47 (td, *J* = 1.2 Hz, *J* = 7.6 Hz, 1H), 7.44–7.38 (m, 2H), 7.37–7.30 (m, 2H), 7.07–6.99 (m, 1H), 5.69–5.65 (dd, *J* = 1.2 Hz, *J* = 17.2 Hz, 1H), 5.29–5.26 (dd, *J* = 1.2 Hz, *J* = 11.2 Hz, 1H), 4.13 (s, 3H). ^{13}C NMR (100 MHz, $CDCl_3$) δ = 189.17, 146.84, 141.67, 138.40, 136.48, 136.17, 134.90, 131.62, 131.00, 126.79, 126.66, 125.71, 123.45, 122.00, 116.53, 110.25, 32.05. HRMS (ESI) m/z calculated for $C_{17}H_{15}N_2O$ $[M+H]^+$ 263.1179, found 263.1184.

(1,5,6-trimethyl-1*H*-benzo[*d*]imidazol-2-yl)(2-vinylphenyl)methanone

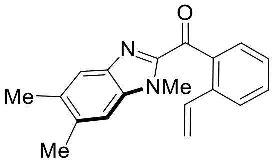

Prepared from **GPA**. Afforded the desired product as a pale yellow solid, Mp = 226–227 °C. ^{1}H NMR (400 MHz, $CDCl_3$) δ = 7.78–7.76 (m, 1H), 7.65–7.61 (m, 2H), 7.528–7.48 (m, 1H), 7.38–7.34 (td, *J* = 1.2 Hz, *J* = 7.6 Hz, 1H), 7.23 (s, 1H), 7.04–7.97 (m, 1H), 5.69–5.65 (dd, *J* = 1.2 Hz, *J* = 17.2 Hz, 1H), 5.28–5.25 (dd, *J* = 1.2 Hz, *J* = 10.8 Hz, 1H), 4.15 (s, 3H), 2.43 (s, 3H), 2.37 (s, 3H). ^{13}C NMR (100 MHz, $CDCl_3$) δ = 189.32, 146.38, 140.69, 138.45, 136.69, 136.14, 135.50, 135.14, 133.16, 131.57, 131.05, 126.93, 126.76, 121.74, 116.48, 110.19, 32.27, 20.97, 20.39. HRMS (ESI) m/z calculated for $C_{19}H_{19}N_2O$ $[M+H]^+$ 291.1492, found 291.1498.

(5-methoxy-1-methyl-1*H*-benzo[*d*]imidazol-2-yl)(2-vinylphenyl)methanone

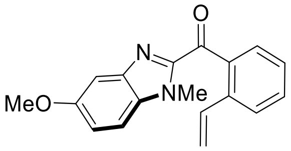

Prepared from **GPA**. Afforded the desired product as a yellow solid, Mp = 134–135 °C. ^{1}H NMR (400 MHz, $CDCl_3$) δ = 7.79–7.77 (m, 1H), 7.67–7.65 (m, 1H), 7.54–7.49 (m, 1H), 7.39–7.35 (m, 2H), 7.28–7.27 (m, 1H), 7.13–7.10 (dd, *J* = 2.4 Hz, *J* = 8.8 Hz, 1H), 7.04–6.97 (dd, *J* = 10.8 Hz, *J* = 17.2 Hz, 1H), 5.72–5.67 (dd, *J* = 1.2 Hz, *J* = 17.6 Hz, 1H), 5.30–5.27 (dd, *J* = 1.2 Hz, *J* = 11.2 Hz, 1H), 4.18 (s, 3H), 3.83 (s, 3H). ^{13}C NMR (100 MHz, $CDCl_3$) δ = 189.15, 157.09, 146.93, 142.60, 138.35, 136.58, 135.02, 131.60, 131.56, 130.90, 126.91, 126.73, 117.81, 116.56, 110.93, 102.17, 77.00, 55.57, 32.40. HRMS (ESI) m/z calculated for $C_{18}H_{17}N_2O_2$ $[M+H]^+$ 293.1285, found 293.1288.

(5-chloro-1-methyl-1*H*-benzo[*d*]imidazol-2-yl)(2-vinylphenyl)methanone

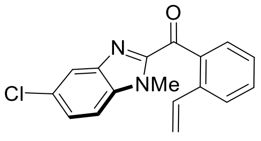

Prepared from **GPA**. Afforded the desired product as a pale yellow solid, Mp = 140–141 °C. ^{1}H NMR (400 MHz, $CDCl_3$) δ = 8.03 (dt, *J* = 1.8 Hz, *J* = 0.9 Hz, 1H), 7.88 (dt, *J* = 8.2 Hz, *J* = 1.0 Hz, 1H), 7.76 (d, *J* = 1.4 Hz, 2H), 7.54–7.45 (m,

2H), 7.37 (ddd, J = 8.2 Hz, J = 5.9 Hz, J = 2.2 Hz, 1H), 6.98 (dd, J = 17.3 Hz, J = 11.0 Hz, 1H), 5.77 (dd, J = 17.4 Hz, J = 0.9 Hz, 1H), 5.39 (dd, J = 11.0 Hz, J = 0.9 Hz, 1H), 4.23 (s, 3H). ^{13}C NMR (100 MHz, $CDCl_3$) δ = 189.09, 147.92, 142.38, 138.83, 136.78, 135.92, 135.21, 135.06, 132.11, 131.22, 129.23, 127.04, 126.51, 121.63, 116.95, 111.33, 32.45. HRMS (ESI) m/z calculated for $C_{17}H_{14}ClN_2O$ $[M+H]^+$ 297.0789, found 297.0785.

(1-methyl-5-(trifluoromethyl)-1H-benzo[d]imidazol-2-yl)(2-vinylphenyl)methanone

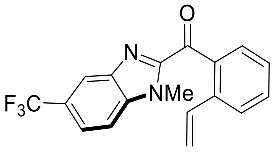

Prepared from **GPA**. Afforded the desired product as a pale yellow solid, Mp = 138–139 °C. ^{1}H NMR (400 MHz, $CDCl_3$) δ = 8.18 (s, 1H), 7.80 (d, J = 7.7 Hz, 1H), 7.68 (t, J = 8.5 Hz, 2H), 7.57 (dd, J = 13.4 Hz, J = 8.1 Hz, 2H), 7.40 (t, J = 7.6 Hz, 1H), 7.02 (dd, J = 17.4 Hz, J = 10.9 Hz, 1H), 5.68 (d, J = 17.3 Hz, 1H), 5.31 (d, J = 11.0 Hz, 1H), 4.22 (s, 3H). ^{13}C NMR (100 MHz, $CDCl_3$) δ = 189.27, 148.90, 141.19, 139.09, 138.47, 135.91, 135.22, 132.40, 131.38, 127.29, 127.22, 126.14(q, J = 33.0 Hz), 123.22, 122.54 (d, J = 3.5 Hz), 120.22 (d, J = 4.4 Hz)., 117.29, 111.29, 32.69. ^{19}F NMR (376 MHz, $CDCl_3$) δ = -61.00. HRMS (ESI) m/z calculated for $C_{18}H_{14}F_3N_2O$ $[M+H]^+$ 331.1053, found 331.1036.

1-methyl-2-(2-vinylbenzoyl)-1H-benzo[d]imidazole-5-carbonitrile

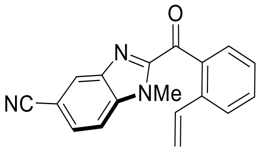

Prepared from **GPA**. Afforded the desired product as a pale yellow solid, Mp = 187–188 °C. ^{1}H NMR (400 MHz, $CDCl_3$) δ = 8.24 (s, 1H), 7.83 (d, J = 7.8 Hz, 1H), 7.70 (dd, J = 8.1 Hz, J = 5.7 Hz, 2H), 7.62 (d, J = 8.3 Hz, 2H), 7.44 (t, J = 7.6 Hz, 1H), 7.05 (dd, J = 17.4 Hz, J = 11.0 Hz, 1H), 5.71 (d, J = 17.4 Hz, 1H), 5.34 (d, J = 11.0 Hz, 1H), 4.24 (s, 3H). ^{13}C NMR (100 MHz, $CDCl_3$) δ = 188.81, 149.18, 141.07, 139.02, 138.91, 135.39, 134.96, 132.43, 131.23, 128.27, 127.49, 127.19, 127.07, 119.18, 117.29, 111.76, 106.97, 32.57. HRMS (ESI) m/z calculated for $C_{18}H_{13}N_3ONa$ $[M+Na]^+$ 310.0951, found 310.0943.

methyl 1-methyl-2-(2-vinylbenzoyl)-1*H*-benzo[*d*]imidazole-5-carboxylate

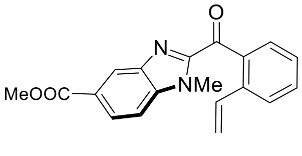

Prepared from **GPA**. Afforded the desired product as a White solid, Mp = 212–213 °C. ^{1}H NMR (400 MHz, $CDCl_3$) δ = 8.66 (s, 1H), 8.21 (dd, J = 8.7 Hz, J =1.6 Hz, 1H), 7.86 (d, J = 7.7 Hz, 1H), 7.72 (d, J = 7.9 Hz, 1H), 7.66–7.54 (m, 2H), 7.45 (t, J = 7.6 Hz, 1H), 7.07 (dd, J = 17.4 Hz, J = 10.9 Hz, 1H), 5.73 (d, J = 17.3 Hz, 1H), 5.35 (d, J = 10.9 Hz, 1H), 4.26 (s, 3H), 3.99 (s, 3H). ^{13}C NMR (100 MHz, $CDCl_3$) δ = 189.13, 167.09, 148.64, 141.35, 139.55, 138.93, 135.86, 135.12, 132.18, 131.30, 127.11, 127.06, 126.90, 125.89, 124.85, 117.02, 110.24, 77.32, 32.56. HRMS (ESI) m/z calculated for $C_{19}H_{16}N_2O_3Na$ $[M+Na]^+$ 343.1053, found 343.1033.

(1-methyl-1*H*-benzo[*d*]imidazol-2-yl)(4-methyl-2-vinylphenyl)methanone

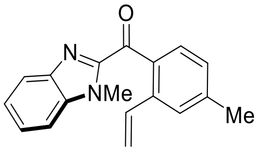

Prepared from **GPA.** Afforded the desired product as a White solid, Mp = 193–194 °C. ^{1}H NMR (400 MHz, $CDCl_3$) δ = 7.88 (d, *J* = 8.2 Hz, 1H), 7.74 (d, *J* = 7.9 Hz, 1H), 7.53–7.39 (m, 3H), 7.34 (m, 1H), 7.19 (dd, *J* = 8.0 Hz, *J* =1.7 Hz, 1H), 7.05 (dd, *J* = 17.5 Hz, *J* = 10.9 Hz, 1H), 5.67 (d, *J* = 17.0 Hz, 1H), 5.28 (d, *J* = 11.3 Hz, 1H), 4.17 (s, 3H), 2.43 (s, 3H). ^{13}C NMR (100 MHz, $CDCl_3$) δ = 188.96, 147.34, 142.60, 141.80, 139.04, 136.62, 135.43, 133.52, 131.79, 127.78, 127.69, 125.73, 123.54, 122.16, 116.39, 110.35, 32.19, 21.65. HRMS (ESI) m/z calculated for $C_{18}H_{17}N_2O$ $[M+H]^+$ 277.1335, found 277.1325.

(1-methyl-1*H*-benzo[*d*]imidazol-2-yl)(3-methyl-2-vinylphenyl)methanone

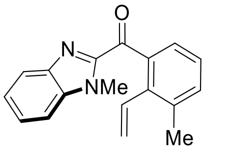

Prepared from **GPA**. Afforded the desired product as a White solid, Mp = 138–139 °C. ^{1}H NMR (400 MHz, $CDCl_3$) δ = 7.84–7.81 (m, 1H), 7.53–7.51 (dd, *J* = 2.0 Hz, *J* = 7.2 Hz, 1H), 7.44–7.38 (m, 2H), 7.36–7.28 (m, 3H), 6.75–6.68 (dd, *J* = 11.2 Hz, *J* = 17.6 Hz, 1H), 5.14–5.11 (dd, *J* = 2.0 Hz, *J* = 11.2 Hz, 1H), 5.06–5.01 (m, 1H), 4.09 (s, 3H), 2.31 (s, 3H). ^{13}C NMR (100 MHz, $CDCl_3$) δ = 191.60, 148.09, 141.75, 138.48, 138.25, 136.35, 136.31, 136.22, 132.80, 126.96, 126.82, 125.34, 123.26, 121.92, 119.35, 110.16, 31.60, 19.71. HRMS (ESI) m/z calculated for $C_{18}H_{17}N_2O$ $[M+H]^+$ 277.1335, found 277.1331.

(5-methoxy-2-vinylphenyl)(1-methyl-1*H*-benzo[*d*]imidazol-2-yl)methanone

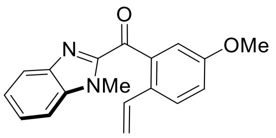

Prepared from **GPA**. Afforded the desired product as a yellow solid, Mp = 148–149 °C. ^{1}H NMR (400 MHz, $CDCl_3$) δ = 7.90–7.88 (m, 1H), 7.60 (d, *J* = 8.8 Hz, 1H), 7.51–7.44 (m, 2H), 7.38–7.34 (m, 1H), 7.29 (d, *J* = 2.8 Hz, 1H), 7.09–7.06 (dd, *J* = 2.8 Hz, *J* = 8.8 Hz, 1H), 6.93–6.86 (dd, *J* = 10.8 Hz, *J* = 17.6 Hz, 1H), 5.59–5.55 (dd, *J* = 1.2 Hz, *J* = 17.2 Hz, 1H), 5.19–5.16 (dd, *J* = 1.2 Hz, *J* = 10.8 Hz, 1H), 4.21 (s, 3H), 3.82 (s, 3H). ^{13}C NMR (100 MHz, $CDCl_3$) δ = 189.34, 158.26, 146.89, 141.89, 137.42, 136.70, 134.39, 131.14, 128.07, 125.98, 123.66, 122.33, 118.03, 115.54, 114.95, 110.40, 55.43, 32.27. HRMS (ESI) m/z calculated for $C_{18}H_{17}N_2O_2$ $[M+H]^+$ 293.1285, found 293.1284.

(4,5-dimethoxy-2-vinylphenyl)(1-methyl-1*H*-benzo[*d*]imidazol-2-yl)methanone

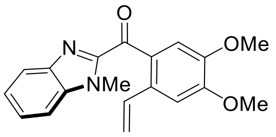

Prepared from **GPA**. Afforded the desired product as a yellow solid, Mp = 198–199 °C. ^{1}H NMR (400 MHz, $CDCl_3$) δ = 7.90 (d, *J* = 8.0 Hz, 1H), 7.51–7.45 (m, 3H), 7.39–7.35 (m, 1H), 7.11 (s, 1H), 7.08–7.01 (m, 1H), 5.61–5.57 (m, 1H), 5.26–5.23 (m, 1H), 4.18 (s, 3H), 3.99 (s, 3H), 3.87 (s, 3H). ^{13}C NMR (100 MHz, $CDCl_3$) δ = 187.77, 152.16, 147.60, 147.55, 141.78, 136.60, 135.36, 134.04, 128.66, 125.69, 123.56, 122.09, 115.32, 114.48, 110.37, 109.34, 56.00, 32.17.

HRMS (ESI) m/z calculated for $C_{19}H_{19}N_2O_3$ $[M+H]^+$ 323.1390, found 323.1379.

(4-fluoro-2-vinylphenyl)(1-methyl-1*H*-benzo[*d*]imidazol-2-yl)methanone

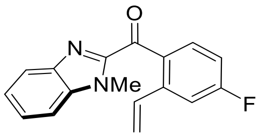

Prepared from **GPA**. Afforded the desired product as a White solid, Mp = 166–167 °C.^{1}H NMR (400 MHz, $CDCl_3$) δ = 7.89–7.86 (m, 2H), 7.46–7.42 (m, 2H), 7.37–7.31 (m, 2H), 7.08–7.00 (m, 2H), 5.72–5.68 (dd, *J* = 1.2 Hz, *J* = 17.2 Hz, 1H), 5.36–5.33 (m, 1H), 4.16 (s, 3H). ^{13}C NMR (100 MHz, $CDCl_3$) δ = 187.76, 165.88 (d, *J* = 252.0 Hz), 146.80, 142.06 (d, *J* = 9.0 Hz), 141.72, 136.63, 134.23, 134.14, 132.38 (d, *J* = 2.0 Hz), 125.98, 123.72, 122.13, 117.70, 114.10 (d, *J* = 22.0 Hz), 113.74 (d, *J* = 22.0 Hz), 110.38, 32.21. ^{19}F NMR (376 MHz, $CDCl_3$) δ = -106.78 (q, *J* = 8.7, *J* = 8.2 Hz, 1F). HRMS (ESI) m/z calculated for $C_{17}H_{13}FN_2ONa$ $[M+Na]^+$ 303.0904, found 303.0905.

(1-methyl-1*H*-benzo[*d*]imidazol-2-yl)(5-(trifluoromethyl)-2-vinylphenyl)methanone

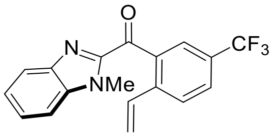

Prepared from **GPA**. Afforded the desired product as a White solid, Mp = 142–143 °C. ^{1}H NMR (400 MHz, $CDCl_3$) δ = 8.04 (d, *J* = 1.3 Hz, 1H), 7.88 (dt, *J* = 8.2, *J* = 1.0 Hz, 1H), 7.76 (d, *J* = 1.5 Hz, 2H), 7.55–7.44 (m, 2H), 7.37 (ddd, *J* = 8.2, *J* = 5.9 Hz, *J* = 2.3 Hz, 1H), 6.98 (dd, *J* = 17.4 Hz, *J* = 11.0 Hz, 1H), 5.77 (dd, *J* = 17.4 Hz, *J* = 0.9 Hz, 1H), 5.39 (dd, *J* = 11.0 Hz, *J* = 0.9 Hz, 1H), 4.22 (s, 3H). ^{13}C NMR (100 MHz, $CDCl_3$) δ = 188.29, 146.24, 141.94, 141.62, 136.89 (d, *J* = 13.8 Hz), 133.90, 128.99 (q, *J* = 32.9 Hz), 128.03 (d, *J* = 3.9 Hz), 127.77 (d, *J* = 4.1 Hz)., 127.26, 126.44, 125.26, 123.96, 122.46, 118.97, 110.49, 32.31. ^{19}F NMR (376 MHz, $CDCl_3$) δ = -62.41. HRMS (ESI) m/z calculated for $C_{18}H_{14}F_3N_2O$ $[M+H]^+$ 331.1053, found 331.1036.

(1-isopropyl-1*H*-benzo[*d*]imidazol-2-yl)(2-vinylphenyl)methanone

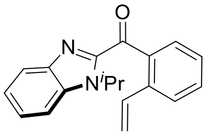

Prepared from **GPA**. Afforded the desired product as a White solid, Mp = 71–72 °C. ^{1}H NMR (400 MHz, $CDCl_3$) δ = 7.88–7.86 (m, 1H), 7.72–7.69 (dd, *J* = 1.6 Hz, *J* = 7.6 Hz, 1H), 7.67–7.64 (m, 1H), 7.62–7.59 (dd, *J* = 1.2 Hz, *J* = 8.0 Hz, 1H), 7.50–7.46 (m, 1H), 7.36–7.27 (m, 3H), 7.12–7.05 (m, 1H), 5.67–5.62 (dd, *J* = 1.2 Hz, *J* = 17.2 Hz, 1H), 5.59–5.52 (m, 1H), 5.29–5.26 (dd, *J* = 1.2 Hz, *J* = 10.8 Hz, 1H), 1.69 (s, 3H), 1.68 (s, 3H). ^{13}C NMR (100 MHz, $CDCl_3$) δ = 189.51, 147.41, 142.23, 138.72, 136.29, 135.01, 134.16, 131.76, 131.06, 126.75, 126.72, 124.75, 122.82, 122.04, 116.58, 112.72, 48.72, 21.23. HRMS (ESI) m/z calculated for $C_{19}H_{19}N_2O$ $[M+H]^+$ 291.1492, found 291.1490.

(1-benzyl-1*H*-benzo[*d*]imidazol-2-yl)(2-vinylphenyl)methanone

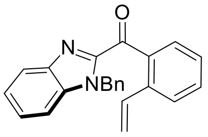

Prepared from **GPA**. Afforded the desired product as a pale yellow solid, Mp = 146–147 °C. ^{1}H NMR (400 MHz, $CDCl_3$) δ = 7.91–7.89 (m, 1H), 7.71–7.69 (m, 1H), 7.63–7.61 (dd, *J* = 1.2 Hz, *J* = 8.0 Hz, 1H), 7.51–7.47 (m, 1H), 7.46–7.44 (m,

1H), 7.41–7.37 (m, 1H), 7.36–7.32 (m, 2H), 7.29–7.23 (m, 3H), 7.22–7.18 (m, 2H), 6.93–6.86 (m, 1H), 5.89 (s, 2H), 5.64–5.59 (dd, *J* = 1.2 Hz, *J* = 17.2 Hz, 1H), 5.22–5.19 (dd, *J* = 1.2 Hz, *J* = 11.2 Hz, 1H). ^{13}C NMR (100 MHz, $CDCl_3$) δ = 189.52, 146.84, 141.95, 138.46, 136.52, 136.43, 136.28, 134.85, 131.74, 130.88, 128.71, 127.75, 126.94, 126.86, 126.74, 126.15, 123.77, 122.30, 116.76, 110.99, 48.51. HRMS (ESI) m/z calculated for $C_{23}H_{19}N_2O$ $[M+H]^+$ 339.1492, found 339.1486.

(1-methyl-4,5-diphenyl-1*H*-imidazol-2-yl)(2-vinylphenyl)methanone

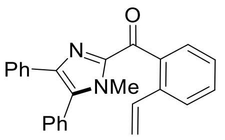

Prepared from **GPA**. Afforded the desired product as a pale yellow solid, Mp = 171–172 °C. ^{1}H NMR (400 MHz, $CDCl_3$) δ = 7.95–7.92 (m, 1H), 7.68 (d, *J* = 8.0 Hz, 1H), 7.53–7.48 (m, 4H), 7.42–7.36 (m, 5H), 7.19–7.14 (m, 3H), 7.12–7.05 (m, 1H), 5.75–5.70 (m, 1H), 5.33–5.29 (m, 1H), 3.89 (s, 3H). ^{13}C NMR (100 MHz, $CDCl_3$) δ = 187.01, 143.02, 139.58, 138.30, 137.08, 135.45, 135.13, 133.54, 131.27, 131.04, 130.60, 129.38, 129.17, 128.11, 127.20, 127.04, 126.67, 126.48, 115.89, 34.27. HRMS (ESI) m/z calculated for $C_{25}H_{21}N_2O$ $[M+H]^+$ 365.1648, found 365.1637.

(1-methyl-4-phenyl-1*H*-imidazol-2-yl)(2-vinylphenyl)methanone

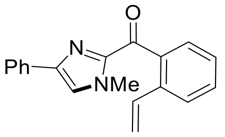

Prepared from **GPA**. Afforded the desired product as a yellow solid, Mp = 184–185 °C. ^{1}H NMR (400 MHz, $CDCl_3$) δ = 7.89–7.88 (m, 1H), 7.74–7.71 (m, 2H), 7.66–7.63 (dd, *J* = 1.2 Hz, *J* = 8.0 Hz, 1H), 7.51–7.46 (m, 1H), 7.38–7.32 (m, 4H), 7.27–7.22 (m, 1H), 7.07–6.99 (m, 1H), 5.71–5.67 (m, 1H), 5.29–5.27 (dd, *J* = 1.2 Hz, *J* = 10.8 Hz, 1H), 4.09 (s, 3H). ^{13}C NMR (100 MHz, $CDCl_3$) δ = 186.79, 143.27, 141.92, 138.27, 136.64, 135.37, 133.08, 131.28, 131.07, 128.54, 127.45, 126.62, 126.46, 125.17, 123.13, 115.91, 36.56. HRMS (ESI) m/z calculated for $C_{19}H_{16}N_2ONa$ $[M+Na]^+$ 311.1155, found 311.1150.

(1-methyl-1*H*-imidazol-2-yl)(2-vinylphenyl)methanone

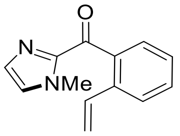

Prepared from **GPA**. Afforded the desired product as a yellow solid, Mp = 104–105 °C. ^{1}H NMR (400 MHz, $CDCl_3$) δ 7.64 (m, 2H), 7.50–7.38 (m, 1H), 7.32 (td, *J* = 7.5 Hz, *J* = 1.3 Hz, 1H), 7.16 (d, *J* = 0.9 Hz, 1H), 7.08 (d, *J* = 1.0 Hz, 1H), 6.91 (dd, *J* = 17.4 Hz, *J* = 11.0 Hz, 1H), 5.67 (dd, *J* = 17.4 Hz, *J* =1.2 Hz, 1H), 5.25 (dd, *J* = 11.0 Hz, *J* =1.2 Hz, 1H), 4.08 (s, 3H). ^{13}C NMR (100 MHz, $CDCl_3$) δ = 187.22, 143.45, 137.33, 136.93, 134.72, 130.78, 129.89, 129.79, 127.07, 126.75, 126.21, 116.05, 36.24. HRMS (ESI) m/z calculated for $C_{13}H_{12}N_2ONa$ $[M+Na]^+$ 235.0842, found 235.0841.

(1-methyl-4,5-di-p-tolyl-1*H*-imidazol-2-yl)(2-vinylphenyl)methanone

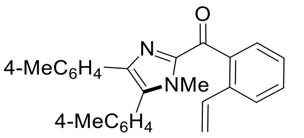

Prepared from **GPA**. Afforded the desired product as a yellow solid, Mp = 158–159 °C. ^{1}H NMR (400 MHz, $CDCl_3$) δ

= 7.93–7.91 (m, 1H), 7.64–7.62 (m, 1H), 7.46–7.41 (td, J = 1.6 Hz, J = 7.6 Hz, 1H), 7.35–7.31 (m, 3H), 7.26–7.20 (m, 4H), 7.13–7.06 (dd, J = 10.8 Hz, J = 17.6 Hz, 1H), 6.97–6.95 (m, 2H), 5.71–5.67 (m, 1H), 5.29–5.26 (dd, J = 1.2 Hz, J = 10.8 Hz, 1H), 3.85 (s, 3H), 2.39 (s, 3H), 2.22 (s, 3H). ^{13}C NMR (100 MHz, $CDCl_3$) δ = 186.74, 142.73, 139.50, 139.11, 138.07, 137.15, 136.43, 135.38, 134.83, 131.16, 130.79, 130.73, 130.30, 129.70, 128.64, 126.92, 126.47, 126.32, 126.23, 115.58, 34.01, 21.25, 20.98. HRMS (ESI) m/z calculated for $C_{27}H_{24}N_2ONa$ [M+Na]$^+$ 415.1781, found 415.1771.

(4,5-bis(4-isopropylphenyl)-1-methyl-1*H*-imidazol-2-yl)(2-vinylphenyl)methanone

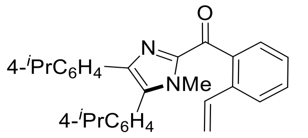

Prepared from **GPA**. Afforded the desired product as a white solid, Mp = 101–102 °C. ^{1}H NMR (400 MHz, $CDCl_3$) δ = 7.91–7.89 (m, 1H), 7.61–7.58 (dd, J = 1.2 Hz, J = 8.0 Hz, 1H), 7.41–7.35 (m, 3H), 7.28–7.20 (m, 5H), 7.15–7.08 (m, 1H), 7.00–6.98 (dd, J = 1.6 Hz, J = 6.8 Hz, 2H), 5.68–5.64 (m, 1H), 5.25–5.22 (dd, J = 1.2 Hz, J = 10.8 Hz, 1H), 3.80 (s, 3H), 2.97–2.86 (m, 1H), 2.81–2.71 (m, 1H), 1.26 (d, J = 7.2 Hz, 6H), 1.26 (d, J = 6.8 Hz, 6H). ^{13}C NMR (100 MHz, $CDCl_3$) δ = 186.95, 150.10, 147.57, 142.80, 139.59, 138.25, 137.29, 135.58, 135.03, 131.35, 130.87, 130.53, 127.19, 126.99, 126.80, 126.60, 126.40, 126.15, 115.70, 34.23, 33.95, 33.71, 23.84. HRMS (ESI) m/z calculated for $C_{31}H_{33}N_2O$ [M+H]$^+$ 449.2587, found 449.2582.

(4,5-bis(4-methoxyphenyl)-1-methyl-1*H*-imidazol-2-yl)(2-vinylphenyl)methanone

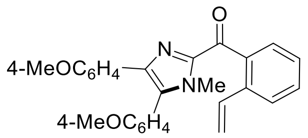

Prepared from **GPA**. Afforded the desired product as a yellow solid, Mp = 125–126 °C. ^{1}H NMR (400 MHz, $CDCl_3$) δ = 7.93–7.90 (m, 1H), 7.67–7.65 (m, 1H), 7.50–7.46 (td, J = 1.6 Hz, J = 7.6 Hz, 1H), 7.39–7.34 (m, 3H), 7.29–7.25 (m, 2H), 7.11–7.04 (dd, J = 10.8 Hz, J = 17.6 Hz, 1H), 7.03–6.99 (m, 2H), 6.74–6.71 (m, 2H), 5.73–5.69 (dd, J = 1.2 Hz, J = 17.2 Hz, 1H), 5.31–5.28 (dd, J = 1.2 Hz, J = 10.8 Hz, 1H), 3.87 (s, 3H), 3.86 (s, 3H), 3.73 (s, 3H). ^{13}C NMR (100 MHz, $CDCl_3$) δ = 186.83, 160.21, 158.64, 142.74, 139.50, 138.18, 137.28, 135.50, 134.34, 131.91, 131.22, 130.85, 128.35, 126.59, 126.46, 126.37, 121.43, 115.69, 114.59, 113.50, 55.27, 55.08, 34.14. HRMS (ESI) m/z calculated for $C_{27}H_{25}N_2O_3$ [M+H]$^+$ 425.1860, found 425.1851.

(1-methyl-4,5-bis(4-(trifluoromethyl)phenyl)-1*H*-imidazol-2-yl)(2-vinylphenyl)methanone

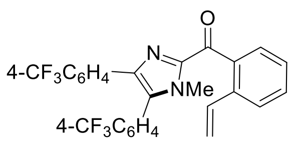

Prepared from **GPA**. Afforded the desired product as a yellow solid, Mp = 169–170 °C. ^{1}H NMR (400 MHz, $CDCl_3$) δ = 7.92–7.89 (dd, J = 1.2 Hz, J = 7.6 Hz, 1H), 7.79 (d, J = 8.4 Hz, 2H), 7.67–7.65 (dd, J = 1.2 Hz, J = 8.0 Hz, 1H), 7.51–7.46 (m, 5H), 7.44–7.42 (m, 2H), 7.38–7.34 (m, 1H), 7.12–7.05 (m, 1H), 5.73–5.68 (dd, J = 1.2 Hz, J = 17.6 Hz, 1H), 5.31–5.28 (dd, J = 1.2 Hz, J = 10.8 Hz, 1H), 3.87 (s, 3H). ^{13}C NMR (100 MHz, $CDCl_3$) δ = 187.08, 143.72, 138.42, 136.69, 136.61, 135.27, 134.00, 132.82, 131.92 (q, J = 33.0 Hz), 131.37, 131.17, 131.07, 129.26 (q, J = 32.0 Hz), 127.29, 126.73, 126.63, 126.42, 126.39, 126.35, 125.43, 125.25, 125.21, 125.17, 125.03, 122.73, 122.32, 116.19, 34.33. ^{19}F NMR (376 MHz, $CDCl_3$) δ = -62.49, -62.71. HRMS (ESI) m/z calculated for $C_{27}H_{18}F_6N_2ONa$ [M+Na]$^+$ 523.1216, found 523.1216.

(4,5-bis(4-chlorophenyl)-1-methyl-1*H*-imidazol-2-yl)(2-vinylphenyl)methanone

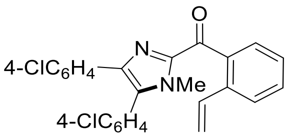

Prepared from **GPA**. Afforded the desired product as a pale yellow solid, Mp = 127–128 °C. ^{1}H NMR (400 MHz, $CDCl_3$) δ = 7.89–7.87 (m, 1H), 7.67–7.64 (m, 1H), 7.51–7.46 (m, 3H), 7.38–7.35 (td, *J* = 1.2 Hz, *J* = 7.6 Hz, 1H), 7.33–7.26 (m, 4H), 7.16–7.13 (m, 2H), 7.09–7.02 (m, 1H), 5.73–5.68 (dd, *J* = 1.2 Hz, *J* = 17.6 Hz, 1H), 5.31–5.28 (dd, *J* = 1.2 Hz, *J* = 10.8 Hz, 1H), 3.86 (s, 3H). ^{13}C NMR (100 MHz, $CDCl_3$) δ = 186.91, 143.27, 138.57, 138.25, 136.78, 135.76, 135.26, 133.71, 132.95, 131.84, 131.81, 131.13, 131.09, 129.63, 128.38, 128.34, 127.49, 126.64, 126.48, 116.00, 34.20. HRMS (ESI) m/z calculated for $C_{25}H_{19}Cl_2N_2O$ $[M+H]^+$ 433.0869, found 433.0866.

(4,5-bis(4-fluorophenyl)-1-methyl-1*H*-imidazol-2-yl)(2-vinylphenyl)methanone

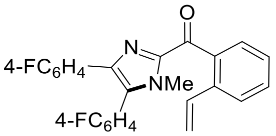

Prepared from **GPA**. Afforded the desired product as a pale yellow solid, Mp = 215–216 °C ^{1}H NMR (400 MHz, $CDCl_3$) δ = 7.91–7.88 (m, 1H), 7.67–7.64 (m, 1H), 7.50–7.46 (m, 1H), 7.71–7.31 (m, 5H), 7.24–7.17 (m, 2H), 7.10–7.03 (m, 1H), 6.89–6.84 (m, 2H), 5.73–5.68 (dd, *J* = 1.2 Hz, *J* = 17.6 Hz, 1H), 5.31–5.28 (dd, *J* = 1.2 Hz, *J* = 11.2 Hz, 1H), 3.86 (s, 3H). ^{13}C NMR (100 MHz, $CDCl_3$) δ = 186.93, 164.45 (d, *J* = 249.0 Hz), 163.20 (d, *J* = 246.0 Hz), 143.05, 138.82, 138.21, 136.92, 135.31, 133.61, 132.56 (d, *J* = 8.0 Hz), 131.07, 131.05, 129.58 (d, *J* = 3.0 Hz), 128.84 (d, *J* = 8.0 Hz), 126.62, 126.44, 125.16 (d, *J* = 3.0 Hz), 116.61, 116.40, 115.90, 115.15, 114.94, 34.14. ^{19}F NMR (376 MHz, $CDCl_3$) δ = -110.42, -114.65. HRMS (ESI) m/z calculated for $C_{25}H_{19}F_2N_2O$ $[M+H]^+$ 401.1460, found 401.1460.

Ketone derived from Ketoconazole

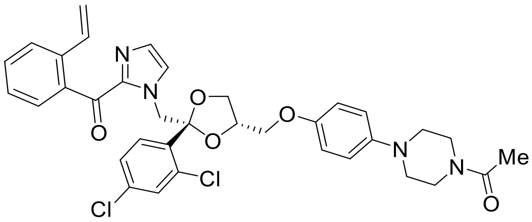

Prepared from **GPA**. Afforded the desired product as a pale red solid, Mp = 215–216 °C. ^{1}H NMR (400 MHz, $CDCl_3$) δ = 7.64–7.58 (m, 2H), 7.49–7.40 (m, 3H), 7.26–7.24 (m, 2H), 7.22–7.18 (td, *J* = 1.2 Hz, *J* = 7.6 Hz, 1H), 7.14 (d, *J* = 0.8 Hz, 1H), 6.96–6.85 (m, 3H), 6.75–6.73 (m, 2H), 5.71–5.57 (dd, *J* = 1.2 Hz, *J* = 17.2 Hz, 1H), 5.30–5.20 (m, 3H), 4.35–4.29 (m, 1H), 3.88–3.70 (m, 5H), 3.62–3.59 (m, 2H), 3.42–3.38 (dd, *J* = 6.4 Hz, *J* = 9.6 Hz, 1H), 3.06–3.01 (m, 4H), 2.14 (s, 3H). ^{13}C NMR (100 MHz, $CDCl_3$) δ = 187.49, 168.87, 152.70, 145.62, 144.15, 137.19, 135.71, 134.58, 134.36, 133.17, 131.24, 130.74, 129.77, 129.69, 128.93, 127.71, 127.08, 126.72, 126.00, 118.67, 115.89, 115.05, 108.09, 74.20, 67.53, 66.93, 50.90, 50.72, 50.58, 46.24, 41.34, 21.27. HRMS (ESI) m/z calculated for $C_{35}H_{34}Cl_2N_4O_5Na$ $[M+Na]^+$ 683.1798, found 683.1775.

thiazol-2-yl(2-vinylphenyl)methanone

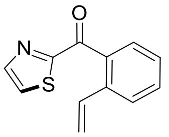

Prepared from **GPB**. Afforded the desired product as a White solid, Mp = 63–64 °C. ^{1}H NMR (400 MHz, $CDCl_3$) δ = 8.04 (d, J = 3.2 Hz, 1H), 7.84–7.82 (m, 1H), 7.73 (d, J = 3.2 Hz, 1H), 7.69–7.67 (m, 1H), 7.55–7.50 (m, 1H), 7.41–7.37 (td, J = 1.2 Hz, J = 8.0 Hz, 1H), 7.03–7.96 (m, 1H), 5.74–5.69 (dd, J = 1.2 Hz, J = 17.6 Hz, 1H), 5.32–5.29 (m, 1H). ^{13}C NMR (100 MHz, $CDCl_3$) δ = 187.81, 167.79, 145.11, 137.98, 134.73, 134.42, 131.65, 130.28, 126.94, 126.68, 126.52, 116.89. HRMS (ESI) m/z calculated for $C_{12}H_9NOSNa$ $[M+Na]^+$ 238.0297, found 238.0296.

benzo[*d*]thiazol-2-yl(2-vinylphenyl)methanone

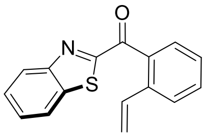

Prepared from **GPB**. Afforded the desired product as a yellow solid, Mp = 134–135 °C. ^{1}H NMR (400 MHz, $CDCl_3$) δ = 8.18–8.15 (m, 1H), 7.99–7.95 (m, 2H), 7.69–7.67 (m, 1H), 7.57–7.49 (m, 3H), 7.43–7.39 (m, 1H), 7.10–7.03 (dd, J = 10.8 Hz, J = 17.2 Hz, 1H), 5.73–5.69 (dd, J = 1.2 Hz, J = 17.2 Hz, 1H), 5.32–5.29 (dd, J = 1.2 Hz, J = 11.2 Hz, 1H). ^{13}C NMR (100 MHz, $CDCl_3$) δ = 188.90, 167.15, 153.66, 138.59, 137.21, 134.61, 134.27, 132.01, 130.96, 127.69, 126.98, 126.89, 126.76, 125.74, 122.15, 117.09. HRMS (ESI) m/z calculated for $C_{16}H_{11}NOSNa$ $[M+Na]^+$ 288.0454, found 288.0446.

(6-methylbenzo[*d*]thiazol-2-yl)(2-vinylphenyl)methanone

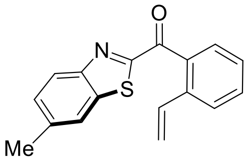

Prepared from **GPB**. Afforded the desired product as a yellow solid, Mp = 123–124 °C. ^{1}H NMR (400 MHz, $CDCl_3$) δ = 8.06 (d, J = 8.4 Hz, 1H), 7.96–7.94 (m, 1H), 7.79–7.78 (m, 1H), 7.70–7.68 (dd, J = 1.2 Hz, J = 9.2 Hz, 1H), 7.58–7.53 (m, 1H), 7.44–7.39 (td, J = 1.2 Hz, J = 7.6 Hz, 1H), 7.38–7.35 (dd, J = 2.0 Hz, J = 8.4 Hz, 1H), 7.09–7.02 (m, 1H), 5.74–5.69 (dd, J = 1.2 Hz, J = 17.2 Hz, 1H), 5.32–5.29 (m, 1H), 2.52 (s, 3H). ^{13}C NMR (100 MHz, $CDCl_3$) δ = 188.99, 166.10, 151.88, 138.46, 138.42, 137.53, 134.61, 134.43, 131.88, 130.88, 128.80, 126.95, 126.67, 125.23, 121.67, 116.95, 21.72. HRMS (ESI) m/z calculated for $C_{17}H_{13}NOSNa$ $[M+Na]^+$ 302.0610, found 302.0604.

(6-methoxybenzo[*d*]thiazol-2-yl)(2-vinylphenyl)methanone

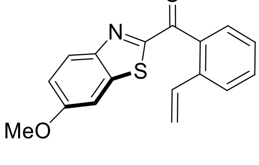

Prepared from **GPB**. Afforded the desired product as a yellow solid, Mp = 183–184 °C. ^{1}H NMR (400 MHz, $CDCl_3$) δ = 7.78 (t, J = 7.1 Hz, 2H), 7.48–7.16 (m, 4H), 6.94 (q, J = 9.8, 8.5 Hz, 2H), 5.60 (d, J = 17.3 Hz, 1H), 5.25 (d, J = 10.9 Hz, 1H), 4.06 (s, 3H). ^{13}C NMR (100 MHz, $CDCl_3$) δ = 186.50, 164.34, 161.53, 150.38, 142.40, 137.97, 134.71, 134.26, 131.88, 130.86, 127.04, 126.74, 126.63, 117.73, 116.98, 103.38, 55.85. HRMS (ESI) m/z calculated for $C_{17}H_{14}NO_2S$

[M+H]$^+$ 296.0740, found 296.0741.

(5,6-dimethoxybenzo[*d*]thiazol-2-yl)(2-vinylphenyl)methanone

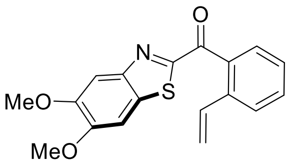

Prepared from **GPB**. Afforded the desired product as a yellow solid, Mp = 174–175 °C. ^{1}H NMR (400 MHz, $CDCl_3$) δ = 7.90 (dd, *J* = 7.8 Hz, *J* = 1.4 Hz, 1H), 7.68 (dd, *J* = 7.9 Hz, *J* = 1.2 Hz, 1H), 7.60–7.49 (m, 2H), 7.40 (td, *J* = 7.6 Hz, *J* = 1.3 Hz, 1H), 7.35 (s, 1H), 7.02 (dd, *J* = 17.4 Hz, *J* = 11.0 Hz, 1H), 5.71 (dd, *J* = 17.4 Hz, *J* = 1.1 Hz, 1H), 5.29 (dd, *J* = 11.0 Hz, *J* = 1.1 Hz, 1H), 3.99 (s, 3H), 3.93 (s, 3H). ^{13}C NMR (100 MHz, $CDCl_3$) δ = 188.78, 165.07, 151.08, 150.27, 148.48, 138.23, 134.89, 134.62, 131.69, 131.09, 130.61, 126.99, 126.61, 116.88, 105.96, 101.87, 56.35, 56.11. HRMS (ESI) m/z calculated for $C_{18}H_{15}NO_3SNa$ [M+Na]$^+$ 348.0665, found 348.0656.

(6-fluorobenzo[*d*]thiazol-2-yl)(2-vinylphenyl)methanone

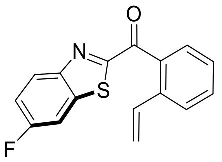

Prepared from **GPB**. Afforded the desired product as a white solid, Mp = 154–155 °C. ^{1}H NMR (400 MHz, $CDCl_3$) δ = 8.14–8.11 (dd, *J* = 4.8 Hz, *J* = 9.2 Hz, 1H), 7.98–7.96 (m, 1H), 7.71–7.65 (m, 2H), 7.59–7.55 (m, 1H), 7.44–7.40 (m, 1H), 7.32–7.27 (m, 1H), 7.09–7.02 (m, 1H), 5.74–5.69 (m, 1H), 5.34–5.31 (m, 1H). ^{13}C NMR (100 MHz, $CDCl_3$) δ = 188.53, 167.14, 163.24 (d, *J* = 250.0 Hz), 150.40, 138.69 (d, *J* = 3.0 Hz), 138.54, 134.59, 134.08, 132.16, 130.95, 127.22 (d, *J* = 10.0 Hz), 127.03, 126.86, 117.25, 116.43 (d, *J* = 25.0 Hz), 108.30 (d, *J* = 27.0 Hz). ^{19}F NMR (376 MHz, $CDCl_3$) δ = -110.84. HRMS (ESI) m/z calculated for $C_{16}H_{11}FNOS$ [M+H]$^+$ 284.0540, found 284.0546.

methyl 2-(2-vinylbenzoyl)benzo[*d*]thiazole-6-carboxylate

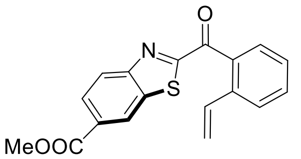

Prepared from **GPB**. Afforded the desired product as a pale yellow solid, Mp = 205–206 °C. ^{1}H NMR (400 MHz, $CDCl_3$) δ = 8.74 (t, *J* = 1.2 Hz, 1H), 8.21 (d, *J* = 1.2 Hz, 2H), 8.01–7.99 (m, 1H), 7.72–7.69 (dd, *J* = 1.2 Hz, *J* = 8.0 Hz, 1H), 7.61–7.57 (td, *J* = 1.6 Hz, *J* = 7.6 Hz, 1H), 7.46–7.42 (dd, *J* = 1.2 Hz, *J* = 7.6 Hz, 1H), 7.10–7.03 (dd, *J* = 10.8 Hz, *J* = 17.2 Hz, 1H), 5.75–5.69 (dd, *J* = 1.2 Hz, *J* = 17.2 Hz, 1H), 5.35–5.32 (dd, *J* = 1.2 Hz, *J* = 11.2 Hz, 1H), 3.99 (s, 3H). ^{13}C NMR (100 MHz, $CDCl_3$) δ = 188.54, 170.31, 166.19, 156.33, 138.93, 137.02, 134.64, 133.86, 132.39, 131.14, 129.19, 127.79, 127.09, 127.01, 125.57, 124.50, 117.45, 52.58. HRMS (ESI) m/z calculated for $C_{18}H_{13}NO_3SNa$ [M+Na]$^+$ 346.0510, found 346.0506.

naphtho[1,2-*d*]thiazol-2-yl(2-vinylphenyl)methanone

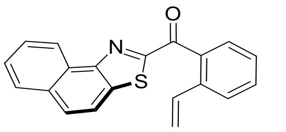

Prepared from **GPB**. Afforded the desired product as a pale yellow solid, Mp = 179–180 °C. ^{1}H NMR (400 MHz, $CDCl_3$)

δ = 8.20–8.14 (m, 1H), 8.10 (d, J = 8.9 Hz, 1H), 8.02 (dd, J = 7.7 Hz, J =1.4 Hz, 1H), 8.00–7.94 (m, 1H), 7.90 (d, J = 8.9 Hz, 1H), 7.72 (dd, J = 7.9 Hz, J =1.2 Hz, 1H), 7.69–7.61 (m, 2H), 7.58 (td, J = 7.6 Hz, J = 1.4 Hz, 1H), 7.45 (td, J = 7.6 Hz, J =1.3 Hz, 1H), 7.09 (dd, J = 17.3 Hz, J = 11.0 Hz, 1H), 5.74 (dd, J = 17.4 Hz, J = 1.2 Hz, 1H), 5.33 (dd, J = 11.0 Hz, J = 1.2 Hz, 1H). ^{13}C NMR (100 MHz, $CDCl_3$) δ = 188.63, 166.00, 152.17, 138.64, 136.47, 134.72, 134.50, 132.02, 131.94, 131.01, 129.06, 128.45, 127.85, 127.66, 127.50, 127.07, 126.83, 125.87, 122.89, 117.11. HRMS (ESI) m/z calculated for $C_{20}H_{13}NOSNa$ [M+Na]$^+$ 338.0610, found 338.0597.

benzo[*d*]thiazol-2-yl(5-methyl-2-vinylphenyl)methanone

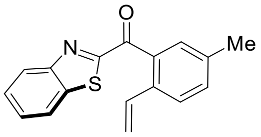

Prepared from **GPB**. Afforded the desired product as a pale yellow solid, Mp = 117–118 °C. ^{1}H NMR (400 MHz, $CDCl_3$) δ = 8.18–8.16 (m, 1H), 7.98–7.96 (m, 1H), 7.70 (s, 1H), 7.59 (d, J = 8.0 Hz, 1H), 7.55–7.48 (m, 2H), 7.35–7.33 (m, 1H), 7.03–6.96 (m, 1H), 5.68–5.64 (m, 1H), 5.26–5.23 (m, 1H), 2.39 (s, 3H). ^{13}C NMR (100 MHz, $CDCl_3$) δ = 188.53, 167.14, 163.24, 161.24, 160.74, 160.28, 150.40, 138.69, 138.66, 138.54, 134.59, 134.08, 132.16, 130.95, 127.22, 127.12, 127.03, 126.86, 117.25, 116.43, 116.18, 108.30, 108.03. HRMS (ESI) m/z calculated for $C_{17}H_{14}NOS$ [M+H]$^+$ 280.0791, found 280.0794.

benzo[*d*]thiazol-2-yl(5-fluoro-2-vinylphenyl)methanone

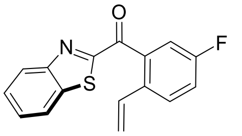

Prepared from **GPB**. Afforded the desired product as a pale yellow solid, Mp = 191–192 °C. ^{1}H NMR (400 MHz, $CDCl_3$) δ = 8.19–8.17 (m, 1H), 8.13–8.09 (m, 1H), 8.03–8.00 (m, 1H), 7.59–7.53 (m, 2H), 7.39–7.36 (dd, J = 2.4 Hz, J = 10.0 Hz, 1H), 7.14–7.06 (m, 2H), 5.76–5.72 (dd, J = 0.8 Hz, J = 17.6 Hz, 1H), 5.41–5.38 (dd, J = 0.8 Hz, J = 10.8 Hz, 1H). ^{13}C NMR (100 MHz, $CDCl_3$) δ = 187.42, 167.25, 166.19 (d, J = 251.0 Hz), 153.70, 142.39 (d, J = 9.0 Hz), 137.31, 134.24, 134.15, 133.99, 130.34 (d, J = 3.0 Hz), 127.86, 127.07, 125.82, 122.26, 118.22, 114.33(d, J = 22.0 Hz), 113.92 (d, J = 22.0 Hz). ^{19}F NMR (376 MHz, $CDCl_3$) δ = -108.38. HRMS (ESI) m/z calculated for $C_{16}H_{10}FNOSNa$ [M+Na]$^+$306.0359, found 306.0346.

benzo[*d*]thiazol-2-yl(2-vinylphenyl)methanone

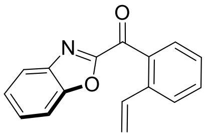

Prepared from **GPC**. Afforded the desired product as a brown solid, Mp = 77–78 °C. ^{1}H NMR (400 MHz, $CDCl_3$) δ = 7.99–7.97 (m, 1H), 7.91–7.88 (m, 1H), 7.69–7.67 (m, 2H), 7.60–7.51 (m, 2H), 7.46–7.42 (m, 2H), 7.11–7.04 (m, 1H), 5.72–5.68 (m, 1H), 5.35–5.32 (dd, J = 1.2 Hz, J = 10.8 Hz, 1H). ^{13}C NMR (100 MHz, $CDCl_3$) δ = 183.62, 157.71, 150.55, 140.65, 138.88, 134.39, 134.15, 132.47, 130.81, 128.47, 127.12, 127.01, 125.67, 122.39, 117.76, 111.77. HRMS (ESI) m/z calculated for $C_{16}H_{12}NO_2$ [M+H]$^+$ 250.0863, found 250.0856.

(4,5-diphenyloxazol-2-yl)(2-vinylphenyl)methanone

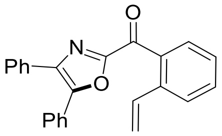

Prepared from **GPC.** Afforded the desired product as a White solid, Mp = 155–156 °C. ^{1}H NMR (400 MHz, $CDCl_3$) δ = 8.03 (dd, *J* = 7.8 Hz, *J* = 1.4 Hz, 1H), 7.73 (dd, *J* = 6.7 Hz, *J* = 3.0 Hz, 2H), 7.68 (dd, *J* = 7.2 Hz, *J* = 2.4 Hz, 3H), 7.55 (td, *J* = 7.6 Hz, *J* = 1.4 Hz, 1H), 7.44–7.33 (m, 7H), 7.14 (dd, *J* = 17.4 Hz, *J* = 10.9 Hz, 1H), 5.73 (d, *J* = 17.3 Hz, 1H), 5.36 (d, *J* = 11.1 Hz, 1H). ^{13}C NMR (100 MHz, $CDCl_3$) δ = 181.85, 156.46, 148.80, 138.78, 137.72, 134.71, 134.57, 132.08, 131.34, 130.89, 129.84, 128.78, 128.75, 128.62, 128.20, 127.55, 127.24, 127.04, 126.82, 117.16. HRMS (ESI) m/z calculated for $C_{24}H_{17}NO_2Na$ $[M+Na]^+$ 374.1152, found 374.1149.

Alkyl bromide derived from Stanolone[5]

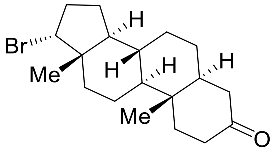

White solid, Mp = 256–257 °C. ^{1}H NMR (400 MHz, $CDCl_3$) δ = 4.25 (d, *J* = 6.4 Hz, 1H), 2.66–2.57 (m, 1H), 2.44–2.18 (m, 4H), 2.13–2.07 (m, 1H), 2.06–2.01 (m, 1H), 1.92–1.84 (m, 1H), 1.78–1.71 (m, 2H), 1.68–1.51 (m, 4H), 1.43–1.24 (m, 6H), 1.08–0.98 (m, 4H), 0.88–0.79 (m, 4H). ^{13}C NMR (100 MHz, $CDCl_3$) δ = 211.90, 65.98, 53.11, 48.34, 46.46, 45.95, 44.59, 38.45, 38.09, 36.01, 35.87, 35.63, 34.90, 31.83, 28.79, 24.68, 21.47, 17.48, 11.46. HRMS (ESI) m/z calculated for $C_{19}H_{29}BrONa$ $[M+Na]^+$ 375.1294, found 375.1294.

Difluoroalkyl bromide derived from Ketoprofen

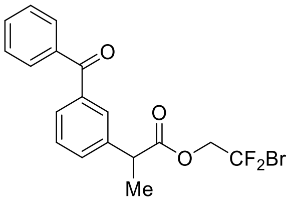

Prepared from **GPD**. Afforded the desired product as a White liquid. ^{1}H NMR (400 MHz, $CDCl_3$) δ = 7.83–7.75 (m, 3H), 7.70 (d, *J* = 7.7 Hz, 1H), 7.63–7.53 (m, 2H), 7.47 (dt, *J* = 10.5 Hz, *J* = 7.7 Hz, 3H), 4.60 (td, *J* = 11.5 Hz, *J* = 2.3 Hz, 2H), 3.92 (q, *J* = 7.2 Hz, 1H), 1.60 (d, *J* = 7.2 Hz, 3H).^{13}C NMR (100 MHz, $CDCl_3$) δ = 196.33, 172.28, 139.56, 137.99, 137.33, 132.54, 131.47, 130.01, 129.28, 129.19, 128.64, 128.29, 117.88 (t, *J* = 315.0 Hz), 67.05 (t, *J* = 27.8 Hz), 44.94, 18.11. ^{19}F NMR (376 MHz, $CDCl_3$) δ = -56.17. HRMS (ESI) m/z calculated for $C_{18}H_{15}BrF_2O_3Na$ $[M+Na]^+$ 419.0065, found 419.0057.

Difluoroalkyl bromide derived from Fernoxone

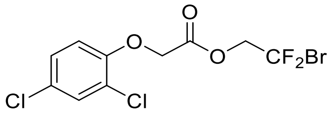

Prepared from **GPD**. Afforded the desired product as a White liquid. ^{1}H NMR (400 MHz, $CDCl_3$) δ = 7.37 (d, *J* = 2.4 Hz, 1H), 7.16–7.13 (dd, *J* = 2.8 Hz, *J* = 8.8 Hz, 1H), 6.78 (d, *J* = 8.8 Hz, 1H), 4.79 (s, 2H), 4.71 (t, *J* = 11.6 Hz, 2H). ^{13}C NMR (100 MHz, $CDCl_3$) δ = 166.47, 151.89, 130.25, 127.49, 127.30, 124.16, 117.32 (t, *J* = 304.0 Hz), 114.73, 67.02 (t, *J* = 28.0 Hz), 65.66. ^{19}F NMR (376 MHz, $CDCl_3$) δ = -56.58. HRMS (ESI) m/z calculated for $C_{10}H_7BrCl_2F_2O_3Na$

[M+Na]+ 384.8816, found 384.8814.

Alkyl bromide derived from Estrone[6]

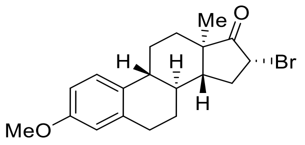

White solid, Mp = 233–234 °C. ^{1}H NMR (400 MHz, $CDCl_3$) δ = 7.22 (d, *J* = 8.8 Hz, 1H), 6.75–6.72 (m, 1H), 6.66–6.65 (m, 1H), 4.61 (d, *J* = 7.2 Hz, 1H), 3.79 (s, 3H), 2.93–2.89 (m, 2H), 2.45–2.24 (m, 4H), 2.08–1.91 (m, 3H), 1.68–1.46 (m, 4H), 0.95 (s, 3H). ^{13}C NMR (100 MHz, $CDCl_3$) δ = 213.23, 157.65, 137.56, 131.61, 126.26, 113.86, 111.62, 55.20, 48.02, 46.97, 46.32, 43.76, 37.58, 33.91, 32.28, 29.48, 26.46, 25.73, 14.21. HRMS (ESI) m/z calculated for $C_{19}H_{23}BrO_3Na$ $[M+Na]^+$ 385.0774, found 385.0774.

Alkyl bromide derived from *L*-menthol[7]

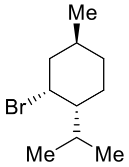

White liquid. ^{1}H NMR (400 MHz, $CDCl_3$) ^{1}H NMR (400 MHz, $CDCl_3$) δ = 4.67 (s, 1H), 2.16 (dq, *J* = 14.3 Hz, *J* = 3.1 Hz, 1H), 2.03–1.88 (m, 1H), 1.81–1.69 (m, 2H), 1.55–1.30 (m, 3H), 0.98–0.84 (m, 10H), 0.78 (ddt, *J* = 12.1 Hz, *J* = 9.3 Hz, *J* = 3.0 Hz, 1H). ^{13}C NMR (100 MHz, $CDCl_3$) δ 60.54, 49.18, 43.86, 34.78, 31.33, 26.73, 25.02, 21.75, 20.63, 20.04. HRMS (ESI) m/z calculated for $C_{10}H_{19}BrNa$ $[M+Na]^+$ 241.0562, found 241.0557.

Difluoroalkyl bromide derived from *D*-Mannofuranose

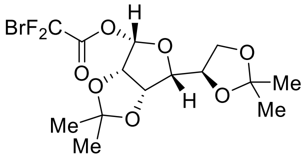

Prepared from **GPE**. Afforded the desired product as a colorless solid, Mp = 74–75 °C. ^{1}H NMR (400 MHz, $CDCl_3$) δ = 6.08 (s, 1H), 4.96 (d, *J* = 5.9 Hz, 1H), 4.89 (dd, *J* = 5.8 Hz, *J* = 3.6 Hz, 1H), 4.44 (ddd, *J* = 8.0 Hz, *J* = 6.2 Hz, *J* = 4.3 Hz, 1H), 4.21 (dd, *J* = 7.8 Hz, *J* = 3.6 Hz, 1H), 4.10 (dd, *J* = 8.9, *J* = 6.2 Hz, 1H), 4.02 (dd, *J* = 8.9, *J* = 4.3 Hz, 1H), 1.47 (s, 6H), 1.39 (s, 3H), 1.34 (s, 3H). ^{13}C NMR (100 MHz, $CDCl_3$) δ = 132.52, 128.60, 113.19, 109.42, 97.54, 89.07, 82.27, 78.43, 72.21, 66.62, 26.82, 25.71, 25.04, 24.52. ^{19}F NMR (376 MHz, $CDCl_3$) δ = -60.69. HRMS (ESI) m/z calculated for $C_{14}H_{19}BrF_2O_7Na$ $[M+Na]^+$ 439.0174, found 439.0171.

Difluoroalkyl bromide derived from Naproxen

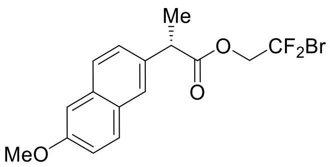

Prepared from **GPD**. Afforded the desired product as a White solid, Mp = 57–58 °C. ^{1}H NMR (400 MHz, $CDCl_3$) δ = 7.69–7.66 (m, 3H), 7.39 (d, *J* = 8.4 Hz, 1H), 7.14–7.08 (m, 2H), 4.66–4.48 (m, 2H), 3.95–3.91 (q, *J* = 7.2 Hz, 1H), 3.86 (s, 3H), 1.62 (d, *J* = 7.2 Hz, 3H). ^{13}C NMR (100 MHz, $CDCl_3$) δ = 172.84, 157.71, 134.34, 133.79, 129.23, 128.81, 127.22, 126.10, 126.02, 119.07, 118.05 (t, *J* = 304.0 Hz), 105.49, 66.94 (t, *J* = 27.0 Hz), 55.16, 44.98, 18.21. ^{19}F NMR (376 MHz, $CDCl_3$) δ = -56.09. HRMS (ESI) m/z calculated for $C_{16}H_{15}BrF_2O_3Na$ $[M+Na]^+$ 395.0065, found 395.0065.

Difluoroalkyl bromide derived from Nortropine

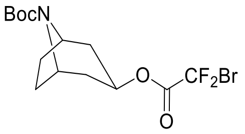

Prepared from **GPE**. Afforded the desired product as a White solid, Mp = 89–90 °C. ^{1}H NMR (400 MHz, $CDCl_3$) δ = 5.30–5.28 (m, 1H), 4.31–4.21 (m, 2H), 2.32–2.16 (br, 2H), 2.03–2.02 (m, 4H), 1.87–1.82 (m, 2H), 1.47 (s, 9H). ^{13}C NMR (100 MHz, $CDCl_3$) δ = 158.48 (t, J = 31.3 Hz), 153.05, 108.82 (t, J = 314.5 Hz), 79.44, 73.28, 52.13, 51.35, 35.08, 34.39, 28.27, 27.97, 27.67, 27.27. ^{19}F NMR (376 MHz, $CDCl_3$) δ = -61.07. HRMS (ESI) m/z calculated for $C_{14}H_{20}BrF_2NO_4Na$ $[M+Na]^+$ 406.0436, found 406.0436.

Difluoroalkyl bromide derived from Oxaprozin

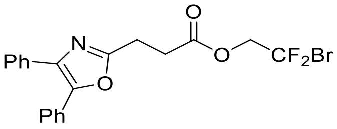

Prepared from **GPD**. Afforded the desired product as a yellow solid, Mp = 79–80 °C. ^{1}H NMR (400 MHz, $CDCl_3$) δ = 7.64–7.61 (m, 2H), 7.58–7.55 (m, 2H), 7.38–7.29 (m, 6H), 4.65 (t, J = 11.6 Hz, 2H). 3.23–3.19 (m, 2H), 3.06–3.02 (m, 2H). ^{13}C NMR (100 MHz, $CDCl_3$) δ = 170.29, 161.04, 145.50, 135.08, 132.27, 128.81, 128.59, 128.50, 128.46, 128.03, 127.78, 126.42, 117.91 (t, J = 304.0 Hz), 77.00, 67.05 (t, J = 27.0 Hz), 30.52, 23.15. ^{19}F NMR (376 MHz, $CDCl_3$) δ = -56.19 (t, J = 11.8 Hz, 2F). HRMS (ESI) m/z calculated for $C_{20}H_{17}BrF_2NO_3$ $[M+H]^+$ 436.0354, found 436.0325.

Difluoroalkyl bromide derived from Indometacin

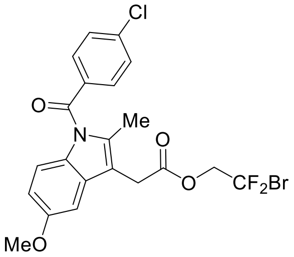

Prepared from **GPD**. Afforded the desired product as a white solid, Mp = 97–98 °C. ^{1}H NMR (400 MHz, $CDCl_3$) δ = 7.65 (d, J = 8.0 Hz, 2H), 7.46–7.44 (m, 2H), 6.95 (s, 1H), 6.89 (d, J = 8.8 Hz, 1H), 6.69–6.66 (m, 1H), 4.62 (t, J = 11.6 Hz, 2H), 3.82 (s, 3H), 3.77 (s, 2H), 2.38 (s, 3H). ^{13}C NMR (100 MHz, $CDCl_3$) δ = 168.95, 168.15, 156.01, 139.25, 136.14, 133.66, 131.11, 130.66, 130.17, 129.04, 114.91 (t, J = 299.0 Hz), 111.86, 111.28, 100.86, 67.13 (t, J = 28.0 Hz), 55.53, 29.68, 13.22. ^{19}F NMR (376 MHz, $CDCl_3$) δ = -56.25. HRMS (ESI) m/z calculated for $C_{21}H_{17}BrF_2ClNO_4Na$ $[M+Na]^+$ 521.9890, found 521.9890.

Difluoroalkyl bromide derived from Cholesterol

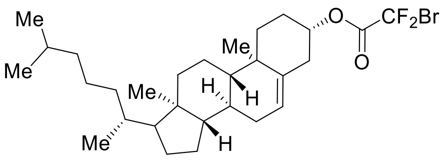

Prepared from **GPE**. Afforded the desired product as a white solid, Mp = 175–176 °C. ^{1}H NMR (400 MHz, $CDCl_3$) δ = 5.45–5.41 (m, 1H), 4.83–4.75 (m, 1H), 2.51–2.38 (m, 2H), 2.05–1.89 (m, 4H), 1.87–1.69 (m, 2H), 1.60–1.44 (m, 6H), 1.39–1.25 (m, 4H), 1.19–1.06 (m, 7H), 1.05 (s, 3H), 1.03–0.95 (m, 3H), 0.93 (d, J = 6.4 Hz, 3H), 0.88 (d, J = 2.0 Hz, 3H), 0.86 (d, J = 1.6 Hz, 3H), 0.68 (s, 3H). ^{13}C NMR (100 MHz, $CDCl_3$) δ = 158.98 (t, J = 30.0 Hz), 138.46, 123.74,

108.97 (t, J = 313.0 Hz), 78.77, 56.62, 56.12, 49.93, 42.28, 39.66, 39.50, 37.30, 36.70, 36.49, 36.17, 35.79, 31.88, 31.78, 28.21, 28.00, 27.12, 24.26, 23.84, 22.81, 22.56, 21.02, 19.23, 18.70, 11.83. ^{19}F NMR (376 MHz, $CDCl_3$) δ = -60.95. HRMS (ESI) m/z calculated for $C_{29}H_{45}BrF_2O_2Na$ $[M+Na]^+$ 565.2643, found 565.2630.

Difluoroalkyl bromide derived from Ibuprofen

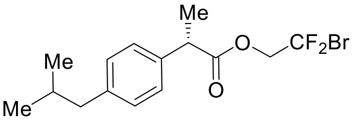

Prepared from **GPD**. Afforded the desired product as a white liquid. ^{1}H NMR (400 MHz, $CDCl_3$) δ = 7.23–7.19 (m, 2H), 7.12–7.08 (m, 2H), 4.66–4.45 (m, 2H), 3.79 (q, J = 7.1 Hz, 1H), 2.44 (d, J = 7.2 Hz, 2H), 1.84 (dt, J = 13.5 Hz, J = 6.8 Hz, 1H), 1.53 (d, J = 7.2 Hz, 3H), 0.88 (d, J = 6.7 Hz, 6H).^{13}C NMR (100 MHz, $CDCl_3$) δ = 172.90, 140.89, 136.48, 129.38, 127.20, 118.08 (t, J = 306.3 Hz), 66.90 (t, J = 27.5 Hz), 44.96, 44.70, 30.15, 22.29, 18.15. ^{19}F NMR (376 MHz, $CDCl_3$) δ = -55.73 – -56.23 (m, 2F). HRMS (ESI) m/z calculated for $C_{15}H_{19}BrF_2O_2Na$ $[M+Na]^+$ 371.0429, found 371.0418.

Difluoroalkyl bromide derived from Flurbiprofen

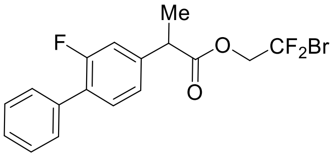

Prepared from **GPD**. Afforded the desired product as a white liquid. ^{1}H NMR (400 MHz, $CDCl_3$) δ = 7.63–7.51 (m, 2H), 7.46–7.34 (m, 4H), 7.18–7.09 (m, 2H), 4.61 (dt, J = 17.7, J = 11.7 Hz, 2H), 3.85 (q, J = 7.3 Hz, 1H), 1.58 (d, J = 7.3 Hz, 3H). ^{13}C NMR (100 MHz, $CDCl_3$) δ = 172.21, 160.89, 158.42, 140.46 (d, J = 7.7 Hz), 135.29, 130.89 (d, J = 4.0 Hz), 128.91 (d, J = 2.9 Hz), 128.44, 128.17 (d, J = 13.5 Hz), 127.72, 123.61, 123.58, 117.92 (t, J = 305.0 Hz), 115.31 (d, J = 23.8 Hz), 67.10 (t, J = 27.6 Hz), 44.57, 18.08. ^{19}F NMR (376 MHz, $CDCl_3$) δ = -56.09 (t, J = 12.0 Hz, 2F), -117.18 (t, J = 11.4 Hz, 1F). HRMS (ESI) m/z calculated for $C_{17}H_{14}BrF_3O_2Na$ $[M+Na]^+$ 409.0021, found 409.0014.

Difluoroalkyl bromide derived from Gemfibrozil

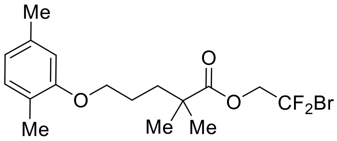

Prepared from **GPD**. Afforded the desired product as a White liquid. ^{1}H NMR (400 MHz, $CDCl_3$) δ = 7.01–6.99 (m, 1H), 6.67–6.64 (m, 1H), 6.60 (d, J = 1.6 Hz, 1H), 4.58 (t, J = 11.6 Hz, 2H), 3.93–3.91 (m, 2H), 2.30 (s, 3H), 2.17 (s, 3H), 1.77–1.75 (m, 4H), 1.27 (s, 6H). ^{13}C NMR (100 MHz, $CDCl_3$) δ = 175.94, 156.82, 136.43, 130.28, 123.54, 120.70, 118.27 (q, J = 304.0 Hz), 111.84, 67.63, 66.84 (q, J = 28.0 Hz), 42.28, 36.84, 25.03, 24.95, 21.36, 15.71. ^{19}F NMR (376 MHz, $CDCl_3$) δ = -55.94. HRMS (ESI) m/z calculated for $C_{17}H_{23}BrF_2O_3Na$ $[M+Na]^+$ 415.0691, found 415.0689.

Difluoroalkyl bromide derived from Adapalene

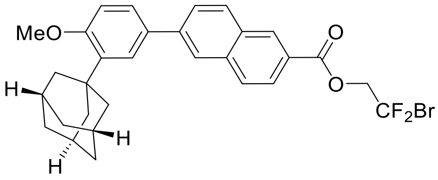

Prepared from **GPD**. Afforded the desired product as a white solid, Mp = 218–219 °C. ^{1}H NMR (400 MHz, $CDCl_3$) δ = 8.67 (dd, *J* = 10.8 Hz, *J* = 1.7 Hz, 1H), 8.08 (dd, *J* = 8.6 Hz, *J* = 1.7 Hz, 1H), 8.03–7.99 (m, 2H), 7.94 (d, *J* = 8.6 Hz, 1H), 7.82 (dd, *J* = 8.4 Hz, *J* = 1.8 Hz, 1H), 7.61 (d, *J* = 2.2 Hz, 1H), 7.55 (dd, *J* = 8.4 Hz, *J* = 2.2 Hz, 1H), 7.00 (d, *J* = 8.4 Hz, 1H), 4.90 (t, *J* = 11.5 Hz, 2H), 3.90 (s, 3H), 2.18 (d, *J* = 2.9 Hz, 6H), 2.16–1.96 (m, 3H), 1.80 (d, *J* = 3.1 Hz, 6H). ^{13}C NMR (100 MHz, $CDCl_3$) δ = 165.00, 158.97,139.06 (t, *J* = 287.0 Hz), 132.26, 132.01, 131.67, 131.07, 129.83, 128.49, 126.69, 125.94, 125.75, 125.45, 125.04, 124.68, 112.03, 67.42 (t, *J* = 9.0 Hz), 54.25, 40.53, 33.85, 29.77, 26.87. ^{19}F NMR (376 MHz, $CDCl_3$) δ = -55.85 (t, *J* = 11.0 Hz, 2F). HRMS (ESI) m/z calculated for $C_{30}H_{29}BrF_2O_3Na$ $[M+Na]^+$ 577.1160, found 577.1159.

3.3 Characterization of Rearrangement Products

ethyl 2,2-difluoro-3-(5-methyl-11-oxo-5,11-dihydrobenzo[4,5]imidazo[1,2-*b*]isoquinolin-6-yl)propanoate (2)

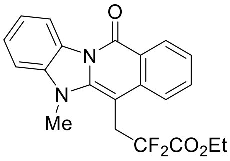

Prepared from **GPF**. Afforded the desired product **2** as a yellow solid, Mp = 253–254 °C. ^{1}H NMR (400 MHz, $CDCl_3$) δ = 8.79–8.77 (m, 1H), 8.50–8.48 (m, 1H), 7.59–7.53 (m, 2H), 7.34–7.26 (m, 2H), 7.19–7.14 (m, 1H), 7.05–7.02 (dd, *J* = 1.2 Hz, *J* = 8.0 Hz, 1H), 4.20–4.15 (m, 2H), 3.87–3.81 (m, 5H), 1.19–1.16 (m, 3H). ^{13}C NMR (100 MHz, $CDCl_3$) δ = 164.36 (t, *J* = 32.0 Hz), 159.59, 140.87, 138.07, 135.08, 132.04, 127.57, 127.50, 125.70, 122.43, 121.36, 121.14, 118.91, 116.84, 115.18, 106.73, 79.91, 63.19, 32.68, 29.85 (t, *J* = 24.0 Hz), 13.67. ^{19}F NMR (376 MHz, $CDCl_3$) δ = -102.73, -104.91. HRMS (ESI) m/z calculated for $C_{21}H_{19}F_2N_2O_3$ $[M+H]^+$ 385.1358, found 385.1358.

6-(cyclopentylmethyl)-5-methylbenzo[4,5]imidazo[1,2-*b*]isoquinolin-11(5*H*)-one (3)

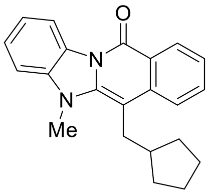

Prepared from **GPF**. Afforded the desired product **3** as a yellow solid, Mp = 157–158 °C. ^{1}H NMR (400 MHz, $CDCl_3$) δ = 8.88 (d, *J* = 8.0 Hz, 1H), 8.58 (d, *J* = 8.0 Hz, 1H), 7.77–7.74 (dd, *J* = 1.2 Hz, *J* = 8.0 Hz, 1H), 7.65–7.61 (m, 1H), 7.37–7.29 (m, 2H), 7.20–7.16 (m, 1H), 7.09–7.06 (m, 1H), 3.84 (s, 3H), 3.15–3.12 (m, 2H), 2.19–2.12 (m, 1H), 1.71–1.62 (m, 4H), 1.52–1.47 (m, 2H), 1.31–1.24 (m, 2H). ^{13}C NMR (100 MHz, $CDCl_3$) δ = 159.62, 139.15, 138.20, 136.00, 131.65, 128.14, 127.82, 125.54, 122.18, 121.94, 120.78, 119.53, 117.00, 106.54, 91.80, 41.91, 33.75, 32.24, 29.30, 24.86. HRMS (ESI) m/z calculated for $C_{22}H_{23}N_2O$ $[M+H]^+$ 331.1805, found 331.1805.

6-(cyclohexylmethyl)-5-methylbenzo[4,5]imidazo[1,2-*b*]isoquinolin-11(5*H*)-one (4)

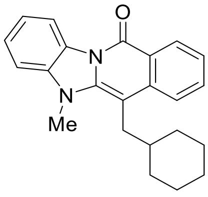

Prepared from **GPF**. Afforded the desired product **4** as a yellow solid, Mp = 258–259 °C. ^{1}H NMR (400 MHz, $CDCl_3$)

δ = 8.89–8.88 (dd, J = 1.2 Hz, J = 8.0 Hz, 1H), 8.59–8.57 (m, 1H), 7.73–7.70 (m, 1H), 7.67–7.62 (m, 1H), 7.39–7.31 (m, 2H), 7.22–7.18 (m, 1H), 7.11–7. 90 (m, 1H), 3.86 (s, 3H), 3.03 (d, J = 7.2 Hz, 2H), 1.72–1.55 (m, 6H), 1.16–1.05 (m, 5H). ^{13}C NMR (100 MHz, $CDCl_3$) δ = 159.67, 139.34, 138.48, 135.97, 131.71, 128.13, 127.82, 125.56, 122.18, 122.11, 120.82, 119.47, 117.06, 106.52, 90.58, 39.69, 33.65, 33.30, 31.48, 26.47, 26.41. HRMS (ESI) m/z calculated for $C_{23}H_{25}N_2O$ [M+H]$^+$ 345.1961, found 345.1961.

6-(cycloheptylmethyl)-5-methylbenzo[4,5]imidazo[1,2-*b*]isoquinolin-11(5*H*)-one (5)

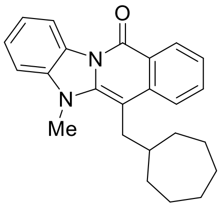

Prepared from **GPF**. Afforded the desired product **5** as a yellow solid, Mp = 118–119 °C. ^{1}H NMR (400 MHz, $CDCl_3$) δ = 8.89–8.86 (m, 1H), 8.59–8.56 (m, 1H), 7.69–7.62 (m, 2H), 7.38–7.30 (m, 2H), 7.21–7.17 (m, 1H), 7.09–7.07 (m, 1H), 3.84 (s, 3H), 3.02 (d, J = 7.2 Hz, 2H), 1.78–1.68 (m, 3H), 1.66–1.42 (m, 6H), 1.32–1.24 (m, 4H). ^{13}C NMR (100 MHz, $CDCl_3$) δ = 159.66, 139.42, 138.43, 135.94, 131.73, 128.09, 127.79, 125.54, 122.17, 122.02, 120.78, 119.50, 117.02, 106.52, 90.98, 41.16, 34.32, 33.65, 31.95, 28.21, 26.36. HRMS (ESI) m/z calculated for $C_{24}H_{26}N_2ONa$ [M+Na]$^+$ 381.1937, found 381.1940.

6-(cyclododecylmethyl)-5-methylbenzo[4,5]imidazo[1,2-*b*]isoquinolin-11(5*H*)-one (6)

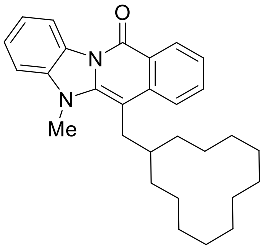

Prepared from **GPF**. Afforded the desired product **6** as a yellow solid, Mp = 189–190 °C. ^{1}H NMR (400 MHz,$CDCl_3$) δ = 8.88 (dd, J = 7.9 Hz, J = 1.2 Hz, 1H), 8.58 (dd, J = 8.1 Hz, J = 1.5 Hz, 1H), 7.78–7.59 (m, 2H), 7.43–7.31 (m, 2H), 7.20 (td, J = 7.7 Hz, J = 1.1 Hz, 1H), 7.14–7.08 (m, 1H), 3.85 (s, 3H), 3.07 (d, J = 7.3 Hz, 2H), 1.39–1.16 (m, 23H). ^{13}C NMR (100 MHz, $CDCl_3$) δ = 159.76, 139.59, 138.32, 136.04, 131.71, 128.18, 127.83, 125.56, 122.27, 121.97, 120.83, 119.63, 117.05, 106.58, 90.79, 35.09, 33.98, 29.64, 29.11, 24.57, 23.91, 23.29, 23.15, 21.81. HRMS (ESI) m/z calculated for $C_{29}H_{37}N_2O$ [M+H]$^+$ 429.2900, found 429.2899.

5-methyl-6-((tetrahydro-2*H*-pyran-4-yl)methyl)benzo[4,5]imidazo[1,2-*b*]isoquinolin-11(5*H*)-one (7)

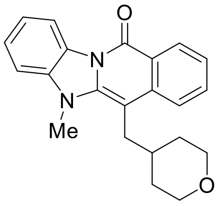

Prepared from **GPF**. Afforded the desired product **7** as a yellow solid, Mp = 106–107 °C. ^{1}H NMR (400 MHz, $CDCl_3$) δ = 8.82 (d, J = 8.0 Hz, 1H), 8.54 (d, J = 8.1 Hz, 1H), 7.72–7.50 (m, 2H), 7.39–7.28 (m, 2H), 7.15 (t, J = 7.7 Hz, 1H), 7.04 (d, J = 8.0 Hz, 1H), 3.98–3.81 (m, 2H), 3.79 (s, 3H), 3.22 (td, J = 11.6 Hz, J =2.4 Hz, 2H), 3.01 (d, J = 7.2 Hz, 2H),

1.78 (m, 1H), 1.55–1.35 (m, 4H). ^{13}C NMR (100 MHz, $CDCl_3$) δ = 159.45, 139.31, 138.04, 135.69, 131.76, 127.88, 127.76, 125.55, 122.20, 121.72, 120.81, 119.36, 116.88, 106.55, 89.14, 67.95, 36.60, 33.66, 32.82, 30.80. HRMS (ESI) m/z calculated for $C_{22}H_{22}N_2O_2Na$ $[M+Na]^+$ 369.1573, found 365.1575.

5-methyl-6-((tetrahydrofuran-2-yl)methyl)benzo[4,5]imidazo[1,2-*b*]isoquinolin-11(5*H*)-one (8)

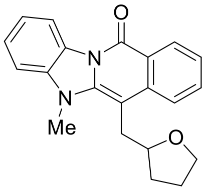

Prepared from **GPF**. Afforded the desired product **8** as a yellow solid, Mp = 97–98 °C. ^{1}H NMR (400 MHz, $CDCl_3$) δ = 8.85 (d, *J* = 8.1 Hz, 1H), 8.57 (dd, *J* = 8.0 Hz, *J* = 1.6 Hz, 1H), 7.77–7.56 (m, 2H), 7.39 – 7.30 (m, 2H), 7.19 (t, *J* = 7.5 Hz, 1H), 7.09 (d, *J* = 8.0 Hz, 1H), 4.01 (td, *J* = 8.5 Hz, *J* = 6.1 Hz, 1H), 3.87 (s, 3H), 3.75 (td, *J* = 8.5 Hz, *J* = 6.3 Hz, 1H), 3.63 – 3.49 (m, 2H), 3.26 (dd, *J* = 15.5 Hz, *J* = 9.3 Hz, 1H), 3.09 (dd, *J* = 15.6 Hz, *J* = 6.4 Hz, 1H), 2.71–2.56 (m, 1H), 2.03 (dtd, *J* = 13.7 Hz, *J* = 8.2 Hz, *J* = 5.9 Hz, 1H), 1.78–1.66 (m, 1H). ^{13}C NMR (100 MHz, $CDCl_3$) δ = 159.54, 139.46, 137.75, 135.71, 131.91, 130.16, 127.88, 125.60, 122.30, 121.41, 120.85, 119.47, 116.91, 106.62, 89.87, 71.82, 67.45, 40.07, 33.43, 31.59, 27.30. HRMS (ESI) m/z calculated for $C_{21}H_{20}N_2O_2Na$ $[M+Na]^+$ 355.1417, found 355.1413.

Rearrangement product derived from stanolone (9)

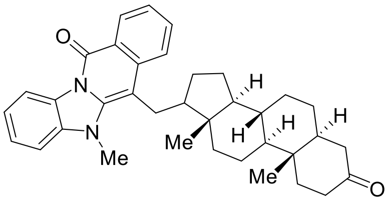

Prepared from **GPF**. Afforded the desired product **9** a yellow solid (dr = 4.6:1), Mp = 130–131 °C. ^{1}H NMR (400 MHz, $CDCl_3$) δ = 8.88 (ddd, *J* = 8.0 Hz, *J* = 4.4 Hz, *J* = 1.2 Hz, 1H), 8.64 – 8.52 (m, 1H), 7.71–7.63 (m, 2H), 7.45–7.28 (m, 2H), 7.21 (td, *J* = 7.8 Hz, *J* = 1.0 Hz, 1H), 7.12 (d, *J* = 8.0 Hz, 1H), 3.85 (s, 3H), 3.21–3.13 (m, 1H), 3.01 (dd, *J* = 15.0 Hz, *J* = 12.4 Hz, 1H), 2.42–2.20 (m, 3H), 2.16–1.98 (m, 3H), 1.79 (dd, *J* = 12.8 Hz, *J* = 3.8 Hz, 2H), 1.73–1.67 (m, 2H), 1.60–1.47 (m, 3H), 1.42–1.34 (m, 4H), 1.30–1.21 (m, 4H), 1.05 (s, 3H), 1.00 (s, 1H), 0.84 (s, 3H), 0.82 (s, 1H). ^{13}C NMR (100 MHz, $CDCl_3$) δ = 210.95, 158.06, 140.78, 138.54, 136.56, 130.62, 128.81, 128.01, 125.63, 122.30, 121.70, 120.97, 119.08, 117.10, 107.34, 106.20, 89.38, 55.48, 53.90, 50.74, 46.80, 45.09, 43.81, 38.70, 37.56, 36.56, 35.82, 33.92, 32.37, 28.92, 25.60, 24.62, 22.08, 20.70, 12.05. HRMS (ESI) m/z calculated for $C_{36}H_{42}N_2O_2Na$ $[M+Na]^+$ 557.3139, found 557.3138.

5-methyl-6-(2-methylbutyl)benzo[4,5]imidazo[1,2-*b*]isoquinolin-11(5*H*)-one (10)

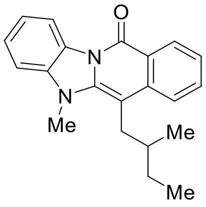

Prepared from **GPF**. Afforded the desired product **10** a yellow solid, Mp = 146–147 °C. ^{1}H NMR (400 MHz, $CDCl_3$) δ = 8.85–8.83 (dd, *J* = 1.2 Hz, *J* = 8.4 Hz, 1H), 8.57–8.54 (m, 1H), 7.45–7.58 (m, 2H), 7.33–7.28 (m, 2H), 7.17–7.13 (m, 1H), 7.04–7.01 (m, 1H), 3.78 (s, 3H), 3.03–2.87 (m, 2H), 1.72–1.64 (m, 1H), 1.49–1.39 (m, 1H), 1.27–1.17 (m, 1H), 0.89 (t, *J* = 7.6 Hz, 3H), 0.86 (d, *J* = 6.8 Hz, 3H). ^{13}C NMR (100 MHz, $CDCl_3$) δ = 159.56, 139.23, 138.29, 135.80,

131.58, 127.95, 127.68, 125.47, 122.07, 122.01, 120.64, 119.38, 116.89, 106.44, 90.72, 36.08, 33.58, 31.12, 29.38, 18.72, 11.78. HRMS (ESI) m/z calculated for $C_{21}H_{22}N_2ONa$ [M+Na]$^+$ 341.1624, found 341.1608.

diethyl 2-((5-methyl-11-oxo-5,11-dihydrobenzo[4,5]imidazo[1,2-*b*]isoquinolin-6-yl)methyl)malonate (11)

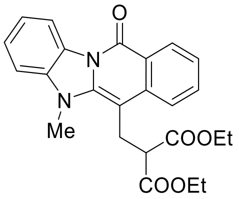

Prepared from **GPF**. Afforded the desired product **11** a yellow solid, Mp = 173–174 °C. ^{1}H NMR (400 MHz, $CDCl_3$) δ = 8.83 (dd, *J* = 8.1, *J* =1.2 Hz, 1H), 8.56 (dt, *J* = 8.1, *J* =1.1 Hz, 1H), 7.76–7.62 (m, 2H), 7.46–7.26 (m, 2H), 7.24–6.99 (m, 2H), 4.33–4.14 (m, 1H), 4.13–3.99 (m, 4H), 3.97 (s, 3H), 3.84 (s, 1H), 3.78 (t, *J* = 7.3 Hz, 1H). 1.38–1.17 (m, 3H), 1.07 (d, *J* = 7.0 Hz, 3H). ^{13}C NMR (100 MHz, $CDCl_3$) δ = 169.00, 159.72, 139.97, 137.34, 135.68, 132.36, 128.14, 127.79, 125.68, 122.49, 120.99, 120.90, 119.62, 116.90, 106.78, 86.30, 61.57, 51.59, 33.62, 23.93, 13.79. HRMS (ESI) m/z calculated for $C_{24}H_{24}N_2O_5Na$ [M+Na]$^+$ 443.1577, found 443.1568

5-methyl-6-phenethylbenzo[4,5]imidazo[1,2-*b*]isoquinolin-11(5*H*)-one (12)

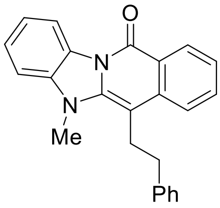

Prepared from **GPF**. Afforded the desired product **12** a yellow solid, Mp = 173–174 °C. ^{1}H NMR (400 MHz, $CDCl_3$) δ = 8.86 (d, *J* = 7.9 Hz, 1H), 8.59 (dd, *J* = 8.1 Hz, *J* = 1.5 Hz, 1H), 7.78 (d, *J* = 8.4 Hz, 1H), 7.69 (ddd, *J* = 8.3 Hz, *J* = 6.7 Hz, *J* = 1.5 Hz, 1H), 7.34 (dd, *J* = 7.7 Hz, *J* = 3.0 Hz, 5H), 7.26 (s, 1H), 7.19 (t, *J* = 7.7 Hz, 2H), 7.04 (d, *J* = 8.0 Hz, 1H), 3.78 (s, 3H), 3.44–3.34 (m, 2H), 3.02 (dd, *J* = 10.1 Hz, *J* = 6.4 Hz, 2H). ^{13}C NMR (100 MHz, $CDCl_3$) δ = 159.69, 141.12, 139.17, 137.84, 135.65, 132.21, 128.66, 128.21, 128.07, 126.37, 125.61, 122.33, 120.94, 120.89, 119.52, 116.99, 106.46, 90.22, 37.36, 33.04, 26.93. HRMS (ESI) m/z calculated for $C_{24}H_{21}N_2O$ [M+H]$^+$ 353.1648, found 353.1646.

ethyl 2-fluoro-3-(5-methyl-11-oxo-5,11-dihydrobenzo[4,5]imidazo[1,2-*b*]isoquinolin-6-yl)propanoate (13)

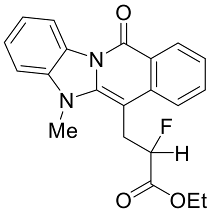

Prepared from **GPF**. Afforded the desired product **13** a yellow solid, Mp = 268–269 °C. ^{1}H NMR (400 MHz, $CDCl_3$) δ = 8.89 (d, *J* = 8.0 Hz, 1H), 8.62 (d, *J* = 8.1 Hz, 1H), 7.77–7.64 (m, 2H), 7.40 (dt, *J* = 13.9 Hz, *J* = 7.5 Hz, 2H), 7.25 (t, *J* = 7.7 Hz, 1H), 7.14 (d, *J* = 8.0 Hz, 1H), 5.17 (ddd, *J* = 49.1 Hz, *J* = 9.7 Hz, *J* = 3.3 Hz, 1H), 4.35 (q, *J* = 7.0 Hz, 2H), 3.94 (s, 3H), 3.84 (dd, *J* = 15.4 Hz, *J* = 9.8 Hz, 1H), 3.81–3.55 (m, 1H), 1.36 (t, *J* = 7.1 Hz, 3H). ^{13}C NMR (100 MHz, $CDCl_3$) δ = 169.35 (d, *J* = 20.0 Hz), 159.71, 140.47, 137.37, 135.34, 132.48, 128.25, 127.74, 125.74, 122.48, 121.10,

120.35, 119.37, 116.98, 106.65, 87.33 (d, *J* = 184.0 Hz), 83.79, 61.93, 33.33, 28.34 (d, *J* = 21.8 Hz), 14.11. ^{19}F NMR (376 MHz, $CDCl_3$) δ = -189.99. HRMS (ESI) m/z calculated for $C_{21}H_{19}FN_2O_3Na$ [M+Na]$^+$ 389.1272, found 389.1272.

6-(2,2-difluoro-3-oxo-3-phenylpropyl)-5-methylbenzo[4,5]imidazo[1,2-*b*]isoquinolin-11(5*H*)-one (14)

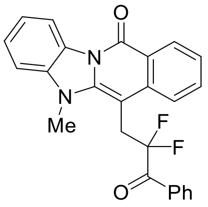

Prepared from **GPF**. Afforded the desired product **14** a yellow solid, Mp = 244–245 °C. ^{1}H NMR (400 MHz, $CDCl_3$) δ = 8.89 (d, *J* = 8.0 Hz, 1H), 8.60–8.53 (m, 1H), 8.01 (d, *J* = 8.5 Hz, 2H), 7.73 (d, *J* = 8.6 Hz, 1H), 7.68–7.57 (m, 2H), 7.42 (td, *J* = 7.6 Hz, *J* = 3.1 Hz, 3H), 7.35 (dd, *J* = 8.0 Hz, *J* = 6.8 Hz, 1H), 7.29–7.22 (m, 1H), 7.15 (d, *J* = 7.9 Hz, 1H), 4.19 (t, *J* = 5.3 Hz, 2H), 3.89 (s, 3H). ^{13}C NMR (100 MHz, $CDCl_3$) δ = 190.10 (t, *J* = 44.0 Hz), 158.04, 141.14, 135.30 (t, *J* = 321.0 Hz), 134.55, 131.78, 130.09, 130.06, 130.02, 128.65, 127.73, 125.73, 122.53, 121.78, 121.24, 119.25, 118.82, 117.01, 106.74, 80.45, 32.93, 28.63 (t, *J* = 24.0 Hz). ^{19}F NMR (376 MHz, $CDCl_3$) δ = -97.39 (t, *J* = 19.9 Hz, 2F). HRMS (ESI) m/z calculated for $C_{25}H_{18}F_2N_2O_2$ [M+H]$^+$ 417.1409, found 417.1409.

5-methyl-6-(2,2,9,9,9,9,9,9,9,9,9,9,9,9,9,9,9,9-octadecafluoro-9l17-nona-3,5,7-triyn-1-yl)benzo[4,5]imidazo[1,2-*b*]isoquinolin-11(5*H*)-one (15)

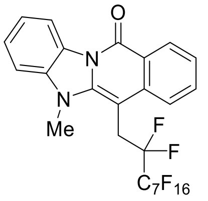

Prepared from **GPF**. Afforded the desired product **15** a yellow solid, Mp = 149–150 °C. ^{1}H NMR (400 MHz, $CDCl_3$) δ 8.83 (d, *J* = 8.0 Hz, 1H), 8.53 (d, *J* = 8.0 Hz, 1H), 7.64 (t, *J* = 7.5 Hz, 1H), 7.53 (d, *J* = 8.6 Hz, 1H), 7.35 (dt, *J* = 18.4 Hz , *J* = 7.6 Hz, 2H), 7.22 (s, 1H), 7.10 (d, *J* = 8.0 Hz, 1H), 3.97 (d, *J* = 23.1 Hz, 2H), 3.82 (s, 3H). ^{13}C NMR (100 MHz, $CDCl_3$) δ = 159.32, 141.32, 138.83, 135.05, 133.31, 128.54, 127.63, 126.48, 123.43, 121.51, 120.56, 119.25, 117.67, 107.24, 90.46, 77.92 , 41.52, 25.81 (t, *J* = 32.0 Hz).^{19}F NMR (376 MHz, $CDCl_3$) δ = -80.56, -80.58, -80.61, -111.67 – -112.21 (m, 2F) , -120.86 – -123.20 (m, 11F) , -125.57 – -126.29 (m, 2F). HRMS (ESI) m/z calculated for $C_{25}H_{14}F_{18}N_2O$ [M+H]$^+$ 700.0813, found 700.0815.

diethyl (1,1-difluoro-2-(5-methyl-11-oxo-5,11-dihydrobenzo[4,5]imidazo[1,2-*b*]isoquinolin-6-yl)ethyl)phosphonate (16)

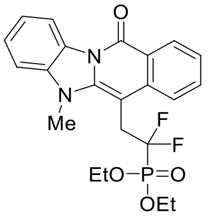

Prepared from **GPF**. Afforded the desired product **16** a yellow solid, Mp = 122–123 °C. ^{1}H NMR (400 MHz, $CDCl_3$) δ = 8.87 (dd, *J* = 8.1 Hz, *J* = 1.1 Hz, 1H), 8.55 (dd, *J* = 8.1 Hz, *J* = 1.5 Hz, 1H), 7.74–7.60 (m, 2H), 7.44–7.29 (m, 2H), 7.22 (td, *J* = 7.8 Hz, *J* = 1.1 Hz, 1H), 7.12 (d, *J* = 8.0 Hz, 1H), 4.33 (p, *J* = 7.3 Hz, 4H), 4.15–3.89 (m, 5H), 1.41 (t, *J* =

7.1 Hz, 6H). ^{13}C NMR (100 MHz, $CDCl_3$) δ = 159.81, 141.08, 138.62, 135.28, 132.06, 127.70, 127.61, 125.71, 122.47, 121.85, 119.12(t, *J* = 211.0 Hz), 106.70, 99.87, 77.32, 77.00, 64.75, 64.68, 32.64 (t, *J* = 4.0 Hz), 16.44, 16.39. ^{19}F NMR (376 MHz, $CDCl_3$) δ = -104.33. HRMS (ESI) m/z calculated for $C_{22}H_{24}F_2N_2O_4P$ $[M+H]^+$ 449.1436, found 449.1432.

6-(2,2-difluoro-2-(phenylsulfonyl)ethyl)-5-methylbenzo[4,5]imidazo[1,2-*b*]isoquinolin-11(5*H*)-one (17)

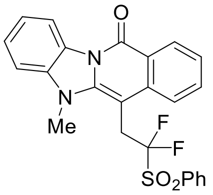

Prepared from **GPF**. Afforded the desired product **17** a yellow solid, Mp = 122–123 °C. ^{1}H NMR (400 MHz, $CDCl_3$) δ = 8.73 (d, *J* = 8.0 Hz, 1H), 8.43 (d, *J* = 8.0 Hz, 1H), 7.94 (dd, *J* = 7.7 Hz, *J* = 2.0 Hz, 2H), 7.66 (d, *J* = 7.4 Hz, 1H), 7.58–7.48 (m, 4H), 7.31 (dd, *J* = 10.3 Hz, *J* = 6.7 Hz, 2H), 7.12 (t, *J* = 7.7 Hz, 1H), 7.04 (d, *J* = 8.0 Hz, 1H), 4.44–4.10 (m, 2H), 3.82 (s, 3H). ^{13}C NMR (100 MHz, $CDCl_3$) δ = 159.49, 141.24, 138.20, 135.61(t, *J* = 55.0Hz), 132.36, 131.69, 131.24, 130.74, 129.67, 128.99, 127.70, 127.58, 127.22, 124.77, 122.68, 121.38, 119.57, 116.94, 106.04, 77.70, 32.68, 24.29 (t, *J* = 16.0 Hz). ^{19}F NMR (376 MHz, $CDCl_3$) δ = -102.03. HRMS (ESI) m/z calculated for $C_{24}H_{18}F_2N_2O_3SNa$ $[M+Na]^+$ 475.0898, found 475.0905.

ethyl 2,2-difluoro-3-(2,3,5-trimethyl-11-oxo-5,11-dihydrobenzo[4,5]imidazo[1,2-*b*]isoquinolin-6-yl)propanoate (18)

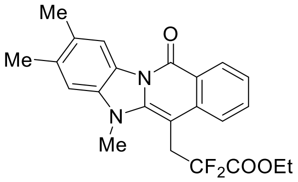

Prepared from **GPF**. Afforded the desired product **18** as a yellow solid, Mp = 122–123 °C. ^{1}H NMR (400 MHz, $CDCl_3$) δ = 8.55 (s, 1H), 8.50 (d, *J* = 8.0 Hz, 1H), 7.61–7.53 (m, 2H), 7.31–7.27 (m, 1H), 6.79 (s, 1H), 4.19–4.14 (q, *J* = 7.2 Hz, 2H), 3.91–3.74 (m, 5H), 2.33 (s, 3H), 2.31 (s, 3H), 1.17 (t, *J* = 7.2 Hz, 3H). ^{13}C NMR (100 MHz, $CDCl_3$) δ = 164.40 (t, *J* = 32.0 Hz), 159.39, 140.92, 138.05, 134.39, 133.27, 131.78, 129.49, 127.55, 125.49, 122.12, 121.27, 118.70, 117.50, 115.23, 107.71, 79.74, 63.14, 32.62, 29.86 (t, *J* = 24.0 Hz), 20.44, 19.81, 13.66. ^{19}F NMR (376 MHz, $CDCl_3$) δ = -103.61. HRMS (ESI) m/z calculated for $C_{23}H_{24}F_2N_2O_3$ $[M+H]^+$ 413.1671, found 413.1665.

ethyl 2,2-difluoro-3-(2-methoxy-5-methyl-11-oxo-5,11-dihydrobenzo[4,5]imidazo[1,2-*b*]isoquinolin-6-yl)propanoate (19)

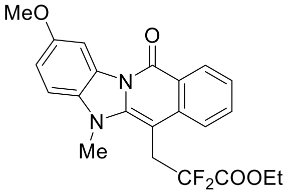

Prepared from **GPF**. Afforded the desired product **19** as a yellow solid, Mp = 162–163 °C. ^{1}H NMR (400 MHz, $CDCl_3$) δ 8.44 (d, *J* = 7.8 Hz, 2H), 7.62–7.48 (m, 2H), 7.31–7.14 (m, 1H), 6.91 (d, *J* = 4.1 Hz, 2H), 4.16 (m, 2H), 3.76–3.89 (m, 8H), 1.17 (t, *J* = 7.2 Hz, 3H). ^{13}C NMR (100 MHz, $CDCl_3$) δ = 164.68 (t, *J* = 32.0 Hz), 159.73, 154.74, 141.34, 138.20, 132.05, 129.17, 128.03, 127.55, 121.28 (t, *J* = 328.0 Hz), 113.04, 107.03, 102.22, 79.63, 63.17, 56.03, 32.75, 29.37 (t, *J*

= 29.0 Hz), 13.66. ^{19}F NMR (376 MHz, $CDCl_3$) δ = -103.24, -104.98. HRMS (ESI) m/z calculated for $C_{22}H_{21}F_2N_2O_4$ $[M+H]^+$ 415.1464, found 415.1465.

ethyl 3-(2-chloro-5-methyl-11-oxo-5,11-dihydrobenzo[4,5]imidazo[1,2-*b*]isoquinolin-6-yl)-2,2-difluoropropanoate (20)

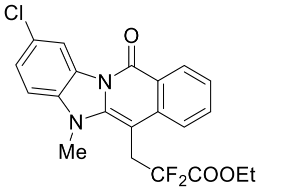

Prepared from **GPF**. Afforded the desired product **20** as a yellow solid, Mp = 234–235 °C. ^{1}H NMR (400 MHz, $CDCl_3$) δ = 8.75 (d, *J* = 2.0 Hz, 1H), 8.45 (dd, *J* = 8.2 Hz, , *J* = 1.5 Hz, 1H), 7.63 – 7.53 (m, 2H), 7.34–7.27 (m, 1H), 7.23 (d, *J* = 11.7 Hz, 1H), 6.92 (d, *J* = 8.6 Hz, 1H), 4.20 (q, *J* = 7.3 Hz, 2H), 3.84 (m, 5H), 1.20 (t, *J* = 7.3 Hz, 3H). ^{13}C NMR (100 MHz, $CDCl_3$) δ = 164.31 (t, *J* = 38.0 Hz), 159.41, 140.90, 138.08, 133.81, 132.39, 127.97, 127.64, 126.31, 125.65, 122.83, 121.56, 116.92 (t, *J* = 181.0 Hz)., 107.25, 82.71, 66.39, 35.88, 29.73 (t, *J* = 24.0 Hz), 12.54. ^{19}F NMR (376 MHz, $CDCl_3$) δ = -103.62. HRMS (ESI) m/z calculated for $C_{21}H_{18}ClF_2N_2O_3$ $[M+H]^+$ 419.0969, found 419.0970.

ethyl 2,2-difluoro-3-(5-methyl-11-oxo-2-(trifluoromethyl)-5,11-dihydrobenzo[4,5]imidazo[1,2-*b*]isoquinolin-6-yl)propanoate (21)

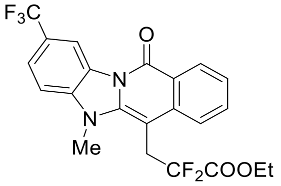

Prepared from **GPF**. Afforded the desired product **21** as a yellow solid, Mp = 249 –250 °C. ^{1}H NMR (400 MHz, $CDCl_3$) δ = 8.91 (d, *J* = 1.6 Hz, 1H), 8.55–8.32 (m, 1H), 7.62 (dt, *J* = 13.8 Hz, *J* = 8.4 Hz, 2H), 7.51 (dt, *J* = 8.4 Hz, *J* = 1.3 Hz, 1H), 7.36 (ddd, *J* = 7.9 Hz, *J* = 6.7 Hz, *J* = 1.3 Hz, 1H), 7.07 (d, *J* = 8.4 Hz, 1H), 4.26 (q, *J* = 7.1 Hz, 2H), 3.92 (m, 5H), 1.26 (t, *J* = 7.2 Hz, 3H). ^{13}C NMR (100 MHz, $CDCl_3$) δ = 164.33 (t, *J* = 30.0 Hz), 140.77, 137.88, 137.44, 132.53, 127.40 (d, *J* = 16.2 Hz), 125.65, 123.21 (q, *J* = 5.5 Hz, *J* = 4.0 Hz), 122.98, 121.76, 119.11, 117.68, 115.01 (t, *J* = 251.0 Hz), 114.03 (d, *J* = 4.4 Hz), 106.53, 81.12, 63.36, 32.88, 29.65 (t, *J* = 24.2 Hz), 13.72. ^{19}F NMR (376 MHz, $CDCl_3$) δ = -60.49 (s, 3F), -103.33, -105.08. HRMS (ESI) m/z calculated for $C_{22}H_{17}F_5N_2O_3SNa$ $[M+Na]^+$ 475.1052, found 475.1051.

ethyl 3-(2-cyano-5-methyl-11-oxo-5,11-dihydrobenzo[4,5]imidazo[1,2-*b*]isoquinolin-6-yl)-2,2-difluoropropanoate (22)

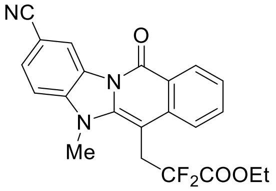

Prepared from **GPF**. Afforded the desired product **22** as a yellow solid, Mp = 125–126 °C. ^{1}H NMR (400 MHz, $CDCl_3$) δ = 8.89 (d, *J* = 1.6 Hz, 1H), 8.46–8.39 (m, 1H), 7.60 (dt, *J* = 13.8 Hz, *J* = 8.4 Hz, 2H), 7.49 (dt, *J* = 8.4 Hz, *J* = 1.3 Hz, 1H), 7.34 (m, 1H), 7.05 (d, *J* = 8.4 Hz, 1H), 4.24 (q, *J* = 7.1 Hz, 2H), 3.90 (m, 5H), 1.24 (t, *J* = 7.2 Hz, 3H). ^{13}C NMR (100 MHz, $CDCl_3$) δ = 164.49(t, *J* = 13.0 Hz), 155.65, 140.41, 138.18, 137.77, 132.73, 130.35, 127.59, 127.32, 123.60, 121.92, 119.33 (t, *J* = 31.0 Hz), 114.91, 107.15, 103.63, 81.75, 63.44, 33.00, 29.57 (t, *J* = 65.0 Hz), 13.76. ^{19}F NMR

(376 MHz, $CDCl_3$) δ = -103.11, -105.01. HRMS (ESI) m/z calculated for $C_{22}H_{18}F_2N_3O_3$ $[M+H]^+$ 410.1311, found 410.1311.

methyl 6-(3-ethoxy-2,2-difluoro-3-oxopropyl)-5-methyl-11-oxo-5,11-dihydrobenzo[4,5]imidazo[1,2-*b*]isoquinoline-2-carboxylate (23)

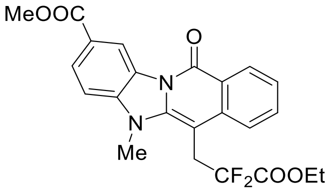

Prepared from **GPF**. Afforded the desired product **23** as a yellow solid, Mp = 270–271 °C. ^{1}H NMR (400 MHz, $CDCl_3$) δ = 9.30 (d, *J* = 1.7 Hz, 1H), 8.49 (d, *J* = 8.0 Hz, 1H), 8.00 (dd, *J* = 8.5 Hz, *J* = 1.7 Hz, 1H), 7.77–7.43 (m, 2H), 7.34 (t, *J* = 7.2 Hz, 1H), 7.02 (d, *J* = 8.5 Hz, 1H), 4.20 (q, *J* = 7.2 Hz, 2H), 3.91 (m, 8H), 1.20 (t, *J* = 7.2 Hz, 3H). ^{13}C NMR (100 MHz, $CDCl_3$) δ = 169.73, 164.24 (t, *J* = 34.0 Hz), 158.28, 140.94, 138.54, 137.89, 132.38, 128.21, 127.64, 127.17, 123.21, 122.95, 119.39, 117.78 (t, *J* = 273.0 Hz), 106.20, 81.22, 63.32, 52.08, 32.97, 29.71 (t, *J* = 24.0 Hz), 13.72. ^{19}F NMR (376 MHz, $CDCl_3$) δ = -103.08, -105.07. HRMS (ESI) m/z calculated for $C_{23}H_{21}F_2N_2O_5$ $[M+H]^+$ 443.1413, found 443.1413.

ethyl 3-(5,8-dimethyl-11-oxo-5,11-dihydrobenzo[4,5]imidazo[1,2-*b*]isoquinolin-6-yl)-2,2-difluoropropanoate (24)

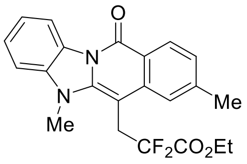

Prepared from **GPF**. Afforded the desired product **24** as a yellow solid, Mp = 221–222 °C. ^{1}H NMR (400 MHz, $CDCl_3$) δ = 8.76–8.74 (dd, *J* = 1.2 Hz, *J* = 8.0 Hz, 1H), 8.35 (d, *J* = 8.0 Hz, 1H), 7.30–7.23 (m, 2H), 7.16–7.12 (m, 1H), 7.07–7.05 (dd, *J* = 1.6 Hz, *J* = 8.4 Hz, 1H), 6.98 (d, *J* = 8.0 Hz, 1H), 4.18–4.12 (q, *J* = 7.2 Hz, 2H), 3.86–3.74 (m, 5H), 2.43 (s, 3H), 1.16 (t, *J* = 7.2 Hz, 3H). ^{13}C NMR (100 MHz, $CDCl_3$) δ = 164.38 (t, *J* = 32.0 Hz), 159.44, 142.44, 140.83, 138.13, 135.03, 127.48, 127.40, 125.46, 124.20, 120.94, 116.77, 115.16 (t, *J* = 251.0 Hz), 106.62, 79.68, 63.11, 32.58, 29.75 (t, *J* = 24.0 Hz), 22.37, 13.61. ^{19}F NMR (376 MHz, $CDCl_3$) δ = -103.55. HRMS (ESI) m/z calculated for $C_{22}H_{21}F_2N_2O_3$ $[M+H]^+$ 399.1515, found 399.1512.

ethyl 3-(5,7-dimethyl-11-oxo-5,11-dihydrobenzo[4,5]imidazo[1,2-*b*]isoquinolin-6-yl)-2,2-difluoropropanoate (25)

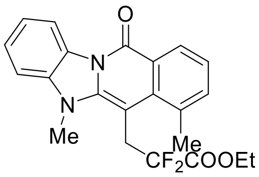

Prepared from **GPF**. Afforded the desired product **25** as a yellow solid, Mp = 184–185 °C. ^{1}H NMR (400 MHz, $CDCl_3$) δ = 8.77 (d, *J* = 8.0 Hz, 1H), 8.46 (d, *J* = 8.1 Hz, 1H), 7.44 (d, *J* = 7.2 Hz, 1H), 7.39 (t, *J* = 7.8 Hz, 1H), 7.30–7.21 (m, 2H), 7.16 (d, *J* = 8.0 Hz, 1H), 4.47–4.25 (m, 1H), 4.10 (dd, *J* = 29.0 Hz, *J* = 15.0 Hz, 1H), 3.83 (s, 3H), 3.69 (q, *J* = 7.6 Hz, 2H), 2.74 (s, 3H), 0.95 (t, *J* = 7.1 Hz, 3H). ^{13}C NMR (100 MHz, $CDCl_3$) δ = 164.12(t, *J* = 13.0 Hz), 159.90, 144.10, 138.59, 136.51, 135.84, 132.01, 127.70, 126.19, 125.87, 122.89, 121.57, 121.53, 116.81, 115.68 (t, *J* =244.0 Hz), 107.23,

78.47, 77.32, 77.00, 76.68, 62.85, 34.89, 32.55 (t, *J* = 24.0 Hz), 25.43, 13.37. ^{19}F NMR (376 MHz, $CDCl_3$) δ = -104.22 (d, *J* = 245.5 Hz), -107.51 (d, *J* = 245.6 Hz). HRMS (ESI) m/z calculated for $C_{22}H_{21}F_2N_2O_3$ $[M+H]^+$ 399.1515, found 399.1514.

ethyl 2,2-difluoro-3-(9-methoxy-5-methyl-11-oxo-5,11-dihydrobenzo[4,5]imidazo[1,2-*b*]isoquinolin-6-yl)propanoate (26)

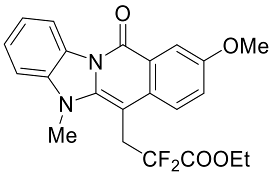

Prepared from **GPF**. Afforded the desired product **26** as a yellow solid, Mp = 202–203 °C. ^{1}H NMR (400 MHz, $CDCl_3$) δ = 8.85–8.82 (dd, *J* = 1.2 Hz, *J* = 8.0 Hz, 1H), 7.89 (d, *J* = 2.8 Hz, 1H), 7.58–7.55 (dd, *J* = 1.6 Hz, *J* = 9.2 Hz, 1H), 7.39–7.34 (td, *J* = 1.2 Hz, *J* = 7.6 Hz, 1H), 7.28–7.25 (m, 1H), 7.22–7.18 (m, 1H), 7.08 (d, *J* = 7.6 Hz, 1H), 4.23–4.17 (q, *J* = 7.2 Hz, 2H), 3.97–3.88 (m, 5H), 3.84 (s, 3H), 1.21 (t, *J* = 7.2 Hz, 3H). ^{13}C NMR (100 MHz, $CDCl_3$) δ = 164.37 (t, *J* = 32.0 Hz), 159.17, 155.61, 139.65, 135.40, 132.53, 127.57, 125.80, 123.37, 123.27, 120.96, 119.79, 117.01, 115.18, 106.59, 106.52, 80.04, 63.21, 55.53, 32.73, 30.07 (t, *J* = 24.0 Hz), 13.71. ^{19}F NMR (376 MHz, $CDCl_3$) δ = -103.19, -104.78. HRMS (ESI) m/z calculated for $C_{22}H_{21}F_2N_2O_4$ $[M+H]^+$ 415.1464, found 415.1466.

ethyl 3-(8,9-dimethoxy-5-methyl-11-oxo-5,11-dihydrobenzo[4,5]imidazo[1,2-*b*]isoquinolin-6-yl)-2,2-difluoropropanoate (27)

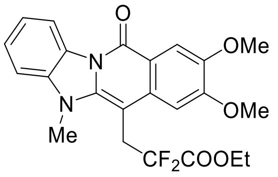

Prepared from **GPF**. Afforded the desired product **27** as a yellow solid, Mp = 249–250 °C. ^{1}H NMR (400 MHz, $CDCl_3$) δ = 8.86 (d, *J* = 7.6 Hz, 1H), 7.88 (s, 1H), 7.39 (t, *J* = 7.8 Hz, 1H), 7.23 (t, *J* = 7.8 Hz, 1H), 7.10 (d, *J* = 8.1 Hz, 1H), 6.94 (s, 1H), 4.14 (q, *J* = 7.2 Hz, 2H), 4.02 (d, *J* = 5.0 Hz, 6H), 4.00 – 3.92 (m, 2H), 3.89 (s, 3H), 1.11 (t, *J* = 7.1 Hz, 3H). ^{13}C NMR (100 MHz, $CDCl_3$) δ = 164.61 (t, *J* = 139.0 Hz), 158.77, 153.63, 146.44, 140.27, 135.15, 134.12, 127.72,125.61, 117.77, 116.92 (t, *J* = 416.0 Hz), 115.25, 106.89, 106.67, 102.04, 79.80, 63.27, 56.06, 55.86, 32.86 (t, *J* = 3.0 Hz), 29.67, 13.63. ^{19}F NMR (376 MHz, $CDCl_3$) δ = -102.68. HRMS (ESI) m/z calculated for $C_{23}H_{23}F_2N_2O_5$ $[M+H]^+$ 445.1570, found 445.1570.

ethyl 2,2-difluoro-3-(8-fluoro-5-methyl-11-oxo-5,11-dihydrobenzo[4,5]imidazo[1,2-*b*]isoquinolin-6-yl)propanoate (28)

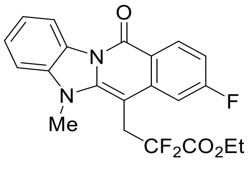

Prepared from **GPF**. Afforded the desired product **28** as a yellow solid, Mp = 255–256 °C ^{1}H NMR (400 MHz, $CDCl_3$) δ = 8.78–8.76 (dd, *J* = 1.2 Hz, *J* = 7.6 Hz, 2H), 8.49–8.45 (dd, *J* = 6.4 Hz, *J* = 9.2 Hz, 1H), 7.38–7.34 (td, *J* = 1.2 Hz, *J* = 7.6 Hz, 1H), 7.23–7.15 (m, 1H), 7.08–7.05 (m, 1H), 7.00–6.95 (m, 1H), 4.29–4.24 (q, *J* = 7.2 Hz, 2H), 3.89–3.74 (m, 5H), 1.27 (t, *J* = 7.2 Hz, 3H). ^{13}C NMR (100 MHz, $CDCl_3$) δ =167.07, 164.25(t, *J* = 26.0 Hz), 158.57, 141.75, 140.65

(d, J = 10.5 Hz), 134.85, 130.80 (d, J = 10.6 Hz), 127.42, 125.84, 121.51, 116.84, 115.59, 115.05, 111.45 (d, J = 24.1 Hz), 106.97, 106.40 (d, J = 23.6 Hz), 79.65, 63.34, 32.66, 30.16 (t, J = 24.3 Hz), 13.74. ^{19}F NMR (376 MHz, $CDCl_3$) δ = -105.58, -124.07. HRMS (ESI) m/z calculated for $C_{21}H_{18}F_3N_2O_5$ $[M+H]^+$ 403.1264, found 403.1265.

ethyl 2,2-difluoro-3-(5-methyl-11-oxo-9-(trifluoromethyl)-5,11-dihydrobenzo[4,5]imidazo[1,2-*b*]isoquinolin-6-yl)propanoate (29)

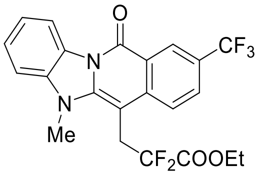

Prepared from **GPF**. Afforded the desired product **29** as a yellow solid, Mp = 193–194 °C. ^{1}H NMR (400 MHz, $CDCl_3$) δ = 8.89–8.70 (m, 2H), 7.75–7.58 (m, 2H), 7.41 (td, J = 7.8, 1.2 Hz, 1H), 7.30–7.20 (m, 1H), 7.14 (d, J = 8.0 Hz, 1H), 4.28 (q, J = 7.1 Hz, 2H), 3.93 (m, 5H), 1.28 (t, J = 7.2 Hz, 3H). ^{13}C NMR (100 MHz, $CDCl_3$) δ = 164.00 (t, J = 30.0 Hz), 140.57, 137.68, 137.24, 132.33, 127.11 (d, J = 16.2 Hz), 125.44, 123.03 (q, J = 5.5 Hz, J = 4.0 Hz) 122.78, 121.56, 118.91, 117.48, 117.32 (t, J = 251.0 Hz), 114.80, 113.84, 113.08 (d, J = 4.4 Hz), 106.33, 80.91, 63.15, 32.68, 29.45 (t, J = 24.2 Hz), 13.52. ^{19}F NMR (376 MHz, $CDCl_3$) δ = -61.79(s, 3F), -102.85, -104.86. HRMS (ESI) m/z calculated for $C_{22}H_{17}F_5N_2O_3Na$ $[M+Na]^+$ 475.1052, found 475.1052.

ethyl 2,2-difluoro-3-(5-isopropyl-11-oxo-5,11-dihydrobenzo[4,5]imidazo[1,2-*b*]isoquinolin-6-yl)propanoate (30)

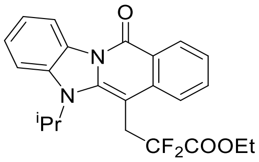

Prepared from **GPF**. Afforded the desired product **30** as a yellow solid, Mp = 146–147 °C ^{1}H NMR (400 MHz, $CDCl_3$) δ = 8.99–8.97 (m, 1H), 8.58–8.56 (dd, J = 1.6 Hz, J = 8.0 Hz, 1H), 7.78–7.76 (m, 1H), 7.69–7.65 (m, 1H), 7.46–7.43 (m, 1H), 7.39–7.34 (m, 2H), 7.28–7.24 (m, 1H), 5.25–5.15 (m, 1H), 4.26–4.20 (q, J = 7.2 Hz, 2H), 3.97 (t, J = 16.0 Hz, 2H), 1.71 (s, 3H), 1.69 (s, 3H), 1.24–1.21 (m, 3H). ^{13}C NMR (100 MHz, $CDCl_3$) δ = 164.52 (t, J = 32.0 Hz), 159.91, 142.04, 139.69, 138.55, 132.86, 132.29, 129.27, 127.86, 125.13, 122.73, 121.73, 121.35, 119.44, 117.40, 112.89, 111.23, 80.31, 77.32, 77.00, 76.68, 63.29, 49.97, 31.68 (t, J = 24.0 Hz), 20.41, 13.76. ^{19}F NMR (376 MHz, $CDCl_3$) δ = -102.39. HRMS (ESI) m/z calculated for $C_{23}H_{23}F_2N_2O_3$ $[M+H]^+$ 413.1671, found 413.1666

ethyl 3-(5-benzyl-11-oxo-5,11-dihydrobenzo[4,5]imidazo[1,2-*b*]isoquinolin-6-yl)-2,2-difluoropropanoate (31)

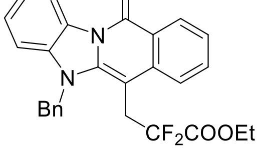

Prepared from **GPF**. Afforded the desired product **31** as a yellow solid, Mp = 251–252 °C. ^{1}H NMR (400 MHz, $CDCl_3$) δ = 8.99 (d, J = 8.0 Hz, 1H), 8.61 (d, J = 8.1 Hz, 1H), 7.67 (d, J = 5.0 Hz, 2H), 7.38 (d, J = 7.7 Hz, 2H), 7.31 (d, J = 7.6 Hz, 4H). 7.16 (d, J = 8.0 Hz, 1H), 7.13–7.08 (m, 2H), 5.66 (s, 2H), 4.24 (q, J = 7.2 Hz, 2H), 3.77–3.59 (m, 2H), 1.22 (t, J = 7.2 Hz, 3H). ^{13}C NMR (100 MHz, $CDCl_3$) δ = 164.42 (t, J = 31.7 Hz), 160.00, 140.63, 138.24, 136.01, 135.31,

132.35, 129.31, 127.99, 127.89, 127.86, 126.09, 125.33, 122.89, 121.82, 121.59, 119.45, 117.32,115.41 (t, J = 256.0 Hz), 106.97, 80.34 , 63.27, 47.60, 30.15 (t, J = 24.0 Hz), 13.77.^{19}F NMR (376 MHz, $CDCl_3$) δ = -103.61, -104.74. HRMS (ESI) m/z calculated for $C_{27}H_{22}F_2N_2O_3Na$ $[M+Na]^+$ 483.1491, found 483.1496.

ethyl 2,2-difluoro-3-(1-methyl-5-oxo-2,3-diphenyl-1,5-dihydroimidazo[1,2-*b*]isoquinolin-10-yl)propanoate (32)

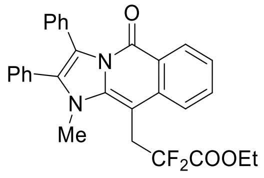

Prepared from **GPF**. Afforded the desired product **32** as a yellow solid, Mp = 74–75 °C. ^{1}H NMR (400 MHz, $CDCl_3$) δ = 8.41 (dd, J = 8.3 Hz , J =1.4 Hz, 1H), 7.71 (d, J = 8.6 Hz, 1H), 7.60 (m, 1H), 7.34 (dt, J = 4.2 Hz, J =2.8 Hz, 3H), 7.32–7.28 (m, 2H), 7.27 (d, J = 2.2 Hz, 2H), 7.25 (m, 2H), 7.24–7.22 (m, 1H), 7.21–7.18 (m, 1H), 4.21–4.01 (m, 4H), 3.69 (s, 3H), 1.15 (t, J = 7.2 Hz, 3H). ^{13}C NMR (100 MHz, $CDCl_3$) δ = 164.53 (t, J = 45.0 Hz), 156.77, 141.30, 137.84, 133.41, 131.49, 131.05, 130.88, 130.34, 129.12, 128.61, 128.15, 127.56, 127.00, 121.59, 120.96, 120.84, 118.07, 115.60, 79.38, 63.15, 35.12, 30.32 (t, J = 24.0 Hz), 13.64. ^{19}F NMR (376 MHz, $CDCl_3$) δ = -103.61 HRMS (ESI) m/z calculated for $C_{29}H_{24}F_2N_2O_3Na$ $[M+Na]^+$ 509.1647, found 509.1652.

ethyl 2,2-difluoro-3-(1-methyl-5-oxo-3-phenyl-1,5-dihydroimidazo[1,2-*b*]isoquinolin-10-yl)propanoate (33)

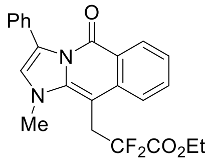

Prepared from **GPF**. Afforded the desired product **33** as a yellow solid, Mp = 148–149 °C. ^{1}H NMR (400 MHz, $CDCl_3$) δ = 8.43–8.41 (m, 1H), 7.68–7.64 (m, 1H), 7.61–7.57 (m, 1H), 7.47–7.43 (m, 2H), 7.42–7.36 (m, 3H), 7.23–7.19 (m, 1H), 6.61 (s, 1H), 4.22–4.16 (q, J = 7.2 Hz, 2H), 4.02–3.94 (m, 2H), 3.91 (s, 3H), 1.19 (t, J = 7.6 Hz, 3H). ^{13}C NMR (100 MHz, $CDCl_3$) δ = 164.51 (t, J = 32.0 Hz), 158.24, 140.45, 137.79, 131.56, 129.98, 129.69, 128.05, 127.68, 127.19, 124.77, 123.61, 121.50, 120.71, 117.39, 115.35, 79.33, 63.21, 37.35, 30.07 (t, J = 25.0 Hz), 13.68. ^{19}FNMR (376 MHz, $CDCl_3$) δ = -100.40 (d, J = 274.8 Hz, 1F), -103.23 (d, J = 274.1 Hz, 1F). HRMS (ESI) m/z calculated for $C_{23}H_{20}F_2N_2O_3Na$ $[M+Na]^+$ 433.1334, found 433.1332.

ethyl 2,2-difluoro-3-(1-methyl-5-oxo-1,5-dihydroimidazo[1,2-*b*]isoquinolin-10-yl)propanoate (34)

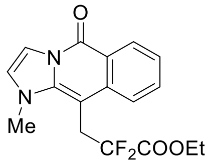

Prepared from **GPF**. Afforded the desired product 34 as a yellow solid, Mp = 243–244 °C. ^{1}H NMR (400 MHz, $CDCl_3$) δ = 8.46 (m, 1H), 7.72–7.49 (m, 3H), 7.28–7.20 (m, 1H), 6.70 (d, J = 2.5 Hz, 1H), 4.16 (q, J = 7.1 Hz, 2H), 3.86 (m, 5H), 1.15 (t, J = 7.2 Hz, 3H). ^{13}C NMR (100 MHz, $CDCl_3$) δ = 164.24 (t, J = 18.0 Hz), 157.36, 139.08, 137.90, 131.38, 127.71, 123.66, 121.33, 121.04, 115.93 (t, J =287.0 Hz), 112.70, 106.60, 79.65, 64.73, 38.88, 29.95 (t, J = 24.0 Hz), 14.35. ^{19}F NMR (376 MHz, $CDCl_3$) δ = -104.38 (t, J = 14.1 Hz, 2F). HRMS (ESI) m/z calculated for $C_{17}H_{17}F_2N_2O_3$ $[M+H]^+$ 335.1202, found 335.1198.

ethyl 2,2-difluoro-3-(1-methyl-5-oxo-2,3-di-*p*-tolyl-1,5-dihydroimidazo[1,2-*b*]isoquinolin-10-yl)propanoate (35)

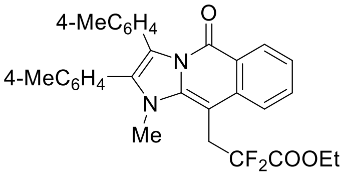

Prepared from **GPF**. Afforded the desired product **35** as a yellow solid, Mp = 265–266 °C. ^{1}H NMR (400 MHz, $CDCl_3$) δ = 8.41 (d, *J* = 8.1 Hz, 1H), 7.70 (d, *J* = 8.7 Hz, 1H), 7.58 (dd, *J* = 8.6 Hz, *J* = 6.7 Hz, 1H), 7.21–7.12 (m, 7H), 7.08 (d, *J* = 7.8 Hz, 2H), 4.19–4.01 (m, 4H), 3.67 (s, 3H), 2.34 (s, 3H), 2.33 (s, 3H), 1.15 (t, *J* = 7.3 Hz, 3H). ^{13}C NMR (100 MHz, $CDCl_3$) δ = 164.55 (t, *J* = 32.0 Hz), 158.14, 141.35, 139.07, 137.81, 137.14, 133.25, 131.36, 130.88, 130.72, 129.33, 128.15, 127.79, 127.44, 124.62, 121.42, 120.79, 118.07 (t, *J* = 246.0 Hz), 79.22, 63.09, 35.04, 30.34 (t, *J* = 34.0 Hz), 21.34, 21.29, 13.62. ^{19}F NMR (376 MHz, $CDCl_3$) δ = -104.45 (t, *J* = 14.1 Hz, 2F). HRMS (ESI) m/z calculated for $C_{31}H_{29}F_2N_2O_3$ $[M+H]^+$ 515.2141, found 515.2141.

ethyl 3-(2,3-bis(4-isopropylphenyl)-1-methyl-5-oxo-1,5-dihydroimidazo[1,2-*b*]isoquinolin-10-yl)-2,2-difluoropropanoate (36)

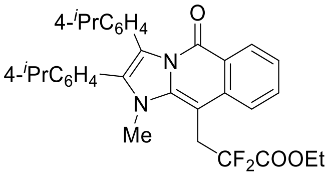

Prepared from **GPF**. Afforded the desired product **36** as a yellow solid, Mp = 60–61 °C. ^{1}H NMR (400 MHz, $CDCl_3$) δ = 8.42 (d, *J* = 8.4 Hz, 1H), 7.70 (d, *J* = 8.6 Hz, 1H), 7.63–7.54 (m, 1H), 7.24–7.09 (m, 9H), 4.18–4.03 (m, 4H), 3.67 (s, 3H), 2.89 (td, *J* = 6.9, 4.0 Hz, 2H), 1.25 (s, 6H), 1.23 (s, 6H), 1.13 (t, *J* = 7.3 Hz, 3H). ^{13}C NMR (100 MHz, $CDCl_3$) δ = 172.71 (t, *J* = 35.0 Hz), 164.63, 158.25, 149.80, 147.77, 141.42, 137.82, 133.42, 131.42, 130.97, 130.84, 128.26, 127.69, 126.63, 125.10, 124.95, 121.44, 120.93, 118.05 (t, *J* = 239.0 Hz), 79.26, 63.13, 35.25, 33.82, 33.74, 30.42 (t, *J* = 32.0 Hz), 23.80, 23.73, 13.65. ^{19}F NMR (376 MHz, $CDCl_3$) δ = -104.67 (t, *J* = 14.1 Hz, 2F). HRMS (ESI) m/z calculated for $C_{35}H_{37}F_2N_2O_3$ $[M+H]^+$ 571.2767, found 571.2764.

ethyl 3-(2,3-bis(4-methoxyphenyl)-1-methyl-5-oxo-1,5-dihydroimidazo[1,2-*b*]isoquinolin-10-yl)-2,2-difluoropropanoate (37)

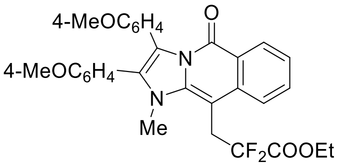

Prepared from **GPF**. Afforded the desired product **37** as a yellow solid, Mp = 130–131 °C. ^{1}H NMR (400 MHz, $CDCl_3$) δ = 8.45–8.36 (m, 1H), 7.71 (d, *J* = 8.6 Hz, 1H), 7.59 (t, *J* = 7.5 Hz, 1H), 7.20 (dd, *J* = 24.2 Hz, *J* = 8.4 Hz, 5H), 6.93–6.85 (m, 2H), 6.82 (d, *J* = 8.4 Hz, 2H), 4.14 (p, *J* = 7.4 Hz, 4H), 3.81 (s, 3H), 3.79 (s, 3H), 3.69 (s, 3H), 1.15 (t, *J* = 7.2 Hz, 3H). ^{13}C NMR (100 MHz, $CDCl_3$) δ = 164.54 (t, J = 33.0 Hz), 159.98, 158.83, 158.21, 141.31, 137.78, 132.29 (t, *J* = 94.0 Hz), 131.35, 128.13, 122.79, 121.41, 120.78, 120.48, 119.67, 118.05, 114.11, 112.58, 63.10, 55.20, 55.06, 39.27, 35.04, 30.36 (t, *J* = 25 Hz) 20.99, 13.63. ^{19}F NMR (376 MHz, $CDCl_3$) δ = -104.74 (t, *J* = 15.0 Hz). HRMS (ESI) m/z calculated for $C_{31}H_{28}F_2N_2O_5Na$ $[M+Na]^+$ 569.1859, found 569.1857.

ethyl 2,2-difluoro-3-(1-methyl-5-oxo-2,3-bis(4-(trifluoromethyl)phenyl)-1,5-dihydroimidazo[1,2-*b*]isoquinolin-10-yl)propanoate (38)

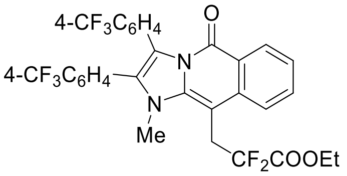

Prepared from **GPF**. Afforded the desired product **38** as a yellow solid, Mp = 200–201 °C. ^{1}H NMR (400 MHz, $CDCl_3$) δ = 8.39–8.37 (dd, *J* = 1.6 Hz, *J* = 8.4 Hz, 1H), 7.75 (d, *J* = 8.8 Hz, 1H), 7.66–7.61 (m, 3H), 7.54–7.52 (m, 2H), 7.39–7.45 (m, 4H), 7.27–7.23 (m, 1H), 4.21–4.16 (q, *J* = 7.2 Hz, 2H), 4.07 (t, *J* = 16.0 Hz, 2H), 3.68 (s, 3H), 1.19 (t, *J* = 7.2 Hz, 3H). ^{13}C NMR (100 MHz, $CDCl_3$) δ = 164.34 (t, *J* = 33.0 Hz), 158.03, 141.14, 137.92, 133.56, 132.78, 131.88, 131.64, 131.22, 131.12, 130.86, 129.77 (q, *J* = 33.0 Hz), 127.93, 125.88, 125.84, 125.37, 124.88, 124.09, 124.05, 122.66, 122.19, 121.14, 120.16, 118.08, 115.47, 112.95, 80.17, 77.32, 77.00, 76.68, 63.24, 35.19, 30.13 (t, *J* = 24.0 Hz), 13.63. ^{19}F NMR (376 MHz, $CDCl_3$) δ = -62.47(s, 3F), -62.79(s, 3F), -100.15 – -100.87 (m, 1F), -102.87 – -103.59 (m, 1F). HRMS (ESI) m/z calculated for $C_{31}H_{23}F_8N_2O_3$ $[M+H]^+$ 623.1575, found 623.1568.

ethyl 3-(2,3-bis(4-chlorophenyl)-1-methyl-5-oxo-1,5-dihydroimidazo[1,2-b]isoquinolin-10-yl)-2,2-difluoropropanoate (39)

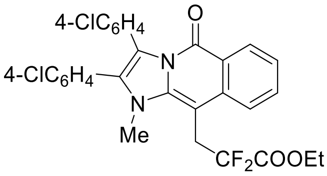

Prepared from **GPF**. Afforded the desired product **39** as a yellow solid, Mp = 76–77 °C. ^{1}H NMR (400 MHz, $CDCl_3$) δ = 8.38 (d, *J* = 8.1 Hz, 1H), 7.71 (d, *J* = 8.6 Hz, 1H), 7.61 (t, *J* = 7.9 Hz, 1H), 7.35 (d, *J* = 8.2 Hz, 2H), 7.28–7.18 (m, 5H), 7.16 (d, *J* = 8.2 Hz, 2H), 4.20–4.01 (m, 4H), 3.67 (s, 3H), 1.17 (t, *J* = 7.1 Hz, 3H) ^{13}C NMR (100 MHz, $CDCl_3$) δ = 164.42 (t, *J* = 23.0 Hz), 158.09, 141.18, 137.84, 135.64, 133.83, 132.64, 132.20, 132.07, 131.72, 129.20, 128.55, 128.01, 127.43, 125.69, 121.96, 120.03, 118.08 (t, *J* = 257.0 Hz), 79.81, 63.20, 35.10, 30.20 (t, *J* = 25.0 Hz), 13.66. ^{19}F NMR (376 MHz, $CDCl_3$) δ = -124.05(s, 2F). HRMS (ESI) m/z calculated for $C_{29}H_{23}F_2Cl_2N_2O_3$ $[M+H]^+$ 555.1048, found 555.1051.

ethyl 3-(2,3-bis(4-fluorophenyl)-1-methyl-5-oxo-1,5-dihydroimidazo[1,2-*b*]isoquinolin-10-yl)-2,2-difluoropropanoate (40)

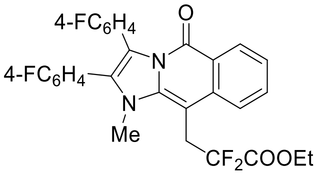

Prepared from GPF. Afforded the desired product 40 as a yellow solid, Mp = 125–126 °C. ^{1}H NMR (400 MHz, $CDCl_3$) δ = 8.39 (d, J = 8.1 Hz, 1H), 7.72 (d, J = 8.6 Hz, 1H), 7.65–7.58 (m, 1H), 7.36 (d, J = 8.3 Hz, 2H), 7.27–7.22 (m, 5H), 7.18 (t, J = 8.2 Hz, 2H), 4.21–4.00 (m, 4H), 3.68 (s, 3H), 1.18 (t, J = 7.1 Hz, 3H). ^{13}C NMR (100 MHz, $CDCl_3$) δ = 164.31 (t, *J* = 372.0 Hz), 162.7 (t, *J* = 178.0 Hz), 161.04, 159.46 (t, *J* = 251.0 Hz), 141.20, 137.89, 132.83, 132.74, 131.69, 128.07, 126.24, 123.42, 121.88, 120.95, 120.18, 118.11, 116.21, 115.99, 114.37, 114.15, 79.44 , 63.19, 53.39,

35.02, 30.03 (t, J = 26.0 Hz), 13.67. ^{19}F NMR (376 MHz, $CDCl_3$) δ = -110.32, -113.60, -124.08 (s, 2F). HRMS (ESI) m/z calculated for C29H22F4N2O3Na $[M+Na]^+$ 545.1459, found 545.1459.

Rearrangement product derived from Ketoconazole (41)

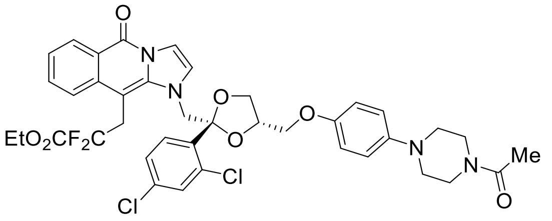

Prepared from **GPF** using DPEPhos as liangd. Afforded the desired product **41** as a pink solid, Mp = 115–116 °C. ^{1}H NMR (400 MHz, $CDCl_3$) δ = 8.50 (d, J = 8.2 Hz, 1H), 7.79 (d, J = 2.9 Hz, 1H), 7.71 (d, J = 8.4 Hz, 1H), 7.66 (d, J = 8.3 Hz, 1H), 7.57 (t, J = 7.4 Hz, 1H), 7.52 (d, J = 2.3 Hz, 1H), 7.39–7.33 (m, 1H), 7.02 (d, J = 2.9 Hz, 1H), 6.87 (td, J = 9.0 Hz, J = 8.5 Hz, J = 4.6 Hz, 1H), 6.62 (s, 2H), 6.26 (s, 2H), 4.80 (s, 2H), 4.32 (p, J = 5.8 Hz, 2H), 4.13 (q, J = 7.1 Hz, 3H), 3.86–3.70 (m, 4H), 3.59 (q, J = 7.6 Hz, J = 6.1 Hz, 4H), 3.01 (d, J = 5.6 Hz, 2H), 2.96 (t, J = 5.2 Hz, 2H), 2.14 (d, J = 3.5 Hz, 3H), 1.11 (t, J = 7.1 Hz, 3H). ^{13}C NMR (100 MHz, $CDCl_3$) δ = 169.42, 164.46 (t, J = 32.0 Hz), 156.44, 152.30, 145.45, 139.06, 138.11, 136.19, 134.36, 132.95, 131.54, 131.47, 129.56, 127.76, 127.39, 125.15, 121.54, 118.57 (t, J = 224.0 Hz), 114.47, 109.15, 106.57, 80.85, 74.62, 67.70, 67.02, 63.09, 51.51, 50.87, 50.53, 46.32, 41.42,29.92 (t, J = 23.0 Hz), 21.35, 3.64. ^{19}F NMR (376 MHz, $CDCl_3$) δ = -103.05, -104.77. HRMS (ESI) m/z calculated for $C_{39}H_{38}F_2Cl_2N_4O_7Na$ $[M+Na]^+$ 805.1979, found 805.1985.

ethyl 2,2-difluoro-3-(5-oxo-5*H*-thiazolo[3,2-*b*]isoquinolin-10-yl)propanoate (42)

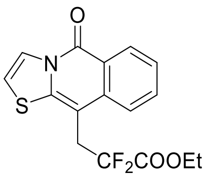

Prepared from **GPF**. Afforded the desired product **42** as a yellow solid, Mp = 116–117 °C. ^{1}H NMR (400 MHz, $CDCl_3$) δ = 8.56 (dd, J = 8.3 Hz, J = 1.5 Hz, 1H), 8.07 (d, J = 4.7 Hz, 1H), 7.78–7.72 (m, 1H), 7.69 (d, J = 8.3 Hz, 1H), 7.48 (t, J = 7.6 Hz, 1H), 6.79 (s, 1H), 4.26 (q, J = 7.0 Hz, 2H), 3.67 (t, J = 16.5 Hz, 2H), 1.24 (t, J = 14.6 Hz, 3H). ^{13}C NMR (100 MHz, $CDCl_3$) δ = 164.03 (t, J = 31.0 Hz), 159.20, 143.03, 136.07, 132.79, 128.40, 124.96, 124.02, 121.49, 120.70, 115.73 (t, J = 230.0 Hz), 109.37, 97.59, 63.32, 35.29 (t, J = 25.0 Hz), 13.76. ^{19}F NMR (376 MHz, $CDCl_3$) δ = -102.35. HRMS (ESI) m/z calculated for $C_{16}H_{14}F_2NO_3S$ $[M+H]^+$ 338.0657, found 338.0655.

ethyl 2,2-difluoro-3-(11-oxo-11*H*-benzo[4,5]thiazolo[3,2-*b*]isoquinolin-6-yl)propanoate (43)

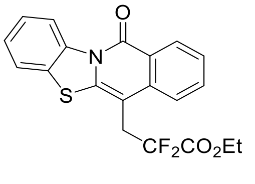

Prepared from **GPF**. Afforded the desired product **43** as a yellow solid, Mp = 103–104 °C. ^{1}H NMR (400 MHz, $CDCl_3$) δ = 9.16–9.13 (m, 1H), 8.58–8.55 (m, 1H), 7.73–7.69 (m, 1H), 7.62–7.59 (m, 1H), 7.53–7.51 (m, 1H), 7.49–7.45 (m, 1H), 7.44–7.40 (m, 1H), 7.37–7.33 (m, 1H), 4.29–4.24 (q, J = 7.2 Hz, 2H), 3.66–3.57 (m, 2H), 1.25 (t, J = 7.2 Hz, 3H). ^{13}C NMR (100 MHz, $CDCl_3$) δ = 163.99 (t, J = 32.0 Hz), 161.56, 141.85, 138.88, 135.27, 132.84, 128.44, 126.36, 126.23, 125.42, 125.11, 122.93, 121.49, 121.11, 119.81, 115.37 (t, J = 253.0 Hz), 98.58, 63.32, 35.22 (t, J = 25.0 Hz), 13.76. ^{19}F NMR (376 MHz, $CDCl_3$) δ = -102.37 (t, J = 16.4 Hz, 2F). HRMS (ESI) m/z calculated for $C_{20}H_{15}F_2NO_3SNa$ $[M+Na]^+$

410.0633, found 410.0632.

ethyl 2,2-difluoro-3-(3-methyl-11-oxo-11*H*-benzo[4,5]thiazolo[3,2-*b*]isoquinolin-6-yl)propanoate (44)

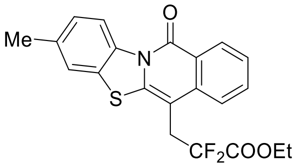

Prepared from **GPF**. Afforded the desired product **44** as a yellow solid, Mp = 167–168 °C. ^{1}H NMR (400 MHz, $CDCl_3$) δ = 9.02 (d, *J* = 8.6 Hz, 1H), 8.58 (d, *J* = 8.2 Hz, 1H), 7.72 (t, *J* = 7.7 Hz, 1H), 7.63 (d, *J* = 8.2 Hz, 1H), 7.49 (t, *J* = 7.6 Hz, 1H), 7.34 (s, 1H), 7.23 (d, *J* = 8.7 Hz, 1H), 4.27 (q, *J* = 7.1 Hz, 2H), 3.64 (t, *J* = 16.5 Hz, 2H), 2.43 (s, 3H), 1.29 (t, *J* = 7.2 Hz, 3H). ^{13}C NMR (100 MHz, $CDCl_3$) δ = 165.48 (t, *J* = 54.0 Hz), 161.49, 142.06, 136.76, 136.54, 135.33, 132.76, 128.44, 127.29, 125.40, 125.11, 122.94, 121.51, 121.37, 119.52, 115.40 (t, *J* = 209.0 Hz), 98.61, 63.33, 35.27 (t, *J* = 24.0 Hz), 29.67, 21.20, 13.78. ^{19}F NMR (376 MHz, $CDCl_3$) δ = -97.49 – -106.40 (m, 2F). HRMS (ESI) m/z calculated for $C_{21}H_{18}F_2NO_3S$ $[M+H]^+$402.0970, found 402.0972.

ethyl 2,2-difluoro-3-(3-methoxy-11-oxo-11*H*-benzo[4,5]thiazolo[3,2-*b*]isoquinolin-6-yl)propanoate (45)

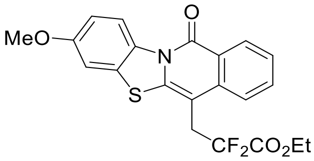

Prepared from **GPF**. Afforded the desired product **45** as a yellow solid, Mp = 195–196 °C. ^{1}H NMR (400 MHz, $CDCl_3$) δ = 9.08 (d, *J* = 9.2 Hz, 1H), 8.59–8.57 (dd, *J* = 1.6 Hz, *J* = 8.4 Hz, 1H), 8.75–8.70 (m, 1H), 7.64 (d, *J* = 8.4 Hz, 1H), 7.51–7.47 (m, 1H), 7.05 (d, *J* = 2.4 Hz, 1H), 6.97–6.94 (dd, *J* = 2.8 Hz, *J* = 9.2 Hz, 1H), 4.29–4.24 (q, *J* = 7.2 Hz, 2H), 3.86 (s, 3H), 3.68–3.59 (m, 2H), 1.25 (t, *J* = 7.2 Hz, 3H). ^{13}C NMR (100 MHz, $CDCl_3$) δ = 164.04 (t, *J* = 34.0 Hz), 161.19, 157.98, 141.95, 135.21, 132.79, 132.67, 128.36, 126.62, 125.44, 122.95, 121.53, 120.66, 116.71 (t, *J* = 120.0 Hz), 112.13, 106.35, 98.72, 63.34, 55.75, 35.23 (t, *J* = 25.0 Hz), 13.78. ^{19}F NMR (376 MHz, $CDCl_3$) δ = -102.32 (t, *J* = 17.0 Hz, 2F). HRMS (ESI) m/z calculated for $C_{21}H_{18}F_2NO_4S$ $[M+H]^+$ 418.0919, found 418.0916.

ethyl 3-(2,3-dimethoxy-11-oxo-11*H*-benzo[4,5]thiazolo[3,2-*b*]isoquinolin-6-yl)-2,2-difluoropropanoate (46)

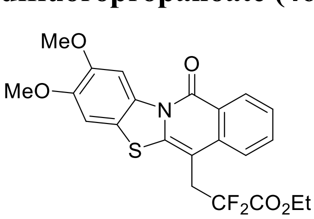

Prepared from **GPF**. Afforded the desired product **46** as a yellow solid, Mp = 260–261 °C. ^{1}H NMR (400 MHz, $CDCl_3$) δ = 8.91 (s, 1H), 8.54 (dd, *J* = 8.3, *J* =1.5 Hz, 1H), 7.75–7.67 (m, 1H), 7.60 (d, *J* = 8.5 Hz, 1H), 7.48 (dd, *J* = 8.1, *J* = 6.9 Hz, 1H), 6.95 (s, 1H), 4.26 (q, *J* = 7.2 Hz, 2H), 4.03 (s, 3H), 3.91 (s, 3H), 3.59 (t, *J* = 16.6 Hz, 2H), 1.25 (t, *J* = 7.1 Hz, 3H). ^{13}C NMR (100 MHz, $CDCl_3$) δ = 163.99 (t, *J* = 32.0 Hz), 161.37, 147.90, 147.58, 142.71, 135.10, 132.63, 132.45, 128.13, 125.27, 122.58, 121.47, 115.90, 115.37 (t, *J* = 254.0 Hz), 104.07, 103.16, 98.68, 63.30, 56.28, 56.20, 35.20 (t, *J*

= 25.0 Hz), 13.75. ^{19}F NMR (376 MHz, $CDCl_3$) δ = -102.25 (t, *J* = 17.4 Hz, 2F). HRMS (ESI) m/z calculated for $C_{22}H_{20}F_2NO_5S$ $[M+H]^+$ 448.1025, found448.1030.

ethyl 2,2-difluoro-3-(3-fluoro-11-oxo-11*H*-benzo[4,5]thiazolo[3,2-*b*]isoquinolin-6-yl)propanoate (47)

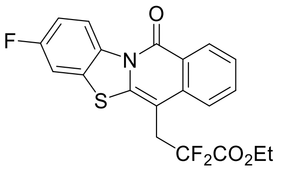

Prepared from **GPF**. Afforded the desired product **47** as a yellow solid, Mp = 234–235 °C. ^{1}H NMR (400 MHz, $CDCl_3$) δ = 9.17–9.13 (dd, *J* = 4.8 Hz, *J* = 9.2 Hz, 1H), 8.59–8.57 (m, 1H), 7.76–7.72 (m, 1H), 7.64 (d, *J* = 8.4 Hz, 1H), 7.53–7.49 (m, 1H), 7.26–7.24 (m, 1H), 7.16–7.10 (m, 1H), 4.31–4.26 (q, *J* = 7.2 Hz, 2H), 3.67–3.59 (m, 2H), 1.27 (t, *J* = 7.2 Hz, 3H). ^{13}C NMR (100 MHz, $CDCl_3$) δ = 163.95, 161.33 (t, *J* = 33.0 Hz), 159.23, 141.59, 135.30, 135.28, 135.22, 132.98, 128.45, 126.98 (d, *J* = 10.0 Hz), 125.73, 122.95, 121.61, 121.01 (d, *J* = 8.0 Hz), 117.85, 115.32, 113.59 (d, *J* = 23.0 Hz), 108.56 (d, *J* = 28.0 Hz), 99.09, 63.40, 35.15 (d, *J* = 25.0 Hz), 13.80. 13.76. ^{19}F NMR (376 MHz, $CDCl_3$) δ = -102.36 (t, *J* = 17.2 Hz, 1F), -113.75 – -113.93 (m, 2F). HRMS (ESI) m/z calculated for $C_{20}H_{14}F_3NO_3SNa$ $[M+Na]^+$ 428.0539, found 428.0542.

methyl 6-(3-ethoxy-2,2-difluoro-3-oxopropyl)-11-oxo-11*H*-benzo[4,5]thiazolo[3,2-*b*]isoquinoline-3-carboxylate (48)

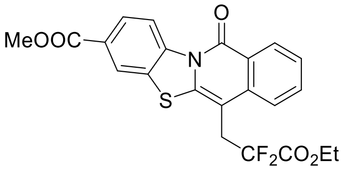

Prepared from **GPF**. Afforded the desired product **48** as a yellow solid, Mp = 75–76 °C. ^{1}H NMR (400 MHz, $CDCl_3$) δ = 9.22 (d, *J* = 8.4 Hz, 1H), 8.61–8.58 (m, 1H), 8.22 (d, *J* = 2.0 Hz, 1H), 8.13–8.11 (dd, *J* = 1.6 Hz, *J* = 8.8 Hz, 1H), 7.79–7.75 (td, *J* = 1.6 Hz, *J* = 6.8 Hz, 1H), 7.66 (d, *J* = 8.4 Hz, 1H), 7.55–7.51 (m, 1H), 4.32–4.27 (q, *J* = 7.2 Hz, 2H), 3.96 (s, 3H), 3.69–3.62 (m, 2H), 1.28 (t, *J* = 7.2 Hz, 3H). ^{13}C NMR (100 MHz, $CDCl_3$) δ = 165.40, 163.98 (t, *J* = 26.0 Hz), 161.35, 142.14, 141.72, 134.92, 133.35, 129.15, 128.28, 128.06, 125.90, 125.75, 122.97, 122.52, 121.72, 119.28, 108.15 (t, *J* = 801.0 Hz), 55.49, 35.24 (t, *J* = 25.0 Hz), 28.69, 11.19. ^{19}F NMR (376 MHz, $CDCl_3$) δ = -102.34. HRMS (ESI) m/z calculated for $C_{22}H_{17}F_2NO_5SNa$ $[M+Na]^+$468.0687, found 468.0687.

ethyl 2,2-difluoro-3-(13-oxo-13H-naphtho[1',2':4,5]thiazolo[3,2-b]isoquinolin-8-yl)propanoate (49)

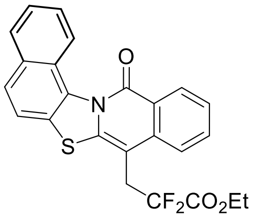

Prepared from GPF. Afforded the desired product 49 as a yellow solid, Mp = 202–203 °C. ^{1}H NMR (400 MHz, $CDCl_3$) δ = 9.31 (d, *J* = 9.2 Hz, 1H), 8.61 (dd, *J* = 8.1 Hz, *J* = 1.4 Hz, 1H), 7.95–7.86 (m, 2 H), 7.85–7.80 (m, 1H), 7.76 (m, 1H), 7.69–7.64 (m, 1H), 7.59 (m, 1H), 7.54–7.48 (m, 1H), 7.42–7.39 (m, 1H), 4.28 (q, *J* = 7.2 Hz, 2H), 3.71 (t, *J* = 16.6 Hz, 2H), 1.26 (t, J = 7.1 Hz, 3H). ^{13}C NMR (100 MHz, $CDCl_3$) δ = 164.01 (t, *J* = 32.0 Hz), 161.56, 150.43, 147.99, 146.38,

142.28, 136.53, 135.15, 134.37, 132.73, 131.34, 129.76, 128.54, 128.43, 127.11, 126.91, 126.34, 125.99, 125.32, 123.78, 122.83, 121.89, 121.49, 121.30, 118.35, 115.48 (t, J = 251.0 Hz), 99.88, 98.69, 63.34, 35.34 (t, J = 25.0 Hz), 13.81. ^{19}FNMR (376 MHz, $CDCl_3$) δ = -102.23 (t, J = 17.1 Hz, 2F). HRMS (ESI) m/z calculated for $C_{24}H_{17}F_2NO_3SNa$ $[M+Na]^+$ 460.0789, found 460.0785.

ethyl 2,2-difluoro-3-(9-methyl-11-oxo-11*H*-benzo[4,5]thiazolo[3,2-*b*]isoquinolin-6-yl)propanoate (50)

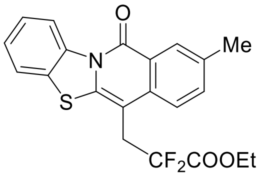

Prepared from **GPF**. Afforded the desired product **50** as a yellow solid, Mp = 226–227 °C. ^{1}H NMR (400 MHz, $CDCl_3$) δ = 9.14–9.11 (dd, J = 7.2 Hz, J = 8.4 Hz, 1H), 8.33 (s, 1H), 7.51–7.46 (m, 3H), 7.43–7.38 (m, 1H), 7.35–7.31 (m, 1H), 4.29–4.23 (q, J = 7.2 Hz, 2H), 3.56 (t, J = 16.8 Hz, 2H), 2.48 (s, 3H), 3.56 (t, J = 7.2 Hz, 3H). ^{13}C NMR (100 MHz, $CDCl_3$) δ = 163.99 (t, J = 32.0 Hz), 161.54, 140.52, 138.91, 135.47, 134.34, 133.05, 127.84, 126.24, 126.11, 125.20, 122.86, 121.47, 121.05, 119.78, 115.37, 98.60, 63.28, 35.19 (t, J = 24.0 Hz), 21.20, 13.75. ^{19}F NMR (376 MHz, $CDCl_3$) δ = -102.33 (t, J = 16.6 Hz, 2F). HRMS (ESI) m/z calculated for $C_{21}H_{18}F_2NO_3S$ $[M+H]^+$ 402.0970, found 402.0968.

ethyl 2,2-difluoro-3-(9-fluoro-11-oxo-11*H*-benzo[4,5]thiazolo[3,2-*b*]isoquinolin-6-yl)propanoate (51)

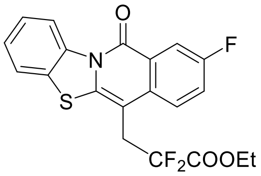

Prepared from **GPF**. Afforded the desired product **51** as a yellow solid, Mp = 192–193 °C. ^{1}H NMR (400 MHz, $CDCl_3$) δ = 9.15 (d, J = 8.2 Hz, 1H), 8.60 (dd, J = 9.2 Hz, J = 6.1 Hz, 1H), 7.56 (dd, J = 7.8 Hz, J = 1.4 Hz, 1H), 7.49–7.43 (m, 1H), 7.39 (dd, J = 8.4 Hz, J = 7.0 Hz, 1H), 7.25 (d, J = 9.0 Hz, 1H), 7.23–7.14 (m, 1H), 4.33 (q, J = 7.3 Hz, 2H), 3.59 (t, J = 16.7 Hz, 2H), 1.32 (t, J = 7.3 Hz, 3H). ^{13}C NMR (100 MHz, $CDCl_3$) δ = 167.05, 164.53 (t, J = 75.0 Hz), 160.87, 143.80, 138.81, 137.84 (d, J = 10.3 Hz), 131.83 (d, J = 10.5 Hz), 126.59, 126.42, 121.22, 119.86 (t, J = 507.0 Hz), 119.57 , 114.10, 107.07, 106.83, 98.04, 63.47, 35.42 (t, J = 25.3 Hz), 13.84. ^{19}F NMR (376 MHz, $CDCl_3$) δ = -102.35 (t, J = 17.7 Hz, 1F), -103.99 – -104.21 (m, 2F). HRMS (ESI) m/z calculated for $C_{20}H_{14}F_3NO_3SNa$ $[M+Na]^+$ 428.0539, found 428.0540.

ethyl 2,2-difluoro-3-(11-oxo-11*H*-benzo[4,5]oxazolo[3,2-*b*]isoquinolin-6-yl)propanoate (52)

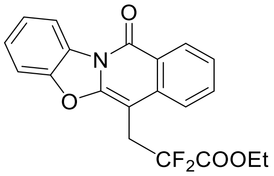

Prepared from **GPF**. Afforded the desired product **52** as a yellow solid, Mp = 228–229 °C. ^{1}H NMR (400 MHz, $CDCl_3$) δ = 8.54–8.50 (m, 2H), 7.75–7.69 (m, 2H), 7.46–7.42 (m, 1H), 7.41–7.34 (m, 3H), 4.29–4.23 (q, J = 7.2 Hz, 2H), 3.79–3.72 (m, 2H), 1.27–1.24 (m, 3H). ^{13}C NMR (100 MHz, $CDCl_3$) δ = 164.08 (t, J = 32.0 Hz), 158.67, 149.17, 146.97, 136.99, 132.93, 128.11, 127.84, 126.27, 124.51, 124.41, 122.47, 121.82, 117.55, 116.48, 115.44, 115.03, 109.94, 82.71,

77.32, 77.00, 76.68, 63.14, 29.87 (t, J = 25.0 Hz), 13.80. ^{19}F NMR (376 MHz, $CDCl_3$) δ = -103.98 (t, J = 16.6 Hz, 2F). HRMS (ESI) m/z calculated for $C_{20}H_{15}F_2NO_4Na$ [M+Na]$^+$394.0861, found 394.0856.

ethyl 2,2-difluoro-3-(5-oxo-2,3-diphenyl-5*H*-thiazolo[3,2-*b*]isoquinolin-10-yl)propanoate (53)

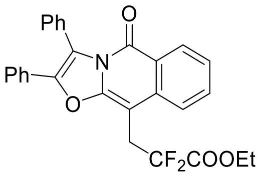

Prepared from **GPF**. Afforded the desired product **53** as a yellow solid, Mp = 240–241 °C. 1H NMR (400 MHz, $CDCl_3$) δ = 8.37 (dd, J = 8.2 Hz, J = 1.5 Hz, 1H), 7.76–7.64 (m, 2H), 7.59 (dd, J = 7.2 Hz, J = 1.9 Hz, 2H), 7.54 (d, J = 7.0 Hz, 3H), 7.47–7.39 (m, 2H), 7.37–7.27 (m, 4H), 4.22 (q, J = 7.2 Hz, 2H), 3.84 (t, J = 15.9 Hz, 2H), 1.21 (t, J = 7.1 Hz, 3H). ^{13}C NMR (100 MHz, $CDCl_3$) δ = 164.26 (t, J = 253.0 Hz), 157.93, 148.65, 141.98, 136.88, 132.54, 130.87, 129.69, 129.40, 128.64, 128.55, 128.26, 127.41, 126.57, 126.01, 123.72, 121.71, 121.30, 121.27, 117.83, 115.31, 81.31, 63.13, 30.07 (t, J = 26.0 Hz), 13.73. ^{19}F NMR (376 MHz, $CDCl_3$) δ = -103.59 (t, J = 16.5 Hz, 2F). HRMS (ESI) m/z calculated for $C_{28}H_{21}F_2NO_3Na$ [M+Na]$^+$ 512.1102, found 512.1091.

Rearrangement product derived from Ketoprofen (60)

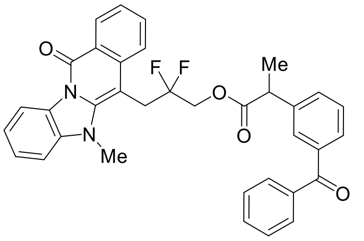

Prepared from **GPH**. Afforded the desired product **60** as a yellow solid, Mp = 68–69 °C. ^{1}H NMR (400 MHz, $CDCl_3$) δ = 8.82 (d, J = 8.0 Hz, 1H), 8.51 (d, J = 8.0 Hz, 1H), 7.82 (d, J = 1.8 Hz, 1H), 7.78–7.72 (m, 2H), 7.69 (d, J = 7.6 Hz, 1H), 7.63–7.44 (m, 4H), 7.42 (t, J = 7.6 Hz, 3H), 7.35 (t, J = 7.8 Hz, 1H), 7.26 (d, J = 2.8 Hz, 1H), 7.19 (t, J = 7.8 Hz, 1H), 7.06 (d, J = 8.0 Hz, 1H), 4.33 (dq, J = 35.6 Hz, J = 12.1 Hz, 2H), 3.93 (q, J = 7.1 Hz, 1H), 3.72 (s, 5H), 1.62 (d, J = 7.1 Hz, 3H). ^{13}C NMR (100 MHz, $CDCl_3$) δ = 196.25, 172.78, 159.63, 140.86, 140.18, 138.17, 137.15, 135.18, 132.62, 132.06, 131.32, 129.97, 129.37, 129.02, 128.76, 128.30, 127.75, 127.58, 122.40,121.15 (t, J = 421.0 Hz), 120.84, 119.04, 118.41, 106.73, 80.77, , 64.05 (t, J = 33.7 Hz), 63.71, 45.24, 32.57, 28.84 (t, J = 24.1 Hz), 18.44. ^{19}F NMR (376 MHz, $CDCl_3$) δ = -102.32, -103.51. HRMS (ESI) m/z calculated for $C_{35}H_{29}F_2N_2O_4$ [M+H]$^+$ 579.2090 found 579.2086.

Rearrangement product derived from Fernoxone (61)

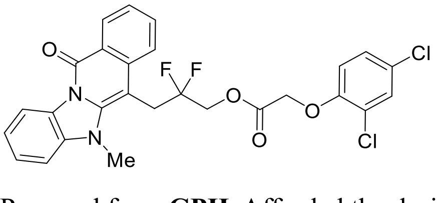

Prepared from **GPH**. Afforded the desired product **61** as a yellow solid, Mp = 87–88°C.H NMR (400 MHz, $CDCl_3$) δ = 8.84 (d, J = 8.0 Hz, 1H), 8.54 (d, J = 8.0 Hz, 1H), 7.67–7.53 (m, 2H), 7.46–7.34 (m, 2H), 7.31 (m, 1H), 7.24–7.15 (m, 2H), 7.08 (d, J = 7.8 Hz, 1H), 6.78 (d, J = 8.9 Hz, 1H), 4.74 (s, 2H), 4.38 (t, J = 12.1 Hz, 2H), 3.88 (d, J = 43.5 Hz, 5H). ^{13}C NMR (100 MHz, $CDCl_3$) δ = 167.08, 159.66, 152.08, 141.09, 138.09, 135.24, 132.24, 131.84, 130.48, 127.94, 127.73, 127.65, 127.55, 125.82, 122.59 (t, J = 164.0 Hz), 122.12, 121.34, 120.71, 119.18, 117.03, 114.76, 106.80, 106.48, 80.65, 77.32, 77.00, 76.68, 66.14, 64.14 (t, J = 39.0 Hz), 63.79, 31.59, 29.12 (t, J = 24.0 Hz). ^{19}F NMR (376 MHz, $CDCl_3$) δ =

-98.94. HRMS (ESI) m/z calculated for $C_{27}H_{20}F_2Cl_2N_2O_4Na$ $[M+Na]^+$ 567.0660, found 567.0681.

Rearrangement product derived from Estrone (62)

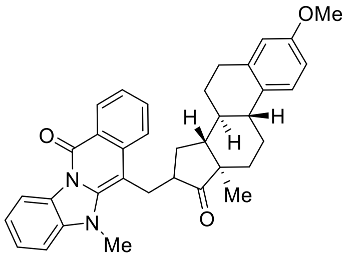

Prepared from **GPH**. Afforded the desired product **62** as a yellow solid, Mp = 194–195 °C. ^1H NMR (400 MHz, $CDCl_3$) δ = 8.88–8.85 (m, 1H), 8.59–8.57 (dd, *J* = 1.6 Hz, *J* = 8.0 Hz, 1H), 7.77 (d, *J* = 8.4 Hz, 1H), 7.69–7.65 (m, 1H), 7.40–7.33 (m, 2H), 7.24–7.19 (m, 2H), 7.14 (d, *J* = 8.0 Hz, 1H), 6.73–6.69 (dd, *J* = 2.8 Hz, *J* = 8.4 Hz, 1H), 6.61 (d, *J* = 2.8 Hz, 1H), 3.89 (s, 3H), 3.76 (s, 3H), 3.62–3.57 (dd, *J* = 3.6 Hz, *J* = 15.6 Hz, 1H), 3.17–3.12 (m, 1H), 2.91–2.79 (m, 3H), 2.43–2.39 (m, 1H), 2.31–2.25 (m, 1H), 2.04–1.99 (m, 1H), 1.84–1.73 (m, 2H), 1.65–1.59 (m, 1H), 1.57–1.48 (m, 3H), 1.37–1.25 (m, 2H), 0.92 (s, 3H). ^{13}C NMR (100 MHz, $CDCl_3$) δ = 221.15, 159.58, 157.52, 139.60, 137.70, 137.55, 135.81, 132.13, 131.67, 128.06, 126.19, 125.66, 122.50, 121.26, 121.08, 119.66, 116.98, 113.73, 111.52, 106.79, 89.37, 55.12, 48.77, 48.73, 46.08, 44.01, 37.99, 33.90, 31.82, 29.40, 26.49, 26.43, 25.72, 25.11, 14.40. HRMS (ESI) m/z calculated for $C_{36}H_{36}N_2O_3Na$ $[M+Na]^+$ 567.2618, found 567.2623.

Rearrangement product derived from Oxaprozin (63)

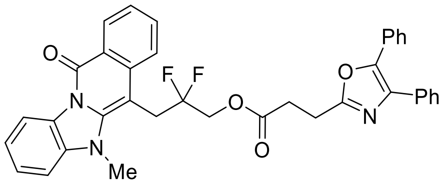

Prepared from **GPH**. Afforded the desired product **63** as a yellow solid, Mp = 121–122 °C. ^1H NMR (400 MHz, $CDCl_3$) δ = 8.81 (d, *J* = 8.0 Hz, 1H), 8.51 (d, *J* = 8.1 Hz, 1H), 7.60–7.45 (m, 6H), 7.36–7.22 (m, 5H), 7.21–7.04 (m, 4H), 6.95 (d, *J* = 8.1 Hz, 1H), 4.40 (t, *J* = 11.8 Hz, 2H), 3.73 (m, 5H), 3.25 (t, *J* = 7.1 Hz, 2H), 3.04 (t, *J* = 7.1 Hz, 2H). ^{13}C NMR (100 MHz, $CDCl_3$) δ = 170.94, 161.19, 159.58, 145.51, 140.56, 138.24, 135.07, 132.10, 131.93, 128.57, 128.52, 128.38, 128.26, 127.88, 127.71, 127.52, 127.45, 126.17, 125.56, 122.25, 121.35 (t, *J* = 248.0 Hz), 120.92, 118.20, 116.78, 106.56, 80.85, 63.99 (t, *J* = 35.0 Hz), 32.35, 30.54, 28.67 (t, *J* = 24.0 Hz), 23.18. ^{19}F NMR (376 MHz, $CDCl_3$) δ = -102.15, -103.12. HRMS (ESI) m/z calculated for $C_{37}H_{30}F_2N_3O_4$ $[M+H]^+$ 618.2199, found 618.2198.

Rearrangement product derived from Gemfibrozil (64)

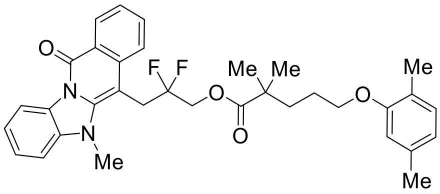

Prepared from **GPH**. Afforded the desired product **64** as a yellow solid, Mp = 58–59 °C. ^1H NMR (400 MHz, $CDCl_3$) δ = 8.82 (dd, *J* = 8.1 Hz, *J* = 1.2 Hz, 1H), 8.52 (dt, *J* = 8.1 Hz, *J* = 1.1 Hz, 1H), 7.57–7.53 (m, 2H), 7.39–7.26 (m, 2H), 7.18 (td, *J* = 7.8 Hz, *J* = 1.1 Hz, 1H), 7.04–6.94 (m, 2H), 6.70–6.56 (m, 2H), 4.31 (t, *J* = 12.4 Hz, 2H), 3.94 (t, *J* = 5.3 Hz, 2H), 3.85–3.62 (m, 5H), 2.28 (s, 3H), 2.15 (s, 3H), 1.88–1.73 (m, 4H), 1.33 (s, 6H). ^{13}C NMR (100 MHz, $CDCl_3$) δ = 176.52, 159.61, 156.77, 140.77, 138.18, 136.46, 135.11, 132.01, 130.28, 127.71, 127.54, 125.68, 123.44, 122.37,

121.22, 121.10, 120.73, 119.01, 111.86 (t, J = 519.0 Hz), 80.84, 67.68 (t, J = 32.8 Hz), 42.25, 37.06, 32.60, 28.99 (t, J = 24.6 Hz), 25.03, 21.34, 15.72. ^{19}F NMR (376 MHz, $CDCl_3$) δ = -102.28, -103.89. HRMS (ESI) m/z calculated for $C_{34}H_{36}F_2N_2O_4Na$ [M+Na]$^+$ 597.2535, found 597.2533.

Rearrangement product derived from Naproxen (65)

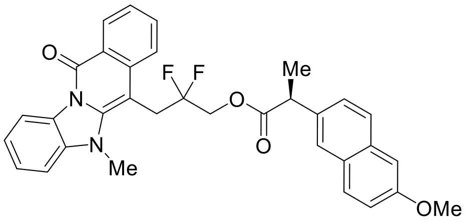

Prepared from **GPH**. Afforded the desired product **65** as a yellow solid, Mp = 72–73 °C. ^{1}H NMR (400 MHz, $CDCl_3$) δ = 8.77 (d, J = 7.9 Hz, 1H), 8.46 (d, J = 8.0 Hz, 1H), 7.78–7.69 (m, 3H), 7.42 (d, J = 8.4 Hz, 1H), 7.33 (d, J = 3.9 Hz, 2H), 7.28 (d, J = 7.8 Hz, 1H), 7.23–7.15 (m, 2H), 7.12 (dd, J = 9.6 Hz, J = 5.2 Hz, 2H), 6.79 (d, J = 8.0 Hz, 1H), 4.43 (q, J = 12.9 Hz, J = 12.1 Hz, 1H), 4.33–4.19 (m, 1H), 3.98 (q, J = 7.1 Hz, 1H), 3.88 (s, 3H), 3.52 (s, 2H), 3.35 (s, 3H), 1.65 (d, J = 7.1 Hz, 3H). ^{13}C NMR (100 MHz, $CDCl_3$) δ = 173.36, 159.58, 157.84, 140.69, 138.19, 135.08, 134.88, 133.82, 131.91, 131.35, 130.87, 130.20, 129.19, 128.91, 127.57, 127.49, 126.10, 125.85, 125.54, 121.22, 120.98, 119.36 (t, J = 252.0 Hz), 118.92, 106.49, 105.57, 80.80, 77.32, 77.00, 76.68, 63.96 (t, J = 35.0 Hz), 55.28, 45.33, 32.13 (t, J = 3.0 Hz), 28.64, 18.54. ^{19}F NMR (376 MHz, $CDCl_3$) δ = -103.32. HRMS (ESI) m/z calculated for $C_{33}H_{28}F_2N_2O_4Na$ [M+Na]$^+$ 577.1909, found 577.1914.

Rearrangement product derived from Nortropine (66)

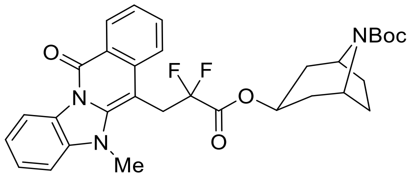

Prepared from **GPH**. Afforded the desired product **66** as a yellow solid, Mp = 262–263 °C. ^{1}H NMR (400 MHz, $CDCl_3$) δ = 8.71 (d, J = 8.0 Hz, 1H), 8.43 (d, J = 8.1 Hz, 1H), 7.44 (dd, J = 26.4 Hz, J = 8.0 Hz, 2H), 7.25 (td, J = 16.0 Hz, J = 14.2 Hz, J = 6.1 Hz, 2H), 7.11 (t, J = 7.7 Hz, 1H), 6.96 (d, J = 8.1 Hz, 1H), 5.06 (t, J = 4.8 Hz, 1H), 4.25–3.98 (m, 2H), 3.75 (m, 5H), 2.31–1.90 (m, 3H), 1.89–1.77 (m, 2H), 1.69 (t, J = 7.3 Hz, 2H), 1.46 (m, 10H). ^{13}C NMR (100 MHz, $CDCl_3$) δ = 163.51 (t, J = 30.0 Hz), 159.23, 152.99, 140.47, 137.73, 134.73, 131.83, 127.28, 127.12, 125.58, 122.24, 121.15, 120.93, 118.58, 116.51, 114.47 (t, J = 213.0 Hz), 106.66, 79.56, 79.38, 71.58, 66.07, 60.24, 52.09, 51.30, 35.00, 34.37, 32.44, 29.47 (t, J = 25.0 Hz), 28.35, 28.28, 27.79, 27.11. ^{19}F NMR (376 MHz, $CDCl_3$) δ = -103.01, -105.19. HRMS (ESI) m/z calculated for $C_{31}H_{33}F_2N_2O_4Na$ [M+Na]$^+$ 588.2280, found 588.2285.

Rearrangement product derived from *L*-menthol (67)

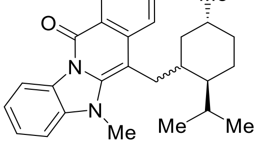

Prepared from **GPH**. Afforded the desired product **67** as a yellow solid (dr = 2:1), Mp = 119–120 °C. ^{1}H NMR (400 MHz, $CDCl_3$) δ = 8.88 (d, J = 7.9 Hz, 2H), 8.84 (d, J = 7.9 Hz, 1H), 8.61–8.54 (m, 3H), 7.72 (s, 1H), 7.67–7.61 (m, 4H), 7.35 (qd, J = 7.9, J = 3.1 Hz, 7H), 7.23–7.17 (m, 3H), 7.12 (d, J = 7.8 Hz, 1H), 7.09 (d, J = 8.1 Hz, 2H), 3.84 (s, 3H),

3.83 (s, 6H), 3.35 (dd, J = 15.3, J = 4.9 Hz, 2H), 3.15 (dd, J = 7.7, J = 2.9 Hz, 2H), 2.82 (dd, J = 15.3, J = 10.8 Hz, 2H), 2.46–2.36 (m, 1H), 2.33–2.26 (m, 2H), 1.85–1.76 (m, 2H), 1.69 (dtd, J = 8.9 Hz, J = 6.4 Hz, J = 3.3 Hz, 7H), 1.61–1.49 (m, 2H), 1.36–1.17 (m, 7H), 1.03 (d, J = 6.8 Hz, 9H), 0.97–0.91 (m, 9H), 0.87 (dd, J = 8.2 Hz, J = 4.5 Hz, 9H), 0.62 (d, J = 6.4 Hz, 6H), 0.55 (d, J = 6.3 Hz, 3H). ^{13}C NMR (100 MHz, $CDCl_3$) δ = 159.69, 138.25, 136.86, 136.01, 131.65, 131.54, 128.13, 127.82, 125.64, 125.54, 122.56, 122.43, 122.27, 122.10, 121.13, 120.75, 119.61, 116.98, 116.87, 107.27, 106.55, 76.68, 49.45, 48.36, 40.44, 35.88, 35.71, 35.26, 34.44, 32.67, 29.29, 28.01, 27.04, 26.55, 24.97, 24.37, 22.59, 21.96, 21.81, 21.60, 16.10. HRMS (ESI) m/z calculated for $C_{27}H_{33}N_2O$ $[M+H]^+$ 401.2587 found 401.2585.

Rearrangement product derived from Indometacin (68)

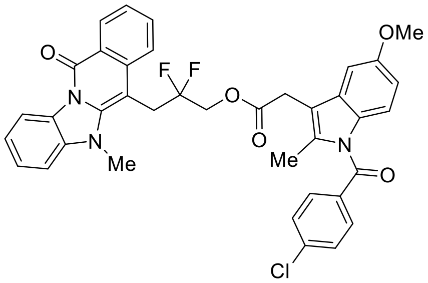

Prepared from **GPH**. Afforded the desired product **68** as a yellow solid, Mp = 101–102 °C. ^{1}H NMR (400 MHz, $CDCl_3$) δ = 8.81 (dd, J = 8.0 Hz, J =1.1 Hz, 1H), 8.50 (dd, J = 8.1 Hz, J =1.5 Hz, 1H), 7.64–7.58 (m, 2H), 7.47–7.31 (m, 5H), 7.26 (m, 1H), 7.18 (td, J = 7.8 Hz, J =1.1 Hz, 1H), 7.04 (d, J = 8.0 Hz, 1H), 6.98 (d, J = 2.5 Hz, 1H), 6.87 (d, J = 9.0 Hz, 1H), 6.68 (dd, J = 9.1 Hz, J =2.6 Hz, 1H), 4.32 (t, J = 12.3 Hz, 2H), 3.80–3.77 (m, 5H), 3.71 (s, 5H), 2.41 (s, 3H). ^{13}C NMR (100 MHz, $CDCl_3$) δ = 169.59, 168.22, 159.56, 156.09, 140.85, 139.39, 138.07, 136.10, 135.13, 133.52, 132.07, 131.13, 130.70, 129.83, 129.09, 128.24, 126.99, 124.53, 122.41, 121.16, 120.99, 120.87, 116.52 (t, J = 249.0 Hz), 111.80, 110.43, 105.71, 100.10, 80.71, 64.02 (t, J = 33.0 Hz), 55.61, 32.53, 29.96, 28.91 (t, J = 35.0 Hz), 13.29. ^{19}F NMR (376 MHz, $CDCl_3$) δ = -102.10, -103.45. HRMS (ESI) m/z calculated for $C_{38}H_{30}ClF_2N_2O_5Na$ $[M+Na]^+$ 704.1734, found 704.1737.

Rearrangement product derived from Cholesterol (69)

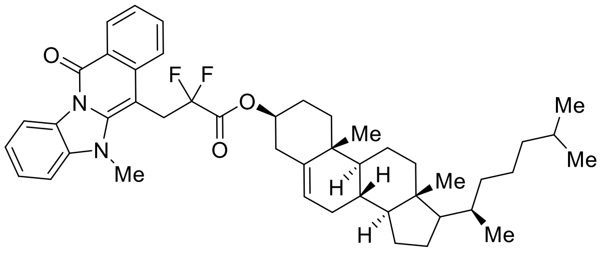

Prepared from **GPH**. Afforded the desired product **69** as a yellow solid, Mp = 137–138 °C. ^{1}H NMR (400 MHz, $CDCl_3$) δ = 8.81 (d, J = 8.0 Hz, 1H), 8.51 (d, J = 8.0 Hz, 1H), 7.60 (d, J = 3.9 Hz, 2H), 7.39–7.26 (m, 2H), 7.19 (t, J = 7.7 Hz, 1H), 7.07 (d, J = 8.1 Hz, 1H), 5.38–5.22 (m, 1H), 4.57 (dq, J = 11.5 Hz, J = 6.3 Hz, J = 5.6 Hz, 1H), 3.86 (m, 5H), 2.23–2.14 (m, 1H), 2.05–1.90 (m, 3H), 1.80 (ddt, J = 12.9 Hz, J = 7.8 Hz, J = 4.2 Hz, 3H), 1.53 (ddd, J = 18.5 Hz, J = 11.2 Hz, J = 4.4 Hz, 4H), 1.44 (dd, J = 11.1 Hz, J = 3.7 Hz, 2H), 1.37–1.30 (m, 4H), 1.26 (dd, J = 8.9 Hz, J = 4.2 Hz, 1H), 1.16–1.05 (m, 6H), 1.04–0.96 (m, 4H), 0.95–0.89 (m, 6H), 0.87 (dd, J = 6.6 Hz, J = 1.9 Hz, 6H), 0.66 (d, J = 4.5 Hz, 3H). ^{13}C NMR (100 MHz, $CDCl_3$) δ = 163.81 (t, J = 35.0 Hz), 159.62, 140.92, 138.61, 138.11, 135.15, 132.14, 127.68, 127.56, 125.74, 123.37, 122.50, 121.81, 121.43, 118.98 (t, J = 223.0 Hz), 115.62, 109.72, 83.87, 71.02, 56.68, 56.56, 54.42, 50.04, 49.82, 42.22, 39.70, 39.59, 39.45, 37.27, 36.63, 36.37, 36.11, 35.73, 32.79, 31.79, 31.68, 30.25 (t, J = 25.0

Hz), 28.59, 27.97, 27.11, 24.20, 23.77, 22.79, 22.53, 20.92, 19.34, 19.14, 18.66, 11.78. ^{19}F NMR (376 MHz, $CDCl_3$) δ = -126.37, -126.51. HRMS (ESI) m/z calculated for $C_{46}H_{59}F_2N_2O_3$ $[M+H]^+$ 725.4488 found 725.4485.

Rearrangement product derived from Ibuprofen (70)

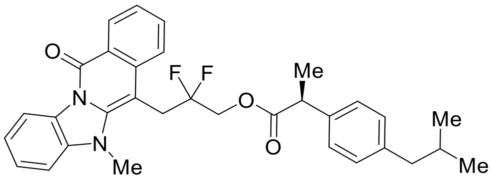

Prepared from **GPH**. Afforded the desired product **70** as a yellow solid, Mp = 77–78 °C. ^{1}H NMR (400 MHz, $CDCl_3$) δ = 8.84 (dd, *J* = 8.0 Hz, *J* = 1.2 Hz, 1H), 8.52 (dt, *J* = 8.1 Hz, *J* = 1.1 Hz, 1H), 7.51–7.42 (m, 2H), 7.35 (td, *J* = 7.8 Hz, *J* = 1.2 Hz, 1H), 7.31–7.24 (m, 3H), 7.22–7.12 (m, 3H), 7.05 (d, *J* = 7.9 Hz, 1H), 4.31 (dq, *J* = 43.7 Hz, *J* = 11.9 Hz, 2H), 3.88–3.77 (m, 1H), 3.68 (s, 5H), 2.44 (d, *J* = 7.2 Hz, 2H), 1.81 (m, 1H), 1.57 (d, *J* = 7.2 Hz, 3H), 0.85 (d, *J* = 2.8 Hz, 3H), 0.84 (d, *J* = 2.8 Hz, 3H).^{13}C NMR (100 MHz, , $CDCl_3$) δ = 173.46, 159.70, 141.05, 140.89, 138.24, 137.02, 135.23, 132.07, 129.60, 127.74, 127.66, 127.15, 122.40, 121.30, 121.15, 120.98 (t, *J* = 245.0 Hz), 119.07, 117.00, 106.61, 80.93, 63.84 (t, *J* = 34.5 Hz), 45.01, 44.94, 32.52, 30.14, 28.68 (t, *J* = 24.7 Hz), 22.30, 22.26, 18.52. ^{19}F NMR (376 MHz, $CDCl_3$) δ = -102.51. HRMS (ESI) m/z calculated for $C_{32}H_{33}F_2N_2O_3$ $[M+H]^+$ 531.2454 found 531.2458.

Rearrangement product derived from Flurbiprofen (71)

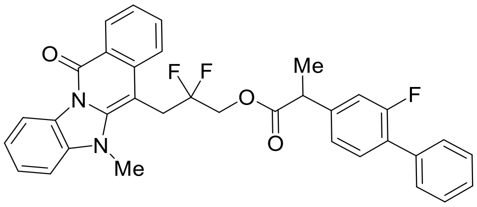

Prepared from **GPH**. Afforded the desired product **71** as a yellow solid, Mp = 74–75 °C. ^{1}H NMR (400 MHz, $CDCl_3$) δ = 8.78 (dd, *J* = 8.0 Hz, *J* = 1.2 Hz, 1H), 8.48 (dd, *J* = 8.1 Hz, *J* = 1.5 Hz, 1H), 7.51 (dt, *J* = 8.1 Hz, *J* = 1.6 Hz, 2H), 7.47–7.33 (m, 6H), 7.30–7.26 (m, 1H), 7.23–7.10 (m, 4H), 6.95 (d, *J* = 8.0 Hz, 1H), 4.44–4.21 (m, 2H), 3.87 (q, *J* = 7.2 Hz, 1H), 3.66 (m, 5H), 1.60 (d, *J* = 7.2 Hz, 3H). ^{13}C NMR (100 Hz, $CDCl_3$) δ = 172.68, 160.94, 159.57, 158.46, 140.77, 138.12, 135.09, 132.00, 131.06 (d, *J* = 3.7 Hz), 128.85 (d, *J* = 2.5 Hz), 128.47, 128.27, 128.13, 127.80, 127.67, 127.52, 125.64, 123.51 (d, *J* = 2.9 Hz), 122.35, 118.98 (t, *J* =211.0 Hz), 115.40, 115.16, 106.65, 80.74, 64.00 (t, *J* = 33.8 Hz), 44.83, 32.46, 28.83 (t, *J* = 24.8 Hz), 18.35. ^{19}F NMR (376 MHz, $CDCl_3$) δ = -57.48 (s, 1F), -116.81 (t, *J* = 11.1 Hz, 2F). HRMS (ESI) m/z calculated for $C_{34}H_{28}F_3N_2O_3$ $[M+H]^+$ 569.2047, found 569.2049.

Rearrangement product derived from *D*-mannofuranose (72)

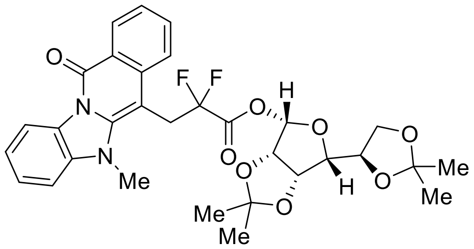

Prepared from **GPH**. Afforded the desired product **72** as a yellow solid, Mp = 87–88 °C. ^{1}H NMR (400 MHz, $CDCl_3$) δ = 8.85 (dd, *J* = 7.9 Hz, *J* =1.2 Hz, 1H), 8.58 (dd, *J* = 8.1 Hz, *J* =1.5 Hz, 1H), 7.78–7.53 (m, 2H), 7.48–7.33 (m, 2H), 7.22 (td, *J* = 7.7 Hz, *J* =1.1 Hz, 1H), 7.12 (d, *J* = 8.0 Hz, 1H), 4.84 (dd, *J* = 6.0 Hz, *J* =3.7 Hz, 1H), 4.63 (dd, *J* = 6.0 Hz, *J* =1.4 Hz, 1H), 4.41–4.31 (m, 2H), 4.06–3.97 (m, 2H), 3.93 (dd, *J* = 8.7 Hz, *J* =4.4 Hz, 1H), 3.89 (s, 3H), 3.32 (d, *J* = 7.0

Hz, 2H), 1.43 (d, *J* = 4.5 Hz, 6H), 1.36 (s, 3H), 1.27 (s, 3H). ^{13}C NMR (100 MHz, $CDCl_3$) δ = 159.64, 140.11, 137.81, 135.74, 132.26, 128.19, 128.05, 125.74, 122.55, 121.26, 120.95, 119.68, 117.03, 113.04, 109.14, 106.85, 86.64, 85.26, 84.53, 80.86, 80.79, 73.34, 66.62, 44.00, 33.73, 26.95, 26.22, 26.14, 25.09, 24.82. ^{19}F NMR (376 MHz, $CDCl_3$) δ = -99.04 (d, *J* = 268.6 Hz, 1F), -103.84 (d, *J* = 263.7 Hz, 1F). HRMS (ESI) m/z calculated for $C_{31}H_{32}F_2N_2O_8Na$ $[M+Na]^+$ 621.2019, found 621.2003.

Rearrangement product derived from Adapalene (73)

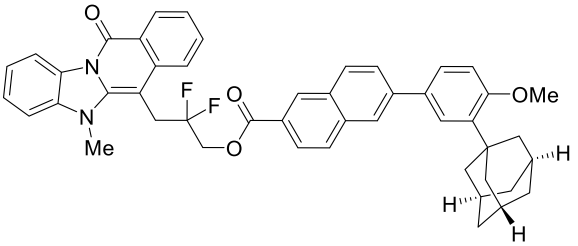

Prepared from **GPH**. Afforded the desired product **73** as a yellow solid, Mp = 218–219 °C. ^{1}H NMR (400 MHz, $CDCl_3$) δ = 8.81 (d, *J* = 7.8 Hz, 1H), 8.61 (d, *J* = 1.6 Hz, 1H), 8.52 (dd, *J* = 8.3 Hz, *J* = 1.6 Hz, 1H), 8.07–7.93 (m, 4H), 7.84 (dd, *J* = 8.6 Hz, *J* = 1.8 Hz, 1H), 7.69–7.61 (m, 2H), 7.59–7.49 (m, 2H), 7.36–7.25 (m, 2H), 7.15 (t, *J* = 7.6 Hz, 1H), 6.99 (t, *J* = 7.9 Hz, 2H), 4.60 (t, *J* = 11.9 Hz, 2H), 3.86 (d, *J* = 34.5 Hz, 8H), 2.20 (d, *J* = 3.0 Hz, 6H), 2.16–2.06 (m, 3H), 1.81 (t, *J* = 3.1 Hz, 6H). ^{13}C NMR (100 MHz, $CDCl_3$) δ = 165.45, 159.67, 159.01, 141.88, 140.88, 139.02, 138.24, 136.22, 135.17, 132.22, 132.09, 131.29, 131.10, 129.77, 128.55, 127.79, 127.61, 126.78, 125.94, 125.74, 125.66, 125.43, 125.20, 124.70, 122.49, 121.36, 121.16, 119.14, 116.96, 112.07, 106.69, 81.03, 64.10 (t, *J* = 39.0 Hz), 55.13, 40.54, 37.17, 37.06, 32.74, 29.04. ^{19}F NMR (376 MHz, $CDCl_3$) δ = -101.53. HRMS (ESI) m/z calculated for $C_{47}H_{43}F_2N_2O_4$ $[M+H]^+$ 737.3185, found737.3181.

ethyl 3-(9-bromo-5-methyl-11-oxo-5,11-dihydrobenzo[4,5]imidazo[1,2-*b*]isoquinolin-6-yl)-2,2-difluoropropanoate (74)

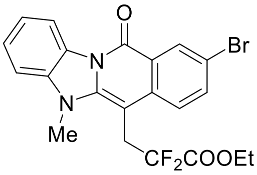

Yellow solid, Mp = 208–209 °C. ^{1}H NMR (400 MHz, $CDCl_3$) δ = 8.79 (d, *J* = 8.0 Hz, 1H), 8.59 (d, *J* = 2.3 Hz, 1H), 7.58 (dd, *J* = 9.0 Hz, *J* = 2.3 Hz, 1H), 7.49 (d, *J* = 8.9 Hz, 1H), 7.45 – 7.39 (m, 1H), 7.22 (d, *J* = 7.7 Hz, 1H), 7.12 (d, *J* = 8.0 Hz, 1H), 4.24 (q, *J* = 7.1 Hz, 2H), 3.89 (s, 5H), 1.24 (t, *J* = 7.1 Hz, 3H). ^{13}C NMR (100 MHz, $CDCl_3$) δ = 163.52(t, *J* = 18.0 Hz), 158.44, 156.94, 155.97, 150.38, 141.14, 136.82, 135.06, 134.95, 129.85, 127.43, 126.10, 124.28, 121.55 (t, *J* = 138.0 Hz), 117.06, 115.69, 115.12, 107.03, 99.89, 79.85, 63.37, 32.82, 29.98 (t, *J* = 25.0 Hz), 13.80. ^{19}F NMR (376 MHz, $CDCl_3$) δ = -103.63, -104.26. HRMS (ESI) m/z calculated for $C_{21}H_{18}F_2BrN_2O_3$ $[M+H]^+$ 463.0463, found 463.0464.

ethyl 2,2-difluoro-4-(2-(3-methyl-2-oxo-2,3-dihydro-1H-benzo[d]imidazole-1-carbonyl)phenyl)-4-oxobutanoate(75)

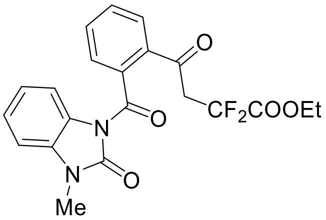

White solid. Mp = 224–225 °C. ^{1}H NMR (400 MHz, $CDCl_3$) δ = 8.31 (d, *J* = 7.8 Hz, 1H), 7.83 (d, *J* = 7.6 Hz, 1H), 7.76–7.69 (m, 1H), 7.66–7.57 (m, 1H), 7.47 (d, *J* = 7.4 Hz, 1H), 7.28 (d, *J* = 7.4 Hz, 1H), 7.25–7.16 (m, 1H), 6.97 (dd, *J* = 7.9 Hz, *J* = 1.3 Hz, 1H), 4.11 (q, *J* = 7.1 Hz, 2H), 3.93 (t, *J* = 13.1 Hz, 2H), 3.26 (s, 3H), 1.13 (t, *J* = 7.3 Hz, 3H). ^{13}C NMR (100 MHz, $CDCl_3$) δ = 193.10, 167.98, 162.54 (t, *J* = 35.0 Hz), 158.20, 151.57, 136.42, 134.09, 133.53, 130.64, 129.78, 127.96, 127.88, 126.13, 124.21, 122.71, 116.44, 114.89, 113.95 (t, *J* = 250.0 Hz), 107.36, 65.90, 44.02, 43.53 (t, *J* = 24.0 Hz), 28.95, 10.25. ^{19}F NMR (376 MHz, $CDCl_3$) δ = -104.33. HRMS (ESI) m/z calculated for $C_{21}H_{19}F_2N_2O_5$ $[M+H]^+$ 417.1257, found 417.1257.

ethyl 3-(5a,6-diazido-5-methyl-11-oxo-5,5a,6,11-tetrahydrobenzo[4,5]imidazo[1,2-b]isoquinolin-6-yl)-2,2-difluoropropanoate(76)

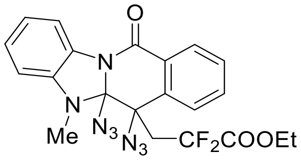

Colorless liquid. ^{1}H NMR (400 MHz, $CDCl_3$) δ = 7.96 (dt, *J* = 7.6 Hz, *J* = 1.0 Hz, 1H), 7.82 – 7.70 (m, 3H), 7.61 (td, *J* = 7.4 Hz, *J* = 1.1 Hz, 1H), 7.32–7.25 (m, 3H), 4.25 (p, *J* = 7.1 Hz, 2H), 3.85 (ddd, *J* = 20.4 Hz, *J* = 15.4 Hz, *J* = 9.6 Hz, 1H), 3.66 (s, 3H), 3.48 (ddd, *J* = 16.5 Hz, *J* = 15.3 Hz, *J* = 9.8 Hz, 1H), 1.32 (t, *J* = 7.2 Hz, 3H). ^{13}C NMR (100 MHz, $CDCl_3$) δ = 168.42, 163.32 (t, *J* = 32.0 Hz), 148.97, 148.86, 141.49 136.67, 135.05, 130.40, 124.51, 124.17, 123.60, 122.63, 120.01, 113.84 (t, *J* = 250.0 Hz), 109.57, 99.85, 82.93, 82.90, 82.87, 82.83, 63.42, 43.25 (t, *J* = 25.0 Hz), 31.39, 26.85, 13.73. ^{19}F NMR (376 MHz, $CDCl_3$) δ = -102.17 (d, *J* = 991.7 Hz, 2F). HRMS (ESI) m/z calculated for $C_{21}H_{19}F_2N_8O_3$ $[M+H]^+$ 469.1527, found 469.1538.

6-(2,2-difluoro-3-hydroxypropyl)-5-methylbenzo[4,5]imidazo[1,2-*b*]isoquinolin-11(5*H*)-one (77)

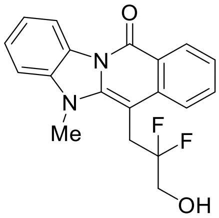

Pale yellow solid. Mp = 225–226 °C. ^{1}H NMR (400 MHz, DMSO-d_6) δ = 8.74 (d, *J* = 7.9 Hz, 1H), 8.37 (d, *J* = 8.0 Hz, 1H), 7.89 (t, *J* = 7.8 Hz, 1H), 7.69 (t, *J* = 7.6 Hz, 1H), 7.54 (d, *J* = 8.1 Hz, 1H), 7.47 (d, *J* = 7.8 Hz, 1H), 7.40–7.30 (m, 1H), 7.24 (t, *J* = 7.7 Hz, 1H), 5.85 (s, 1H), 3.95 (s, 5H), 3.80 (t, *J* = 13.0 Hz, 2H). ^{13}C NMR (100 MHz, DMSO-d_6) δ = 158.76, 140.58, 138.78, 135.46, 131.97, 126.99, 126.92, 125.98, 123.95, 122.15, 120.70 (t, *J* = 254.0 Hz), 115.97, 107.98, 82.13, 62.80(t, *J* = 30.0 Hz), 32.67, 27.32 (t, *J* = 24.0 Hz). ^{19}F NMR (376 MHz, DMSO-d_6) δ = -96.12 (d, *J* = 308.3 Hz, 1F), -103.82 (d, *J* = 273.1 Hz, 1F). HRMS (ESI) m/z calculated for $C_{19}H_{17}F_2N_2O_2$ $[M+H]^+$ 343.1253, found 343.1253.

(1-methyl-1H-benzo[d]imidazol-2-yl)(2-(vinyl-2,2-d2)phenyl)methanone (D-1)

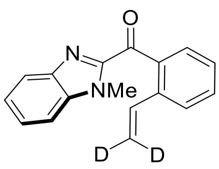

Pale yellow solid. ^{1}H NMR (400 MHz, $CDCl_3$) δ = 7.89 (dt, *J* = 8.2 Hz, *J* = 1.0 Hz, 1H), 7.81 (dd, *J* = 7.7 Hz, *J* = 1.5 Hz, 1H), 7.66 (d, *J* = 7.9 Hz, 1H), 7.54 (td, *J* = 7.8 Hz, *J* = 1.8 Hz, 1H), 7.50–7.46 (m, 2H), 7.43–7.31 (m, 3H), 7.06–6.97 (m, 1H), 5.68 (dd, *J* = 17.3 Hz, *J* = 1.6 Hz, 0.22H), 5.29 (d, *J* = 11.0 Hz, 0.18H), 4.21 (s, 3H).

ethyl 2,2-difluoro-3-(5-methyl-11-oxo-5,11-dihydrobenzo[4,5]imidazo[1,2-*b*]isoquinolin-6-yl)propanoate-3,3-d2 (D-2)

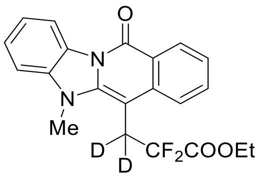

Pale yellow solid. ^{1}H NMR (400 MHz, $CDCl_3$) δ = 8.84 (d, *J* = 8.0 Hz, 1H), 8.65–8.43 (m, 1H), 7.62 (d, *J* = 4.0 Hz, 2H), 7.37 (t, *J* = 7.7 Hz, 1H), 7.31 (dt, *J* = 8.1 Hz, *J* = 3.9 Hz, 1H), 7.21 (t, *J* = 7.8 Hz, 1H), 7.10 (d, *J* = 8.0 Hz, 1H), 4.17 (q, *J* = 7.2 Hz, 2H), 3.88 (s, 3.44H), 1.17 (t, *J* = 7.2 Hz, 3H).

3.4 NMR Spectra

3.4.1 Copies of ^{1}H and ^{13}C NMR spectra

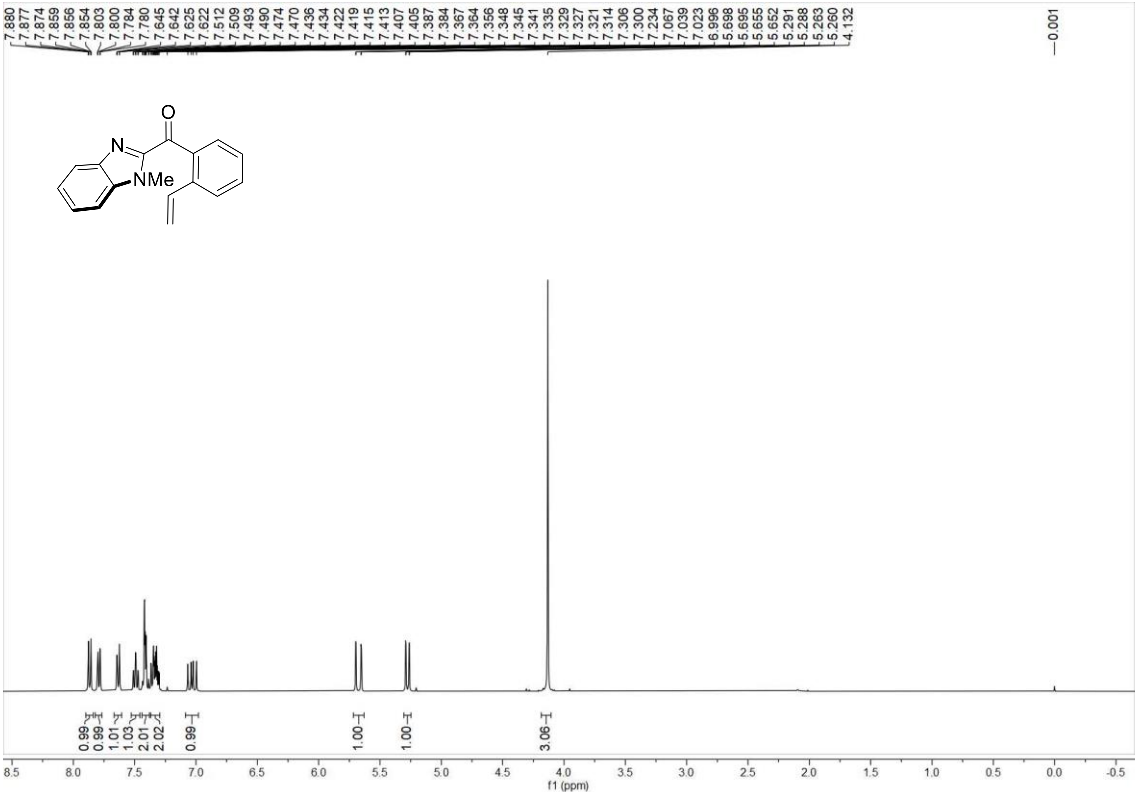

Supplementary Figure 4. ^{1}H-NMR of compound **(1-methyl-1*H*-benzo[*d*]imidazol-2-yl)(2-vinylphenyl)methanone**, recorded at 400 MHz and 25 °C in $CDCl_3$.

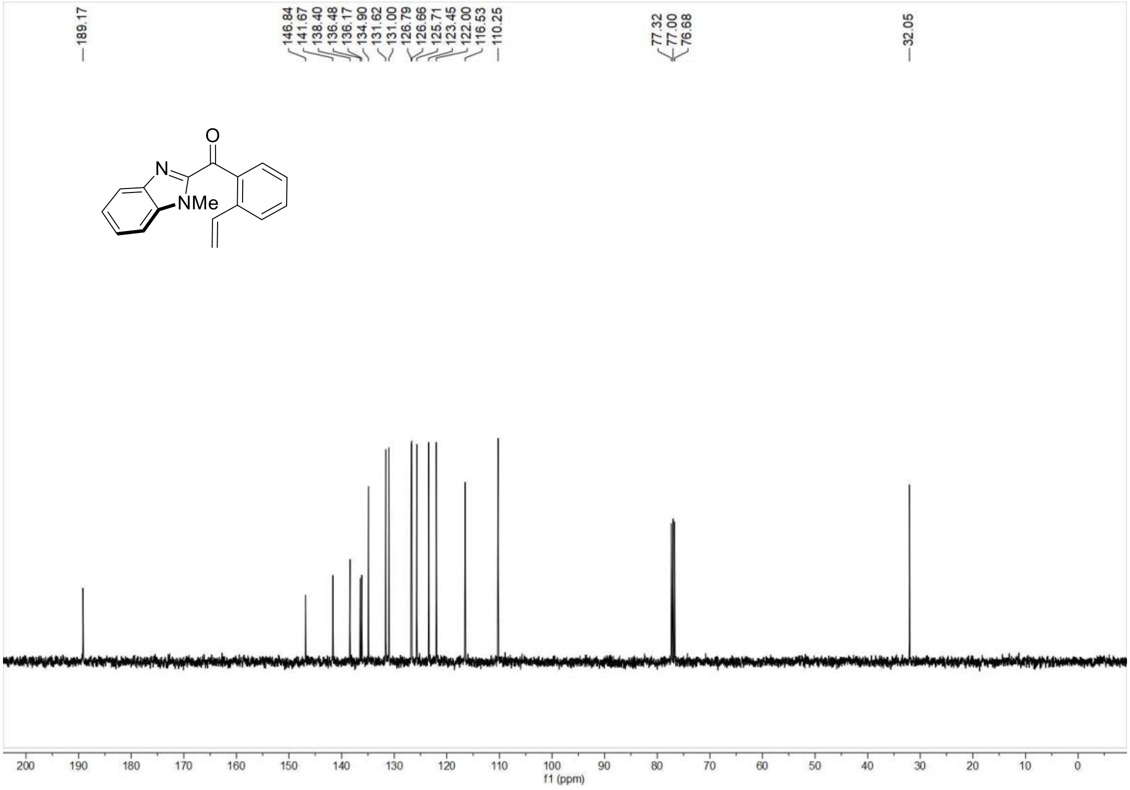

Supplementary Figure 5. ^{13}C-NMR of compound **(1-methyl-1*H*-benzo[*d*]imidazol-2-yl)(2-vinylphenyl)methanone**, recorded at 100 MHz and 25 °C in $CDCl_3$.

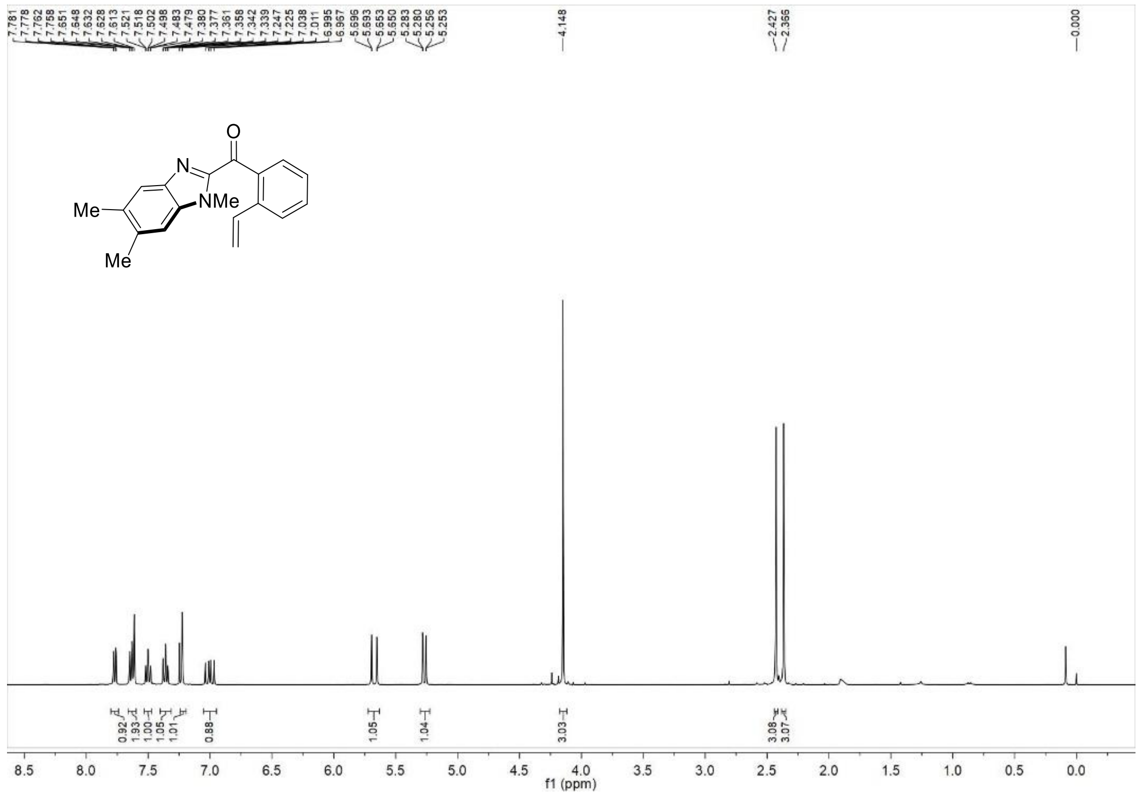

Supplementary Figure 6. ^{1}H-NMR of compound **(1,5,6-trimethyl-1*H*-benzo[*d*]imidazol-2-yl)(2-vinylphenyl)methanone**, recorded at 400 MHz and 25 °C in $CDCl_3$.

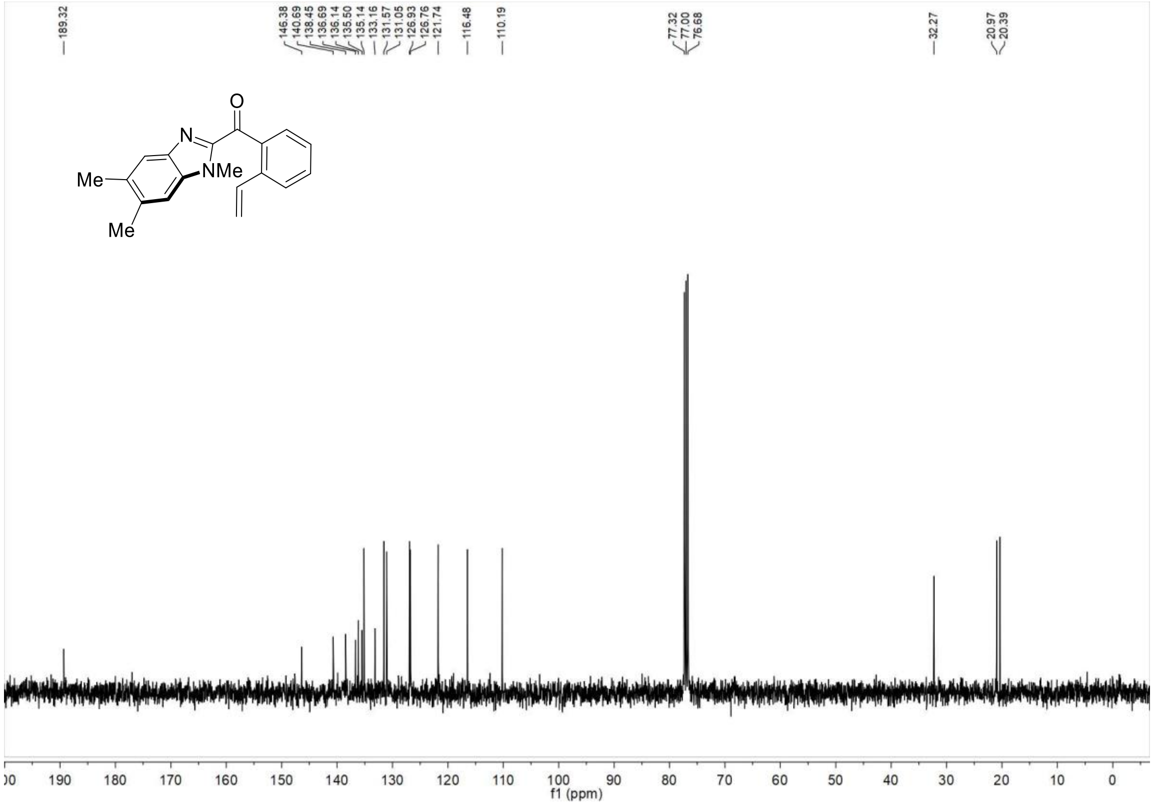

Supplementary Figure 7. ^{13}C-NMR of compound **(1,5,6-trimethyl-1*H*-benzo[*d*]imidazol-2-yl)(2-vinylphenyl)methanone**, recorded at 100 MHz and 25 °C in $CDCl_3$.

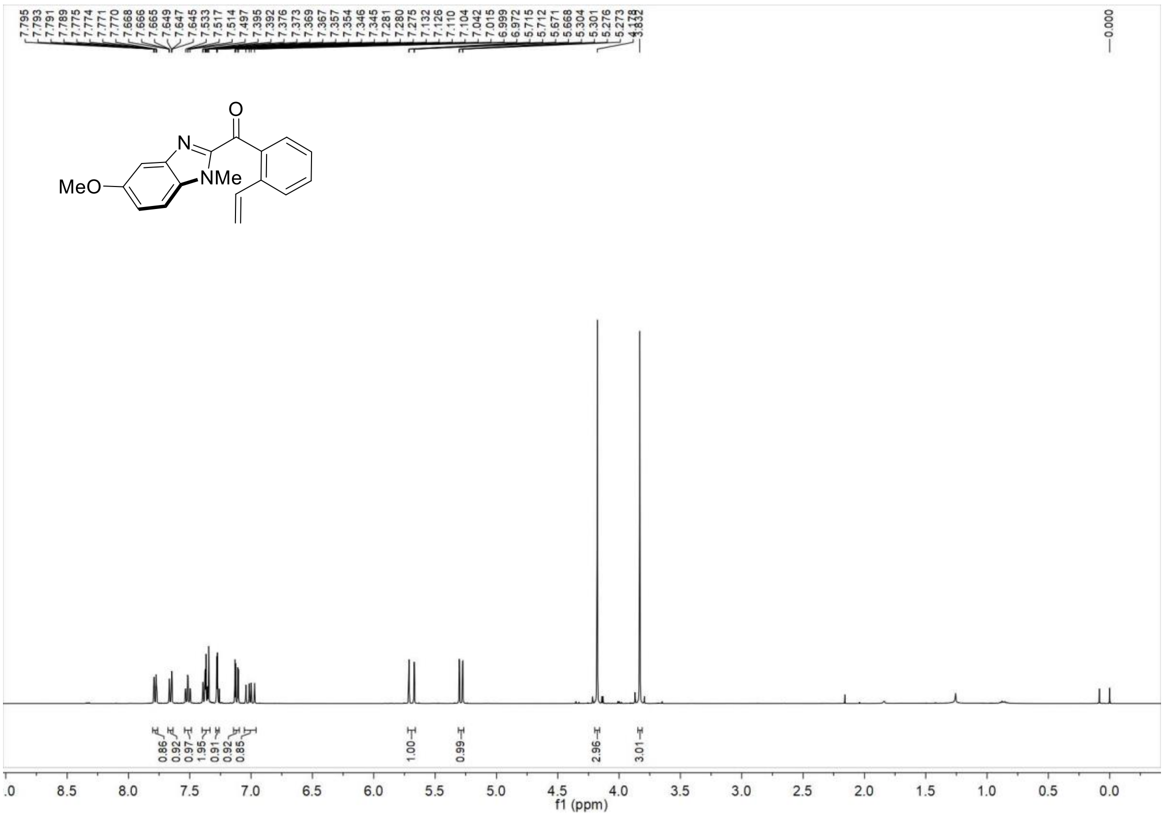

Supplementary Figure 8. ^{1}H-NMR of compound **(5-methoxy-1-methyl-1*H*-benzo[*d*]imidazol-2-yl)(2-vinylphenyl)methanone**, recorded at 400 MHz and 25 °C in $CDCl_3$.

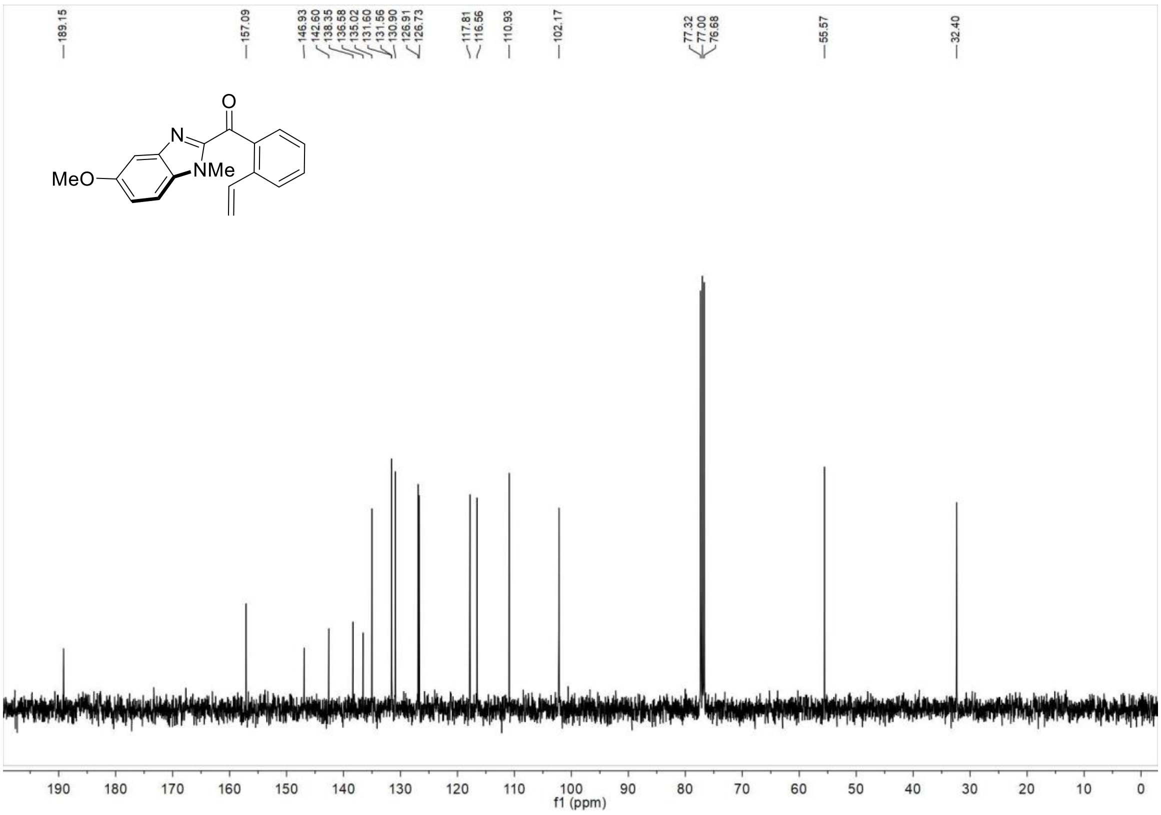

Supplementary Figure 9. ^{13}C-NMR of compound **(5-methoxy-1-methyl-1*H*-benzo[*d*]imidazol-2-yl)(2-vinylphenyl)methanone**, recorded at 100 MHz and 25 °C in $CDCl_3$.

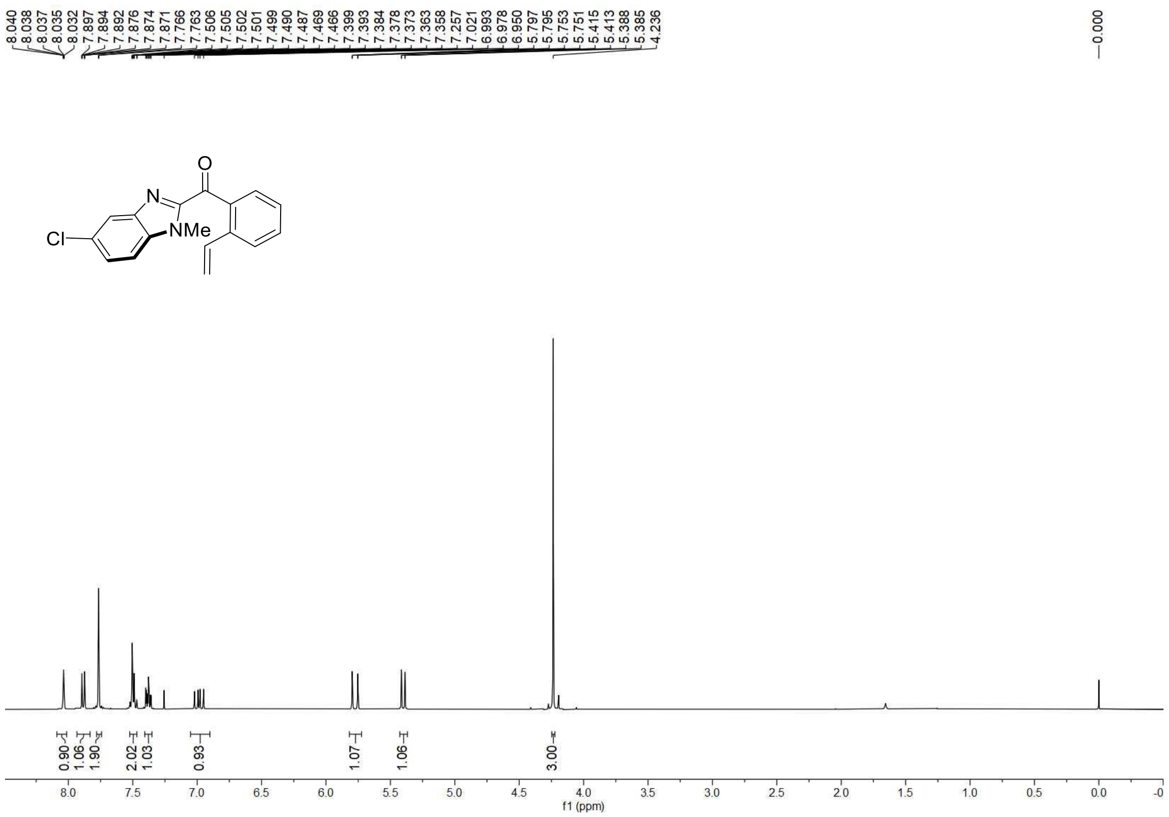

Supplementary Figure 10. ^{1}H-NMR of compound **(5-chloro-1-methyl-1*H*-benzo[*d*]imidazol-2-yl)(2-vinylphenyl)methanone**, recorded at 400 MHz and 25 °C in $CDCl_3$.

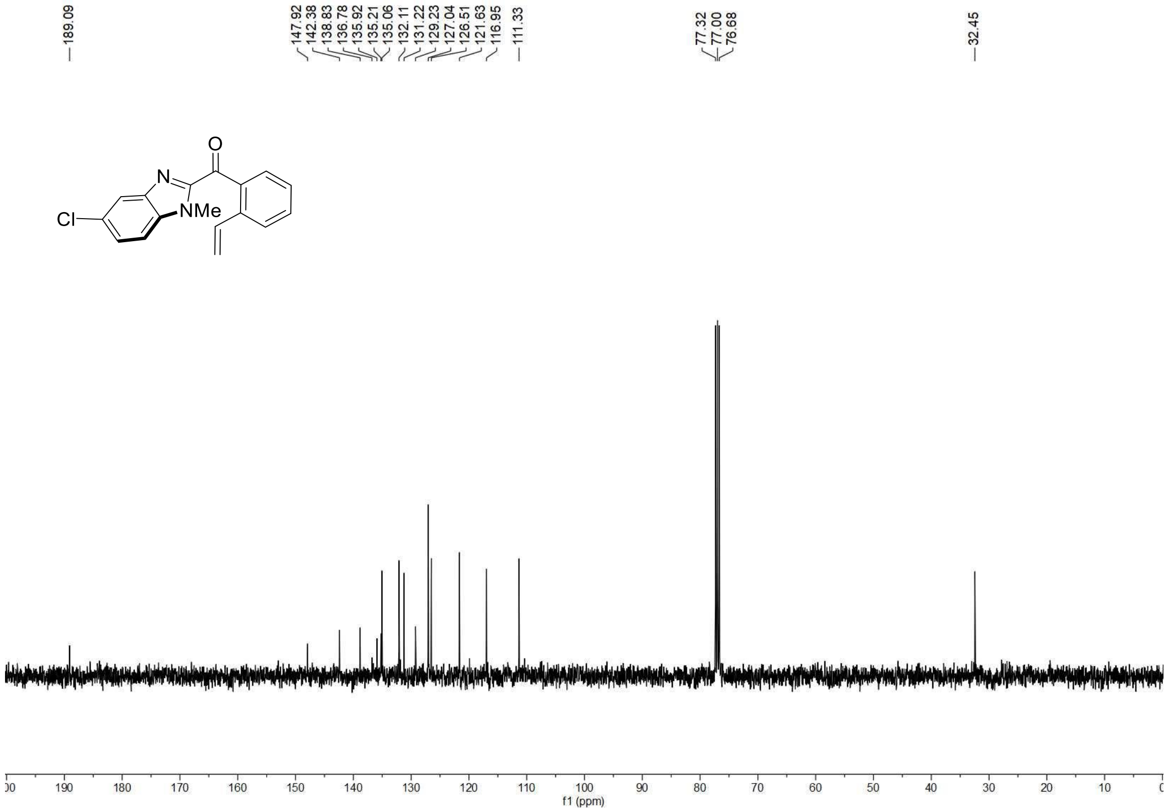

Supplementary Figure 11. ^{13}C-NMR of compound **(5-chloro-1-methyl-1*H*-benzo[*d*]imidazol-2-yl)(2-vinylphenyl)methanone**, recorded at 100 MHz and 25 °C in $CDCl_3$.

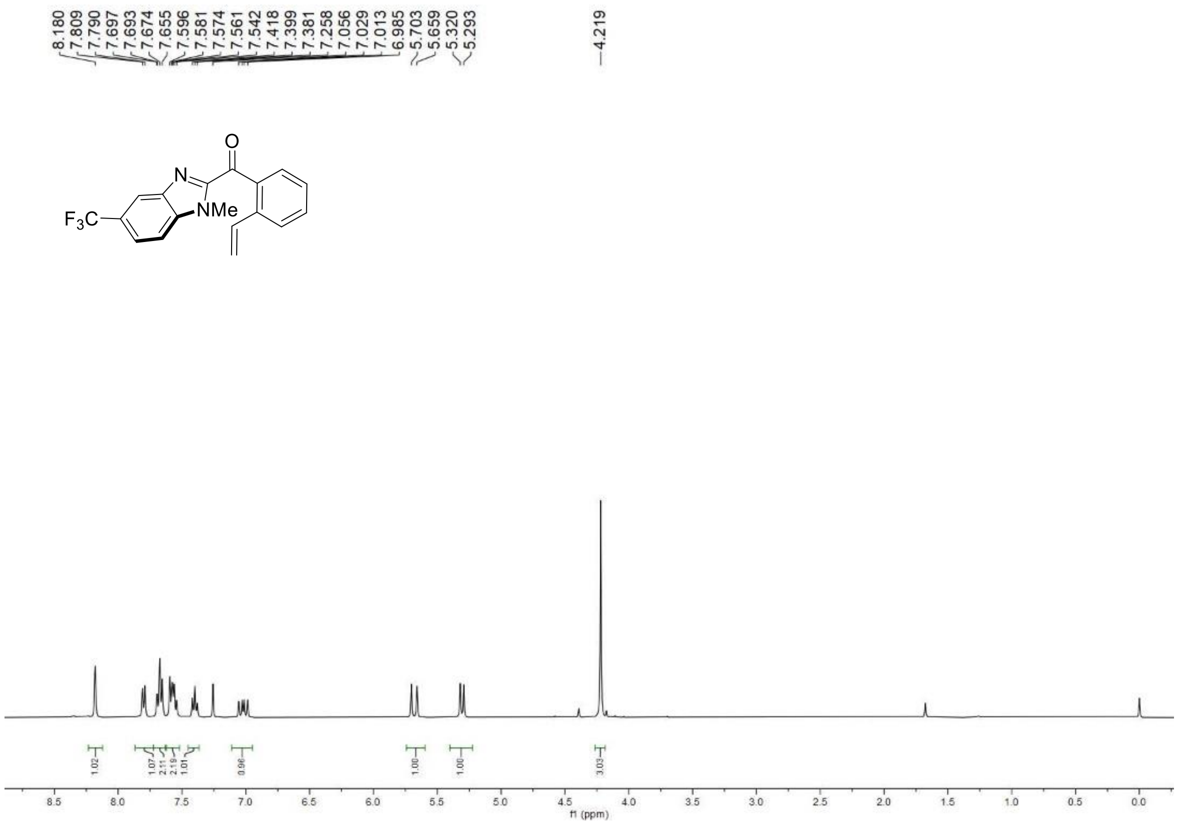

Supplementary Figure 12. ^{1}H-NMR of compound **(1-methyl-5-(trifluoromethyl)-1H-benzo[d]imidazol-2-yl)(2-vinylphenyl)methanone**, recorded at 400 MHz and 25 °C in $CDCl_3$.

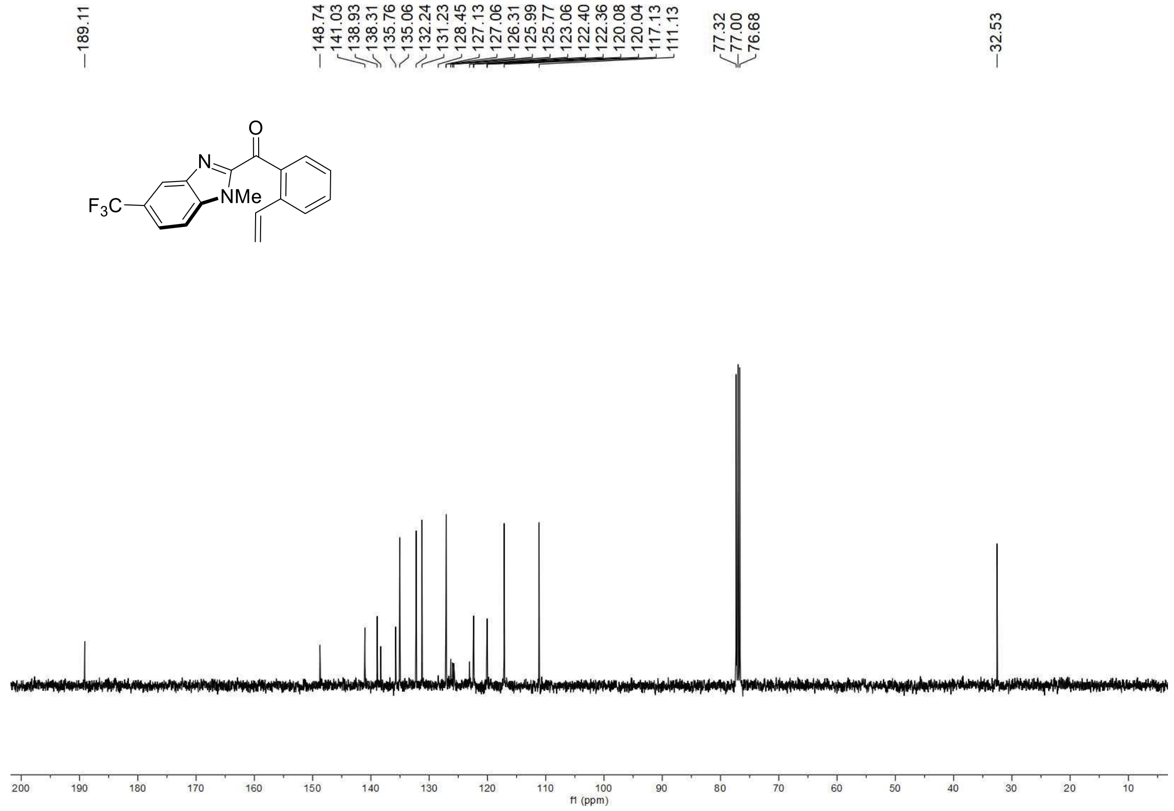

Supplementary Figure 13. ^{13}C-NMR of compound **(1-methyl-5-(trifluoromethyl)-1H-benzo[d]imidazol-2-yl)(2-vinylphenyl)methanone**, recorded at 100 MHz and 25 °C in $CDCl_3$.

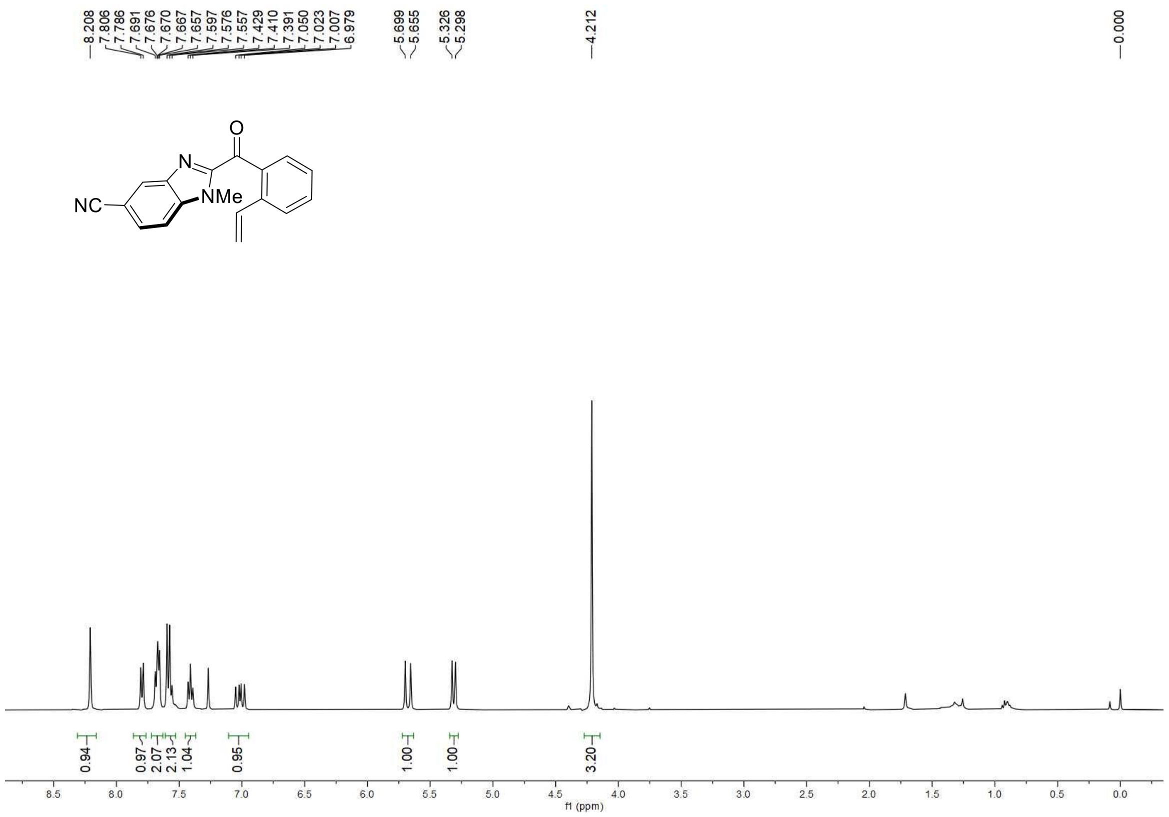

Supplementary Figure 14. ^{1}H-NMR of compound **1-methyl-2-(2-vinylbenzoyl)-1H-benzo[d]imidazole-5-carbonitrile**, recorded at 400 MHz and 25 °C in $CDCl_3$.

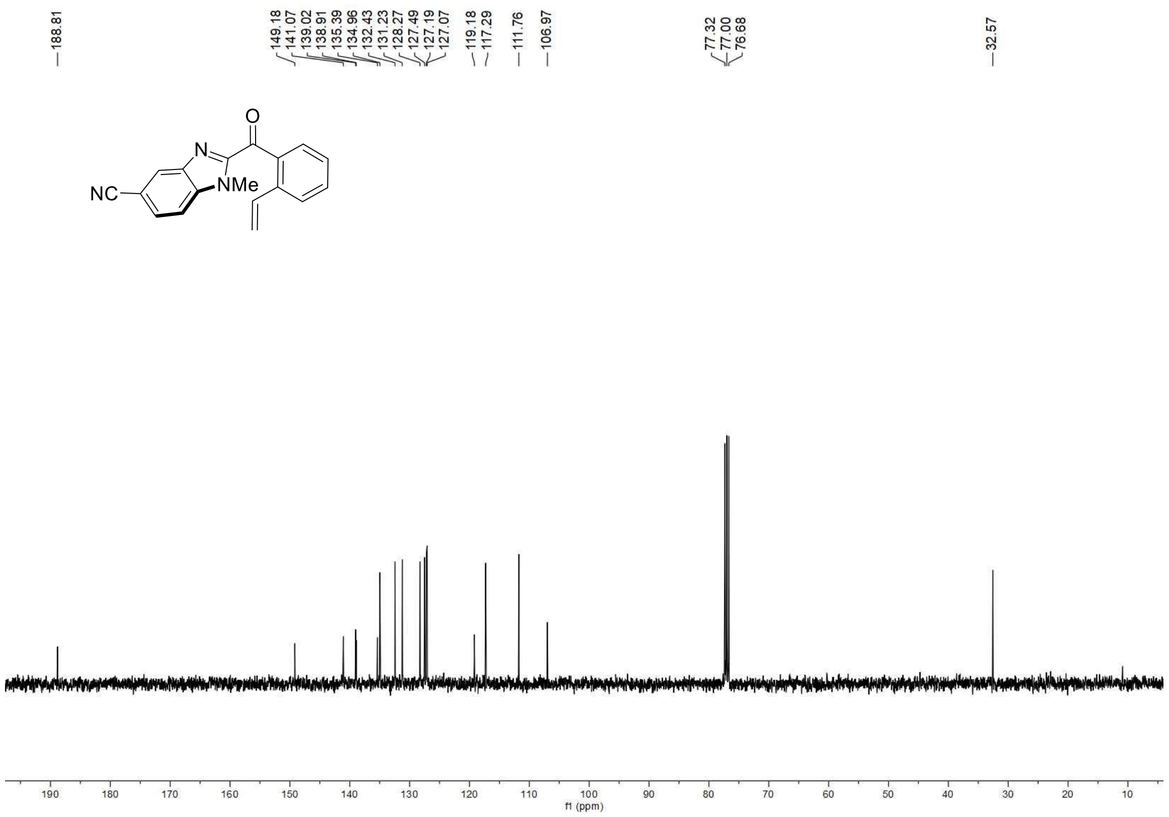

Supplementary Figure 15. ^{13}C-NMR of compound **1-methyl-2-(2-vinylbenzoyl)-1H-benzo[d]imidazole-5-carbonitrile**, recorded at 100 MHz and 25 °C in $CDCl_3$.

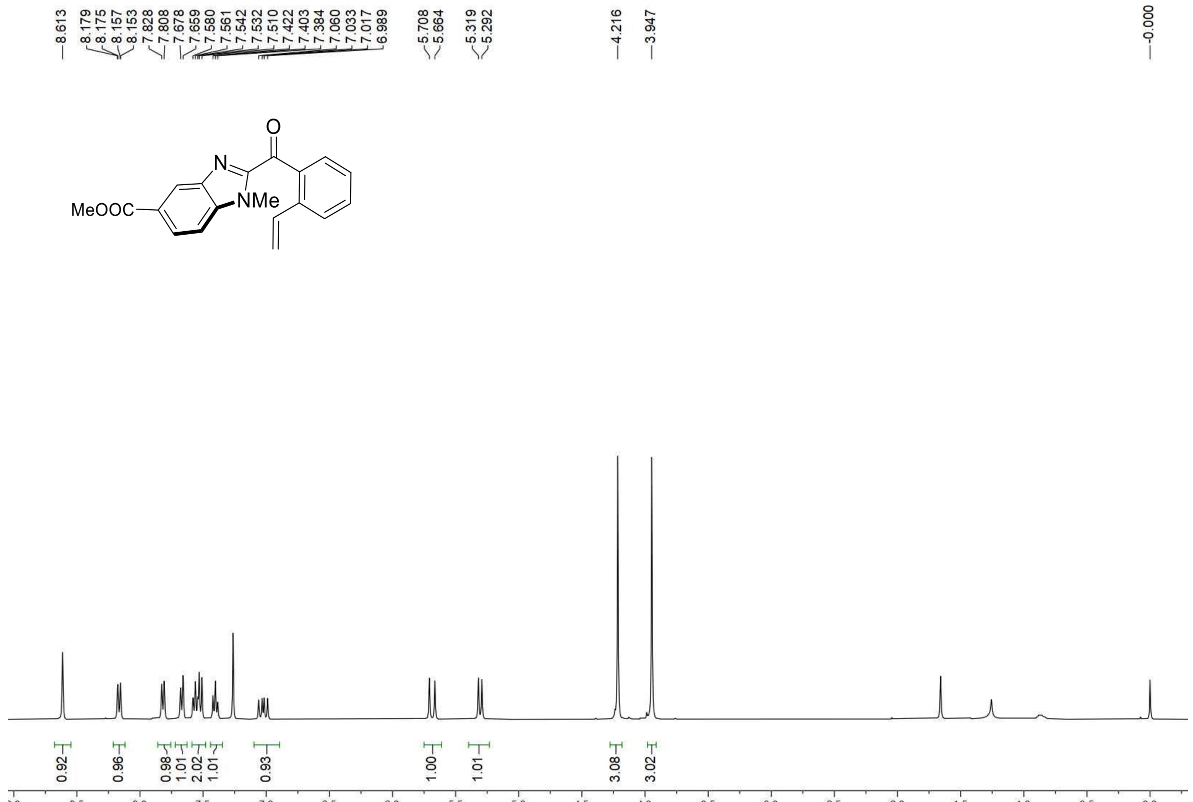

Supplementary Figure 16. ^{1}H-NMR of compound **methyl 1-methyl-2-(2-vinylbenzoyl)-1*H*-benzo[*d*]imidazole-5-carboxylate** , recorded at 400 MHz and 25 °C in $CDCl_3$.

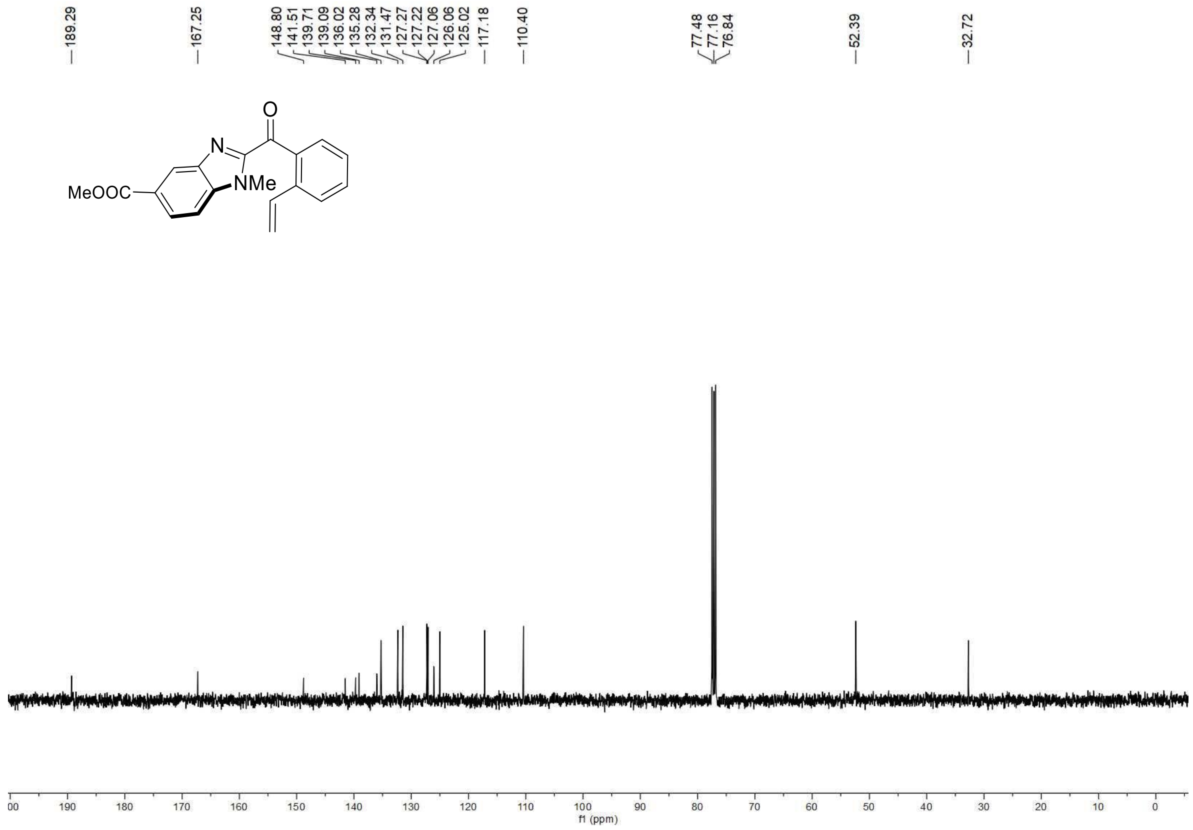

Supplementary Figure 17. ^{13}C-NMR of compound **methyl 1-methyl-2-(2-vinylbenzoyl)-1*H*-benzo[*d*]imidazole-5-carboxylate**, recorded at 100 MHz and 25 °C in $CDCl_3$.

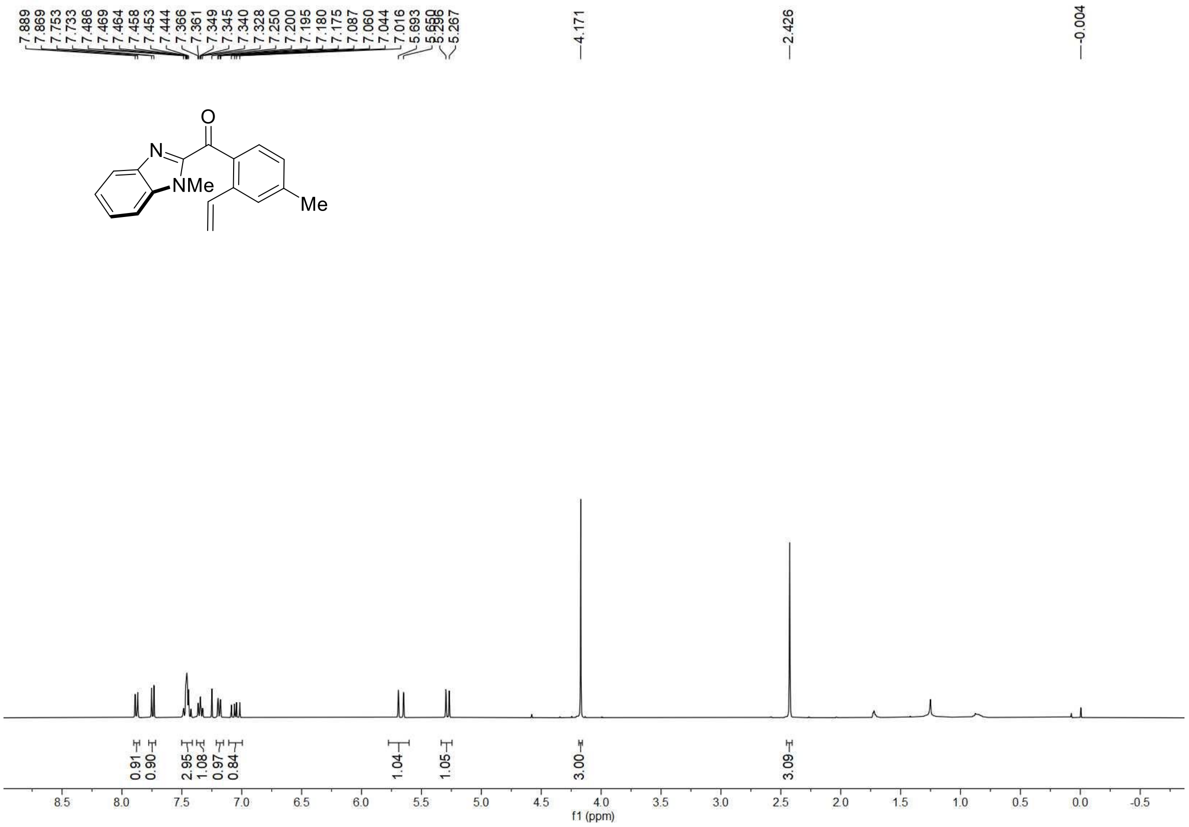

Supplementary Figure 18. ^{1}H-NMR of compound **(1-methyl-1*H*-benzo[*d*]imidazol-2-yl)(4-methyl-2-vinylphenyl)methanone**, recorded at 400 MHz and 25 °C in $CDCl_3$.

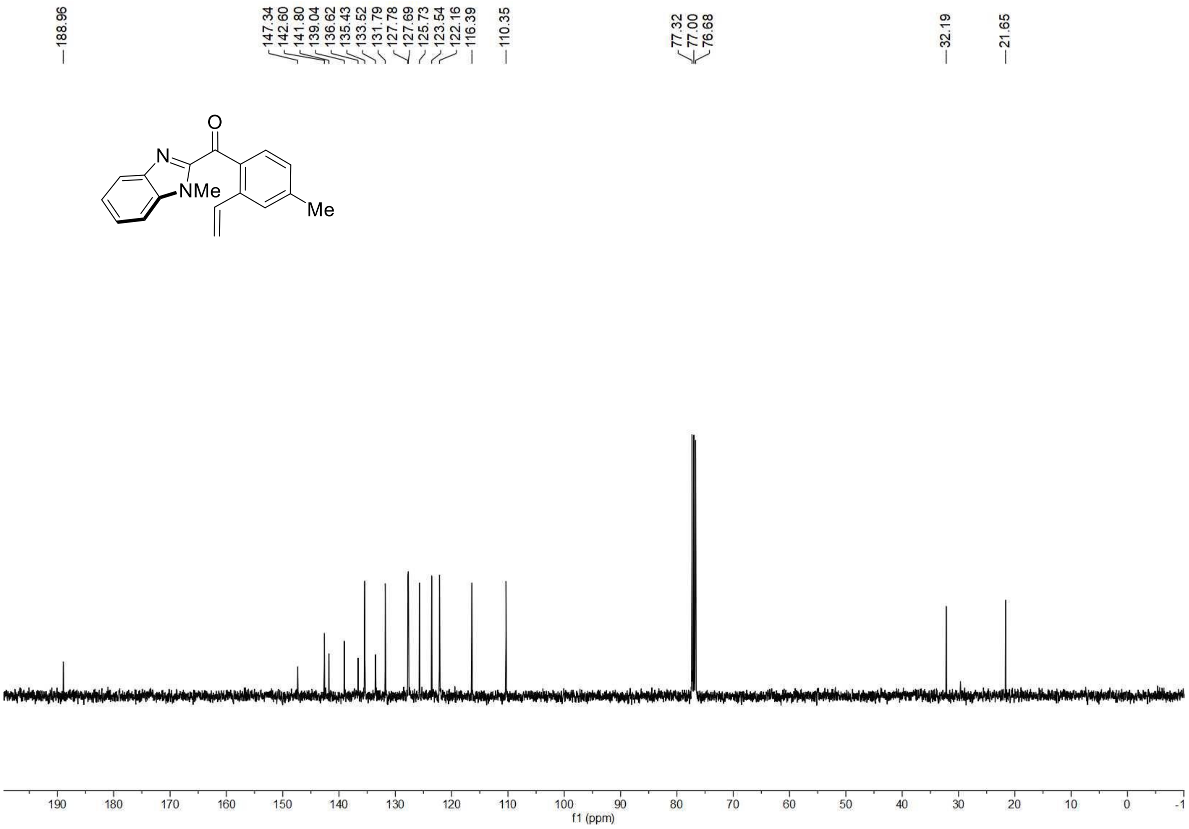

Supplementary Figure 19. ^{13}C-NMR of compound **(1-methyl-1*H*-benzo[*d*]imidazol-2-yl)(4-methyl-2-vinylphenyl)methanone**, recorded at 100 MHz and 25 °C in $CDCl_3$.

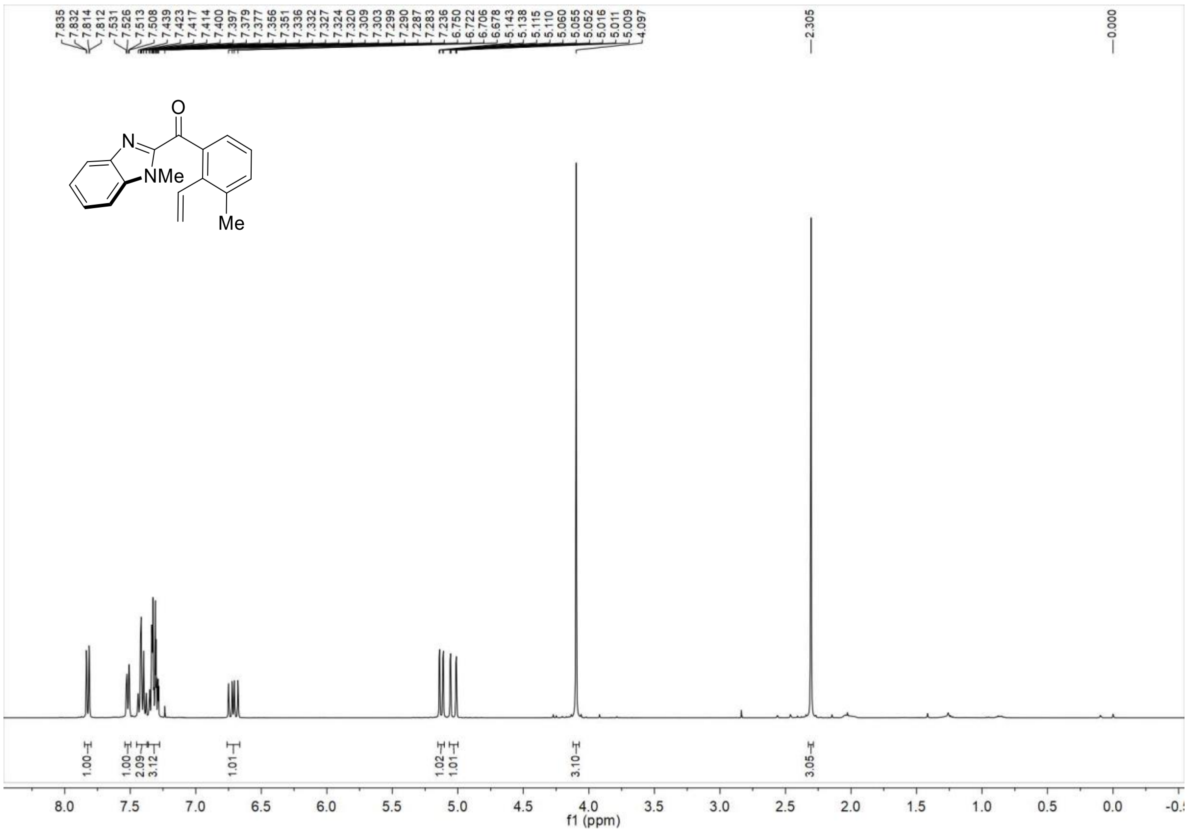

Supplementary Figure 20. ^{1}H-NMR of compound **(1-methyl-1*H*-benzo[*d*]imidazol-2-yl)(3-methyl-2-vinylphenyl)methanone**, recorded at 400 MHz and 25 °C in $CDCl_3$.

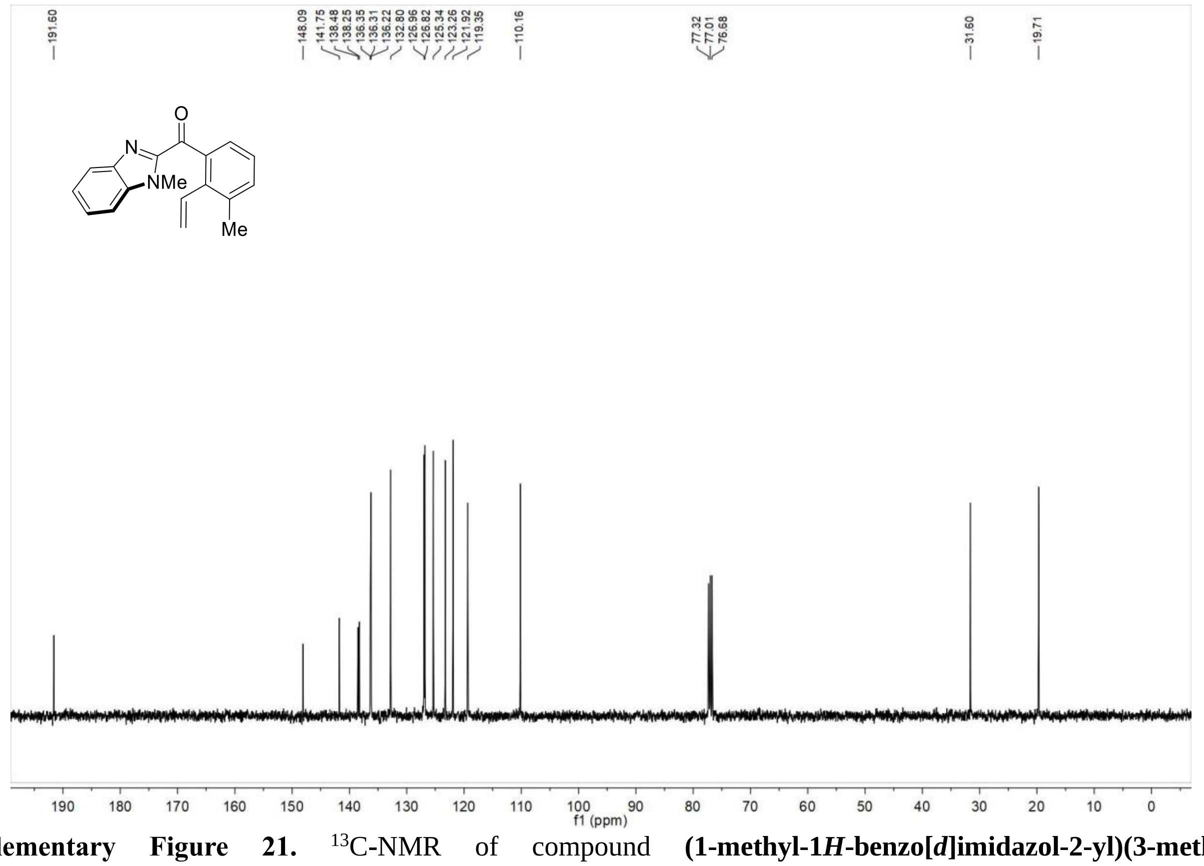

Supplementary Figure 21. ^{13}C-NMR of compound **(1-methyl-1*H*-benzo[*d*]imidazol-2-yl)(3-methyl-2-vinylphenyl)methanone**, recorded at 100 MHz and 25 °C in $CDCl_3$.

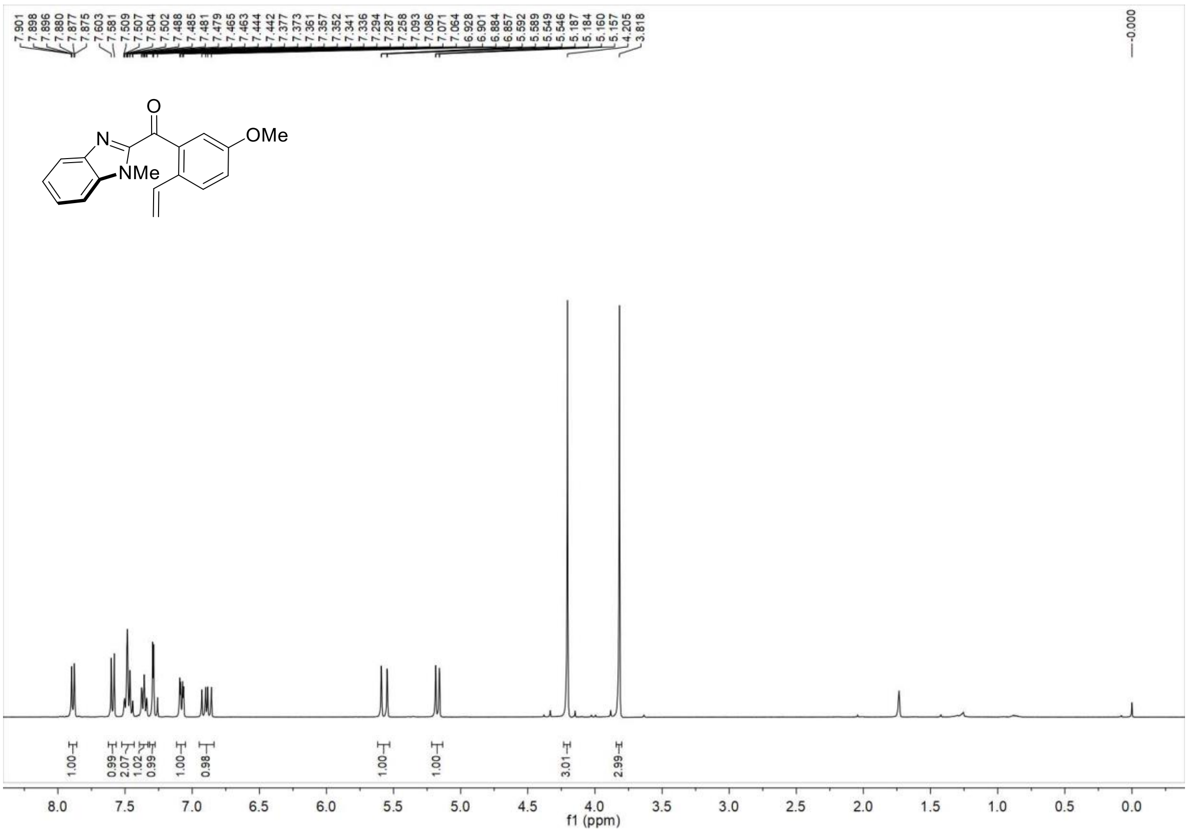

Supplementary Figure 22. ^{1}H-NMR of compound **(5-methoxy-2-vinylphenyl)(1-methyl-1*H*-benzo[*d*]imidazol-2-yl)methanone**, recorded at 400 MHz and 25 °C in $CDCl_3$.

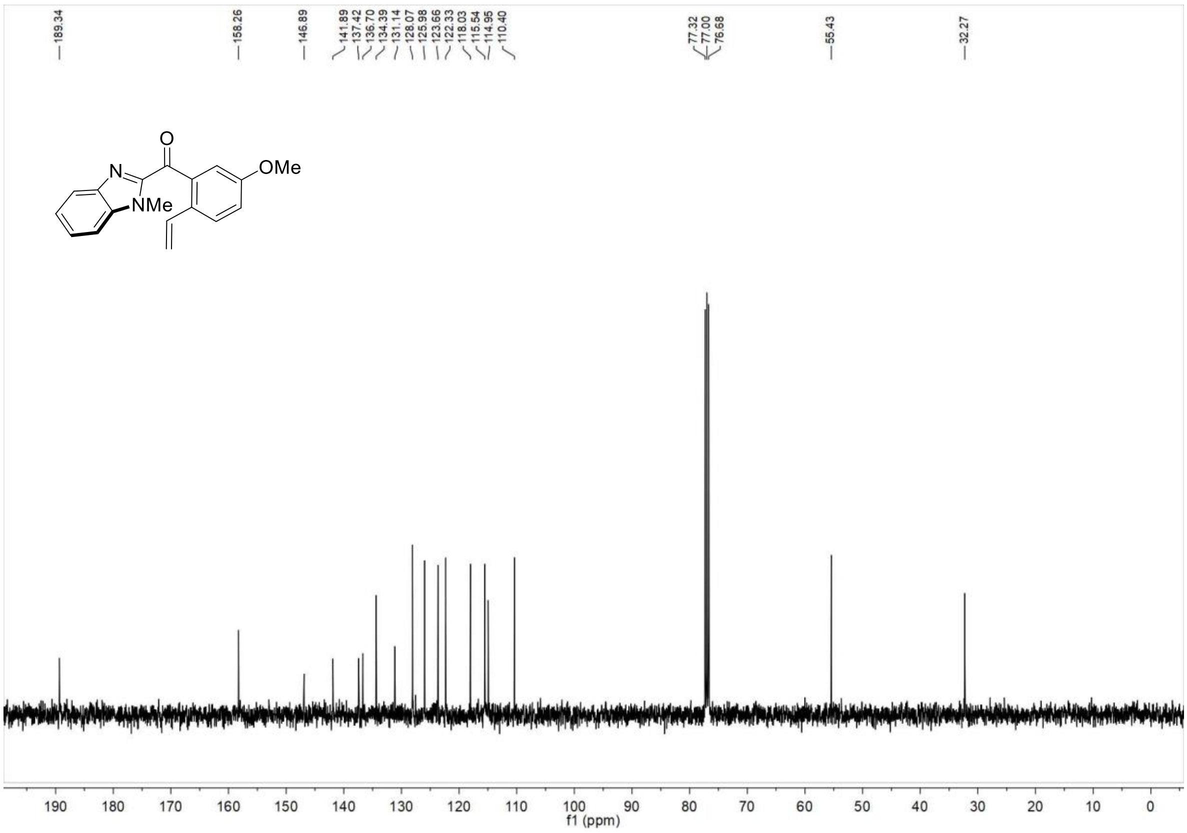

Supplementary Figure 23. ^{13}C-NMR of compound **(5-methoxy-2-vinylphenyl)(1-methyl-1*H*-benzo[*d*]imidazol-2-yl)methanone**, recorded at 100 MHz and 25 °C in $CDCl_3$

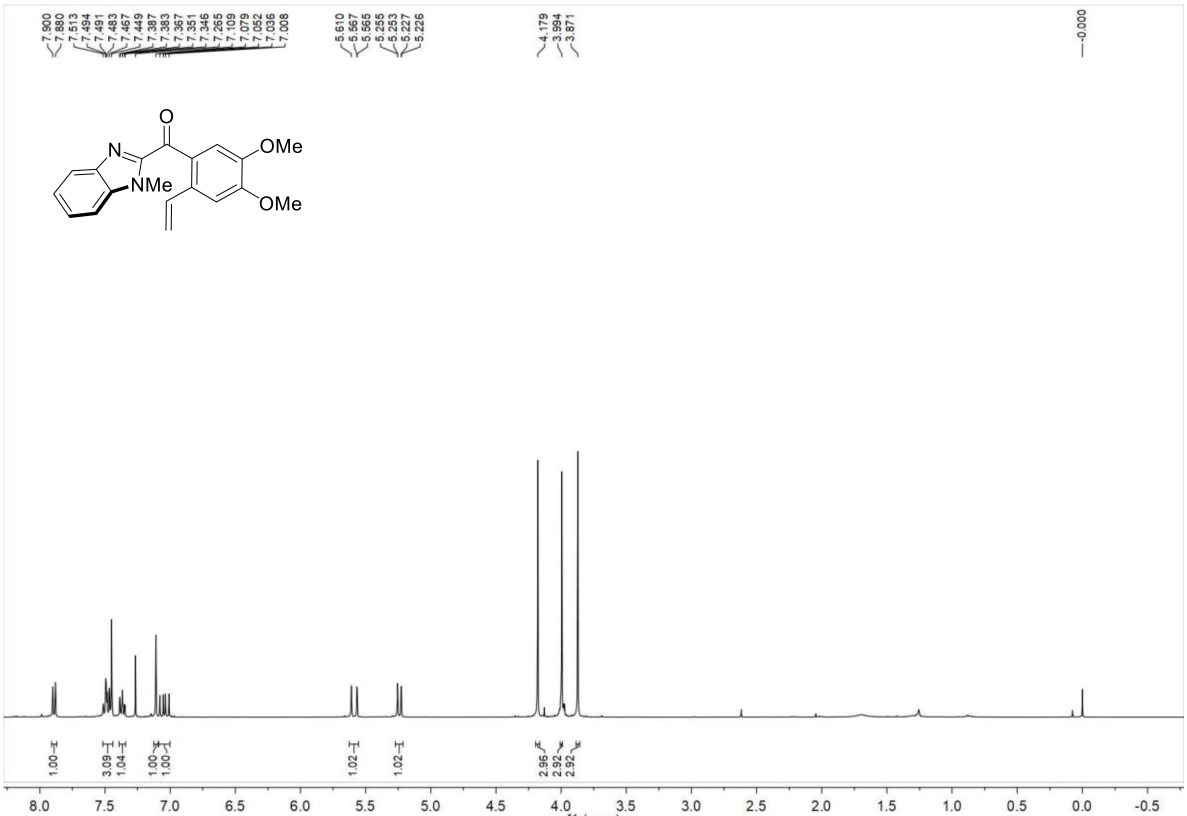

Supplementary Figure 24. ^{1}H-NMR of compound **(4,5-dimethoxy-2-vinylphenyl)(1-methyl-1*H*-benzo[*d*]imidazol-2-yl)methanone**, recorded at 400 MHz and 25 °C in $CDCl_3$.

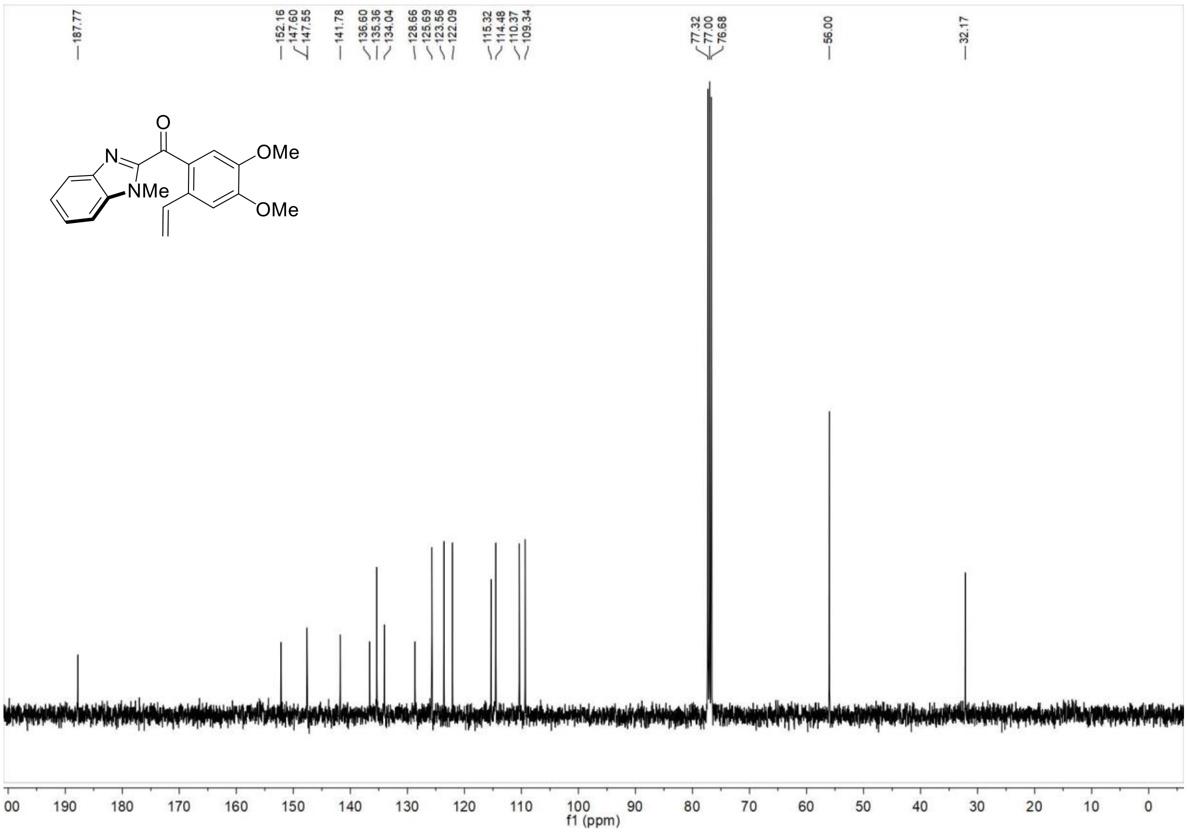

Supplementary Figure 25. ^{13}C-NMR of compound **(4,5-dimethoxy-2-vinylphenyl)(1-methyl-1*H*-benzo[*d*]imidazol-2-yl)methanone**, recorded at 100 MHz and 25 °C in $CDCl_3$.

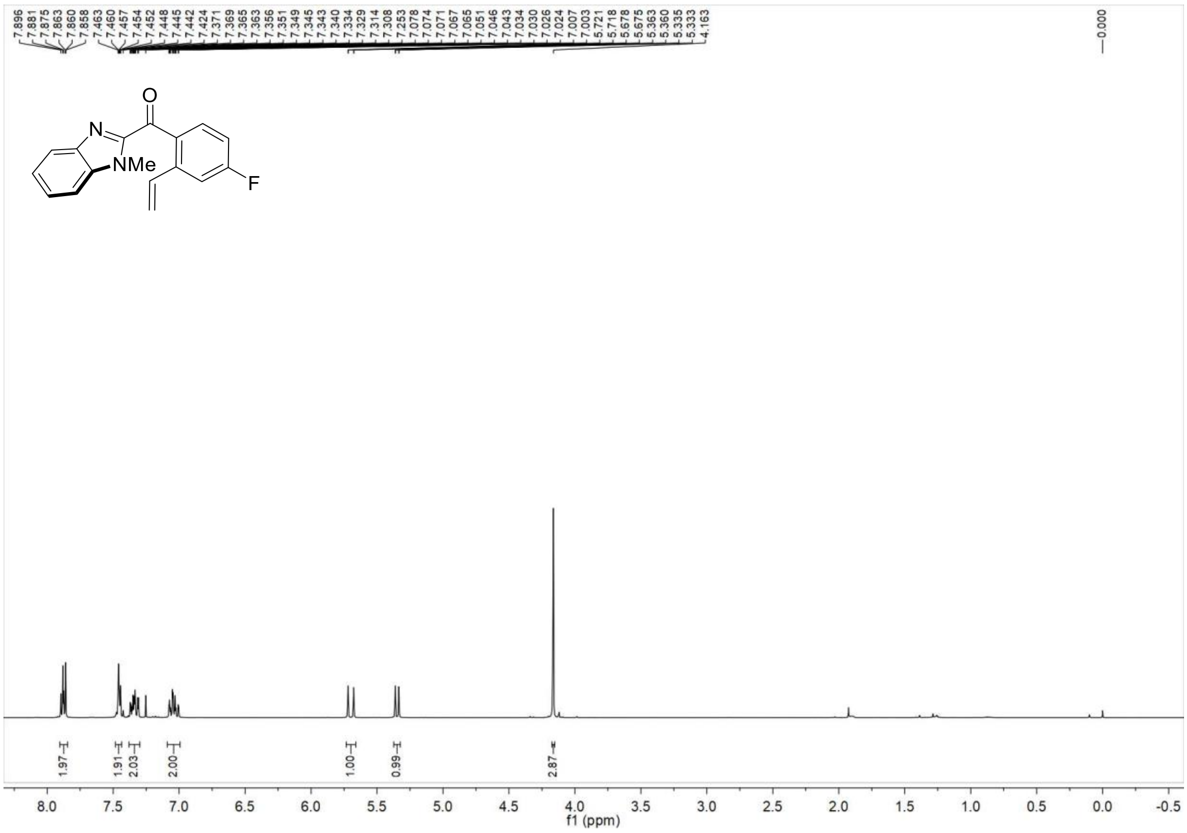

Supplementary Figure 26. ^{1}H-NMR of compound **(4-fluoro-2-vinylphenyl)(1-methyl-1*H*-benzo[*d*]imidazol-2-yl)methanone**, recorded at 400 MHz and 25 °C in $CDCl_3$.

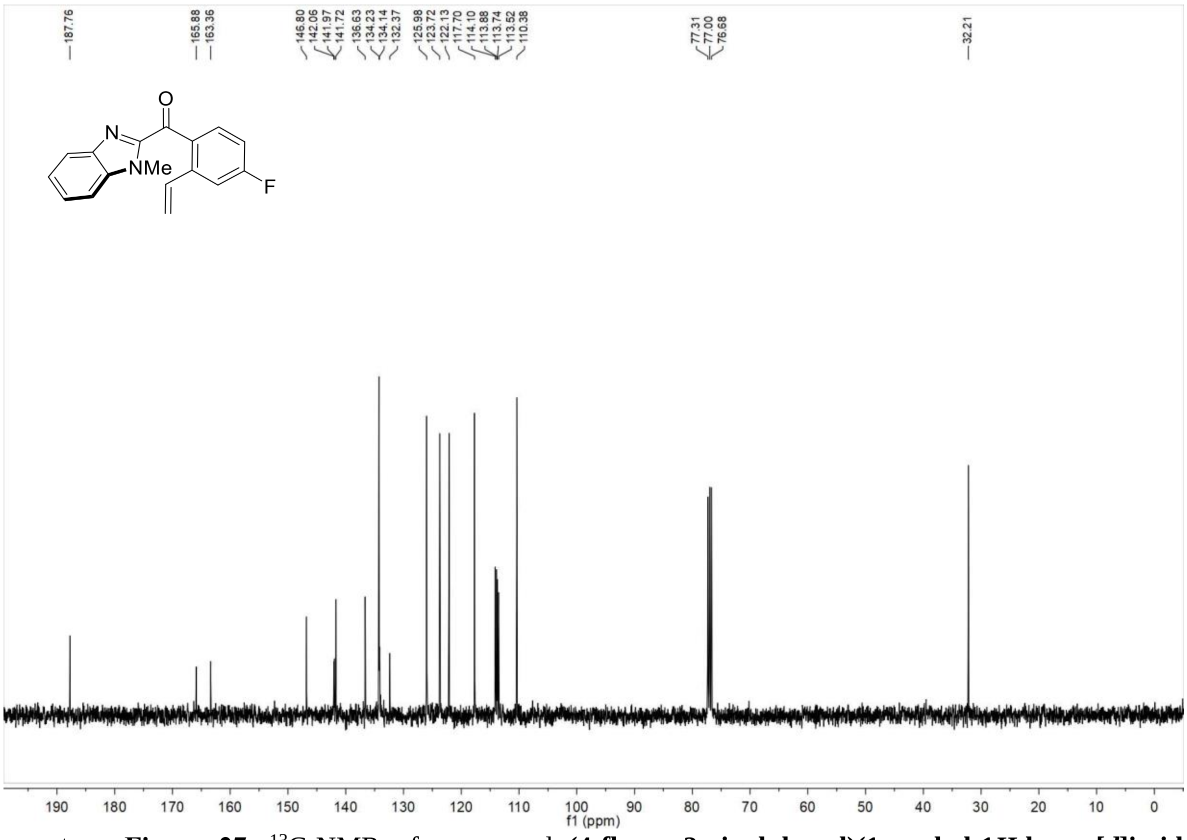

Supplementary Figure 27. ^{13}C-NMR of compound **(4-fluoro-2-vinylphenyl)(1-methyl-1*H*-benzo[*d*]imidazol-2-yl)methanone**, recorded at 100 MHz and 25 °C in $CDCl_3$.

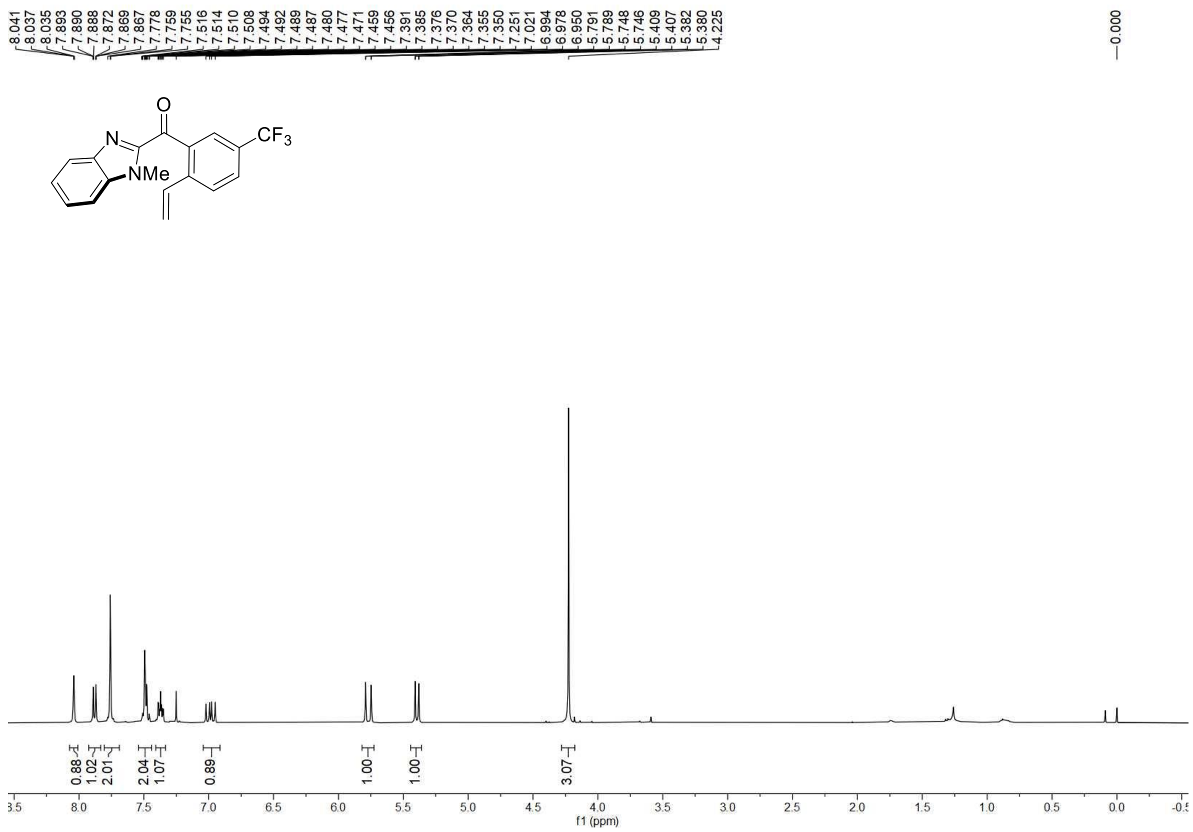

Supplementary Figure 28. ^{1}H-NMR of compound **(1-methyl-1*H*-benzo[*d*]imidazol-2-yl)(5-(trifluoromethyl)-2-vinylphenyl)methanone**, recorded at 400 MHz and 25 °C in $CDCl_3$.

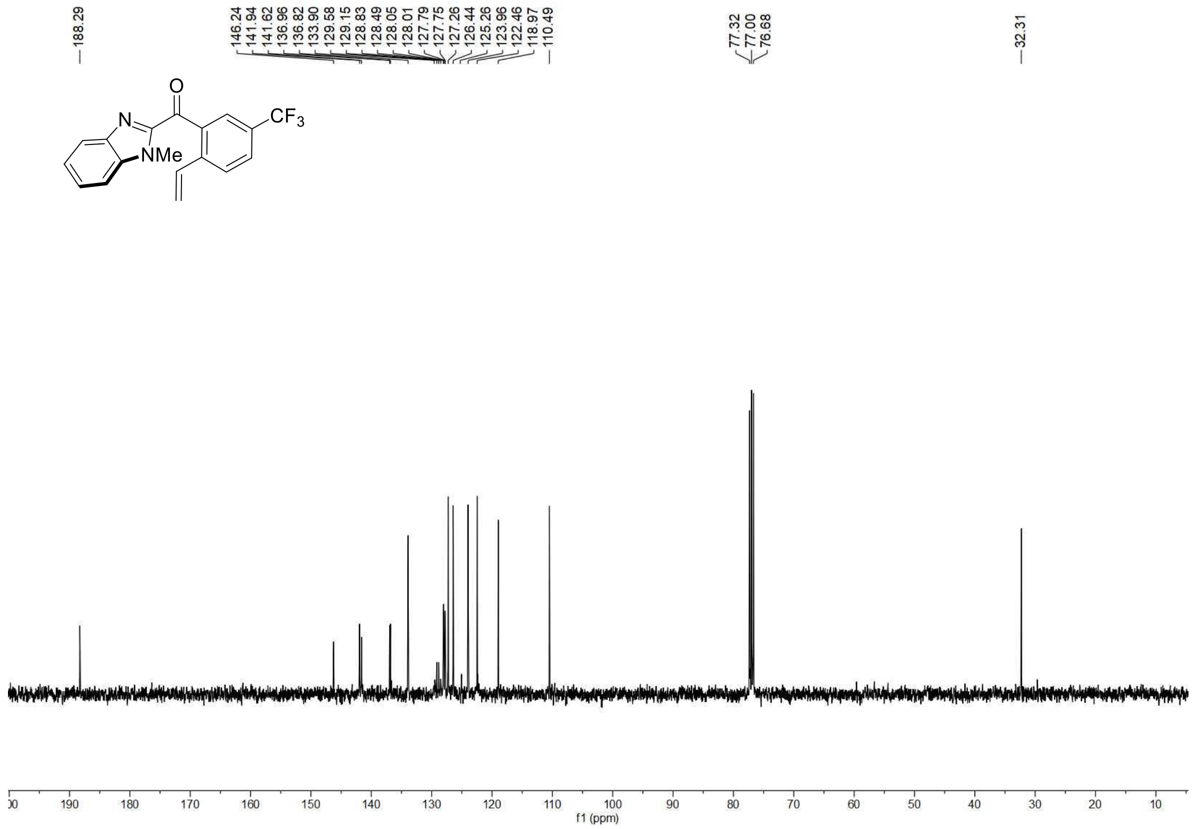

Supplementary Figure 29. ^{13}C-NMR of compound **(1-methyl-1*H*-benzo[*d*]imidazol-2-yl)(5-(trifluoromethyl)-2-vinylphenyl)methanone**, recorded at 100 MHz and 25 °C in $CDCl_3$.

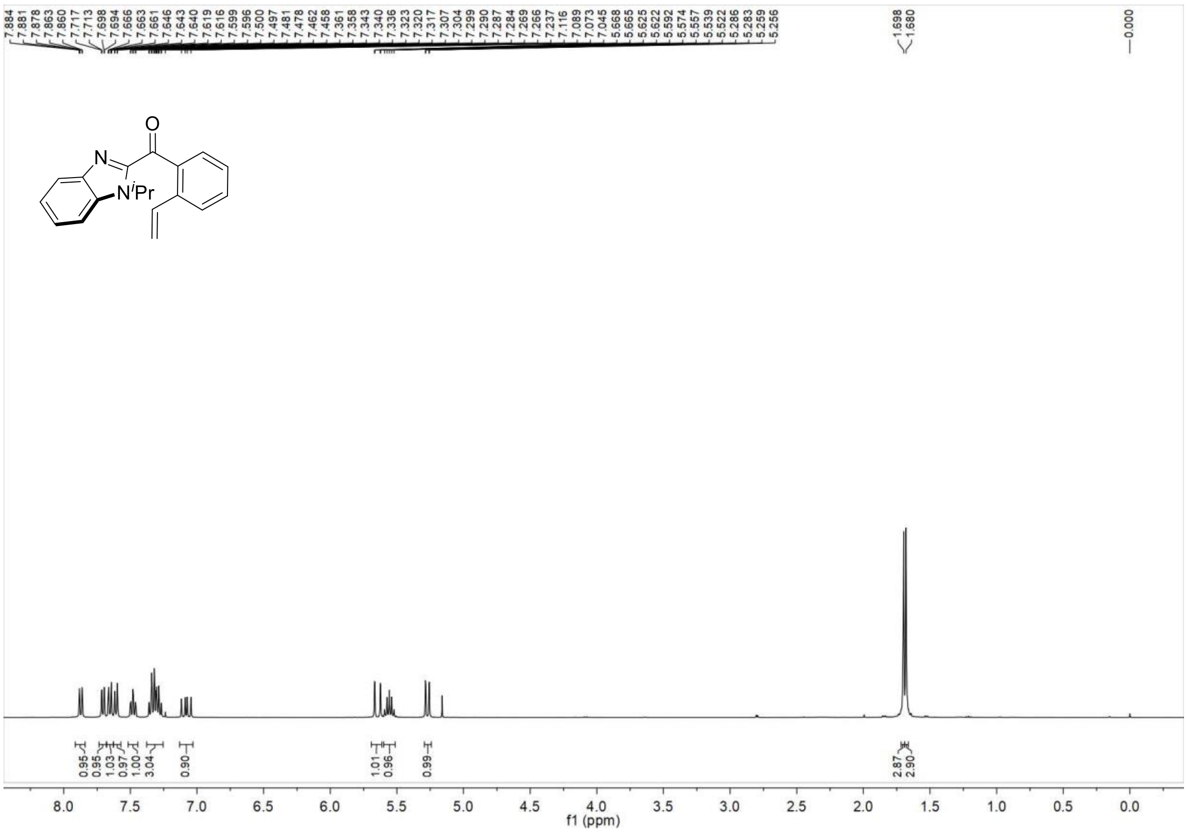

Supplementary Figure 30. ^{1}H-NMR of compound **(1-isopropyl-1*H*-benzo[*d*]imidazol-2-yl)(2-vinylphenyl)methanone**, recorded at 400 MHz and 25 °C in $CDCl_3$.

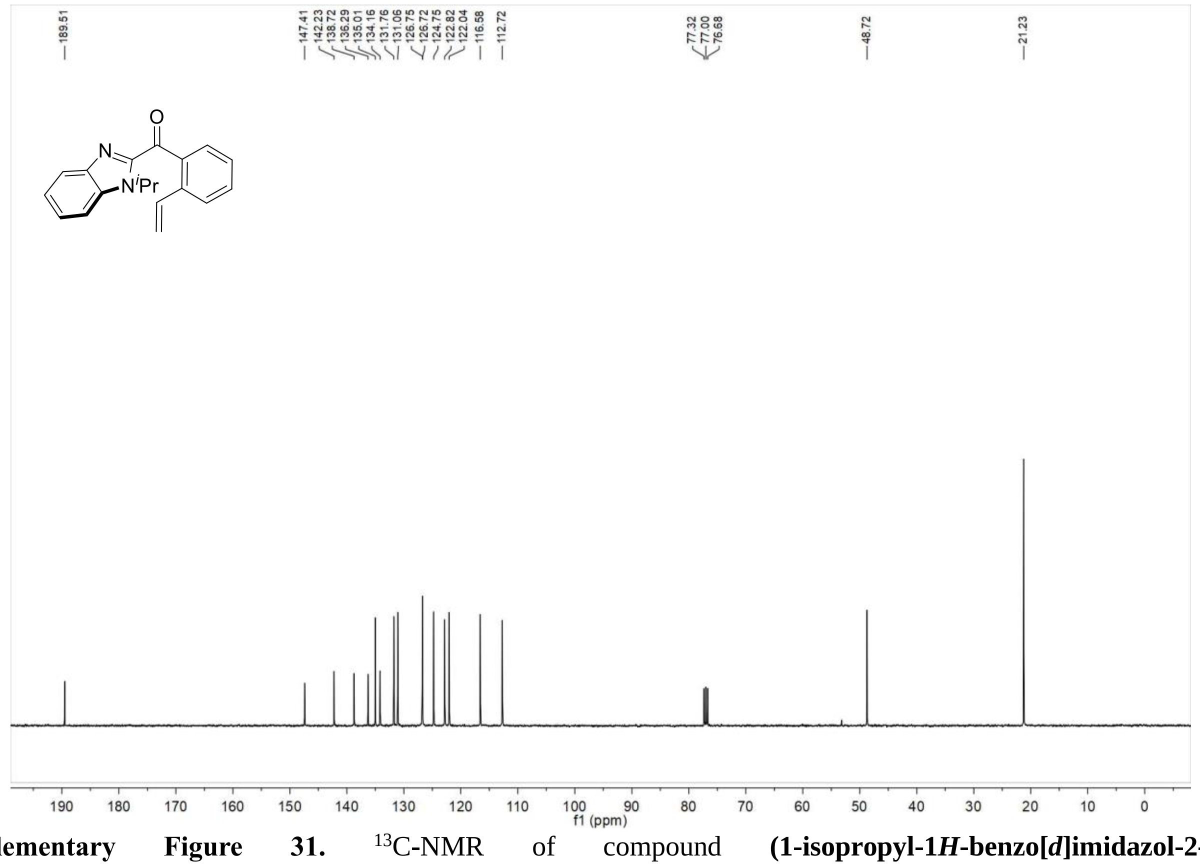

Supplementary Figure 31. ^{13}C-NMR of compound **(1-isopropyl-1*H*-benzo[*d*]imidazol-2-yl)(2-vinylphenyl)methanone**, recorded at 100 MHz and 25 °C in $CDCl_3$.

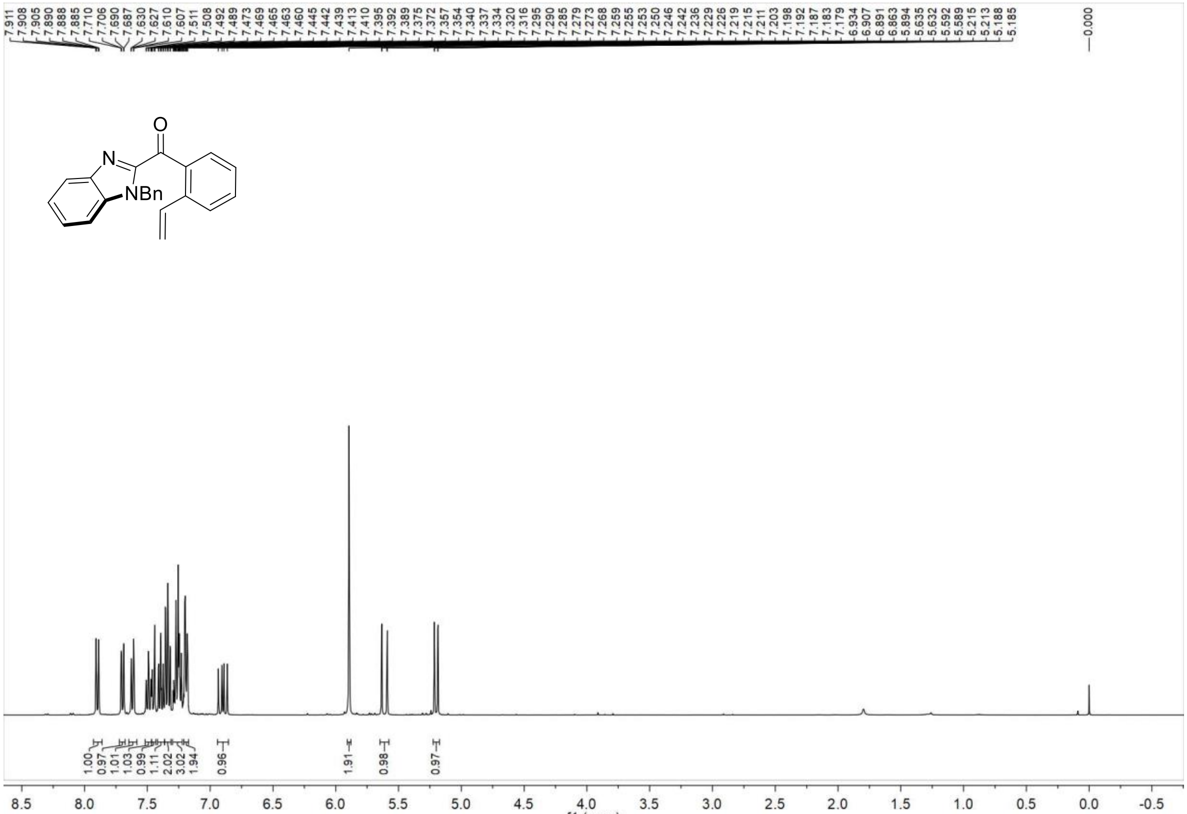

Supplementary Figure 32. ^{1}H-NMR of compound **(1-benzyl-1*H*-benzo[*d*]imidazol-2-yl)(2-vinylphenyl)methanone**, recorded at 400 MHz and 25 °C in $CDCl_3$.

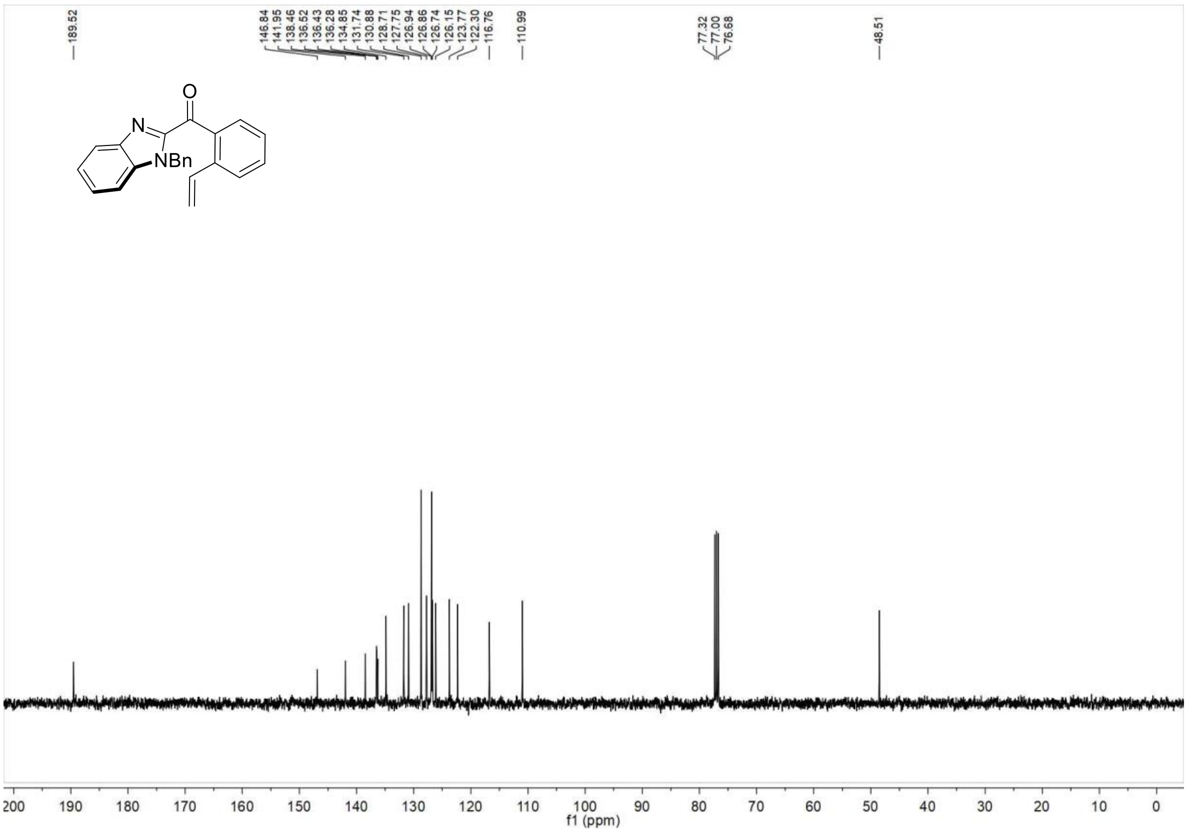

Supplementary Figure 33. ^{13}C-NMR of compound **(1-benzyl-1*H*-benzo[*d*]imidazol-2-yl)(2-vinylphenyl)methanone**, recorded at 100 MHz and 25 °C in $CDCl_3$.

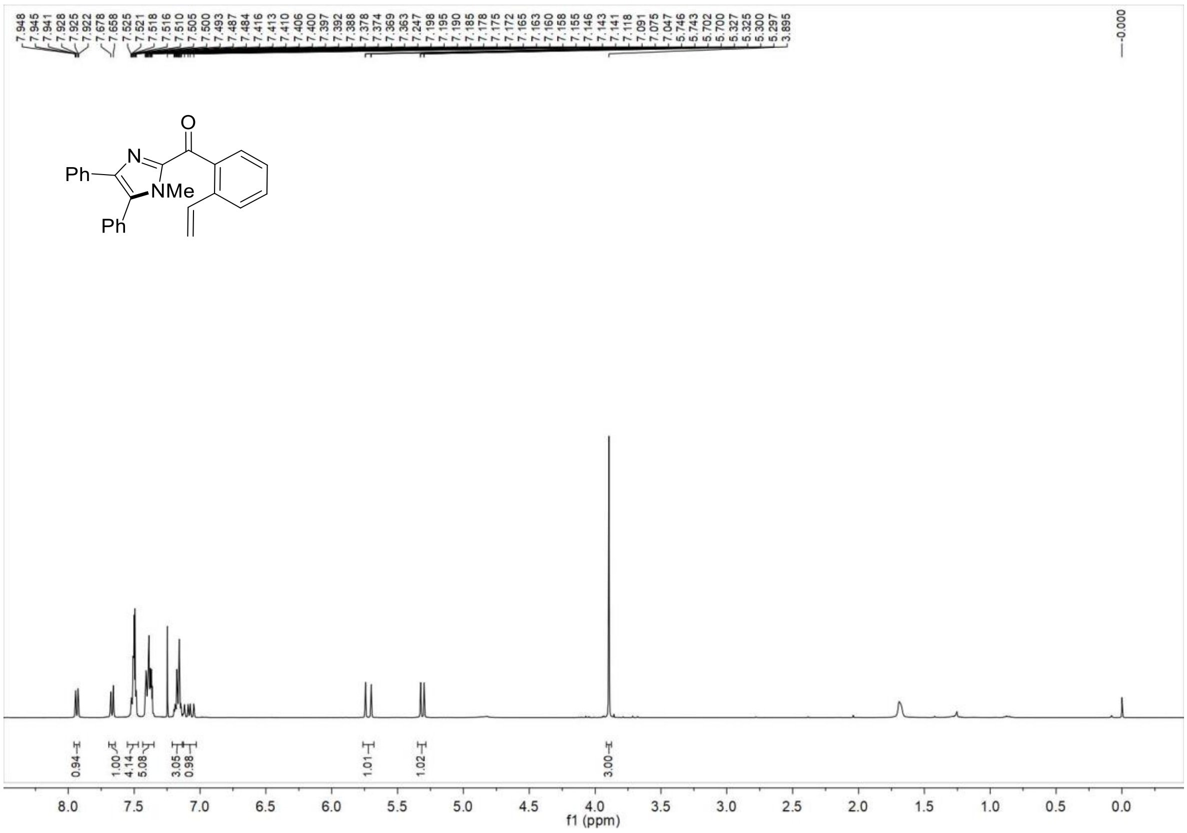

Supplementary Figure 34. ^{1}H-NMR of compound **(1-methyl-4,5-diphenyl-1*H*-imidazol-2-yl)(2-vinylphenyl)methanone**, recorded at 400 MHz and 25 °C in $CDCl_3$.

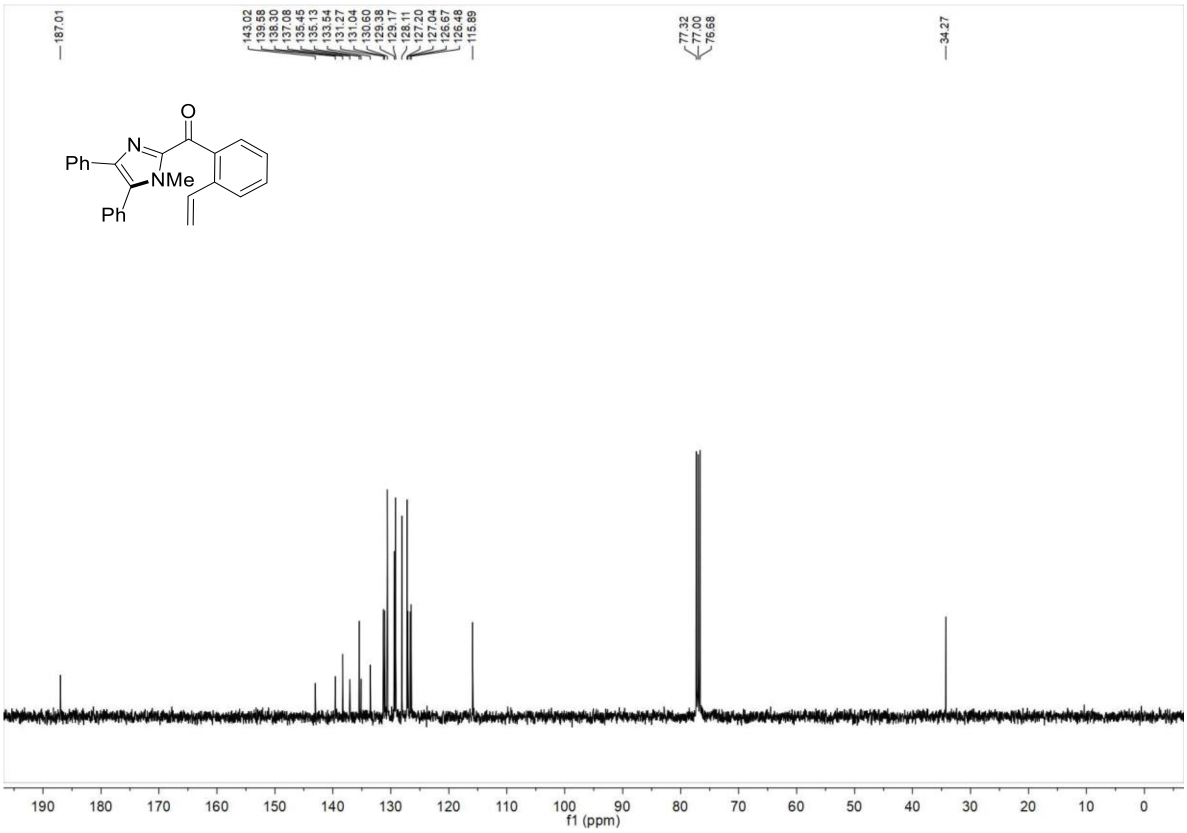

Supplementary Figure 35. ^{13}C-NMR of compound **(1-methyl-4,5-diphenyl-1*H*-imidazol-2-yl)(2-vinylphenyl)methanone**, recorded at 100 MHz and 25 °C in $CDCl_3$.

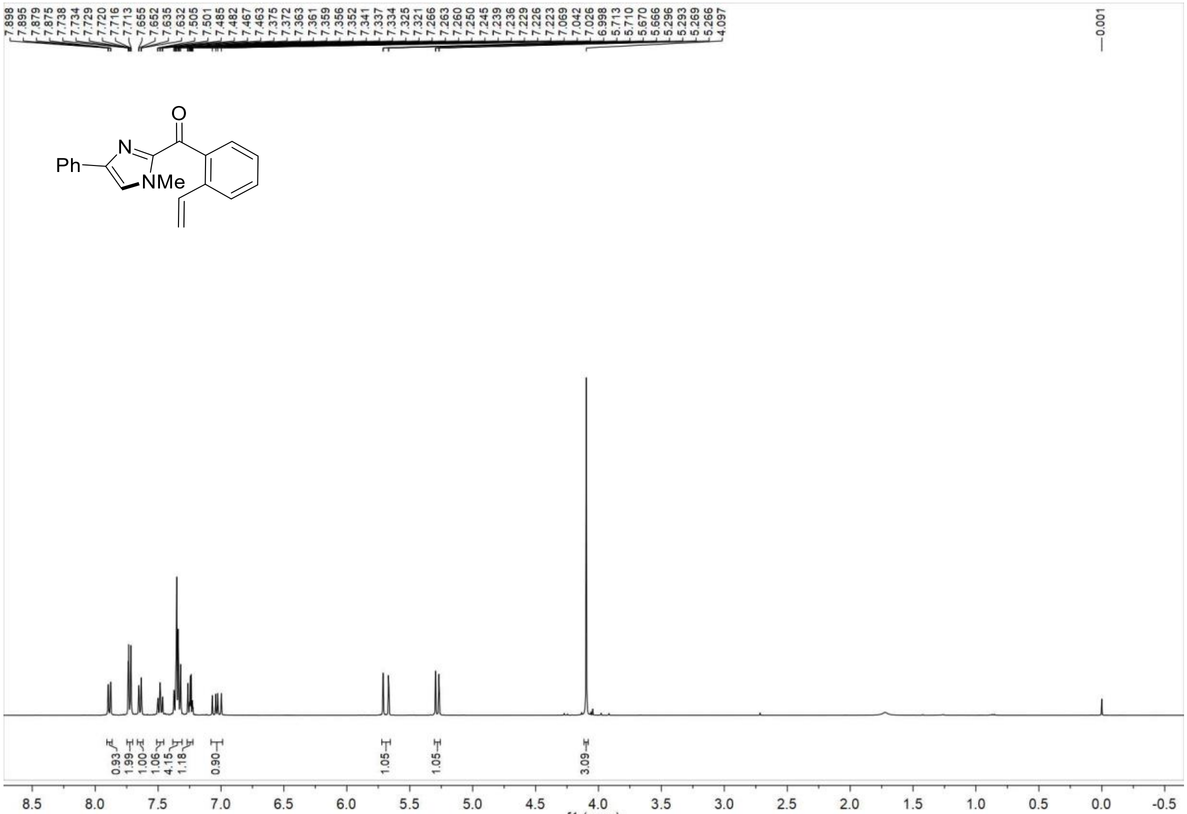

Supplementary Figure 36. ^{1}H-NMR of compound **(1-methyl-4-phenyl-1*H*-imidazol-2-yl)(2-vinylphenyl)methanone**, recorded at 400 MHz and 25 °C in $CDCl_3$.

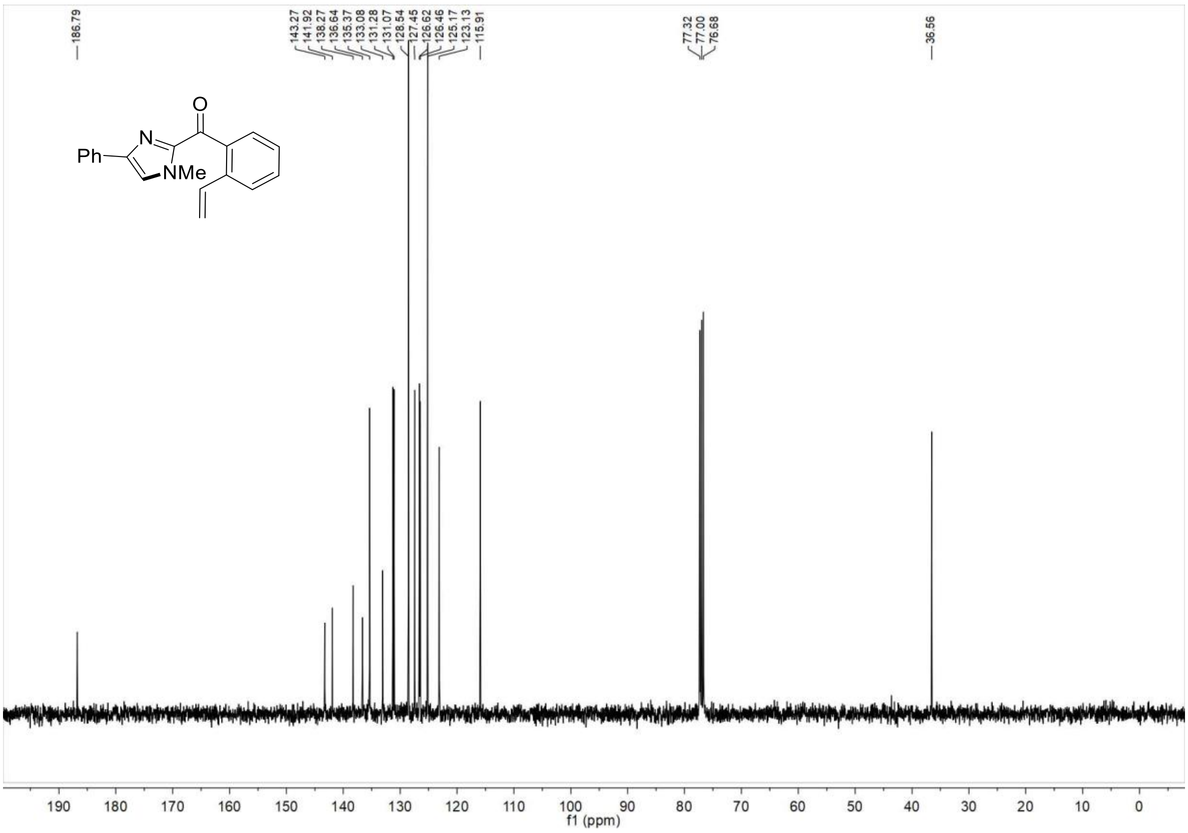

Supplementary Figure 37. ^{13}C-NMR of compound **(1-methyl-4-phenyl-1*H*-imidazol-2-yl)(2-vinylphenyl)methanone**, recorded at 100 MHz and 25 °C in $CDCl_3$.

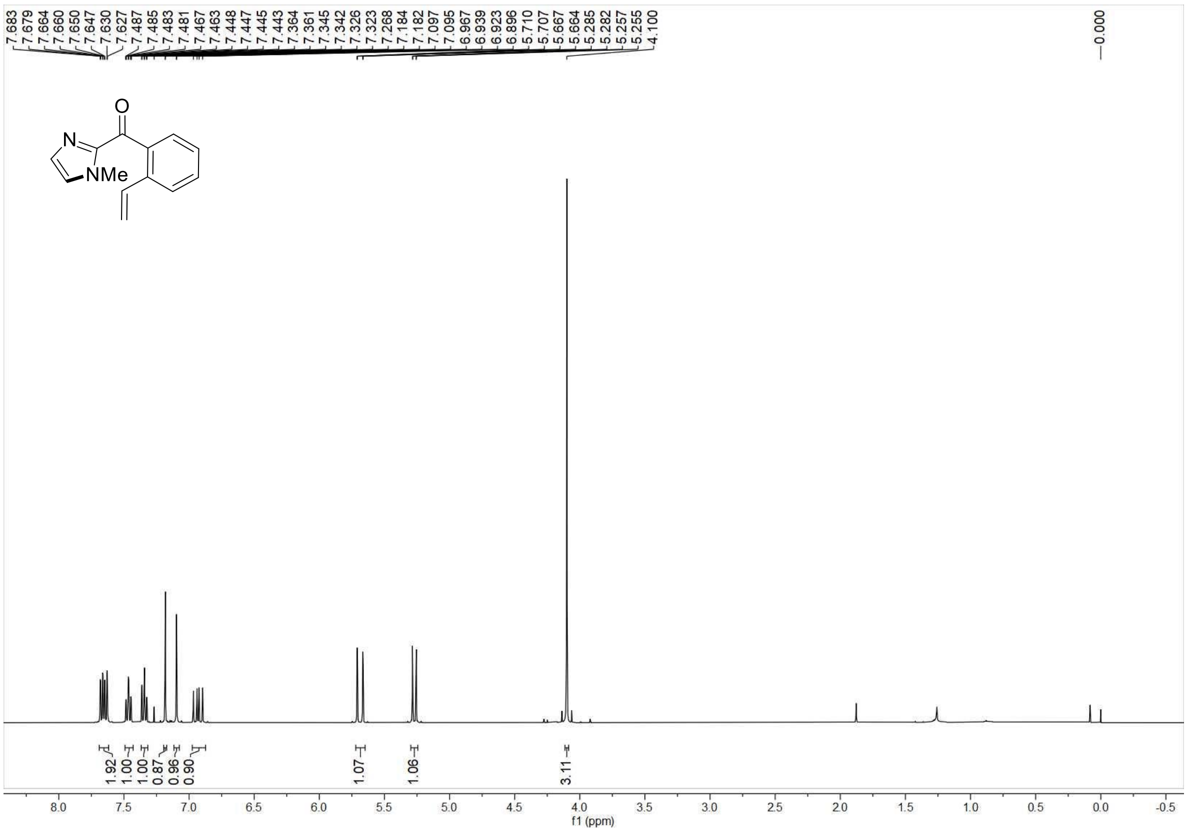

Supplementary Figure 38. ^{1}H-NMR of compound **(1-methyl-1*H*-imidazol-2-yl)(2-vinylphenyl)methanone**, recorded at 400 MHz and 25 °C in $CDCl_3$.

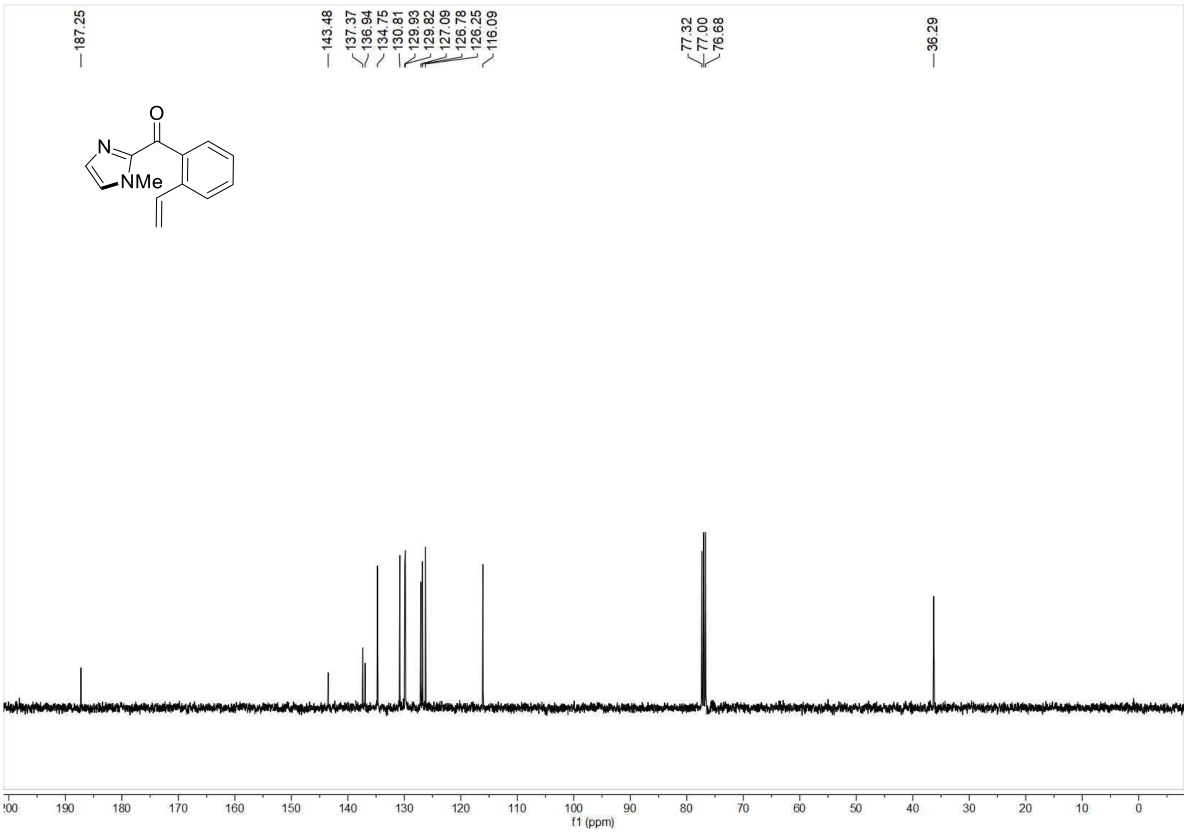

Supplementary Figure 39. ^{13}C-NMR of compound **(1-methyl-1*H*-imidazol-2-yl)(2-vinylphenyl)methanone**, recorded at 100 MHz and 25 °C in $CDCl_3$.

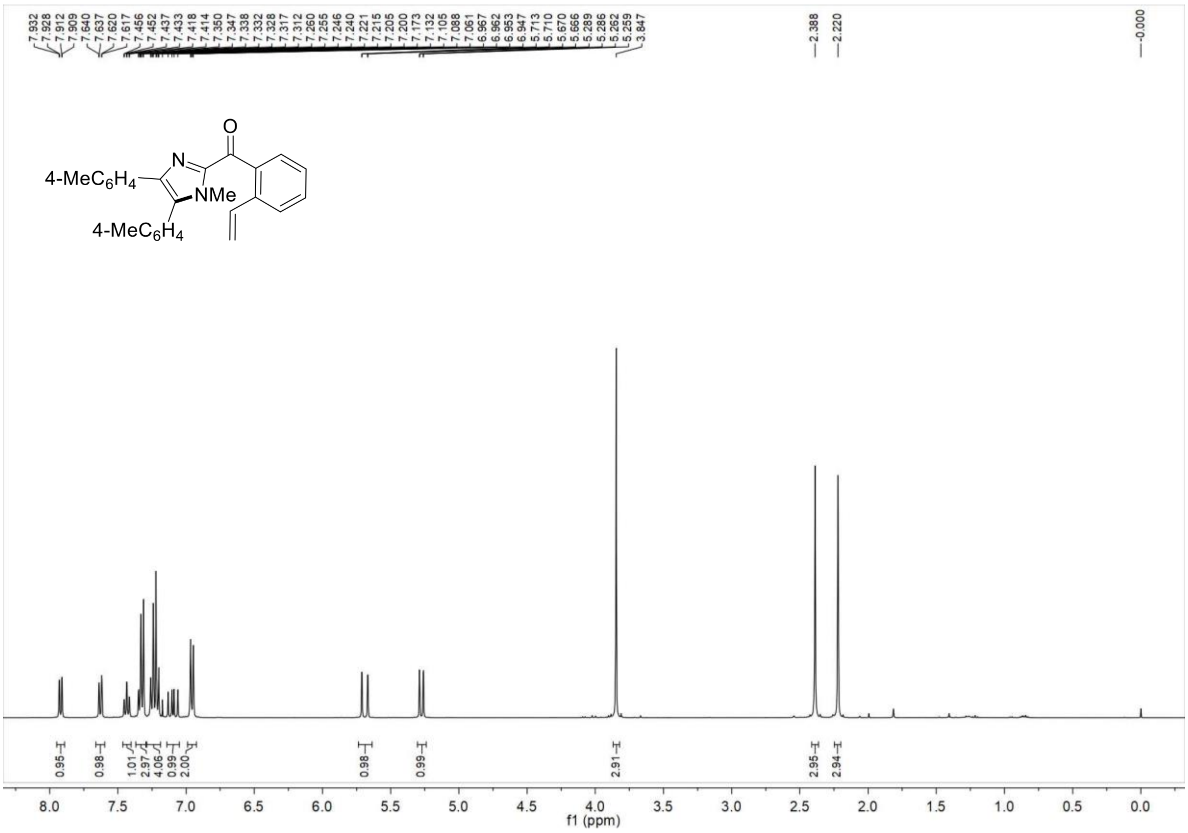

Supplementary Figure 40. ^{1}H-NMR of compound **(1-methyl-4,5-di-p-tolyl-1*H*-imidazol-2-yl)(2-vinylphenyl)methanone**, recorded at 400 MHz and 25 °C in $CDCl_3$.

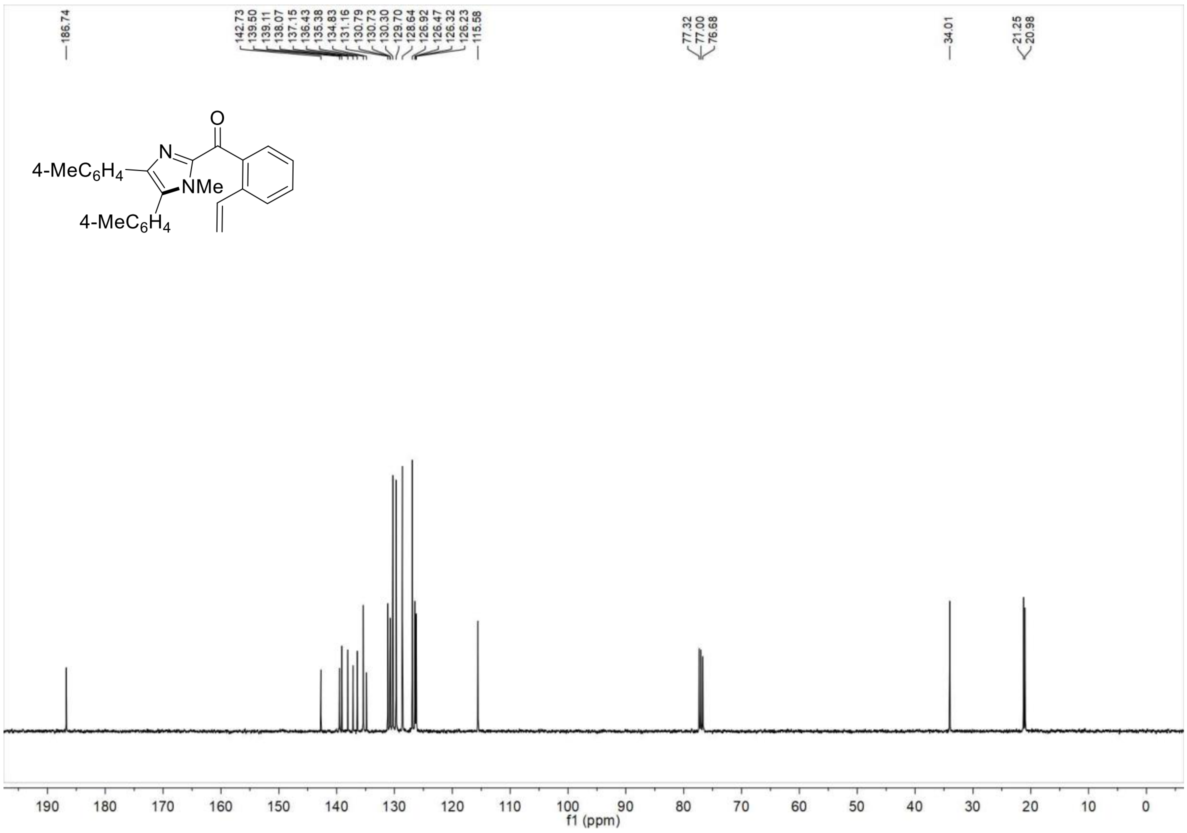

Supplementary Figure 41. ^{13}C-NMR of compound**(1-methyl-4,5-di-p-tolyl-1*H*-imidazol-2-yl)(2-vinylphenyl)methanone**, recorded at 100 MHz and 25 °C in $CDCl_3$.

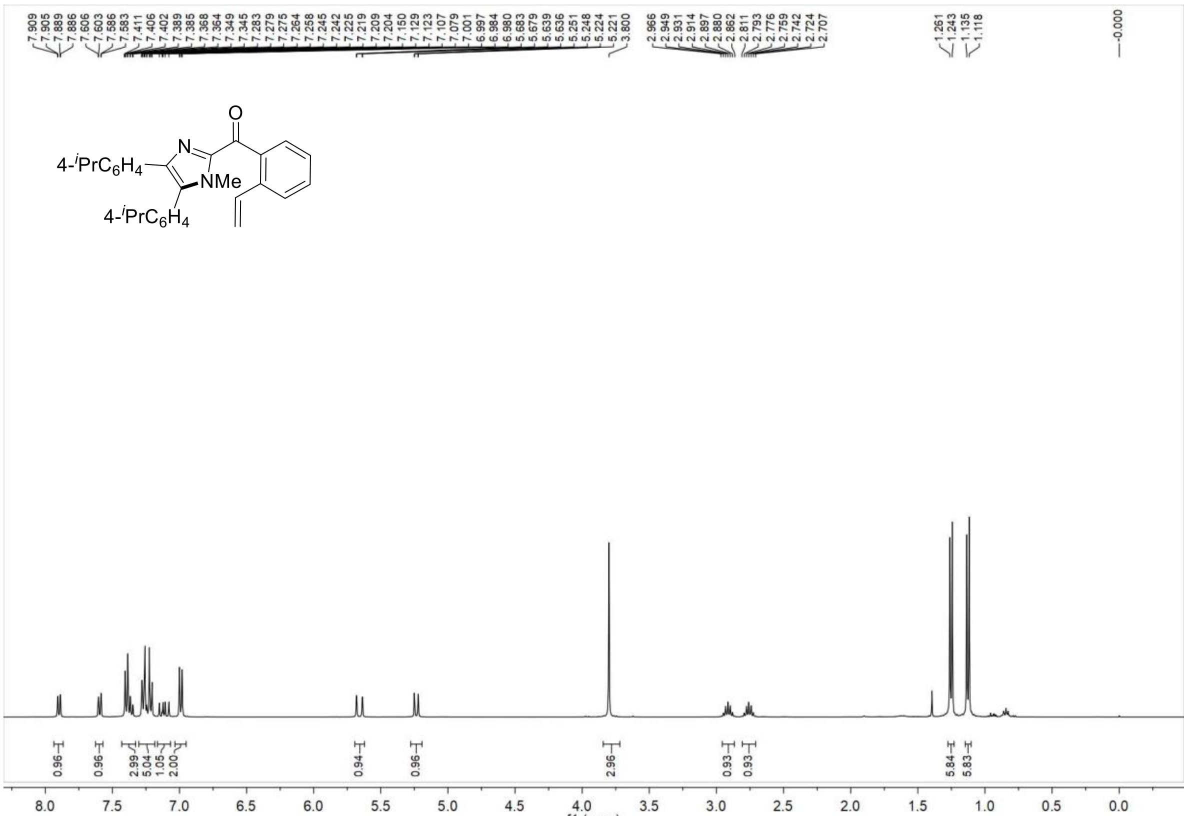

Supplementary Figure 42. ^{1}H-NMR of compound **(4,5-bis(4-isopropylphenyl)-1-methyl-1*H*-imidazol-2-yl)(2-vinylphenyl)methanone**, recorded at 400 MHz and 25 °C in $CDCl_3$.

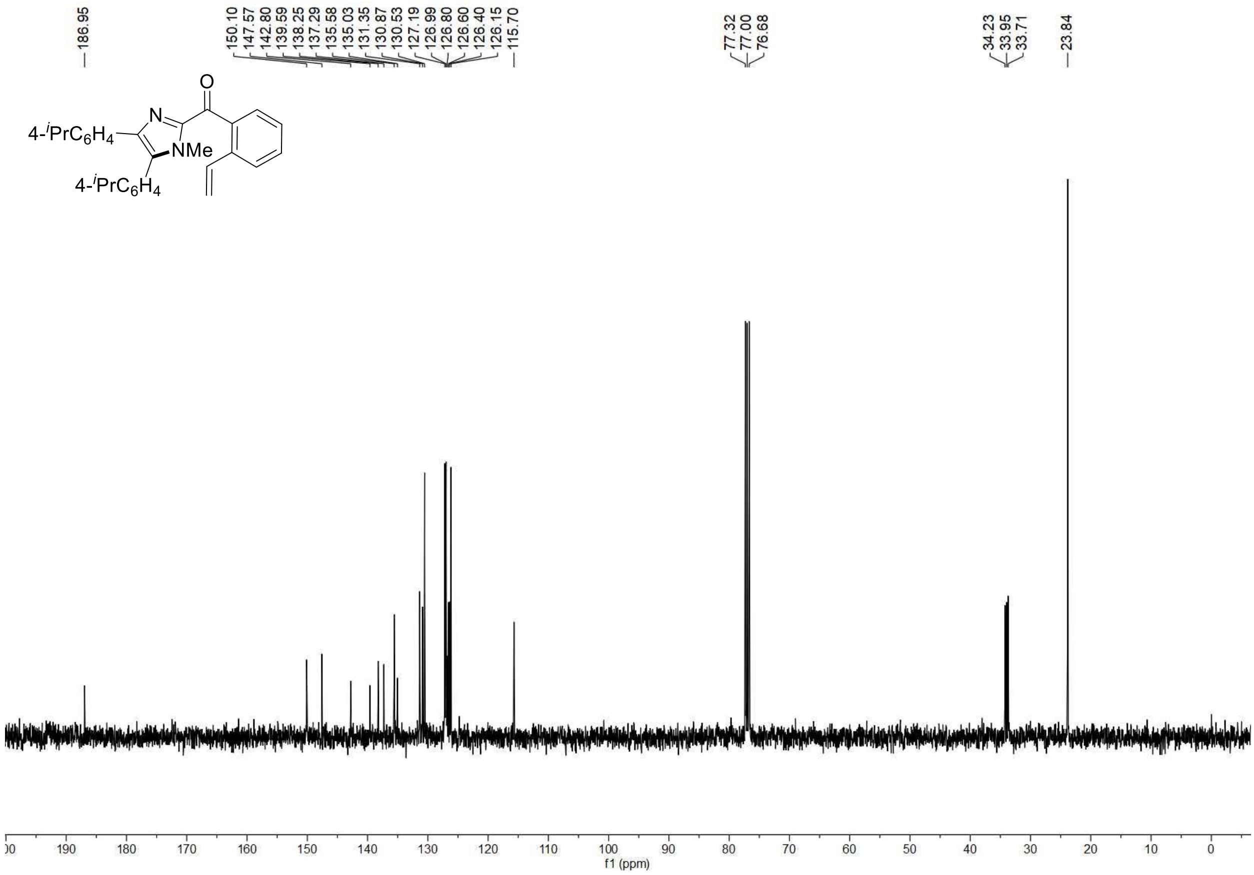

Supplementary Figure 43. ^{13}C-NMR of compound **(4,5-bis(4-isopropylphenyl)-1-methyl-1*H*-imidazol-2-yl)(2-vinylphenyl)methanone**, recorded at 100 MHz and 25 °C in $CDCl_3$.

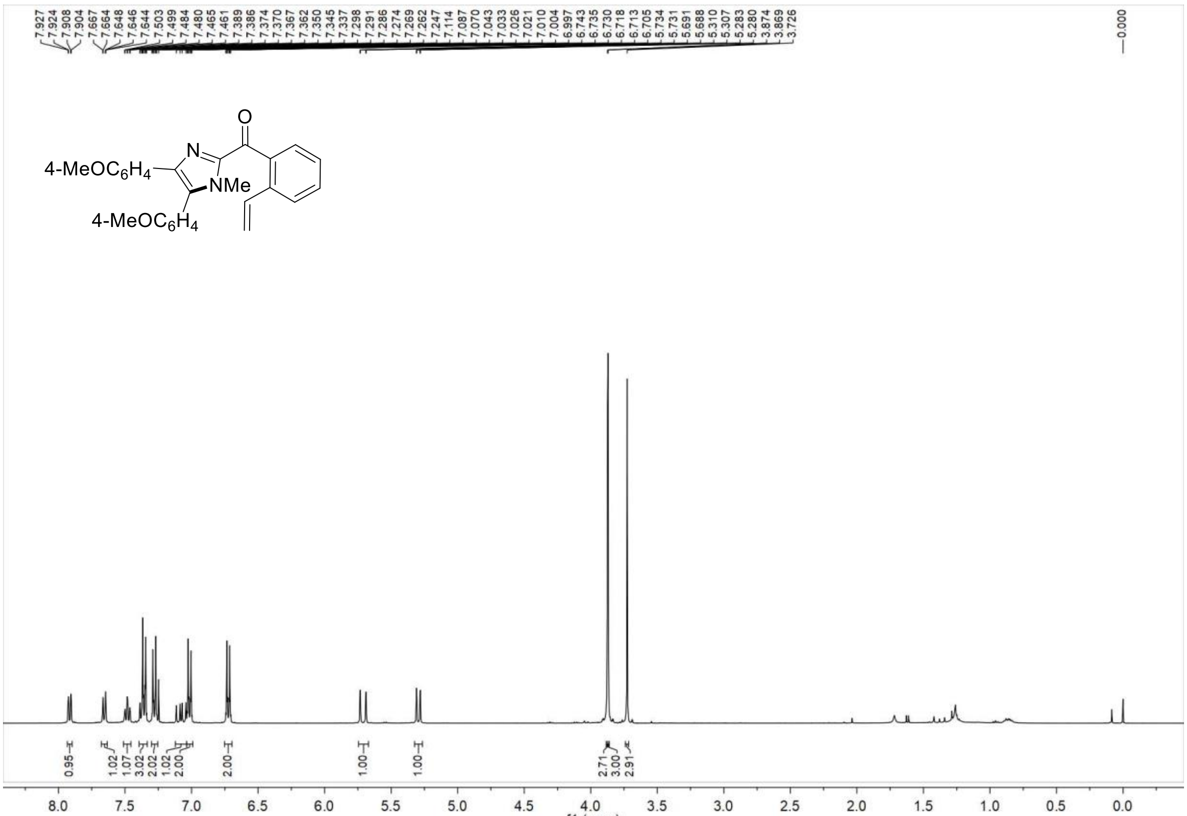

Supplementary Figure 44. ^{1}H-NMR of compound **(4,5-bis(4-methoxyphenyl)-1-methyl-1*H*-imidazol-2-yl)(2-vinylphenyl)methanone**, recorded at 400 MHz and 25 °C in $CDCl_3$.

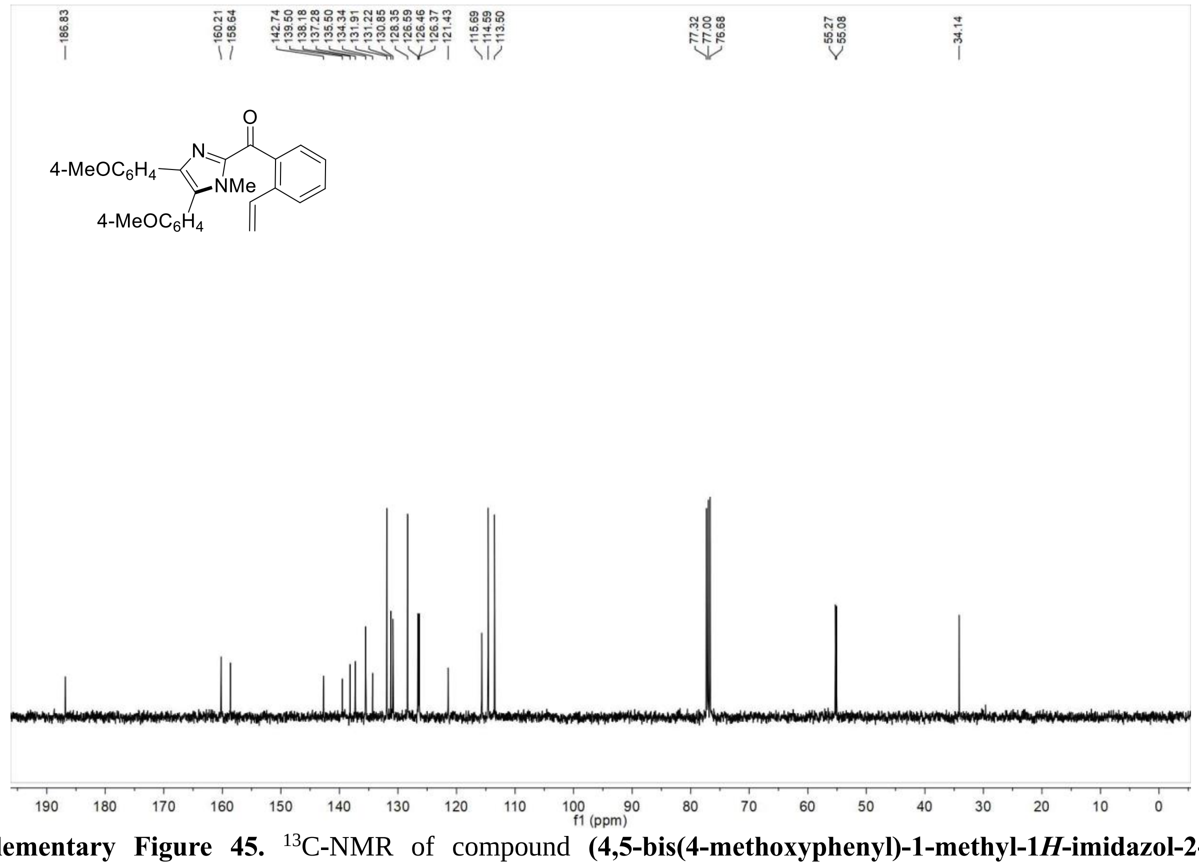

Supplementary Figure 45. ^{13}C-NMR of compound **(4,5-bis(4-methoxyphenyl)-1-methyl-1*H*-imidazol-2-yl)(2-vinylphenyl)methanone**, recorded at 100 MHz and 25 °C in $CDCl_3$.

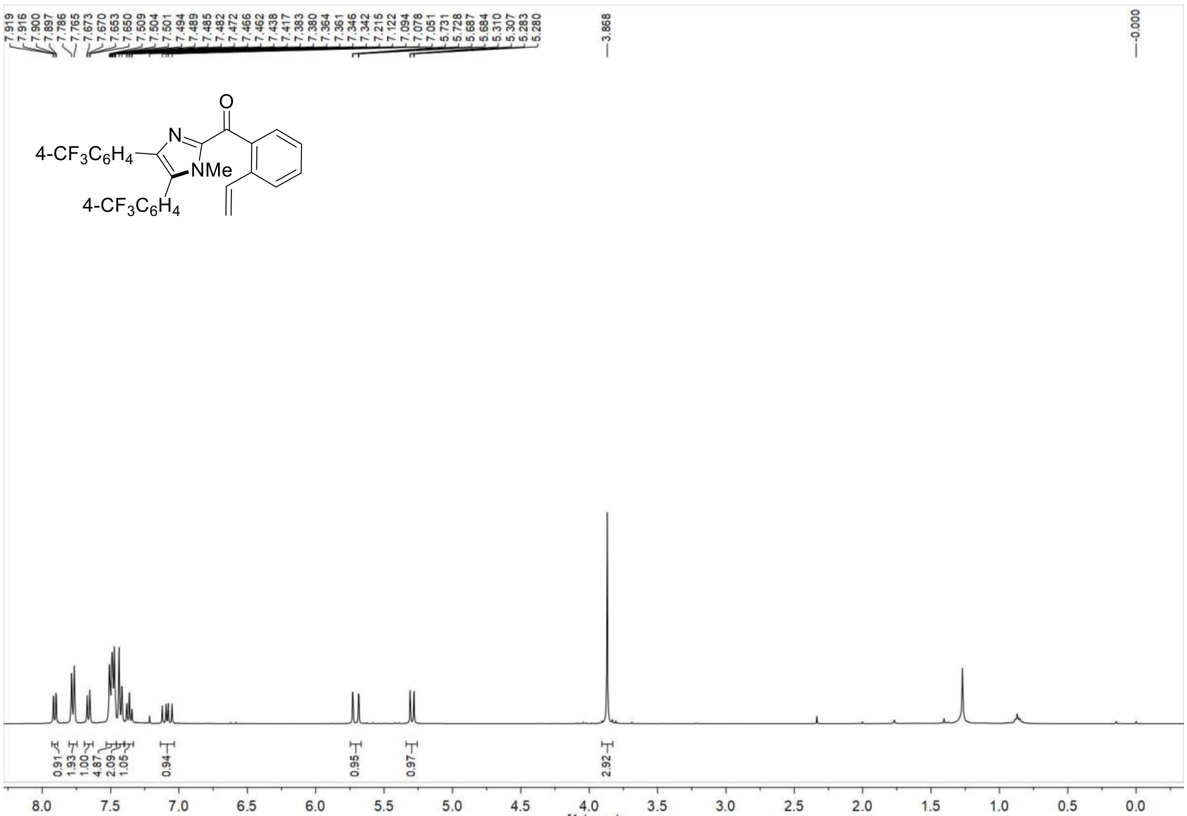

Supplementary Figure 46. ^{1}H-NMR of compound **(1-methyl-4,5-bis(4-(trifluoromethyl)phenyl)-1*H*-imidazol-2-yl)(2-vinylphenyl)methanone**, recorded at 400 MHz and 25 °C in $CDCl_3$.

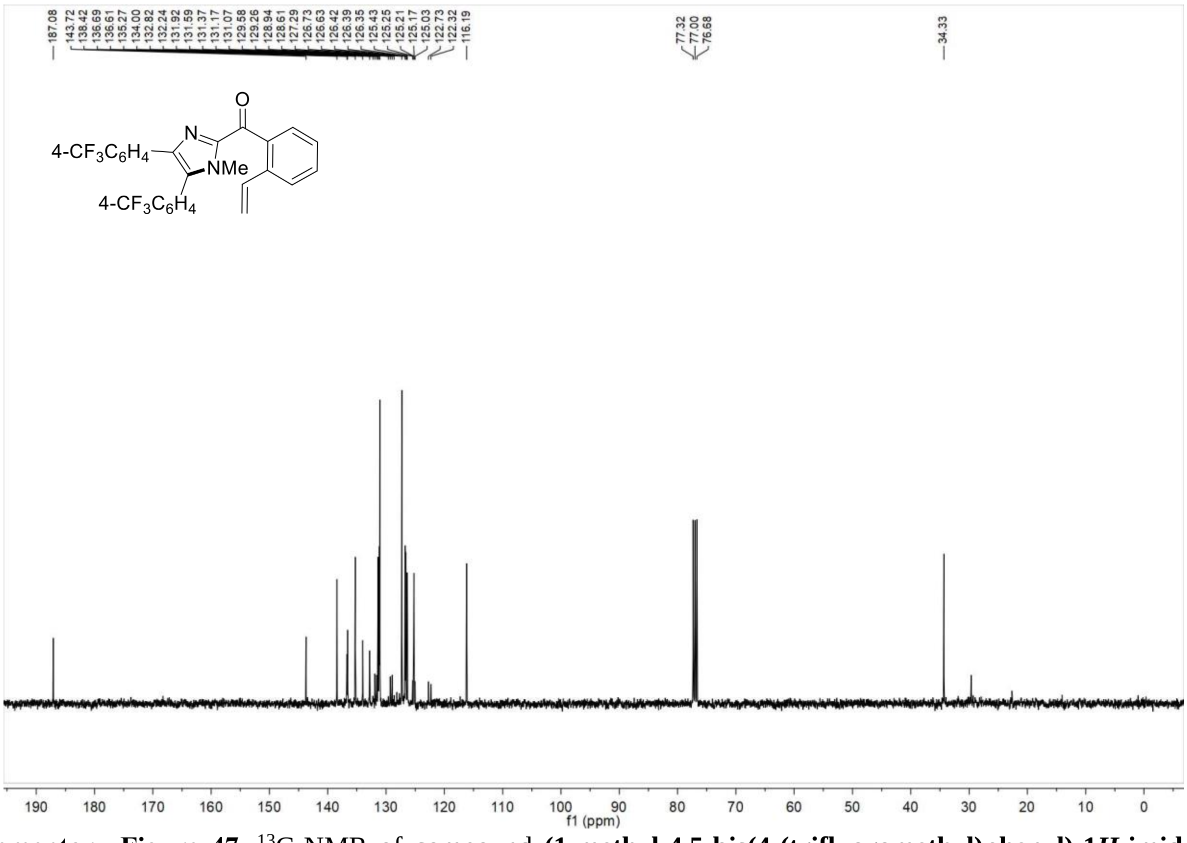

Supplementary Figure 47. ^{13}C-NMR of compound **(1-methyl-4,5-bis(4-(trifluoromethyl)phenyl)-1*H*-imidazol-2-yl)(2-vinylphenyl)methanone**, recorded at 100 MHz and 25 °C in $CDCl_3$.

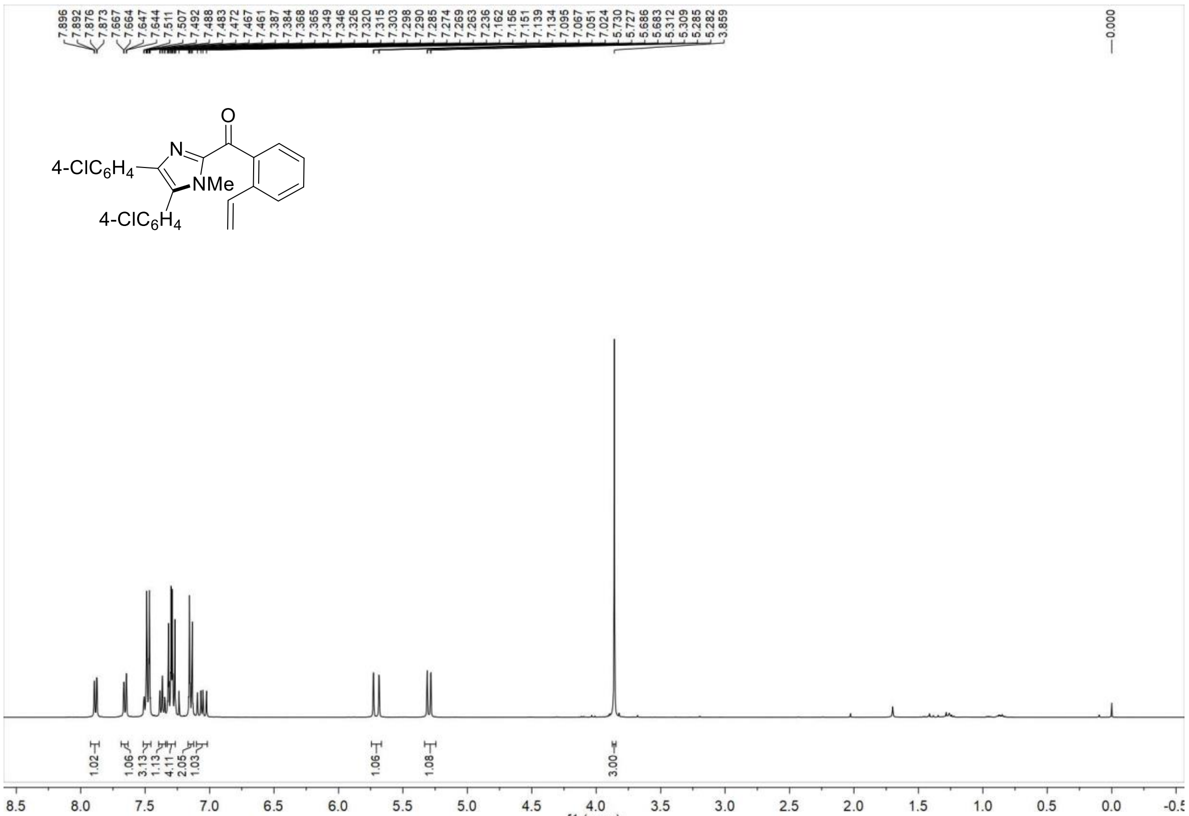

Supplementary Figure 48. ^{1}H-NMR of compound **(4,5-bis(4-chlorophenyl)-1-methyl-1*H*-imidazol-2-yl)(2-vinylphenyl)methanone**, recorded at 400 MHz and 25 °C in $CDCl_3$.

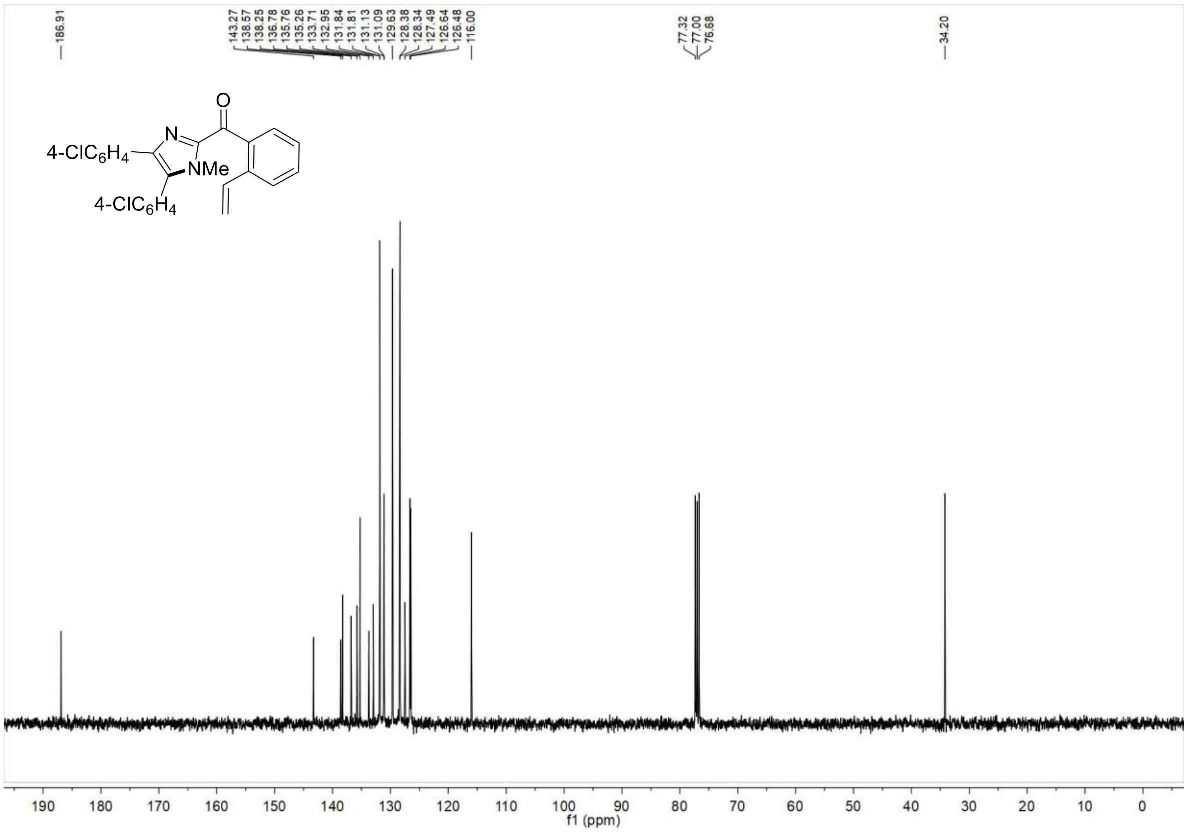

Supplementary Figure 49. ^{13}C-NMR of compound **(4,5-bis(4-chlorophenyl)-1-methyl-1*H*-imidazol-2-yl)(2-vinylphenyl)methanone**, recorded at 100 MHz and 25 °C in $CDCl_3$.

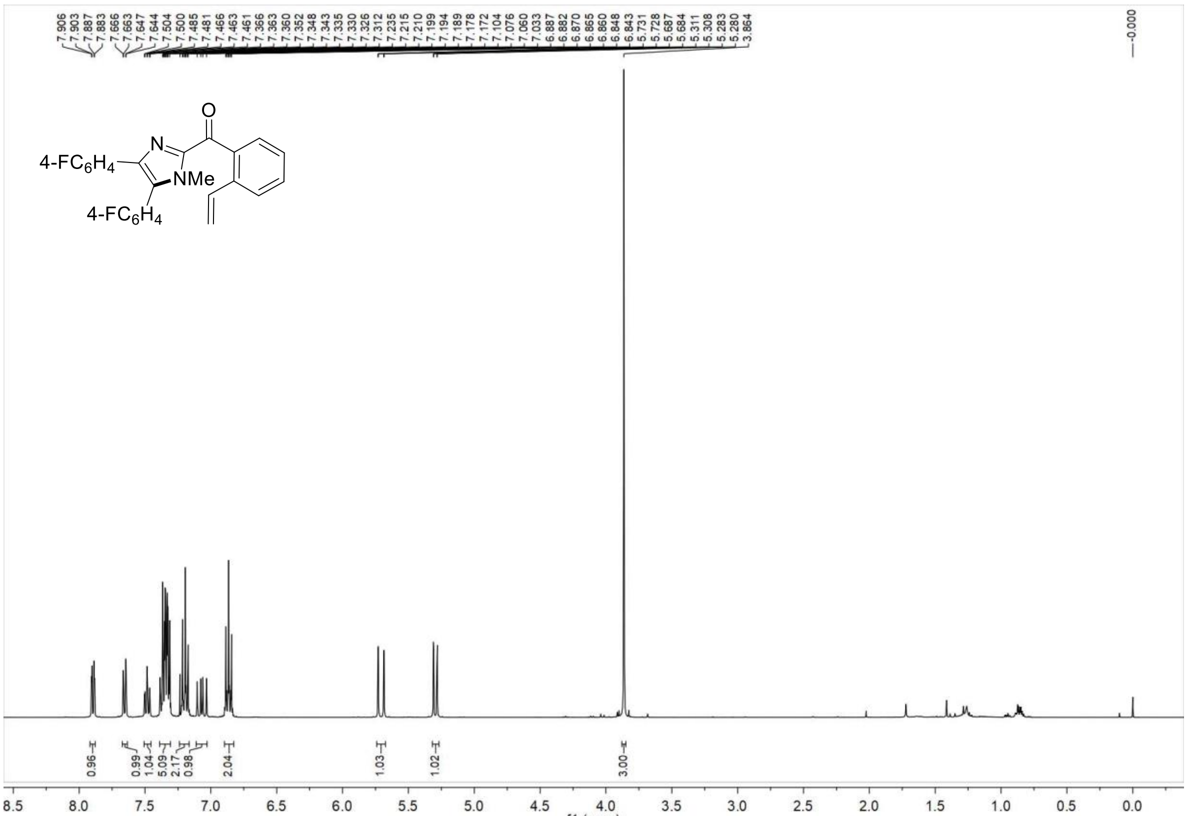

Supplementary Figure 50. ^{1}H-NMR of compound **(4,5-bis(4-fluorophenyl)-1-methyl-1*H*-imidazol-2-yl)(2-vinylphenyl)methanone**, recorded at 400 MHz and 25 °C in $CDCl_3$.

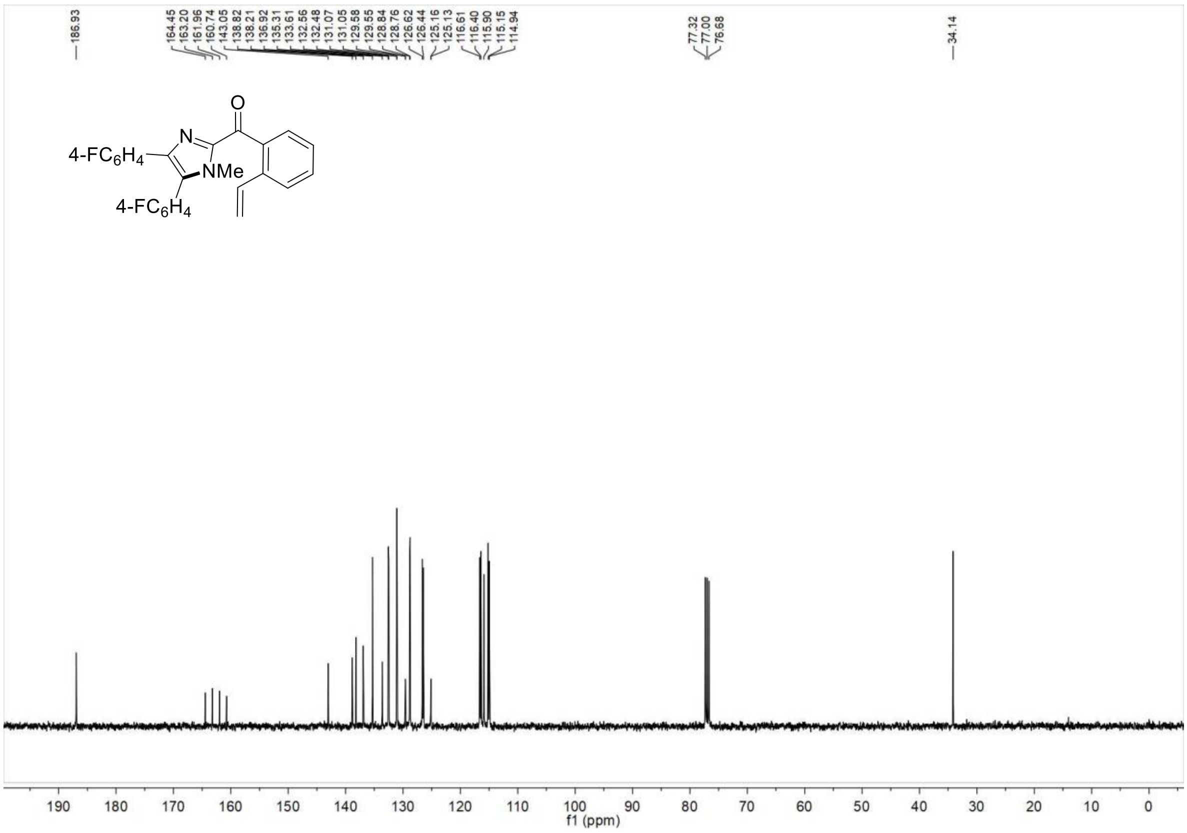

Supplementary Figure 51. ^{13}C-NMR of compound **(4,5-bis(4-fluorophenyl)-1-methyl-1*H*-imidazol-2-yl)(2-vinylphenyl)methanone**, recorded at 100 MHz and 25 °C in $CDCl_3$.

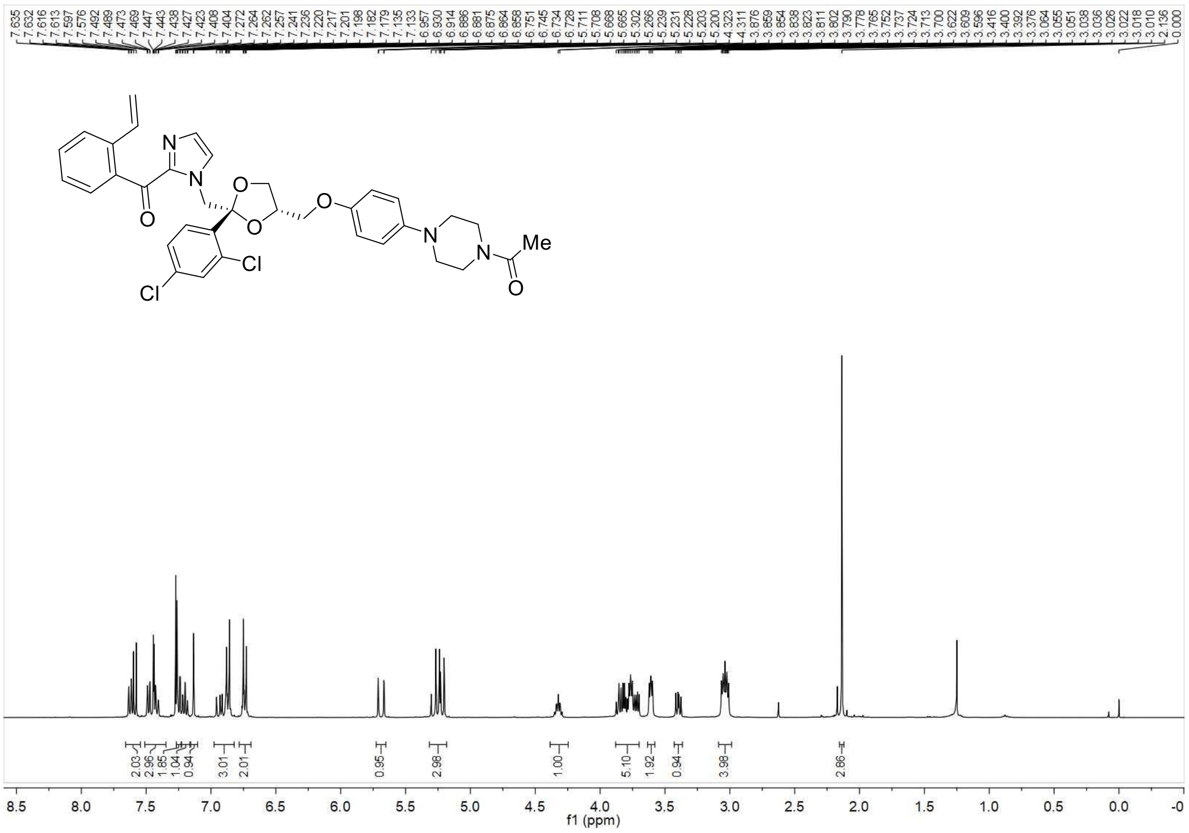

Supplementary Figure 52. ^{1}H-NMR of compound **Ketone derived from Ketoconazole**, recorded at 400 MHz and 25 °C in $CDCl_3$.

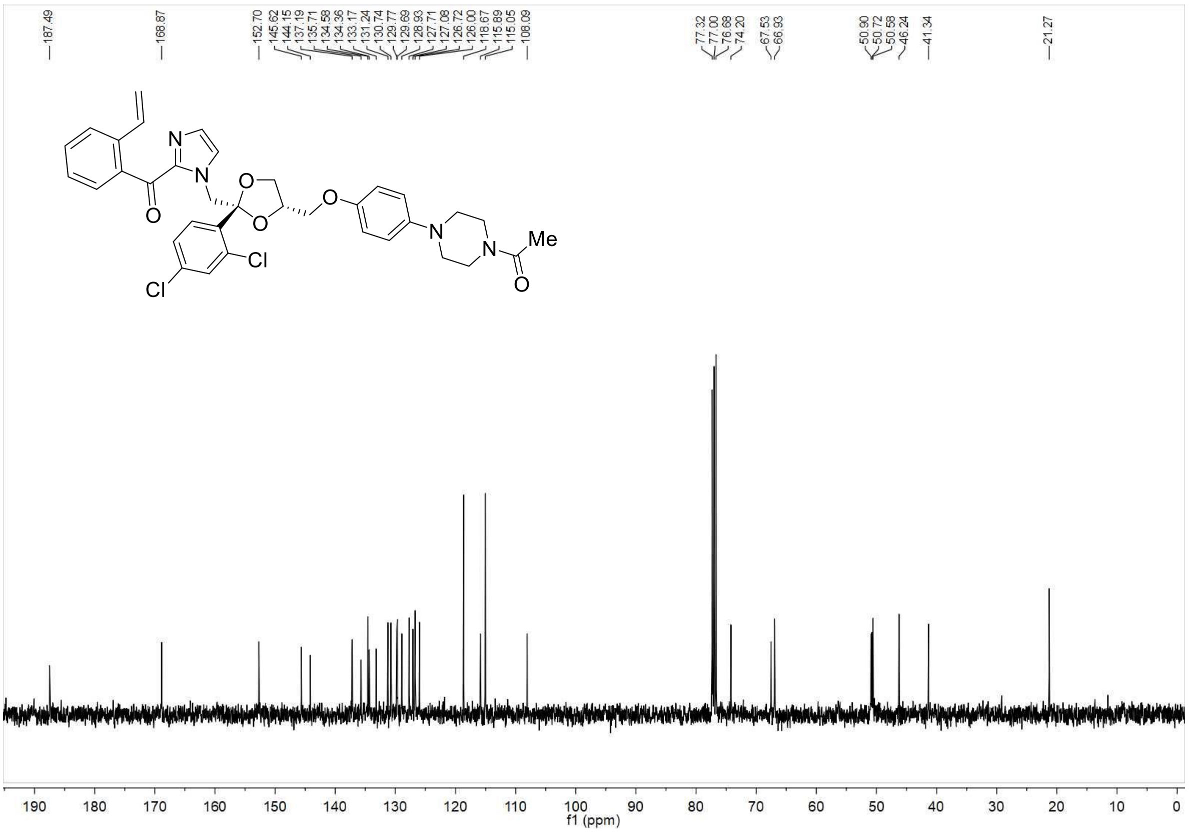

Supplementary Figure 53. ^{13}C-NMR of compound **Ketone derived from Ketoconazole**, recorded at 100 MHz and 25 °C in $CDCl_3$.

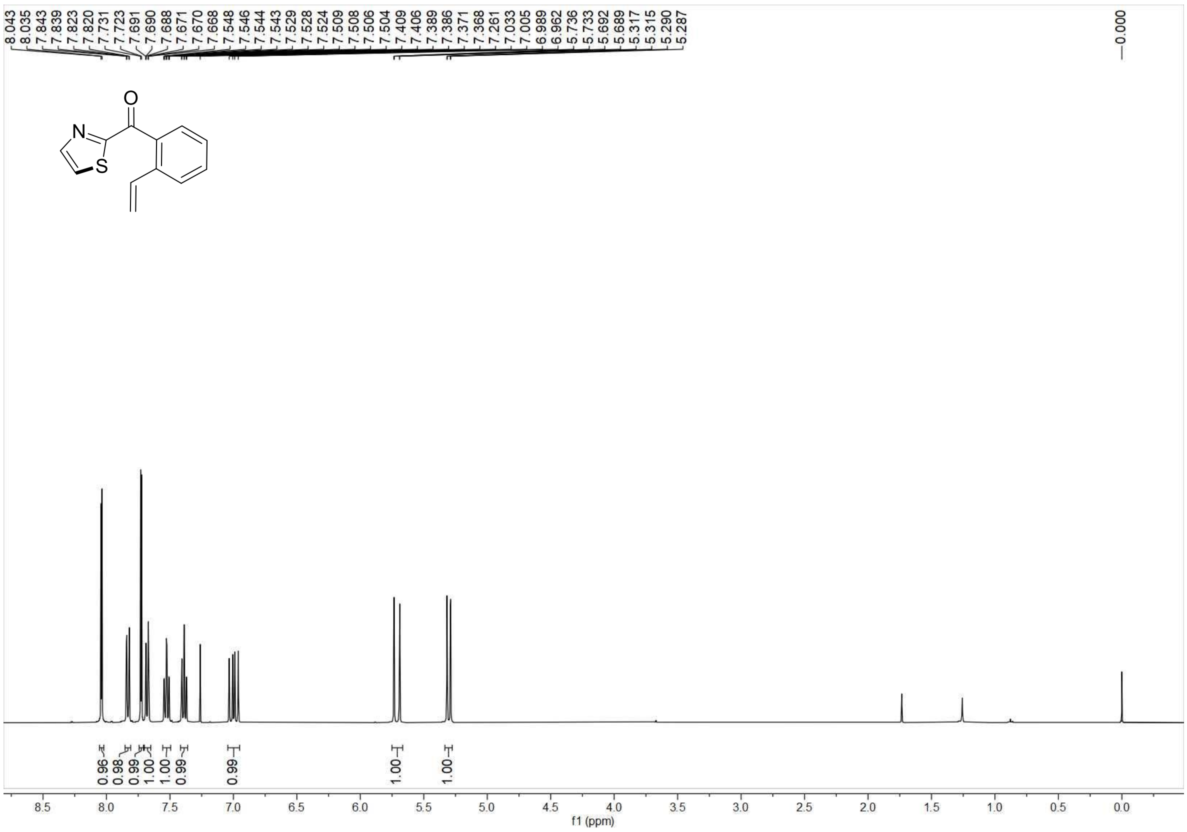

Supplementary Figure 54. ^{1}H-NMR of compound **thiazol-2-yl(2-vinylphenyl)methanone**, recorded at 400 MHz and 25 °C in $CDCl_3$.

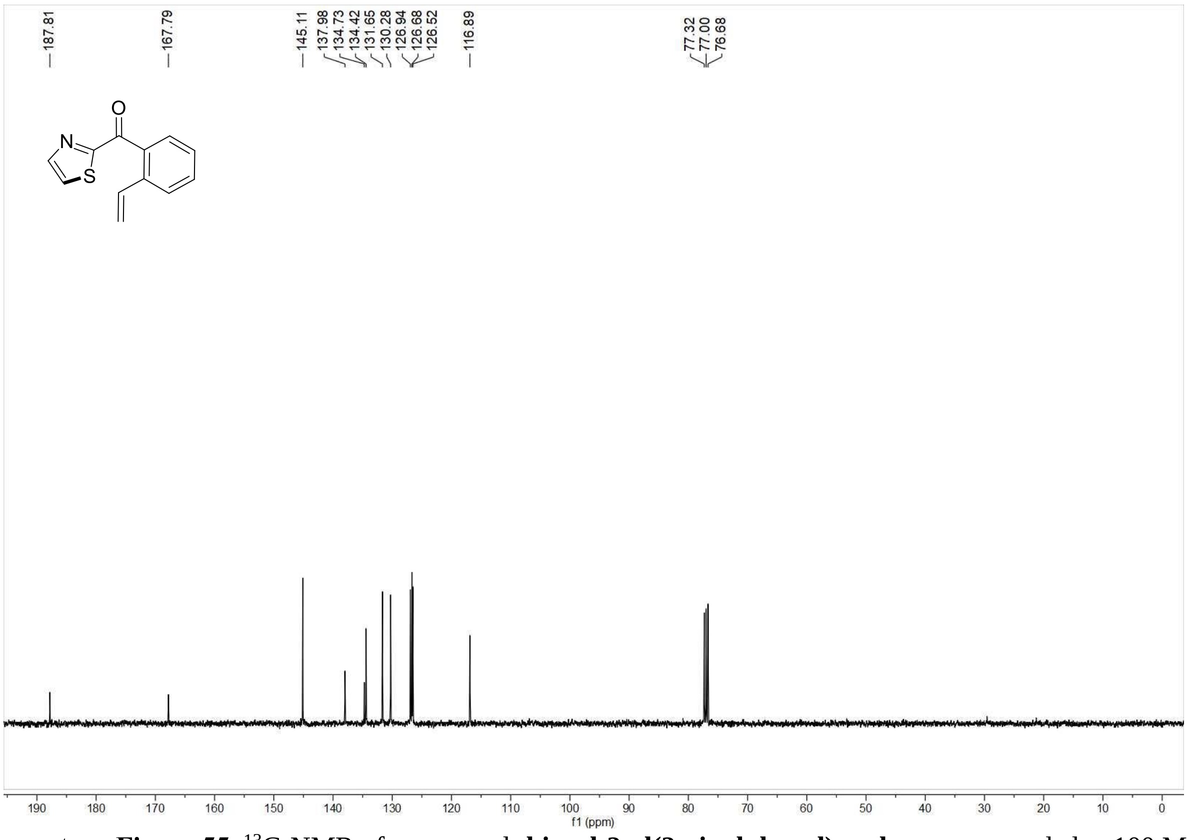

Supplementary Figure 55. ^{13}C-NMR of compound **thiazol-2-yl(2-vinylphenyl)methanone**, recorded at 100 MHz and 25 °C in $CDCl_3$.

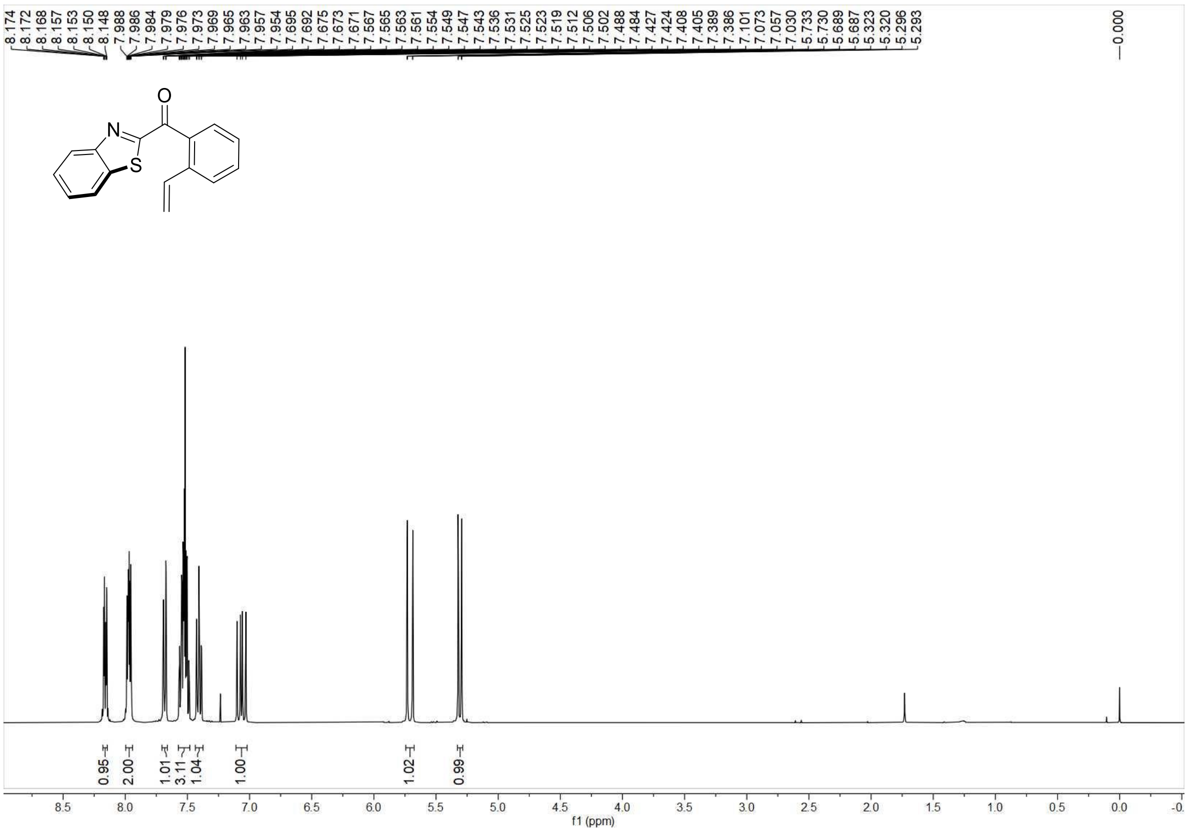

Supplementary Figure 56. ^{1}H-NMR of compound **benzo[*d*]thiazol-2-yl(2-vinylphenyl)methanone**, recorded at 400 MHz and 25 °C in $CDCl_3$.

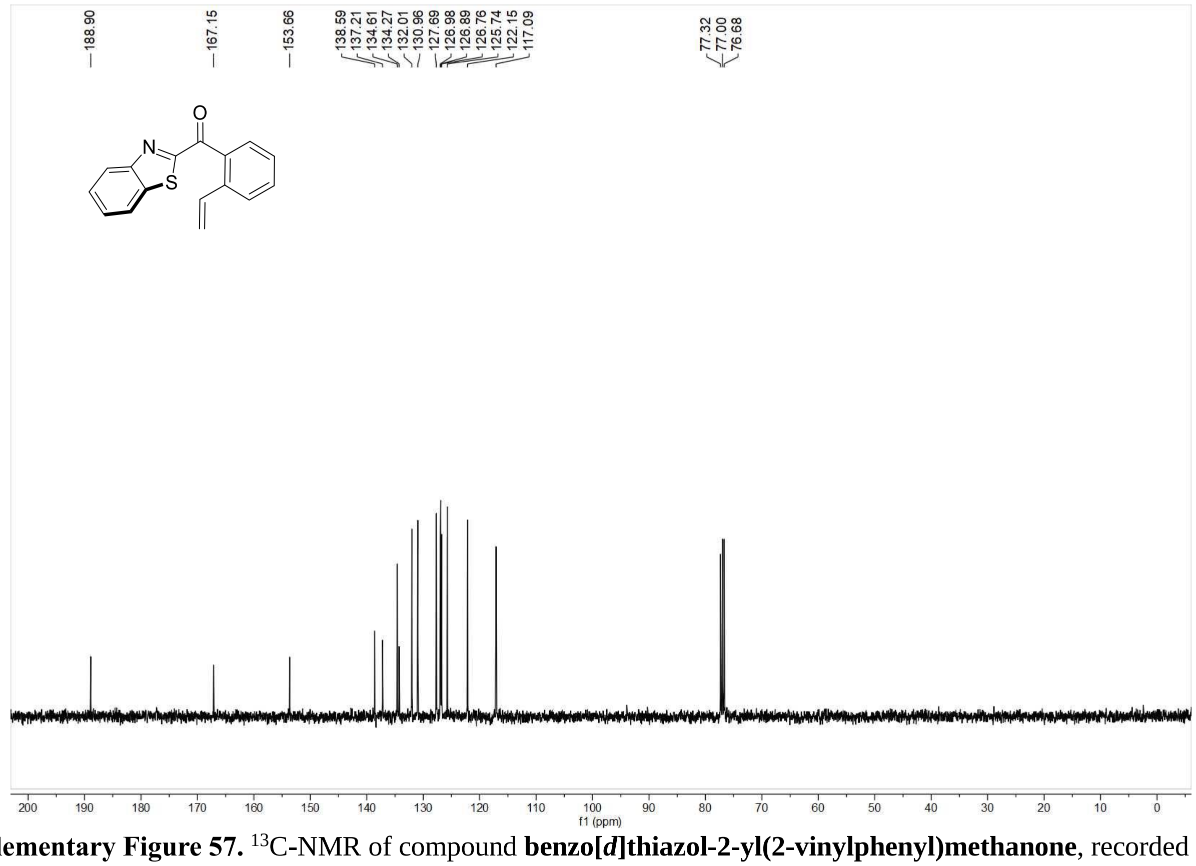

Supplementary Figure 57. ^{13}C-NMR of compound **benzo[*d*]thiazol-2-yl(2-vinylphenyl)methanone**, recorded at 100 MHz and 25 °C in $CDCl_3$.

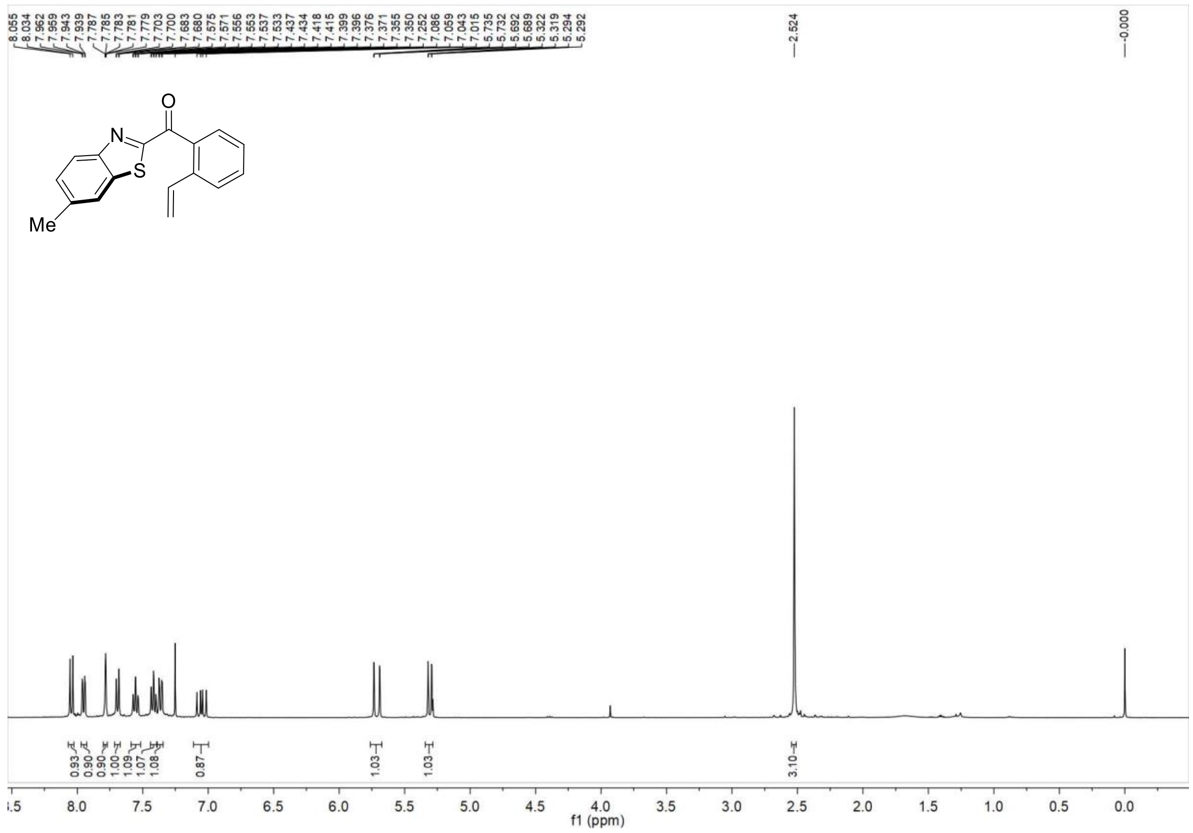

Supplementary Figure 58. ^{1}H-NMR of compound **(6-methylbenzo[*d*]thiazol-2-yl)(2-vinylphenyl)methanone**, recorded at 400 MHz and 25 °C in $CDCl_3$.

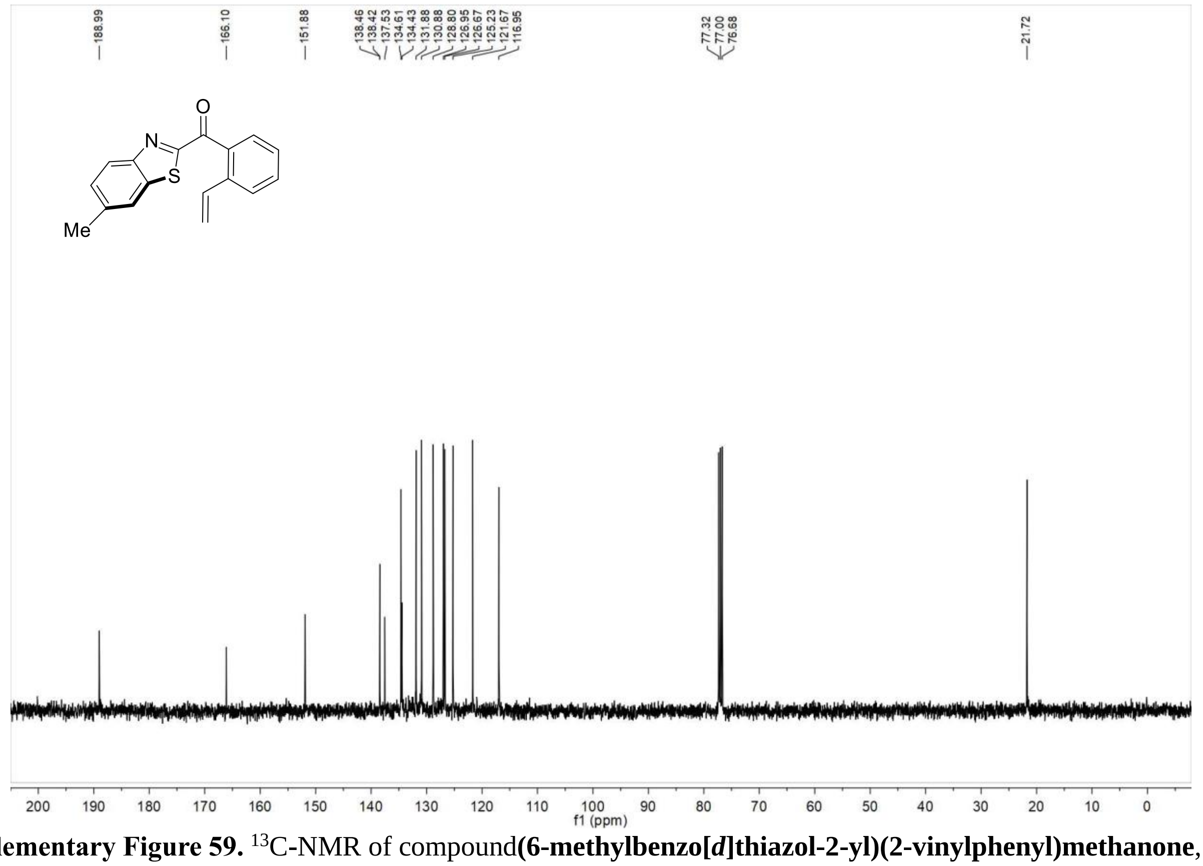

Supplementary Figure 59. ^{13}C-NMR of compound**(6-methylbenzo[*d*]thiazol-2-yl)(2-vinylphenyl)methanone**, recorded at 100 MHz and 25 °C in $CDCl_3$.

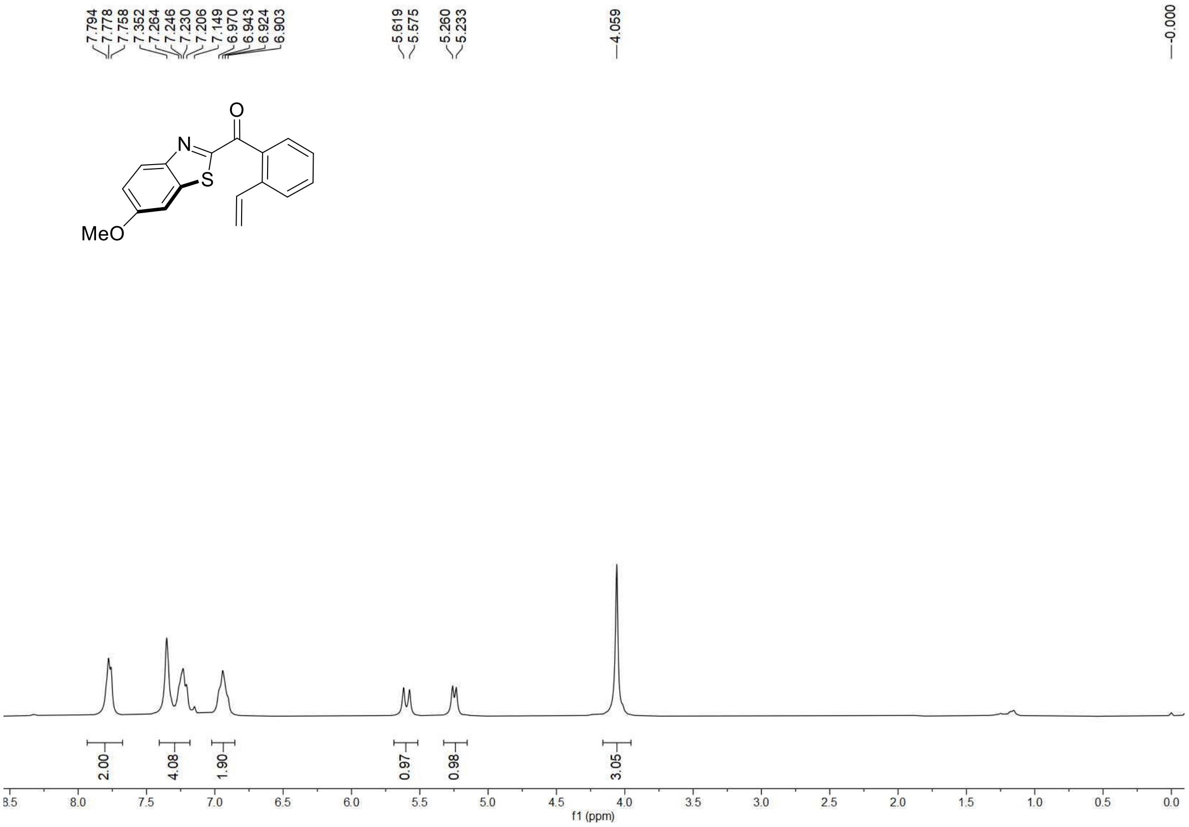

Supplementary Figure 60. ^{1}H-NMR of compound **(6-methoxybenzo[*d*]thiazol-2-yl)(2-vinylphenyl)methanone**, recorded at 400 MHz and 25 °C in $CDCl_3$.

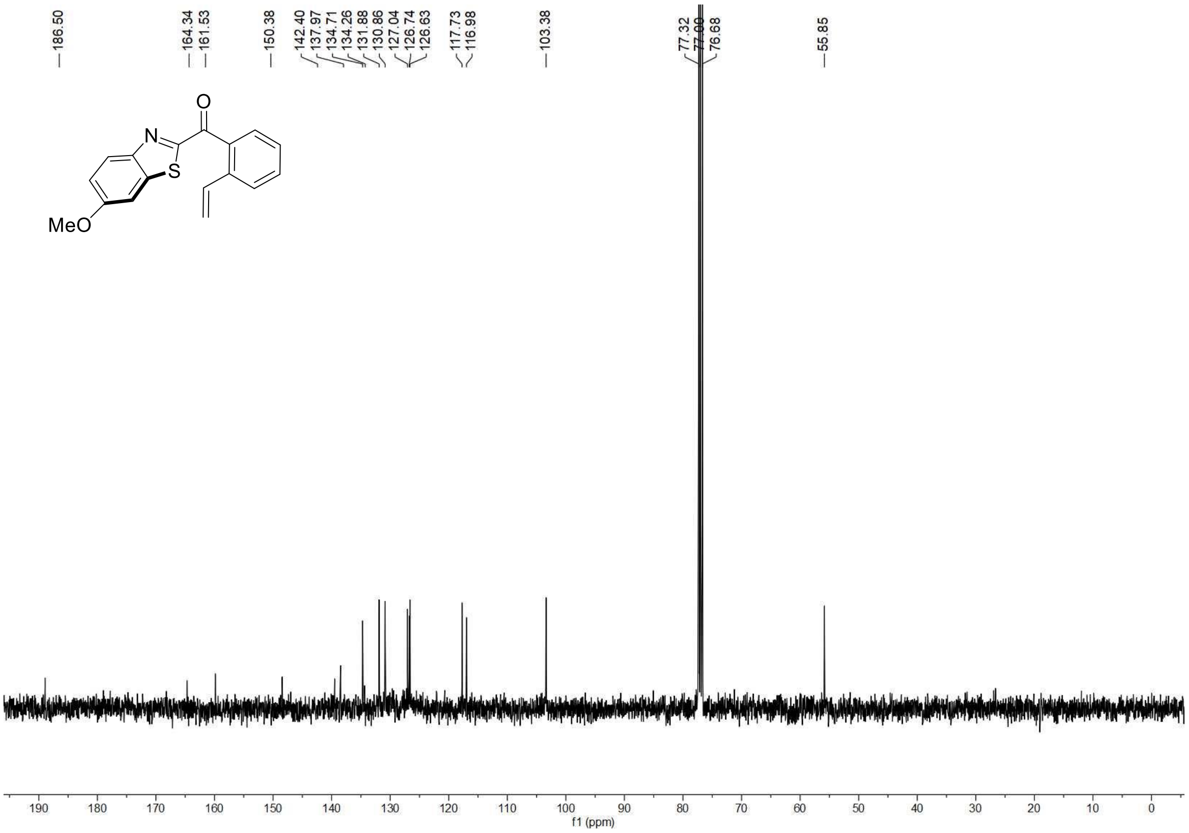

Supplementary Figure 61. ^{13}C-NMR of compound **(6-methoxybenzo[*d*]thiazol-2-yl)(2-vinylphenyl)methanone**, recorded at 100 MHz and 25 °C in $CDCl_3$.

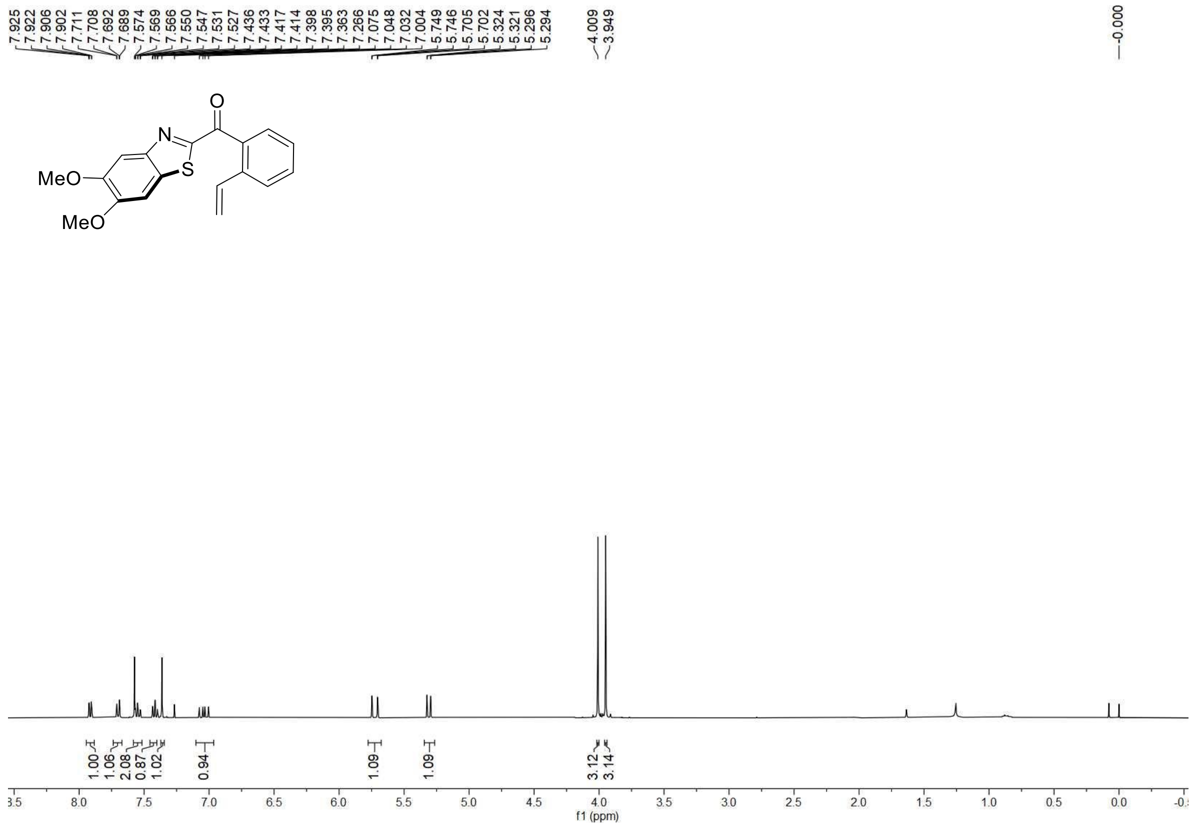

Supplementary Figure 62. ^{1}H-NMR of compound **(5,6-dimethoxybenzo[*d*]thiazol-2-yl)(2-vinylphenyl)methanone**, recorded at 400 MHz and 25 °C in $CDCl_3$.

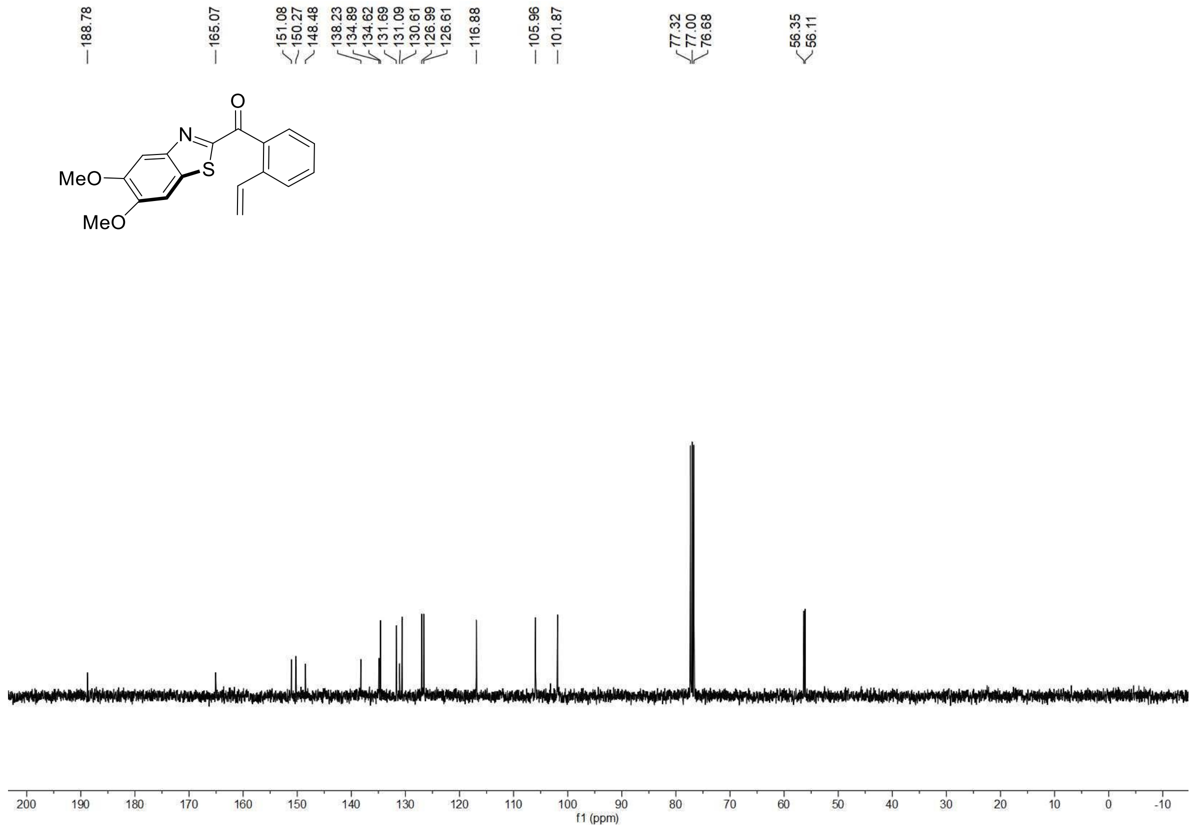

Supplementary Figure 63. ^{13}C-NMR of compound **(5,6-dimethoxybenzo[*d*]thiazol-2-yl)(2-vinylphenyl)methanone**, recorded at 100 MHz and 25 °C in $CDCl_3$.

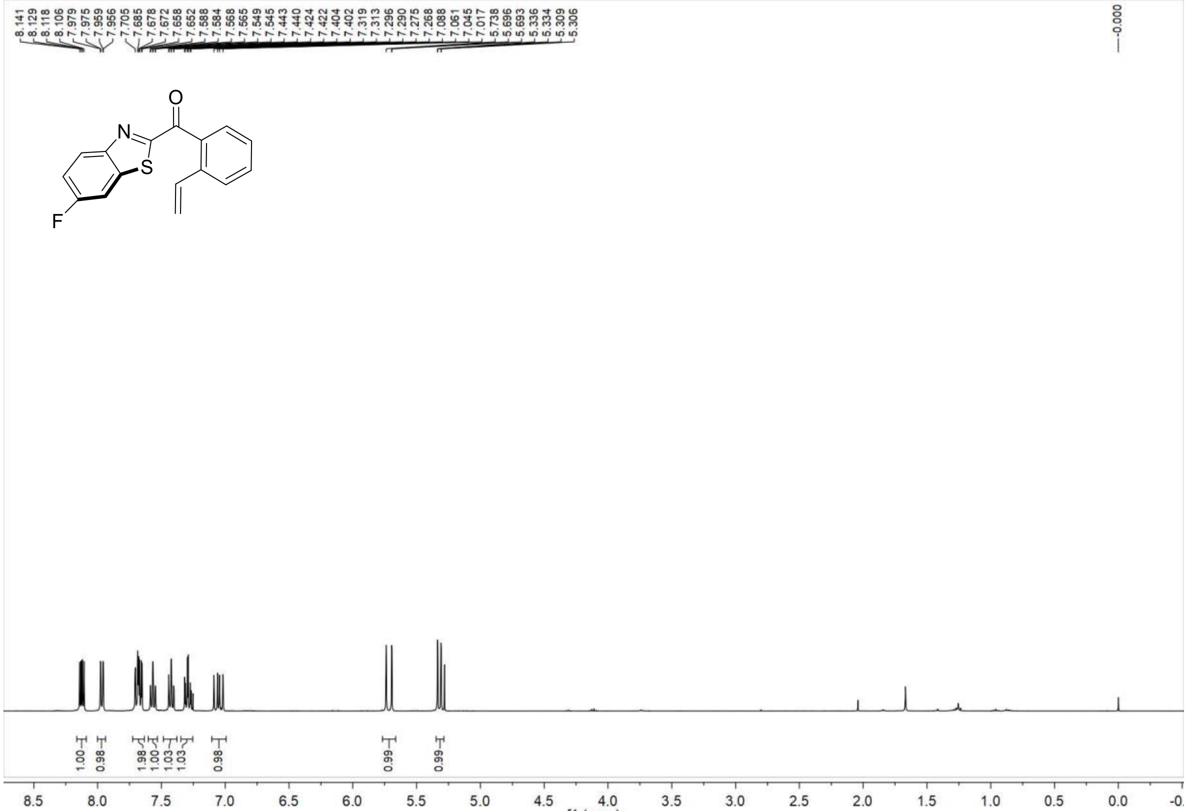

Supplementary Figure 64. ^{1}H-NMR of compound **(6-fluorobenzo[*d*]thiazol-2-yl)(2-vinylphenyl)methanone**, recorded at 400 MHz and 25 °C in $CDCl_3$.

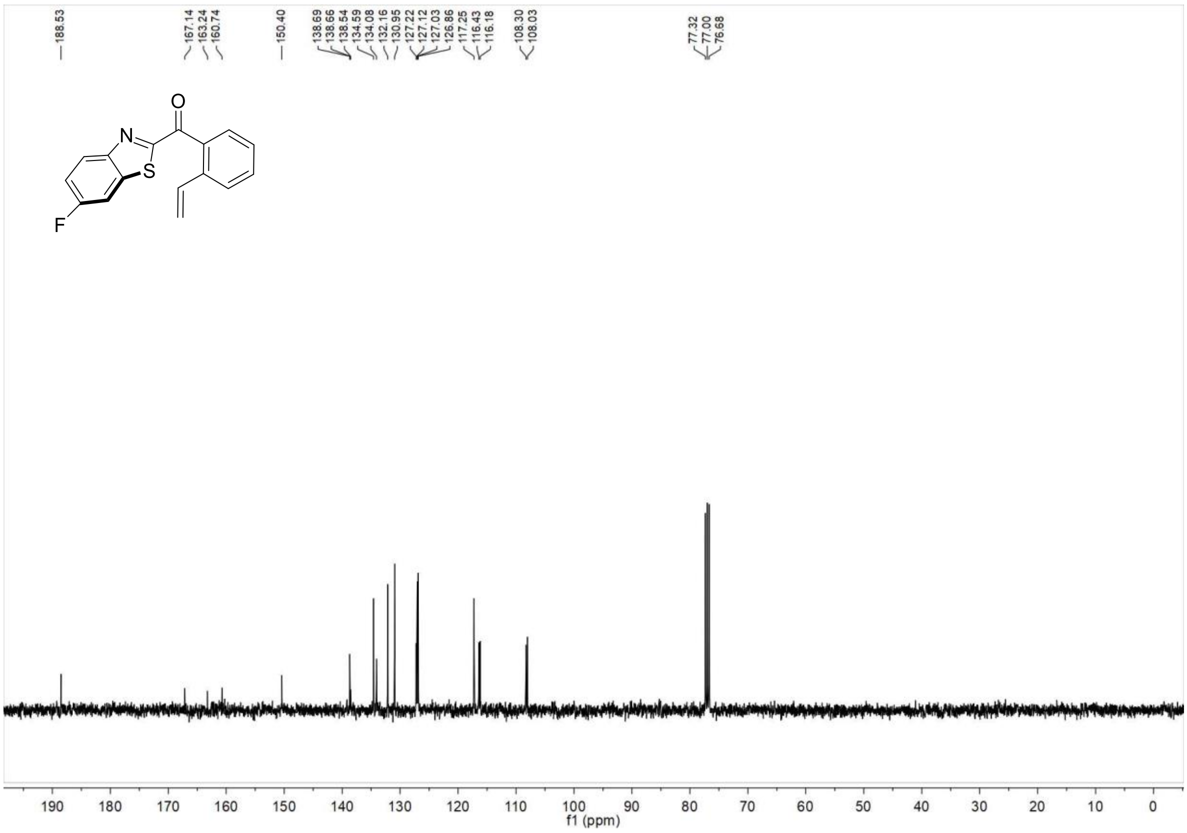

Supplementary Figure 65. ^{13}C-NMR of compound **(6-fluorobenzo[*d*]thiazol-2-yl)(2-vinylphenyl)methanone**, recorded at 100 MHz and 25 °C in $CDCl_3$.

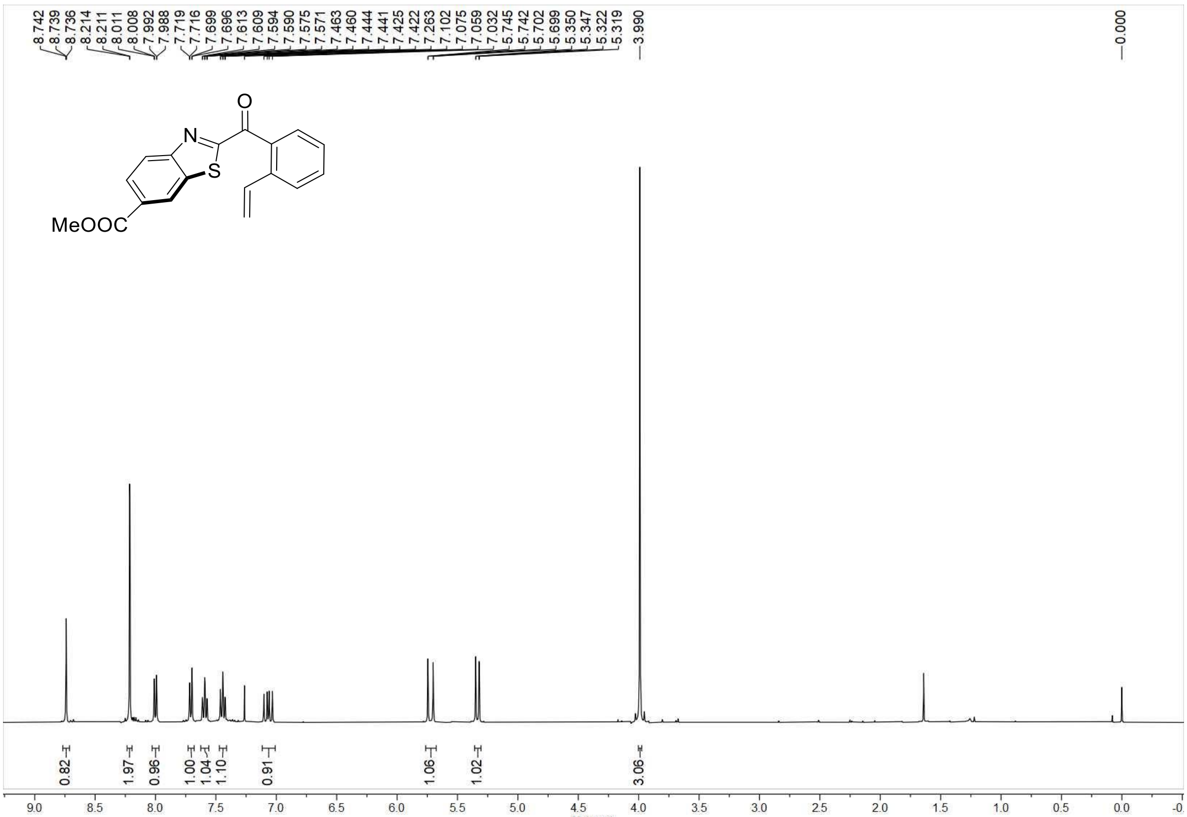

Supplementary Figure 66. ^{1}H-NMR of compound **methyl 2-(2-vinylbenzoyl)benzo[*d*]thiazole-6-carboxylate**, recorded at 400 MHz and 25 °C in $CDCl_3$.

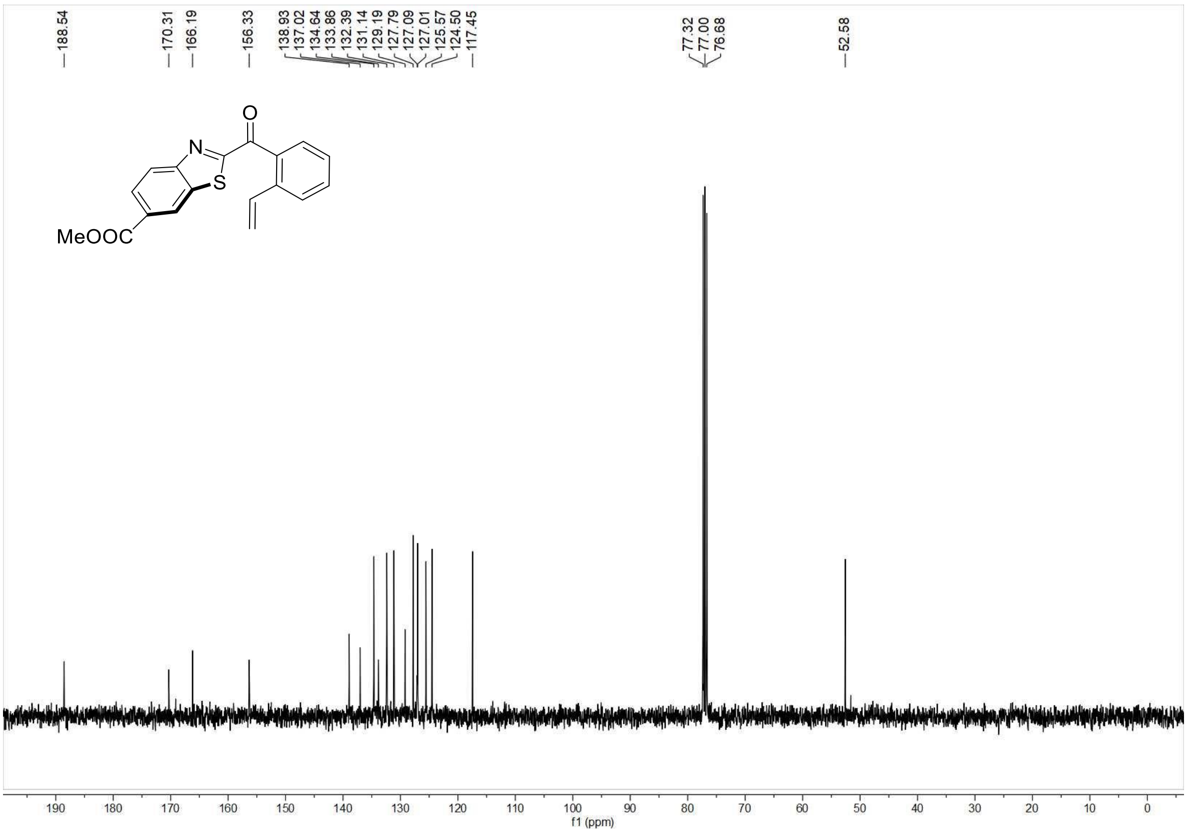

Supplementary Figure 67. ^{13}C-NMR of compound **methyl 2-(2-vinylbenzoyl)benzo[*d*]thiazole-6-carboxylate**, recorded at 100 MHz and 25 °C in $CDCl_3$.

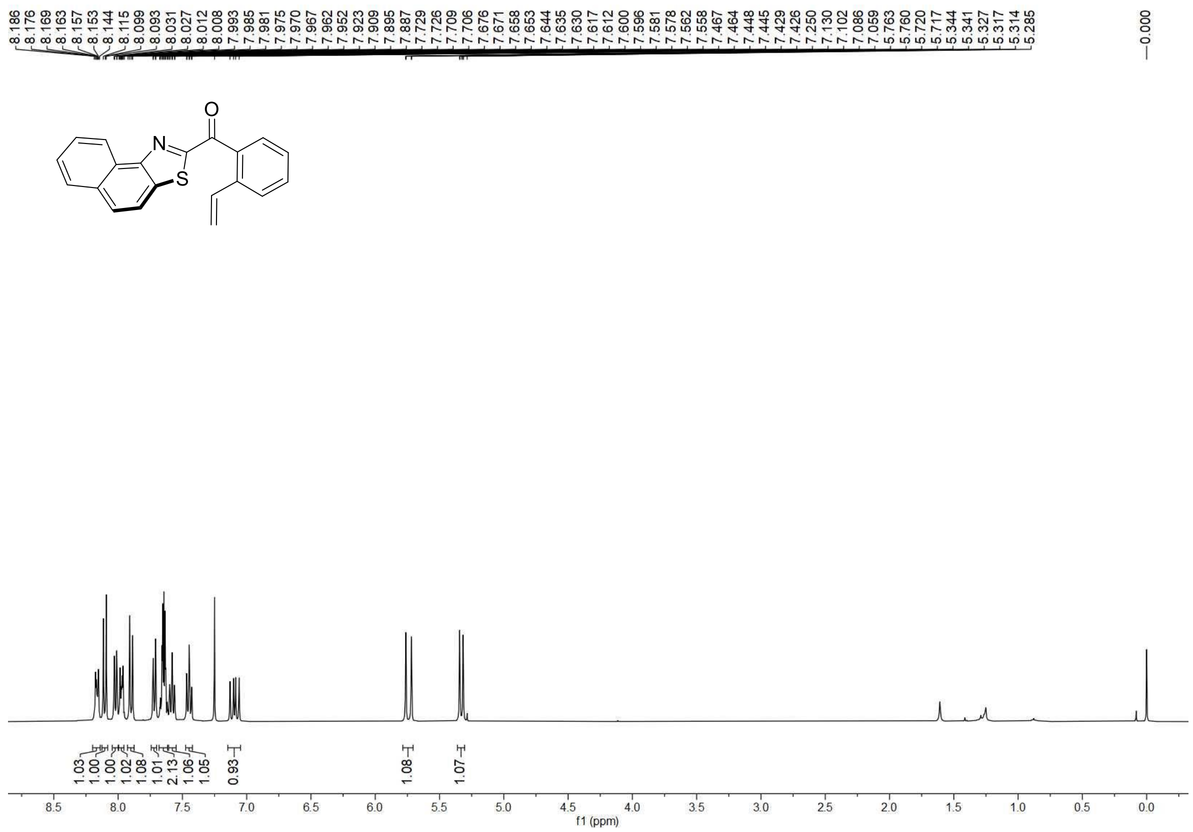

Supplementary Figure 68. ^{1}H-NMR of compound **naphtho[1,2-*d*]thiazol-2-yl(2-vinylphenyl)methanone**, recorded at 400 MHz and 25 °C in $CDCl_3$.

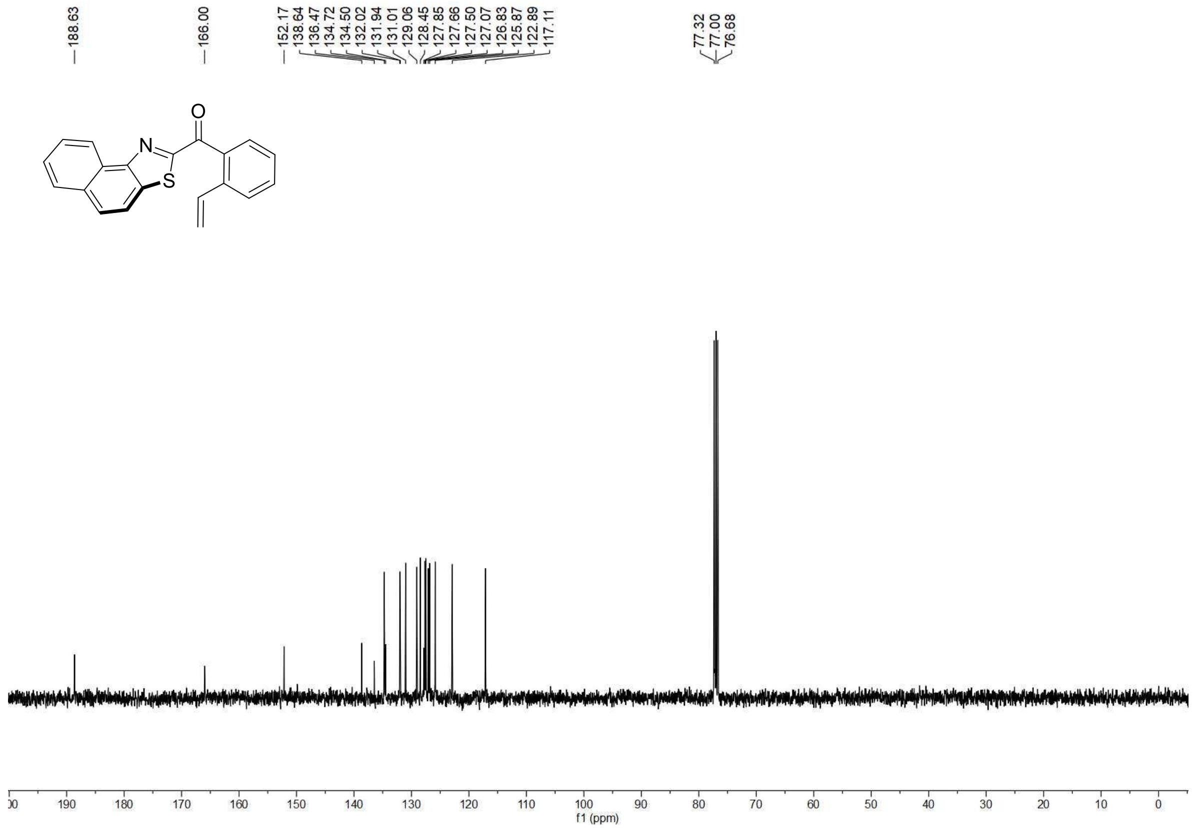

Supplementary Figure 69. ^{13}C-NMR of compound **naphtho[1,2-*d*]thiazol-2-yl(2-vinylphenyl)methanone**, recorded at 100 MHz and 25 °C in $CDCl_3$.

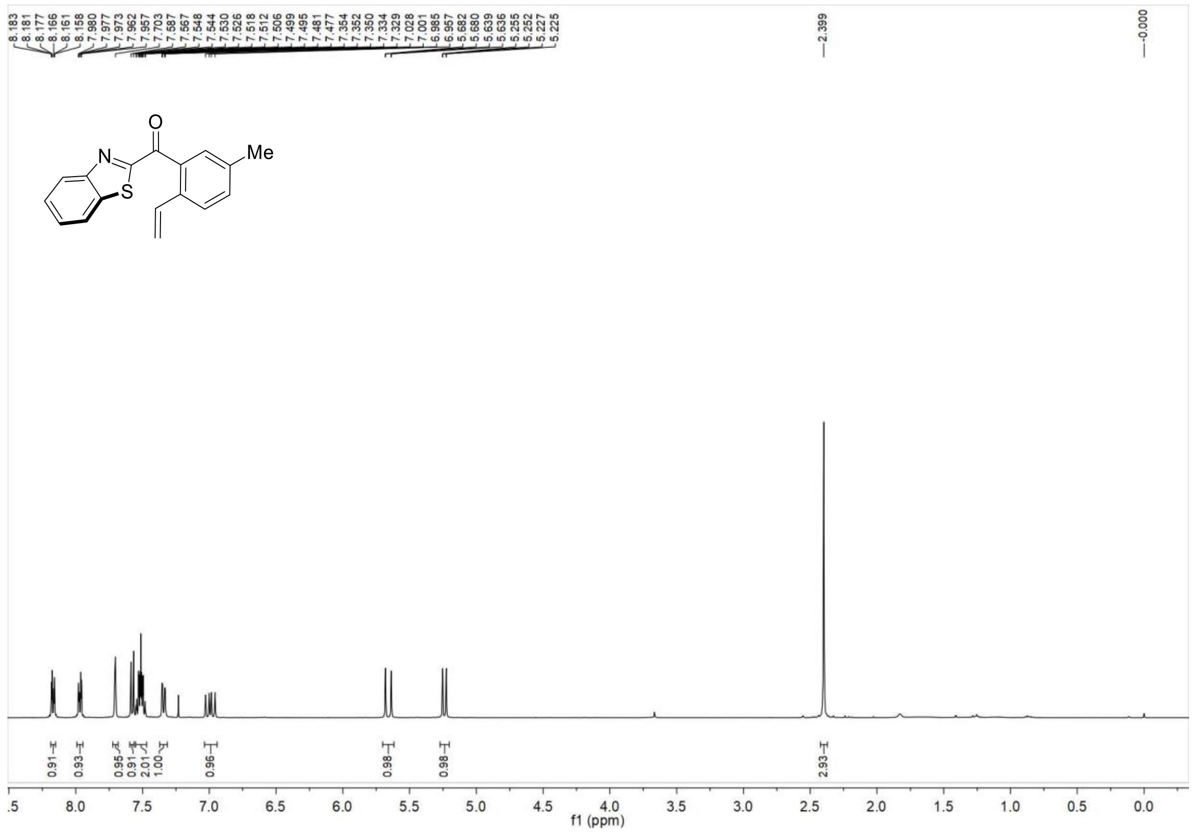

Supplementary Figure 70. ^{1}H-NMR of compound **benzo[*d*]thiazol-2-yl(5-methyl-2-vinylphenyl)methanone**, recorded at 400 MHz and 25 °C in $CDCl_3$.

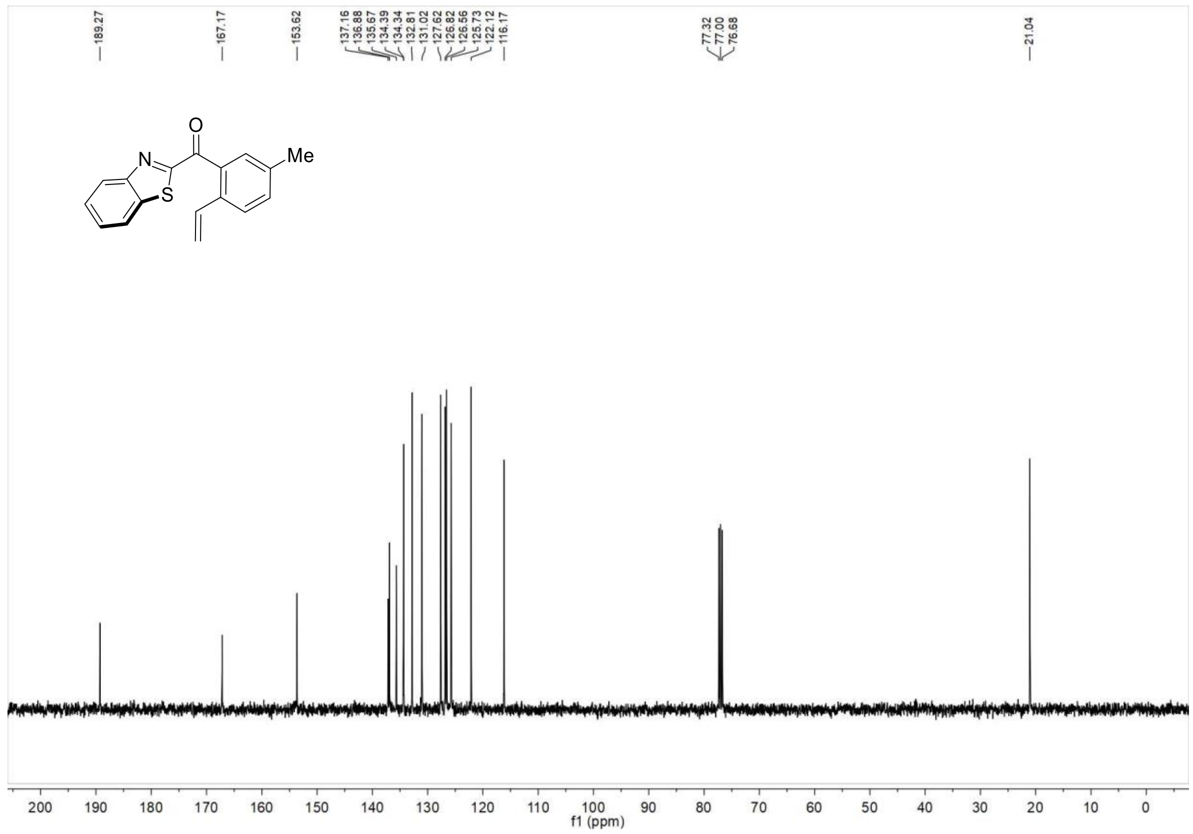

Supplementary Figure 71. ^{13}C-NMR of compound **benzo[*d*]thiazol-2-yl(5-methyl-2-vinylphenyl)methanone**, recorded at 100 MHz and 25 °C in $CDCl_3$.

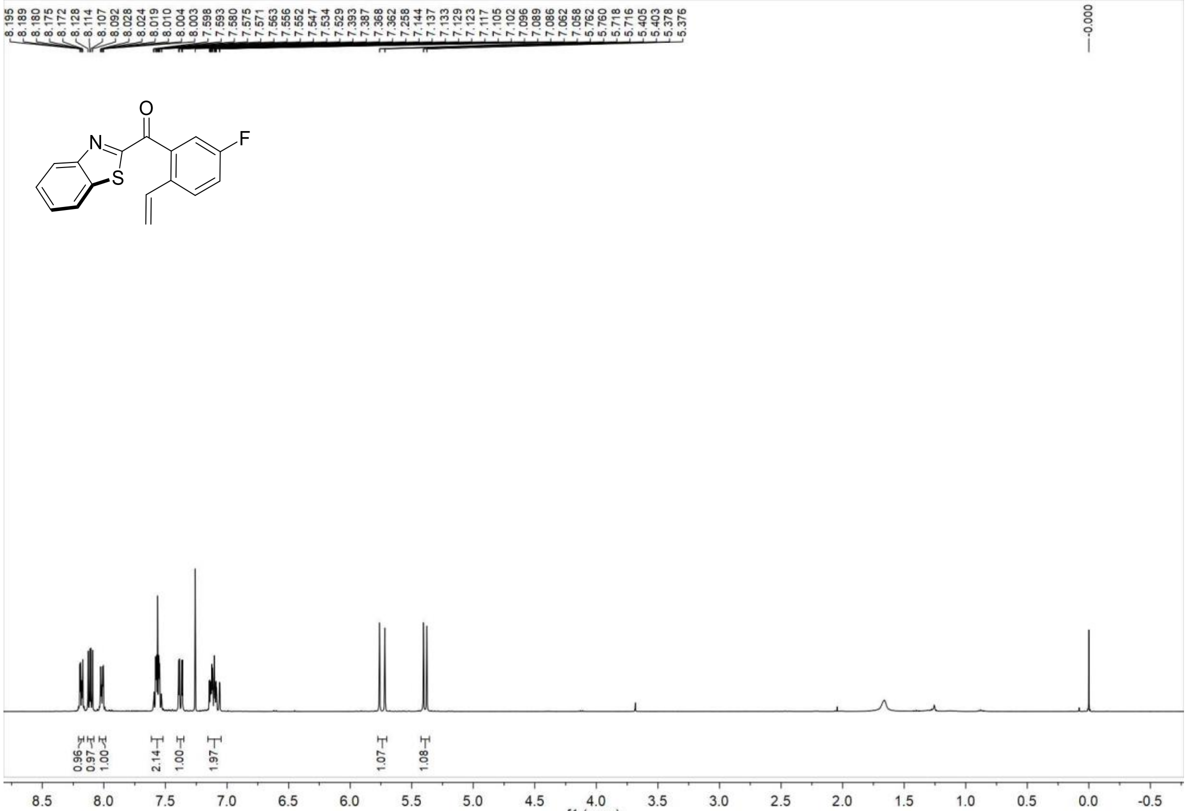

Supplementary Figure 72. ^{1}H-NMR of compound **benzo[*d*]thiazol-2-yl(5-fluoro-2-vinylphenyl)methanone**, recorded at 400 MHz and 25 °C in $CDCl_3$.

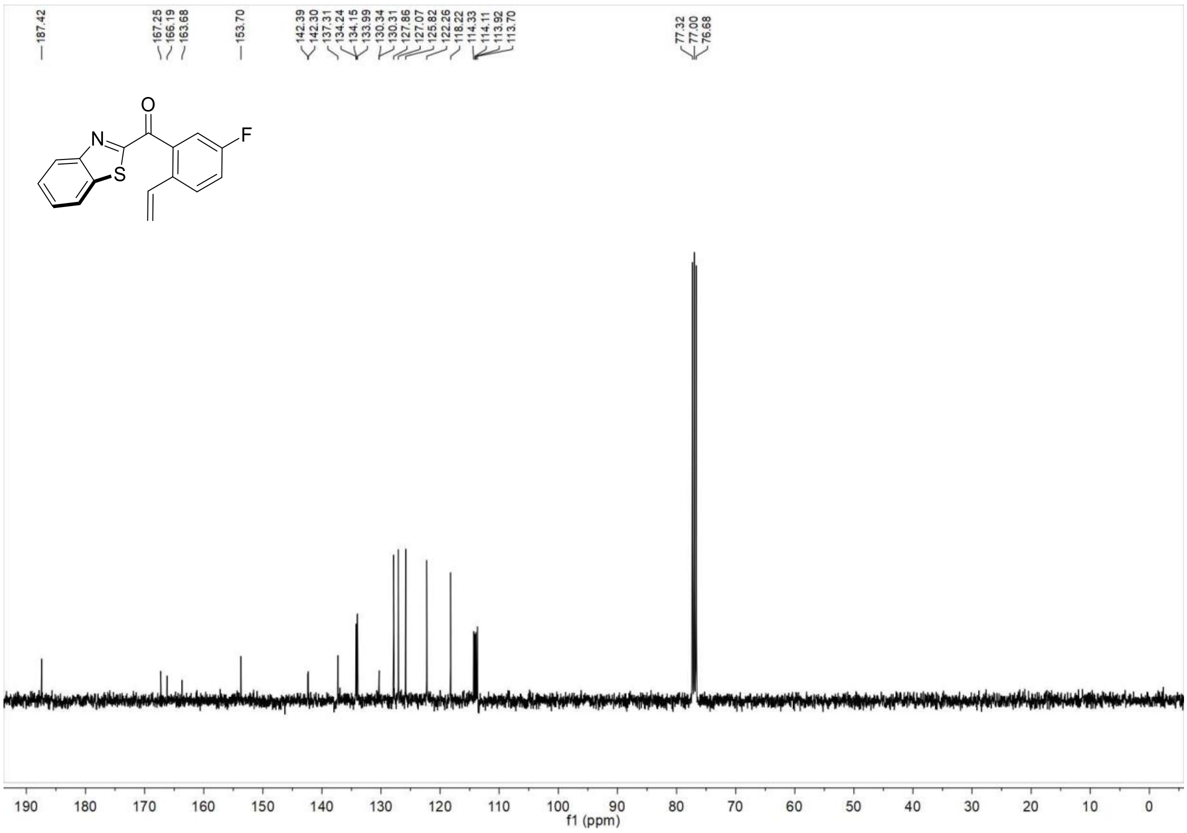

Supplementary Figure 73. ^{13}C-NMR of compound **benzo[*d*]thiazol-2-yl(5-fluoro-2-vinylphenyl)methanone**, recorded at 100 MHz and 25 °C in $CDCl_3$.

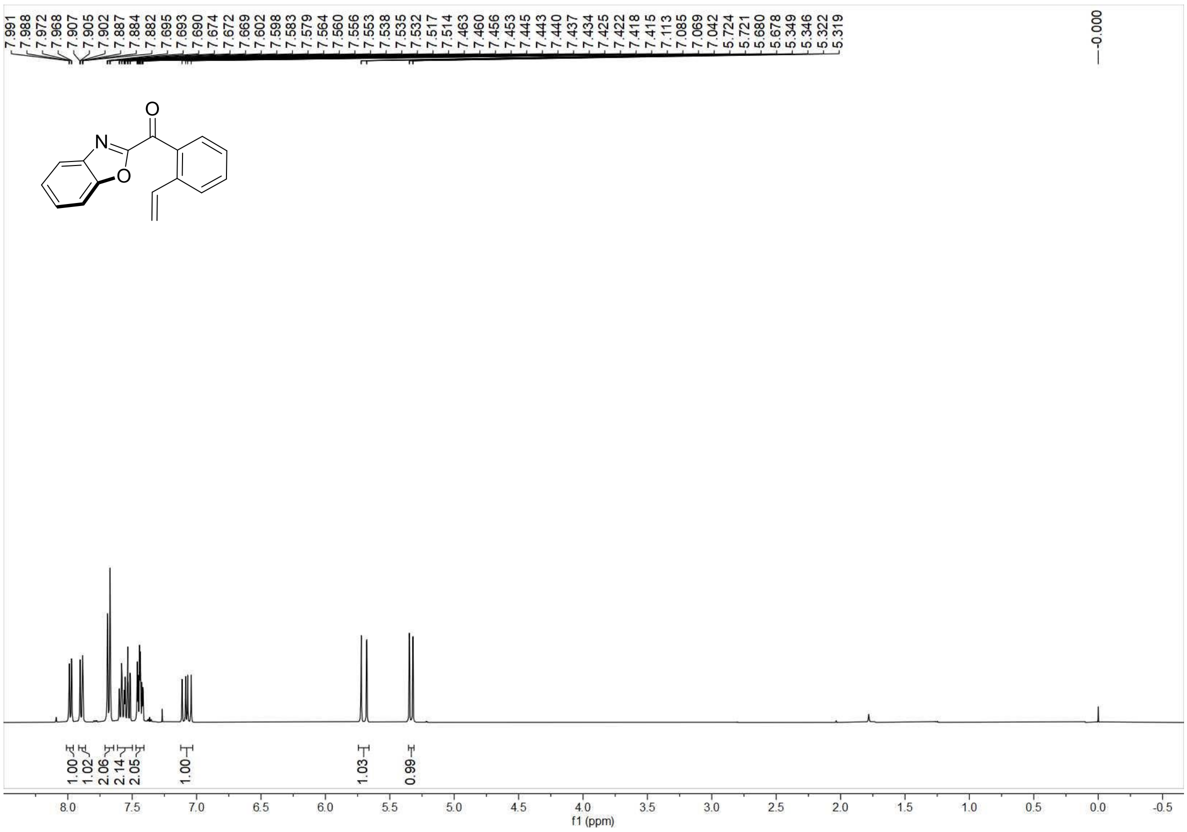

Supplementary Figure 74. ^{1}H-NMR of compound **benzo[*d*]thiazol-2-yl(2-vinylphenyl)methanone**, recorded at 400 MHz and 25 °C in $CDCl_3$.

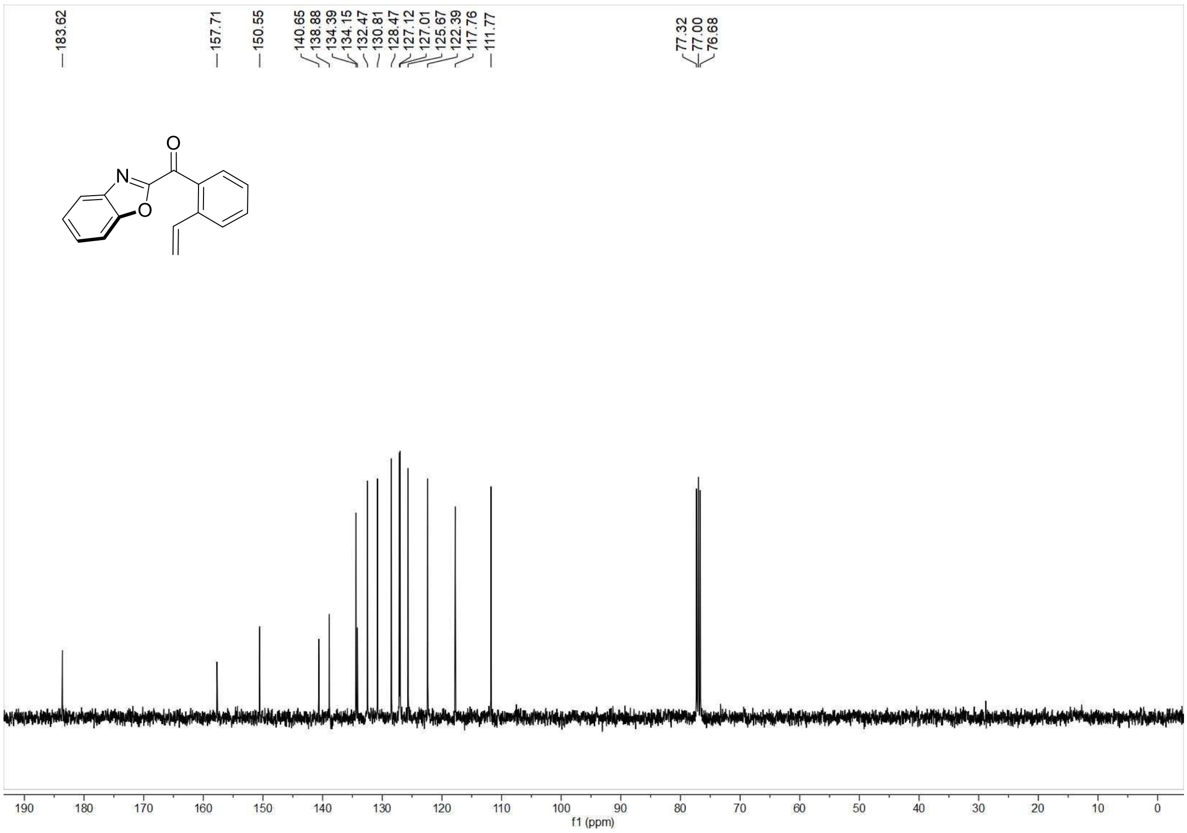

Supplementary Figure 75. ^{13}C-NMR of compound **benzo[*d*]thiazol-2-yl(2-vinylphenyl)methanone**, recorded at 100 MHz and 25 °C in $CDCl_3$.

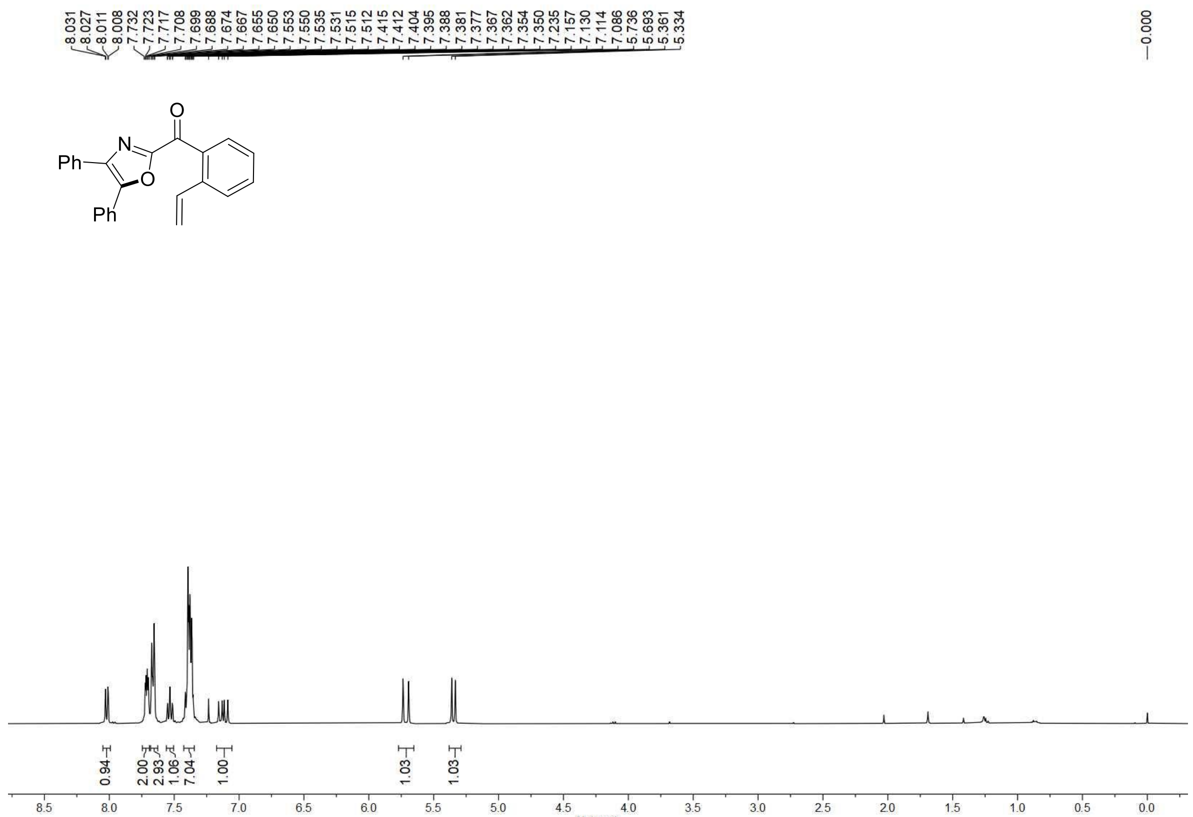

Supplementary Figure 76. ^{1}H-NMR of compound **(4,5-diphenyloxazol-2-yl)(2-vinylphenyl)methanone**, recorded at 400 MHz and 25 °C in $CDCl_3$.

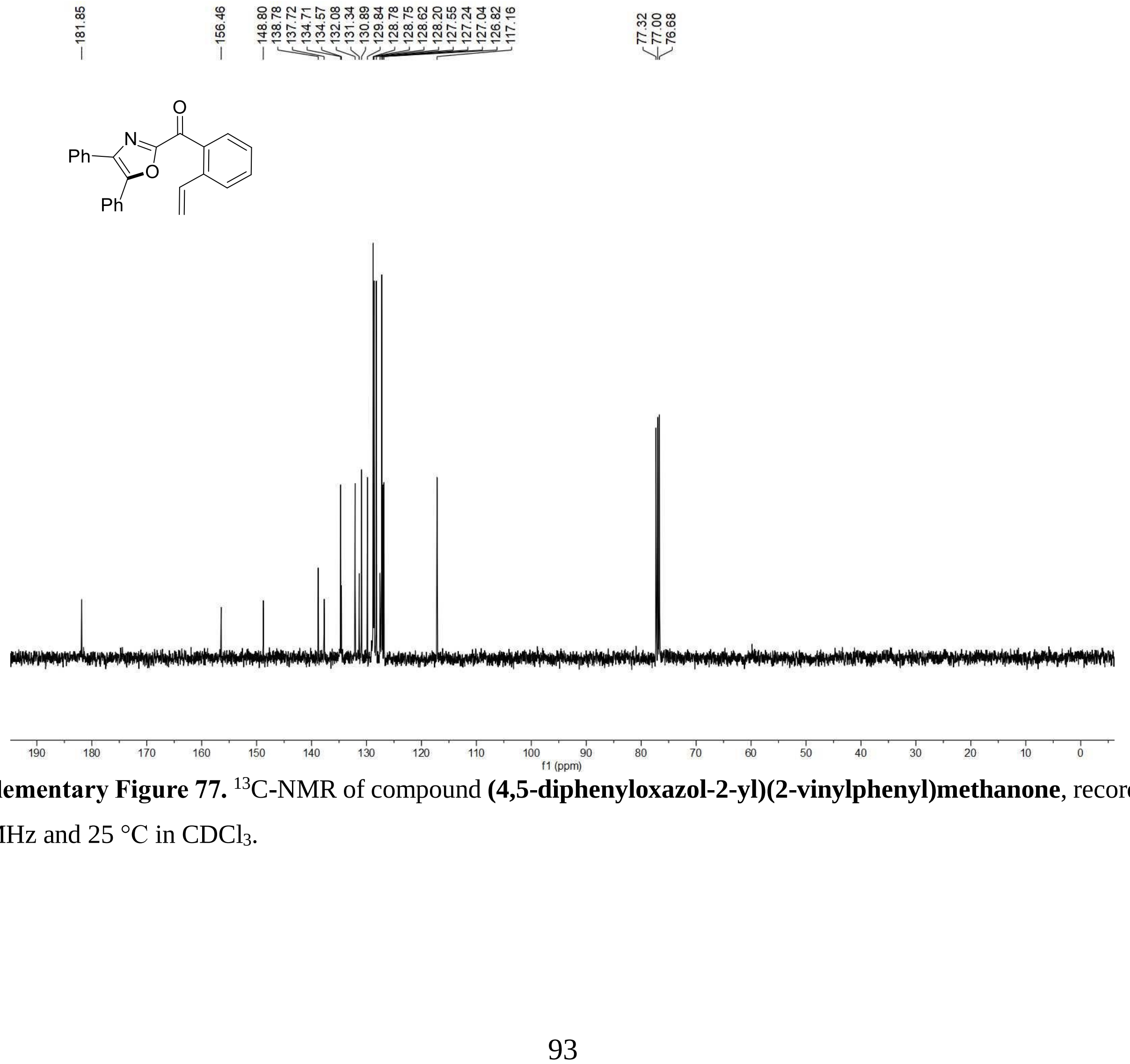

Supplementary Figure 77. ^{13}C-NMR of compound **(4,5-diphenyloxazol-2-yl)(2-vinylphenyl)methanone**, recorded at 100 MHz and 25 °C in $CDCl_3$.

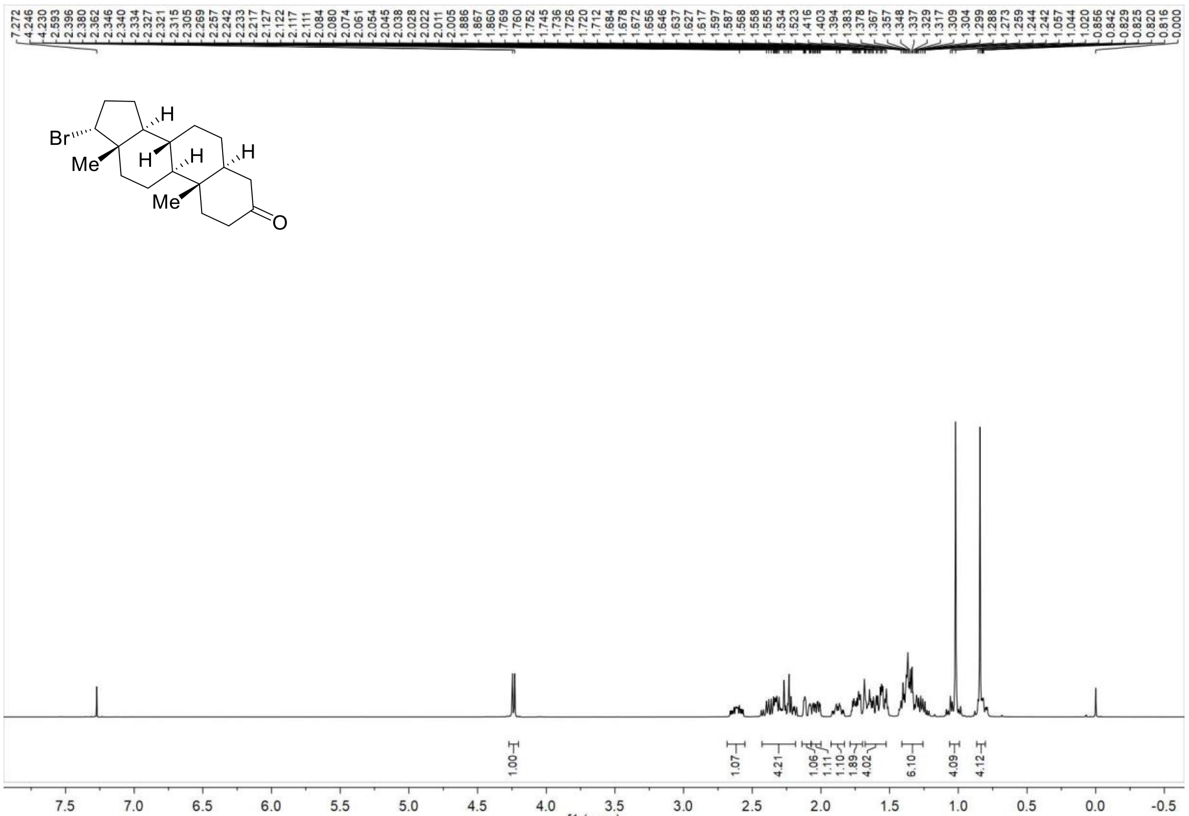

Supplementary Figure 78. ^{1}H-NMR of compound **Alkyl bromide derived from Stanolone** , recorded at 400 MHz and 25 °C in $CDCl_3$.

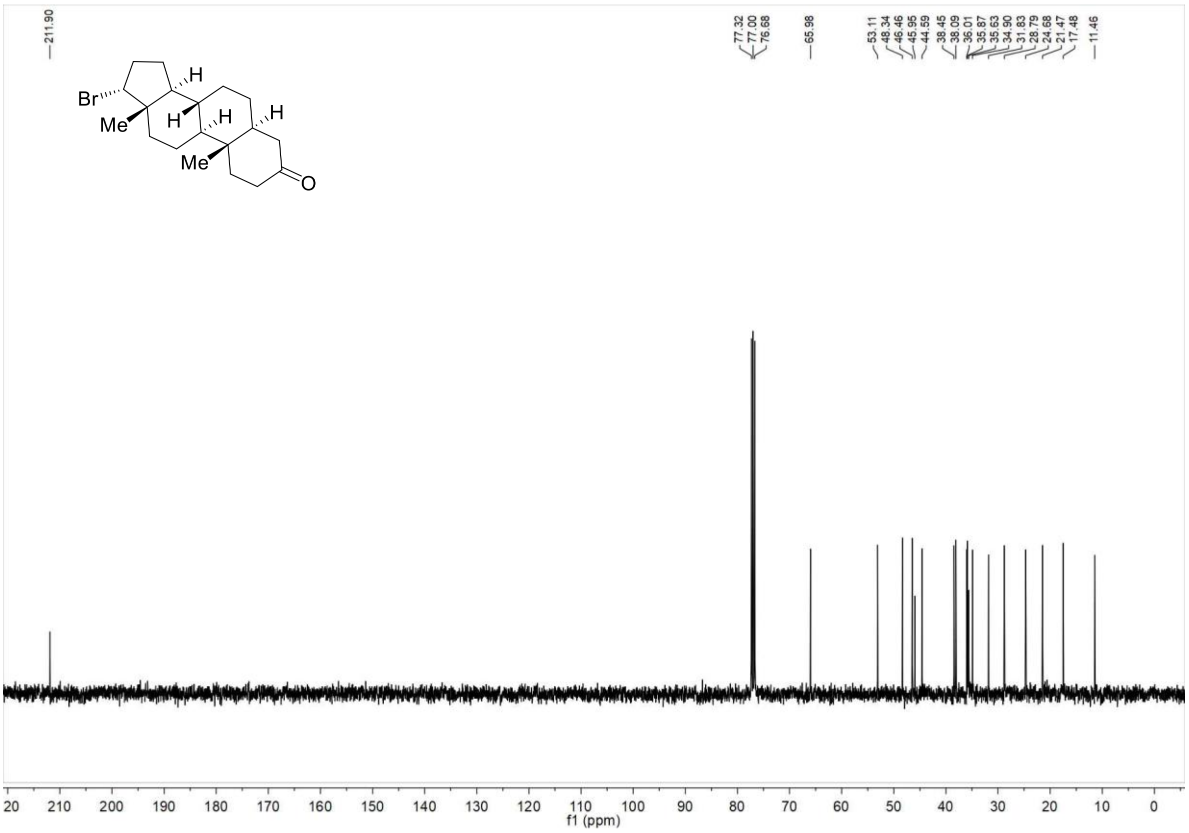

Supplementary Figure 79. ^{13}C-NMR of compound **Alkyl bromide derived from Stanolone**, recorded at 100 MHz and 25 °C in $CDCl_3$.

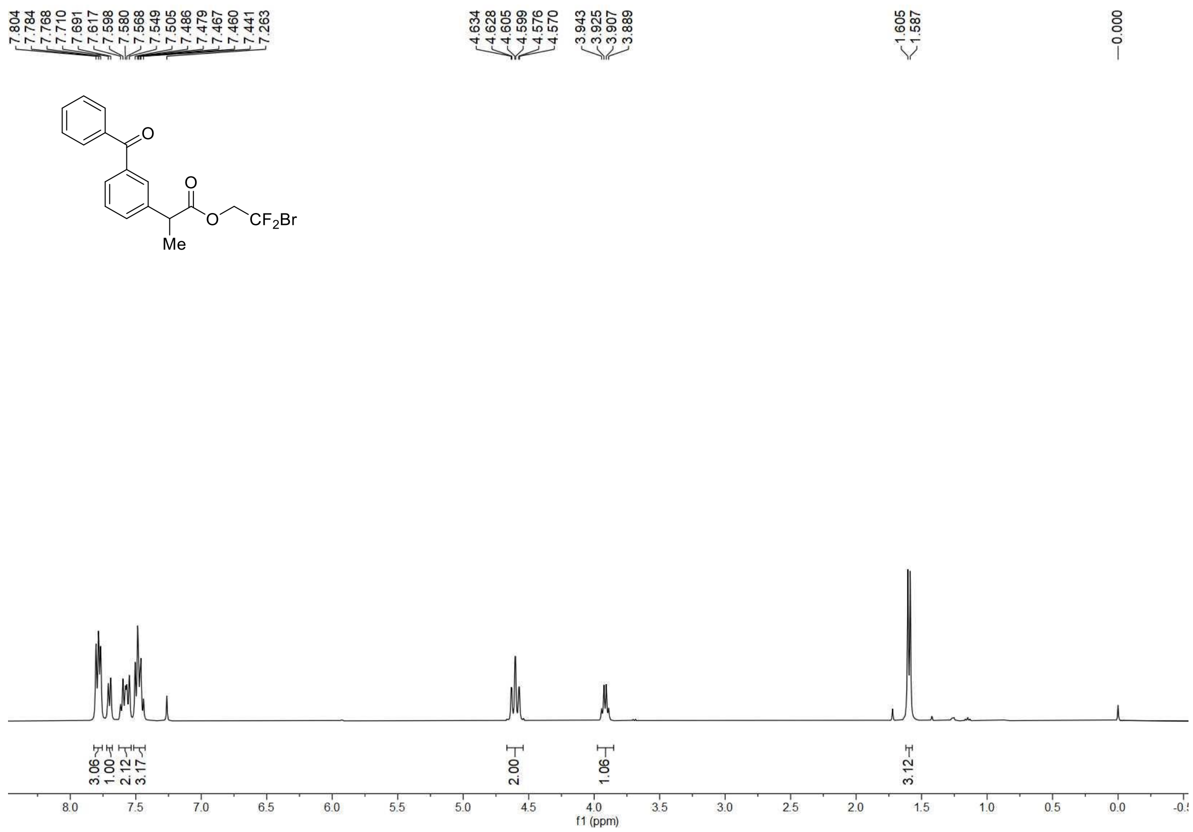

Supplementary Figure 80. ^{1}H-NMR of compound **Difluoroalkyl bromide derived from Ketoprofen** , recorded at 400 MHz and 25 °C in $CDCl_3$.

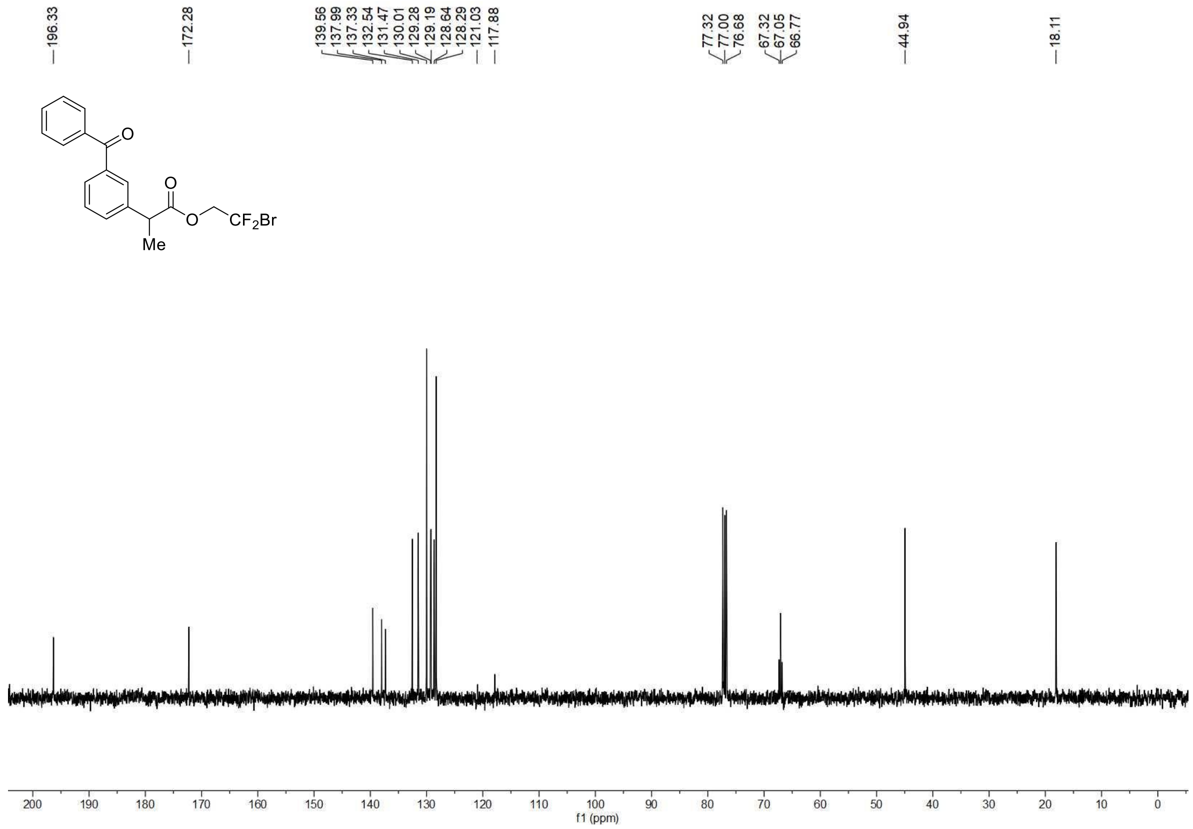

Supplementary Figure 81. ^{13}C-NMR of compound **Difluoroalkyl bromide derived from Ketoprofen**, recorded at 100 MHz and 25 °C in $CDCl_3$.

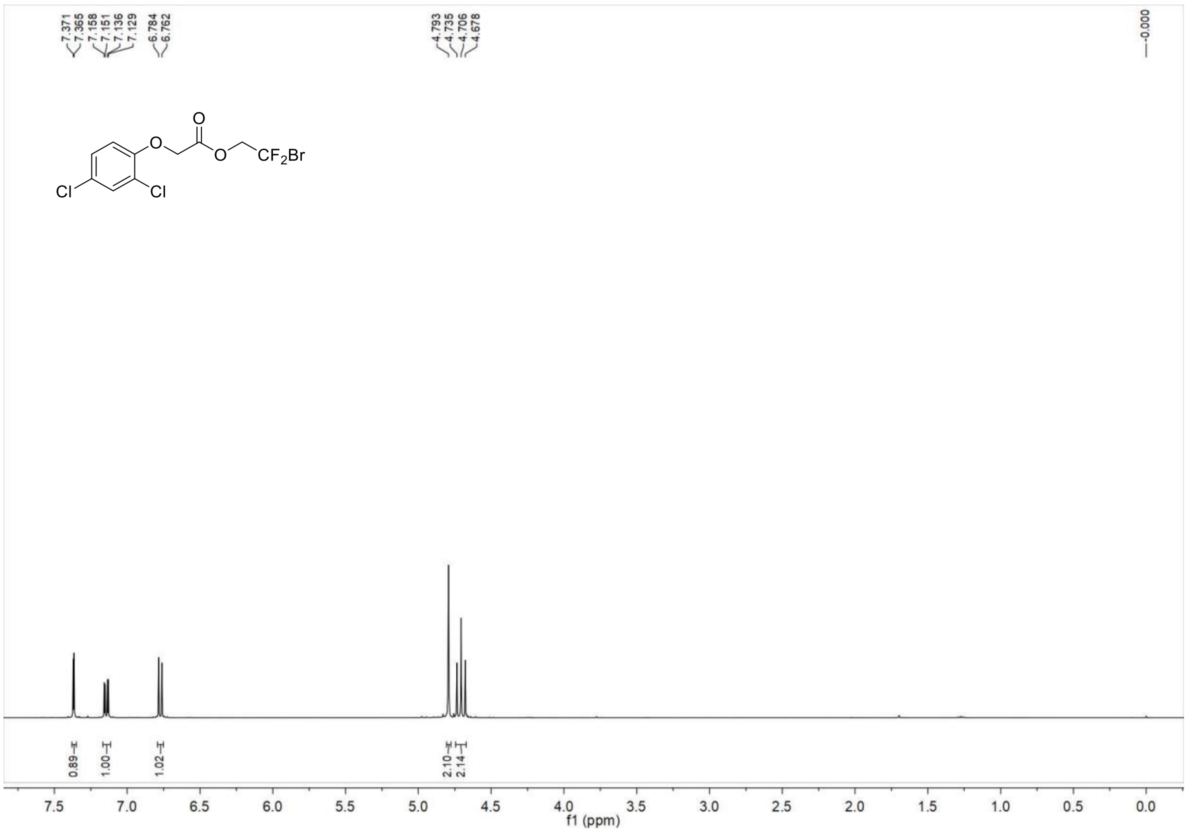

Supplementary Figure 82. ^{1}H-NMR of compound **Difluoroalkyl bromide derived from Fernoxone** , recorded at 400 MHz and 25 °C in $CDCl_3$.

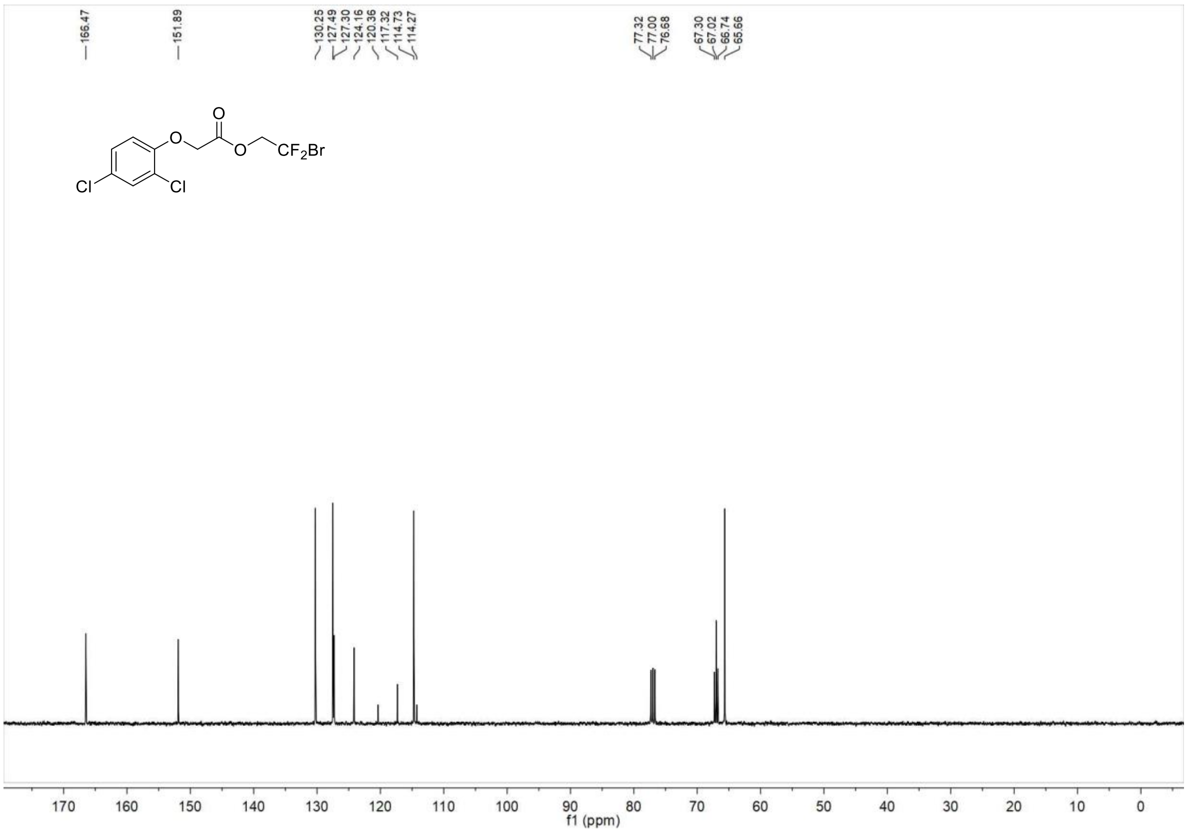

Supplementary Figure 83. ^{13}C-NMR of compound **Difluoroalkyl bromide derived from Fernoxone**, recorded at 100 MHz and 25 °C in $CDCl_3$.

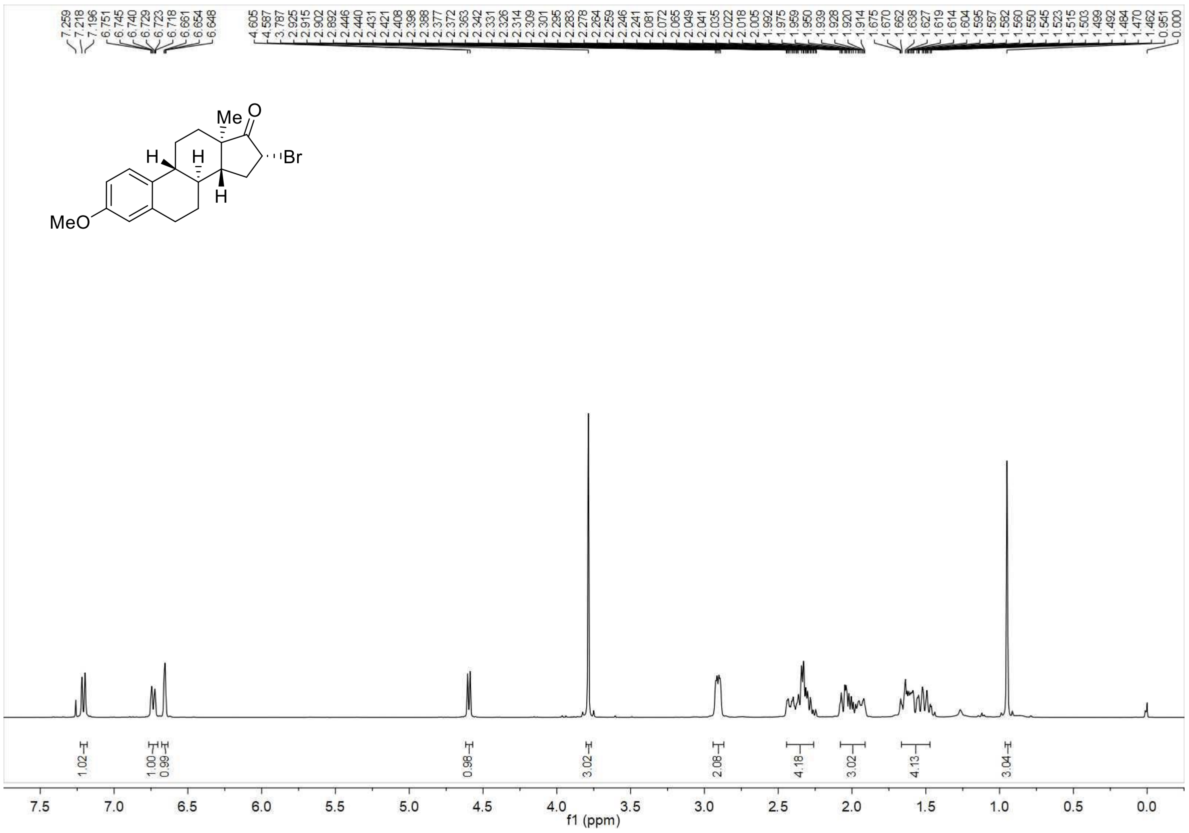

Supplementary Figure 84. ^{1}H-NMR of compound **Alkyl bromide derived from Estrone** , recorded at 400 MHz and 25 °C in $CDCl_3$.

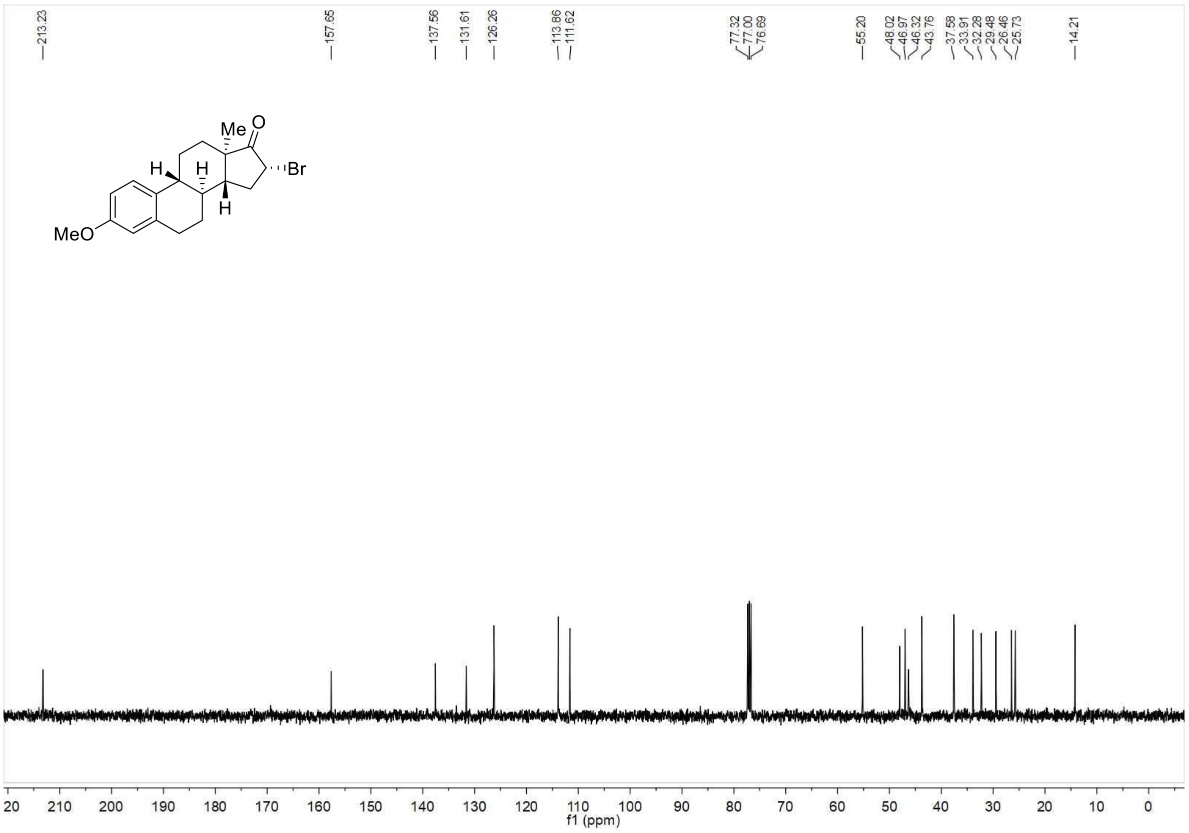

Supplementary Figure 85. ^{13}C-NMR of compound **Alkyl bromide derived from Estrone**, recorded at 100 MHz and 25 °C in $CDCl_3$.

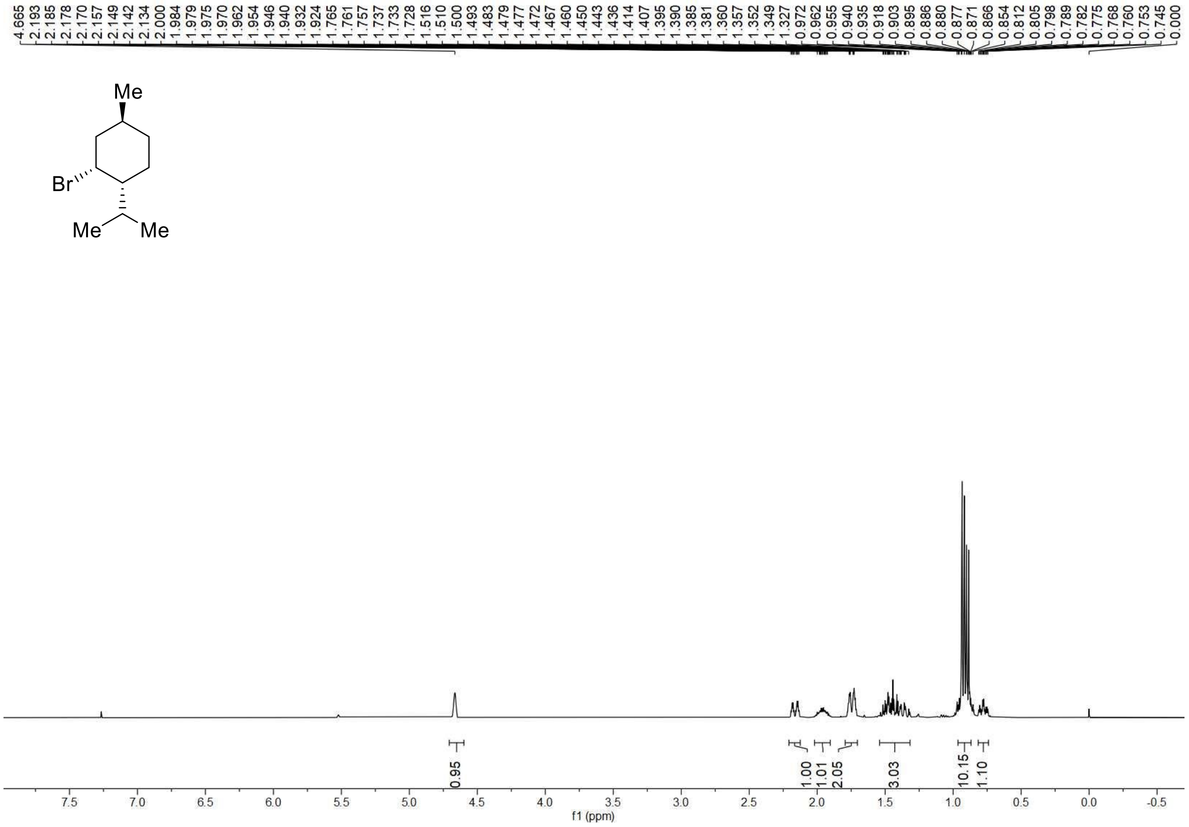

Supplementary Figure 86. ^{1}H-NMR of compound **Alkyl bromide derived from *L*-menthol** , recorded at 400 MHz and 25 °C in $CDCl_3$.

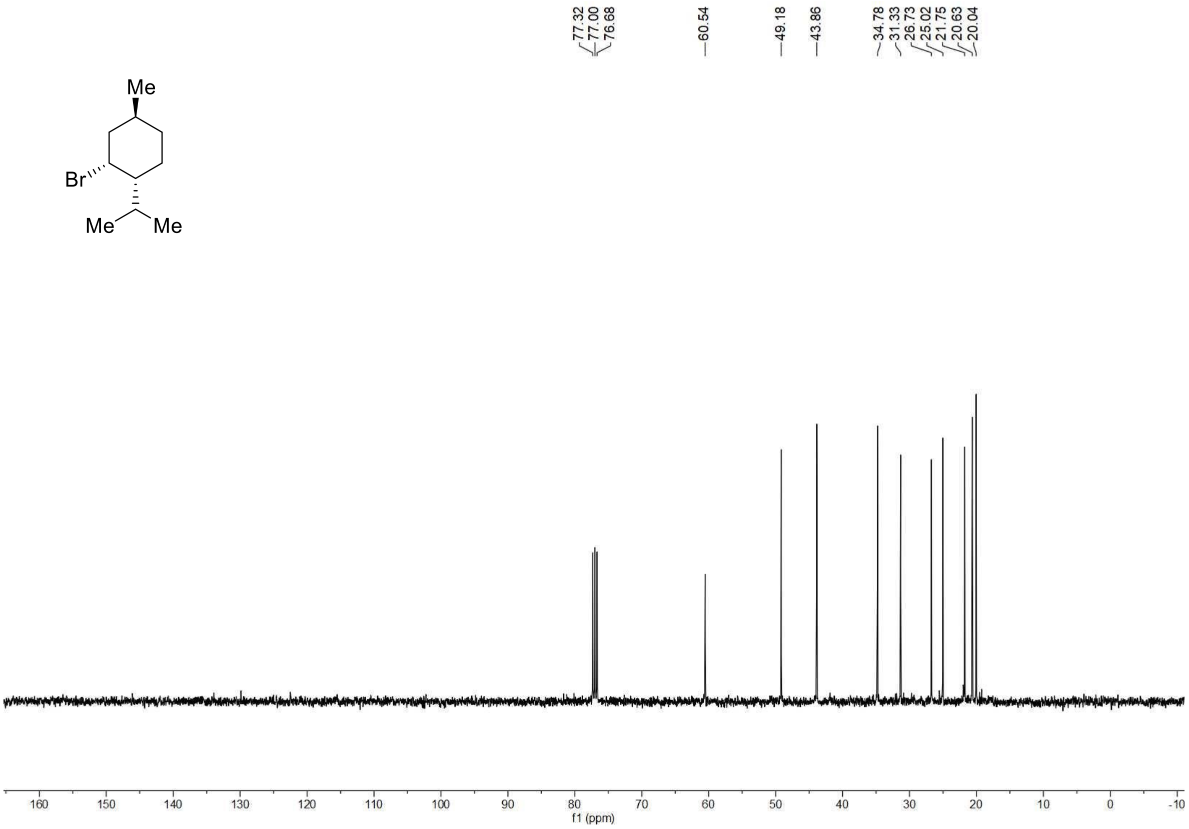

Supplementary Figure 87. ^{13}C-NMR of compound **Alkyl bromide derived from *L*-menthol**, recorded at 100 MHz and 25 °C in $CDCl_3$.

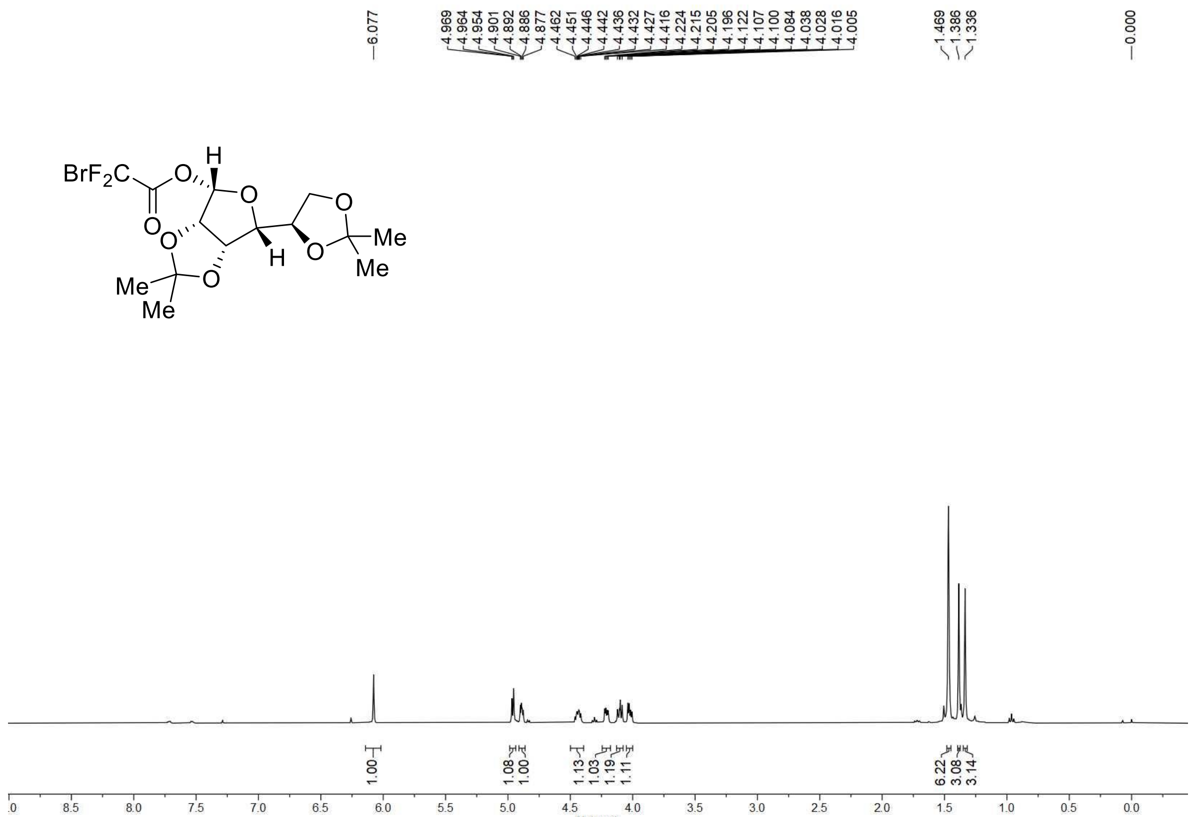

Supplementary Figure 88. ^{1}H-NMR of compound **Difluoroalkyl bromide derived from *D*-Mannofuranose**, recorded at 400 MHz and 25 °C in $CDCl_3$.

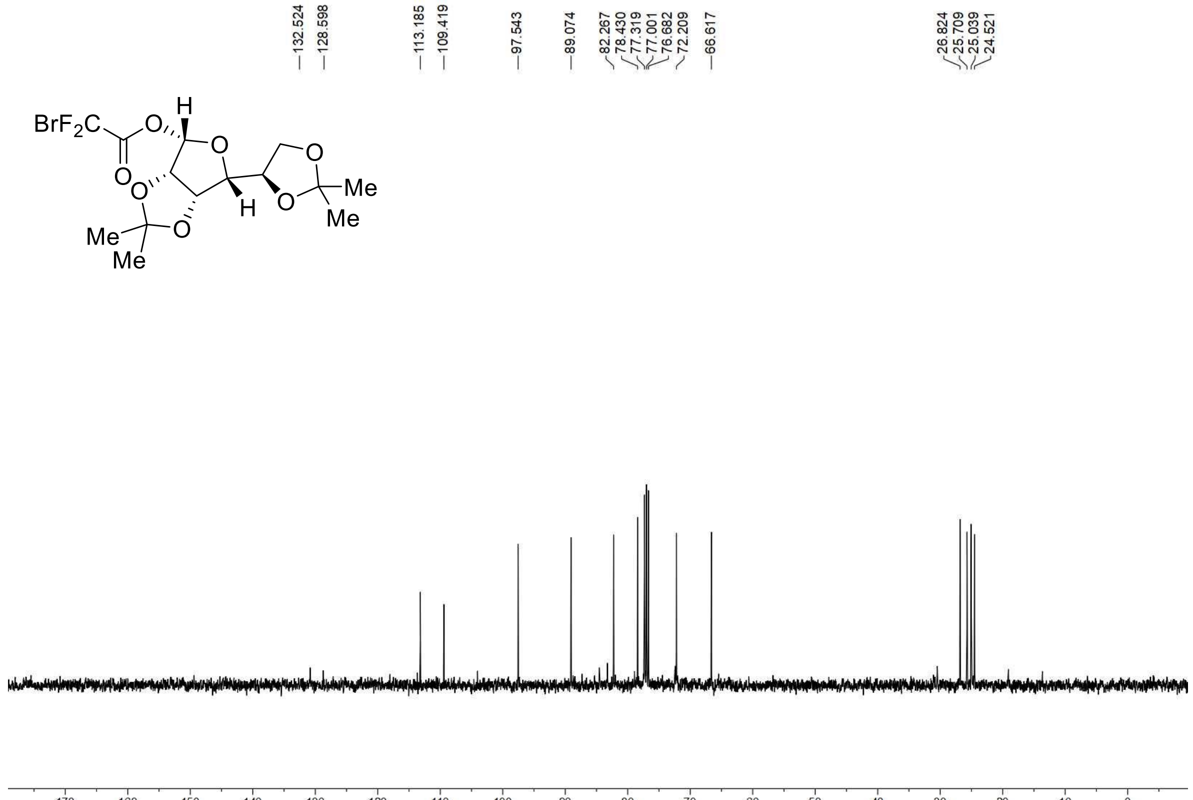

Supplementary Figure 89. ^{13}C-NMR of compound **Difluoroalkyl bromide derived from *D*-Mannofuranose**, recorded at 100 MHz and 25 °C in $CDCl_3$.

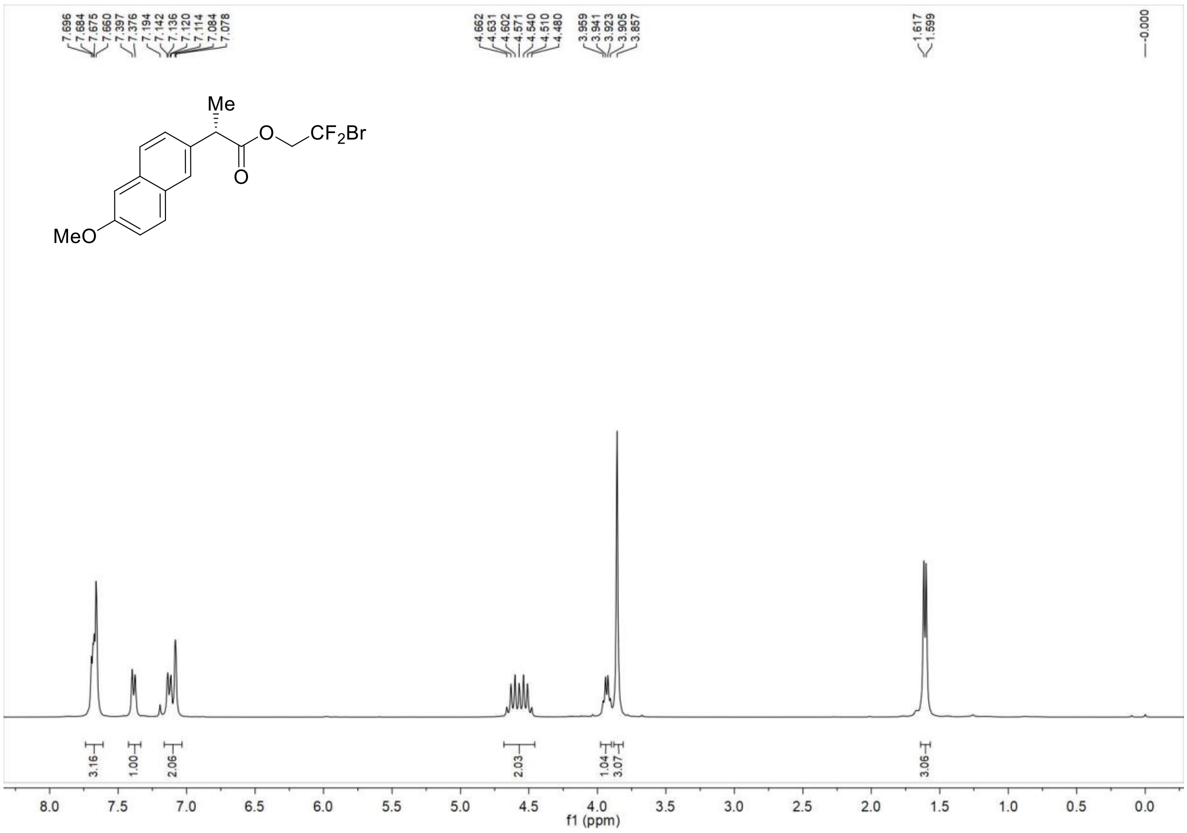

Supplementary Figure 90. ^{1}H-NMR of compound **Difluoroalkyl bromide derived from Naproxen**, recorded at 400 MHz and 25 °C in $CDCl_3$.

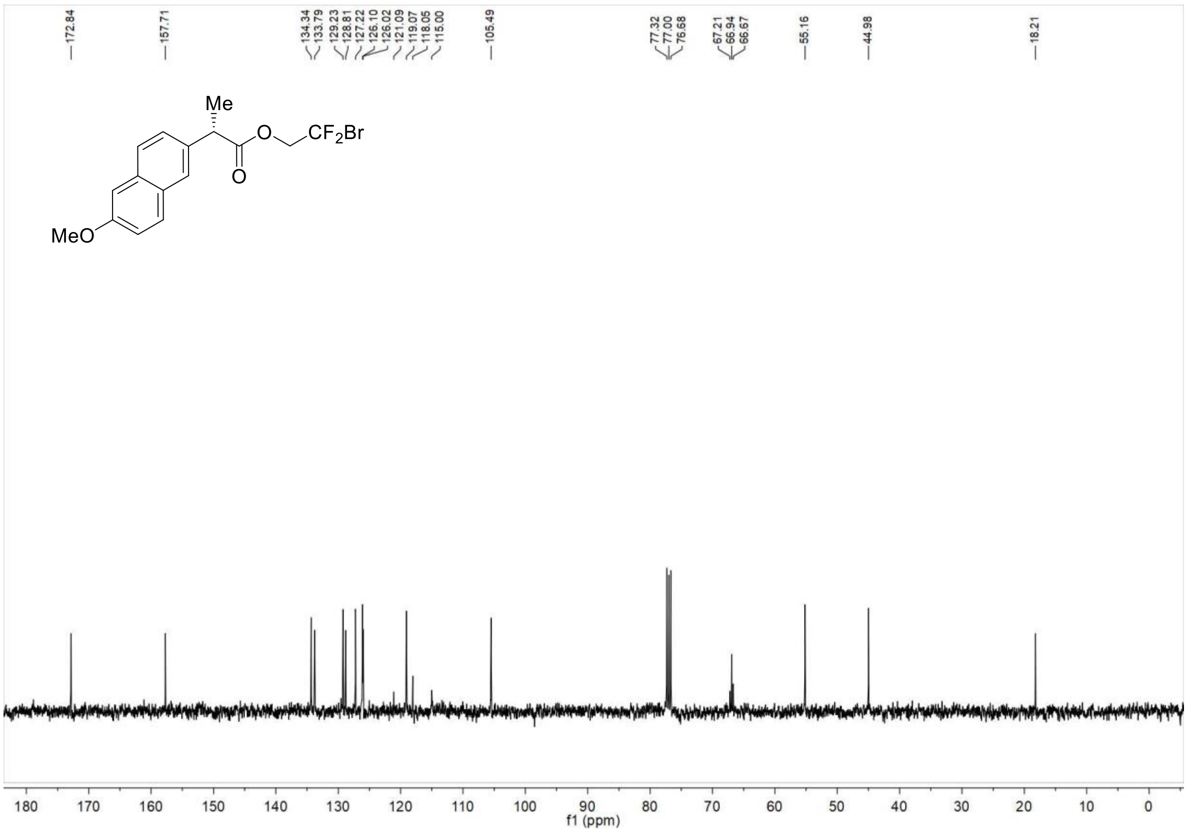

Supplementary Figure 91. ^{13}C-NMR of compound **Difluoroalkyl bromide derived from Naproxen**, recorded at 100 MHz and 25 °C in $CDCl_3$.

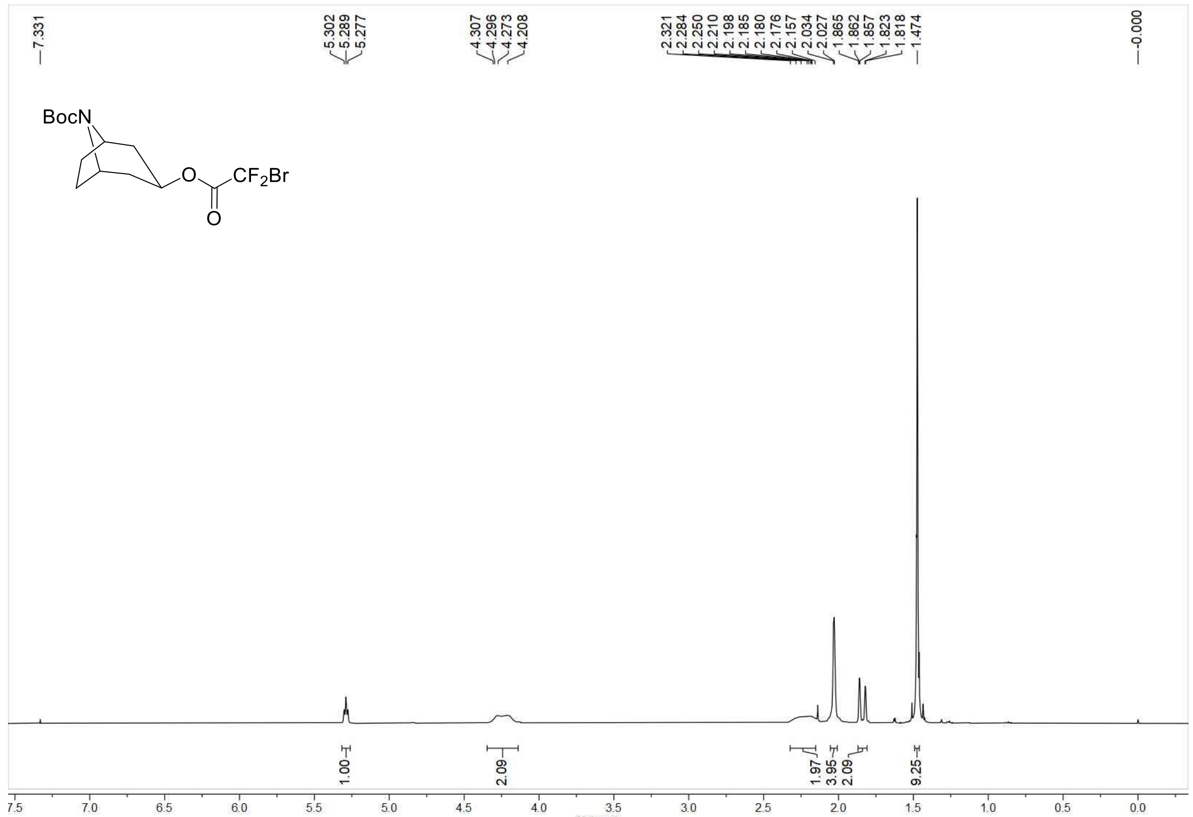

Supplementary Figure 92. ^{1}H-NMR of compound **Difluoroalkyl bromide derived from Nortropine**, recorded at 400 MHz and 25 °C in $CDCl_3$.

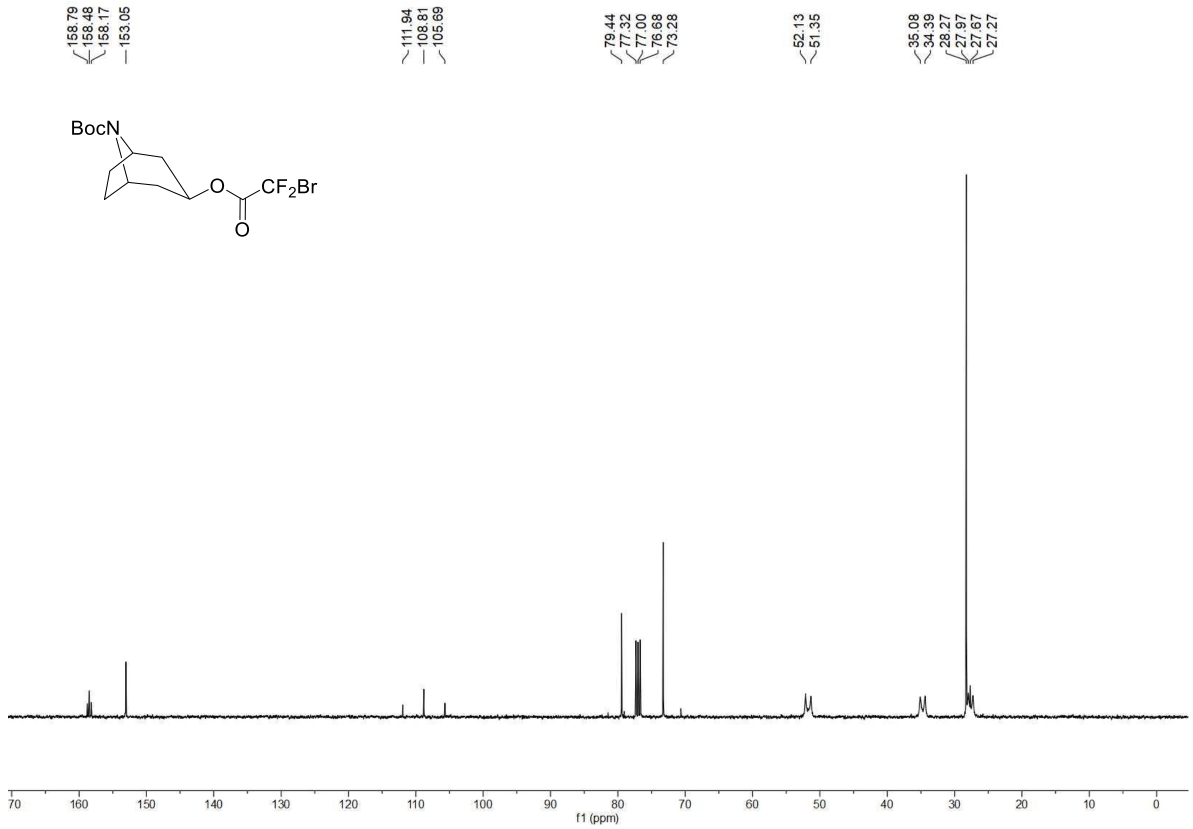

Supplementary Figure 93. ^{13}C-NMR of compound **Difluoroalkyl bromide derived from Nortropine**, recorded at 100 MHz and 25 °C in $CDCl_3$.

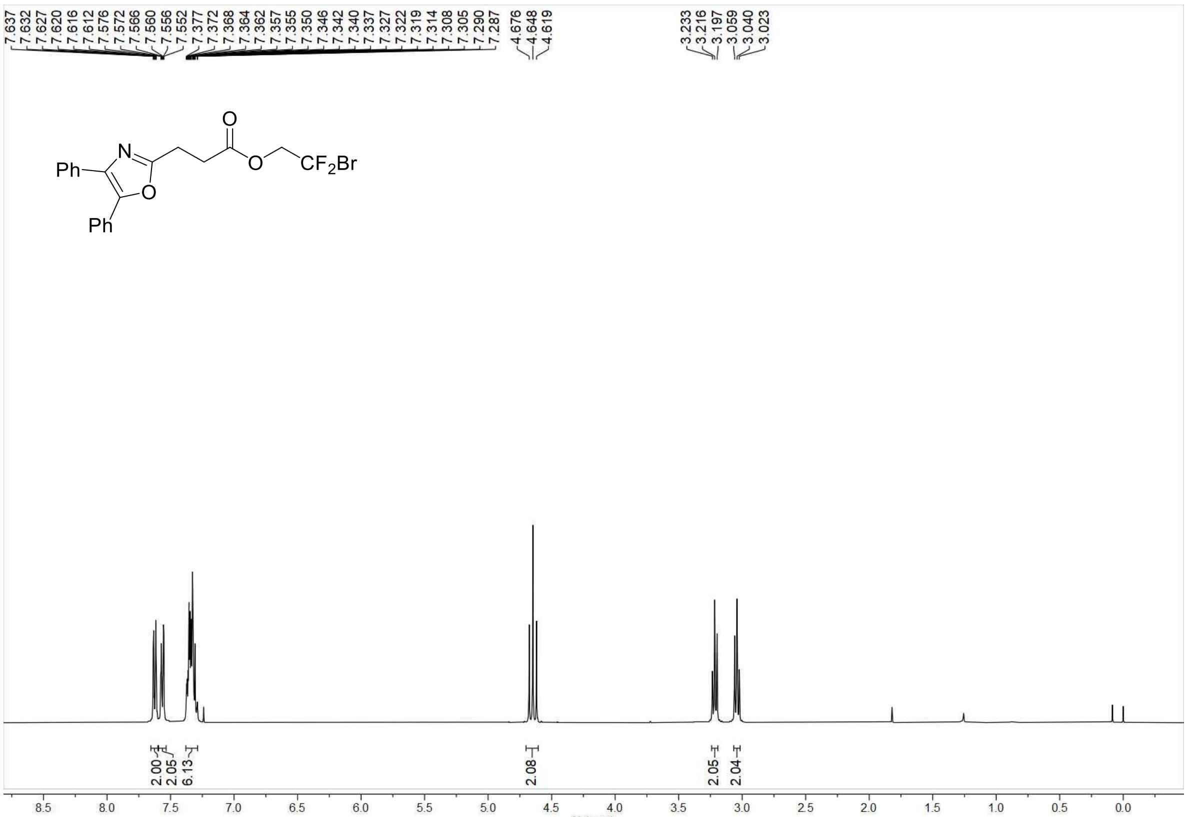

Supplementary Figure 94. ^{1}H-NMR of compound **Difluoroalkyl bromide derived from Oxaprozin**, recorded at 400 MHz and 25 °C in $CDCl_3$.

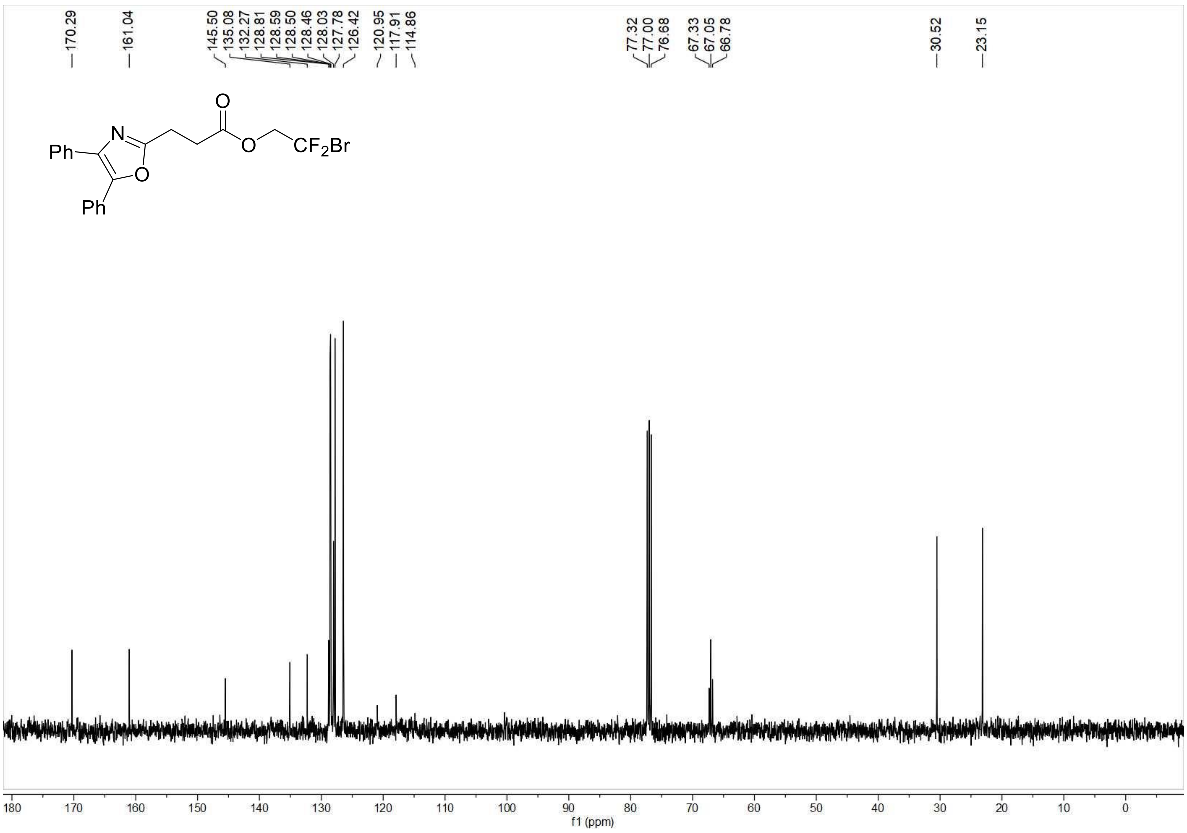

Supplementary Figure 95. ^{13}C-NMR of compound **Difluoroalkyl bromide derived from Oxaprozin**, recorded at 100 MHz and 25 °C in $CDCl_3$.

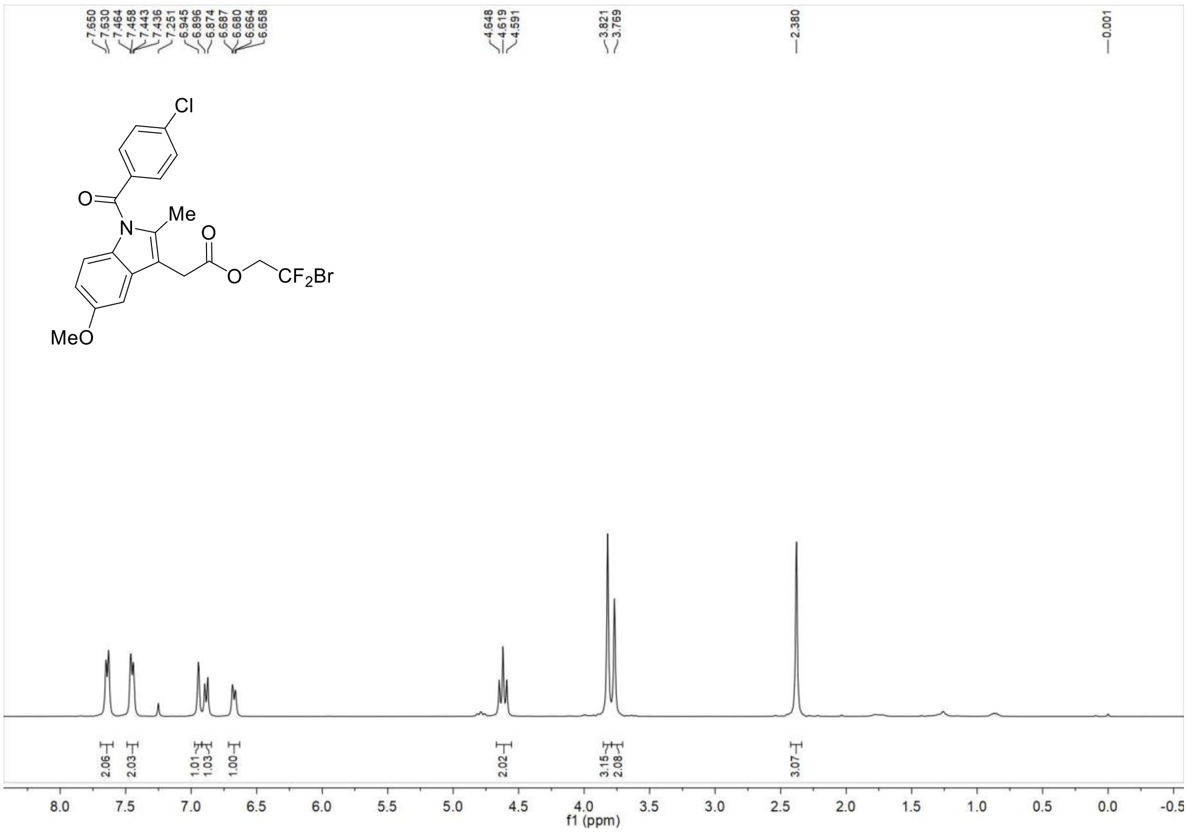

Supplementary Figure 96. ^{1}H-NMR of compound **Difluoroalkyl bromide derived from Indometacin**, recorded at 400 MHz and 25 °C in $CDCl_3$.

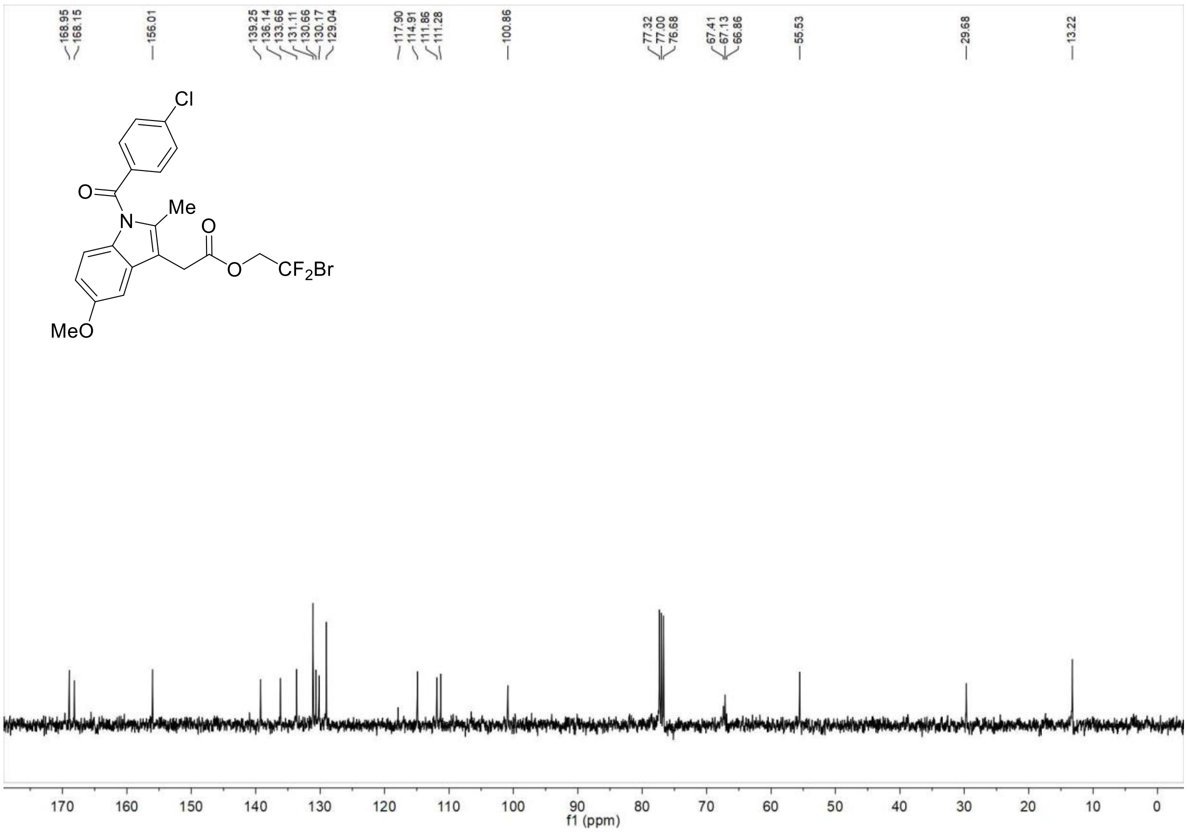

Supplementary Figure 97. ^{13}C-NMR of compound **Difluoroalkyl bromide derived from Indometacin**, recorded at 100 MHz and 25 °C in $CDCl_3$.

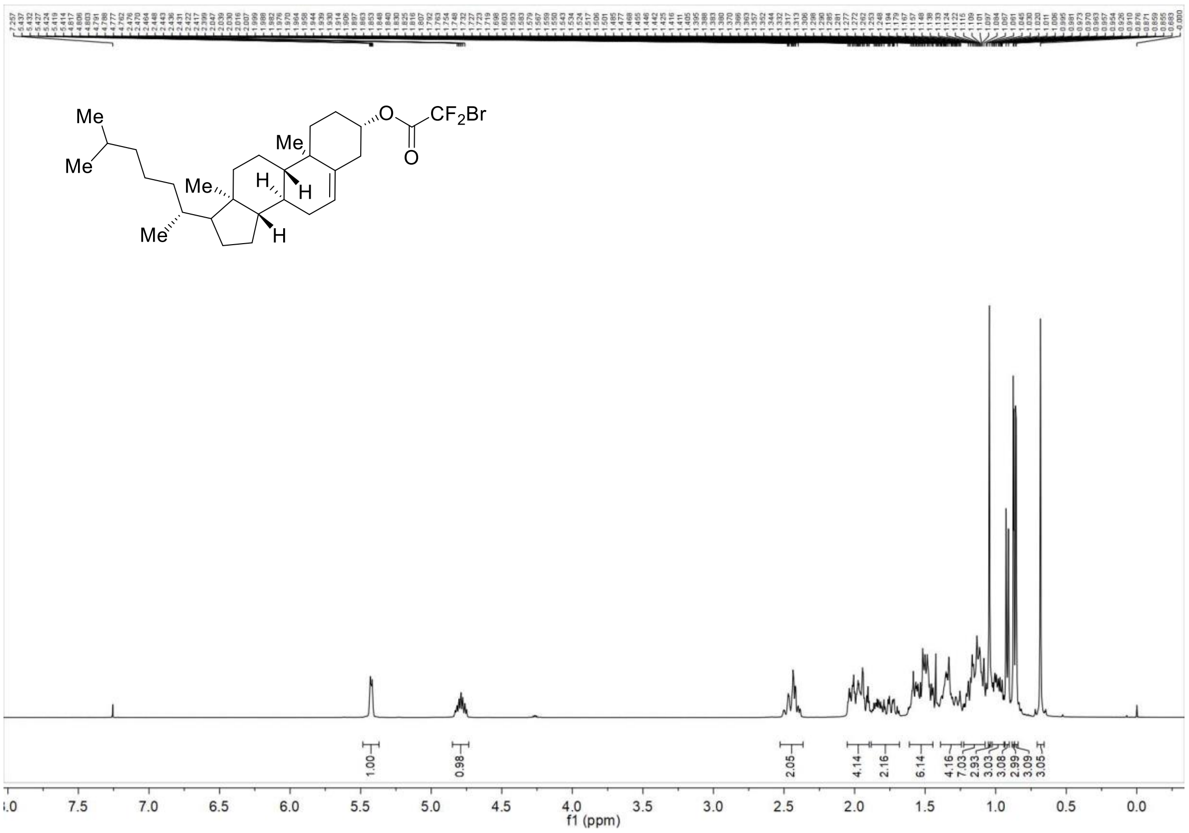

Supplementary Figure 98. ^{1}H-NMR of compound **Difluoroalkyl bromide derived from Cholesterol**, recorded at 400 MHz and 25 °C in $CDCl_3$.

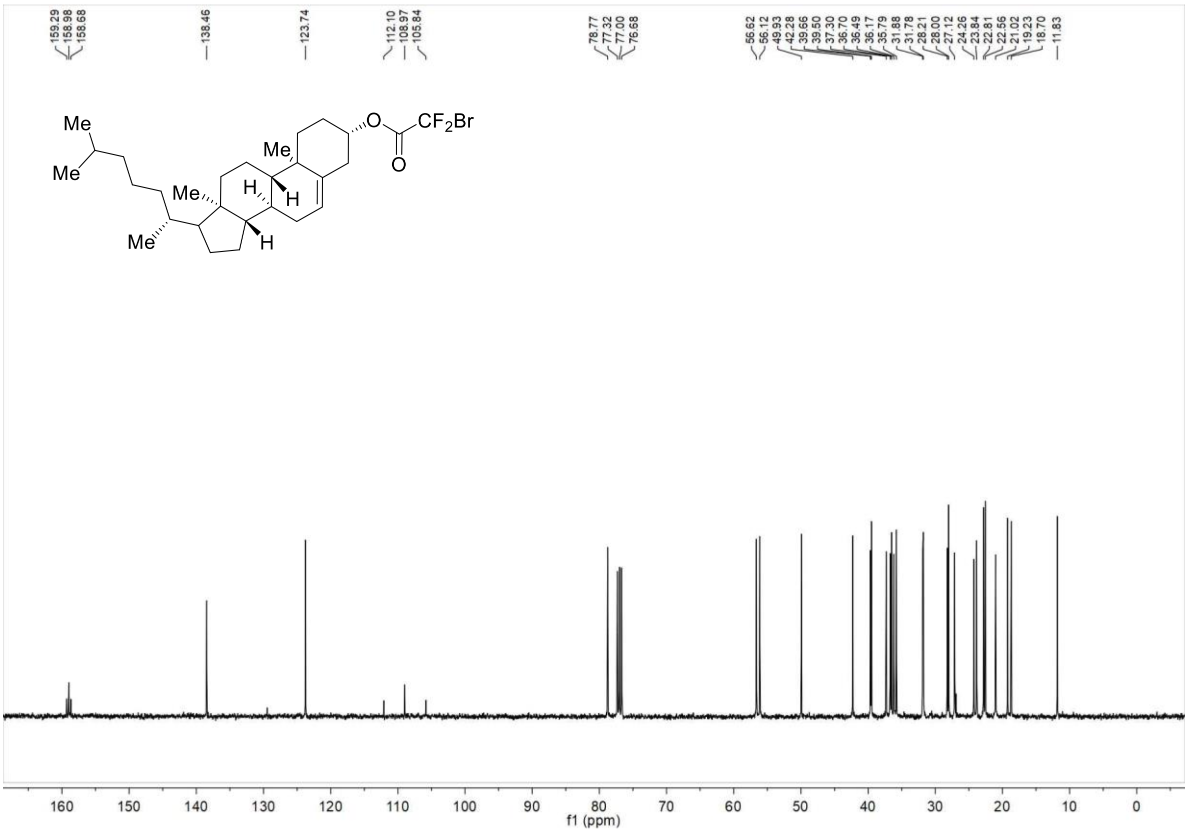

Supplementary Figure 99. ^{13}C-NMR of compound **Difluoroalkyl bromide derived from Cholesterol**, recorded at 100 MHz and 25 °C in $CDCl_3$.

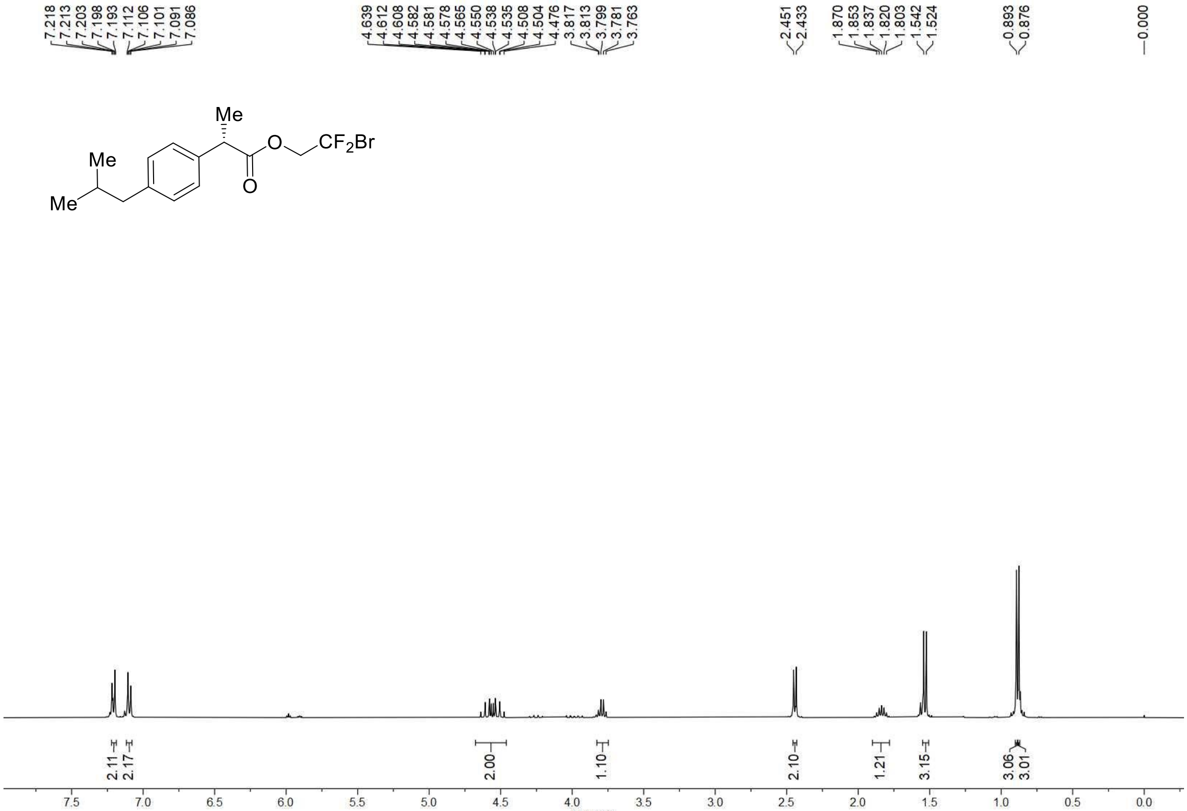

Supplementary Figure 100. ^{1}H-NMR of compound **Difluoroalkyl bromide derived from Ibuprofen**, recorded at 400 MHz and 25 °C in $CDCl_3$.

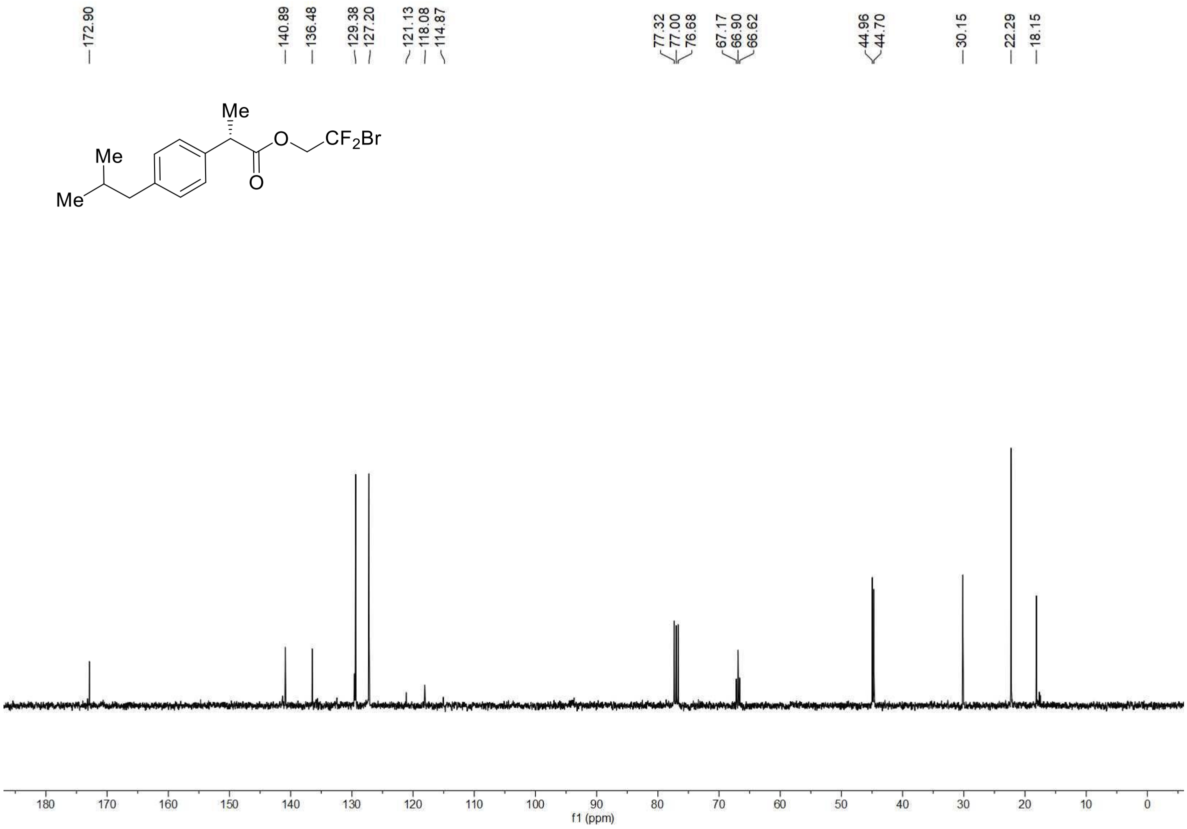

Supplementary Figure 101. ^{13}C-NMR of compound **Difluoroalkyl bromide derived from Ibuprofen**, recorded at 100 MHz and 25 °C in $CDCl_3$.

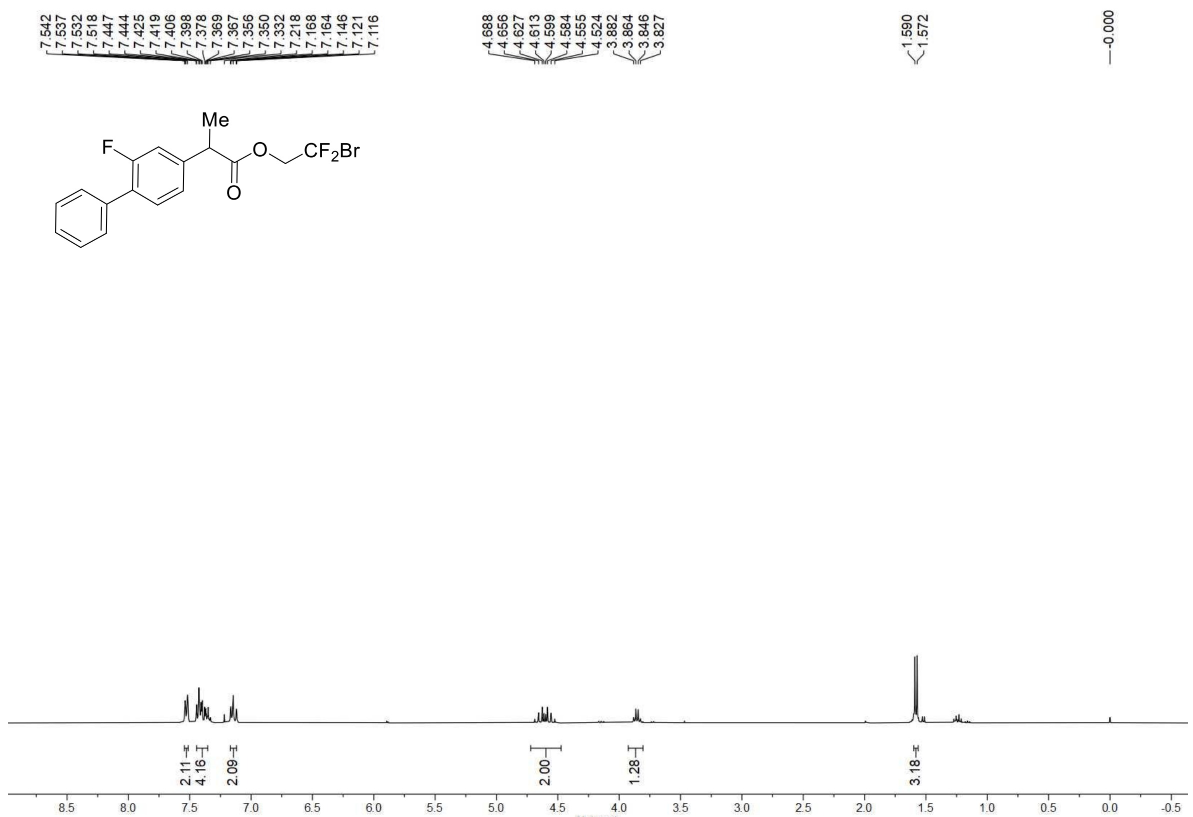

Supplementary Figure 102. ^{1}H-NMR of compound **Difluoroalkyl bromide derived from Flurbiprofen**, recorded at 400 MHz and 25 °C in $CDCl_3$.

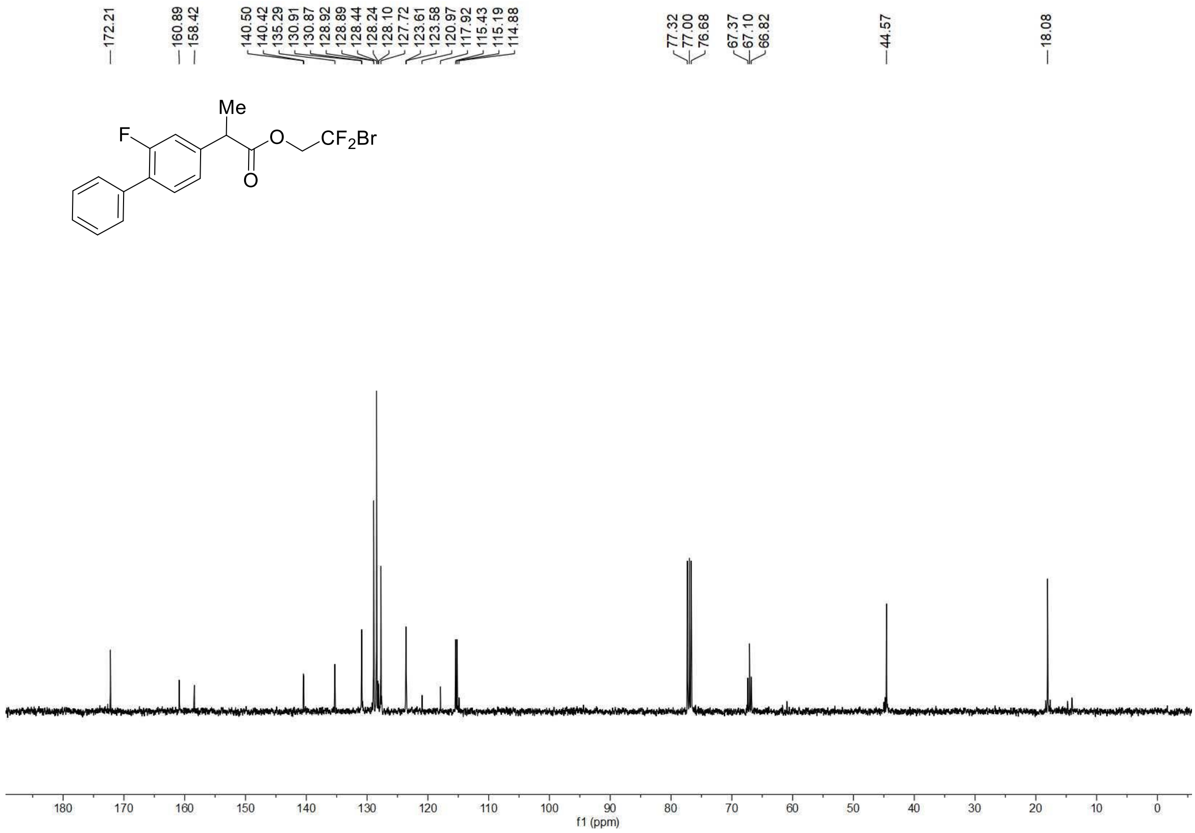

Supplementary Figure 103. ^{13}C-NMR of compound **Difluoroalkyl bromide derived from Flurbiprofen**, recorded at 100 MHz and 25 °C in $CDCl_3$.

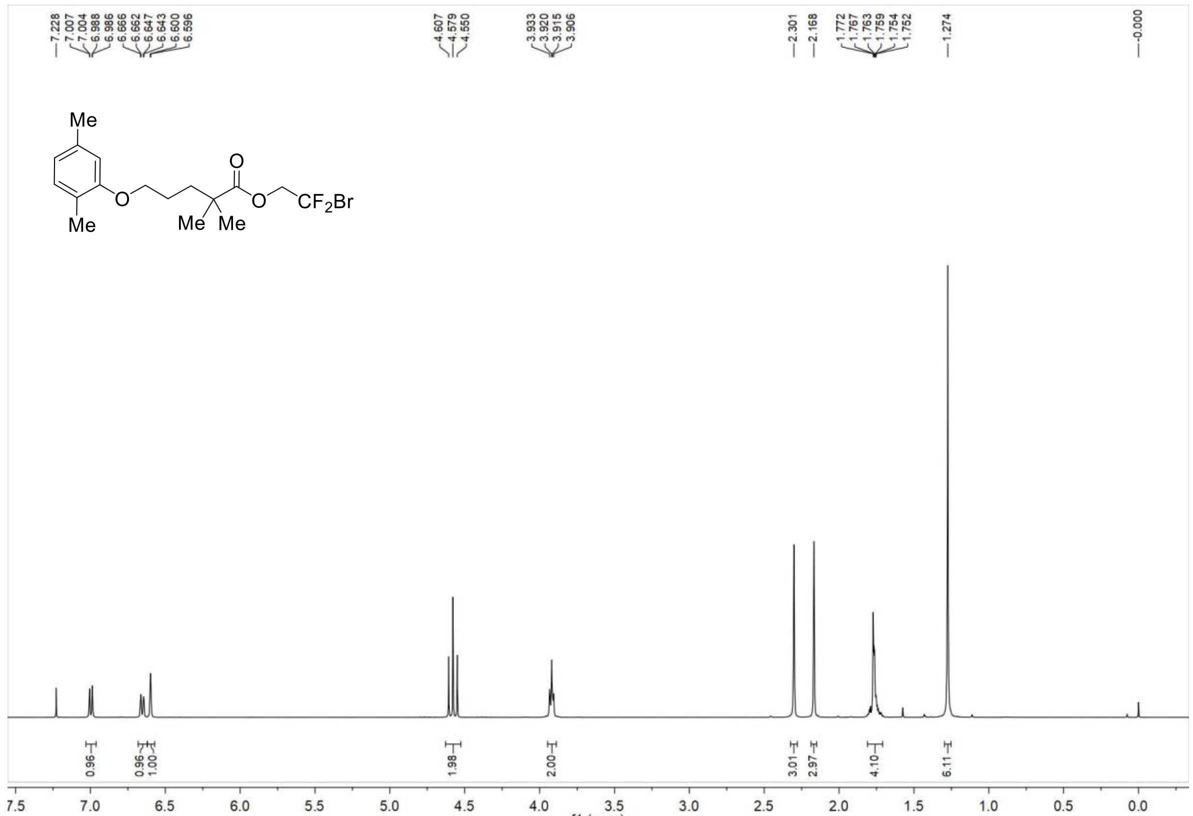

Supplementary Figure 104. ^{1}H-NMR of compound **Difluoroalkyl bromide derived from Gemfibrozil**, recorded at 400 MHz and 25 °C in $CDCl_3$.

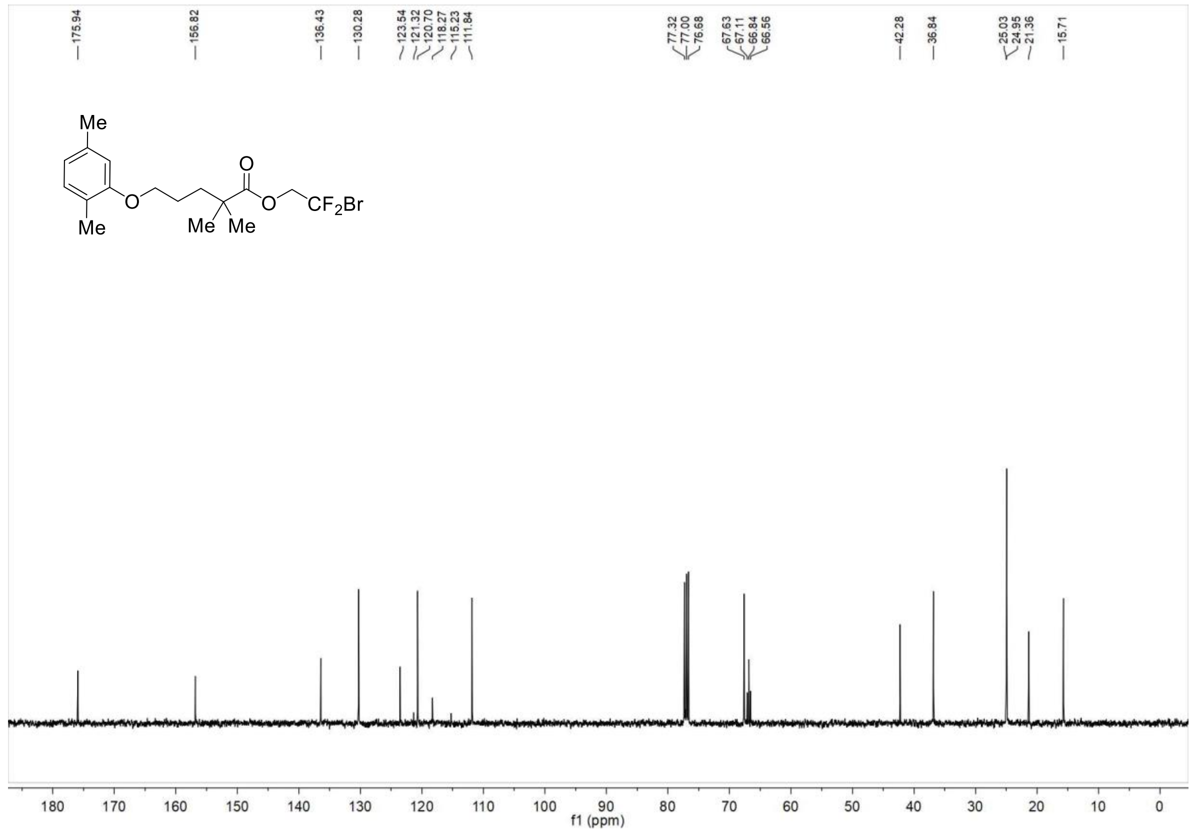

Supplementary Figure 105. ^{13}C-NMR of compound **Difluoroalkyl bromide derived from Gemfibrozil**, recorded at 100 MHz and 25 °C in $CDCl_3$.

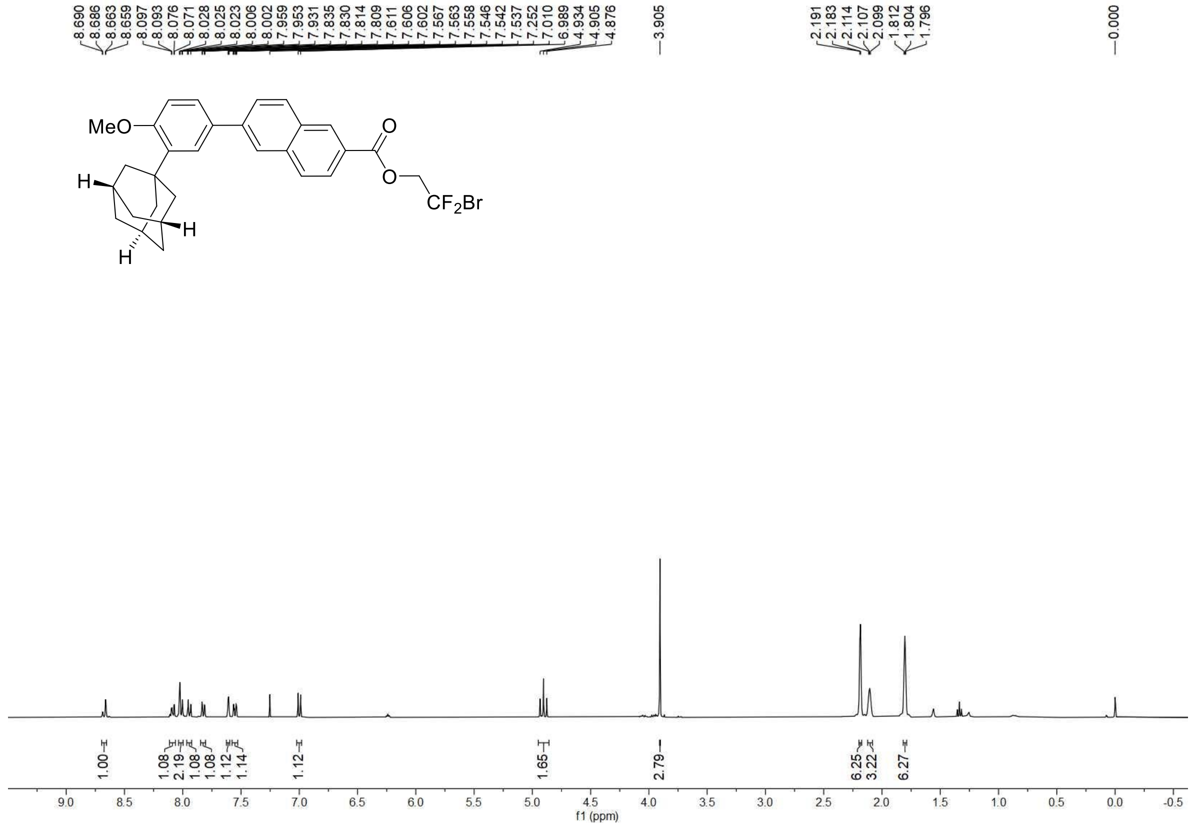

Supplementary Figure 106. ^{1}H-NMR of compound **Difluoroalkyl bromide derived from Adapalene**, recorded at 400 MHz and 25 °C in $CDCl_3$.

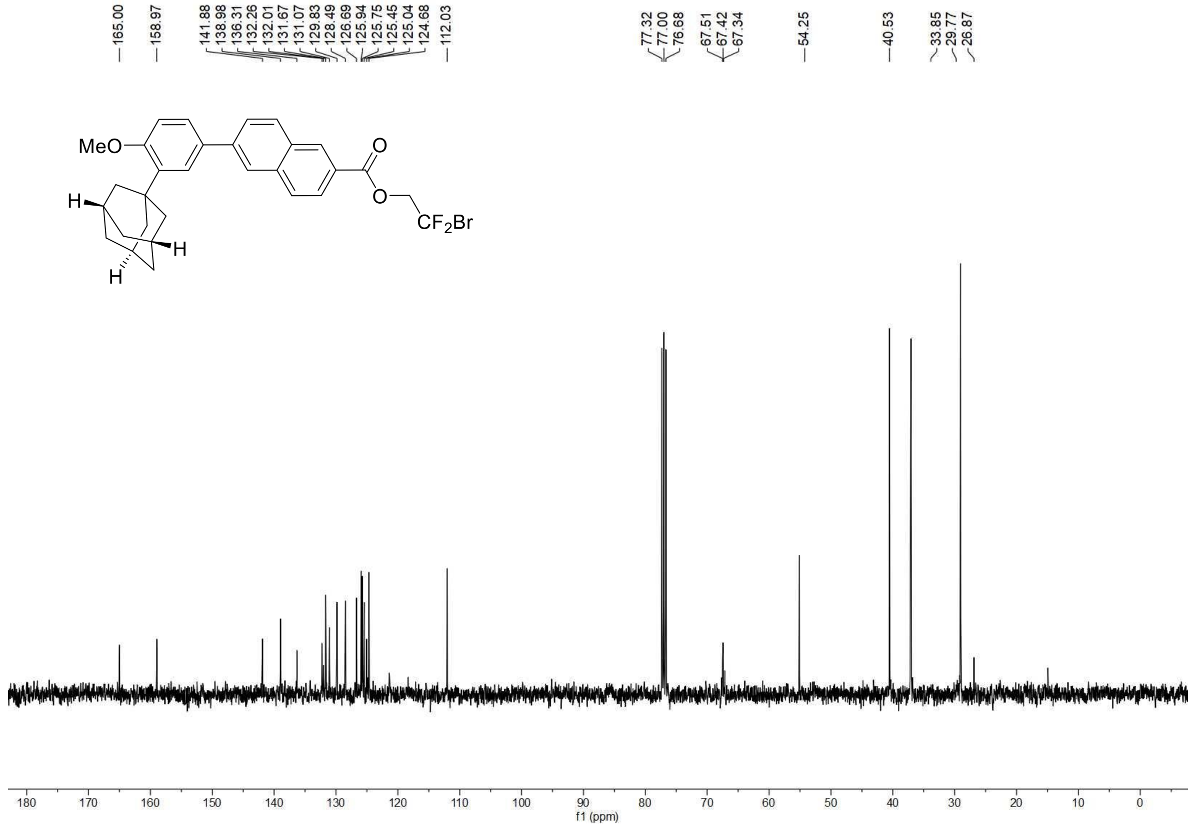

Supplementary Figure 107. ^{13}C-NMR of compound **Difluoroalkyl bromide derived from Adapalene**, recorded at 100 MHz and 25 °C in $CDCl_3$.

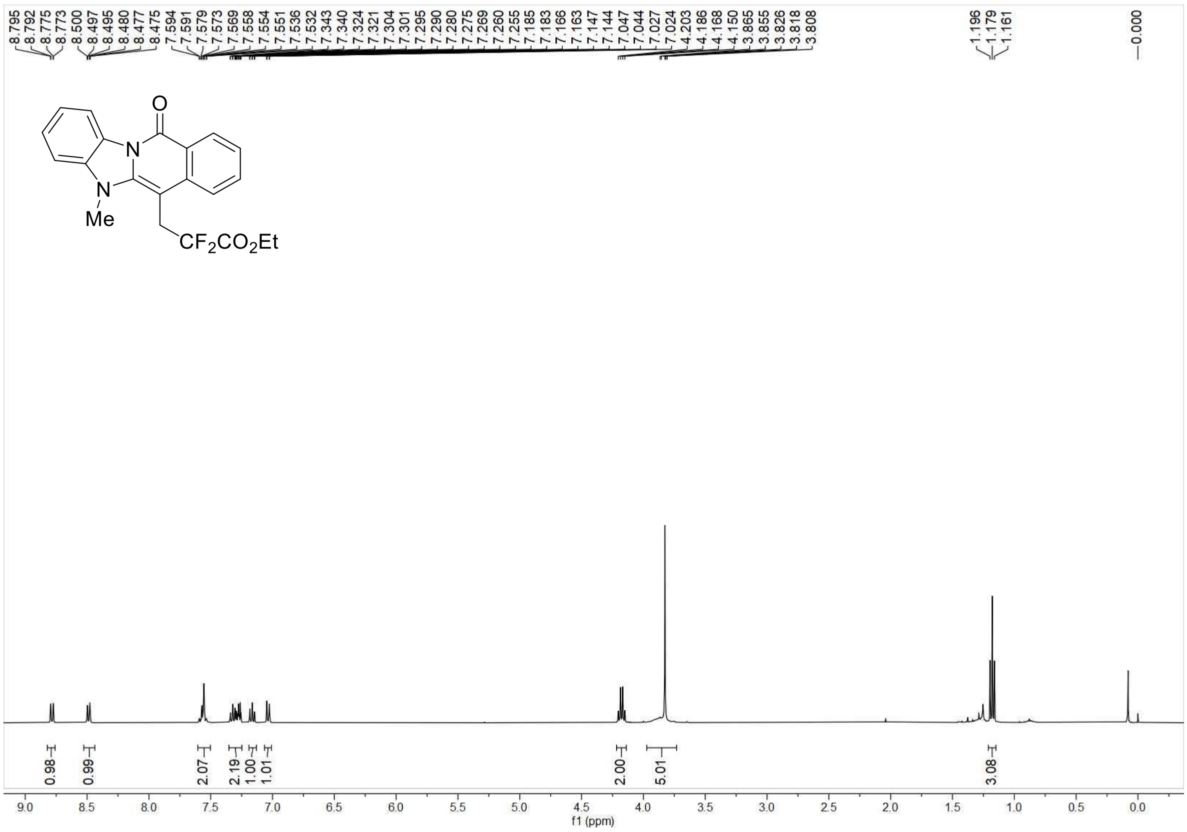

Supplementary Figure 108. ^{1}H-NMR of compound **2**, recorded at 400 MHz and 25 °C in $CDCl_3$.

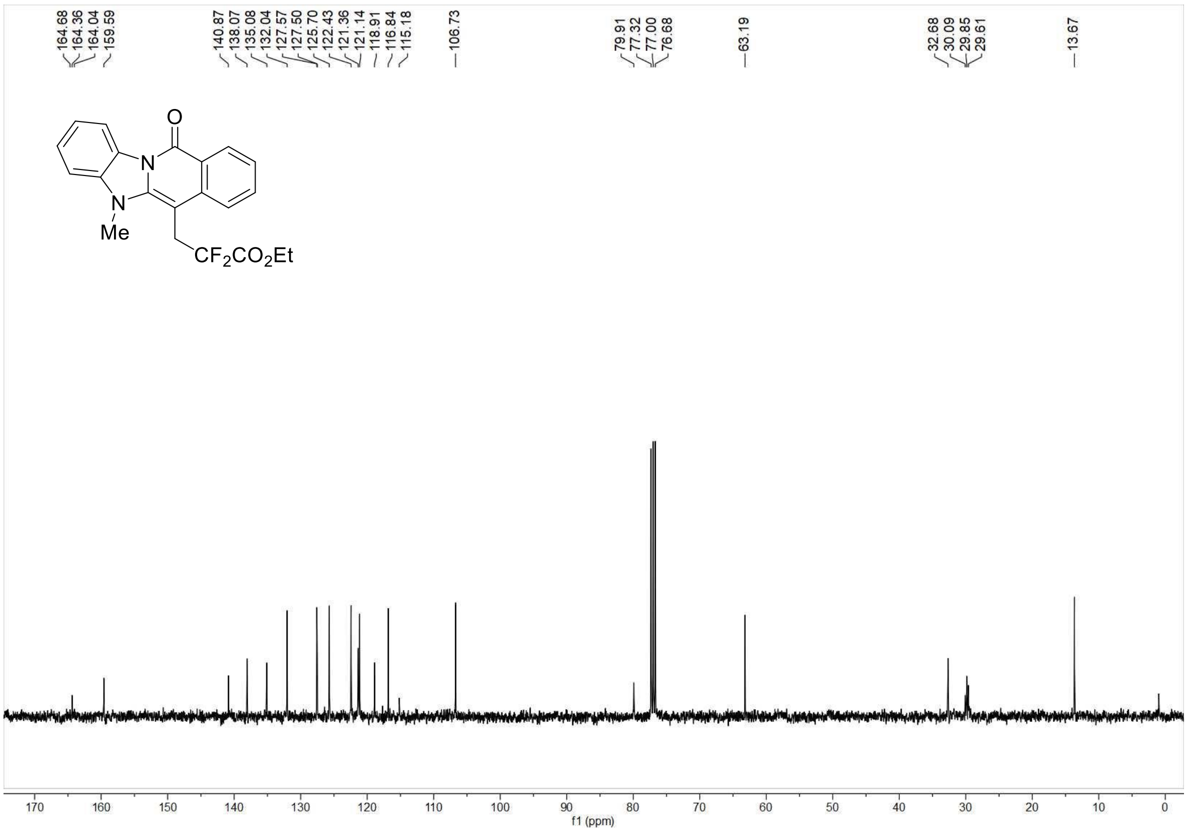

Supplementary Figure 109. ^{13}C-NMR of compound **2**, recorded at 100 MHz and 25 °C in $CDCl_3$.

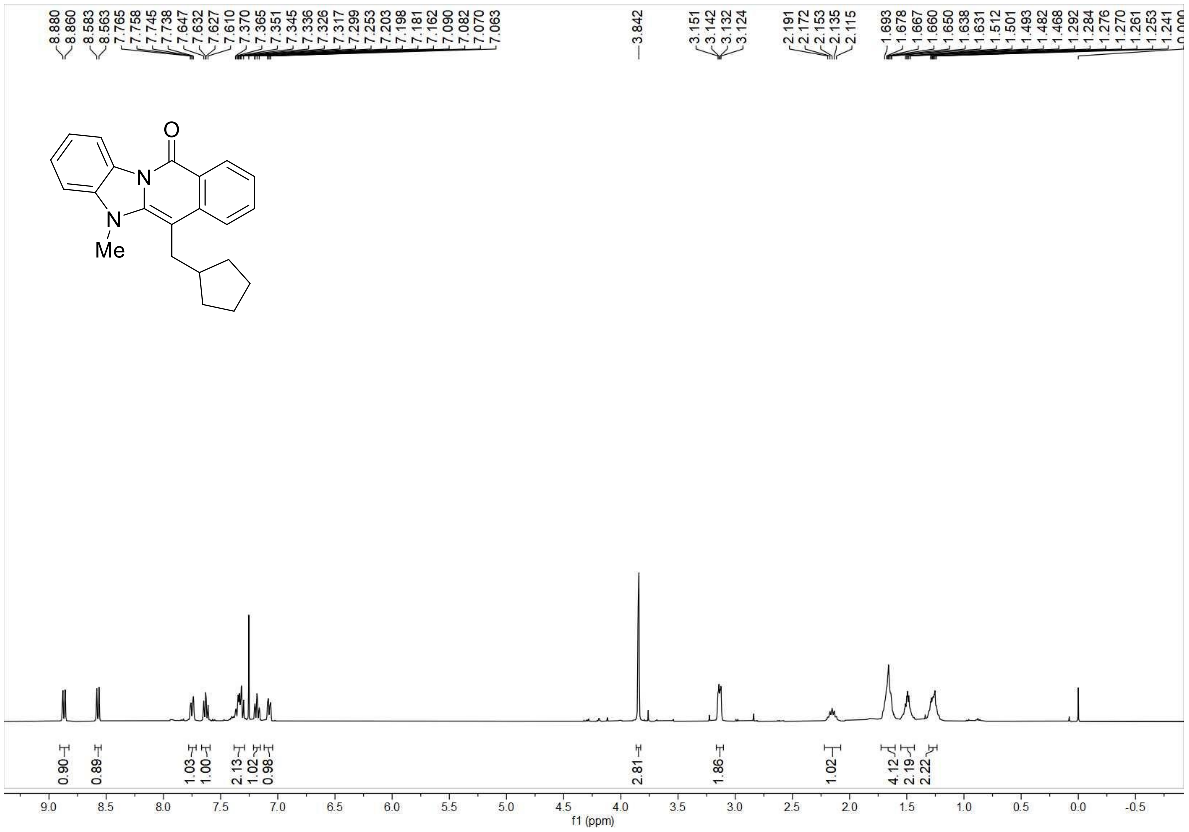

Supplementary Figure 110. ^{1}H-NMR of compound **3**, recorded at 400 MHz and 25 °C in $CDCl_3$.

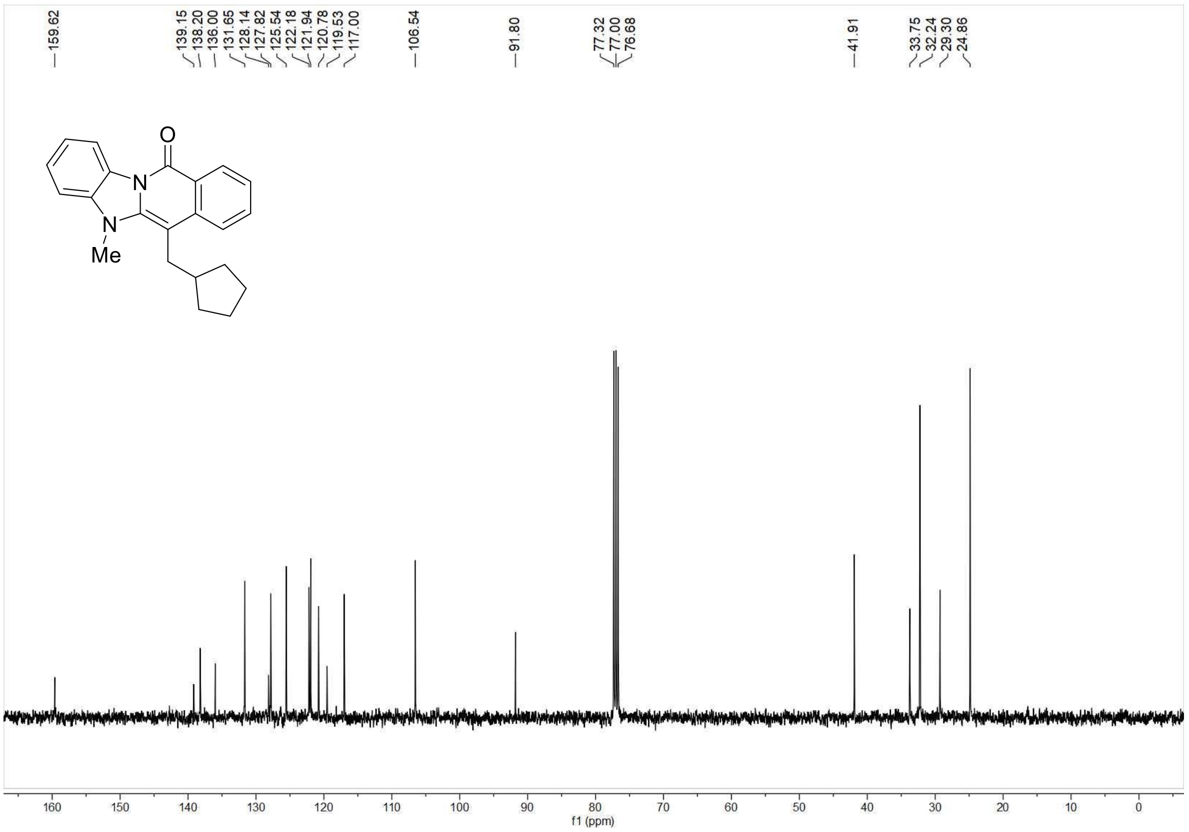

Supplementary Figure 111. ^{13}C-NMR of compound **3**, recorded at 100 MHz and 25 °C in $CDCl_3$.

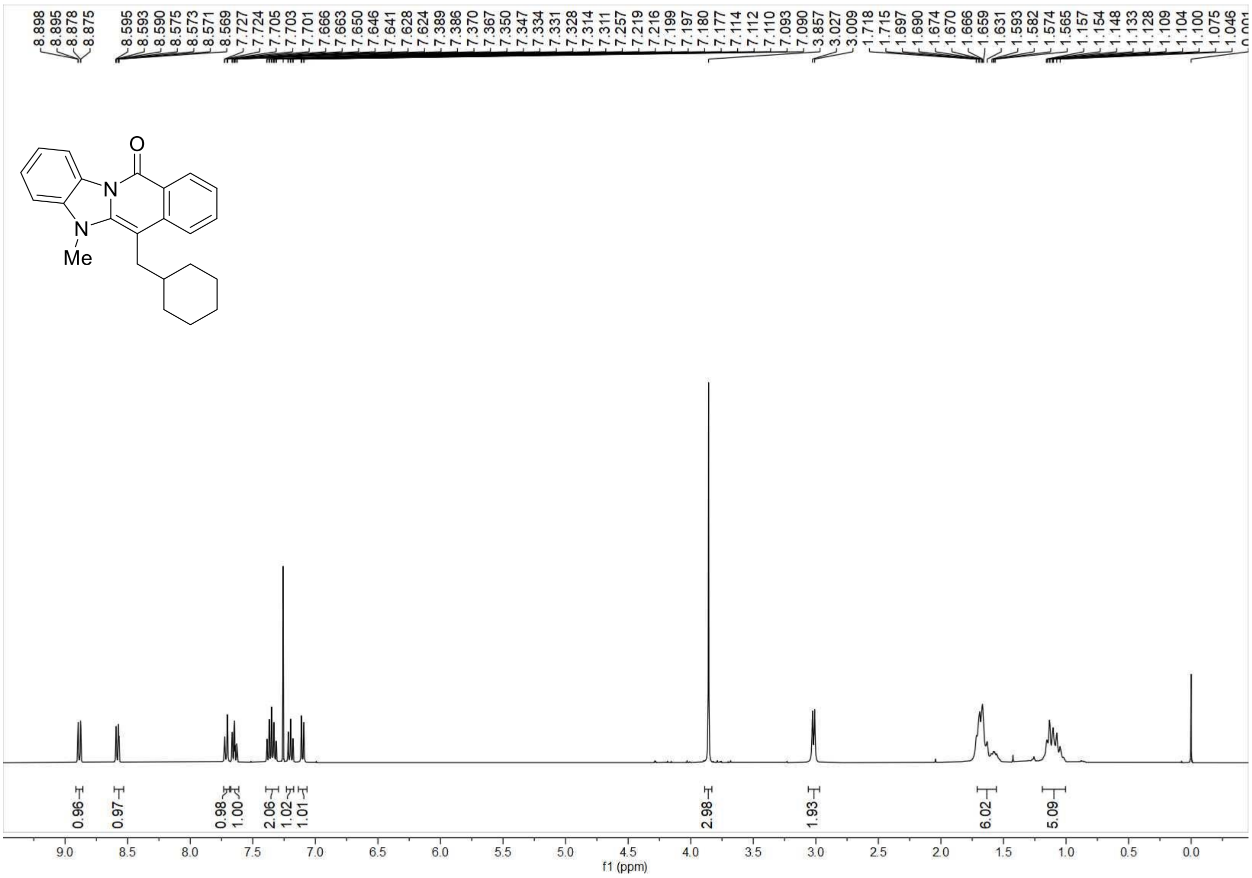

Supplementary Figure 112. ^{1}H-NMR of compound **4**, recorded at 400 MHz and 25 °C in $CDCl_3$.

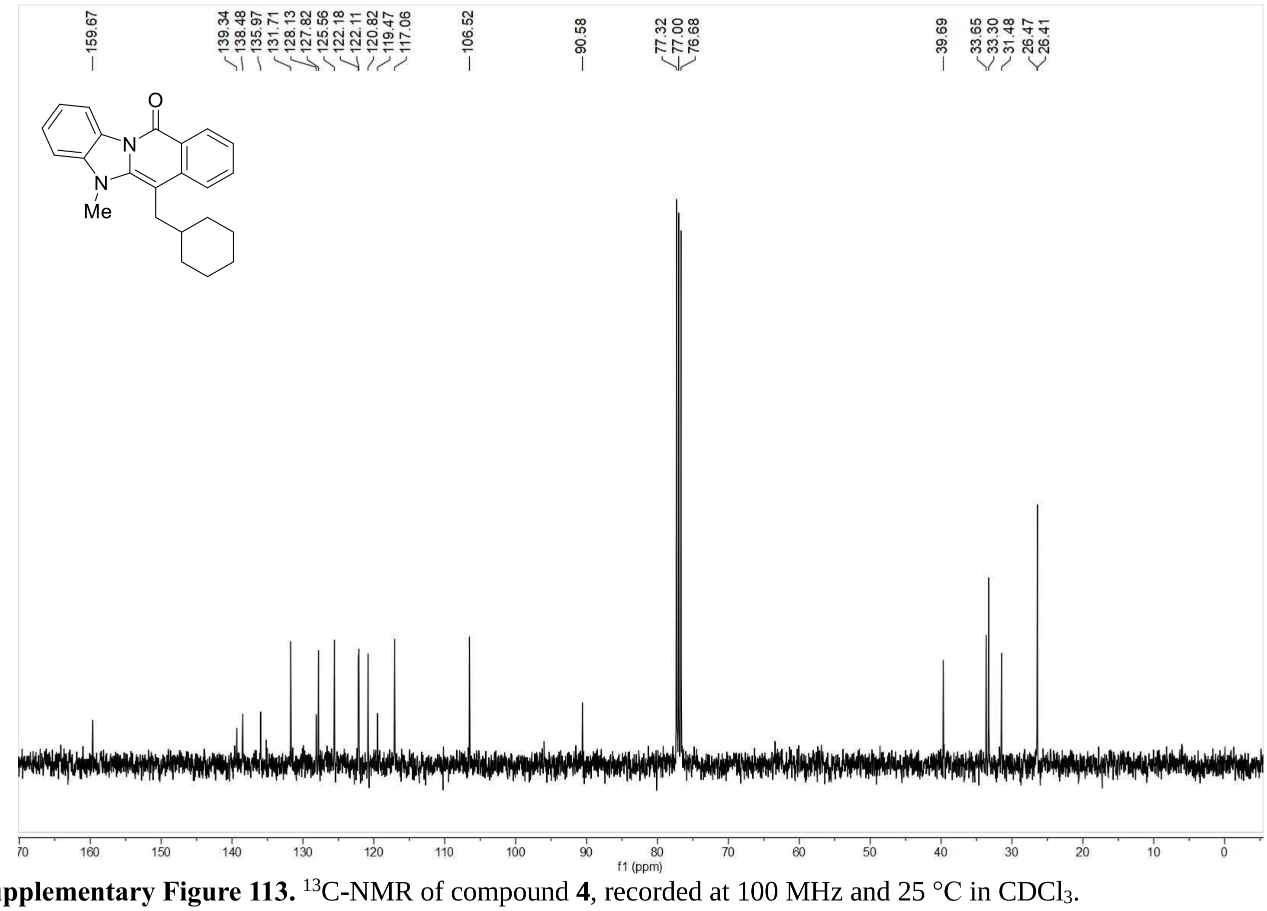

Supplementary Figure 113. ^{13}C-NMR of compound **4**, recorded at 100 MHz and 25 °C in $CDCl_3$.

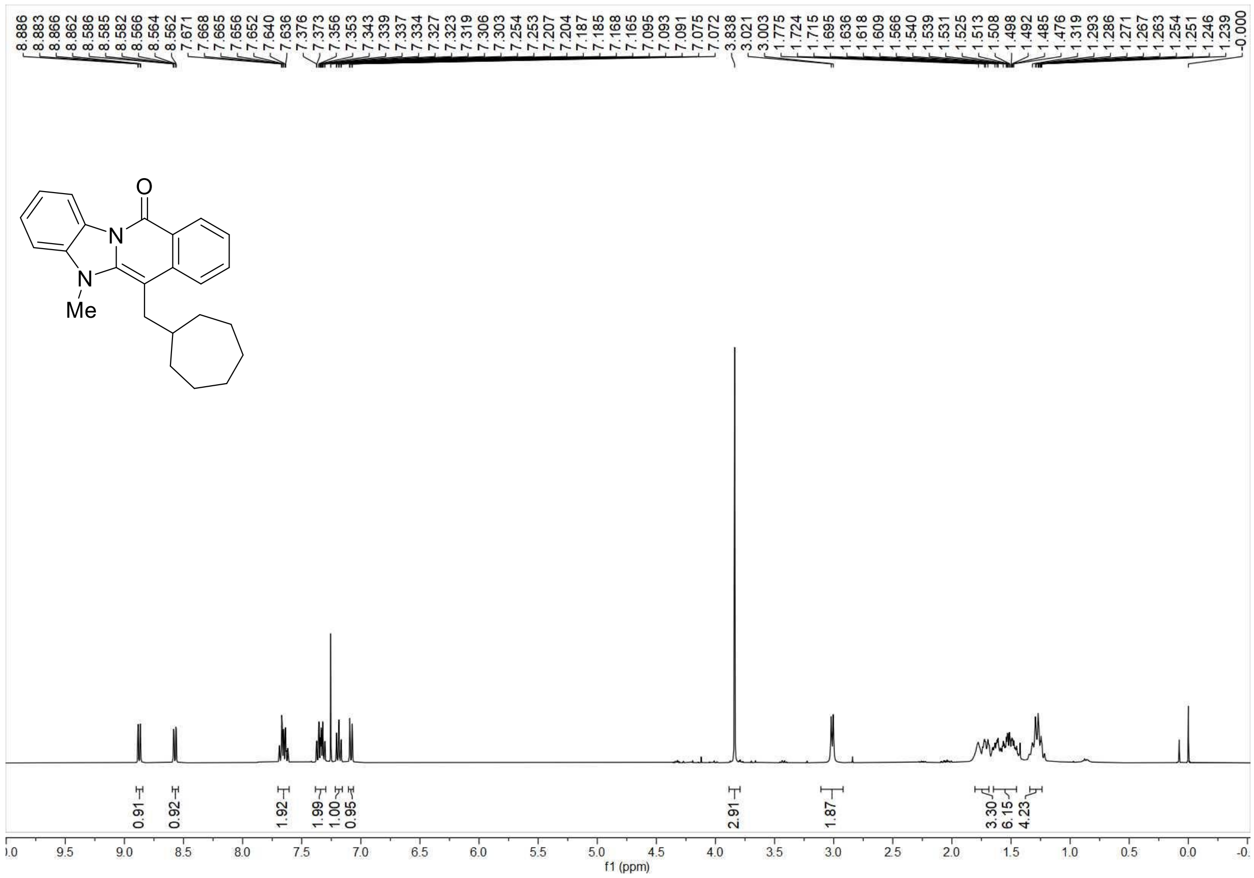

Supplementary Figure 114. ^{1}H-NMR of compound **5**, recorded at 400 MHz and 25 °C in $CDCl_3$.

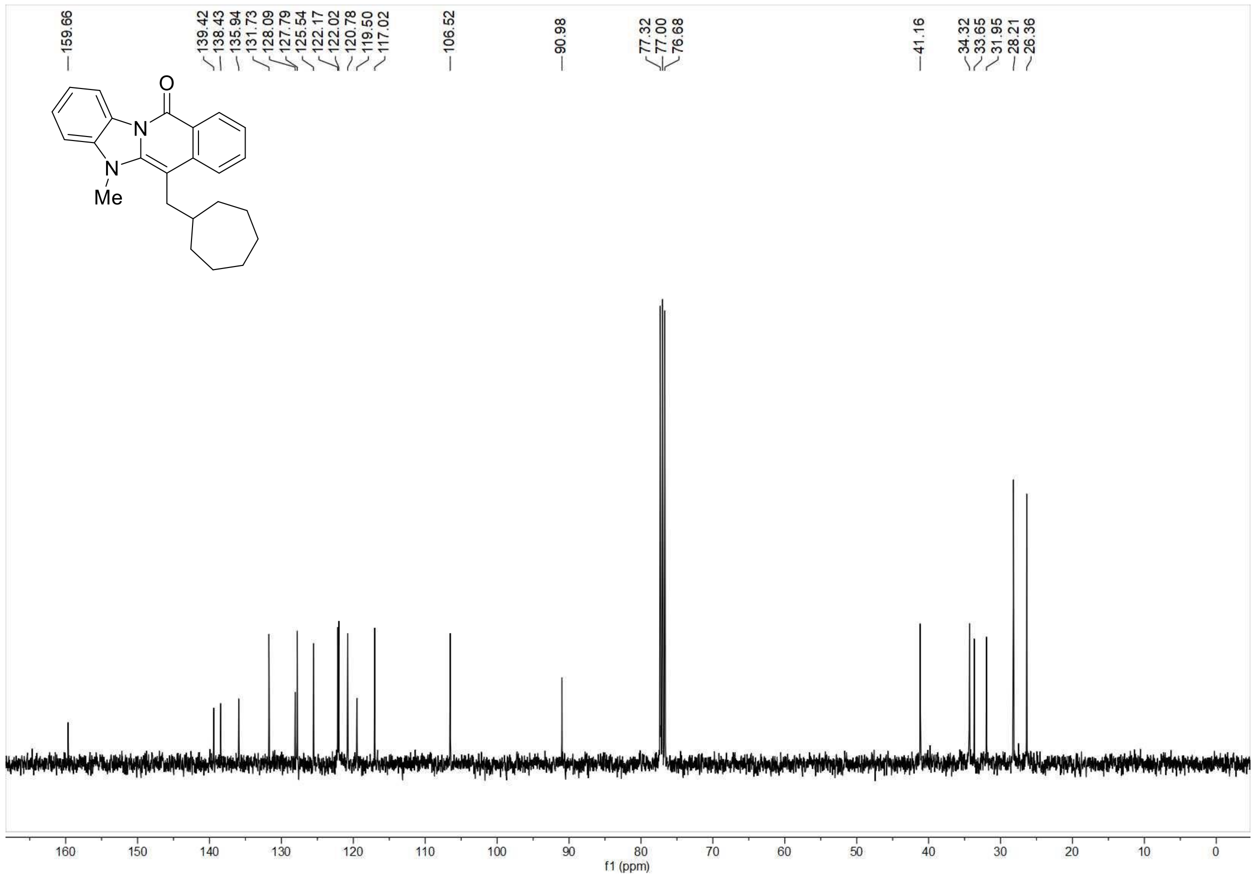

Supplementary Figure 115. ^{13}C-NMR of compound **5**, recorded at 100 MHz and 25 °C in $CDCl_3$.

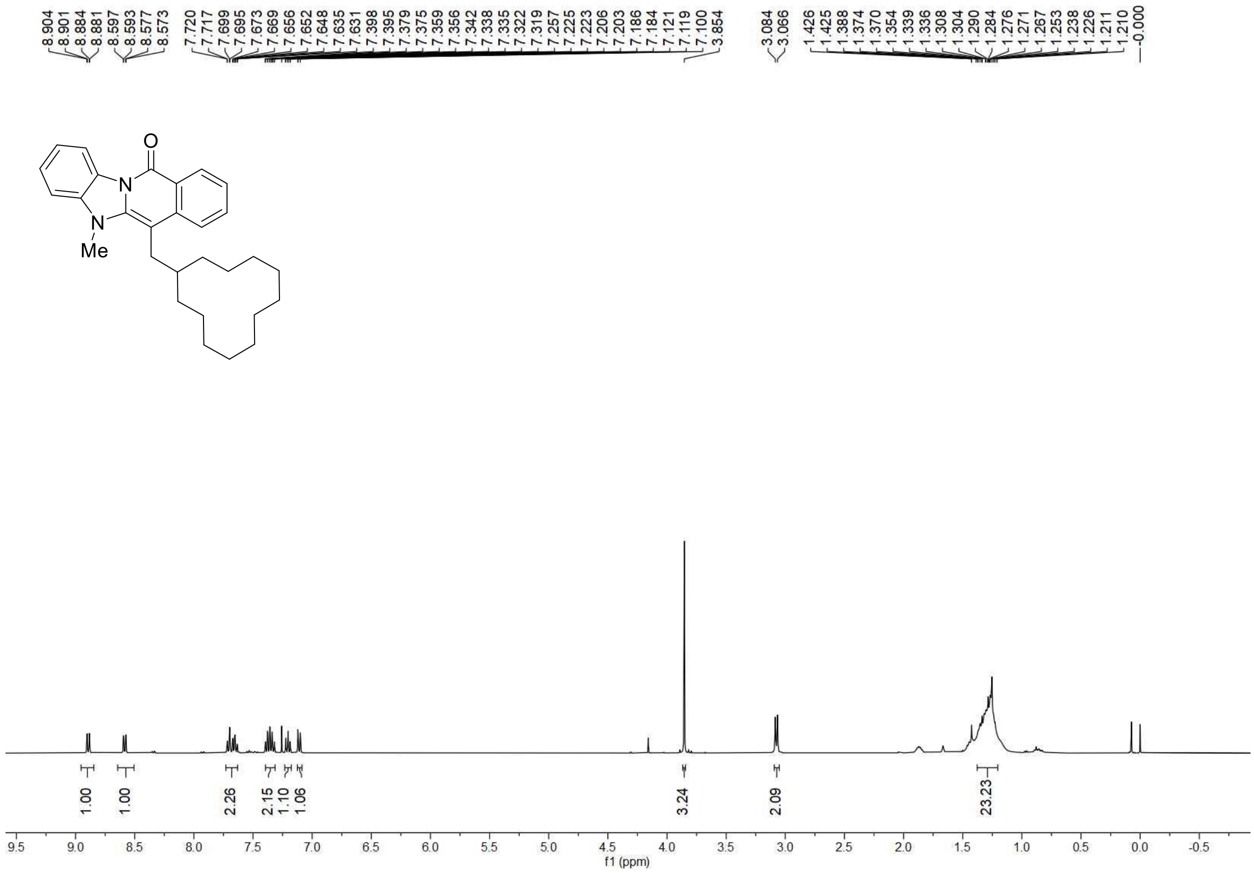

Supplementary Figure 116. ^{1}H-NMR of compound **6**, recorded at 400 MHz and 25 °C in $CDCl_3$.

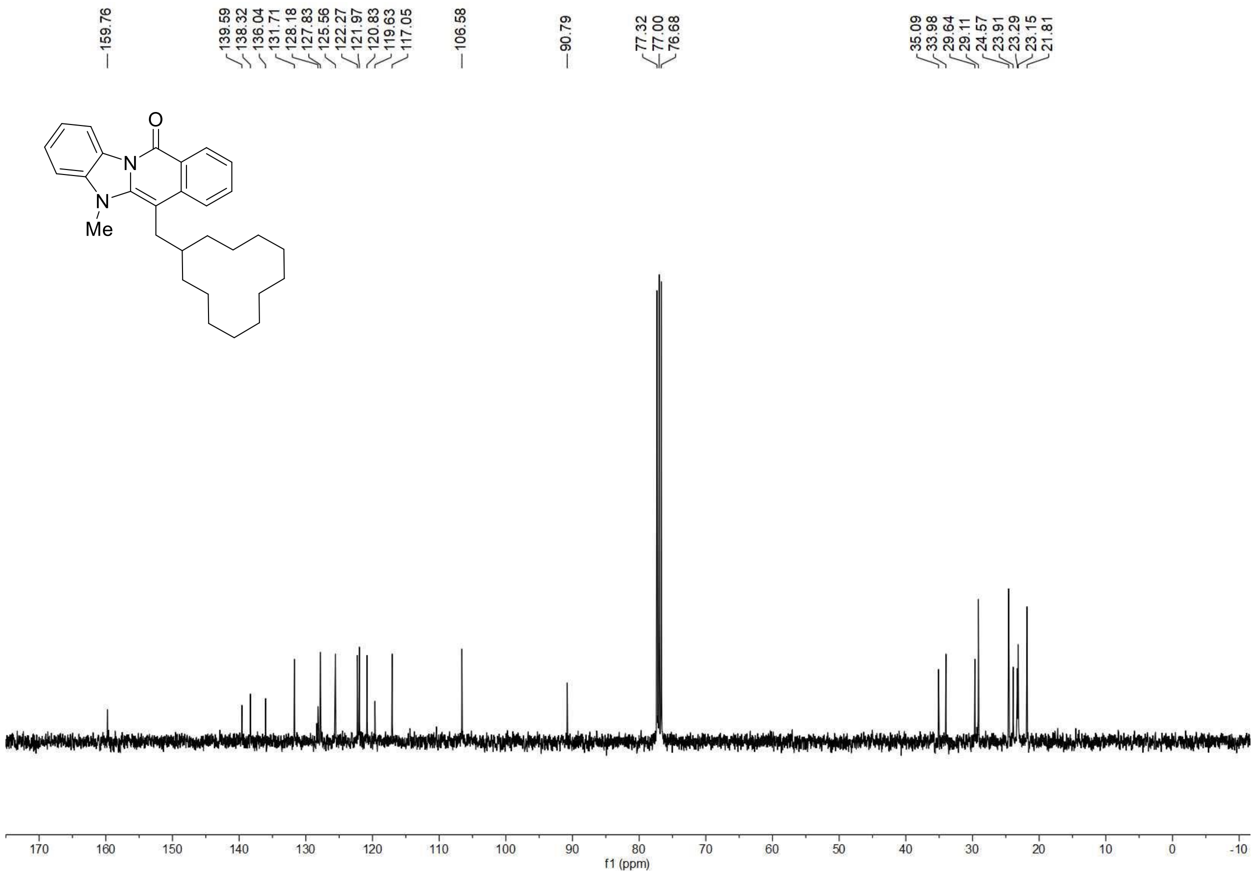

Supplementary Figure 117. ^{13}C-NMR of compound **6**, recorded at 100 MHz and 25 °C in $CDCl_3$.

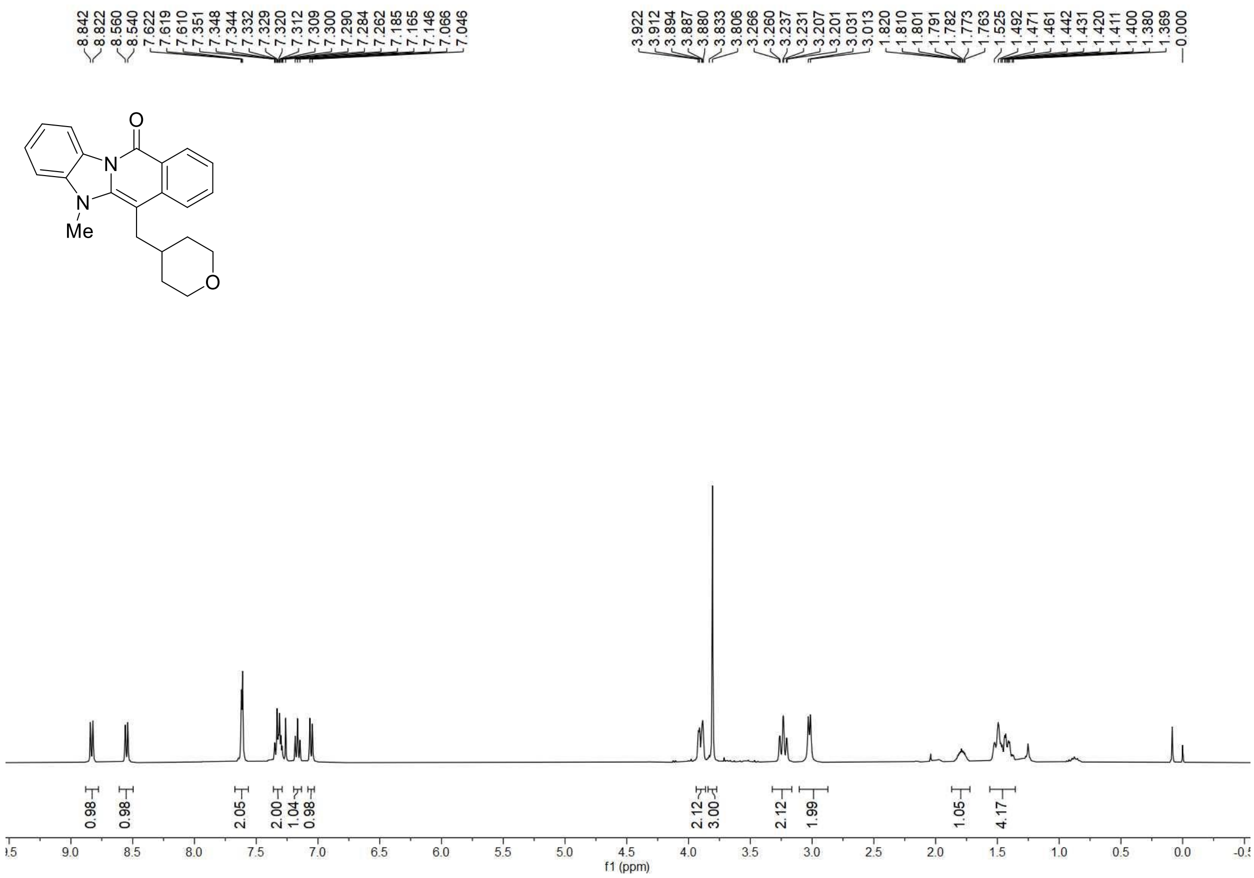

Supplementary Figure 118. ^{1}H-NMR of compound **7**, recorded at 400 MHz and 25 °C in $CDCl_3$.

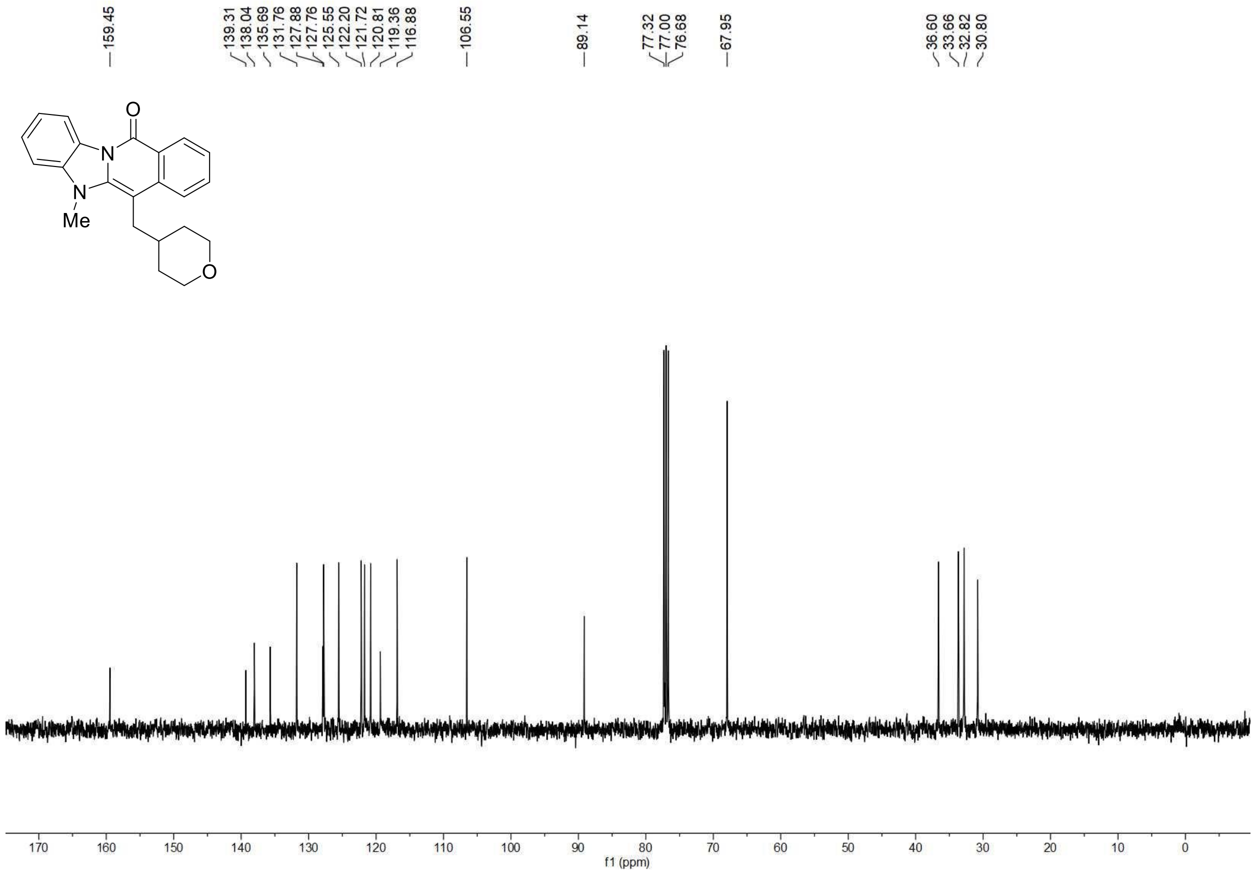

Supplementary Figure 119. ^{13}C-NMR of compound **7**, recorded at 100 MHz and 25 °C in $CDCl_3$.

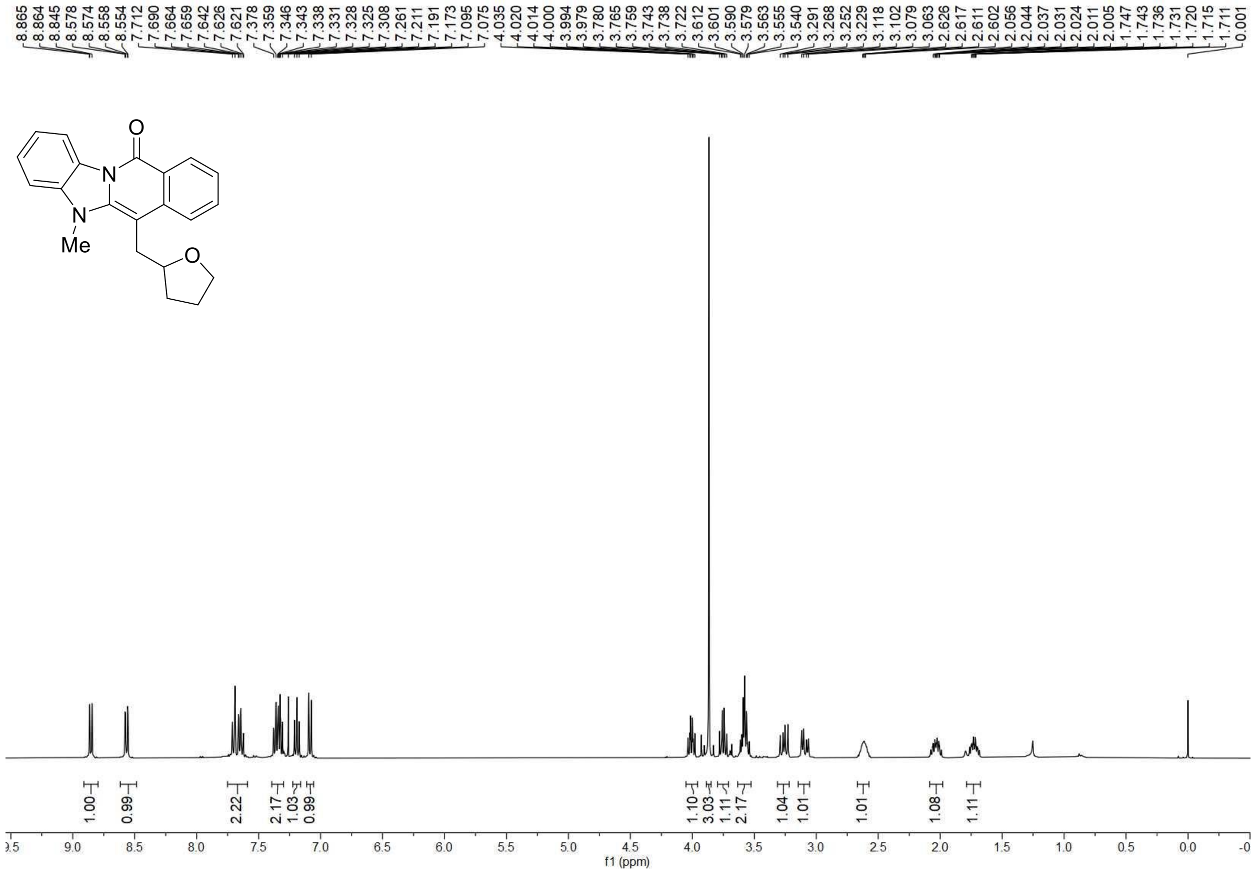

Supplementary Figure 120. ^{1}H-NMR of compound **8**, recorded at 400 MHz and 25 °C in $CDCl_3$.

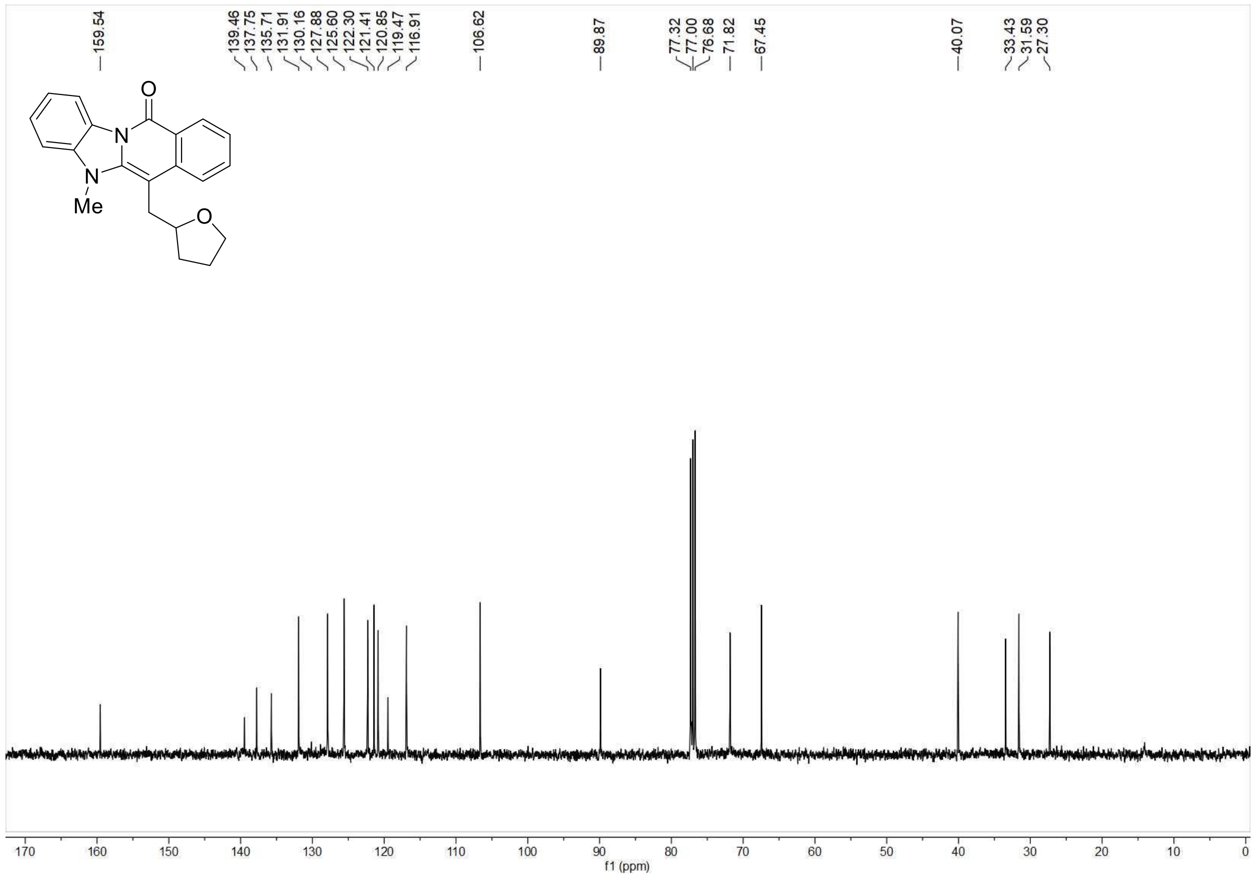

Supplementary Figure 121. ^{13}C-NMR of compound **8**, recorded at 100 MHz and 25 °C in $CDCl_3$.

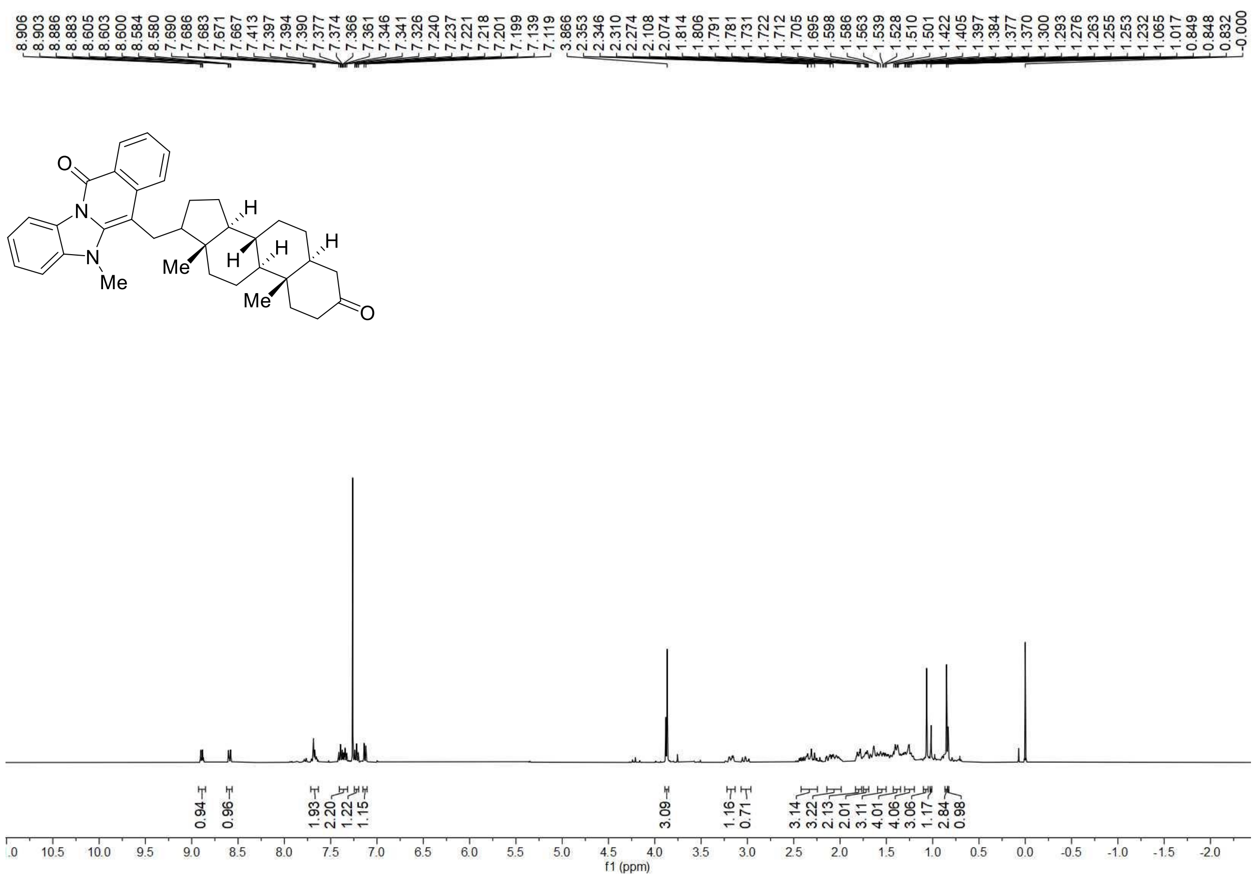

Supplementary Figure 122. ^{1}H-NMR of compound **9**, recorded at 400 MHz and 25 °C in $CDCl_3$.

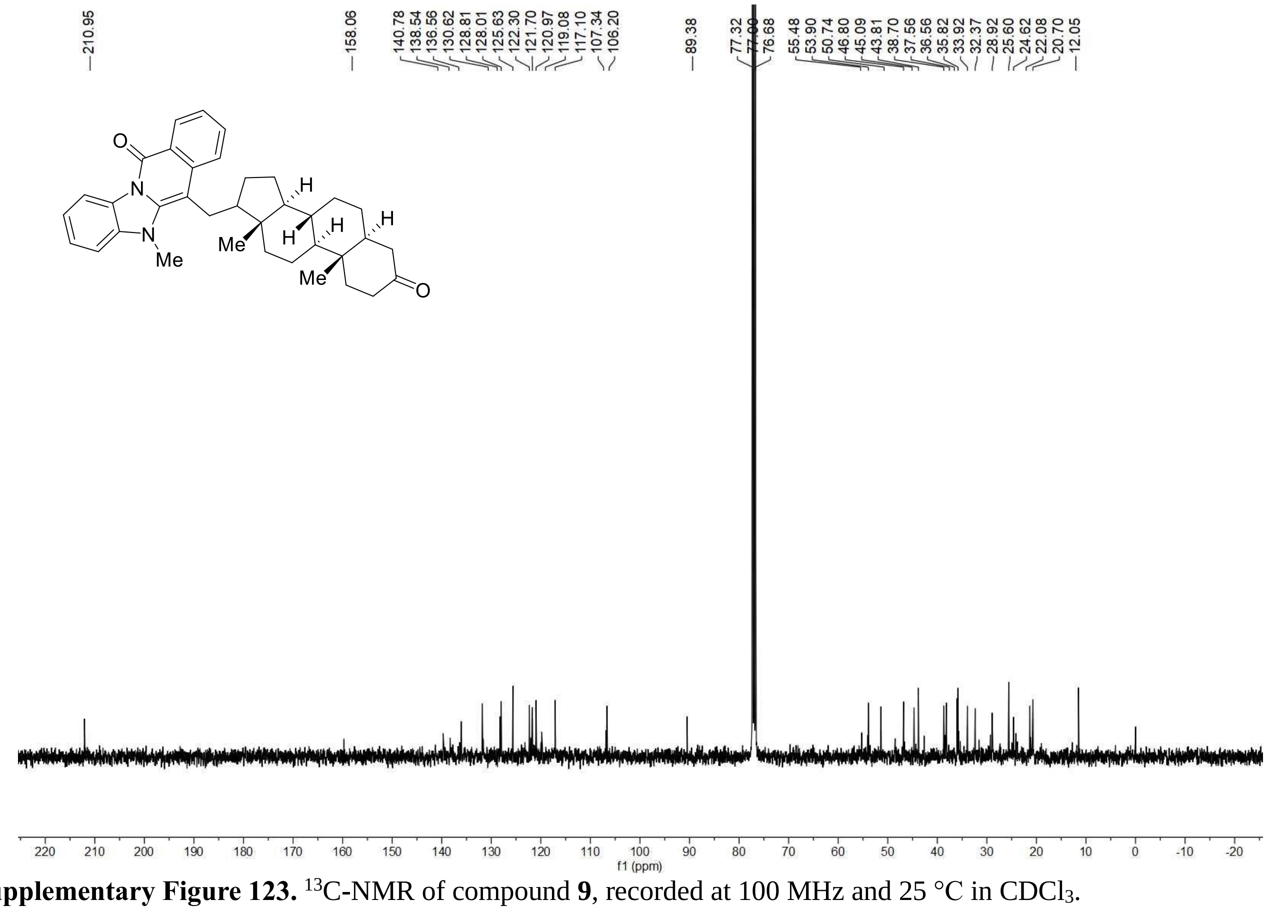

Supplementary Figure 123. ^{13}C-NMR of compound **9**, recorded at 100 MHz and 25 °C in $CDCl_3$.

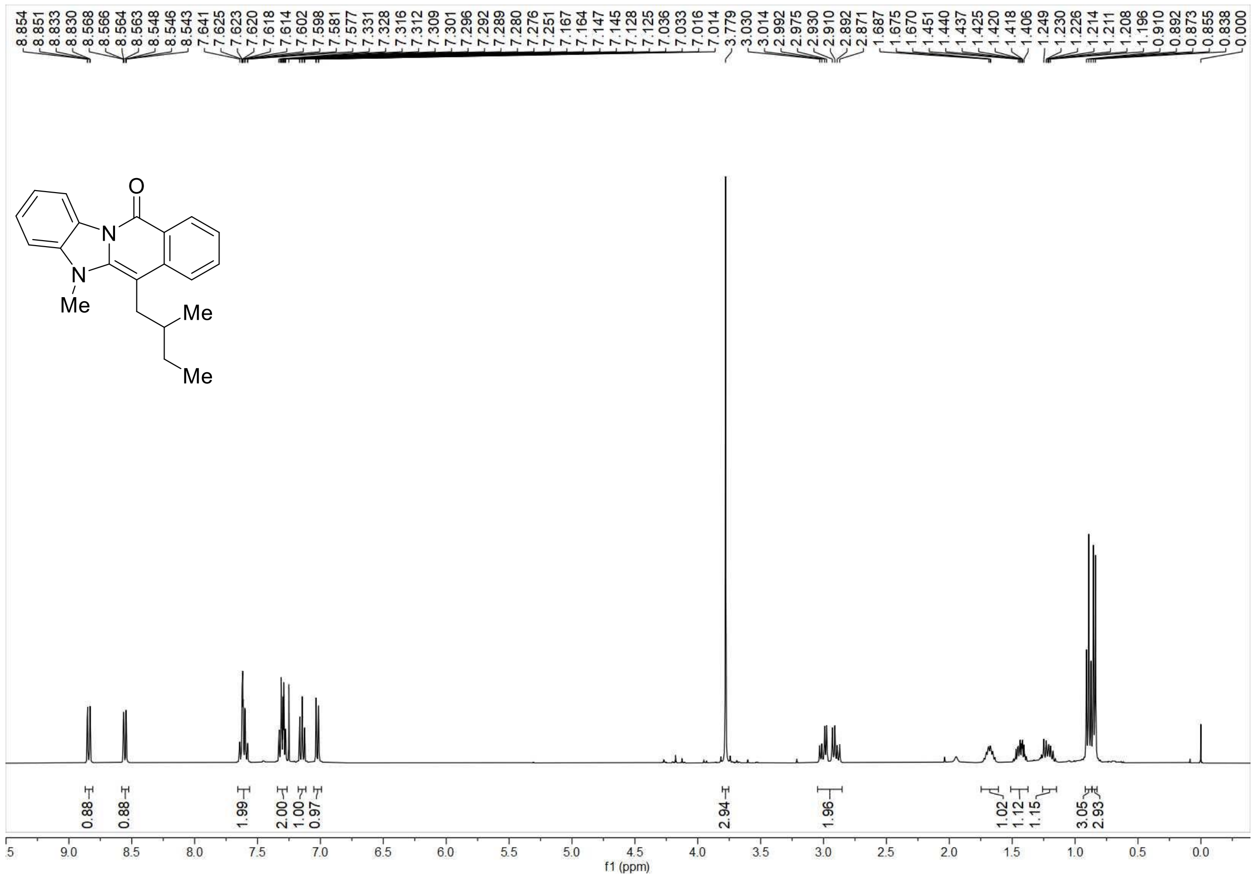

Supplementary Figure 124. ^{1}H-NMR of compound **10**, recorded at 400 MHz and 25 °C in $CDCl_3$.

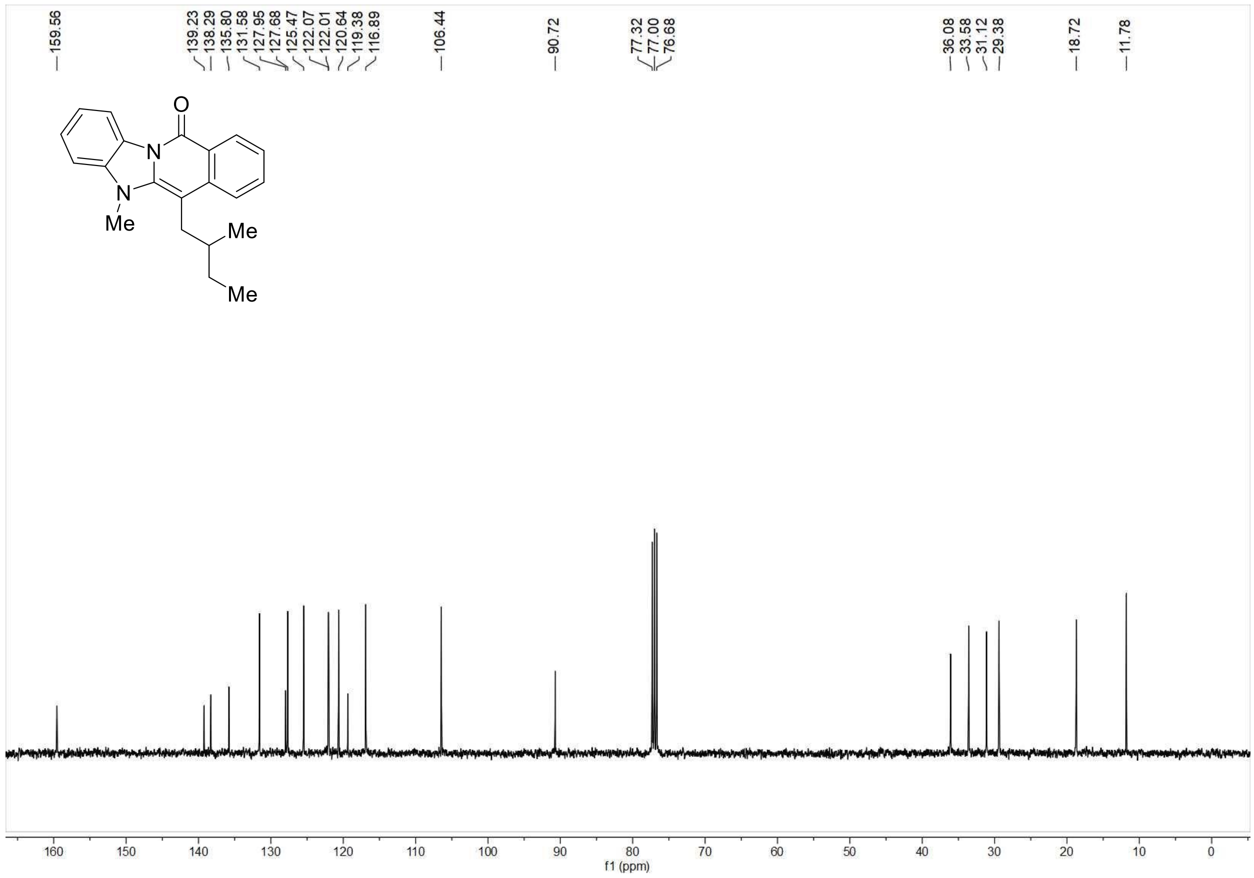

Supplementary Figure 125. ^{13}C-NMR of compound **10**, recorded at 100 MHz and 25 °C in $CDCl_3$.

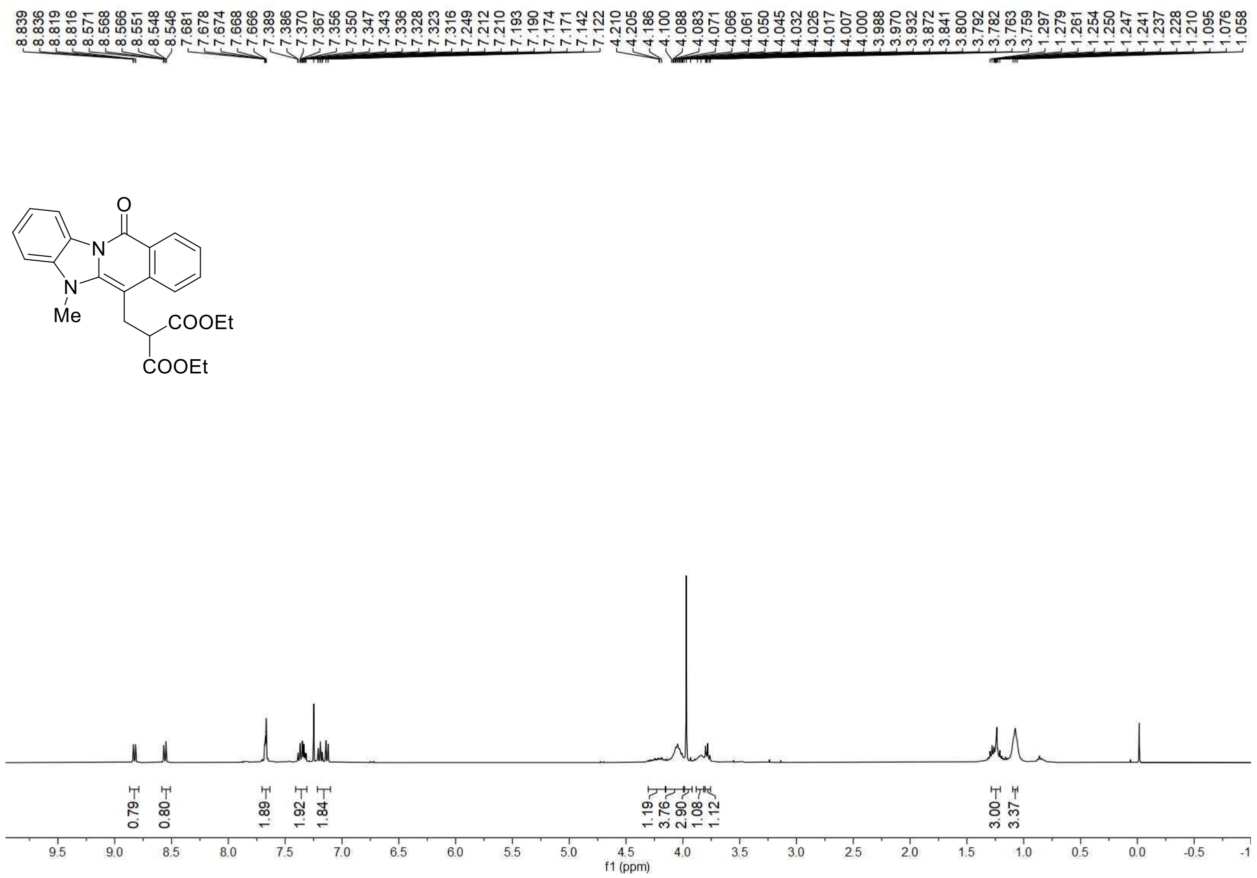

Supplementary Figure 126. ^{1}H-NMR of compound **11**, recorded at 400 MHz and 25 °C in $CDCl_3$.

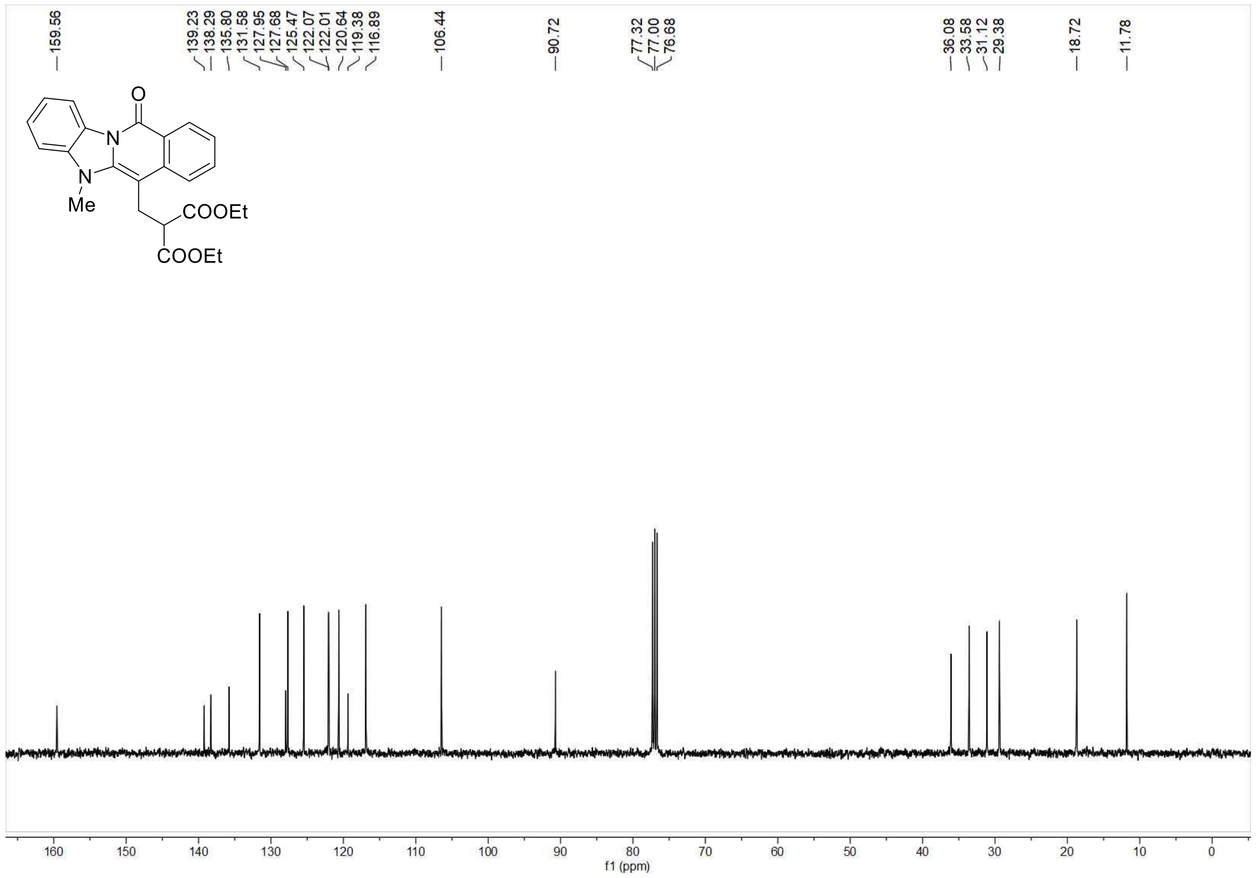

Supplementary Figure 127. ^{13}C-NMR of compound **11**, recorded at 100 MHz and 25 °C in $CDCl_3$.

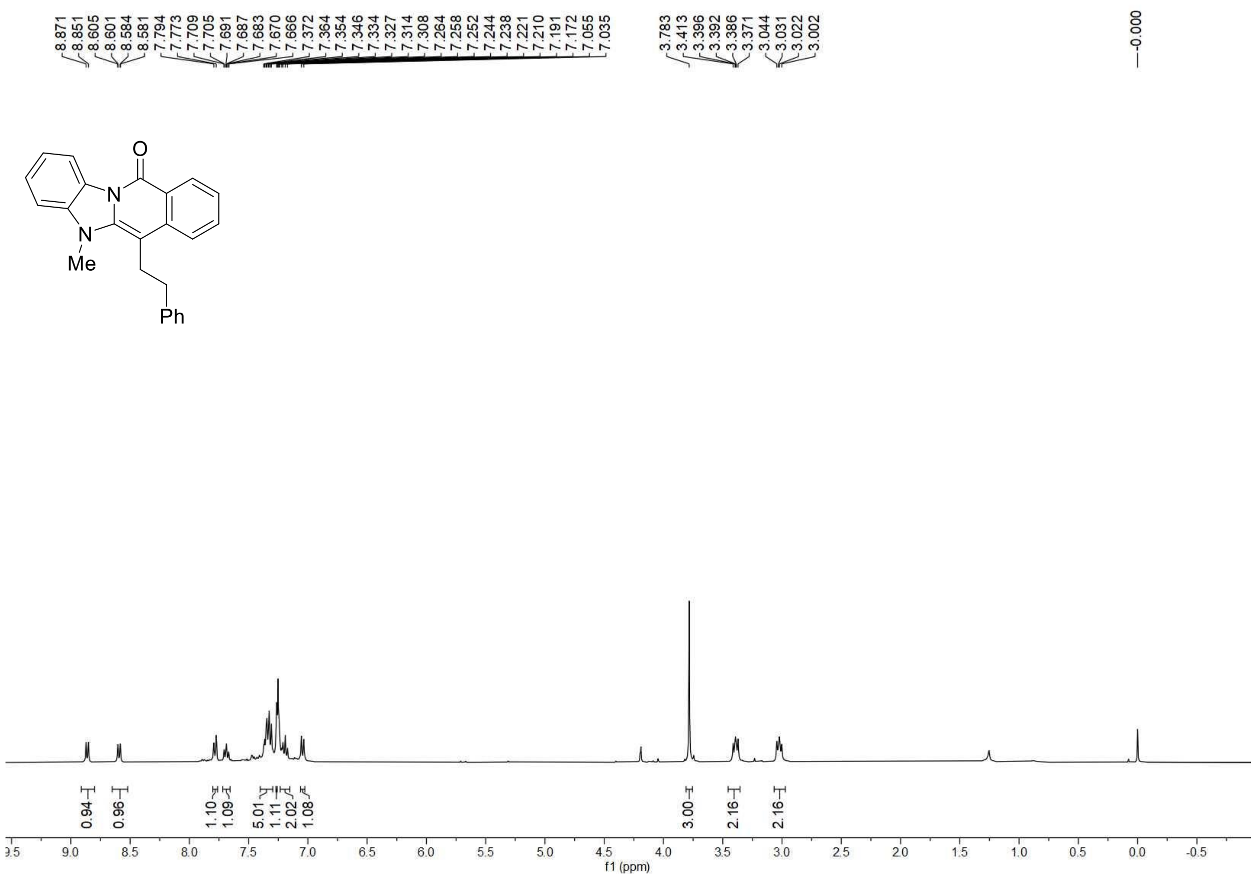

Supplementary Figure 128. ^{1}H-NMR of compound **12**, recorded at 400 MHz and 25 °C in $CDCl_3$.

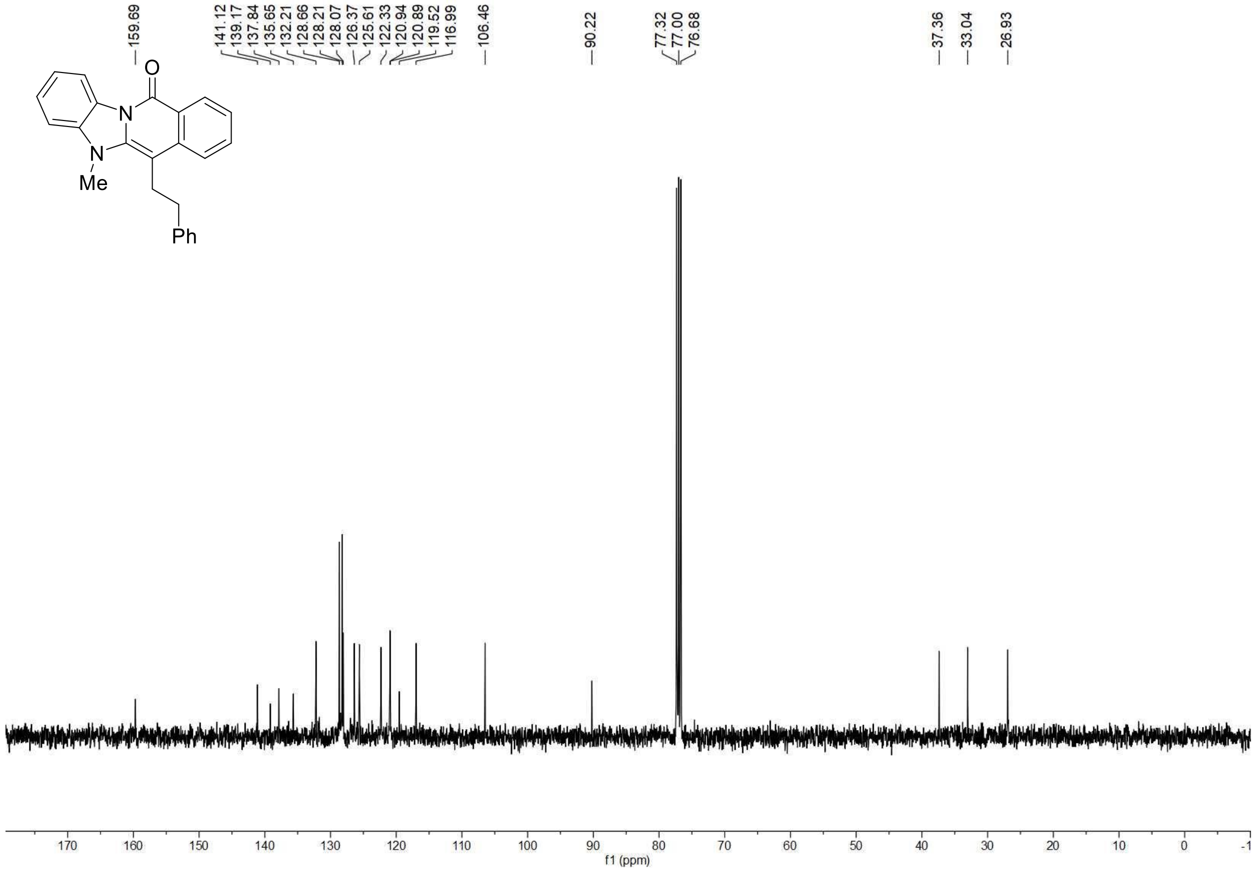

Supplementary Figure 129. ^{13}C-NMR of compound **12**, recorded at 100 MHz and 25 °C in $CDCl_3$.

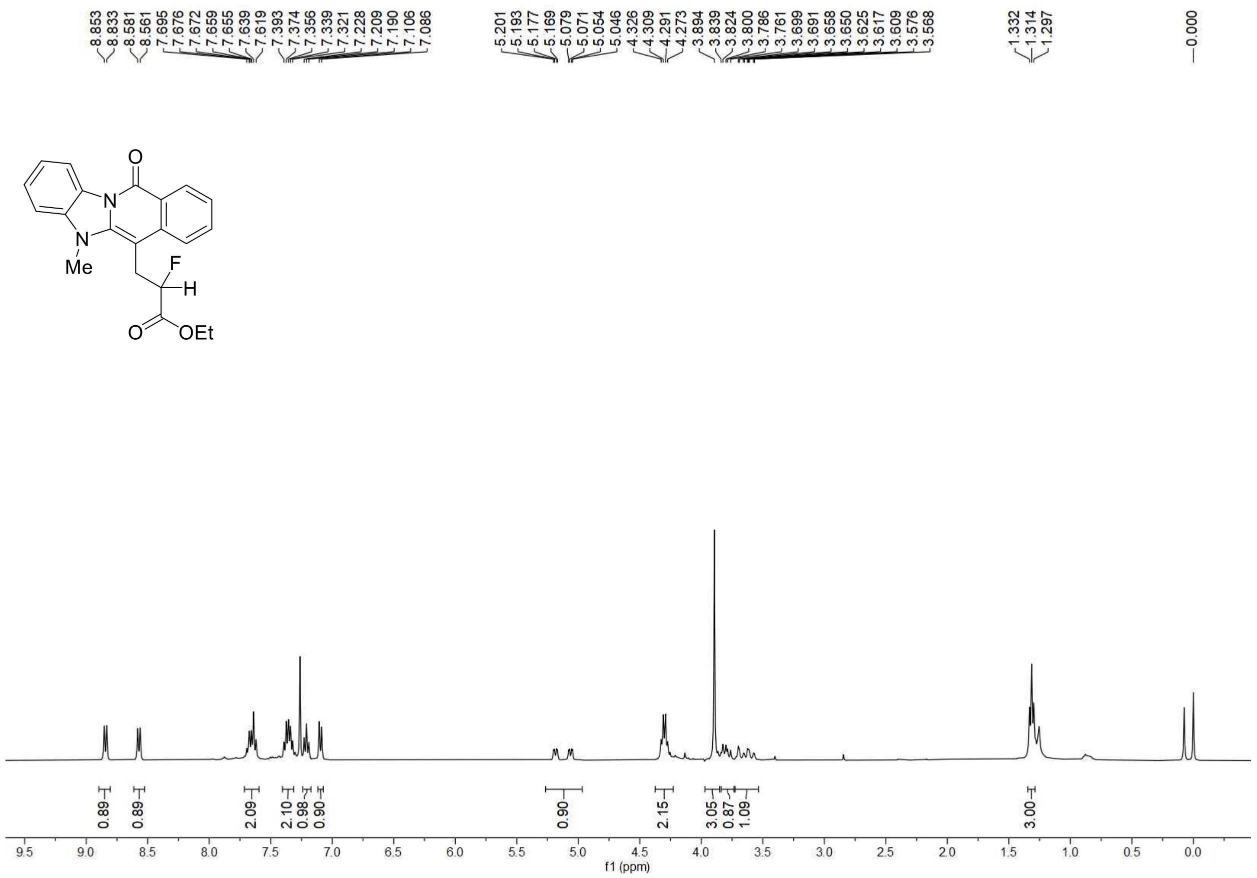

Supplementary Figure 130. ^{1}H-NMR of compound **13**, recorded at 400 MHz and 25 °C in $CDCl_3$.

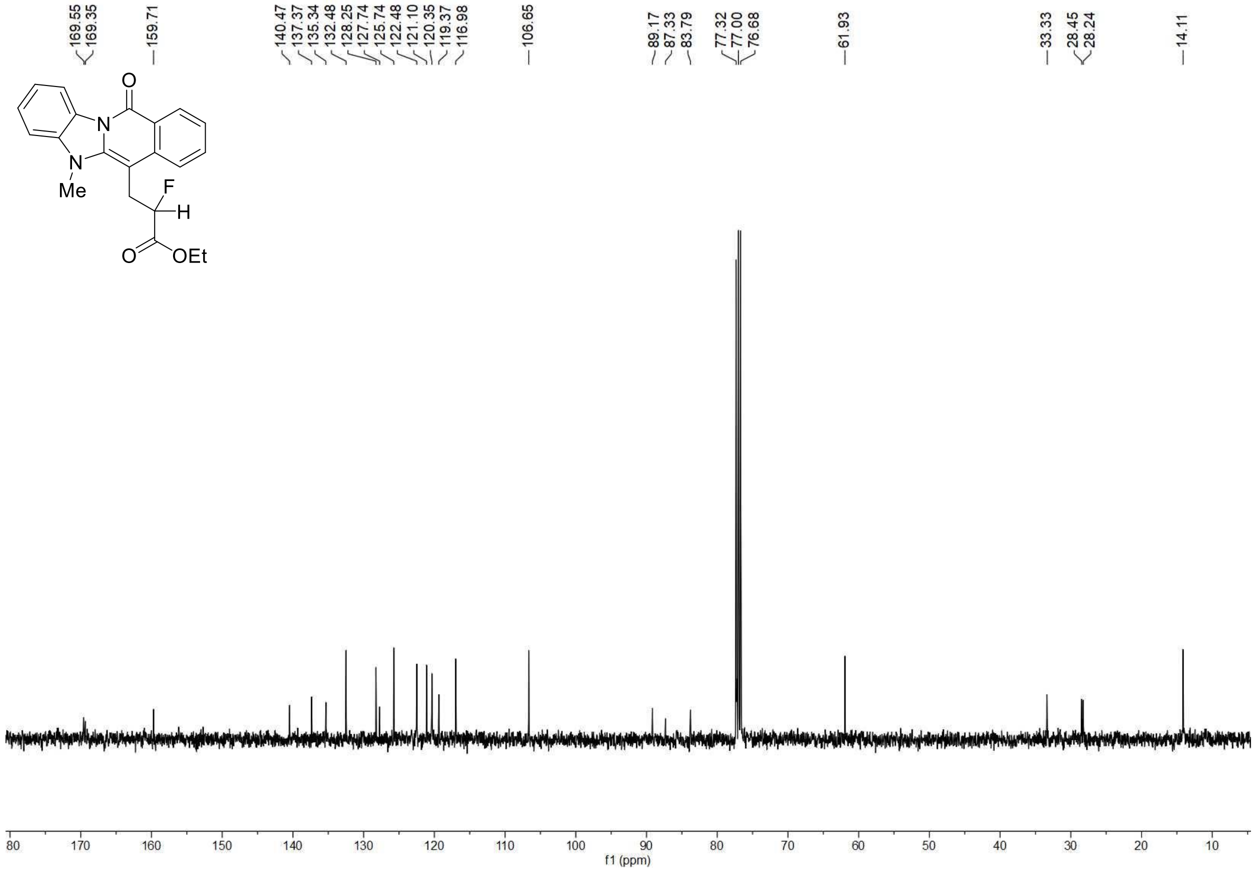

Supplementary Figure 131. ^{13}C-NMR of compound **13**, recorded at 100 MHz and 25 °C in $CDCl_3$.

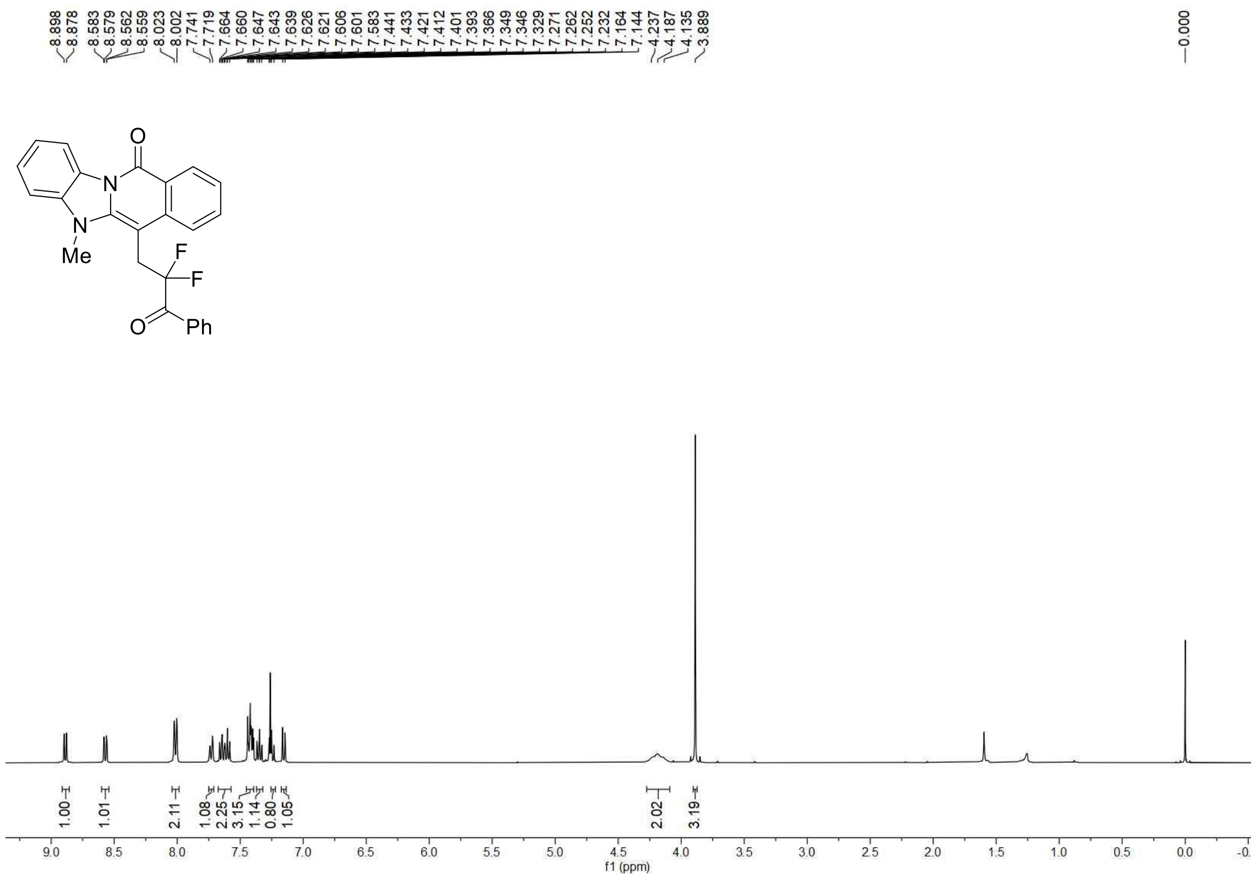

Supplementary Figure 132. ^{1}H-NMR of compound **14**, recorded at 400 MHz and 25 °C in $CDCl_3$.

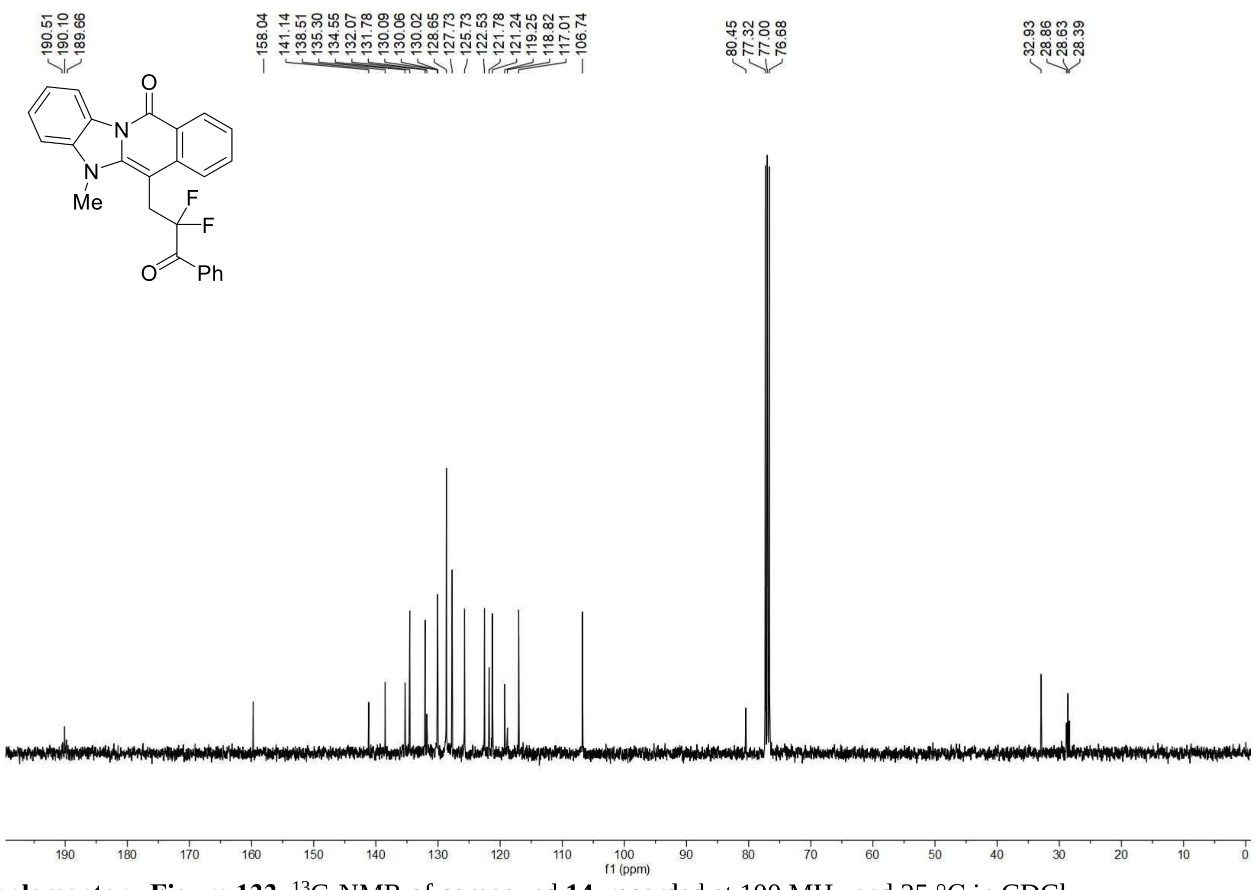

Supplementary Figure 133. ^{13}C-NMR of compound **14**, recorded at 100 MHz and 25 °C in $CDCl_3$.

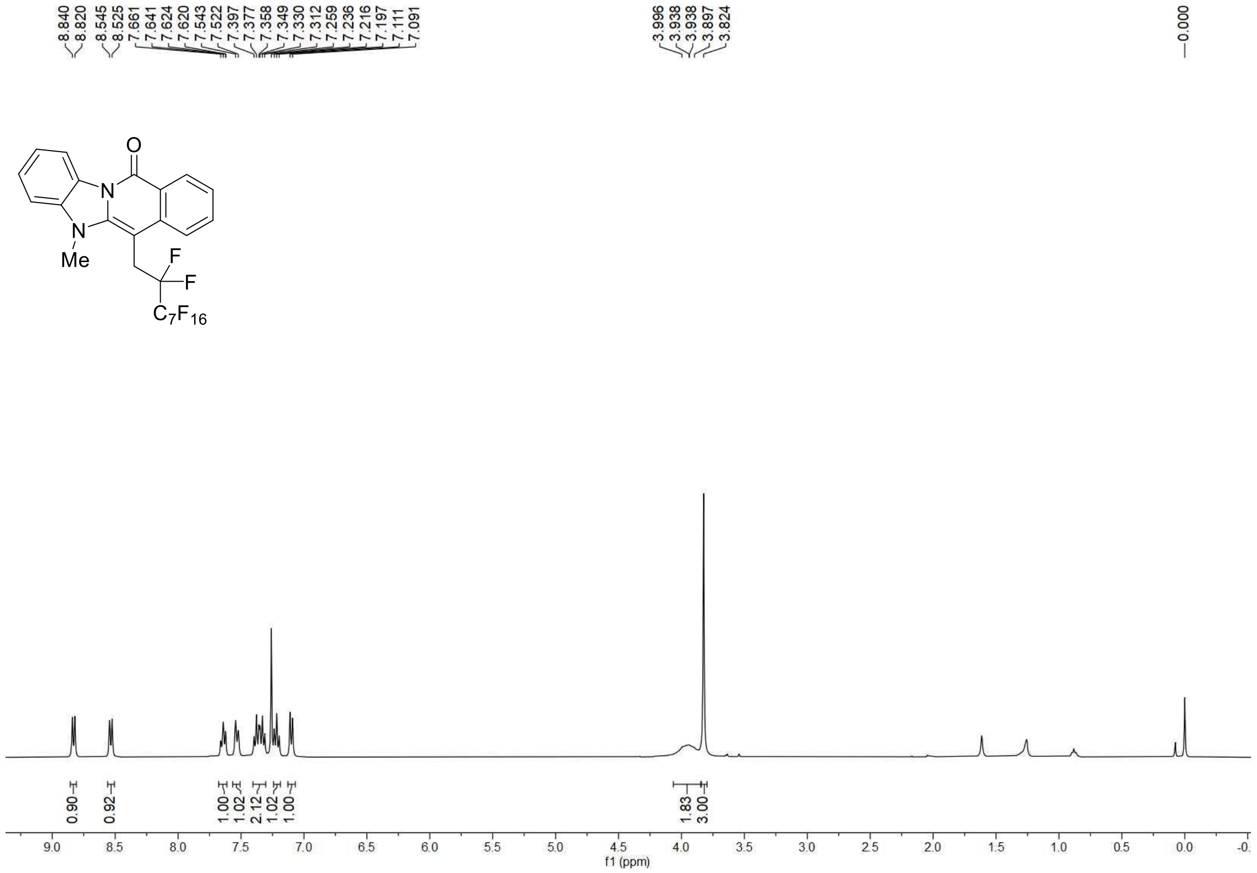

Supplementary Figure 134. ^{1}H-NMR of compound **15**, recorded at 400 MHz and 25 °C in $CDCl_3$.

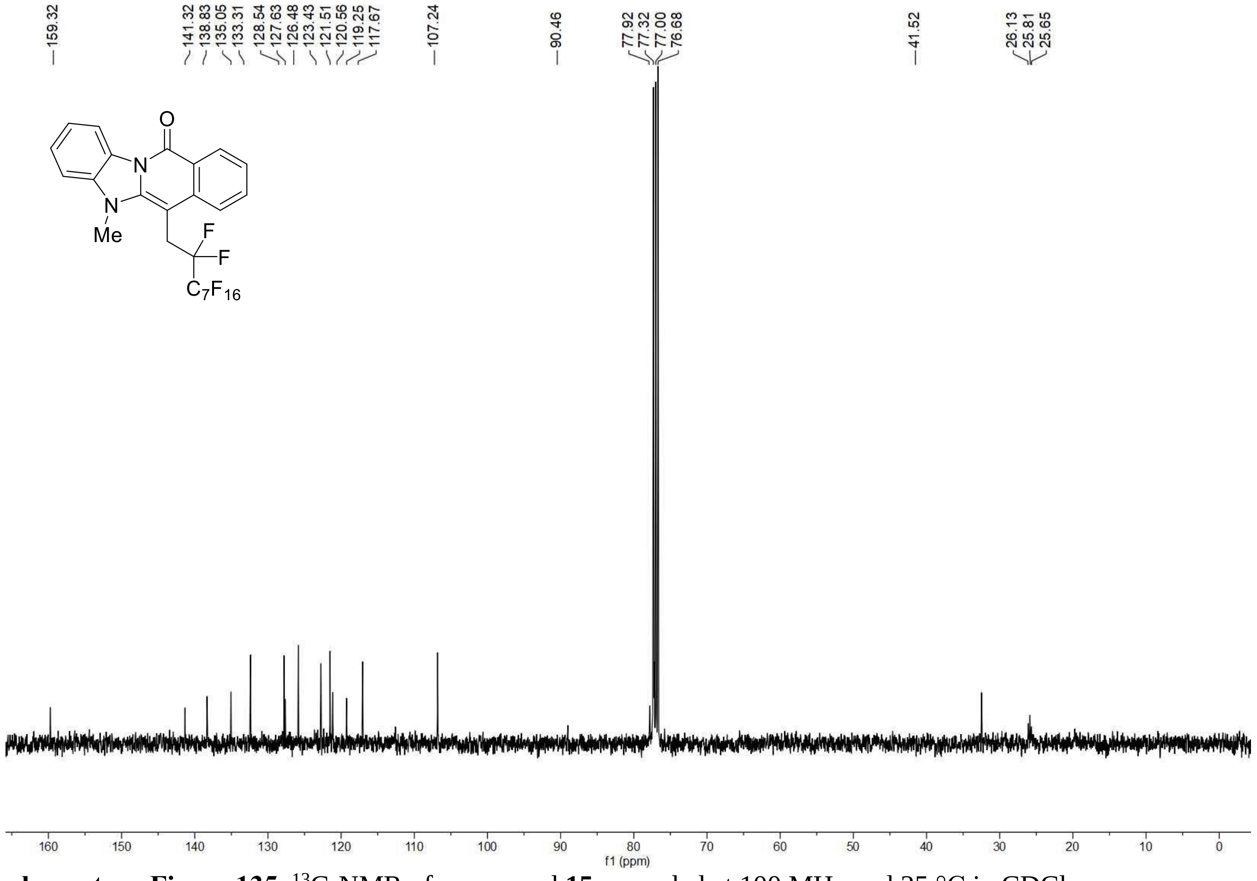

Supplementary Figure 135. ^{13}C-NMR of compound **15**, recorded at 100 MHz and 25 °C in $CDCl_3$.

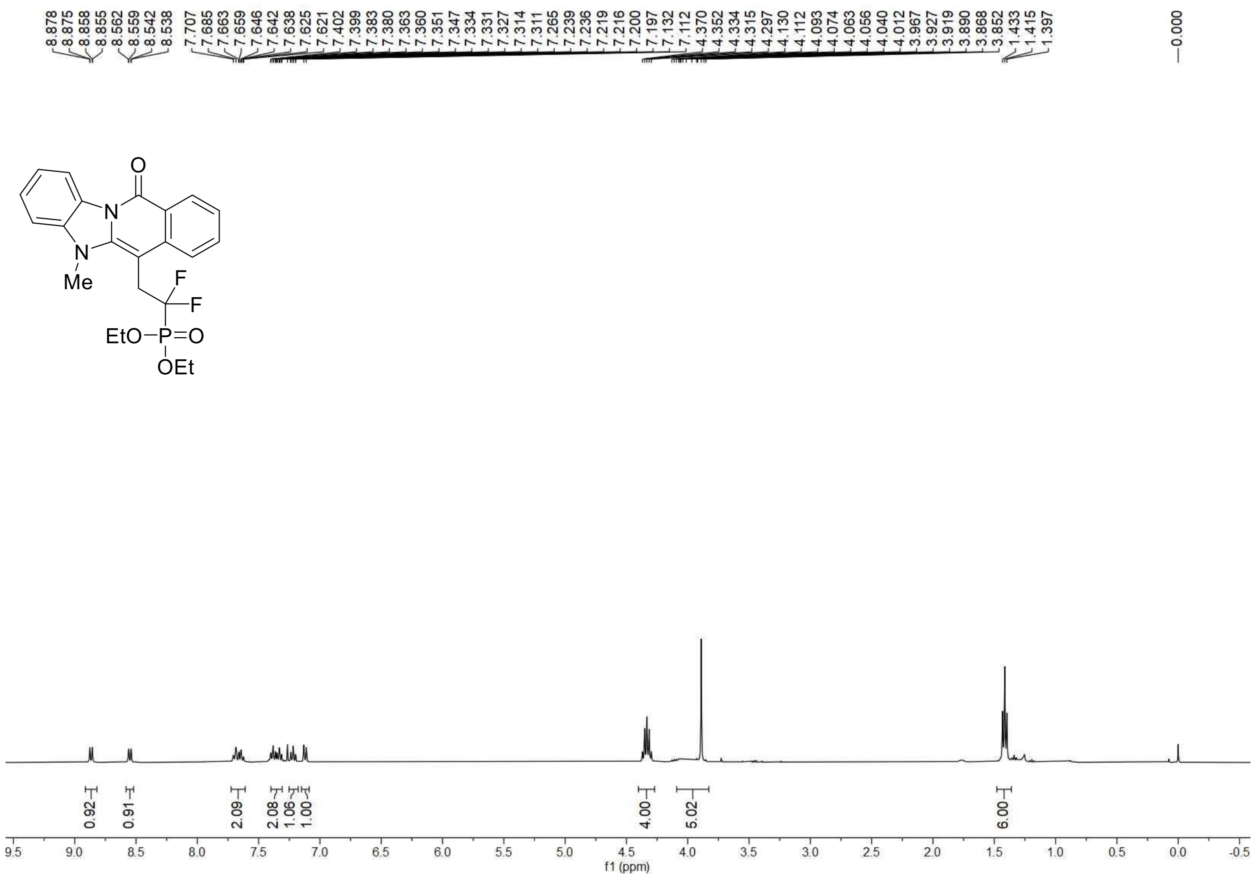

Supplementary Figure 136. ^{1}H-NMR of compound **16**, recorded at 400 MHz and 25 °C in $CDCl_3$.

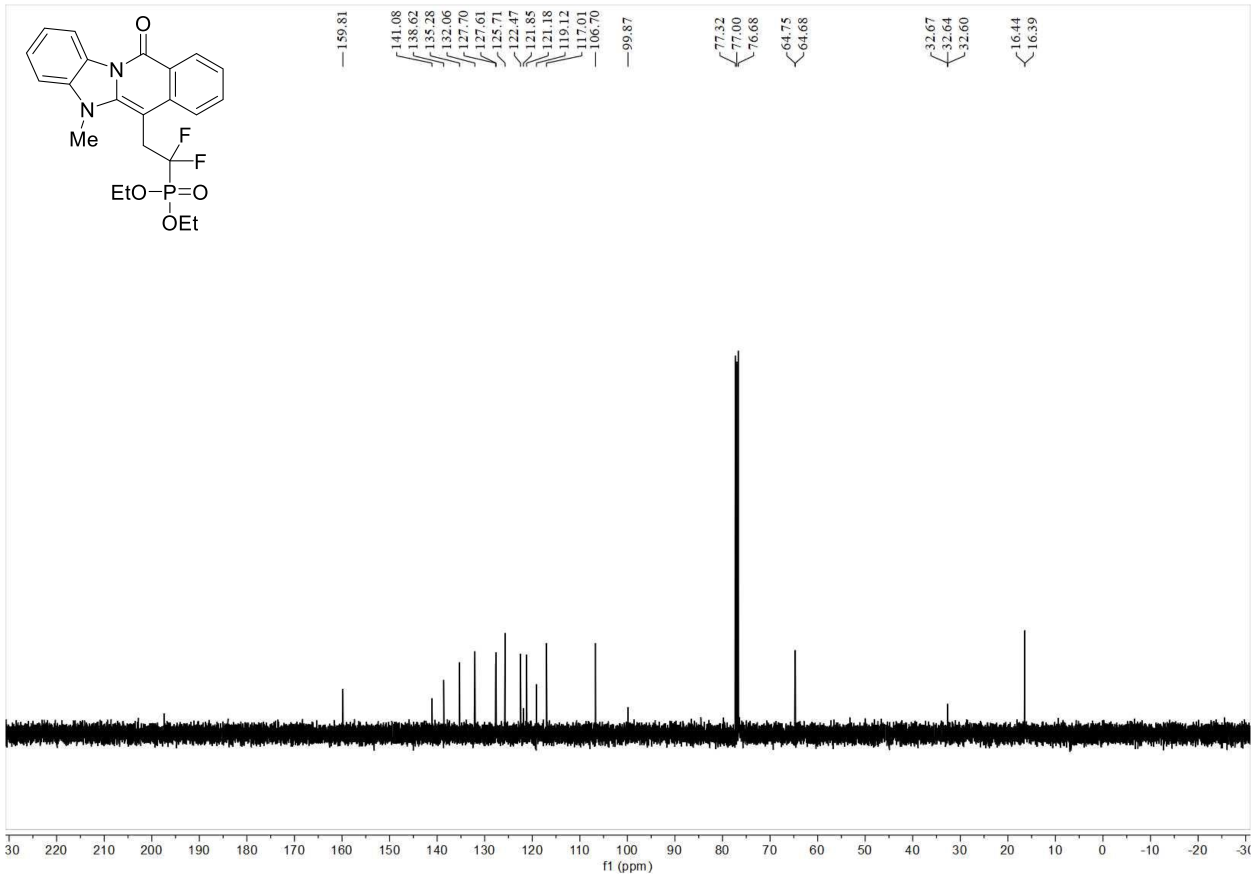

Supplementary Figure 137. ^{13}C-NMR of compound **16**, recorded at 100 MHz and 25 °C in $CDCl_3$.

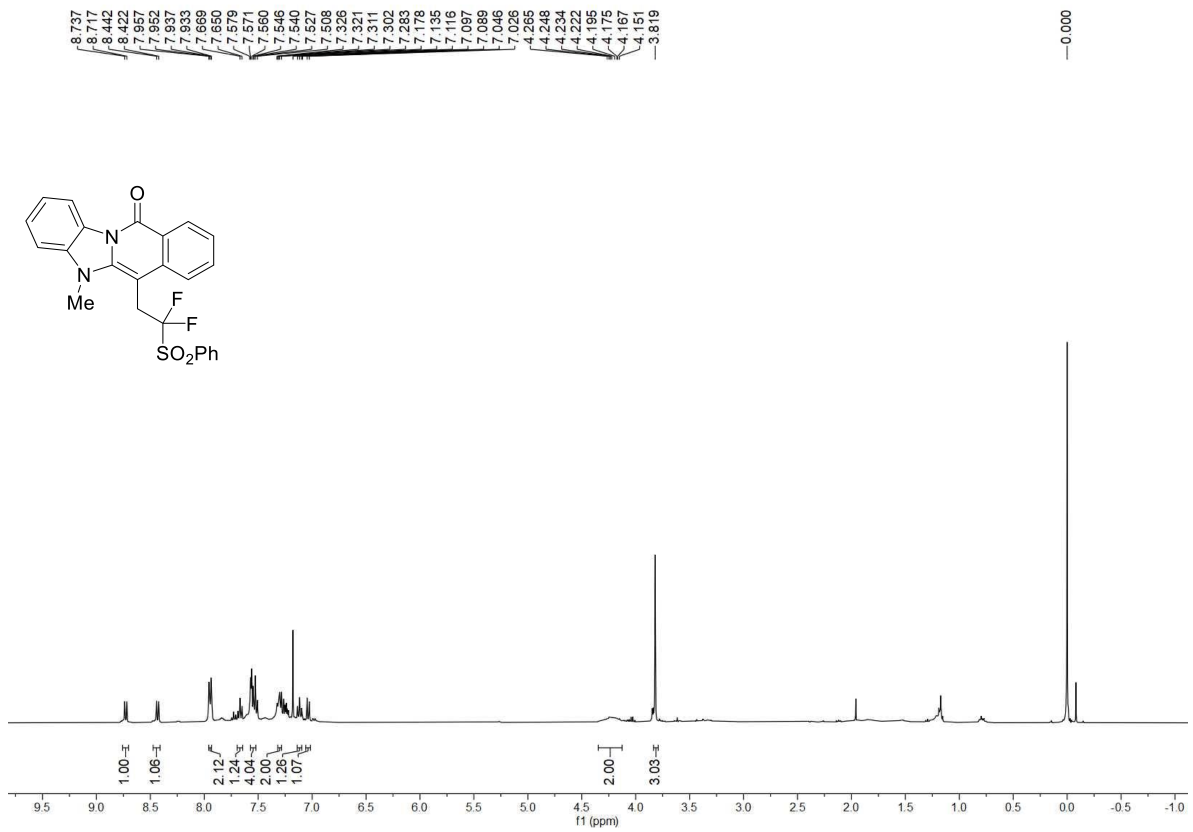

Supplementary Figure 138. ^{1}H-NMR of compound **17**, recorded at 400 MHz and 25 °C in $CDCl_3$.

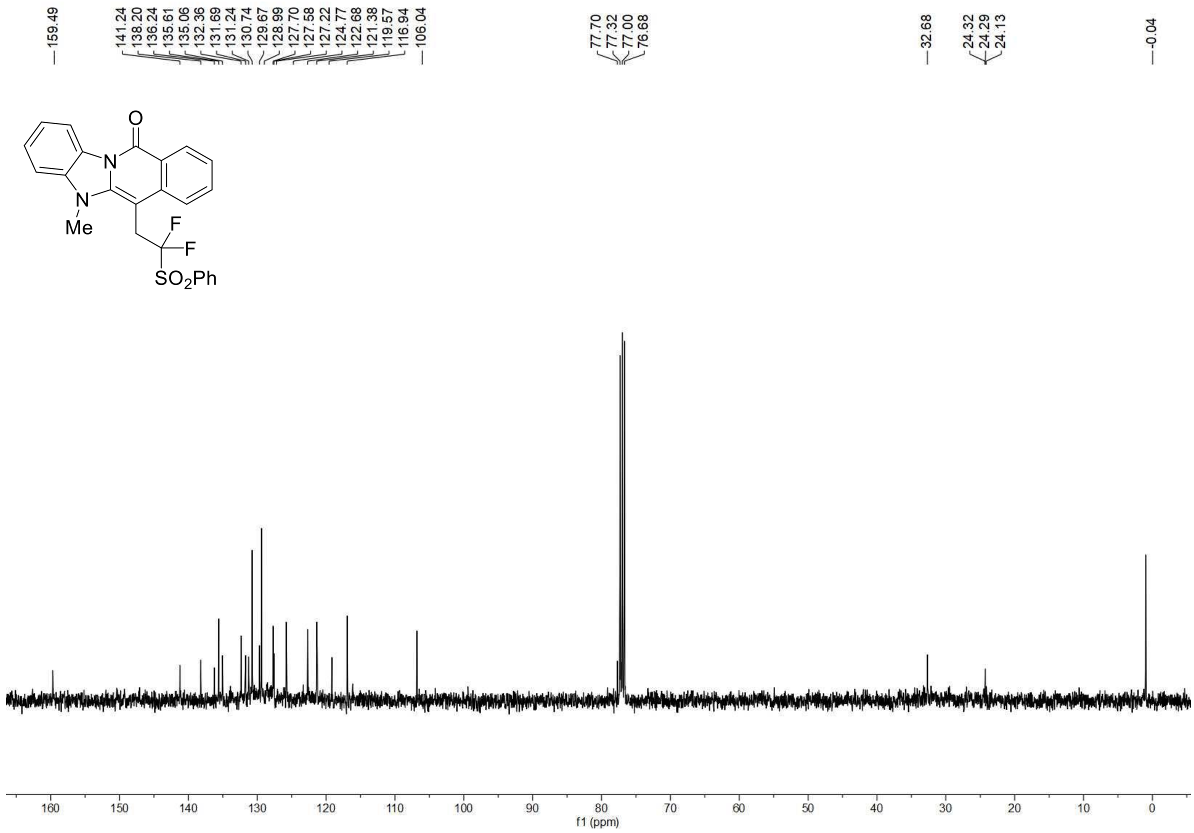

Supplementary Figure 139. ^{13}C-NMR of compound **17**, recorded at 100 MHz and 25 °C in $CDCl_3$.

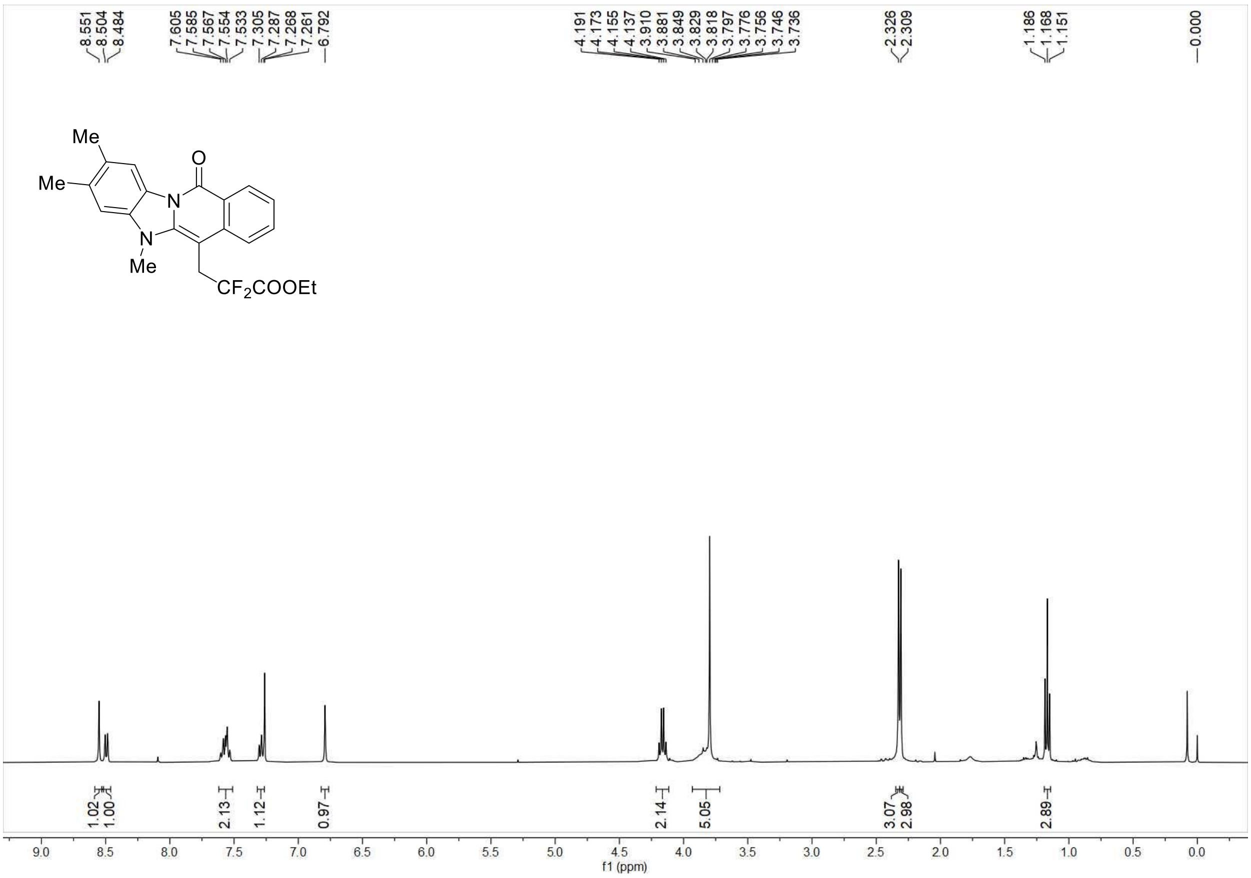

Supplementary Figure 140. ^{1}H-NMR of compound **18**, recorded at 400 MHz and 25 °C in $CDCl_3$.

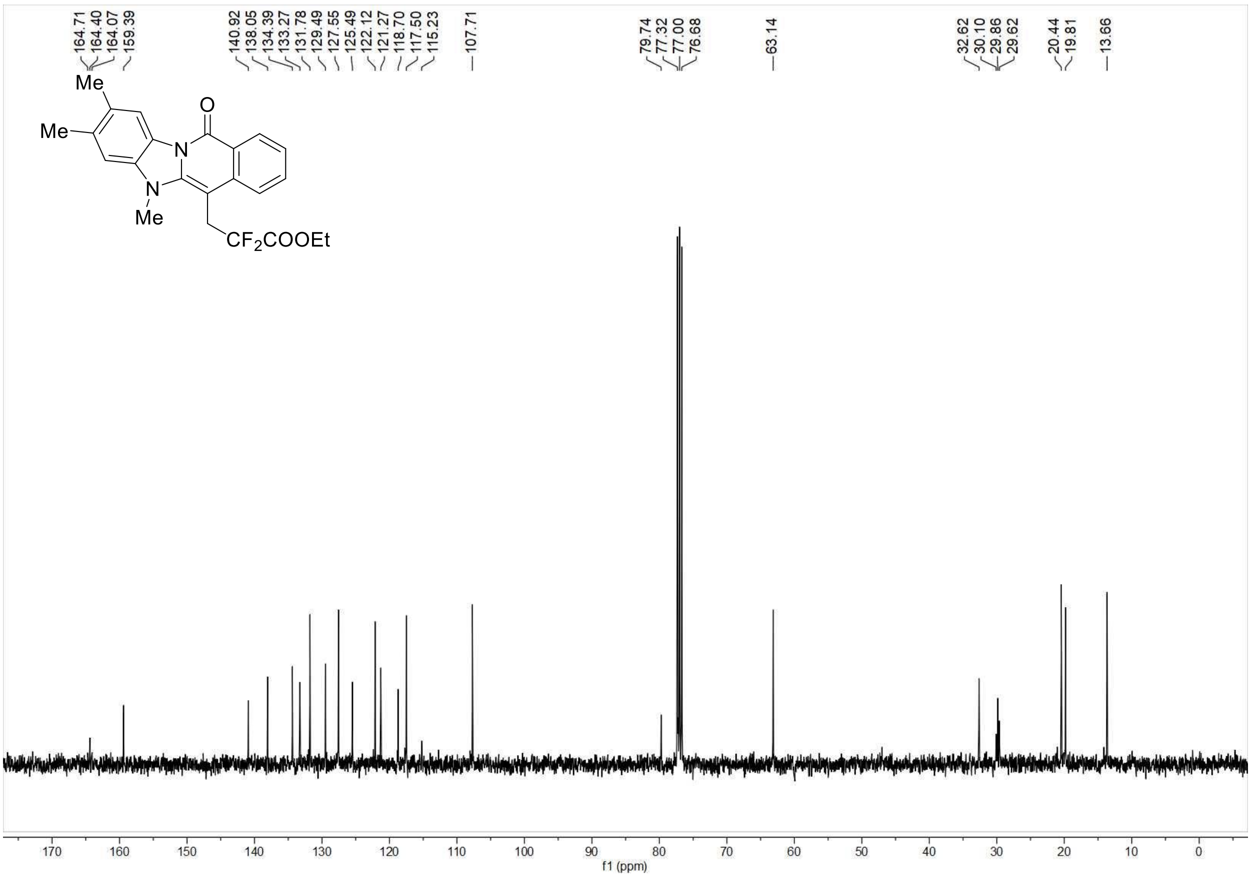

Supplementary Figure 141. ^{13}C-NMR of compound **18**, recorded at 100 MHz and 25 °C in $CDCl_3$.

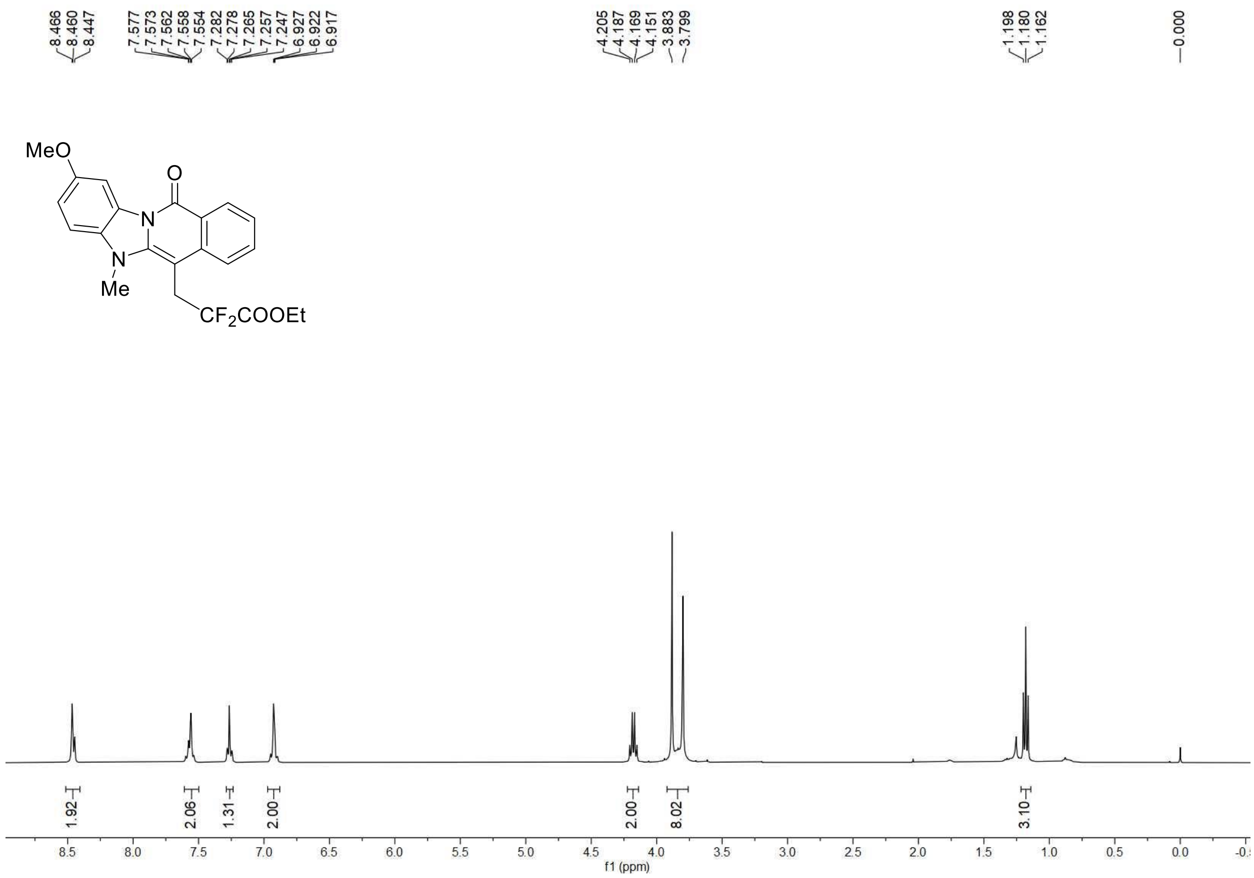

Supplementary Figure 142. ^{1}H-NMR of compound **19**, recorded at 400 MHz and 25 °C in $CDCl_3$.

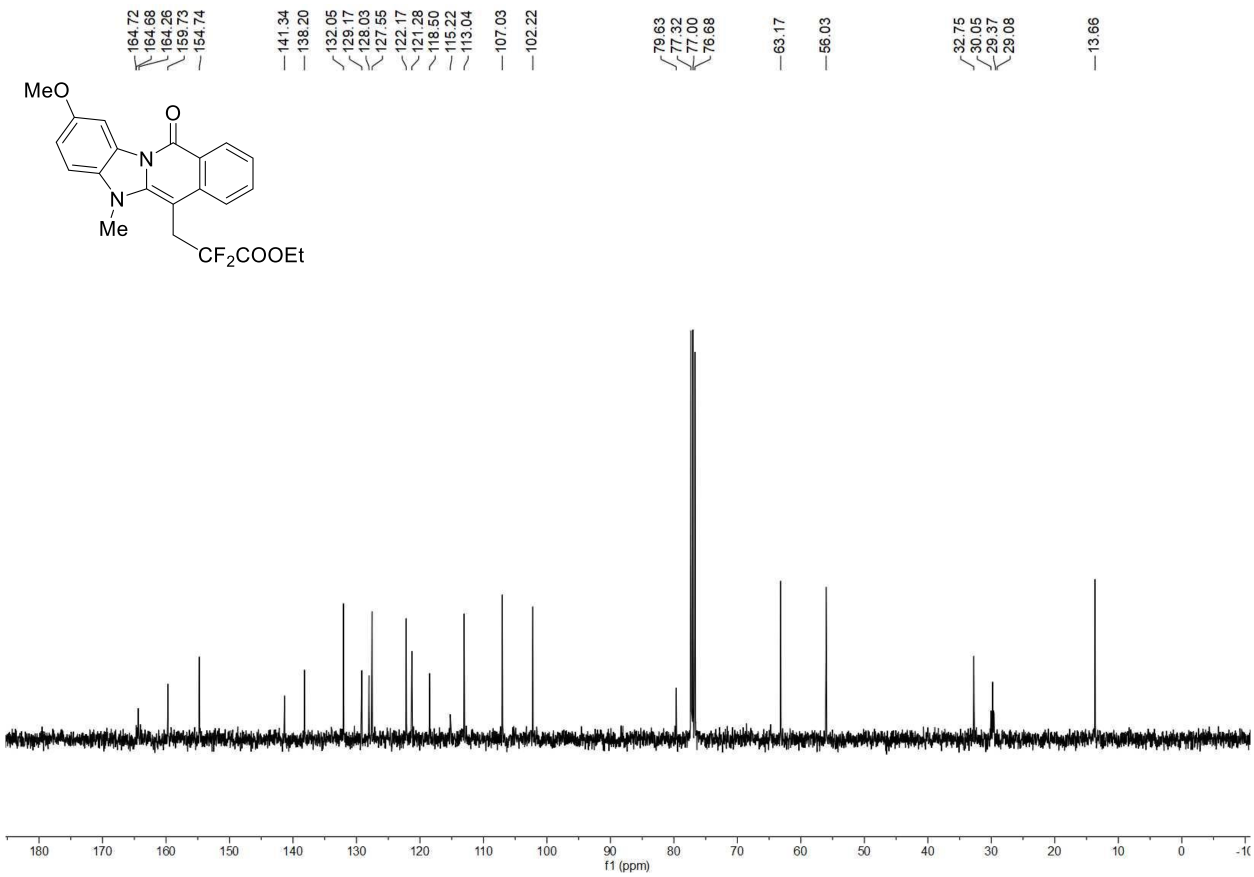

Supplementary Figure 143. ^{13}C-NMR of compound **19**, recorded at 100 MHz and 25 °C in $CDCl_3$.

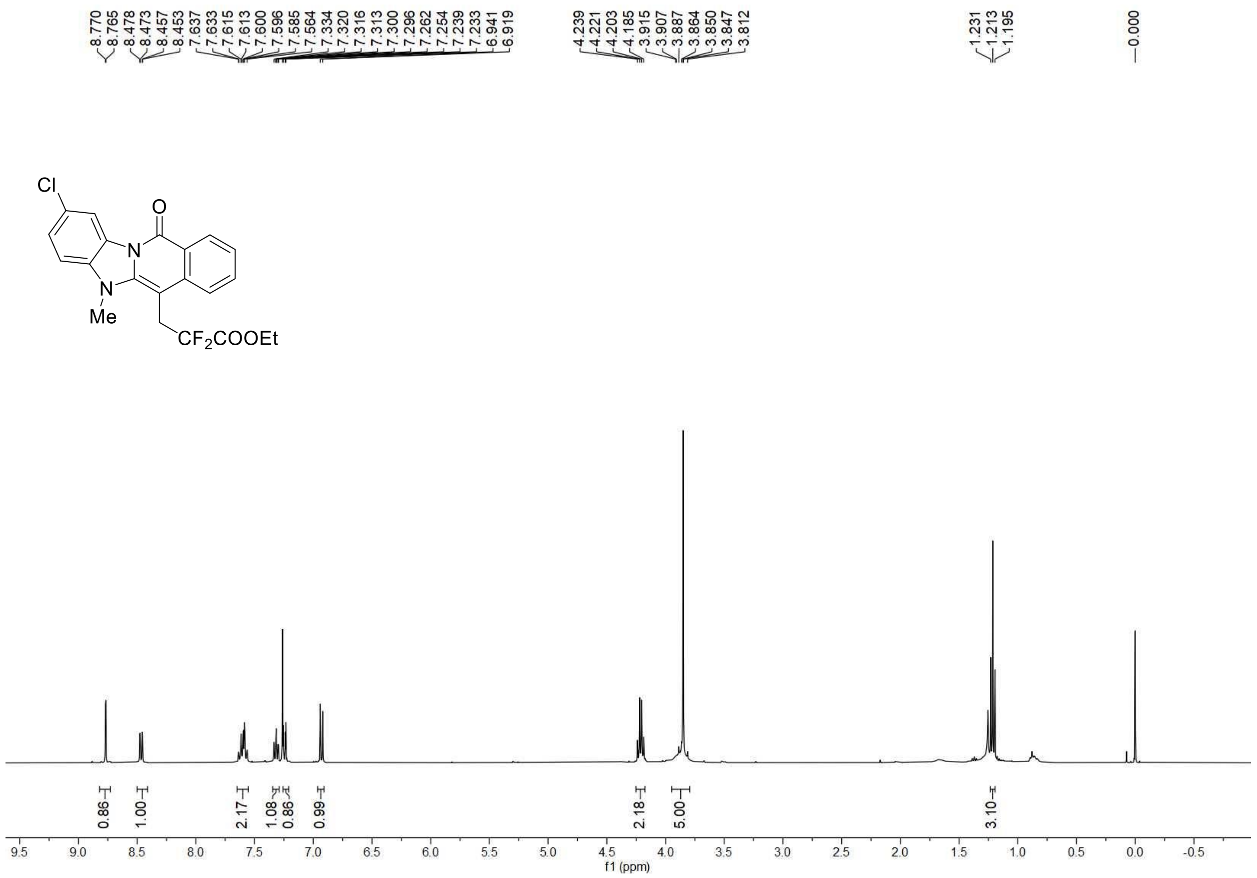

Supplementary Figure 144. ^{1}H-NMR of compound **20**, recorded at 400 MHz and 25 °C in $CDCl_3$.

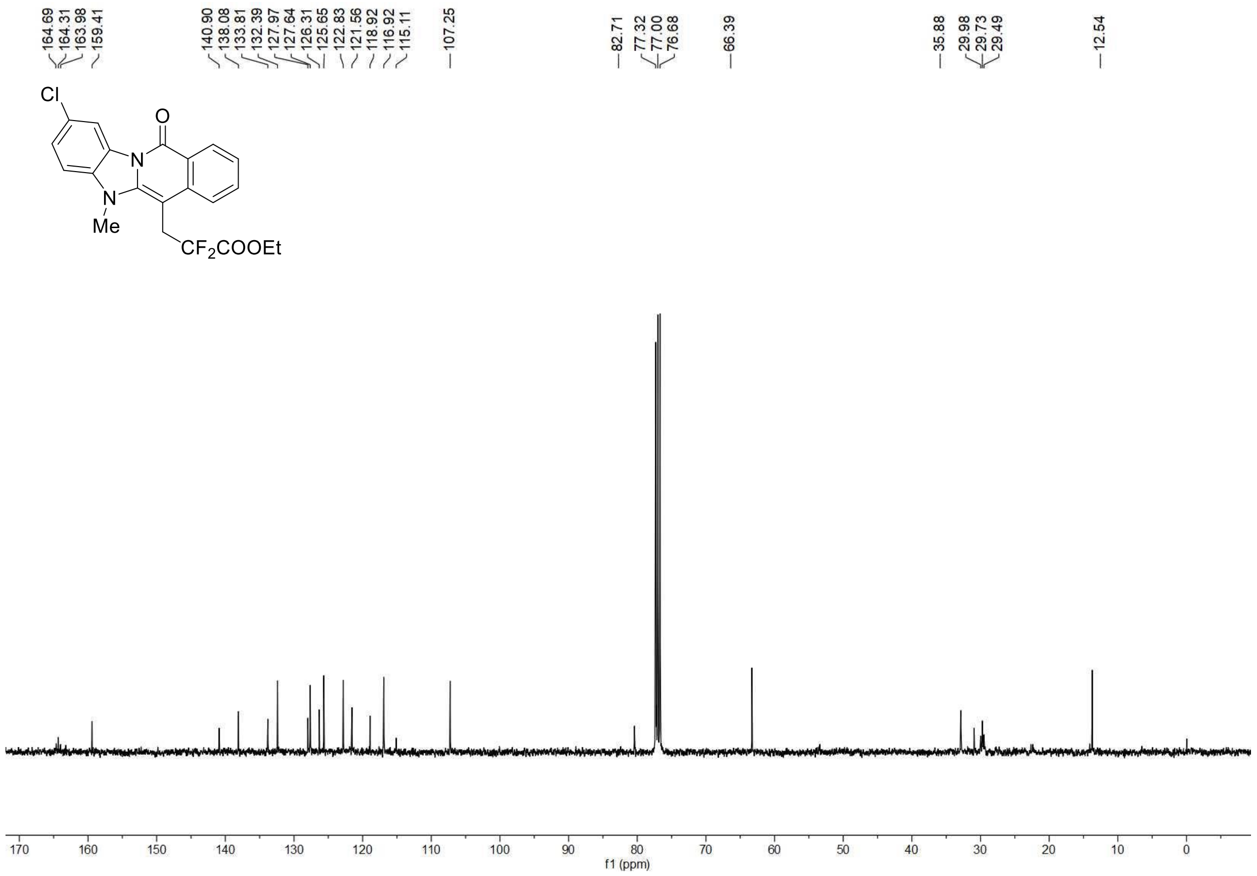

Supplementary Figure 145. ^{13}C-NMR of compound **20**, recorded at 100 MHz and 25 °C in $CDCl_3$.\

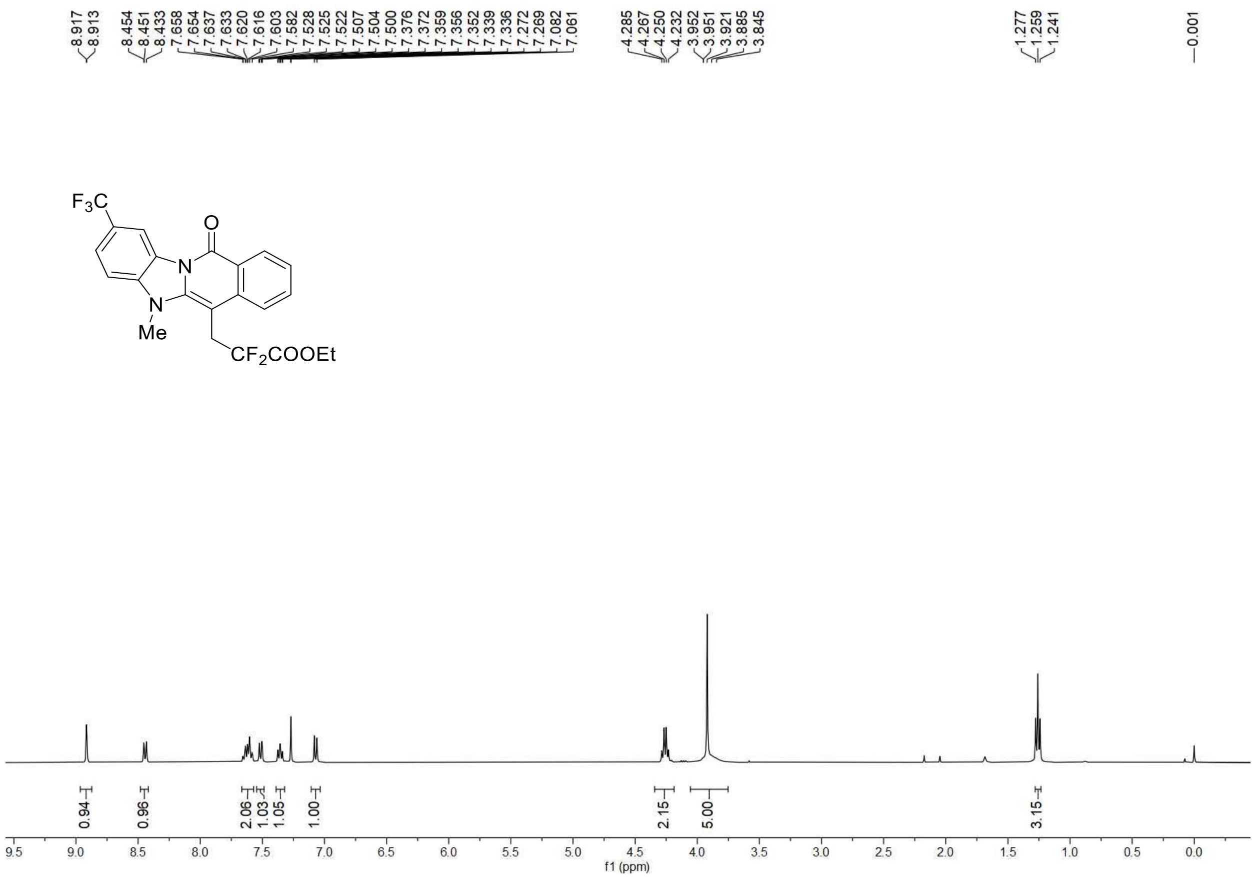

Supplementary Figure 146. ^{1}H-NMR of compound **21**, recorded at 400 MHz and 25 °C in $CDCl_3$.

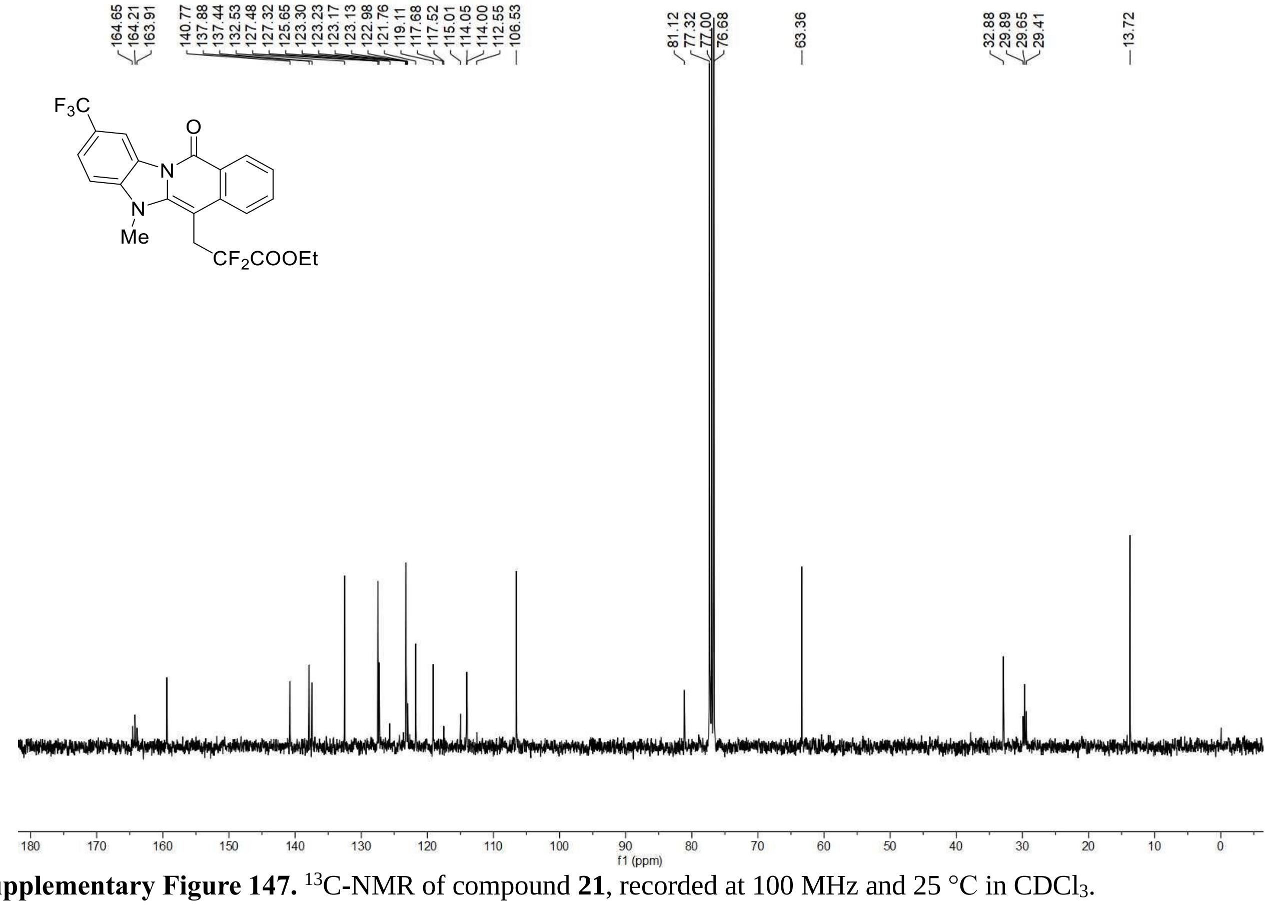

Supplementary Figure 147. ^{13}C-NMR of compound **21**, recorded at 100 MHz and 25 °C in $CDCl_3$.

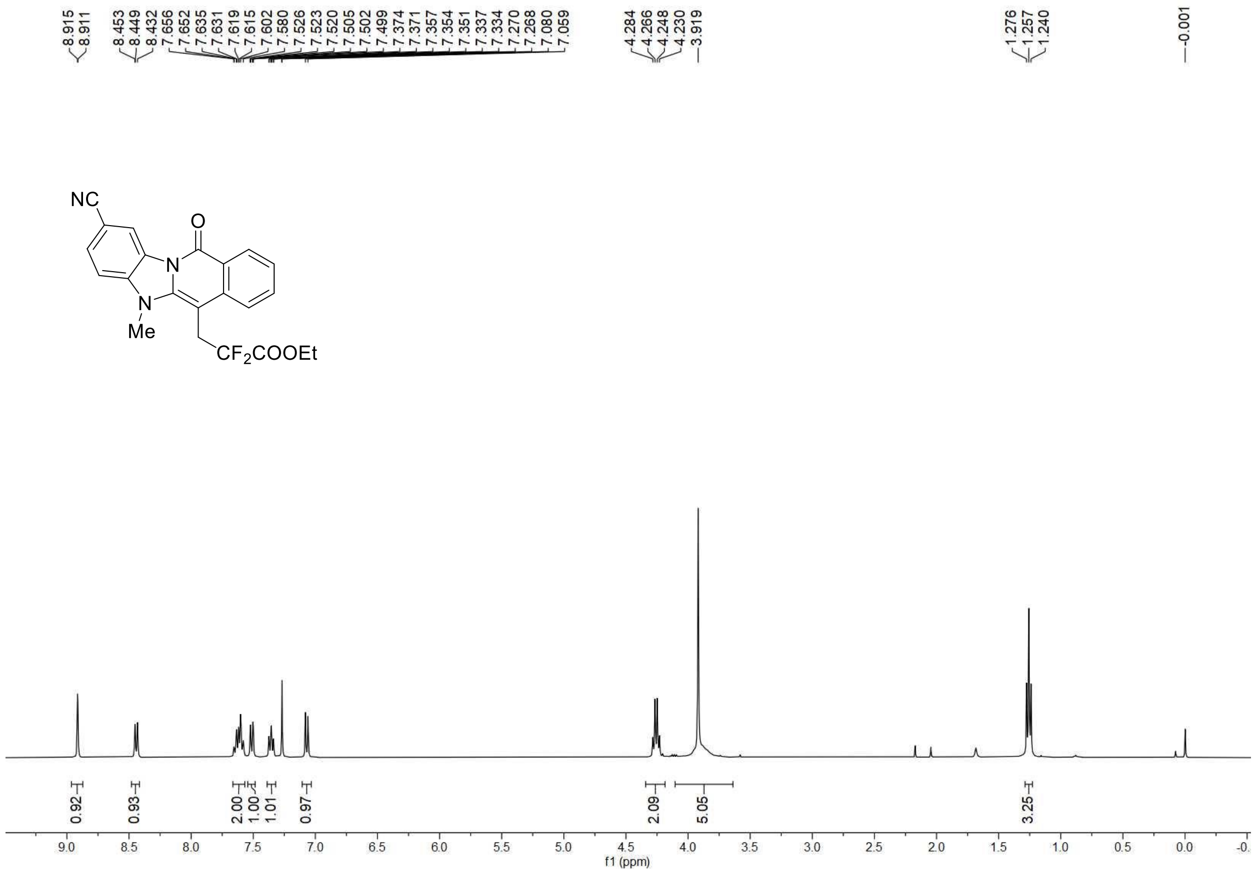

Supplementary Figure 148. ^{1}H-NMR of compound **22**, recorded at 400 MHz and 25 °C in $CDCl_3$.

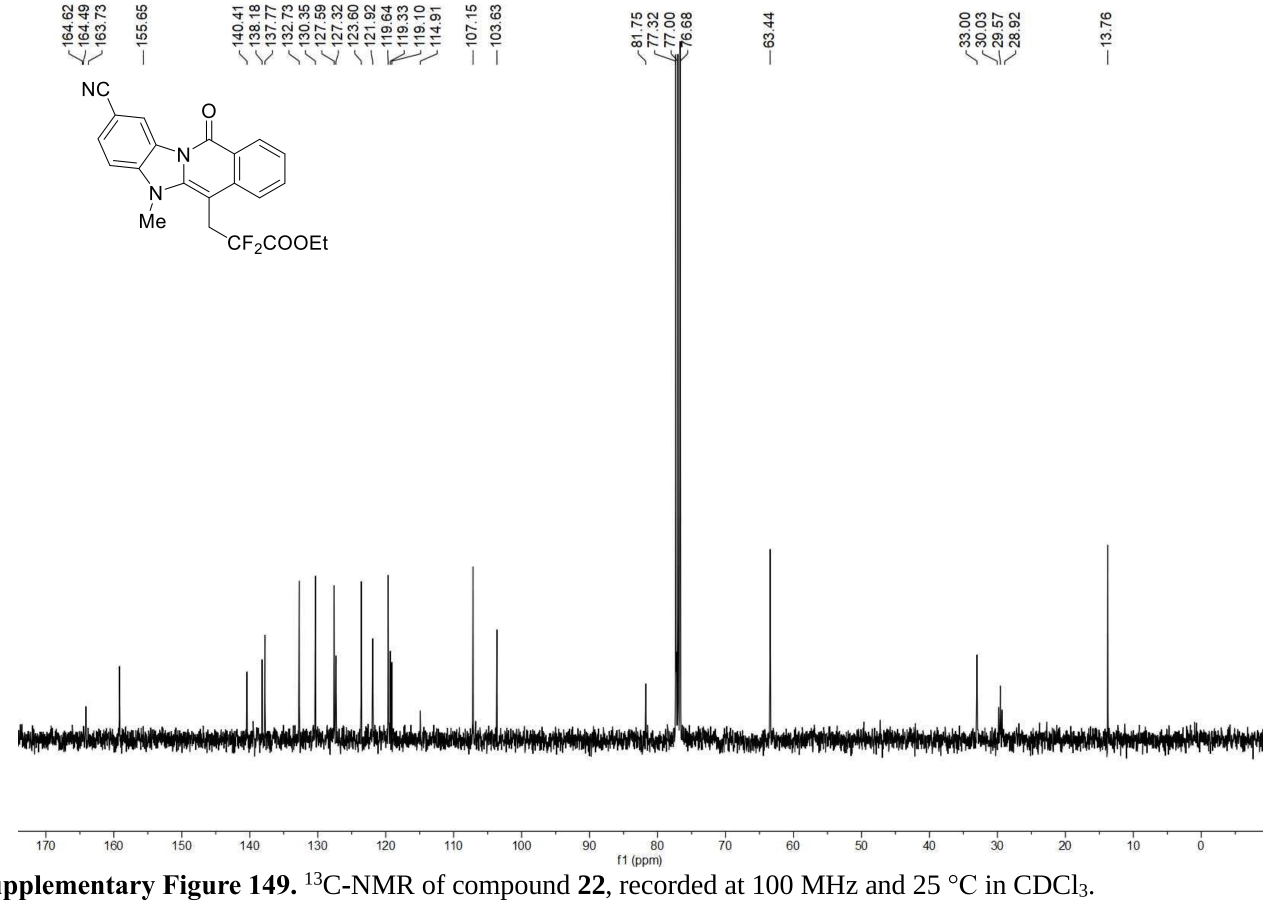

Supplementary Figure 149. ^{13}C-NMR of compound **22**, recorded at 100 MHz and 25 °C in $CDCl_3$.

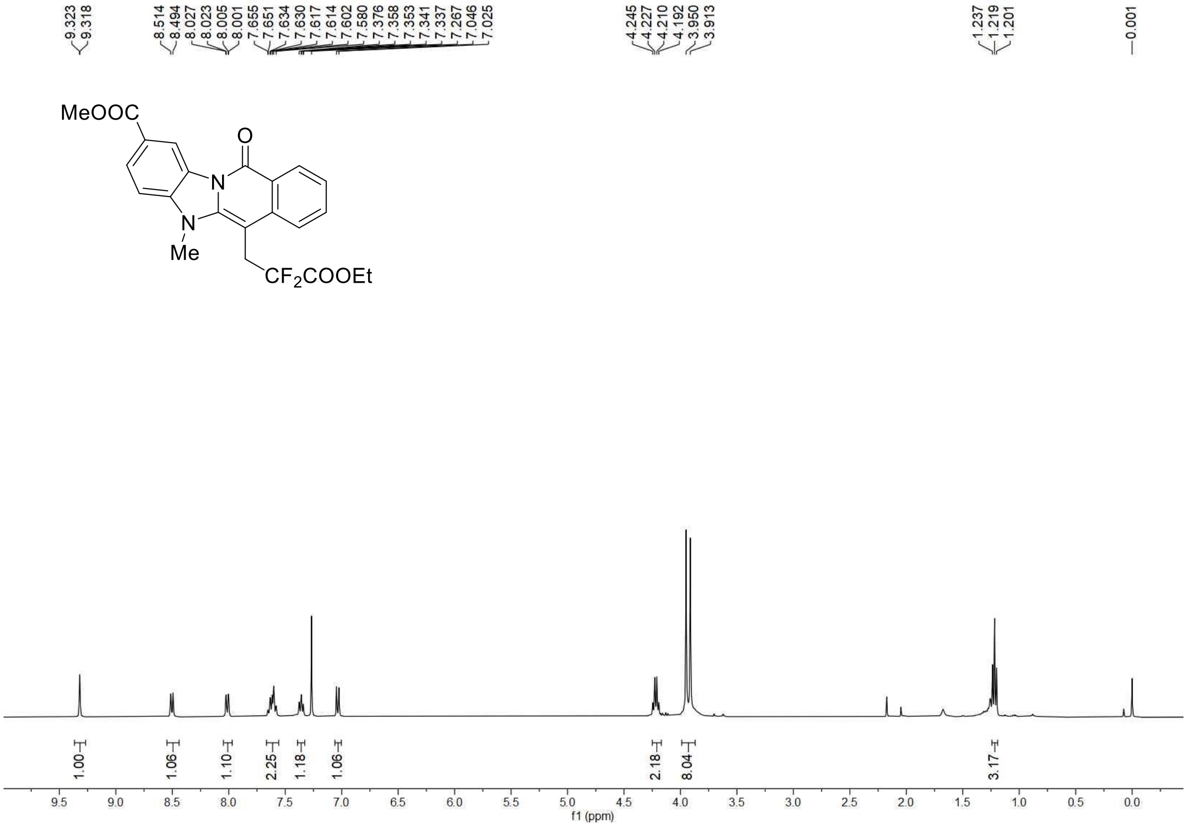

Supplementary Figure 150. ^{1}H-NMR of compound **23**, recorded at 400 MHz and 25 °C in $CDCl_3$.

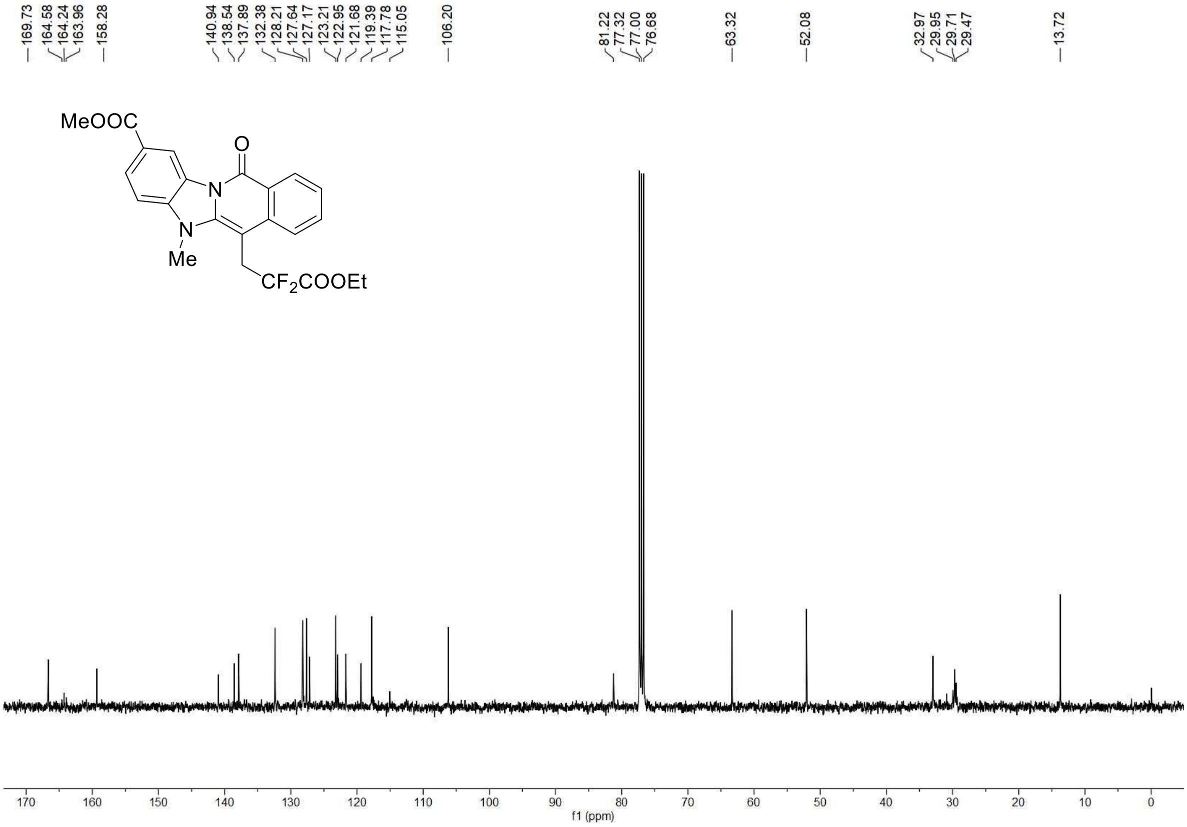

Supplementary Figure 151. ^{13}C-NMR of compound **23**, recorded at 100 MHz and 25 °C in $CDCl_3$.

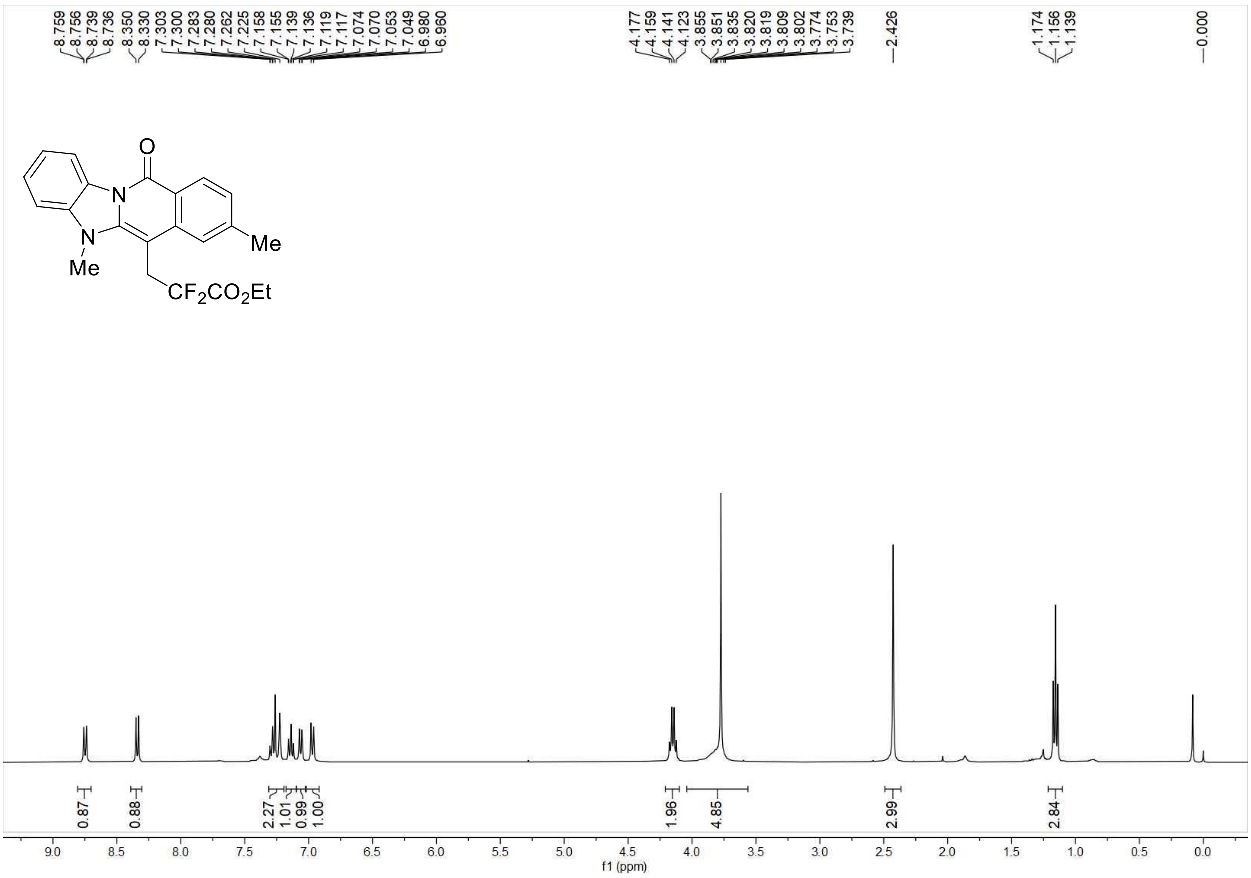

Supplementary Figure 152. ^{1}H-NMR of compound **24**, recorded at 400 MHz and 25 °C in $CDCl_3$.

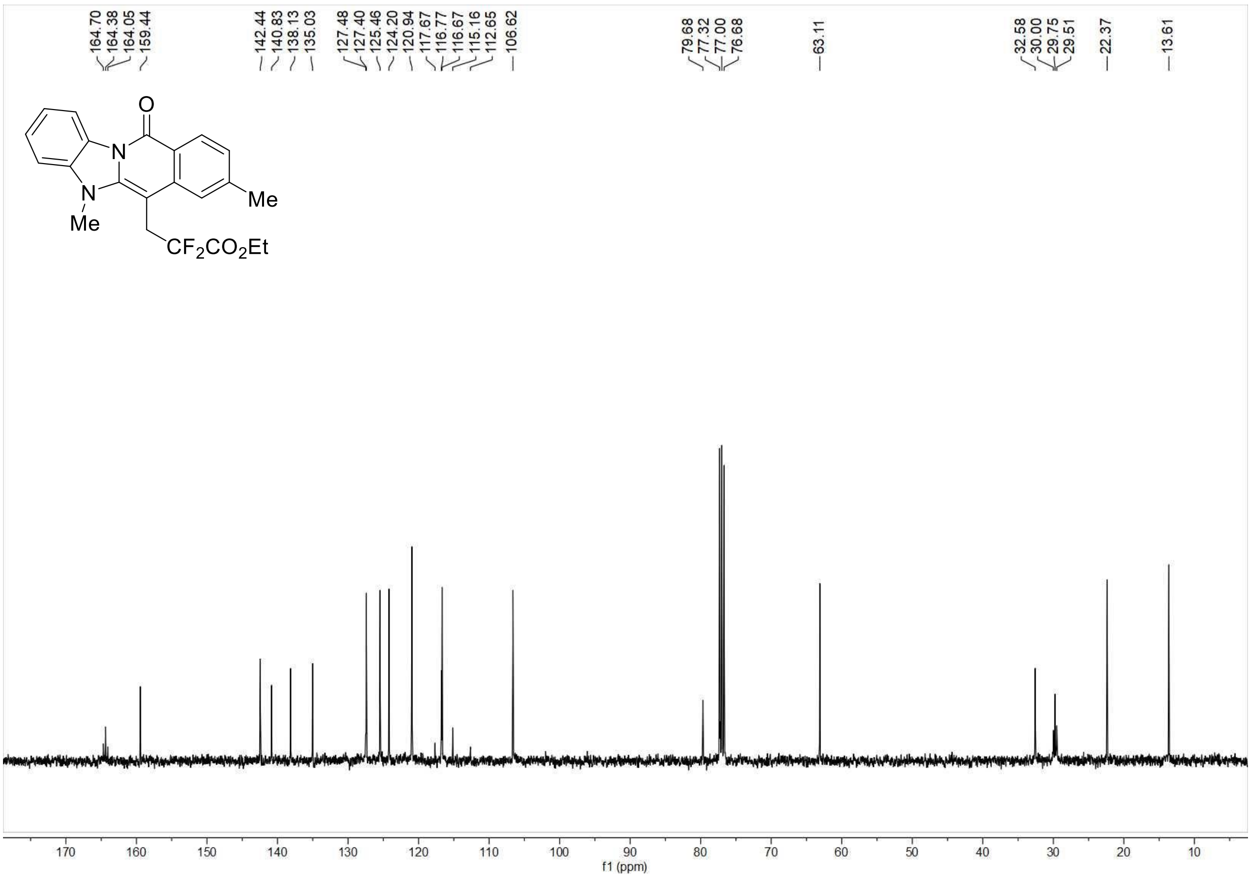

Supplementary Figure 153. ^{13}C-NMR of compound **24**, recorded at 100 MHz and 25 °C in $CDCl_3$.

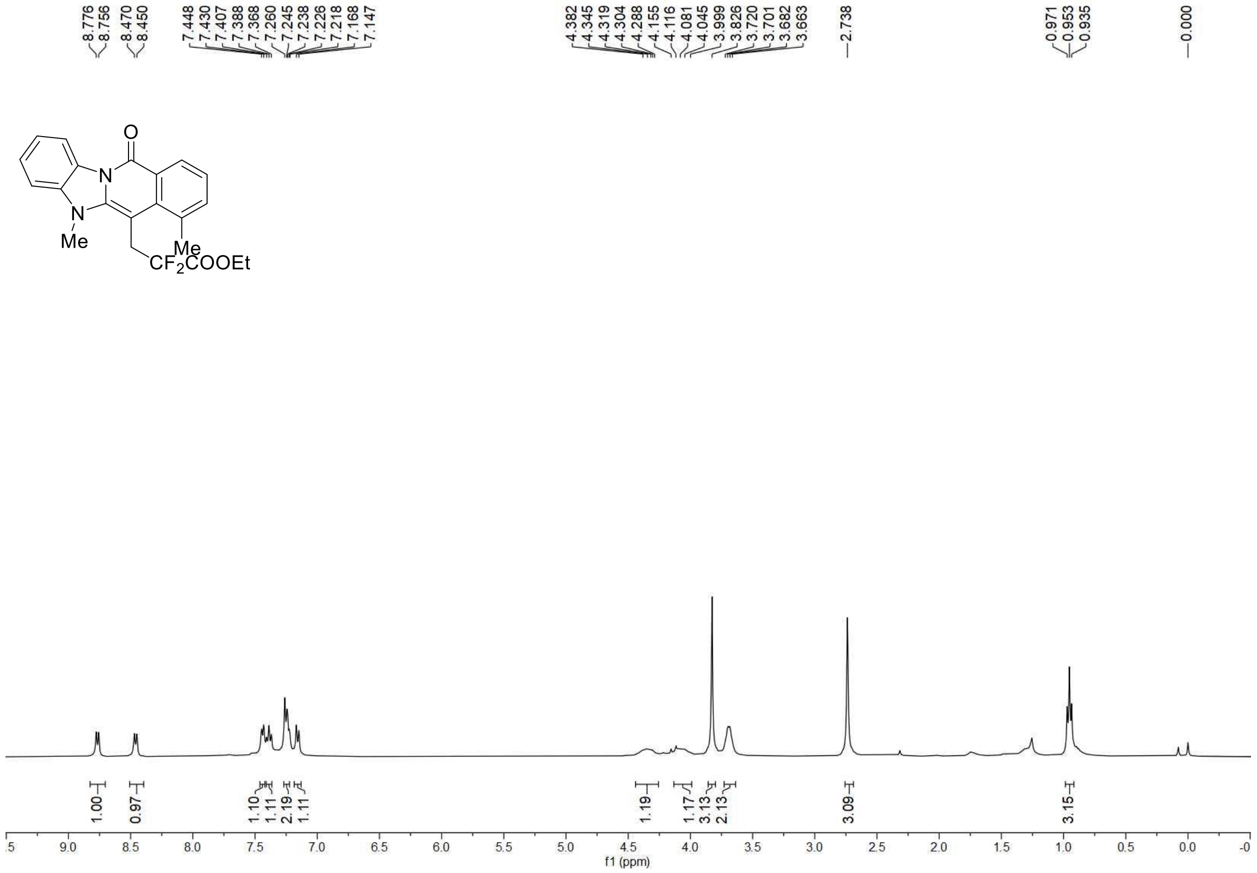

Supplementary Figure 154. ^{1}H-NMR of compound **25**, recorded at 400 MHz and 25 °C in $CDCl_3$.

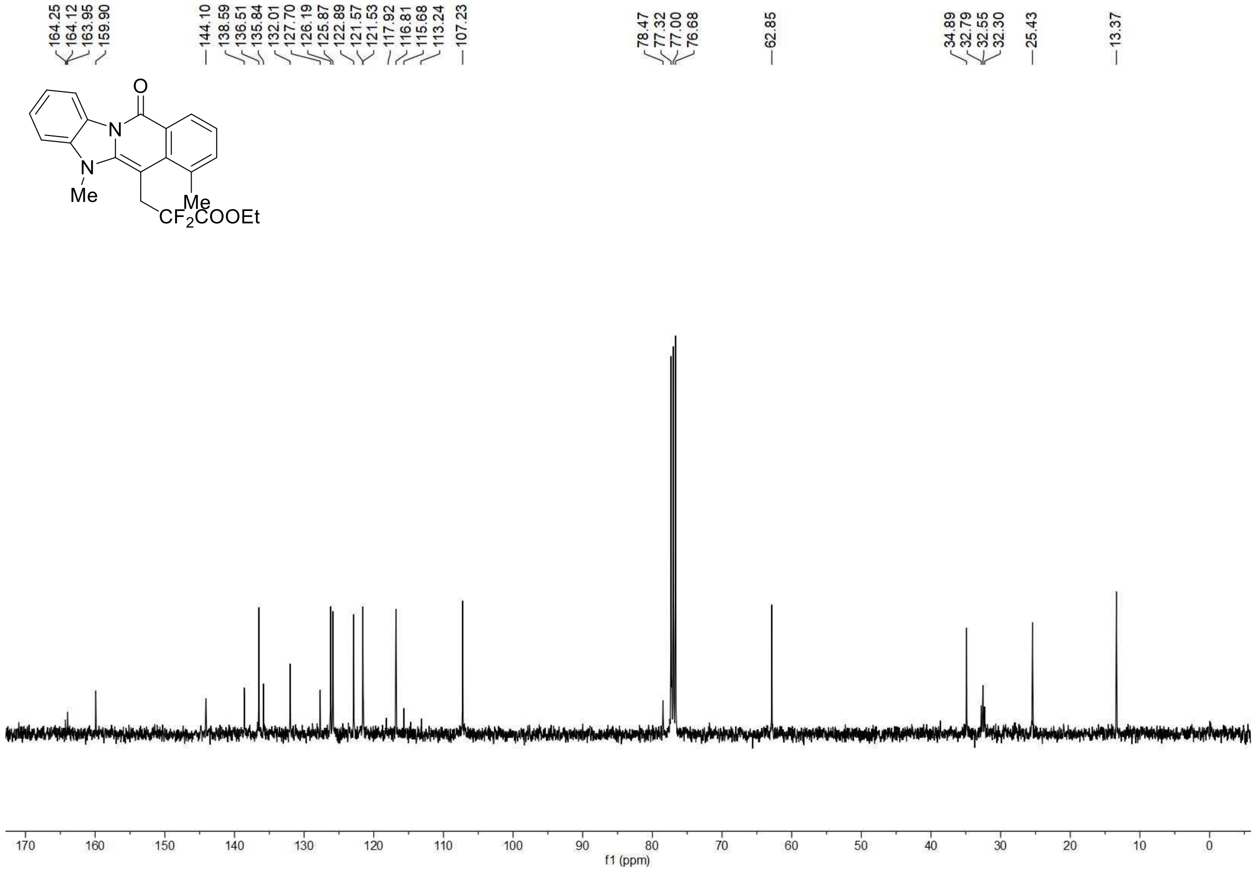

Supplementary Figure 155. ^{13}C-NMR of compound **25**, recorded at 100 MHz and 25 °C in $CDCl_3$.

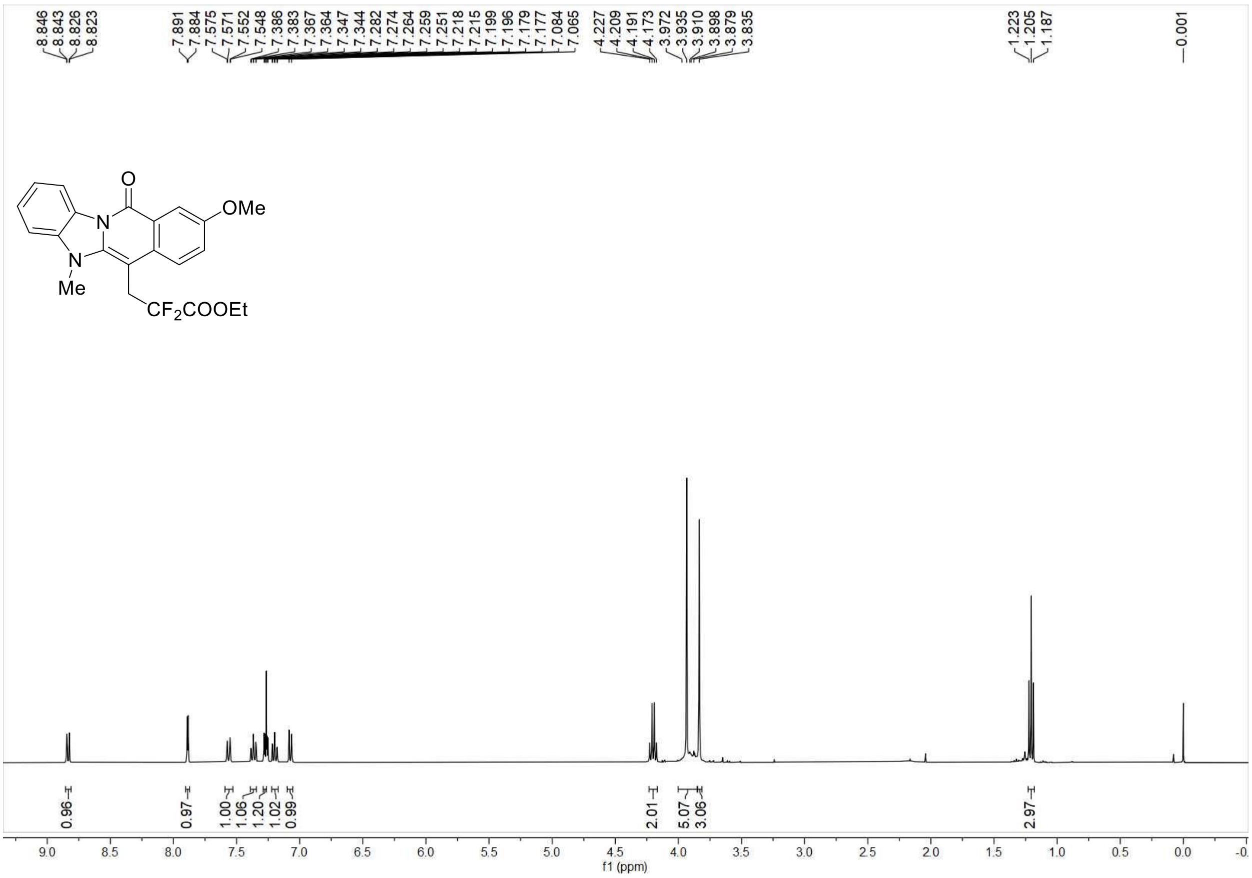

Supplementary Figure 156. ^{1}H-NMR of compound **26**, recorded at 400 MHz and 25 °C in $CDCl_3$.

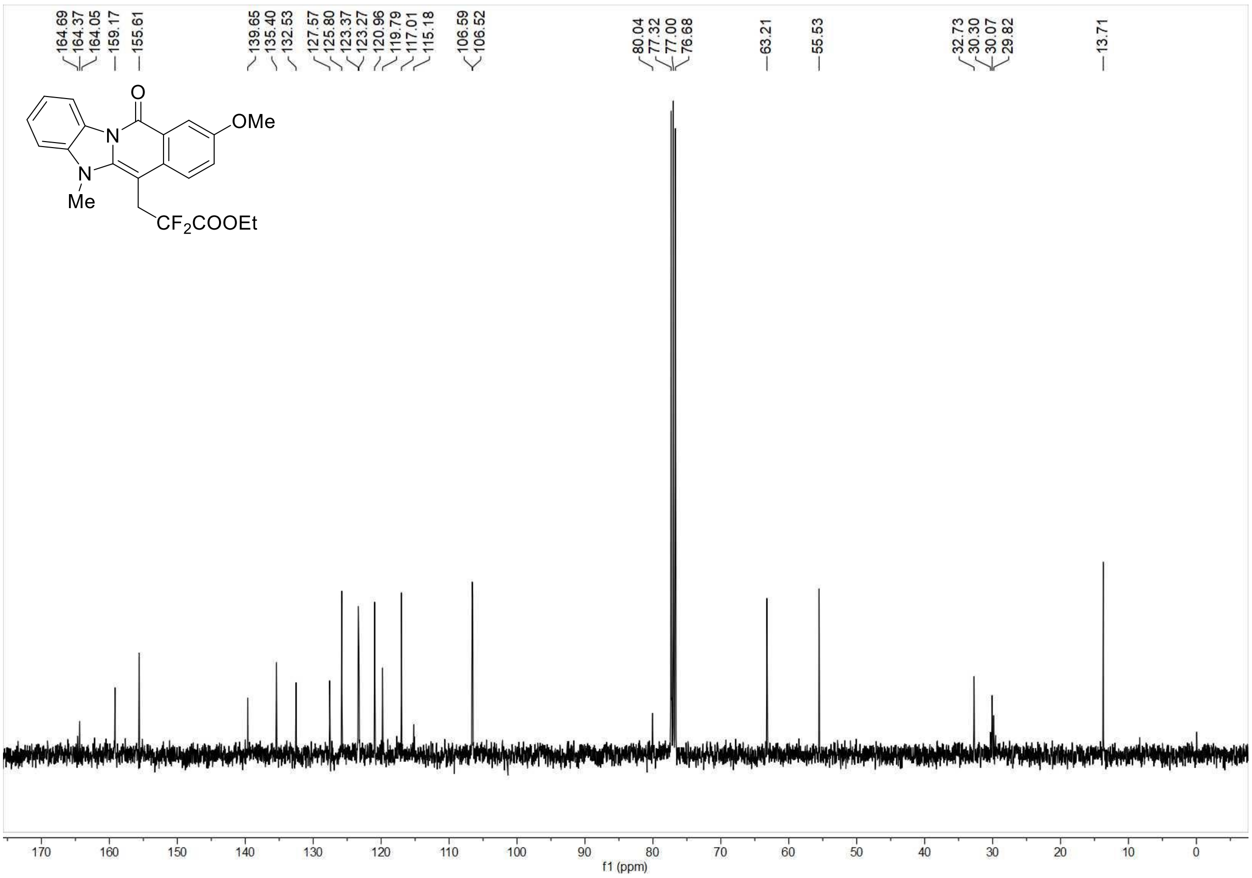

Supplementary Figure 157. ^{13}C-NMR of compound **26**, recorded at 100 MHz and 25 °C in $CDCl_3$.

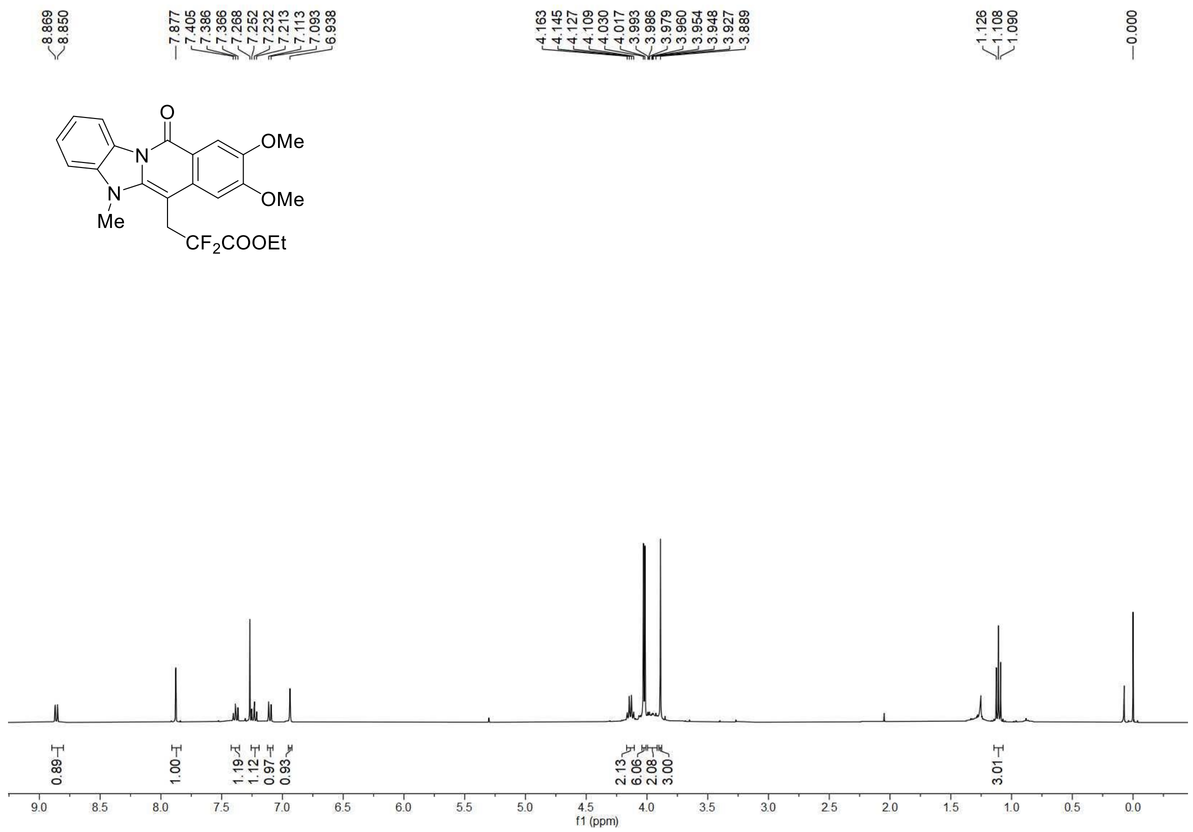

Supplementary Figure 158. ^{1}H-NMR of compound **27**, recorded at 400 MHz and 25 °C in $CDCl_3$.

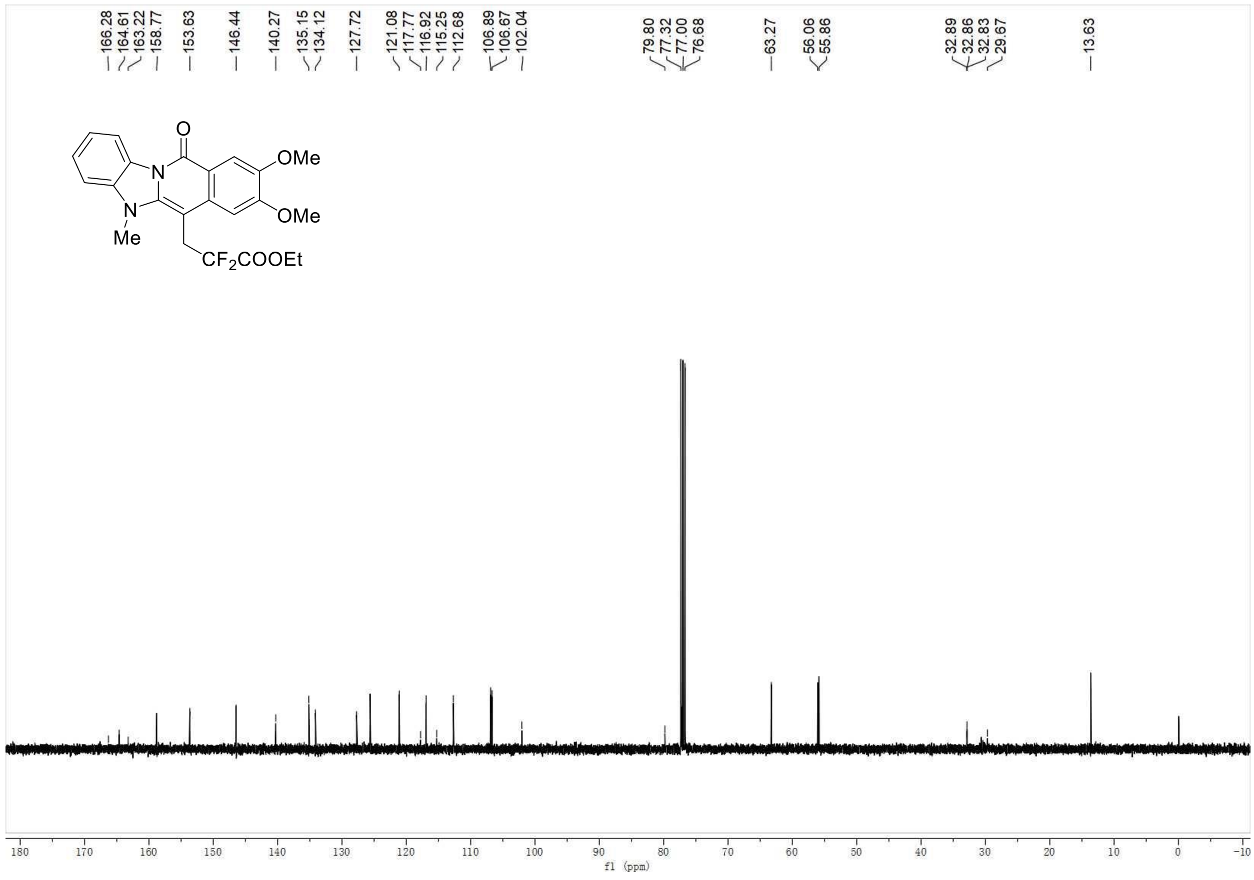

Supplementary Figure 159. ^{13}C-NMR of compound **27**, recorded at 100 MHz and 25 °C in $CDCl_3$.

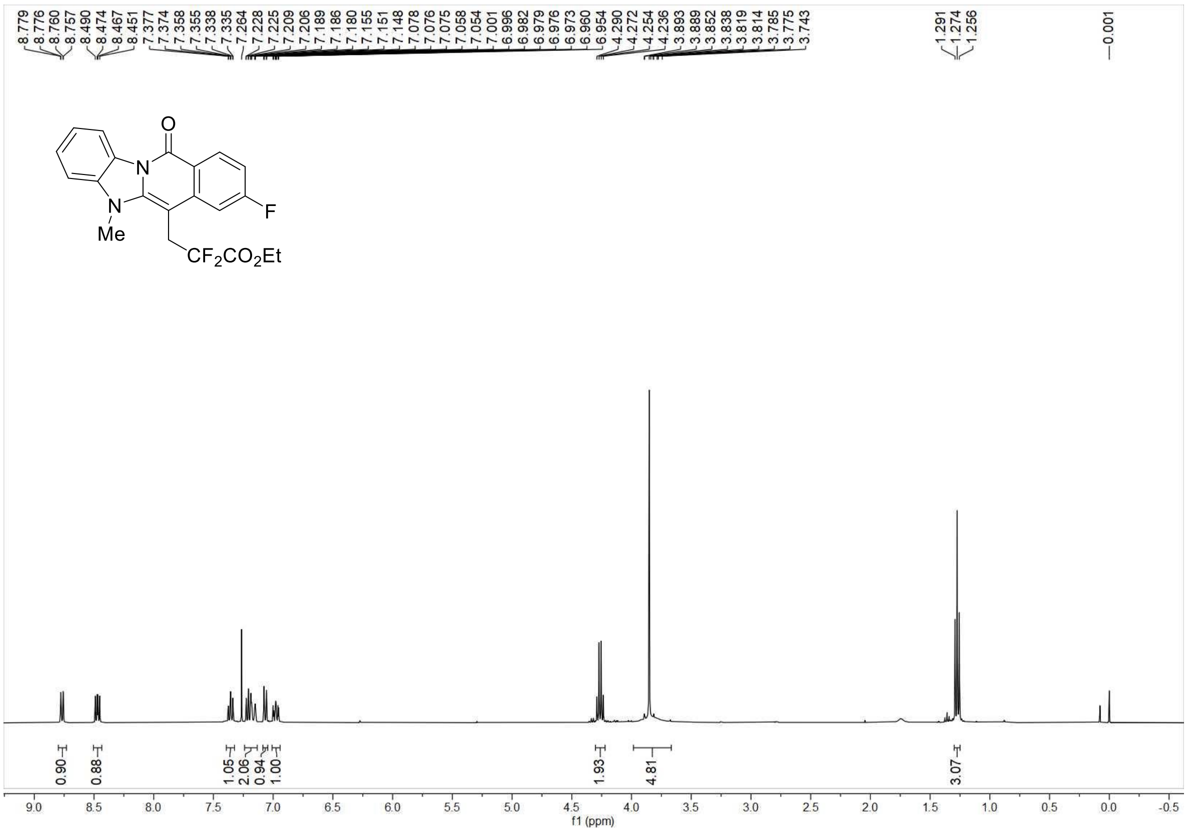

Supplementary Figure 160. ^{1}H-NMR of compound **28**, recorded at 400 MHz and 25 °C in $CDCl_3$.

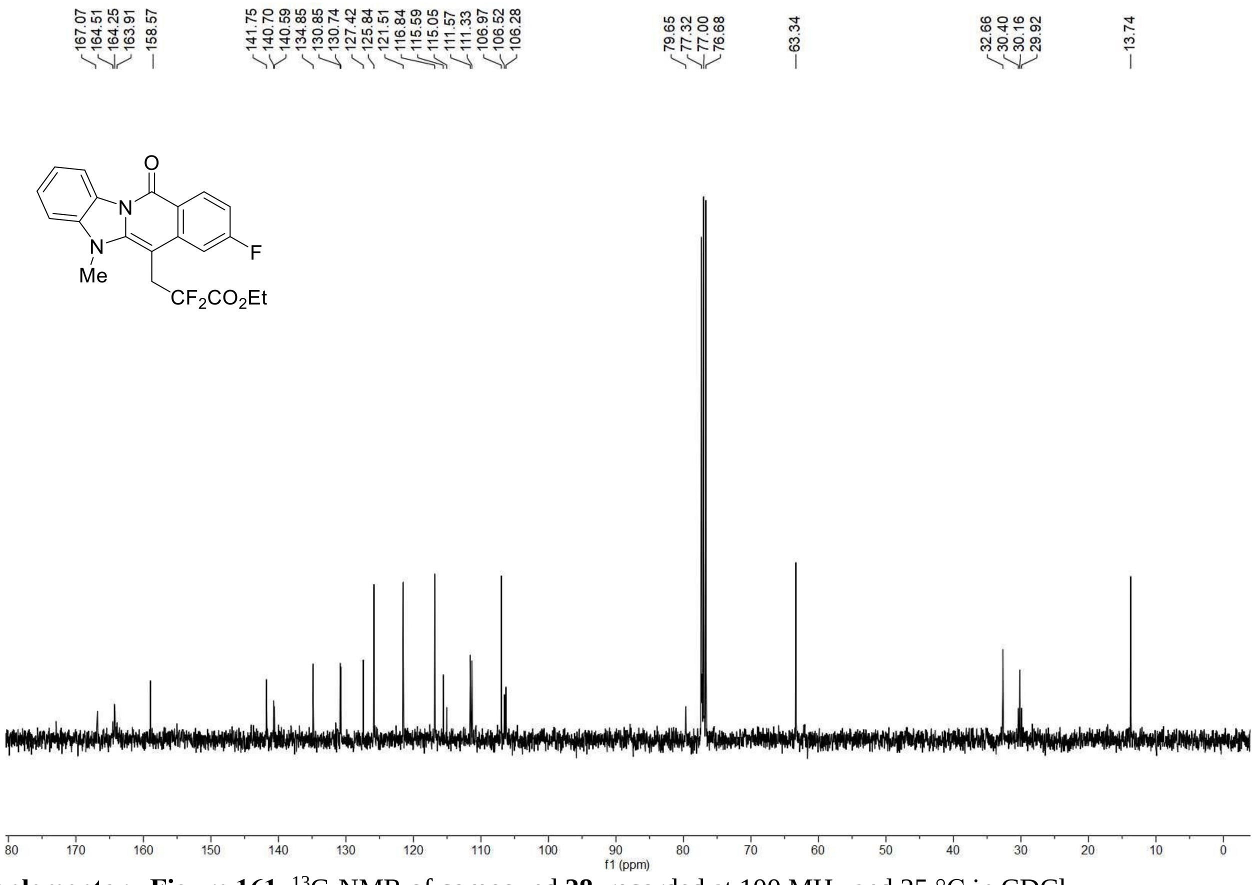

Supplementary Figure 161. ^{13}C-NMR of compound **28**, recorded at 100 MHz and 25 °C in $CDCl_3$.

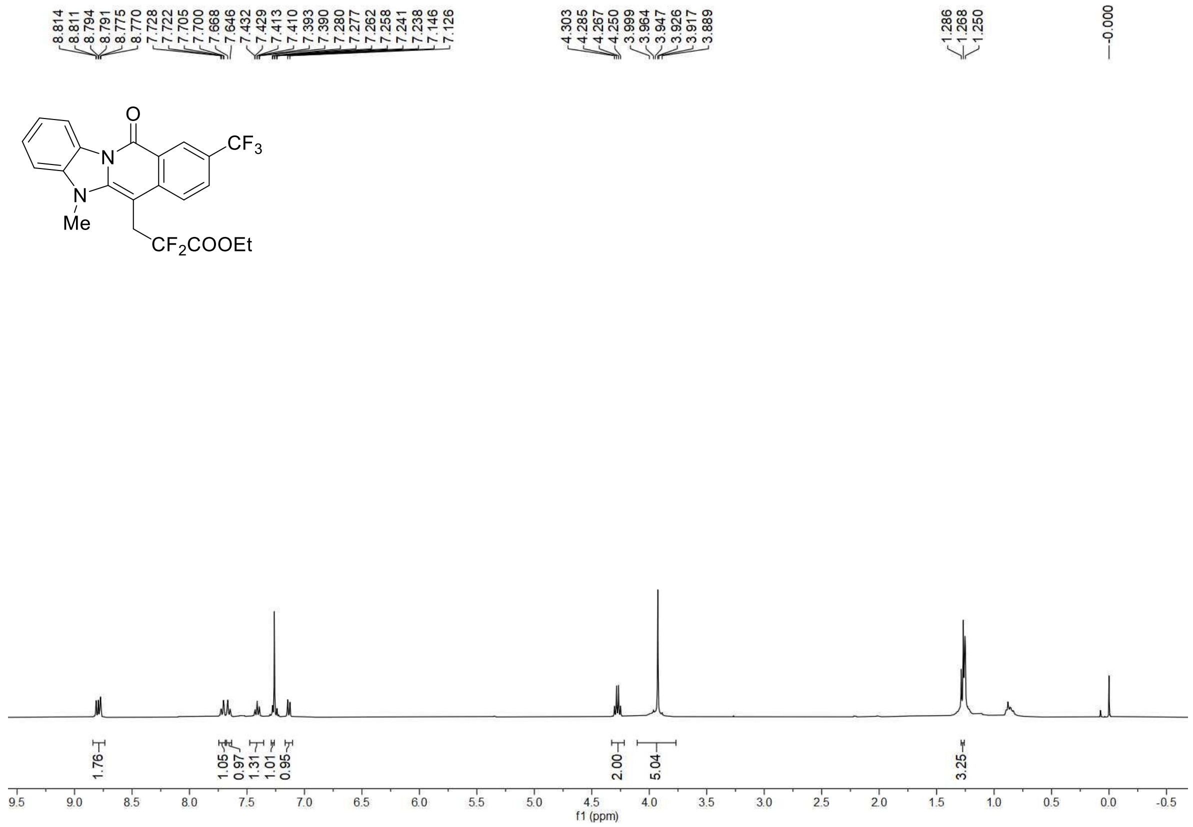

Supplementary Figure 162. ^{1}H-NMR of compound **29**, recorded at 400 MHz and 25 °C in $CDCl_3$.

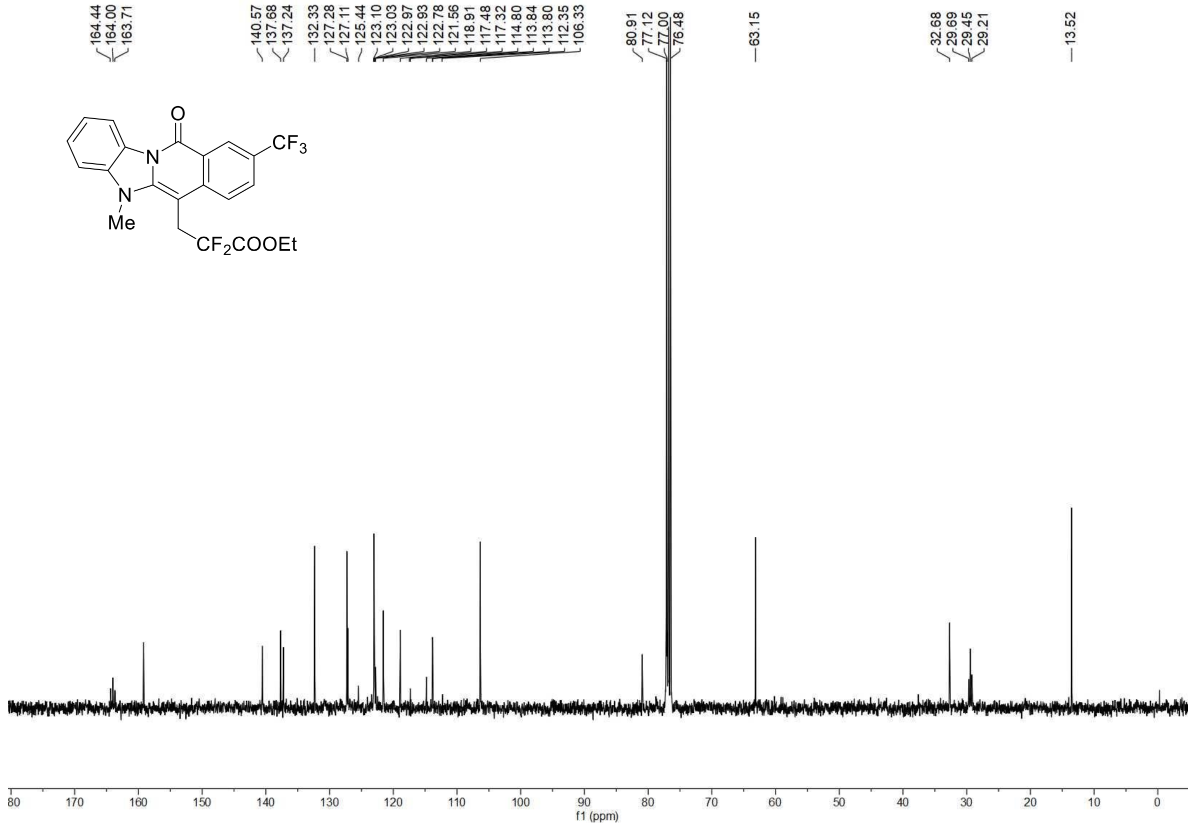

Supplementary Figure 163. ^{13}C-NMR of compound **29**, recorded at 100 MHz and 25 °C in $CDCl_3$.

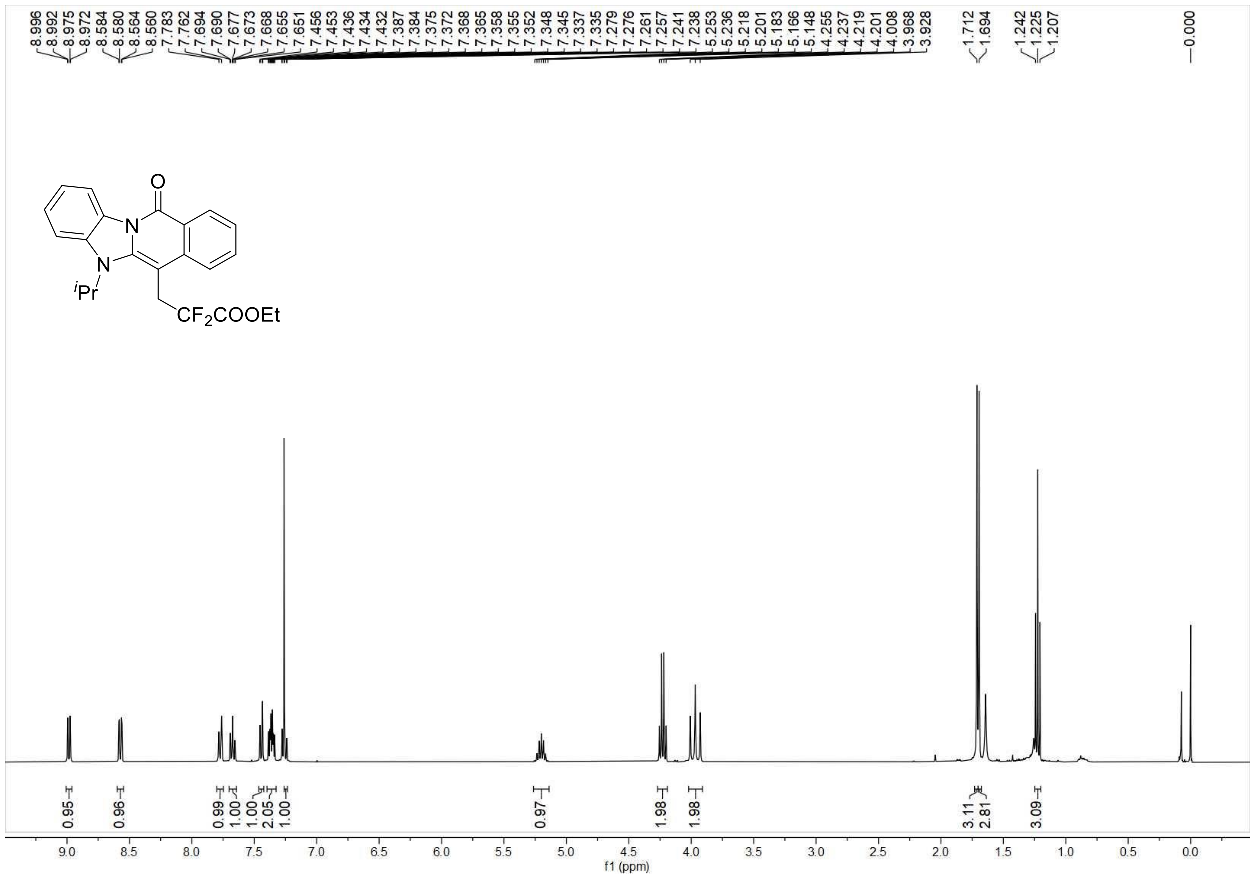

Supplementary Figure 164. ^{1}H-NMR of compound **30**, recorded at 400 MHz and 25 °C in $CDCl_3$.

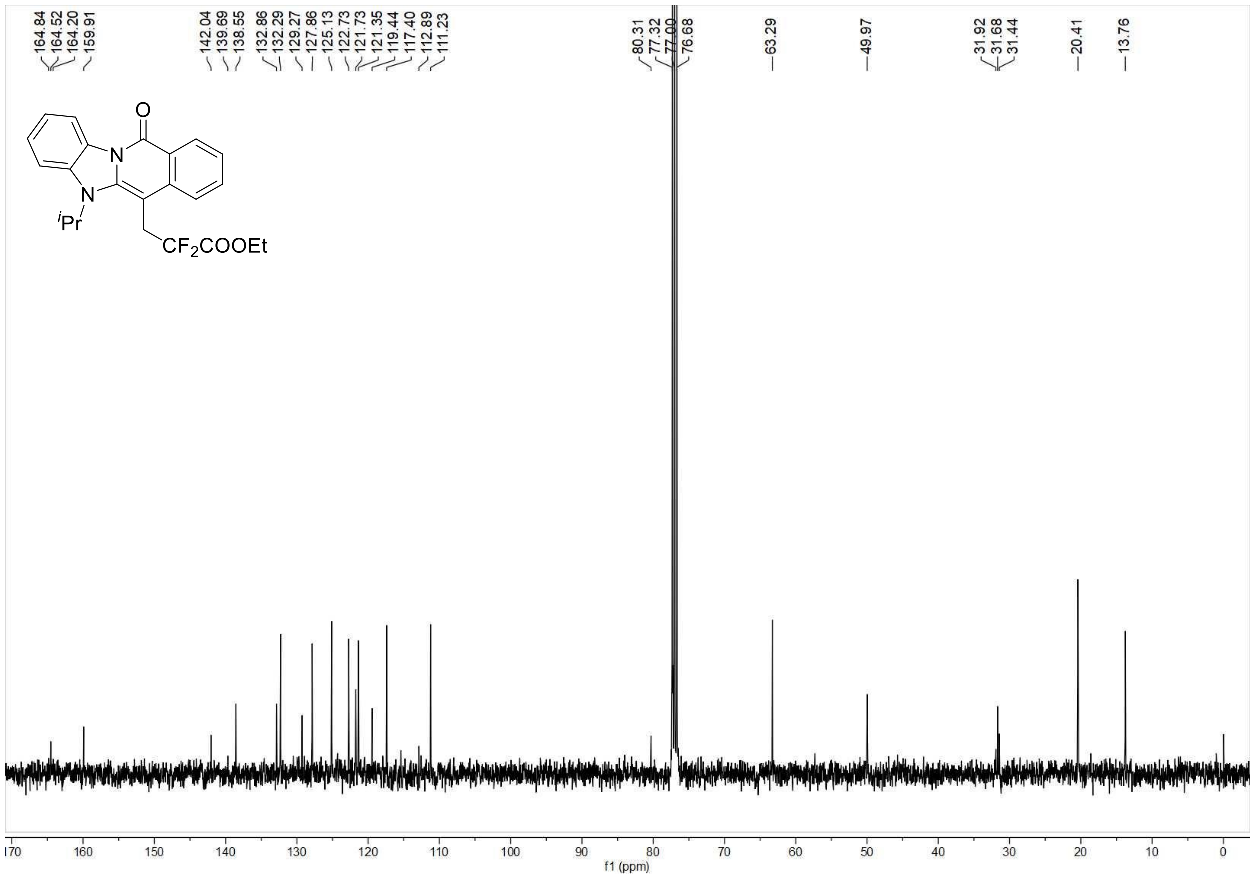

Supplementary Figure 165. ^{13}C-NMR of compound **30**, recorded at 100 MHz and 25 °C in $CDCl_3$.

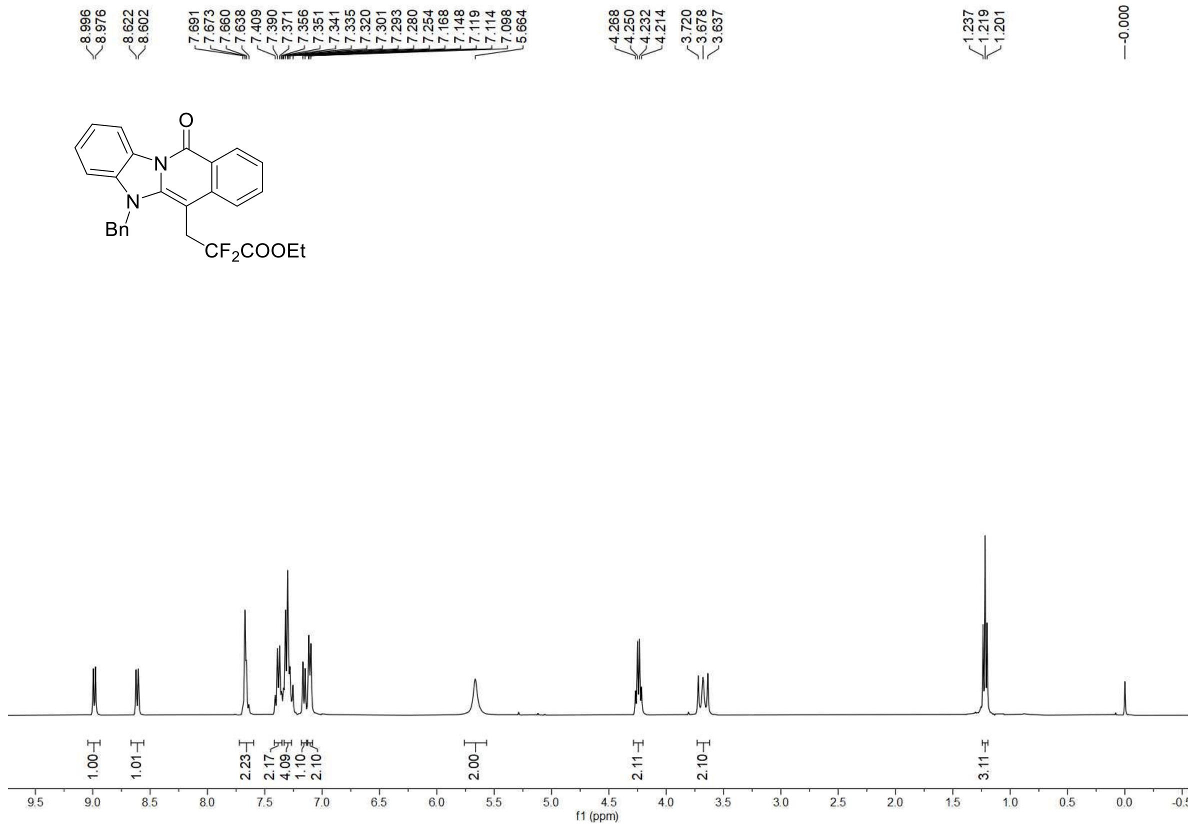

Supplementary Figure 166. ^{1}H-NMR of compound **31**, recorded at 400 MHz and 25 °C in $CDCl_3$.

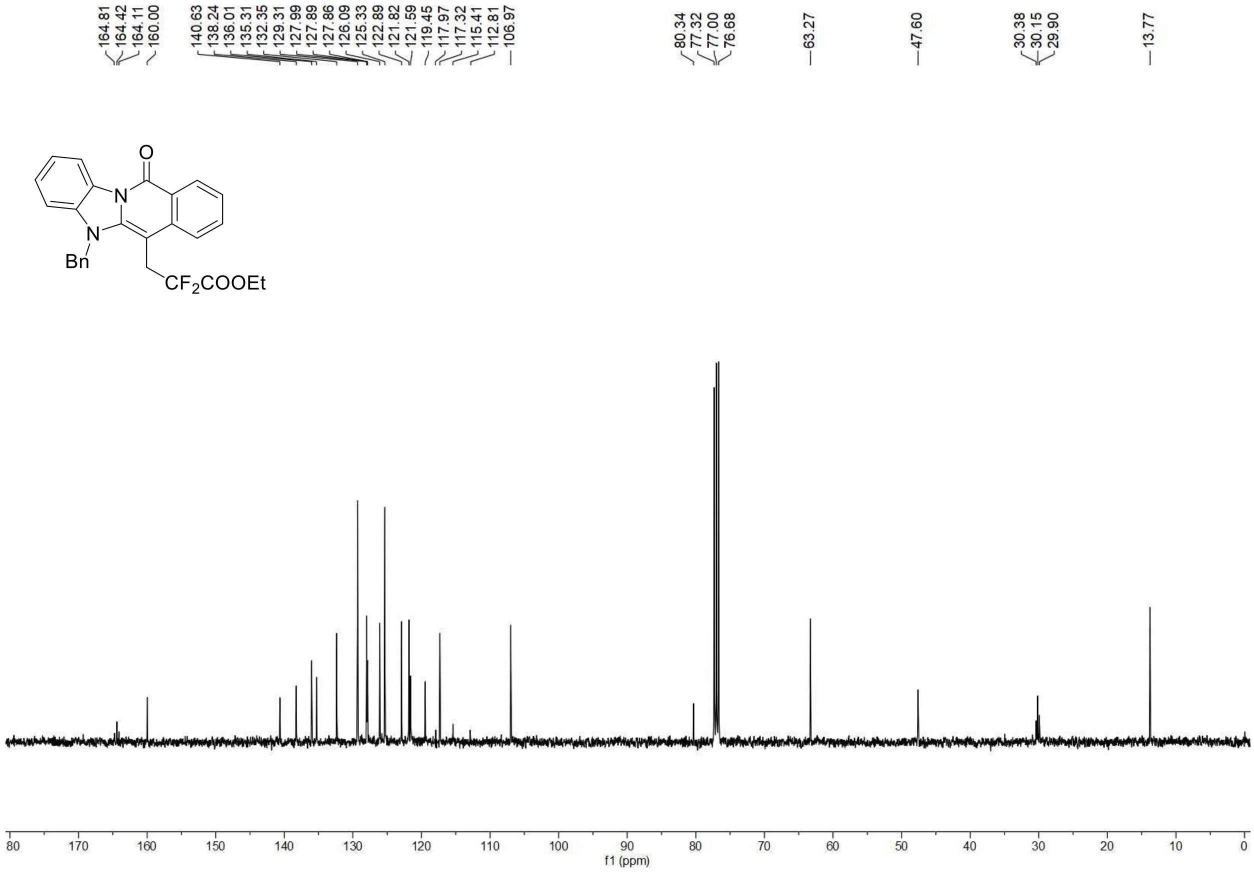

Supplementary Figure 167. ^{13}C-NMR of compound **31**, recorded at 100 MHz and 25 °C in $CDCl_3$.

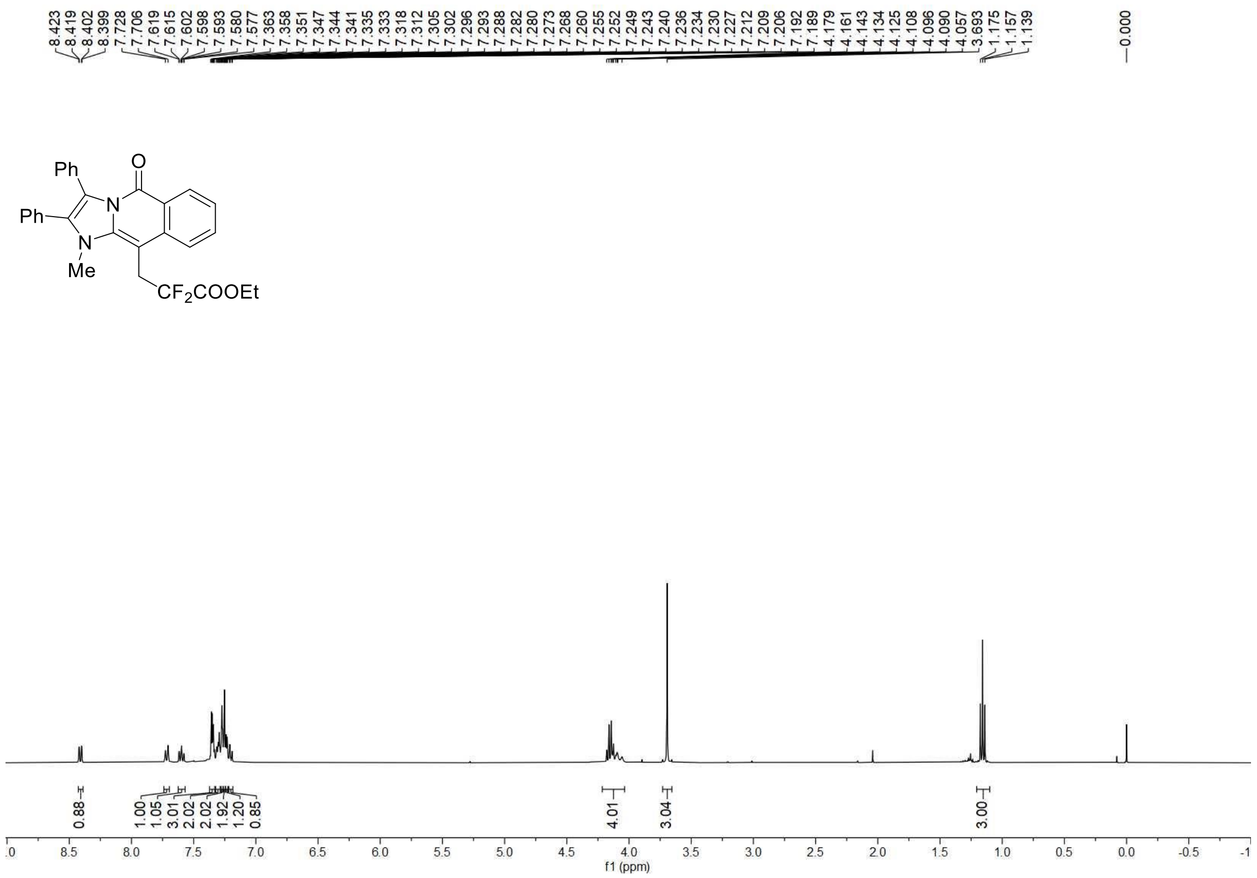

Supplementary Figure 168. ^{1}H-NMR of compound **32**, recorded at 400 MHz and 25 °C in $CDCl_3$.

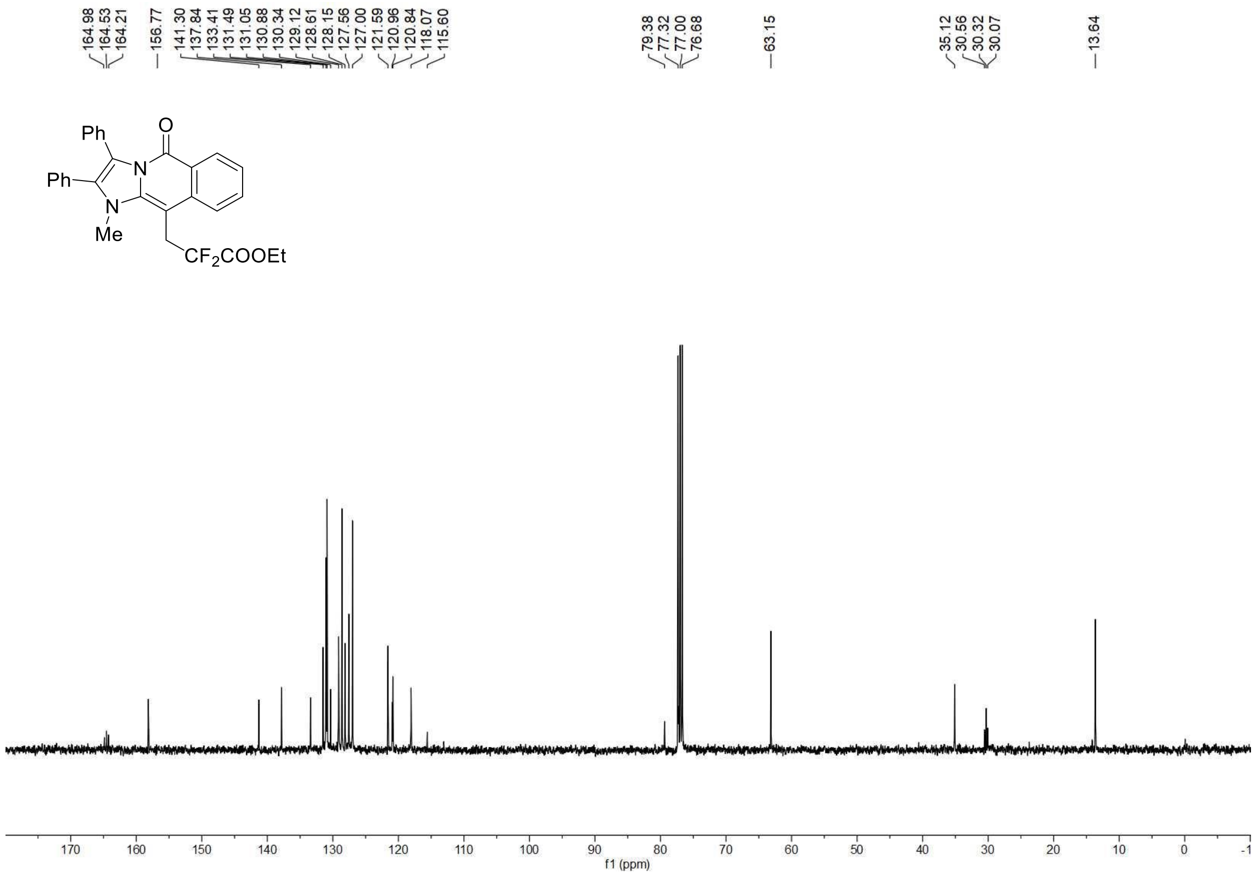

Supplementary Figure 169. ^{13}C-NMR of compound **32**, recorded at 100 MHz and 25 °C in $CDCl_3$.

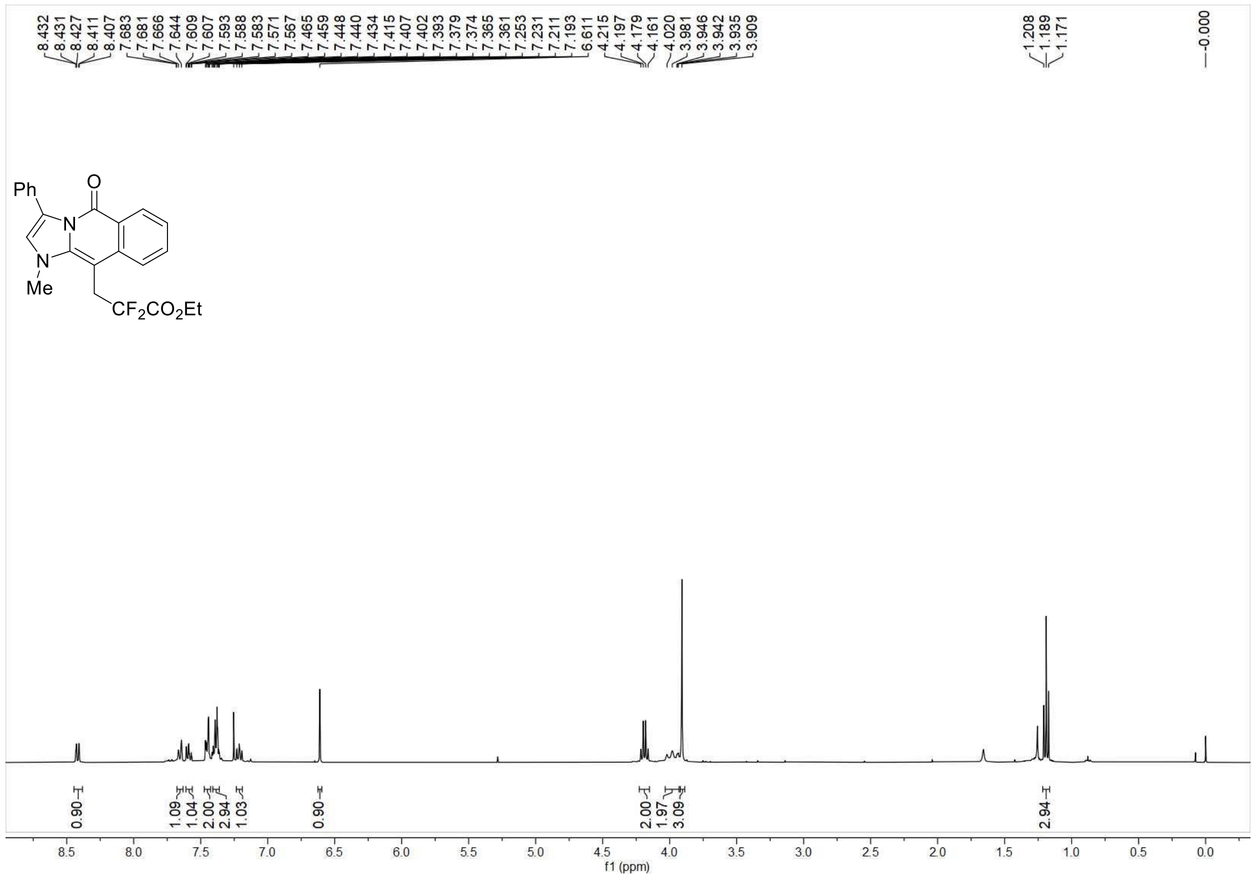

Supplementary Figure 170. ^{1}H-NMR of compound **33**, recorded at 400 MHz and 25 °C in $CDCl_3$.

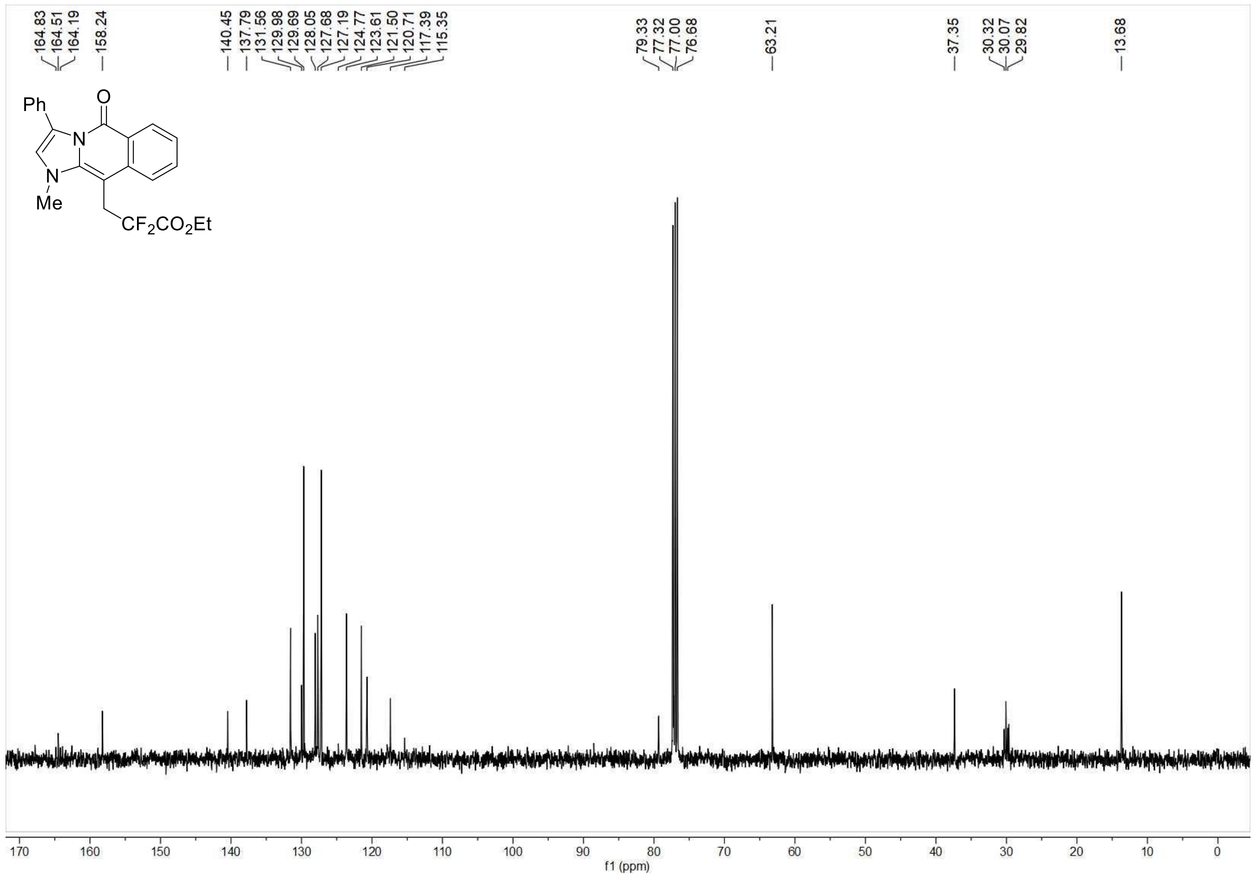

Supplementary Figure 171. ^{13}C-NMR of compound **33**, recorded at 100 MHz and 25 °C in $CDCl_3$.

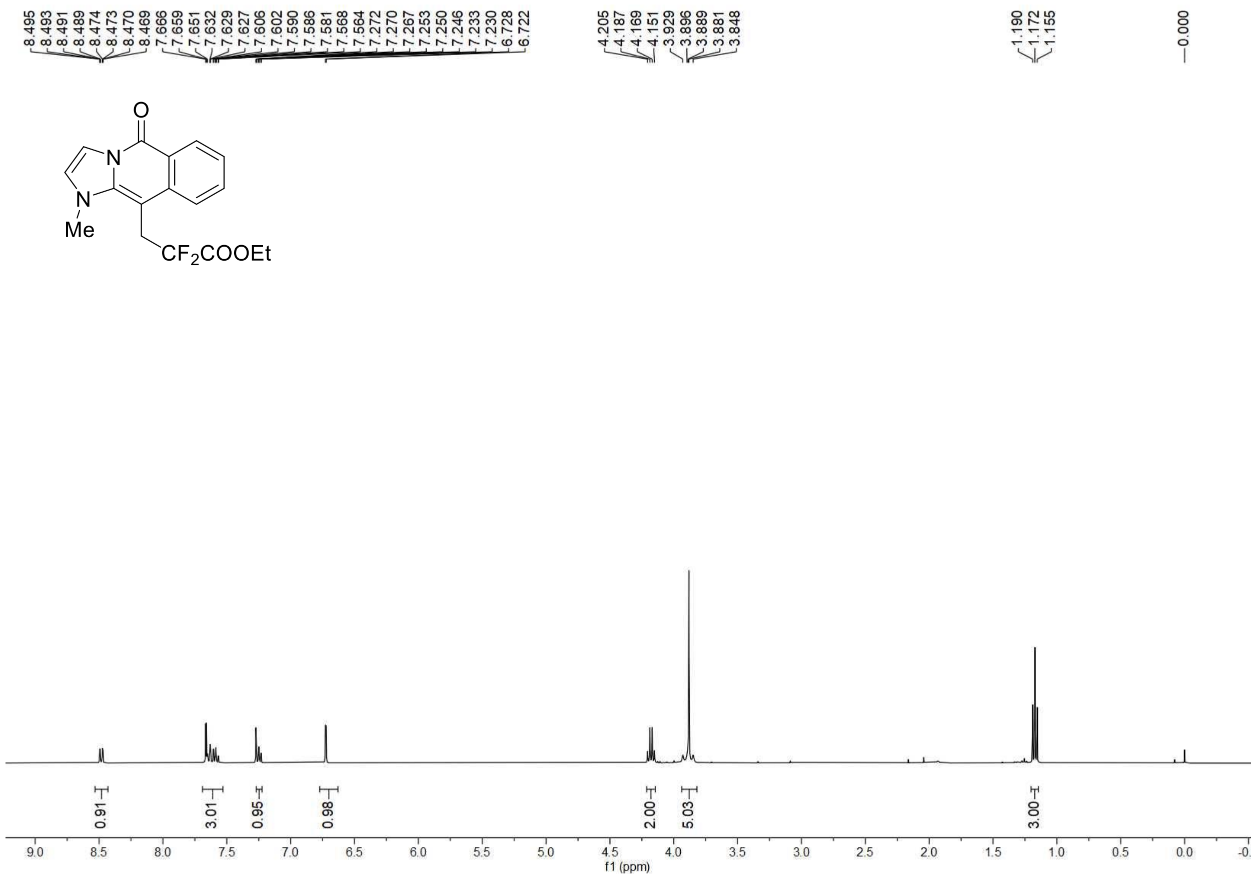

Supplementary Figure 172. ^{1}H-NMR of compound **34**, recorded at 400 MHz and 25 °C in $CDCl_3$.

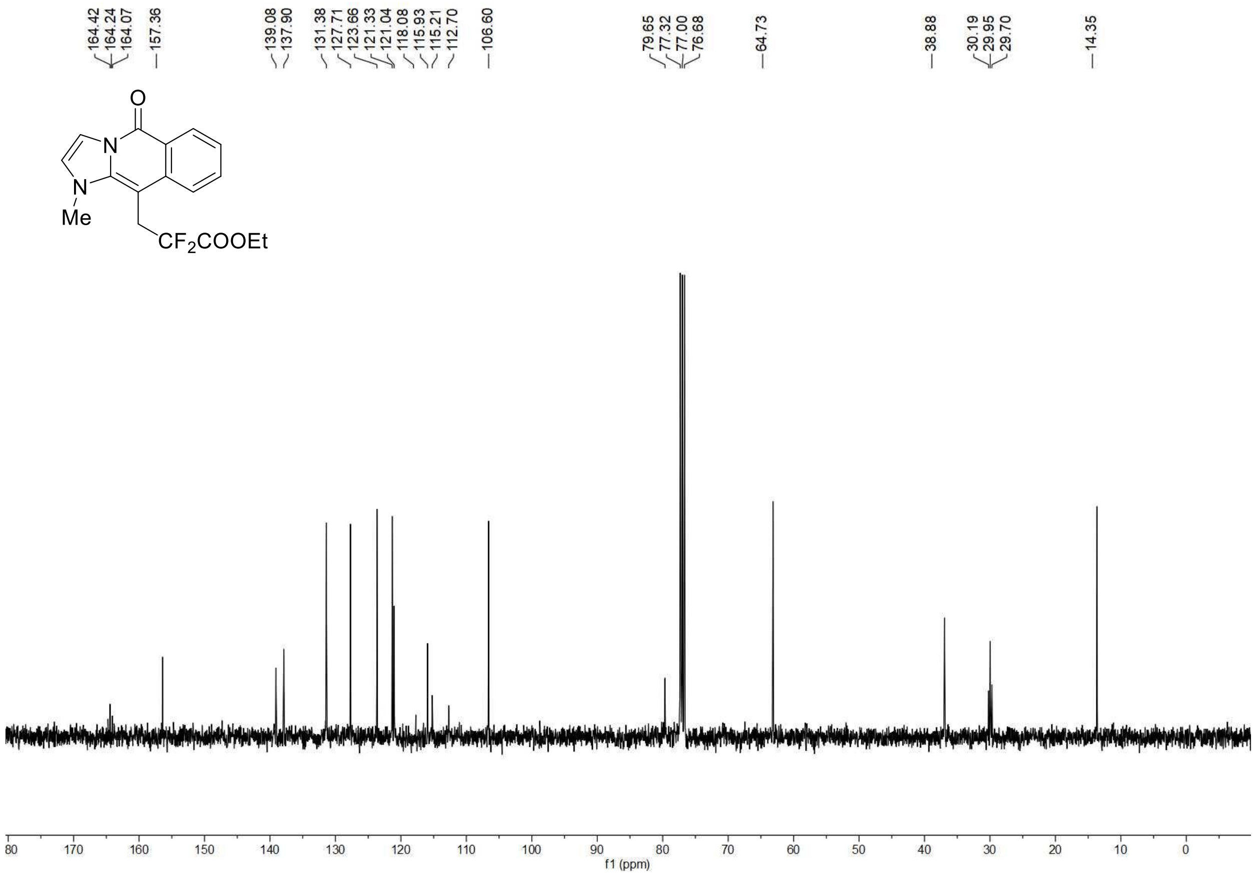

Supplementary Figure 173. ^{13}C-NMR of compound **34**, recorded at 100 MHz and 25 °C in $CDCl_3$.

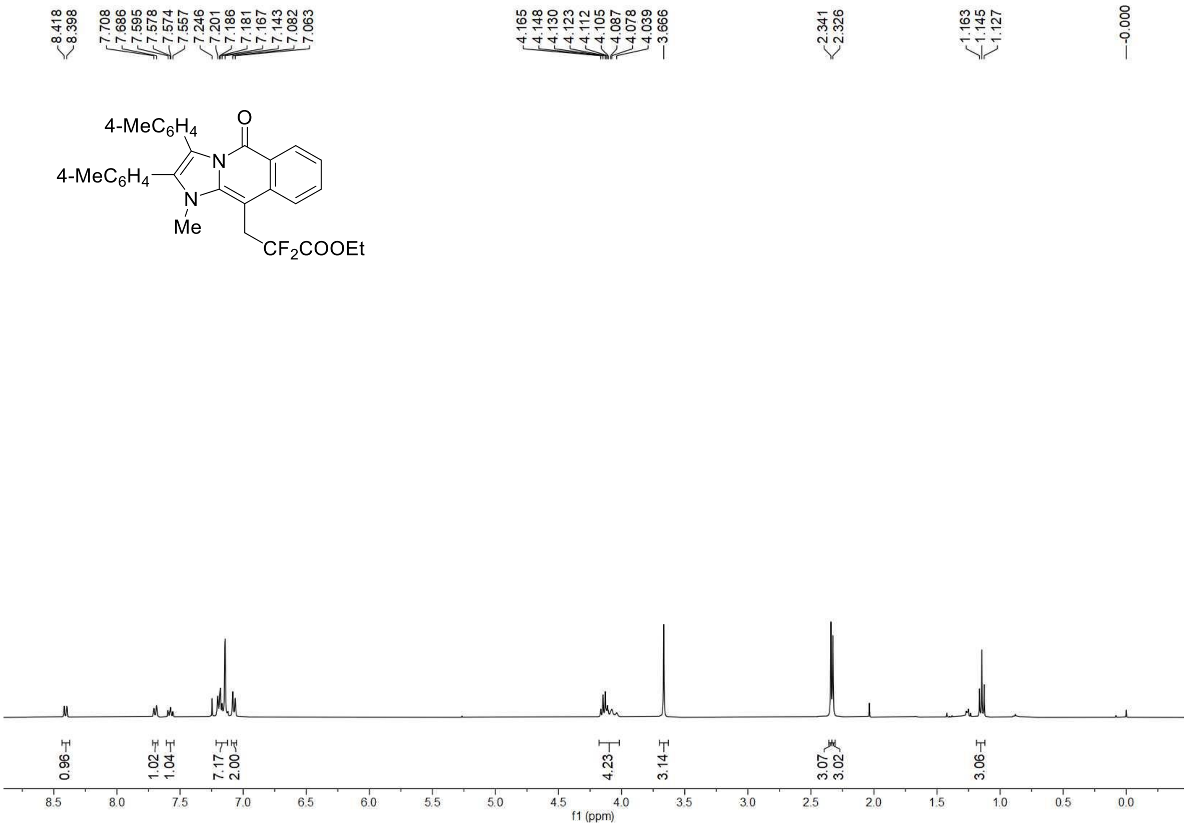

Supplementary Figure 174. ^{1}H-NMR of compound **35**, recorded at 400 MHz and 25 °C in $CDCl_3$.

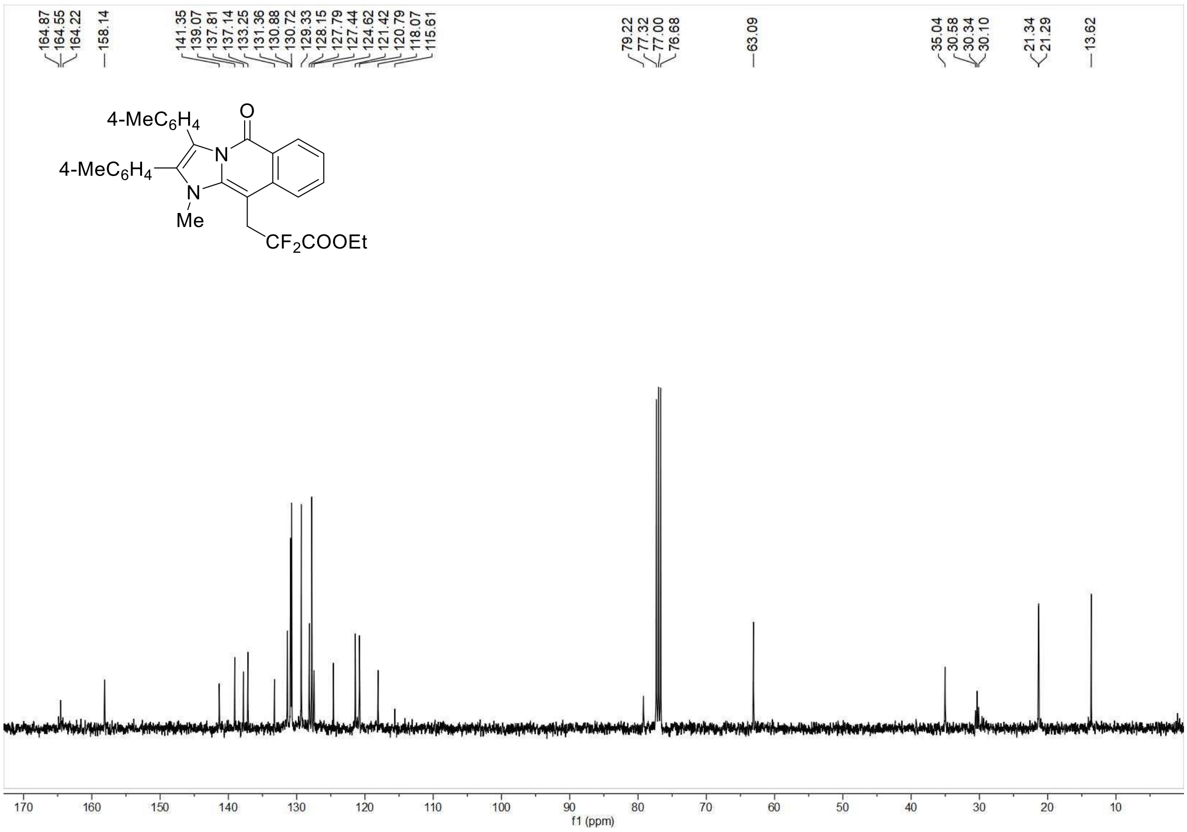

Supplementary Figure 175. ^{13}C-NMR of compound **35**, recorded at 100 MHz and 25 °C in $CDCl_3$.

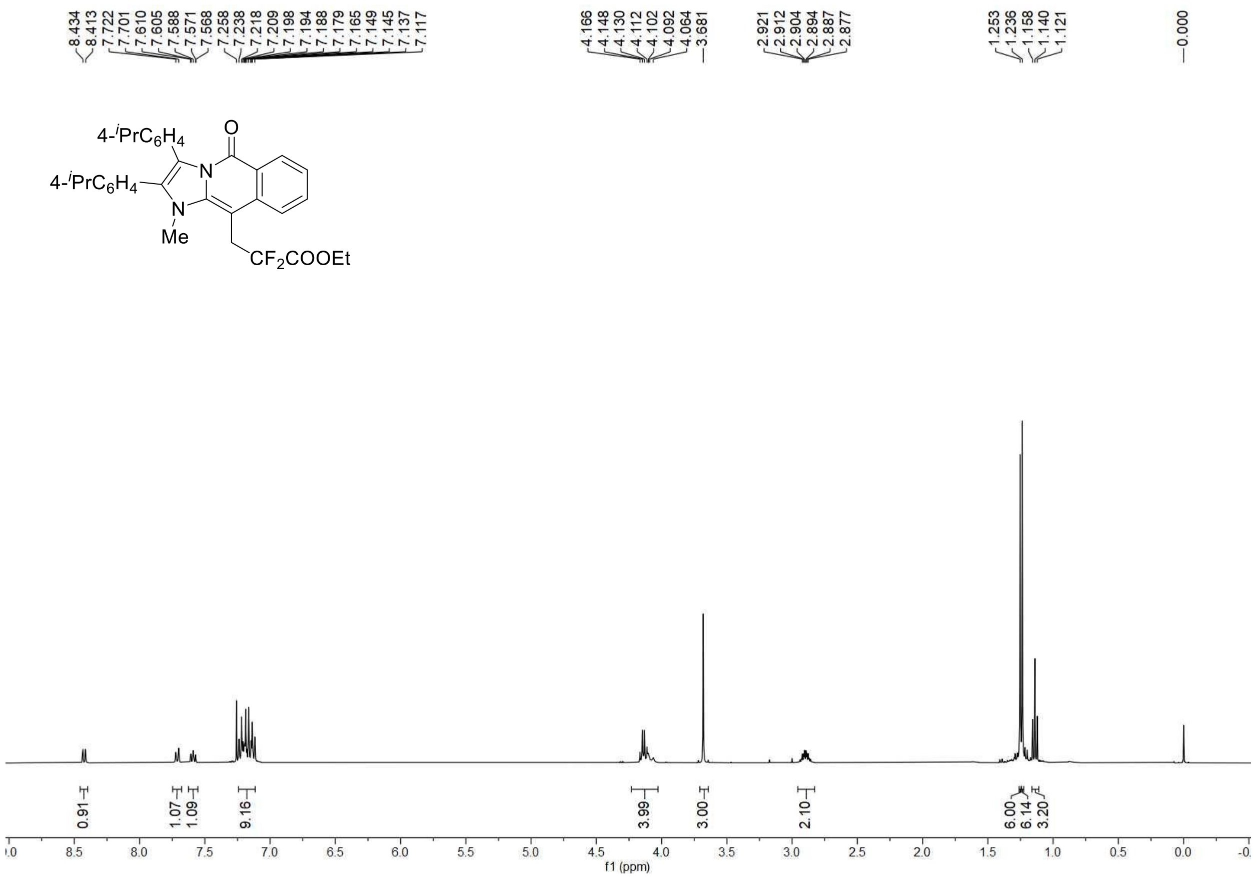

Supplementary Figure 176. ^{1}H-NMR of compound **36**, recorded at 400 MHz and 25 °C in $CDCl_3$.

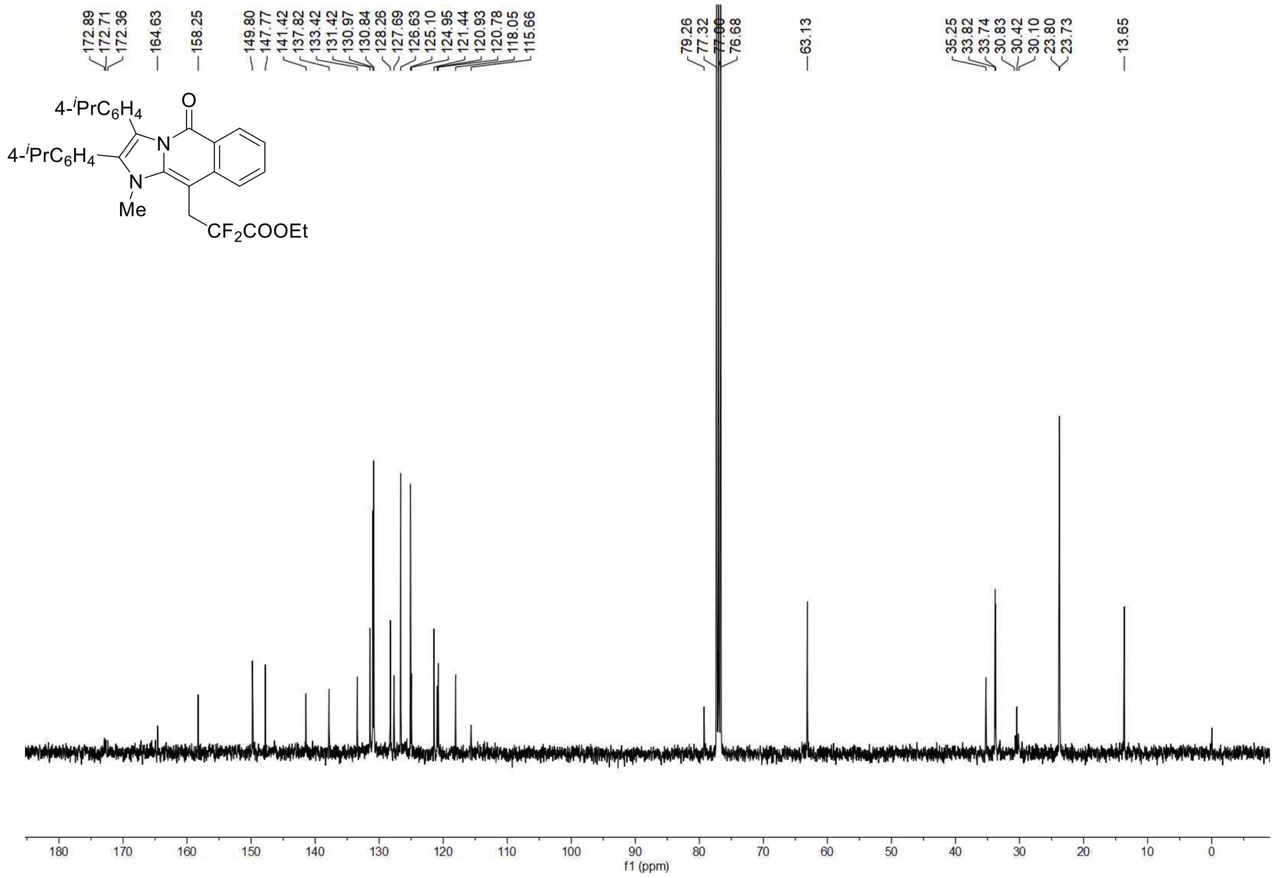

Supplementary Figure 177. ^{13}C-NMR of compound **36**, recorded at 100 MHz and 25 °C in $CDCl_3$.

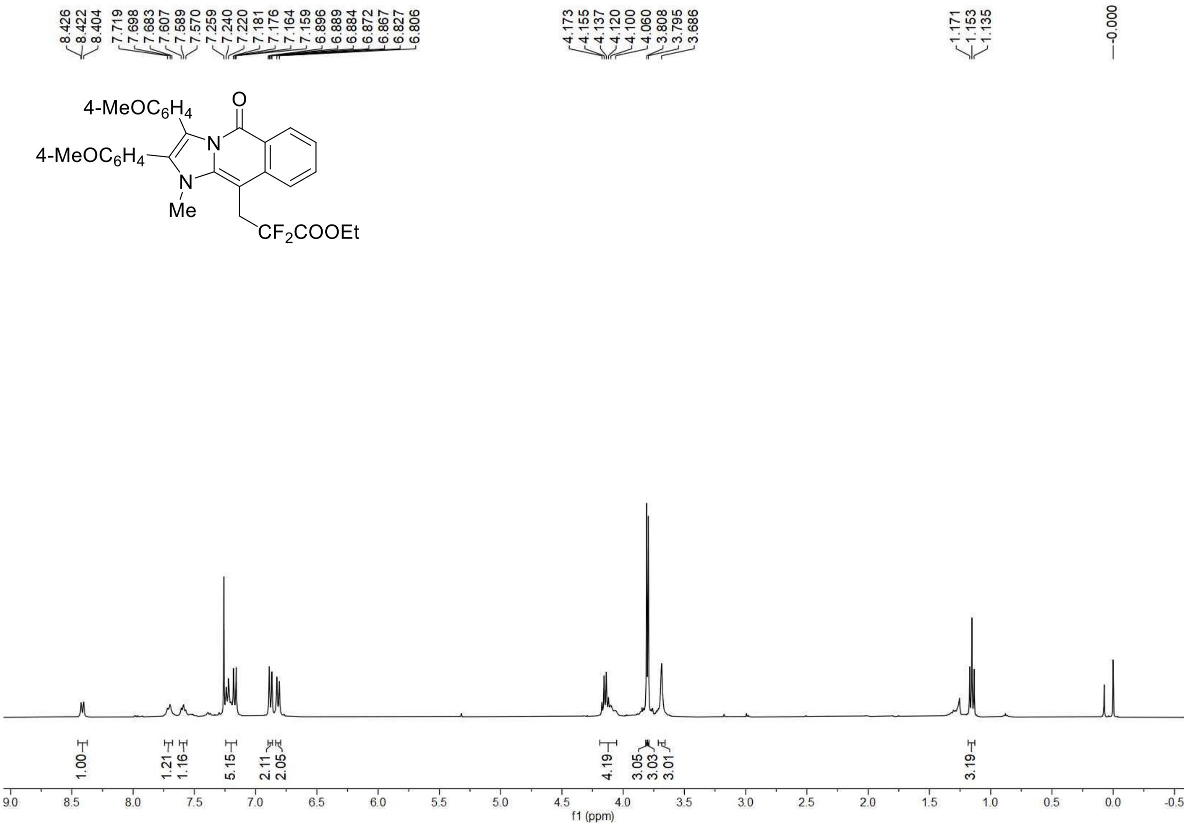

Supplementary Figure 178. ^{1}H-NMR of compound **37**, recorded at 400 MHz and 25 °C in $CDCl_3$.

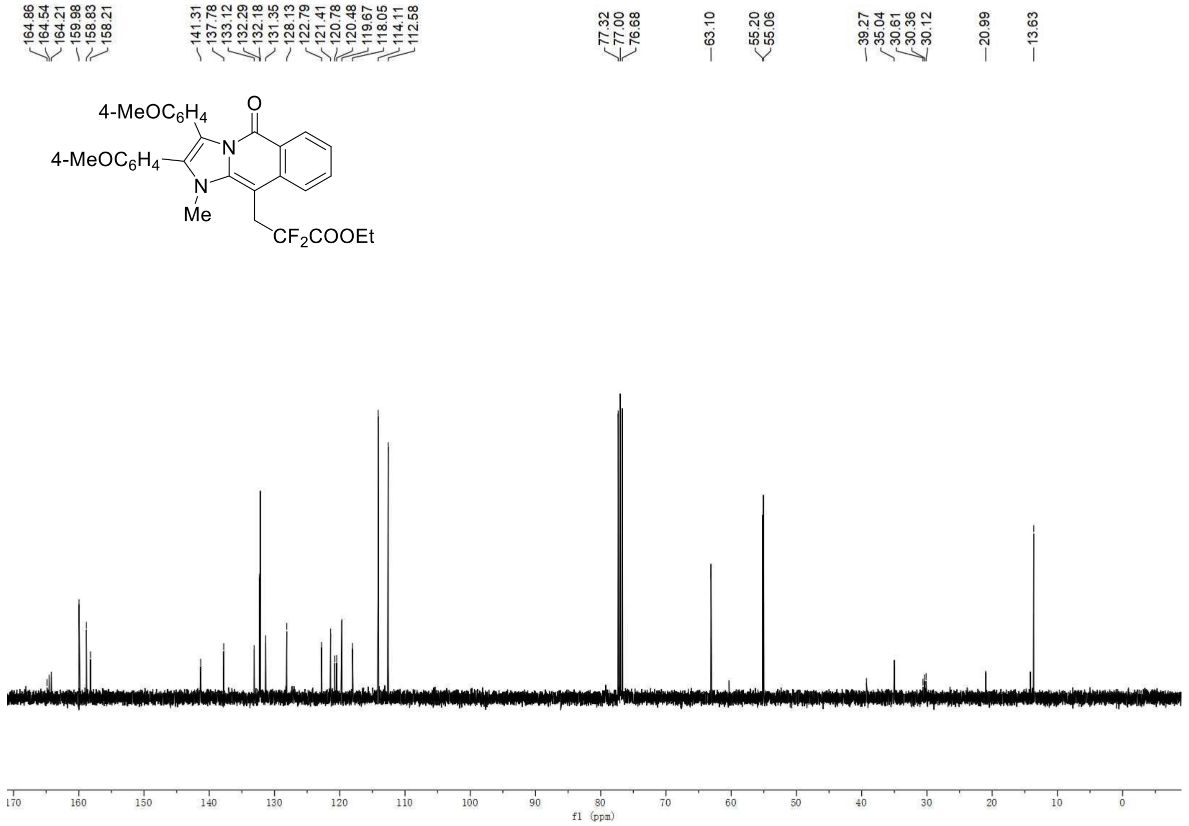

Supplementary Figure 179. ^{13}C-NMR of compound **37**, recorded at 100 MHz and 25 °C in $CDCl_3$.

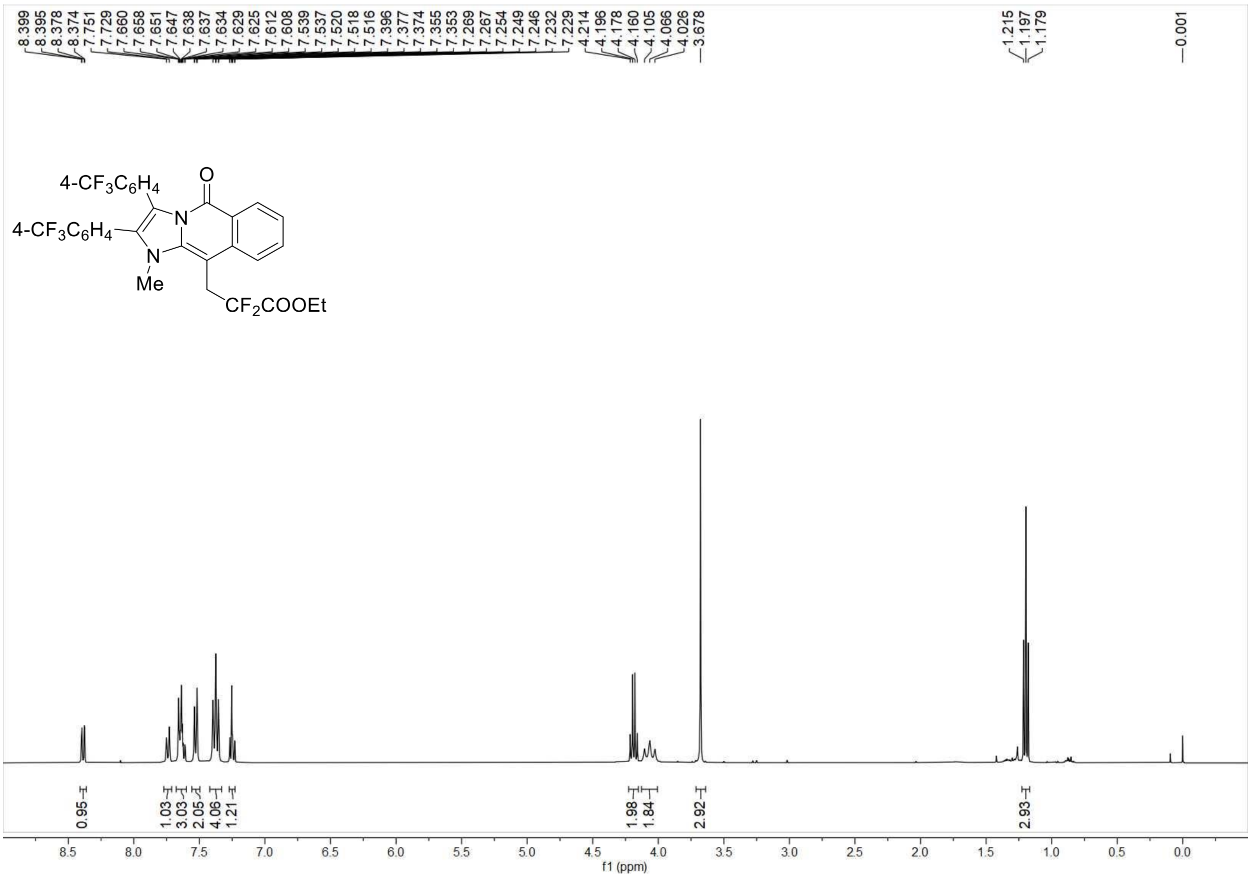

Supplementary Figure 180. ^{1}H-NMR of compound **38**, recorded at 400 MHz and 25 °C in $CDCl_3$.

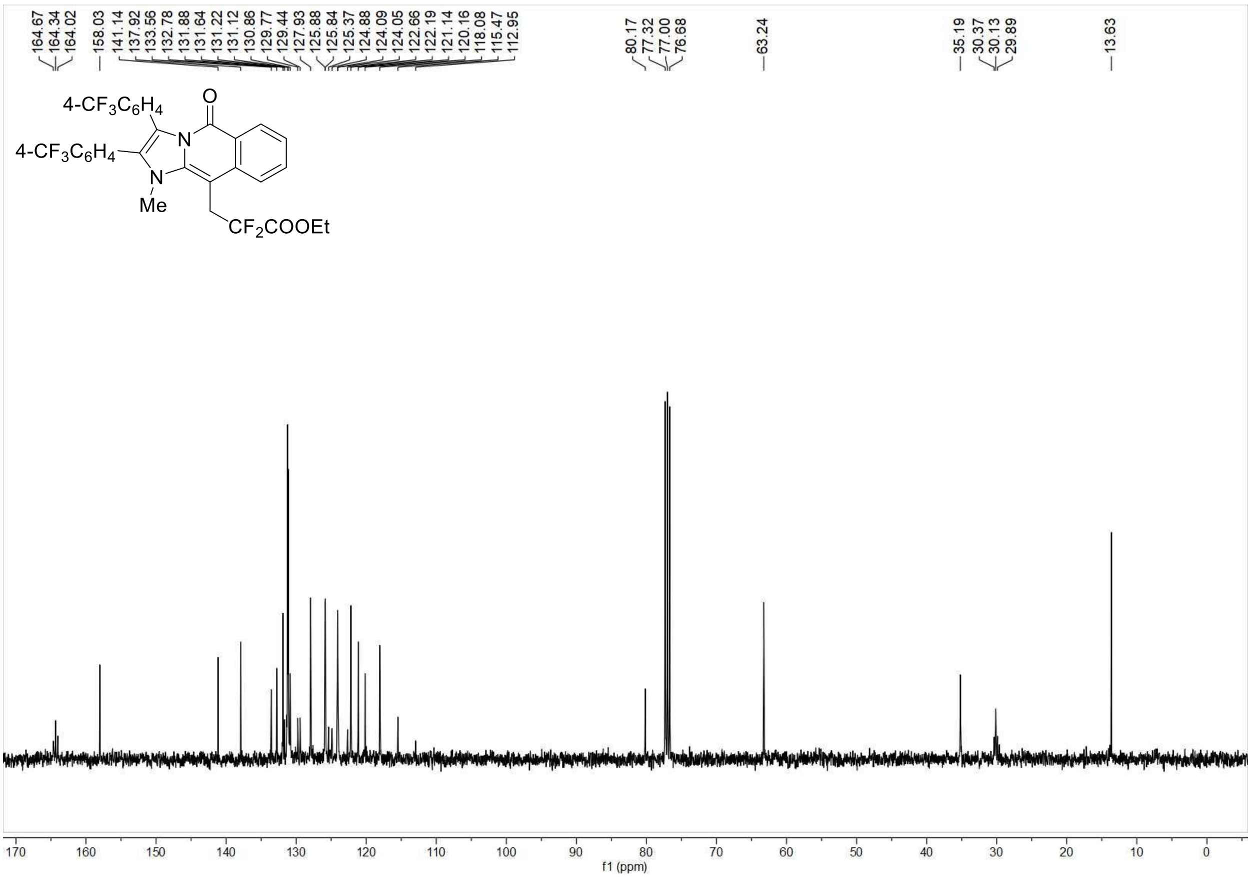

Supplementary Figure 181. ^{13}C-NMR of compound **38**, recorded at 100 MHz and 25 °C in $CDCl_3$.

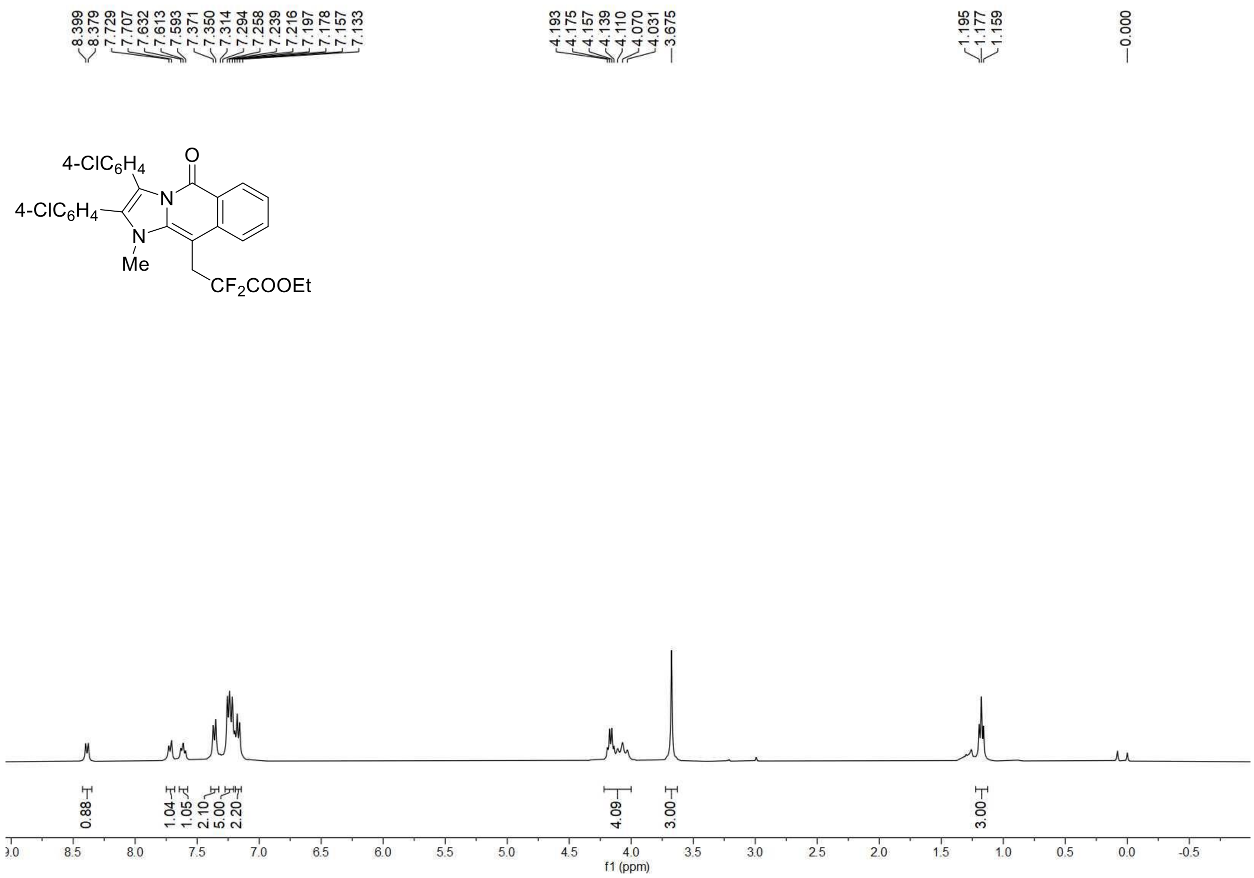

Supplementary Figure 182. ^{1}H-NMR of compound **39**, recorded at 400 MHz and 25 °C in $CDCl_3$.

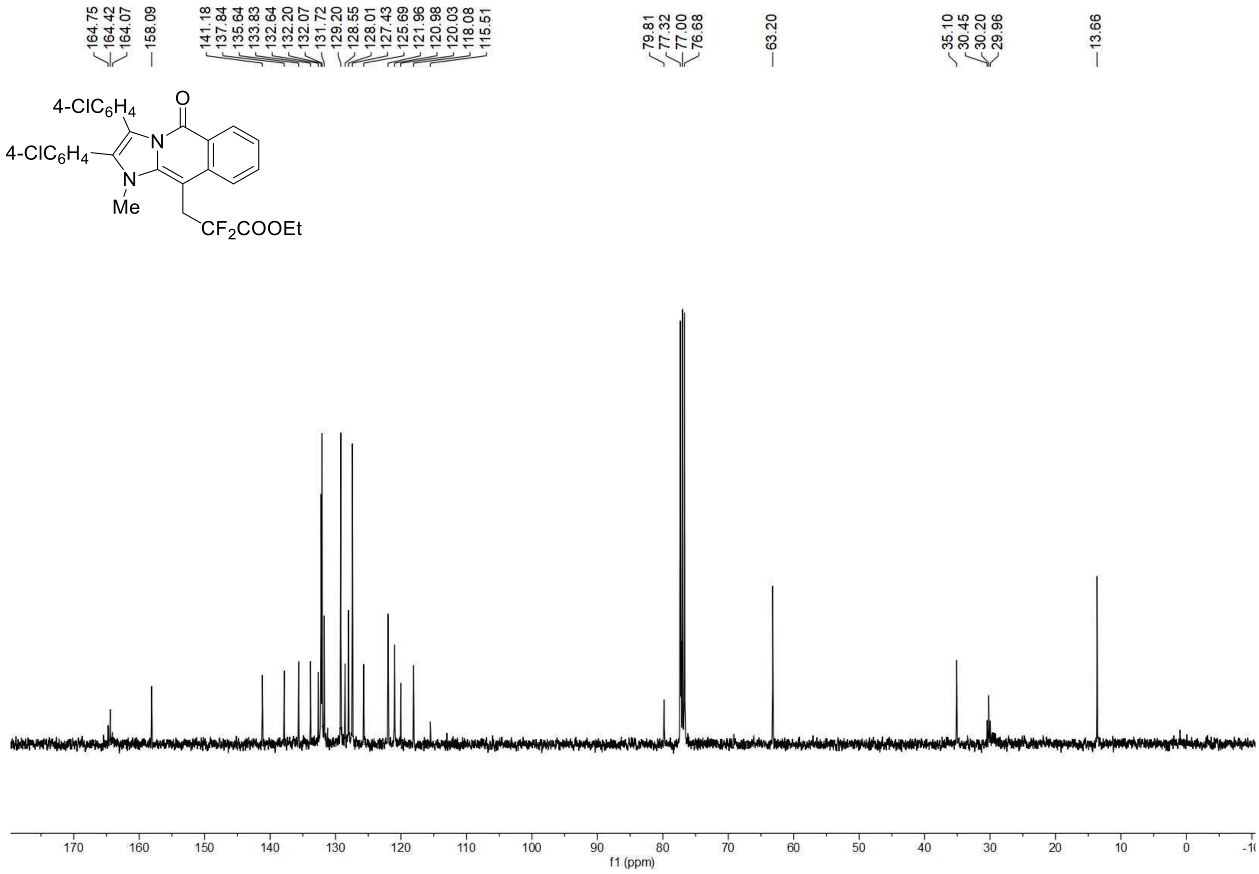

Supplementary Figure 183. ^{13}C-NMR of compound **39**, recorded at 100 MHz and 25 °C in $CDCl_3$.

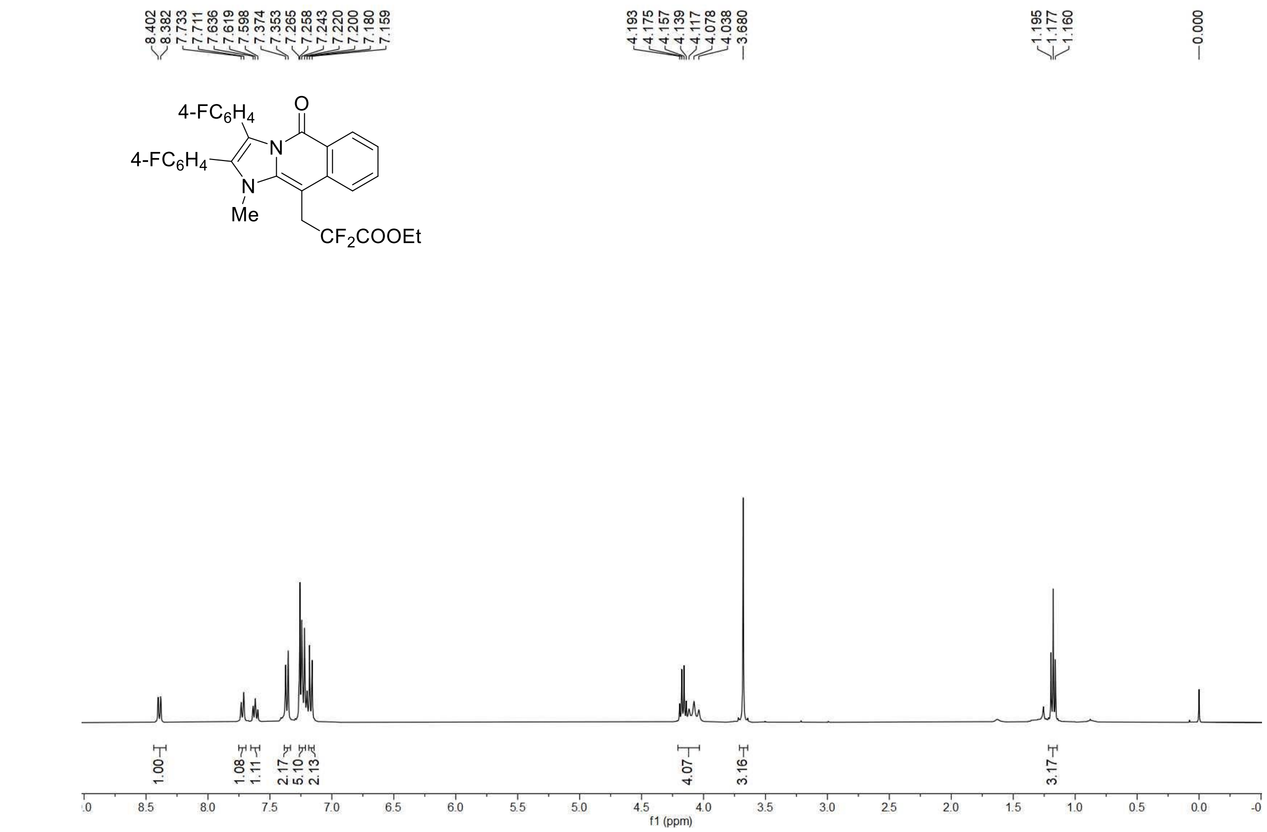

Supplementary Figure 184. ^{1}H-NMR of compound **40**, recorded at 400 MHz and 25 °C in $CDCl_3$.

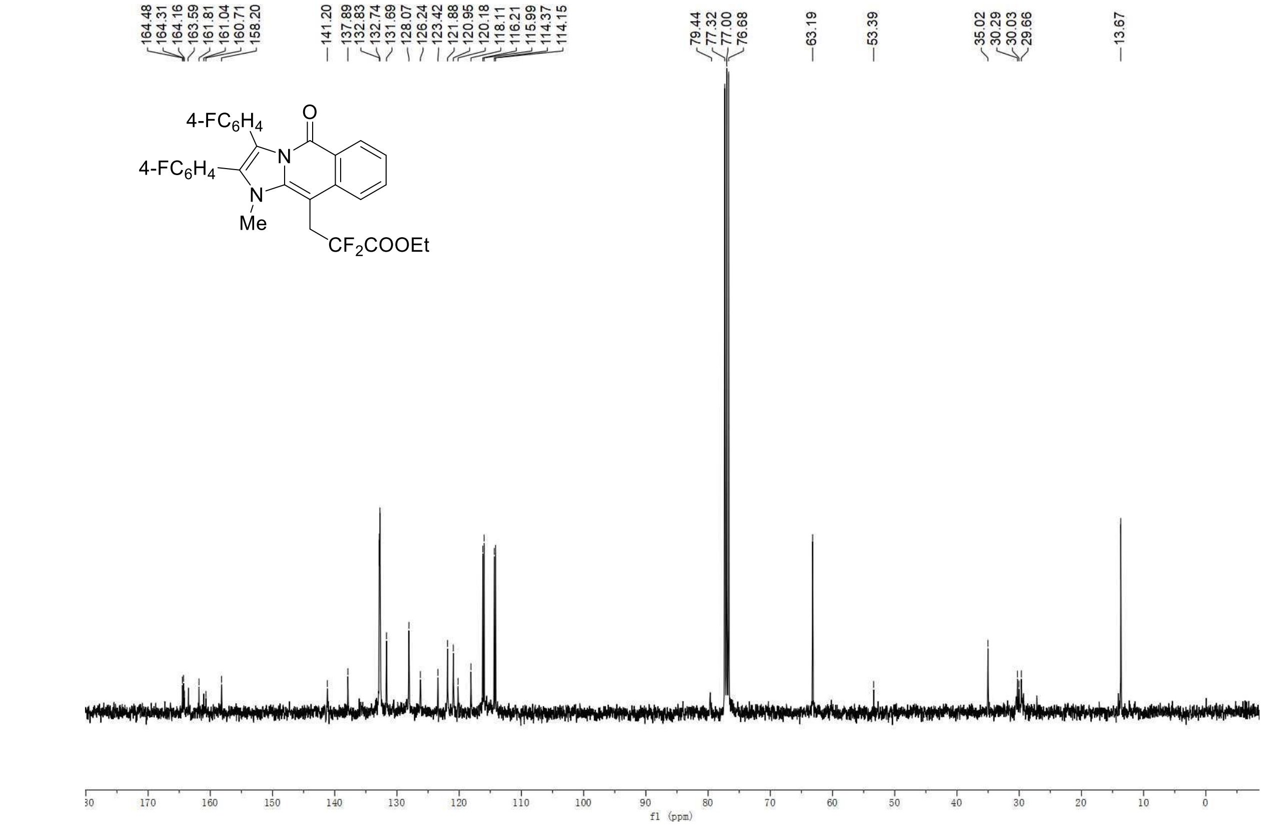

Supplementary Figure 185. ^{13}C-NMR of compound **40**, recorded at 100 MHz and 25 °C in $CDCl_3$.

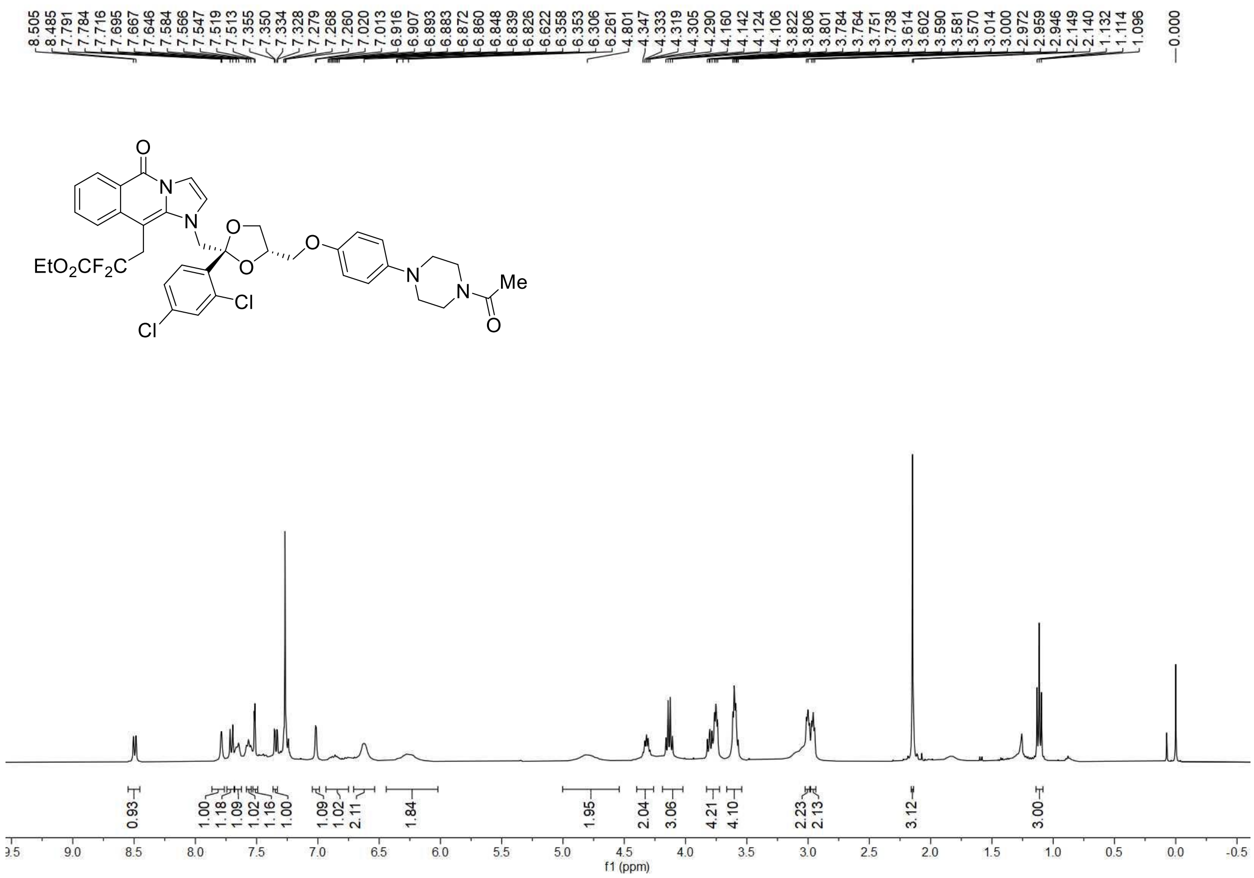

Supplementary Figure 186. ^{1}H-NMR of compound **41**, recorded at 400 MHz and 25 °C in $CDCl_3$.

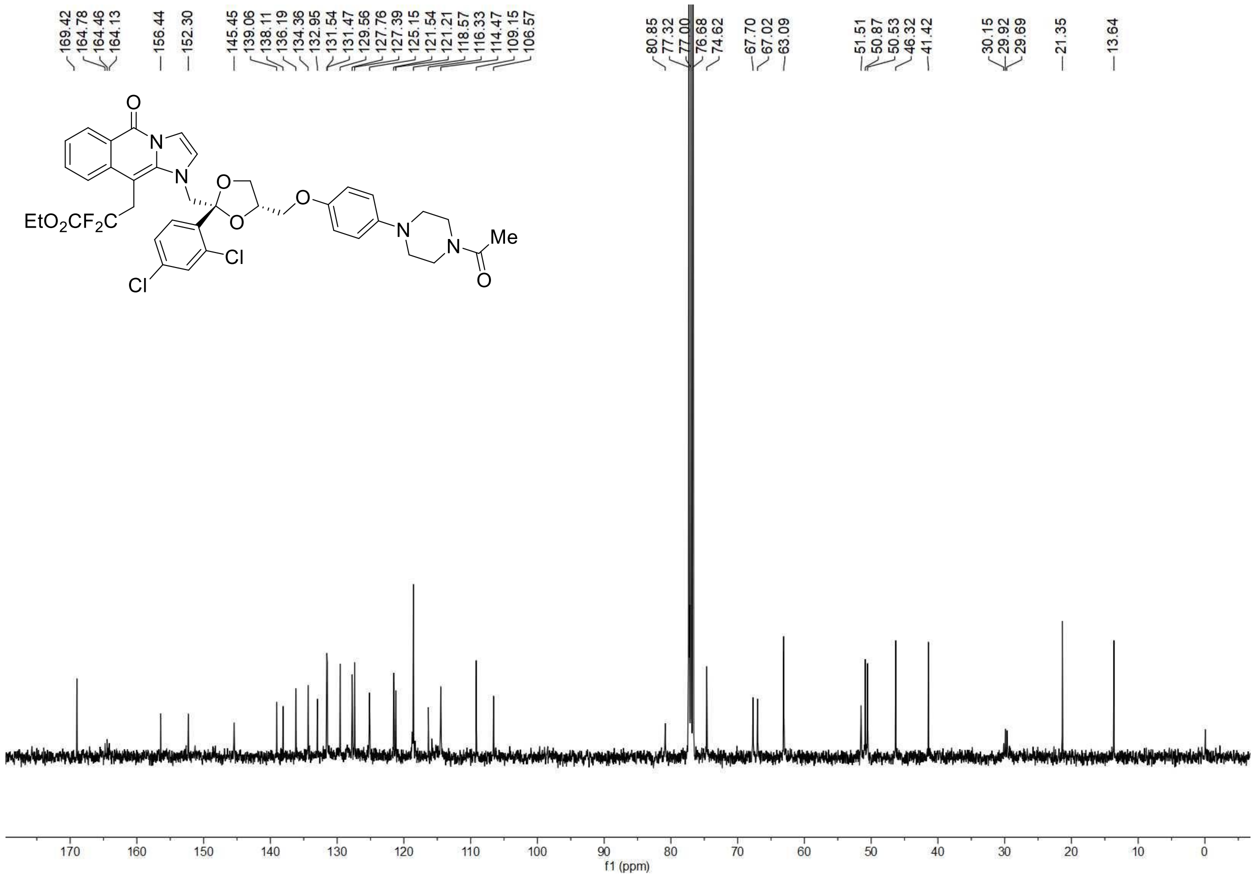

Supplementary Figure 187. ^{13}C-NMR of compound **41**, recorded at 100 MHz and 25 °C in $CDCl_3$.

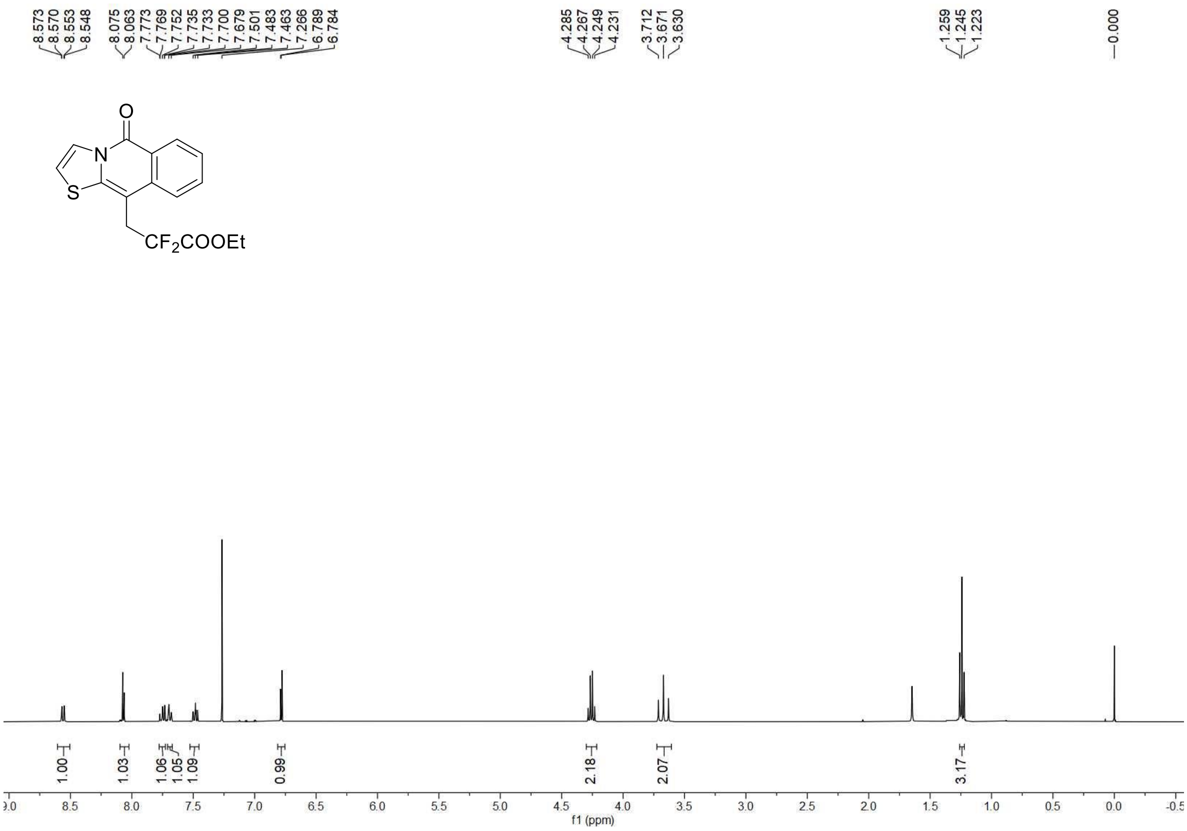

Supplementary Figure 188. ^{1}H-NMR of compound **42**, recorded at 400 MHz and 25 °C in $CDCl_3$.

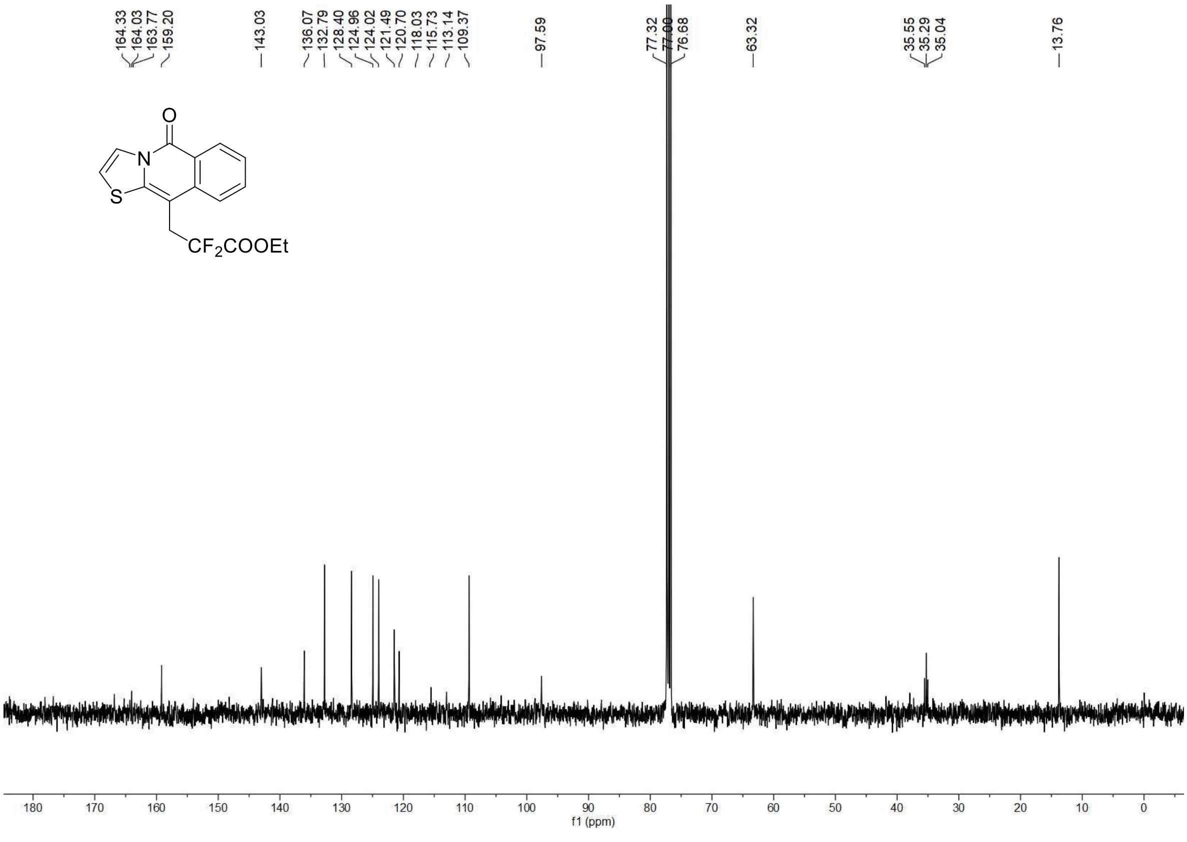

Supplementary Figure 189. ^{13}C-NMR of compound **42**, recorded at 100 MHz and 25 °C in $CDCl_3$.

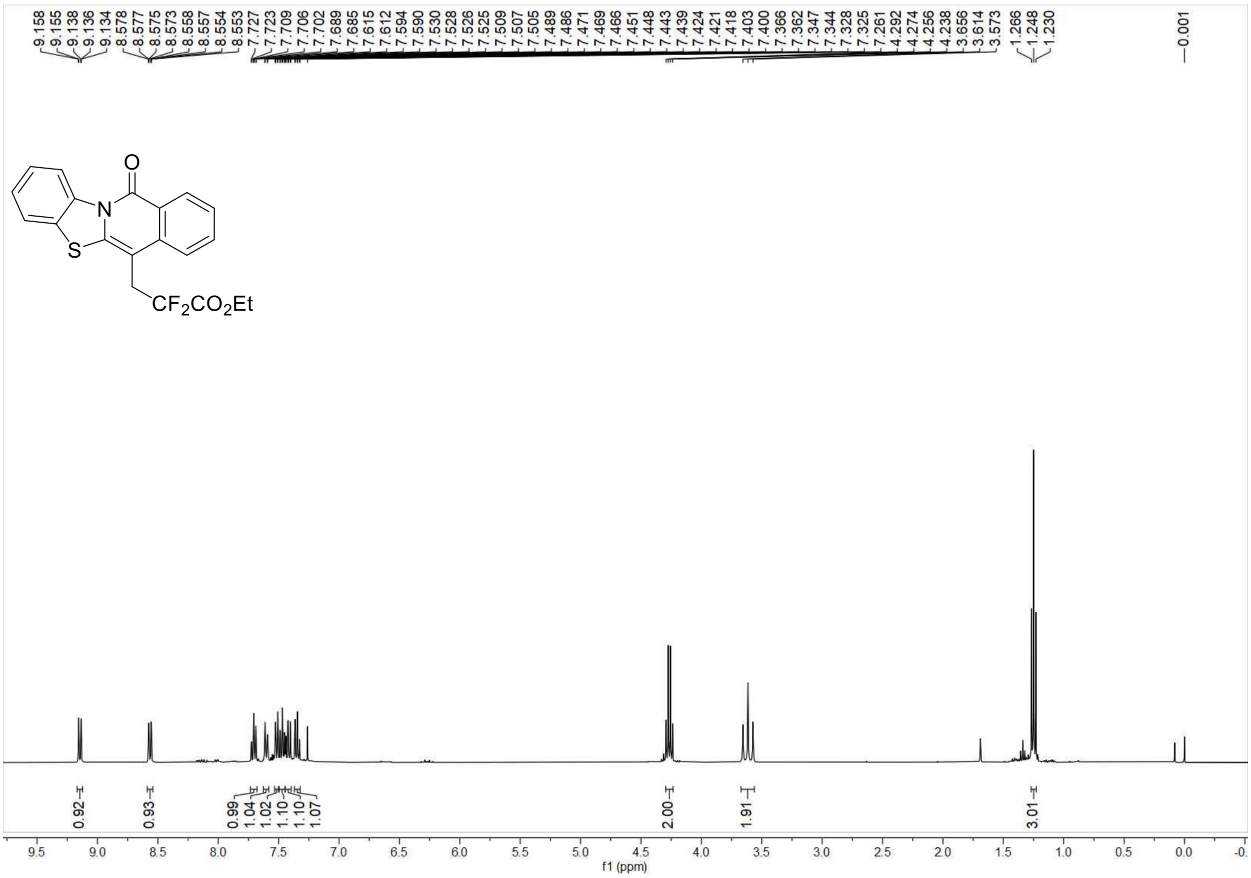

Supplementary Figure 190. ^{1}H-NMR of compound **43**, recorded at 400 MHz and 25 °C in $CDCl_3$.

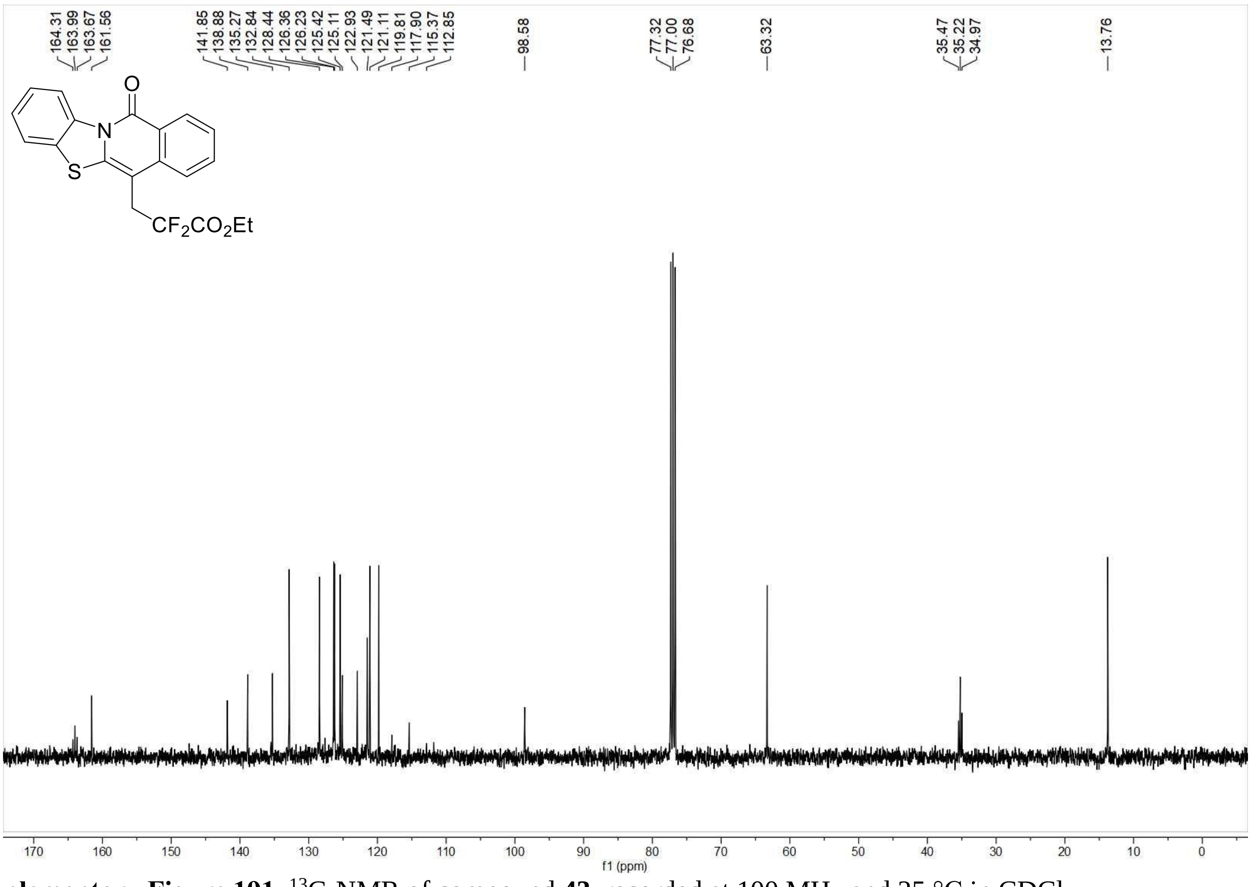

Supplementary Figure 191. ^{13}C-NMR of compound **43**, recorded at 100 MHz and 25 °C in $CDCl_3$.

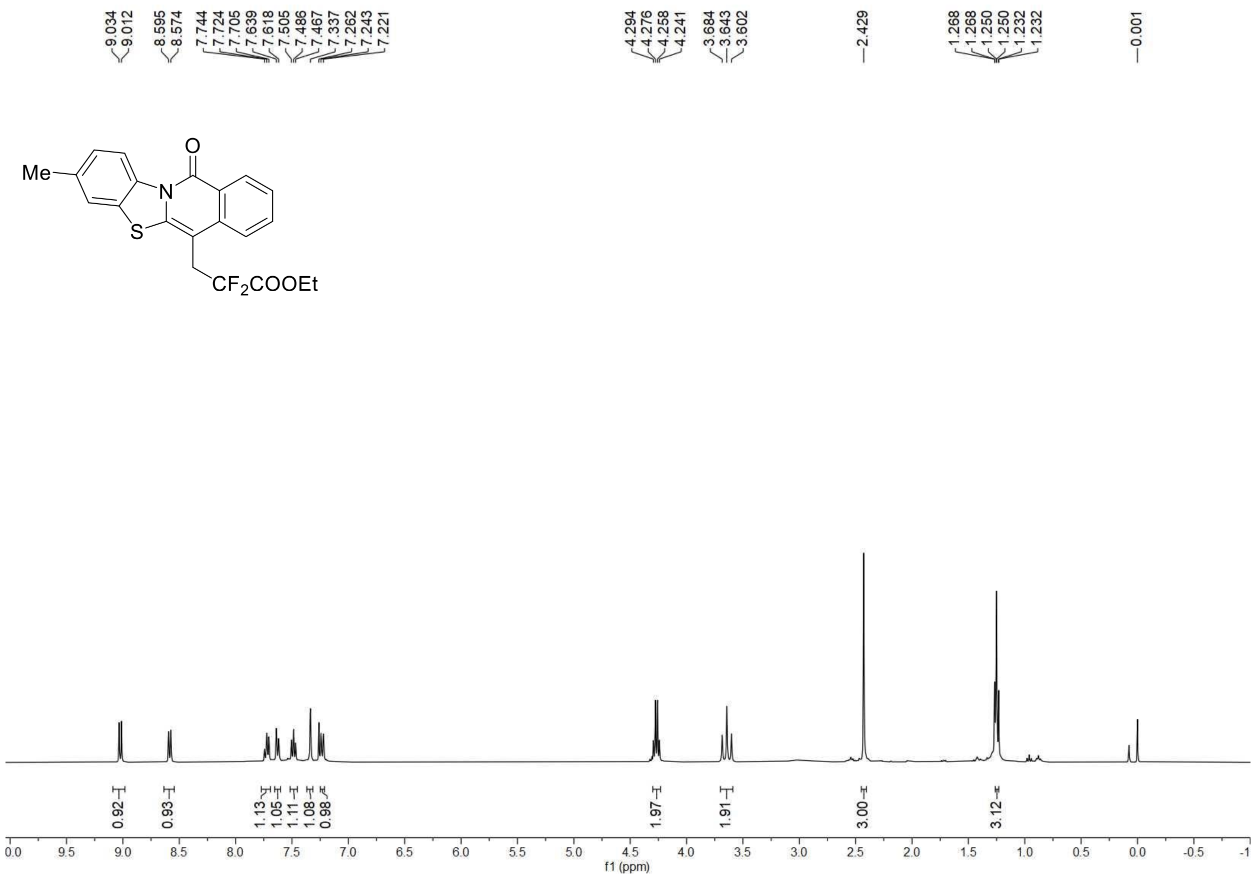

Supplementary Figure 192. ^{1}H-NMR of compound **44**, recorded at 400 MHz and 25 °C in $CDCl_3$.

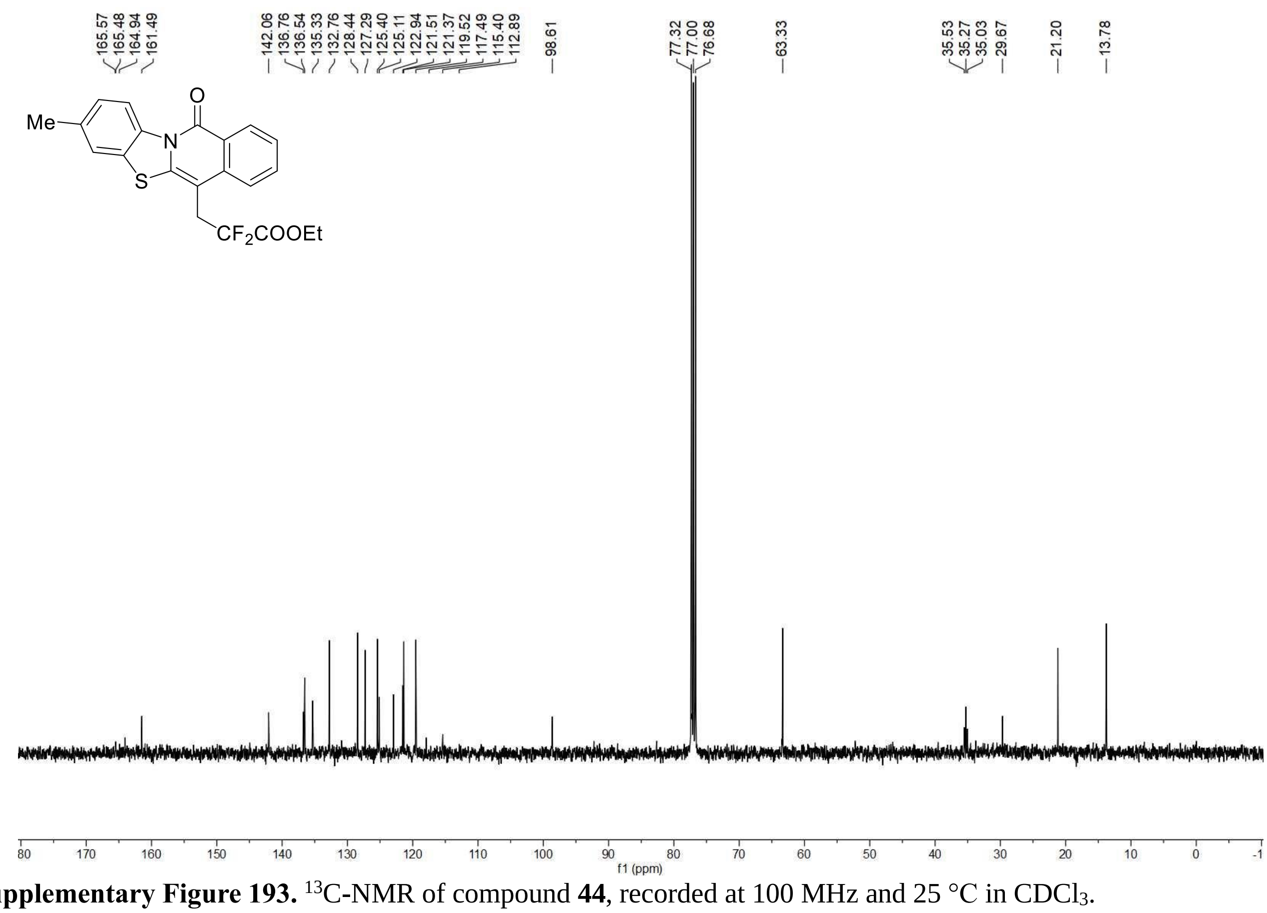

Supplementary Figure 193. ^{13}C-NMR of compound **44**, recorded at 100 MHz and 25 °C in $CDCl_3$.

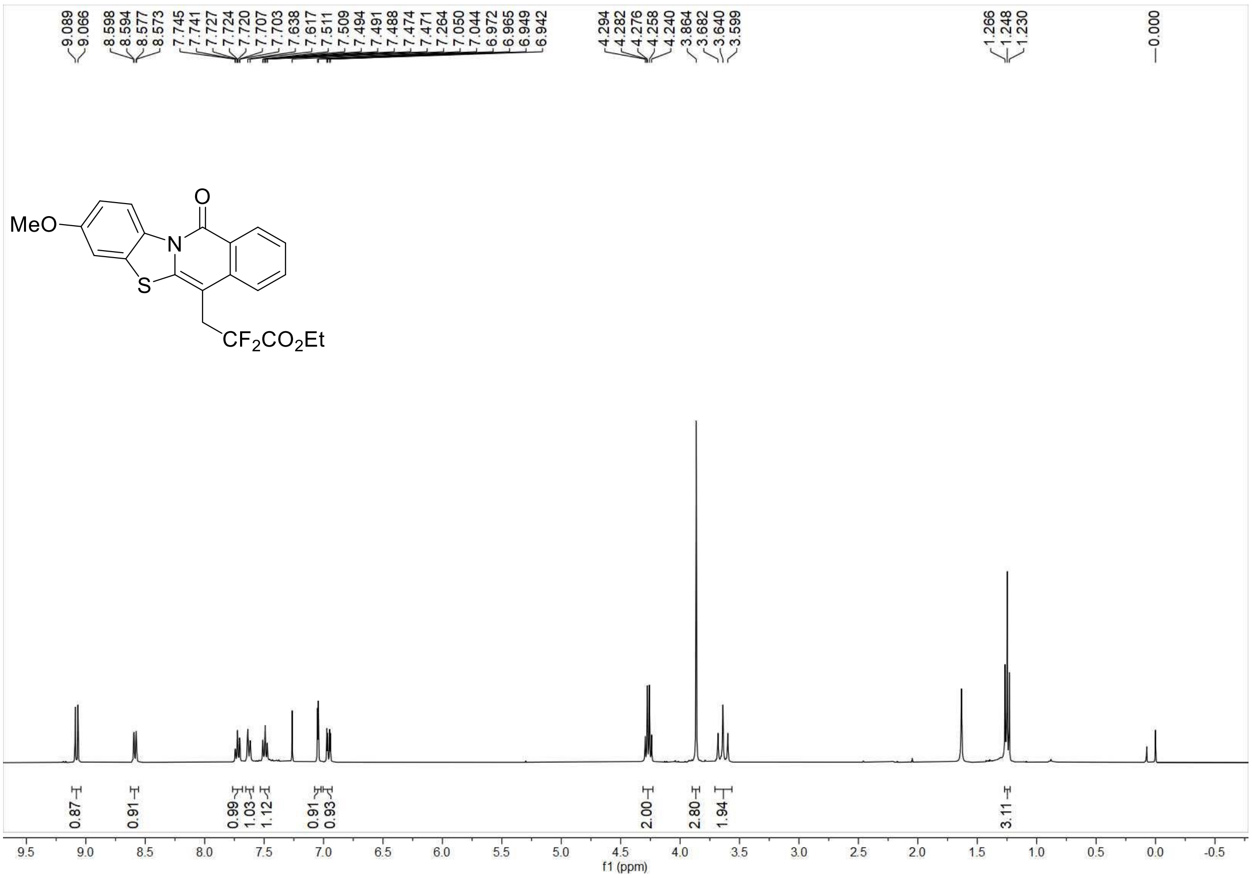

Supplementary Figure 194. ^{1}H-NMR of compound **45**, recorded at 400 MHz and 25 °C in $CDCl_3$.

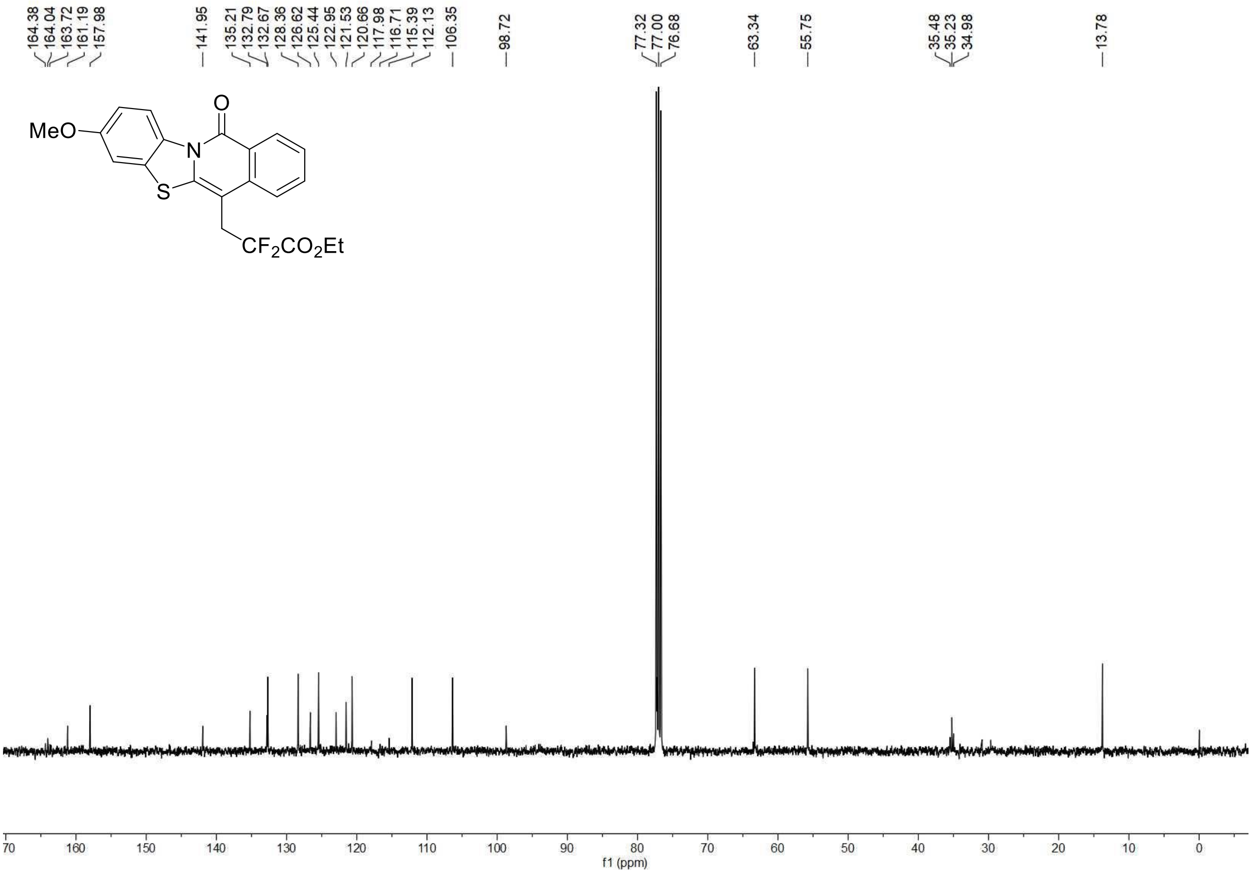

Supplementary Figure 195. ^{13}C-NMR of compound **45**, recorded at 100 MHz and 25 °C in $CDCl_3$.

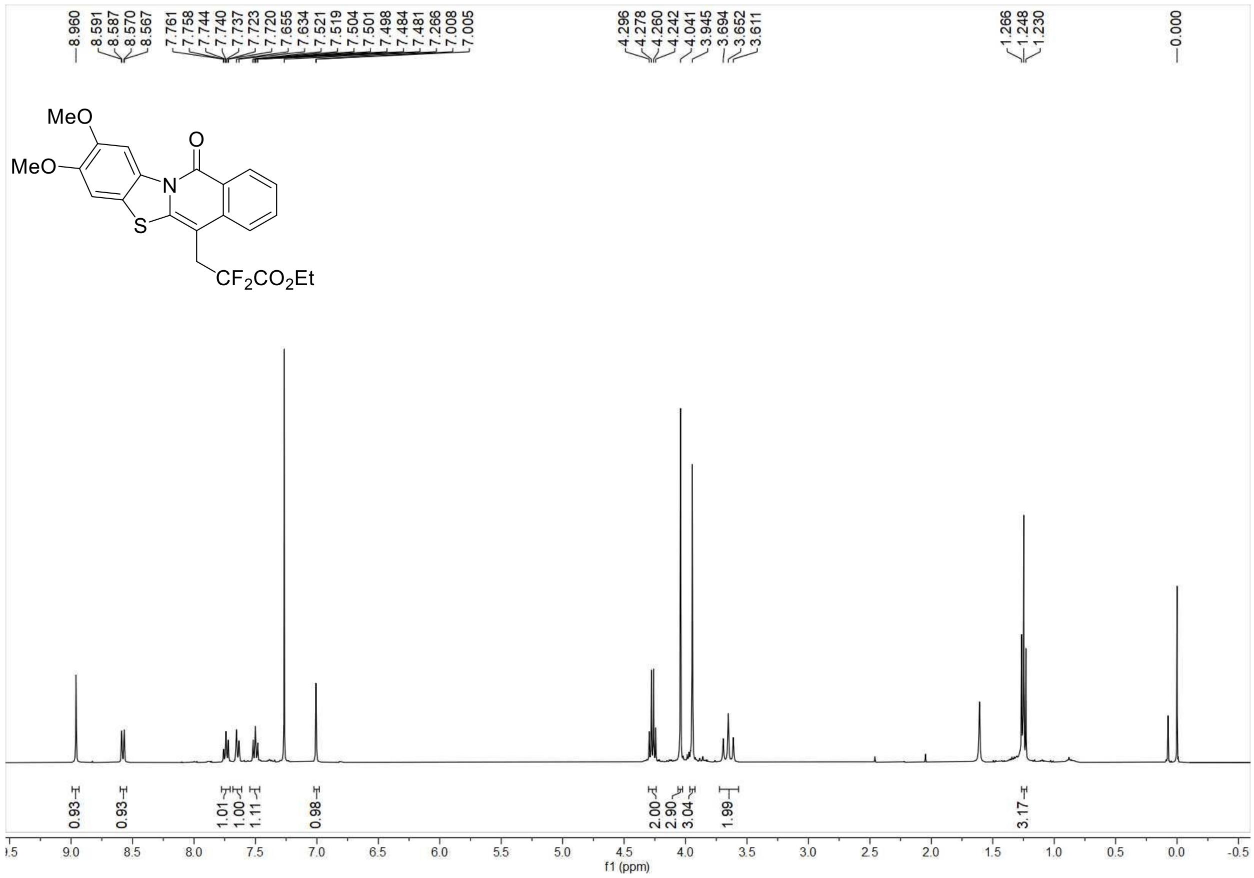

Supplementary Figure 196. ^{1}H-NMR of compound **46**, recorded at 400 MHz and 25 °C in $CDCl_3$.

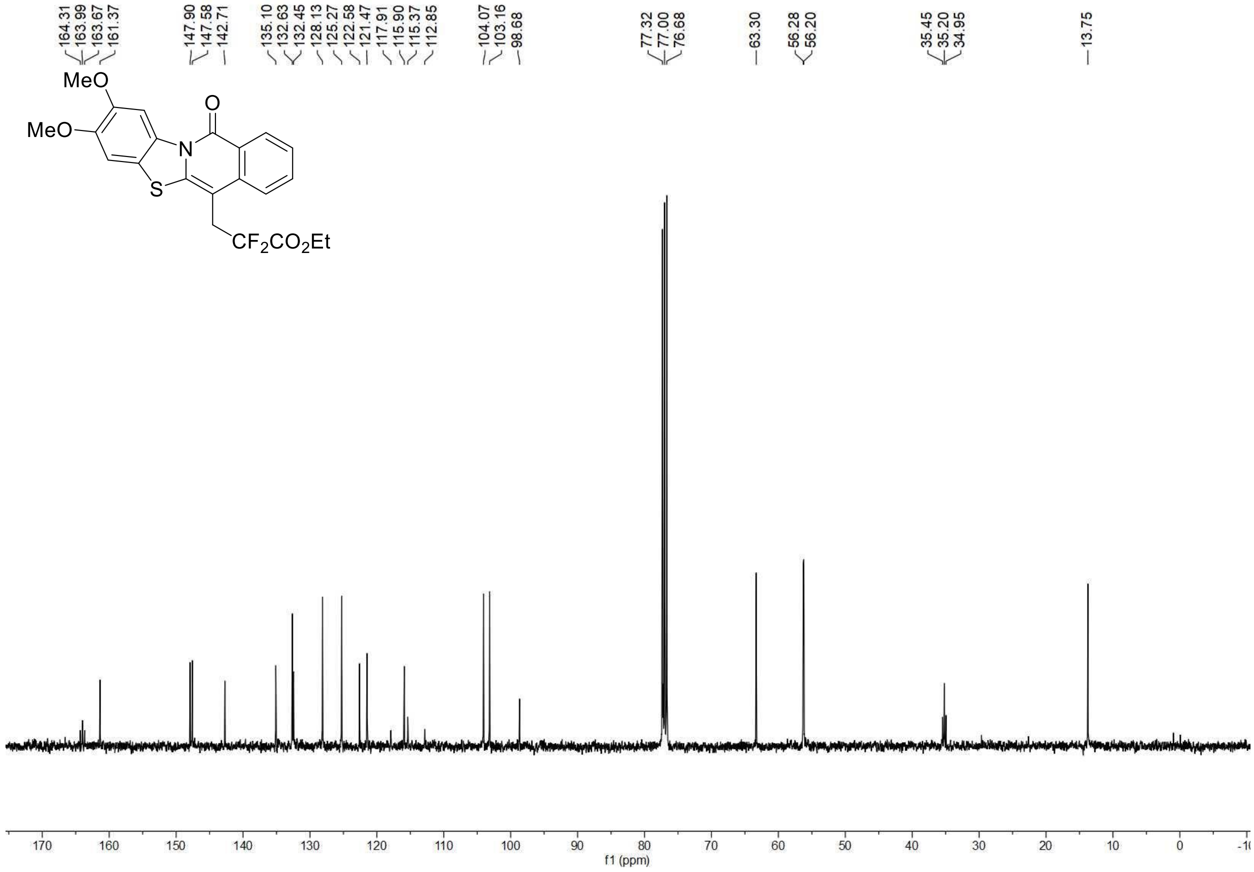

Supplementary Figure 197. ^{13}C-NMR of compound **46**, recorded at 100 MHz and 25 °C in $CDCl_3$.

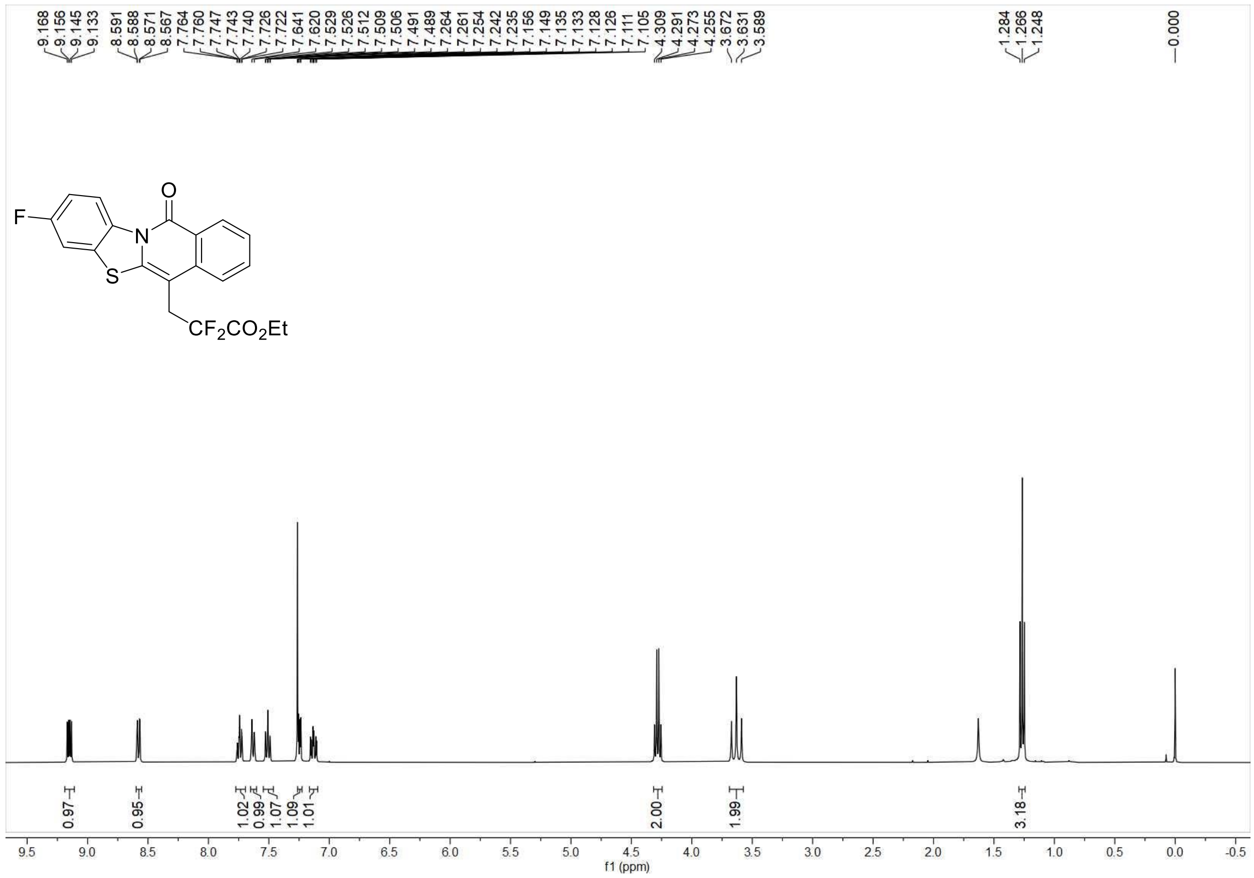

Supplementary Figure 198. ^{1}H-NMR of compound **47**, recorded at 400 MHz and 25 °C in $CDCl_3$.

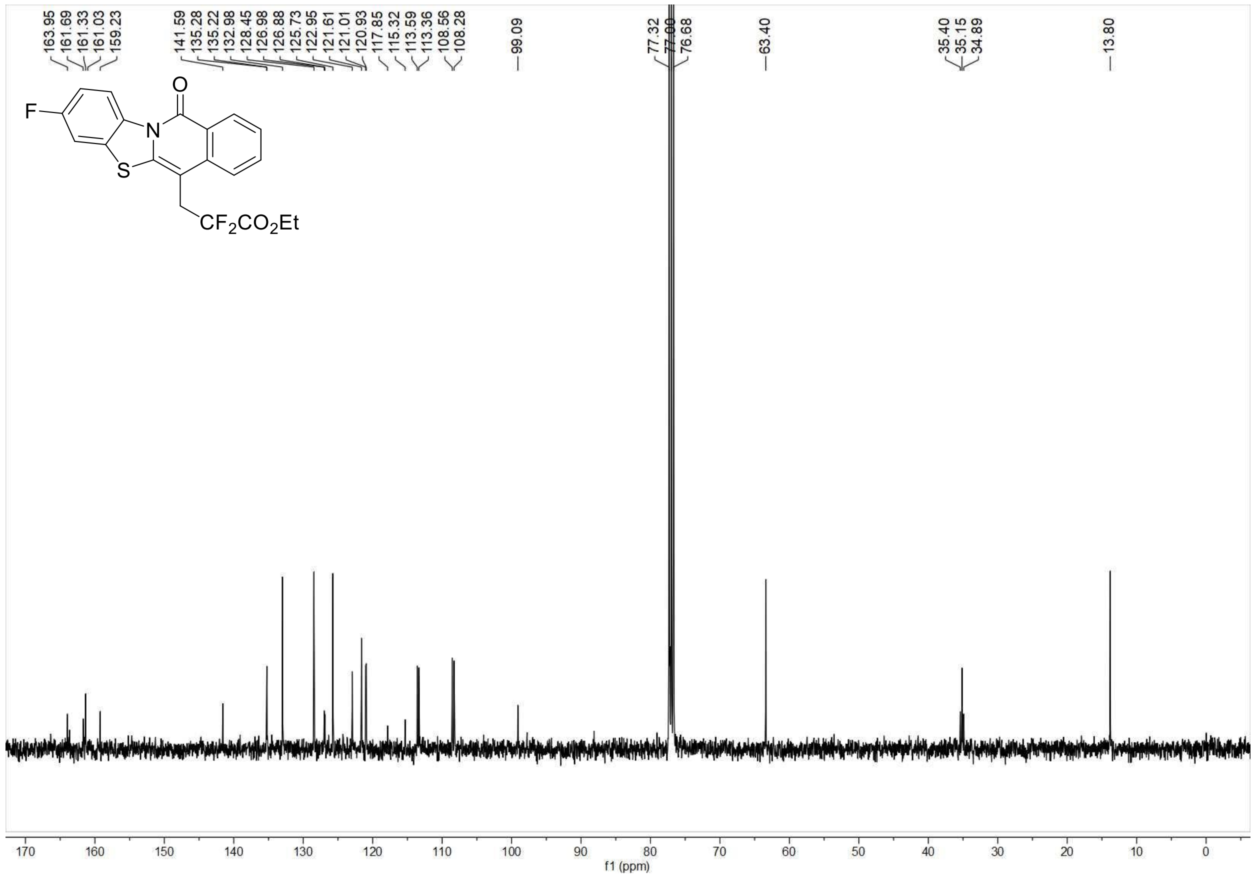

Supplementary Figure 199. ^{13}C-NMR of compound **47**, recorded at 100 MHz and 25 °C in $CDCl_3$.

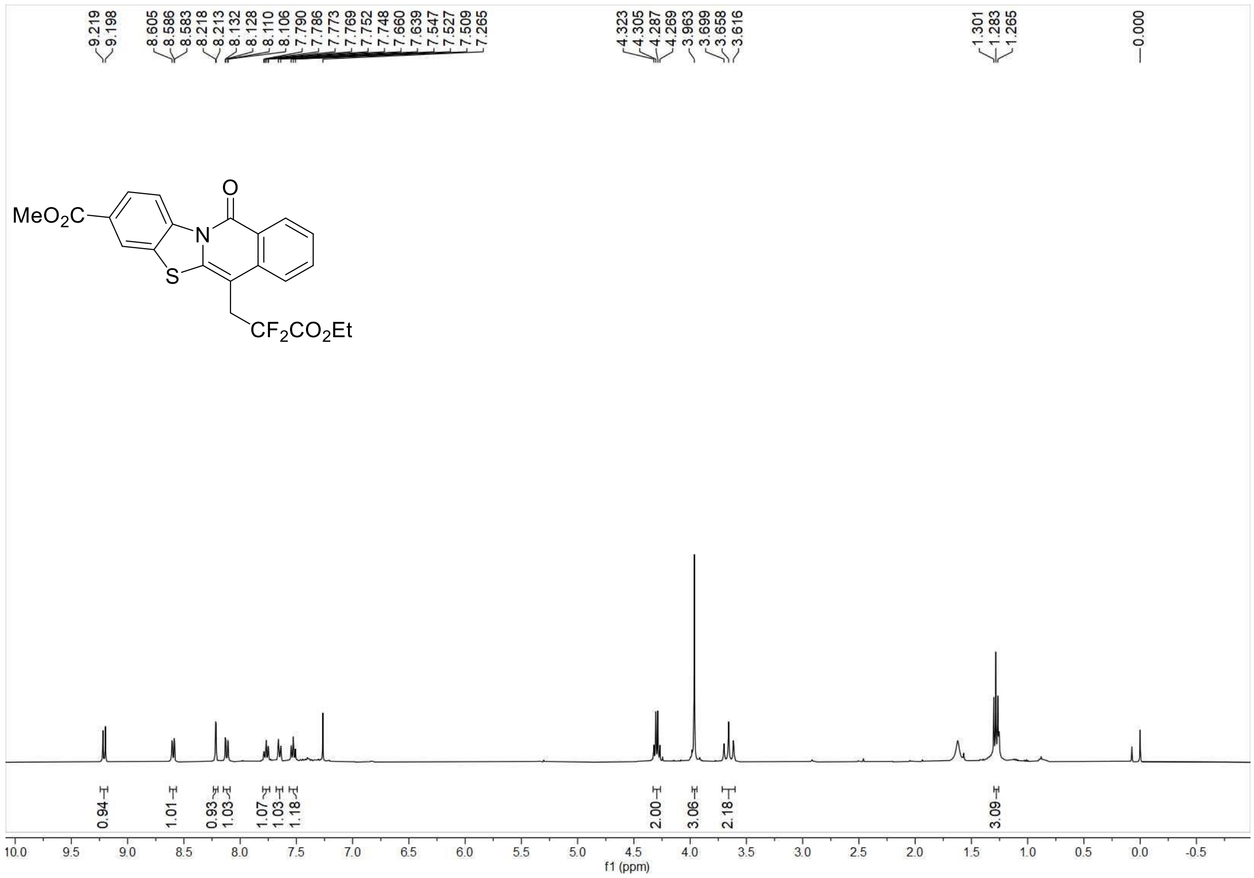

Supplementary Figure 200. ^{1}H-NMR of compound **48**, recorded at 400 MHz and 25 °C in $CDCl_3$.

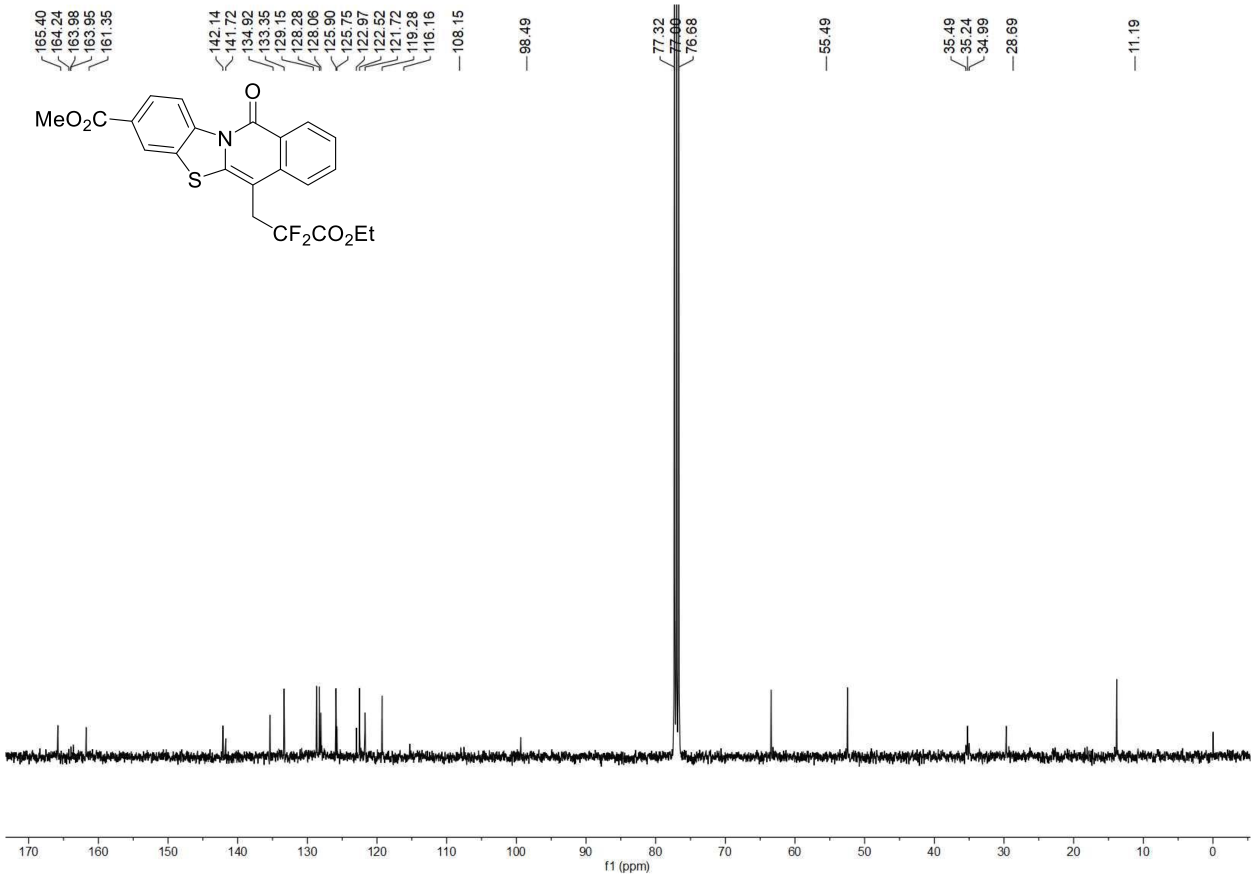

Supplementary Figure 201. ^{13}C-NMR of compound **48**, recorded at 100 MHz and 25 °C in $CDCl_3$.

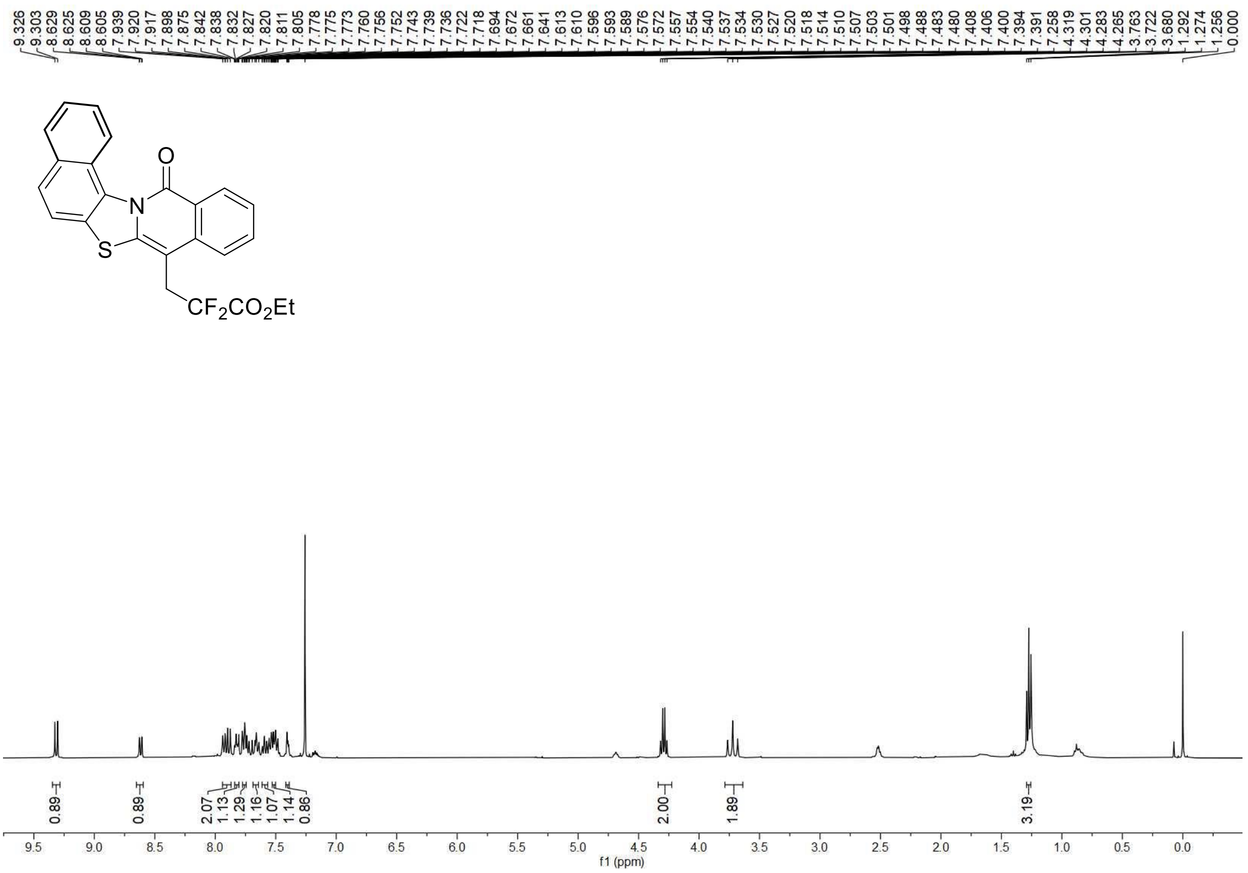

Supplementary Figure 202. ^{1}H-NMR of compound **49**, recorded at 400 MHz and 25 °C in $CDCl_3$.

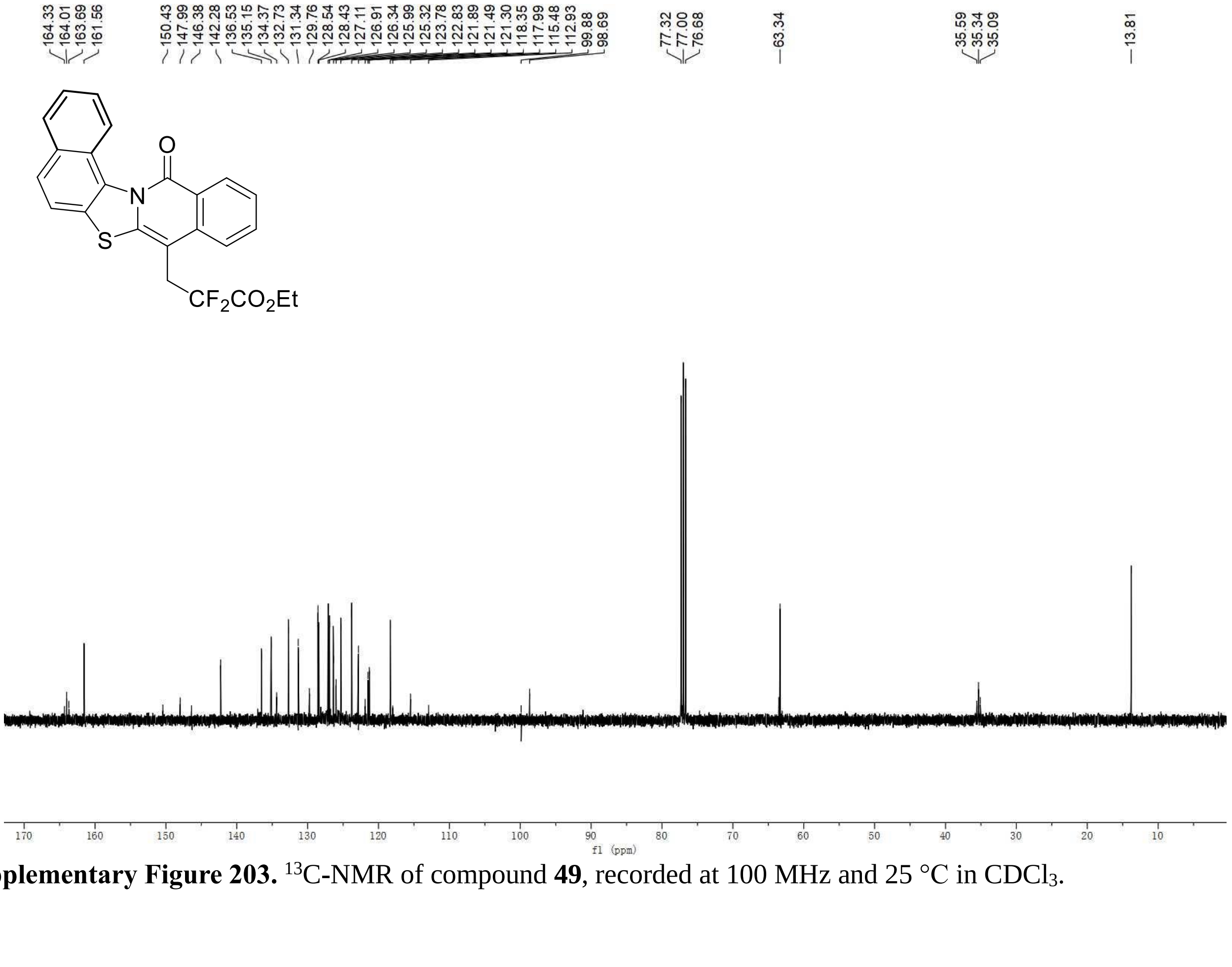

Supplementary Figure 203. ^{13}C-NMR of compound **49**, recorded at 100 MHz and 25 °C in $CDCl_3$.

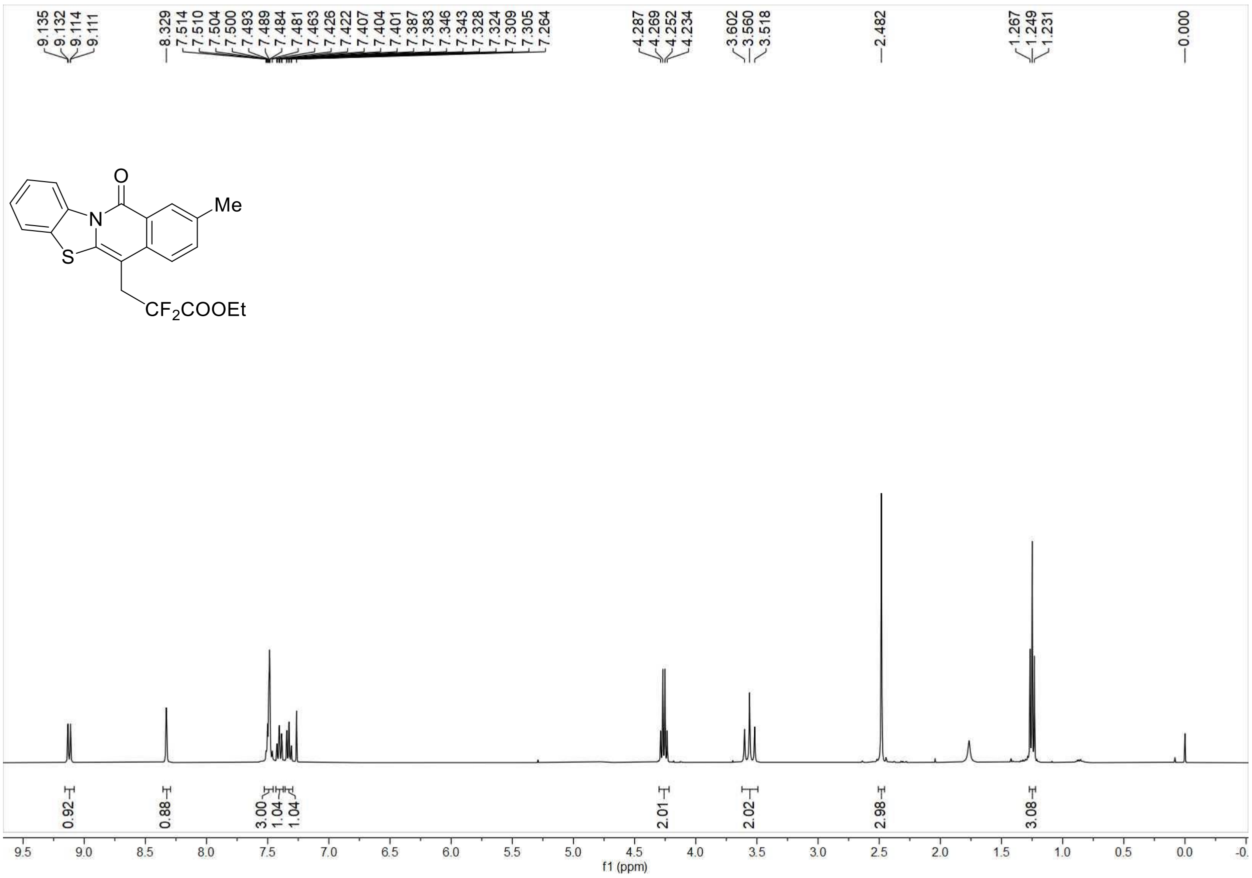

Supplementary Figure 204. ^{1}H-NMR of compound **50**, recorded at 400 MHz and 25 °C in $CDCl_3$.

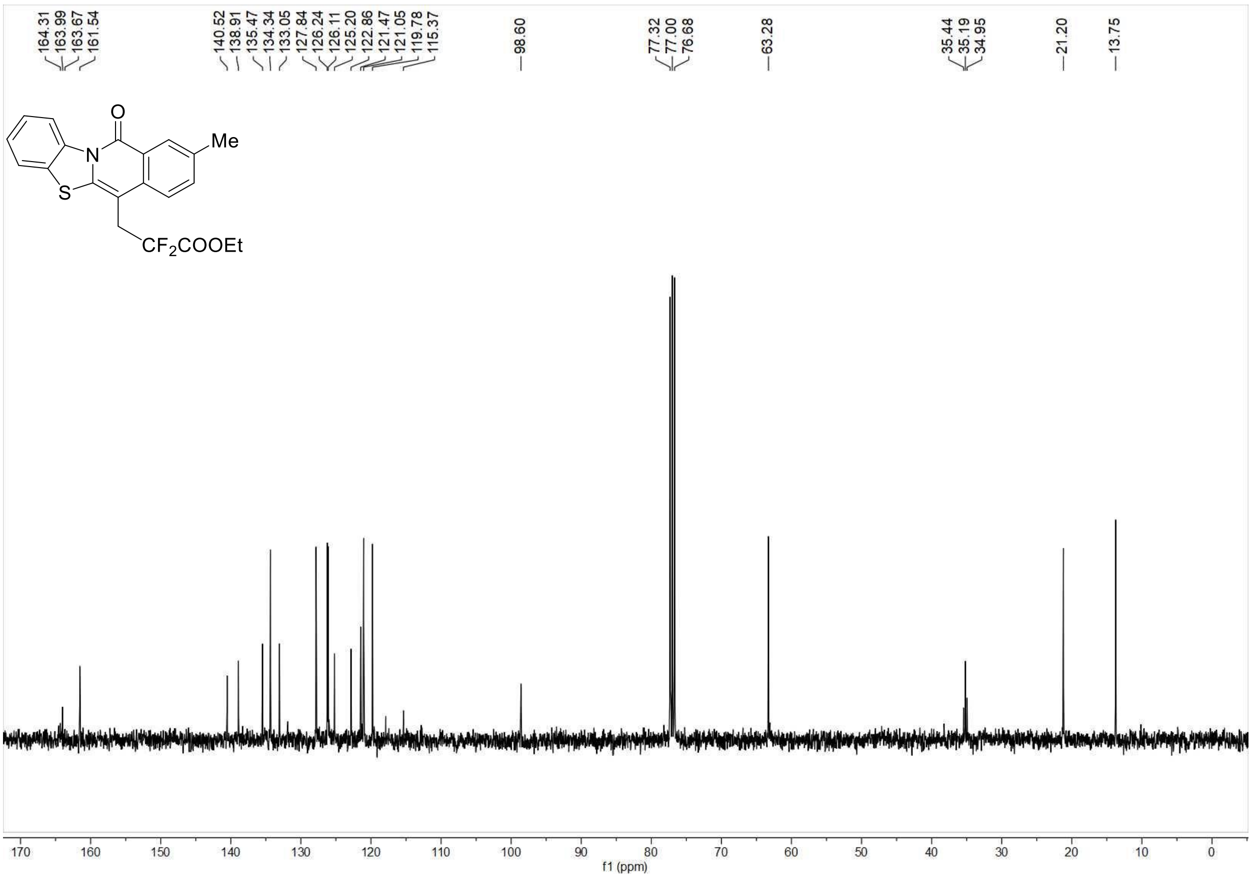

Supplementary Figure 205. ^{13}C-NMR of compound **50**, recorded at 100 MHz and 25 °C in $CDCl_3$.

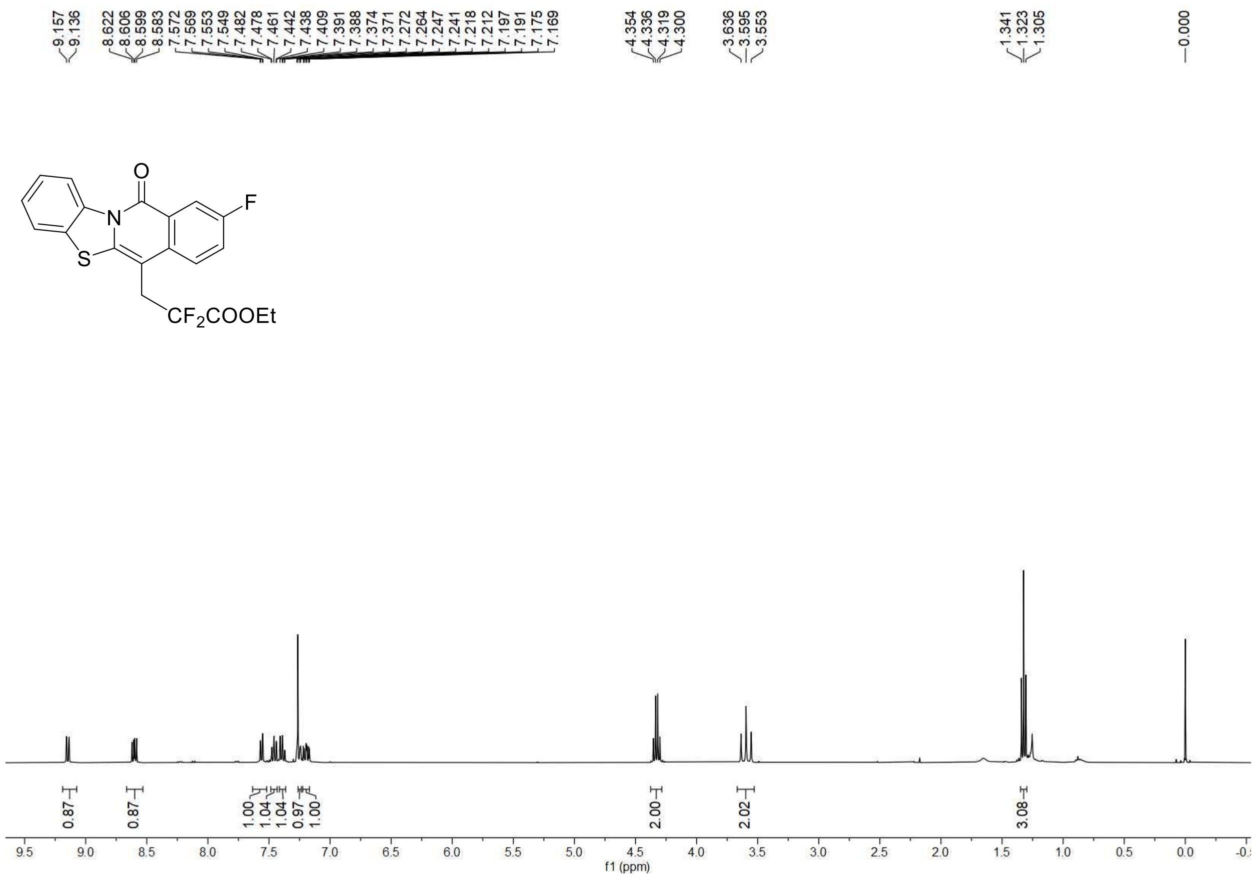

Supplementary Figure 206. ^{1}H-NMR of compound **51**, recorded at 400 MHz and 25 °C in $CDCl_3$.

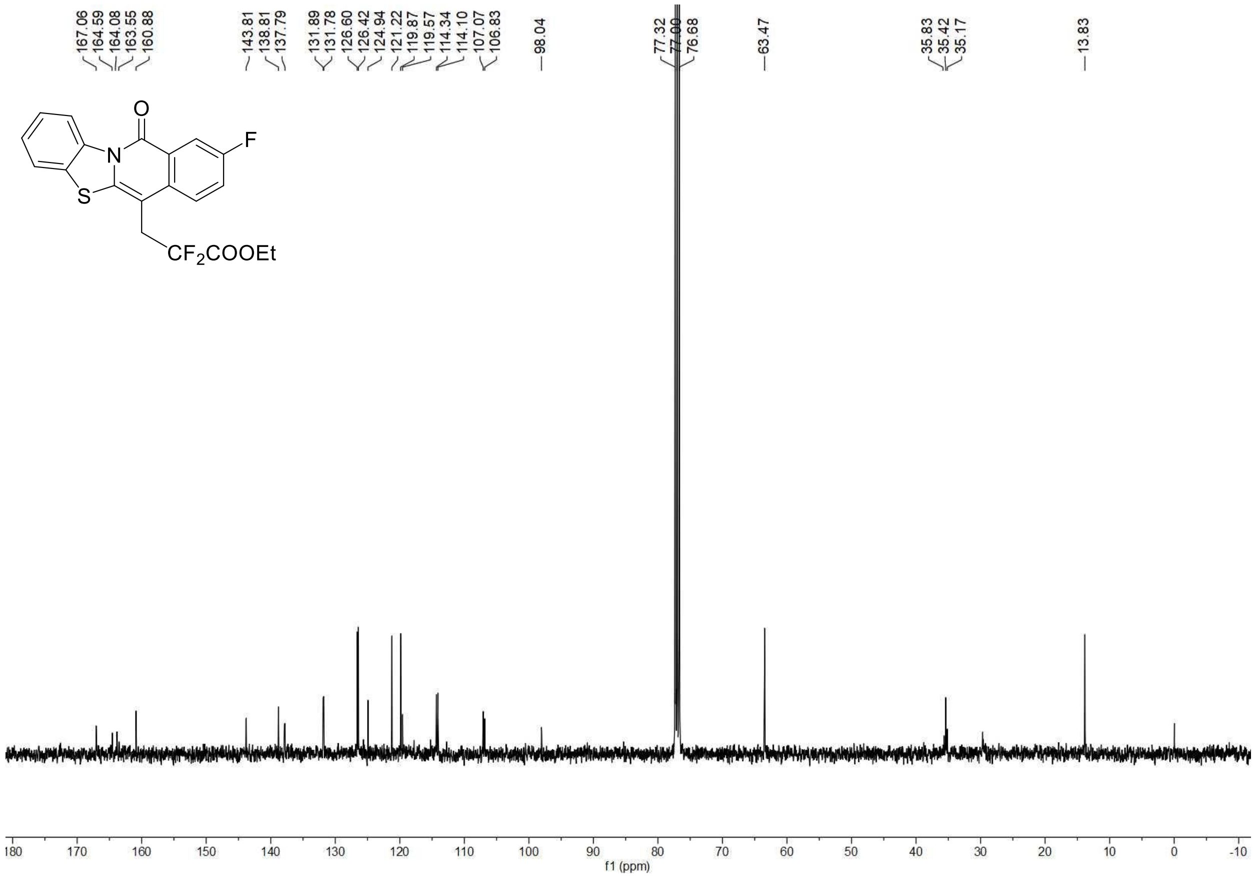

Supplementary Figure 207. ^{13}C-NMR of compound **51**, recorded at 100 MHz and 25 °C in $CDCl_3$.

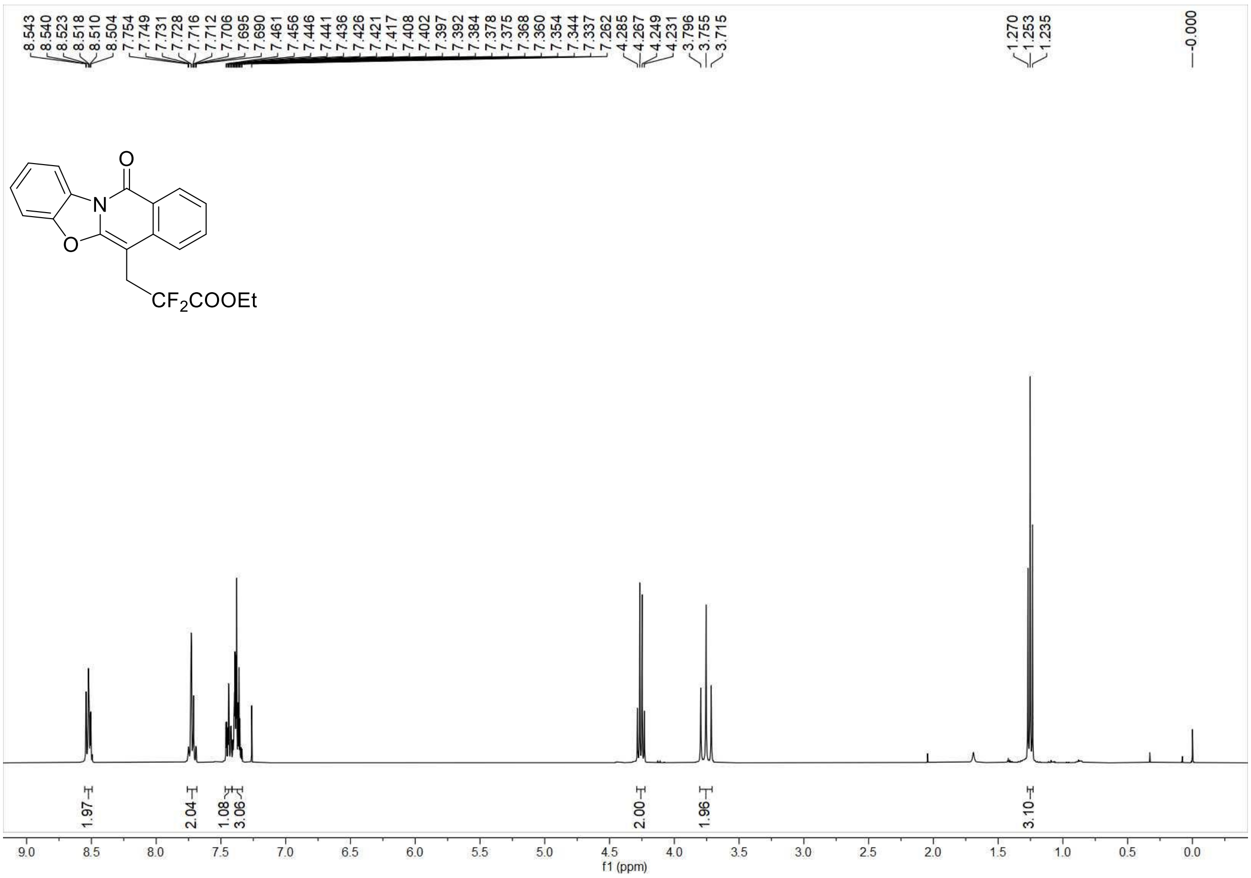

Supplementary Figure 208. ^{1}H-NMR of compound **52**, recorded at 400 MHz and 25 °C in $CDCl_3$.

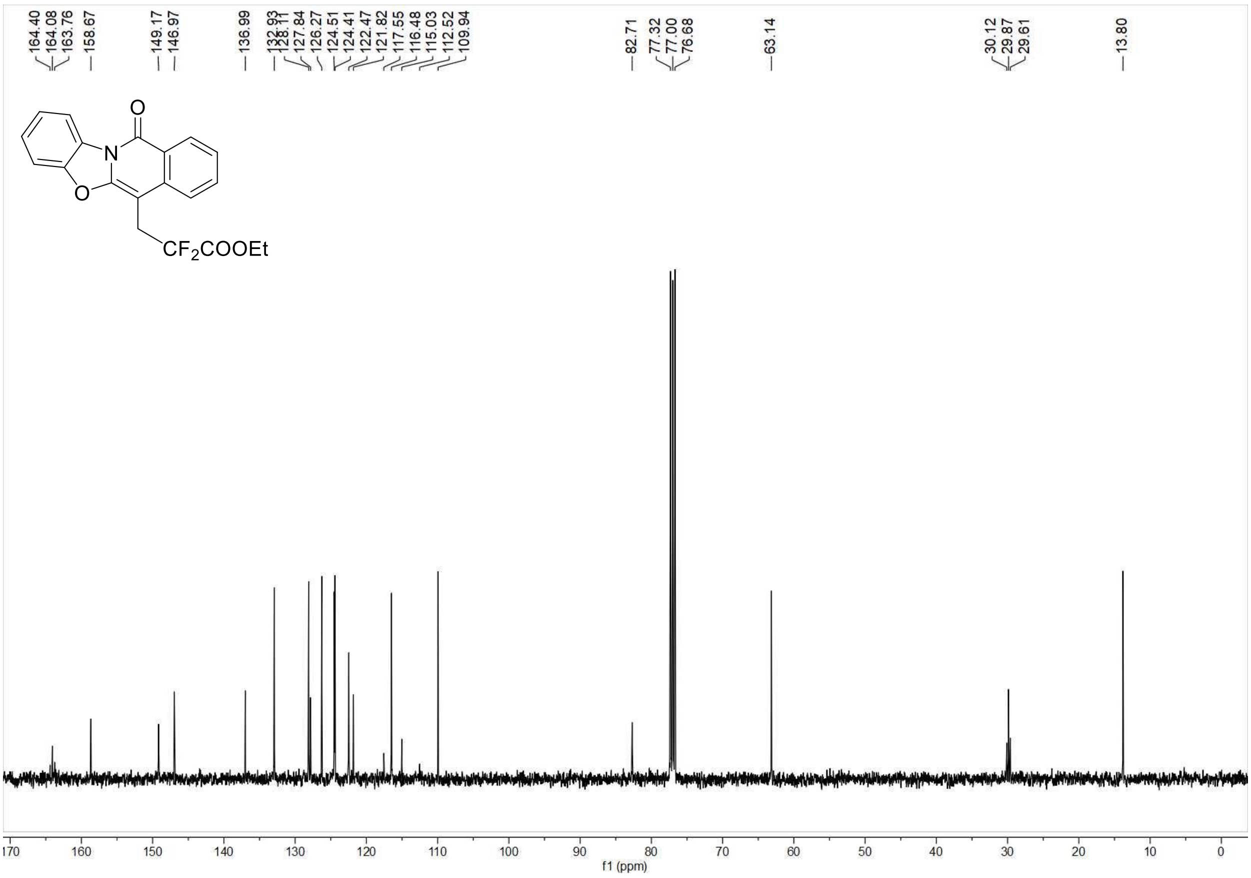

Supplementary Figure 209. ^{13}C-NMR of compound **52**, recorded at 100 MHz and 25 °C in $CDCl_3$.

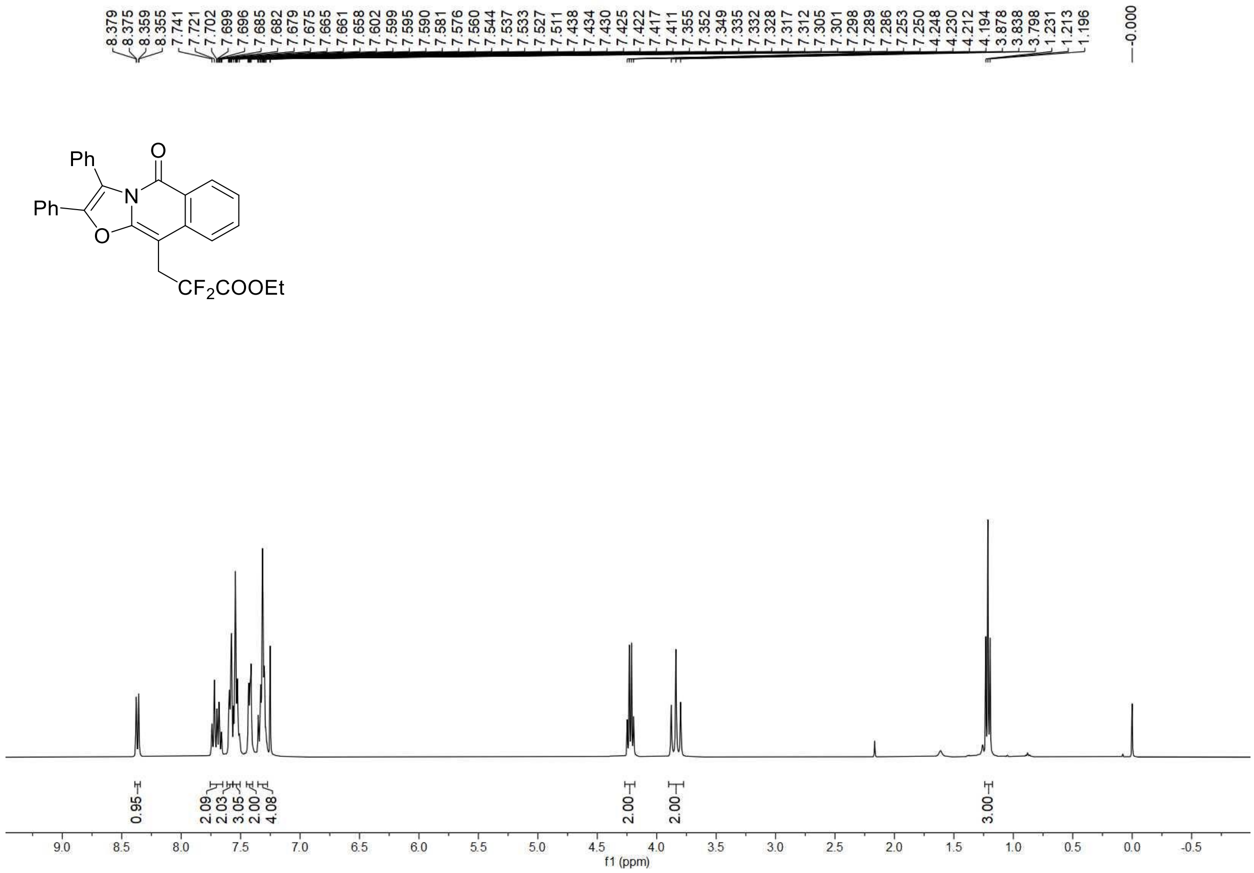

Supplementary Figure 210. ^{1}H-NMR of compound **53**, recorded at 400 MHz and 25 °C in $CDCl_3$.

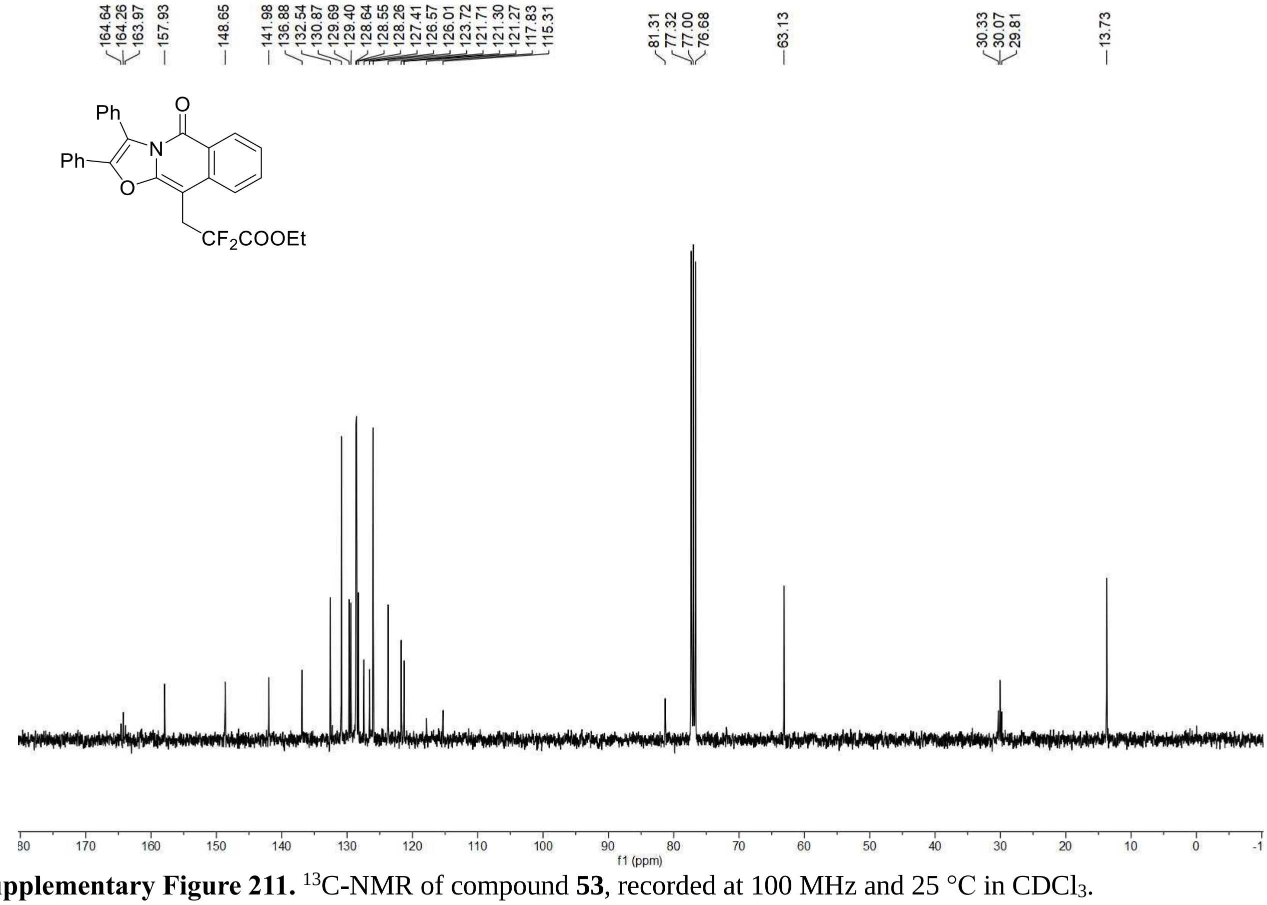

Supplementary Figure 211. ^{13}C-NMR of compound **53**, recorded at 100 MHz and 25 °C in $CDCl_3$.

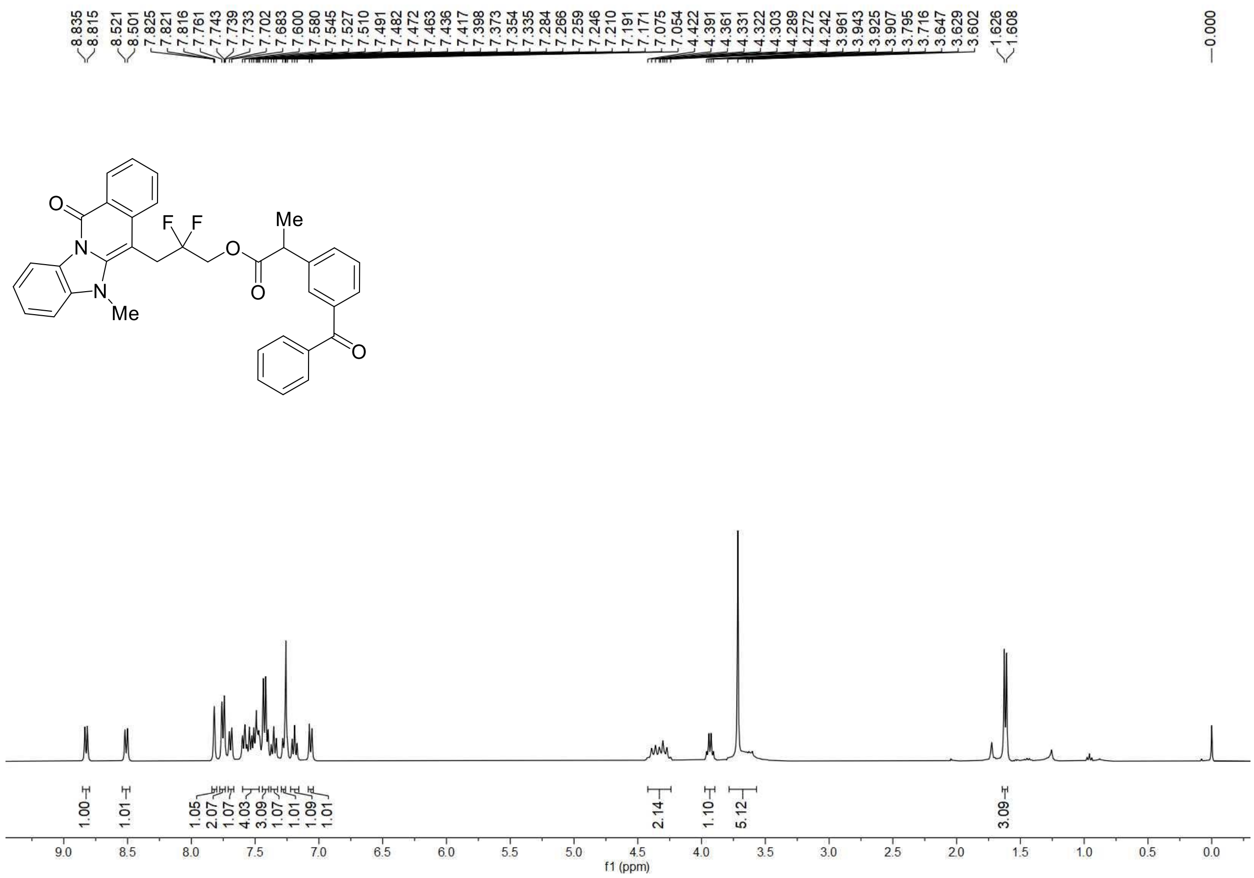

Supplementary Figure 212. ^{1}H-NMR of compound **60**, recorded at 400 MHz and 25 °C in $CDCl_3$.

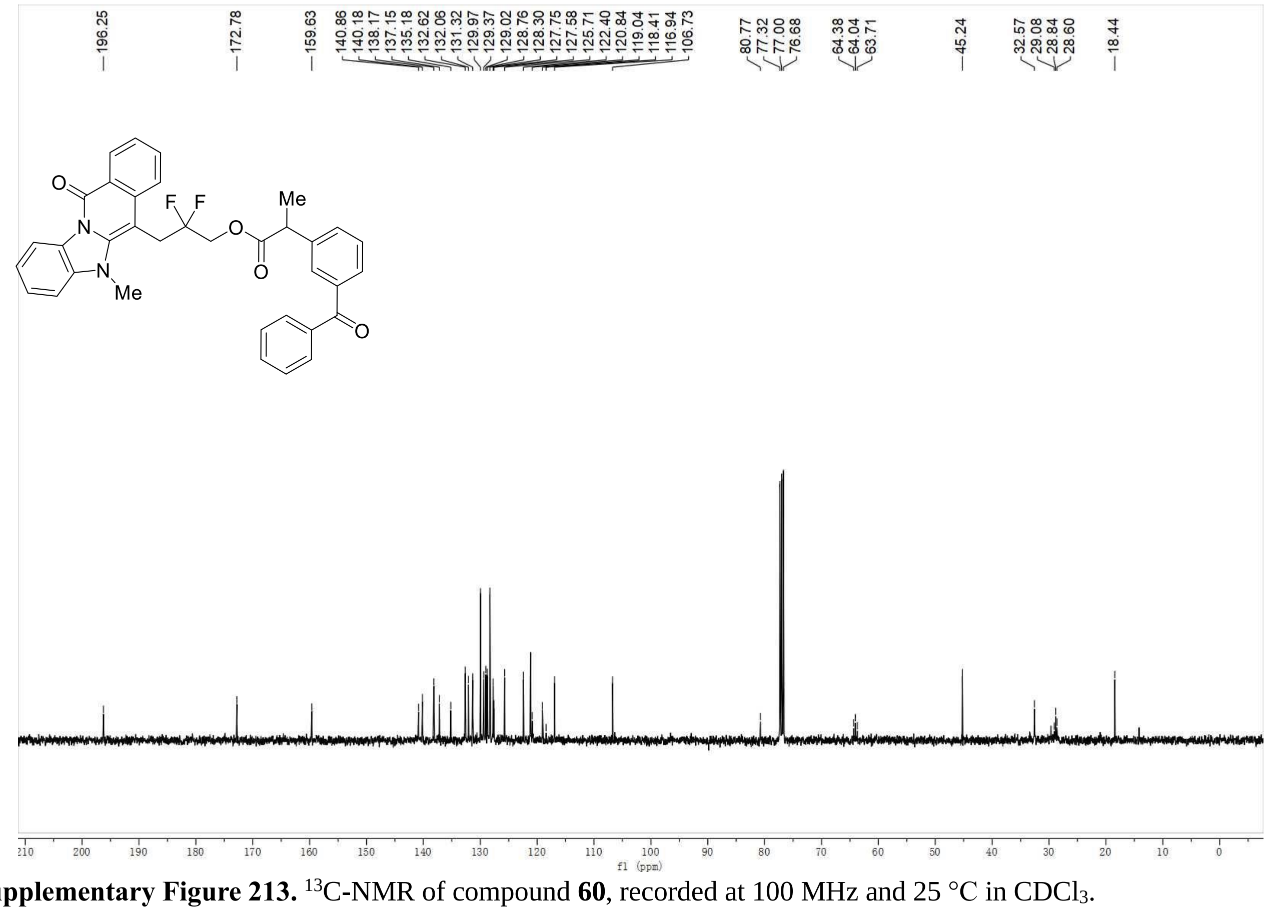

Supplementary Figure 213. ^{13}C-NMR of compound **60**, recorded at 100 MHz and 25 °C in $CDCl_3$.

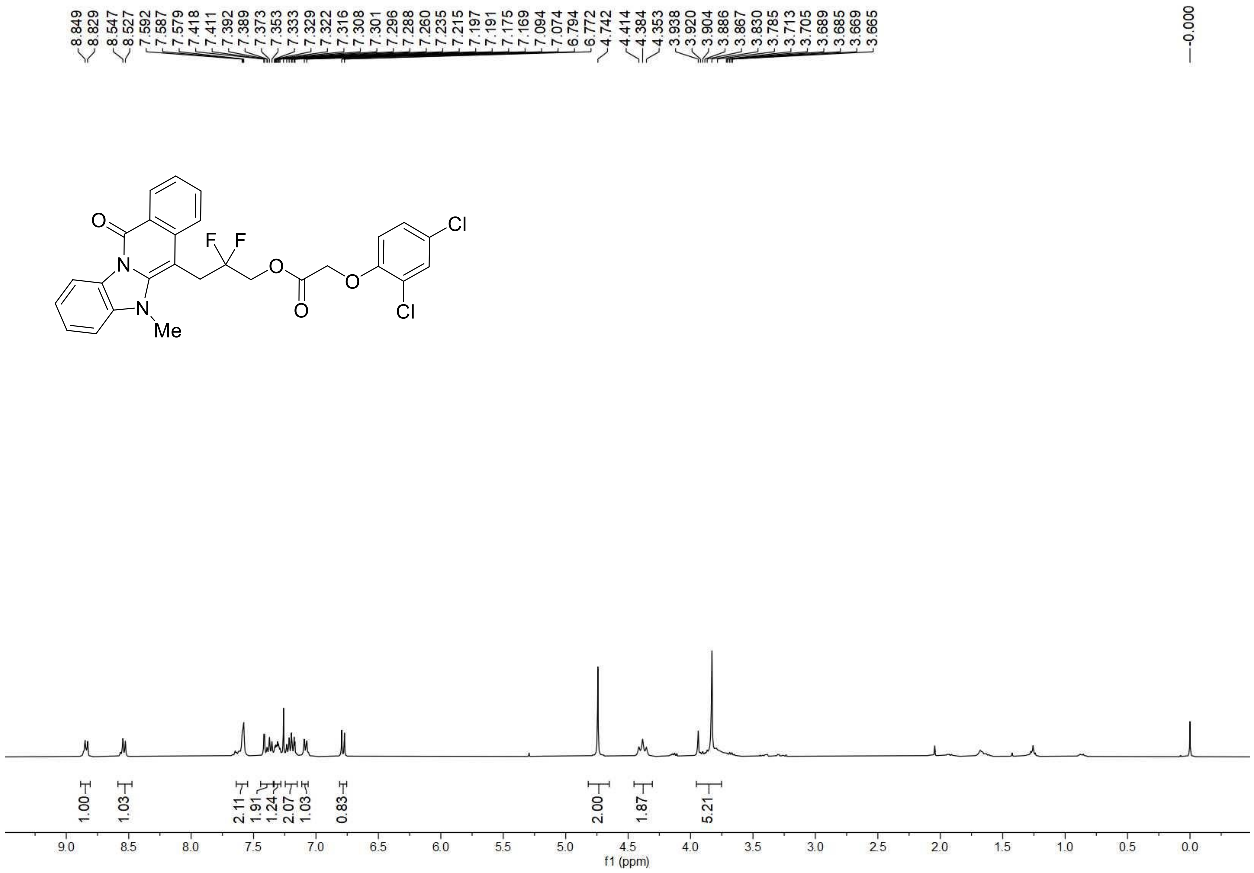

Supplementary Figure 214. ^{1}H-NMR of compound **61**, recorded at 400 MHz and 25 °C in $CDCl_3$.

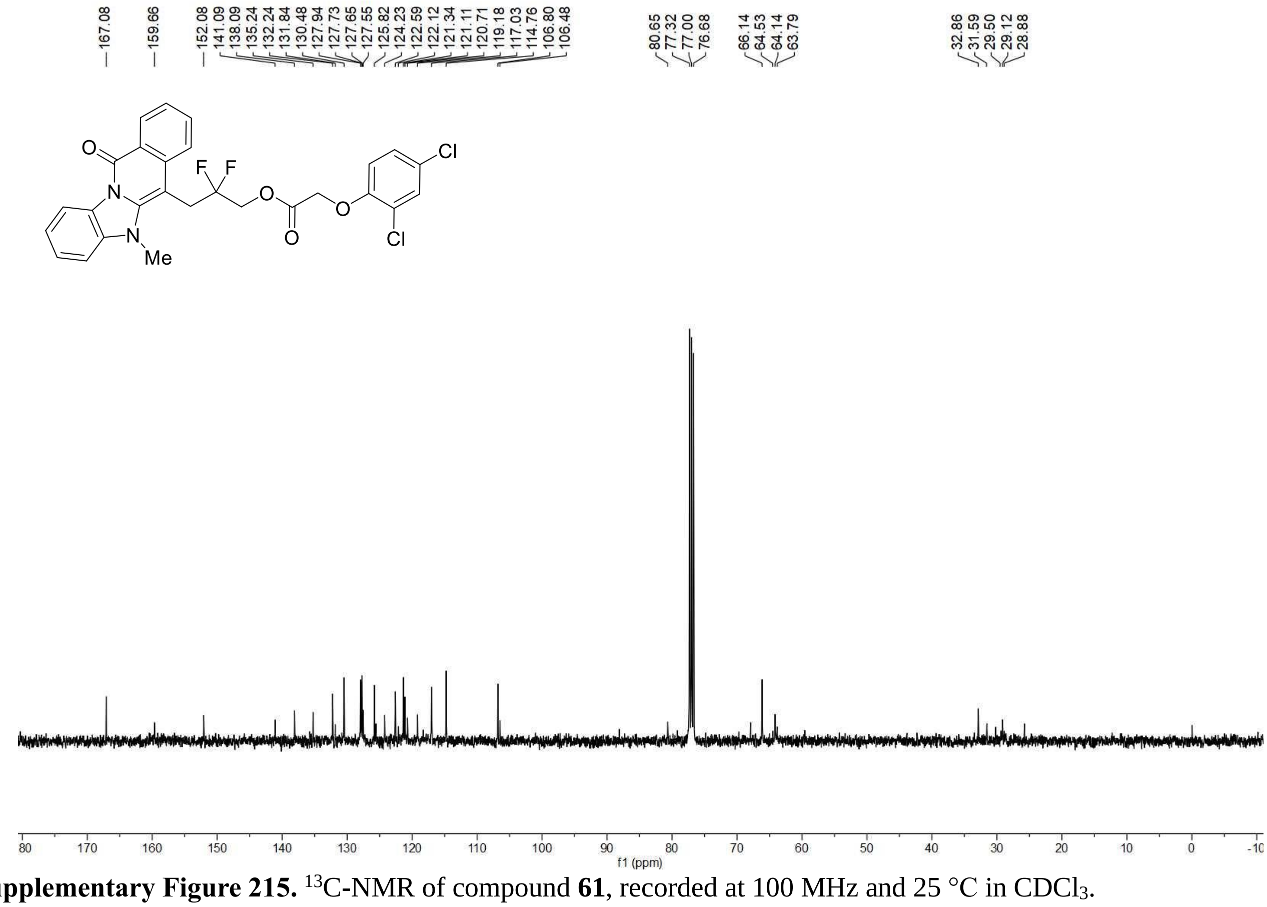

Supplementary Figure 215. ^{13}C-NMR of compound **61**, recorded at 100 MHz and 25 °C in $CDCl_3$.

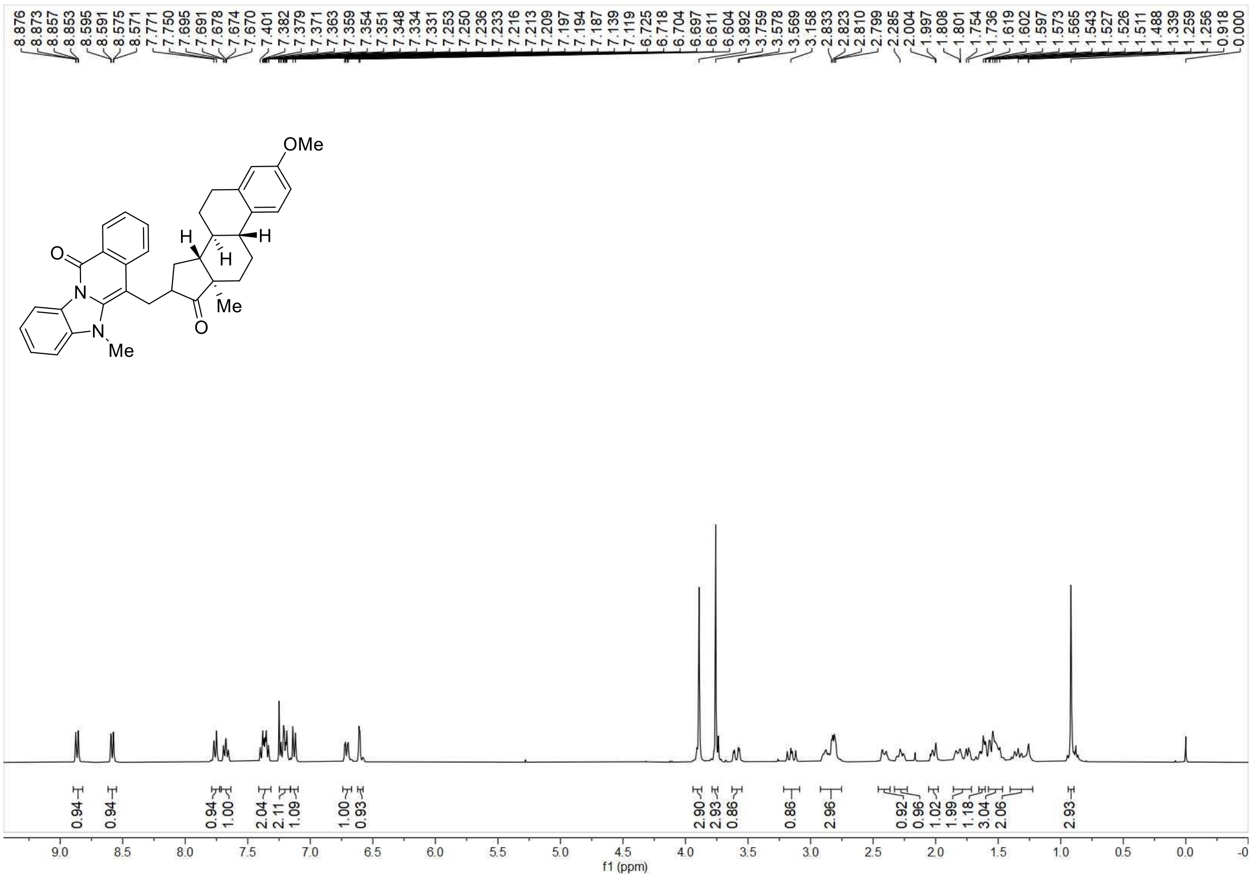

Supplementary Figure 216. ^{1}H-NMR of compound **62**, recorded at 400 MHz and 25 °C in $CDCl_3$.

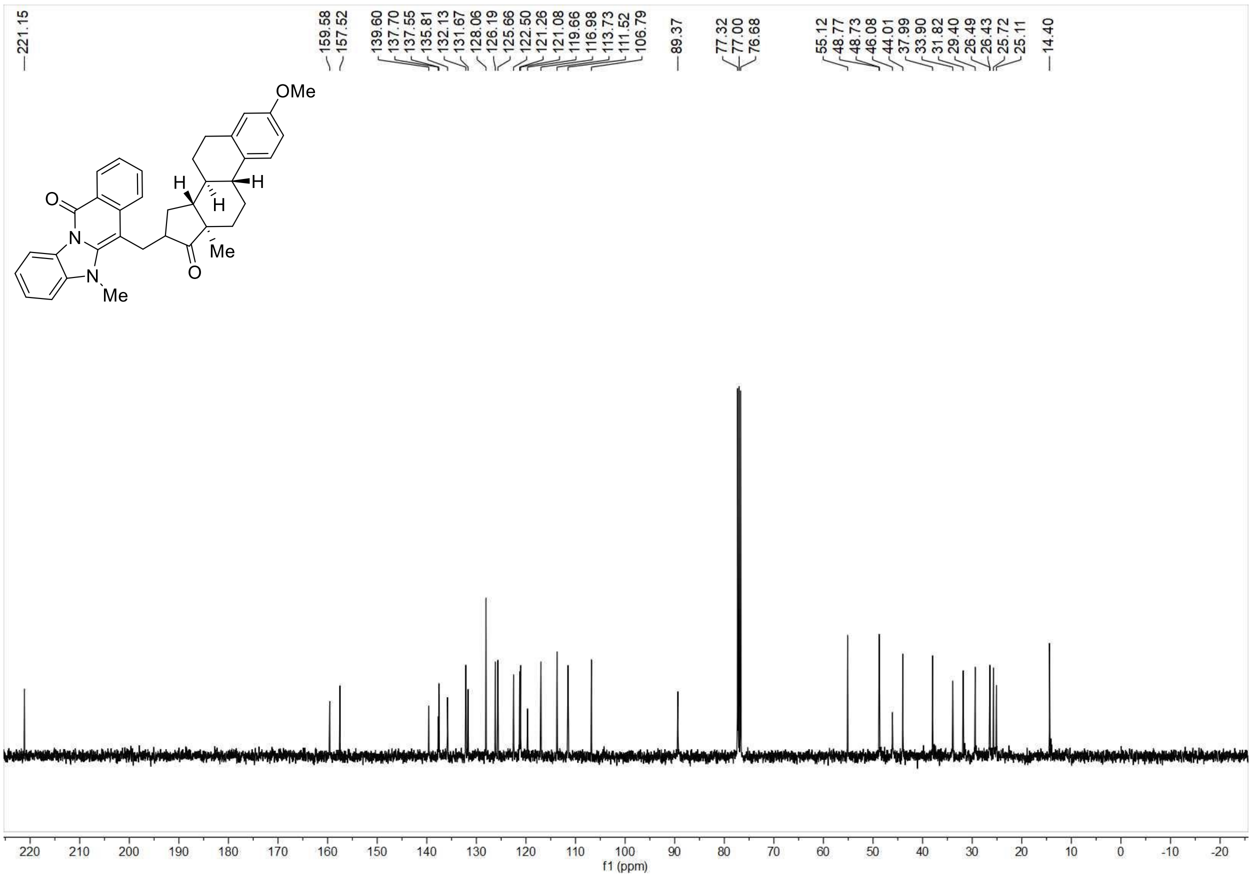

Supplementary Figure 217. ^{13}C-NMR of compound **62**, recorded at 100 MHz and 25 °C in $CDCl_3$.

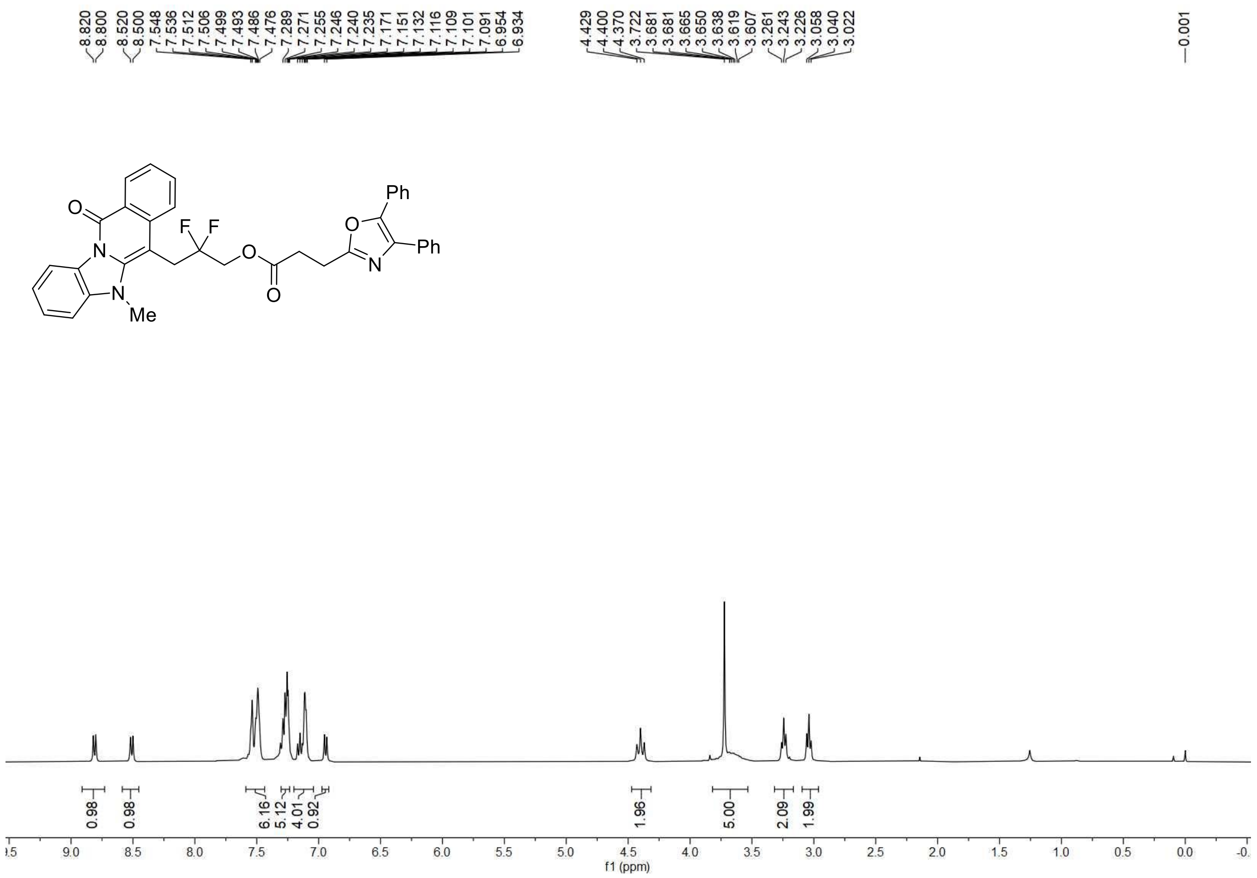

Supplementary Figure 218. ^{1}H-NMR of compound **63**, recorded at 400 MHz and 25 °C in $CDCl_3$.

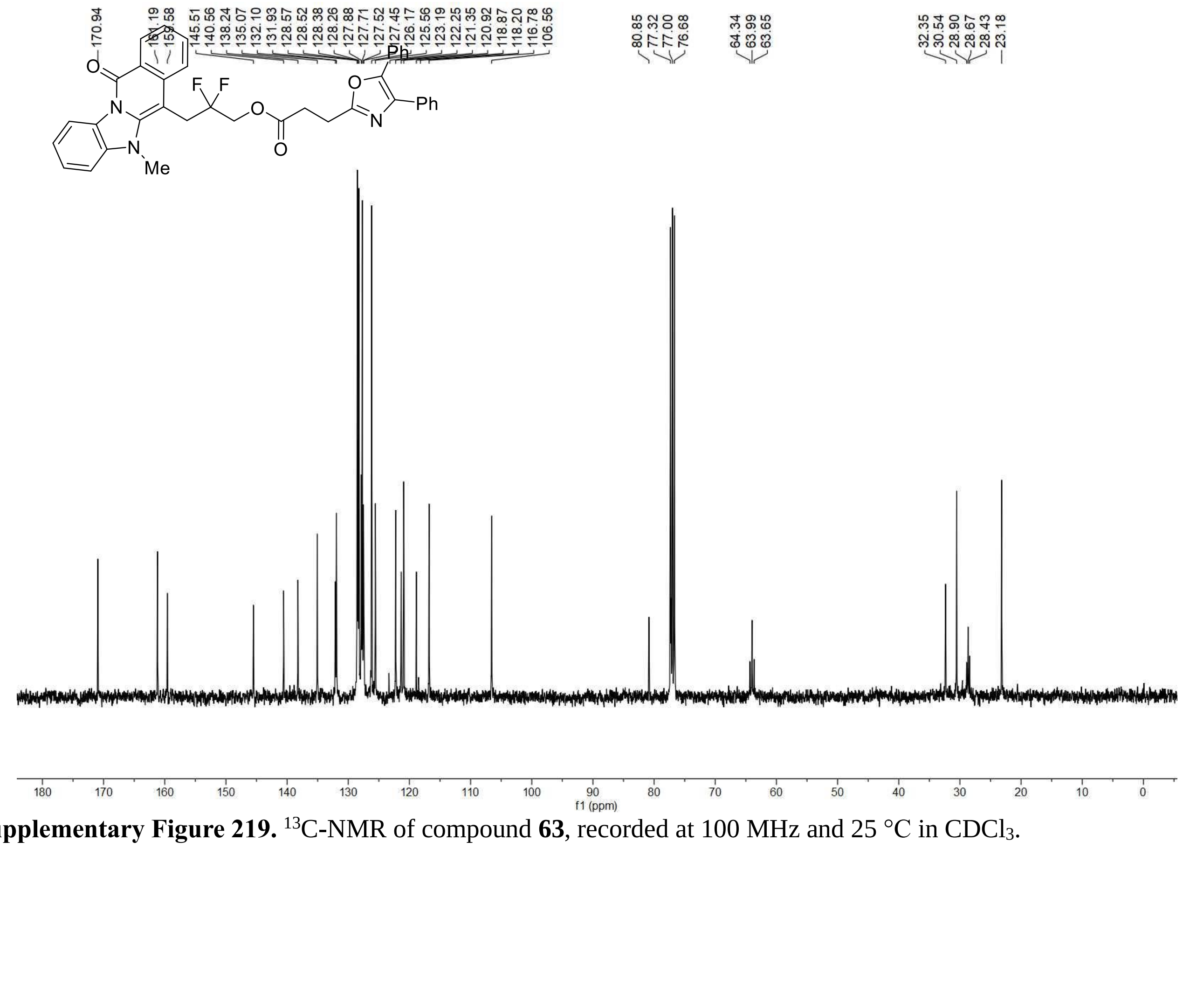

Supplementary Figure 219. ^{13}C-NMR of compound **63**, recorded at 100 MHz and 25 °C in $CDCl_3$.

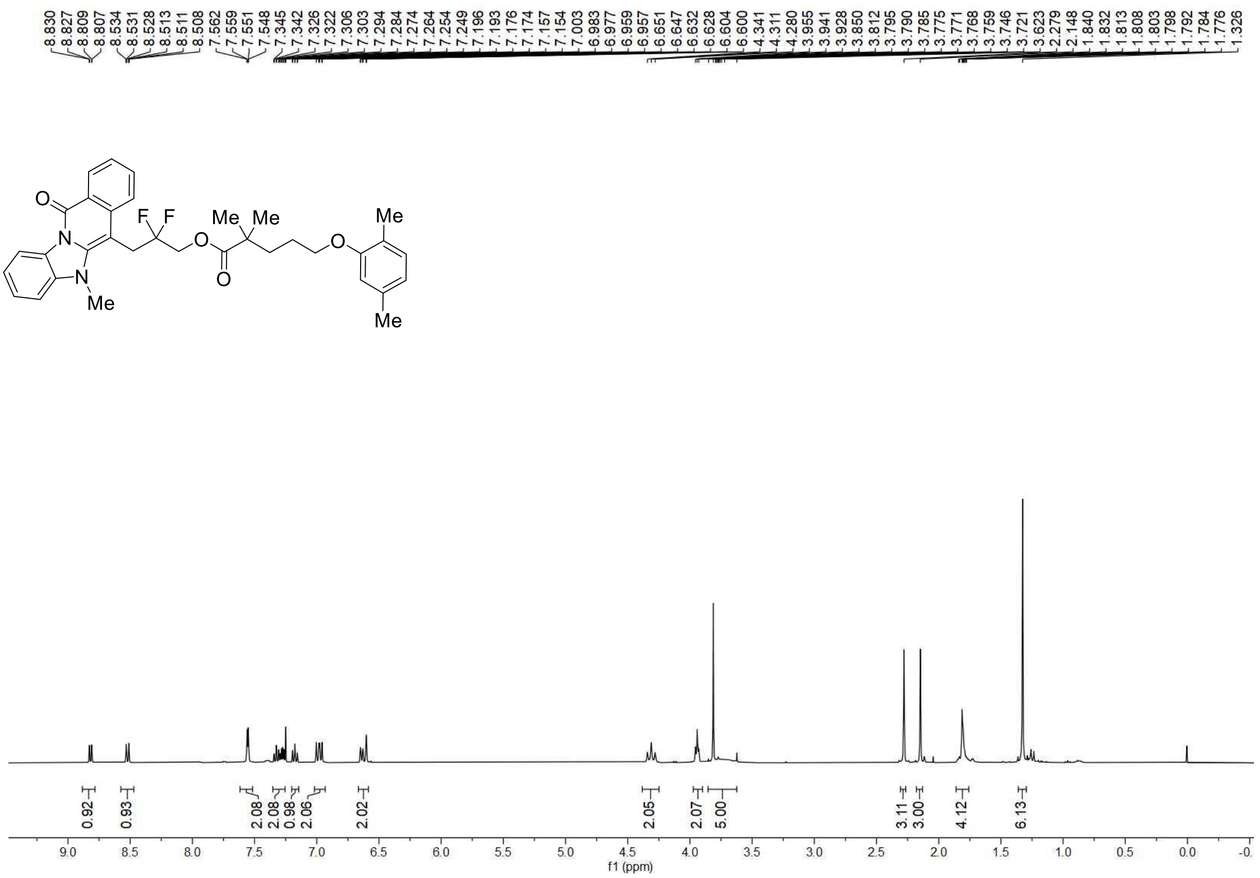

Supplementary Figure 220. ^{1}H-NMR of compound **64**, recorded at 400 MHz and 25 °C in $CDCl_3$.

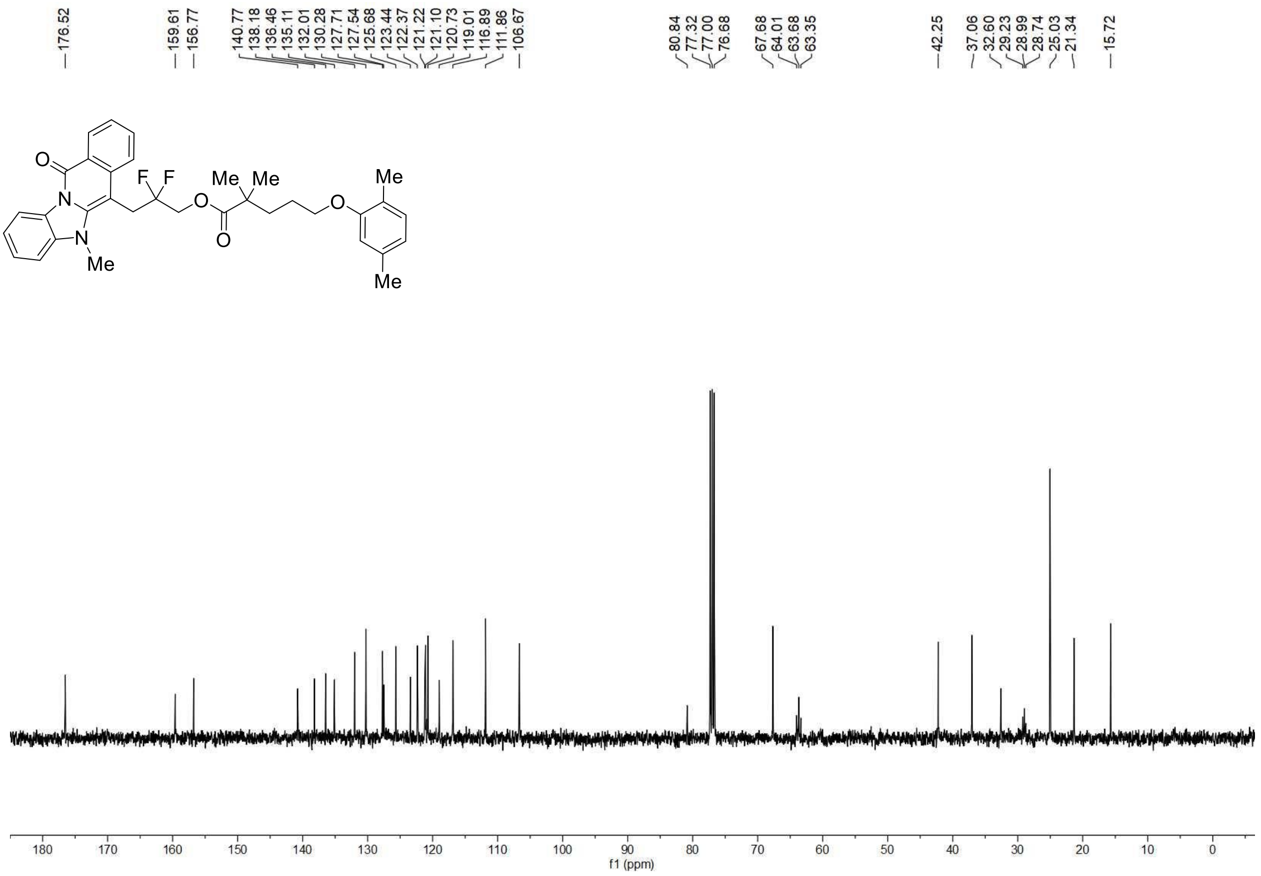

Supplementary Figure 221. ^{13}C-NMR of compound **64**, recorded at 100 MHz and 25 °C in $CDCl_3$.

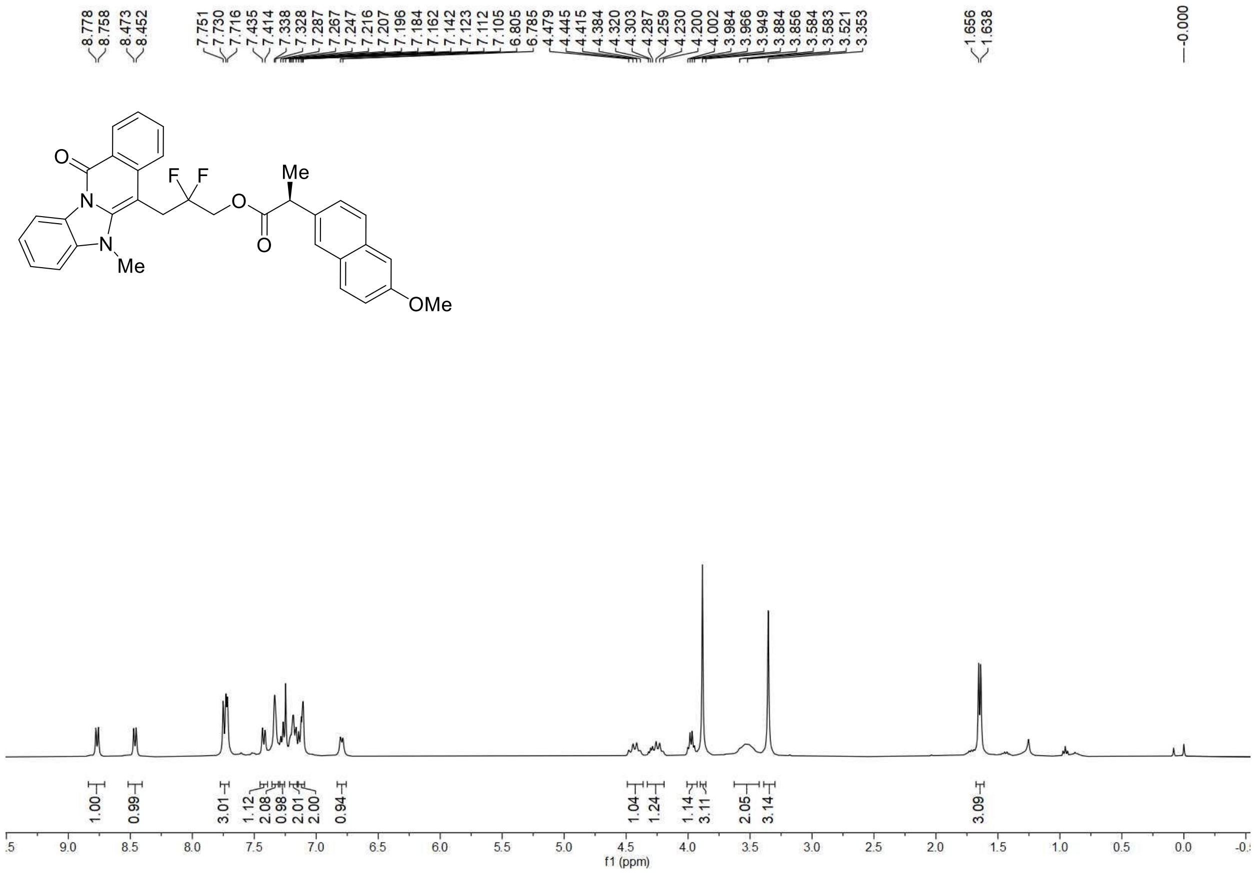

Supplementary Figure 222. ^{1}H-NMR of compound **65**, recorded at 400 MHz and 25 °C in $CDCl_3$.

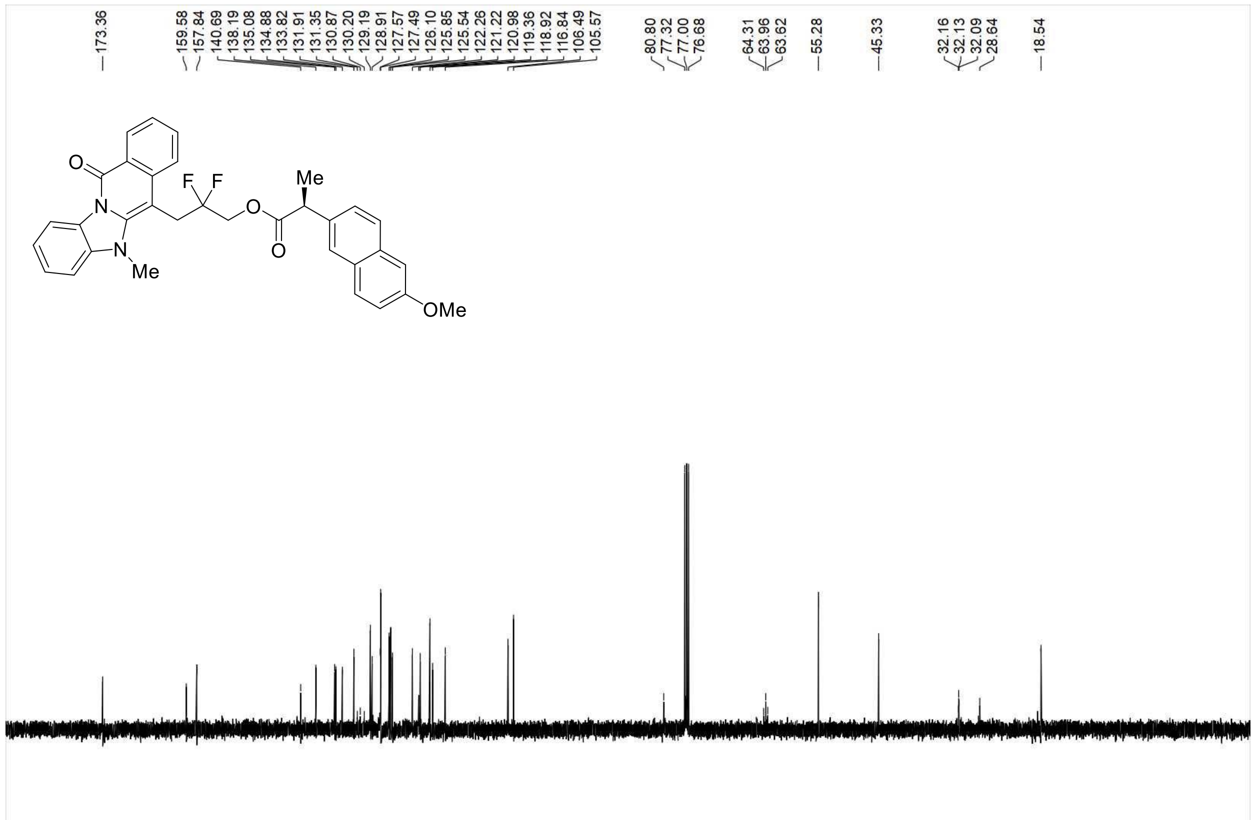

Supplementary Figure 223. ^{13}C-NMR of compound **65**, recorded at 100 MHz and 25 °C in $CDCl_3$.

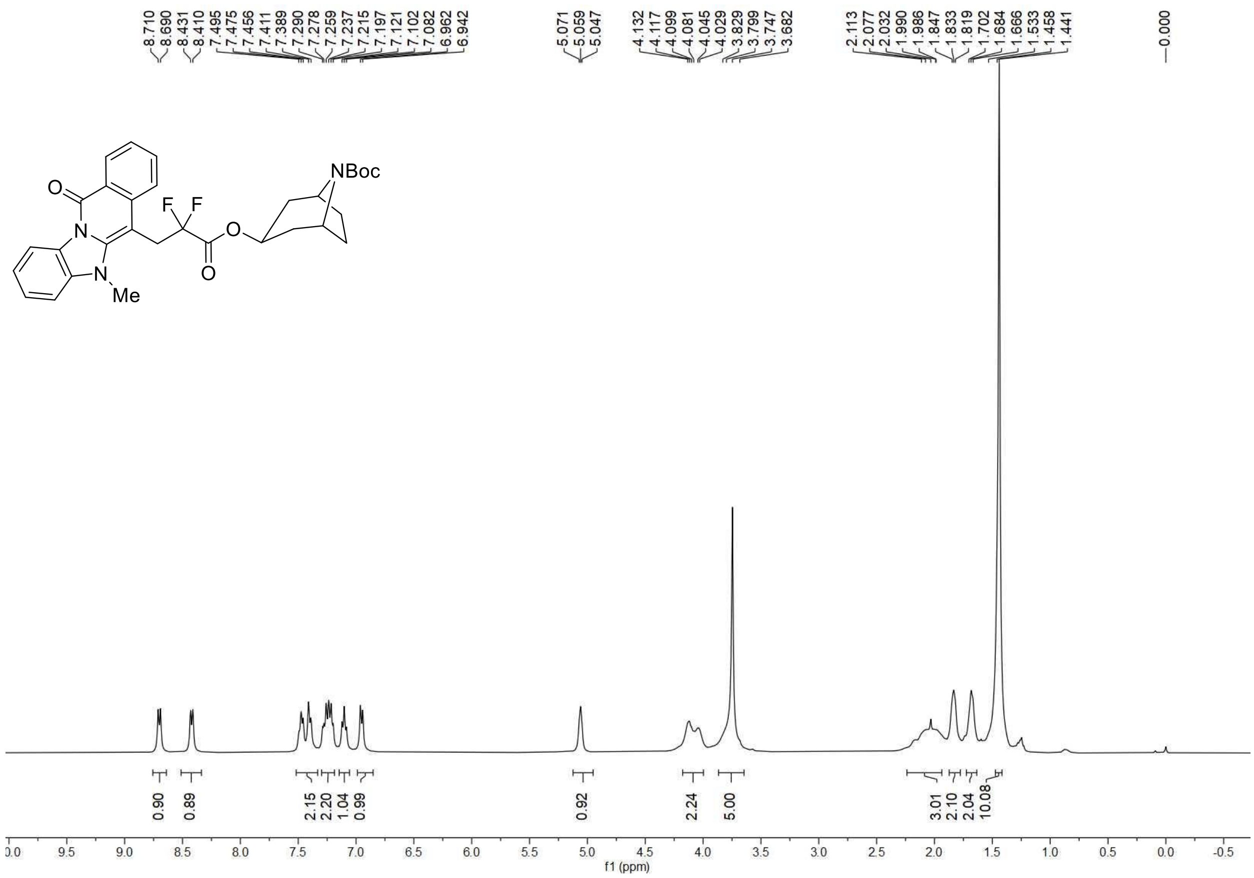

Supplementary Figure 224. ^{1}H-NMR of compound **66**, recorded at 400 MHz and 25 °C in $CDCl_3$.

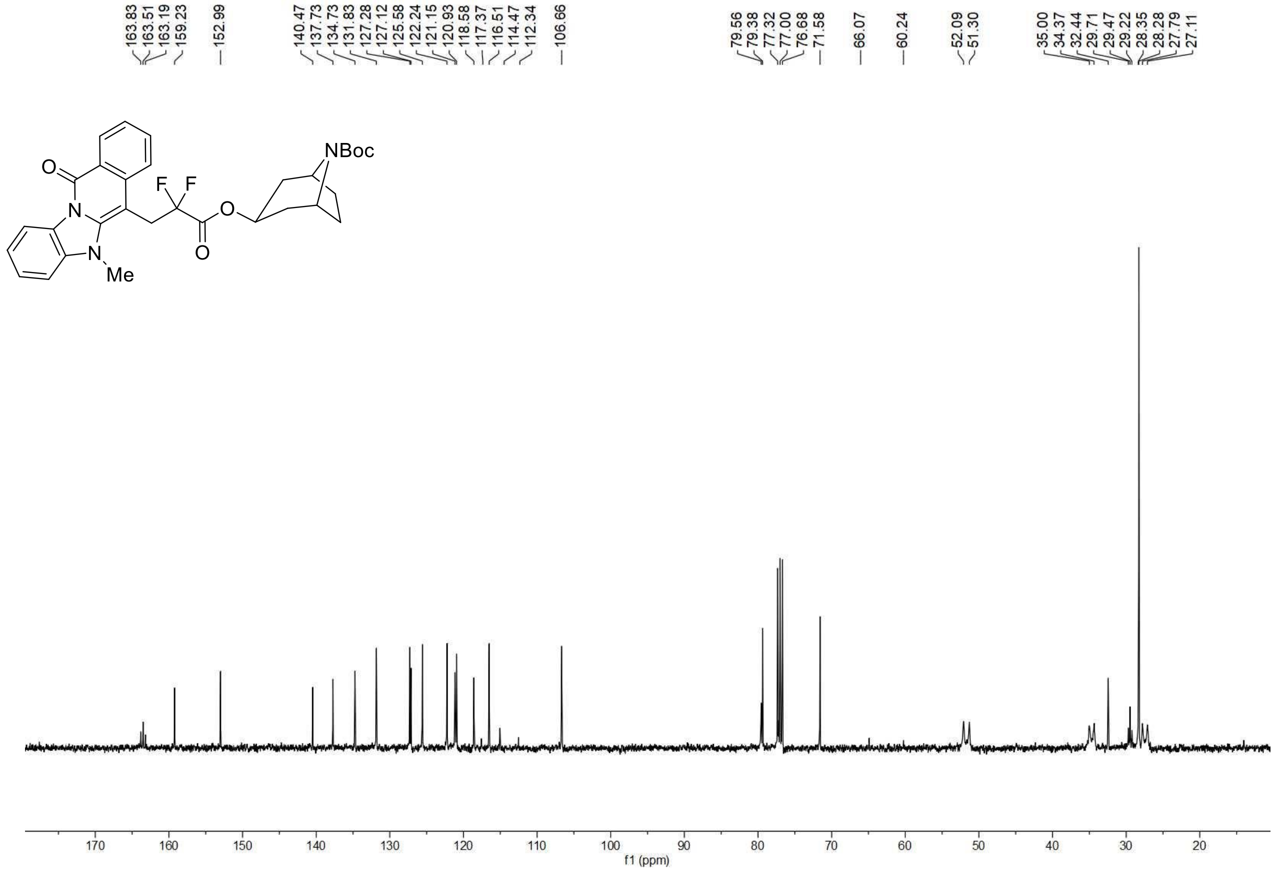

Supplementary Figure 225. ^{13}C-NMR of compound **66**, recorded at 100 MHz and 25 °C in $CDCl_3$.

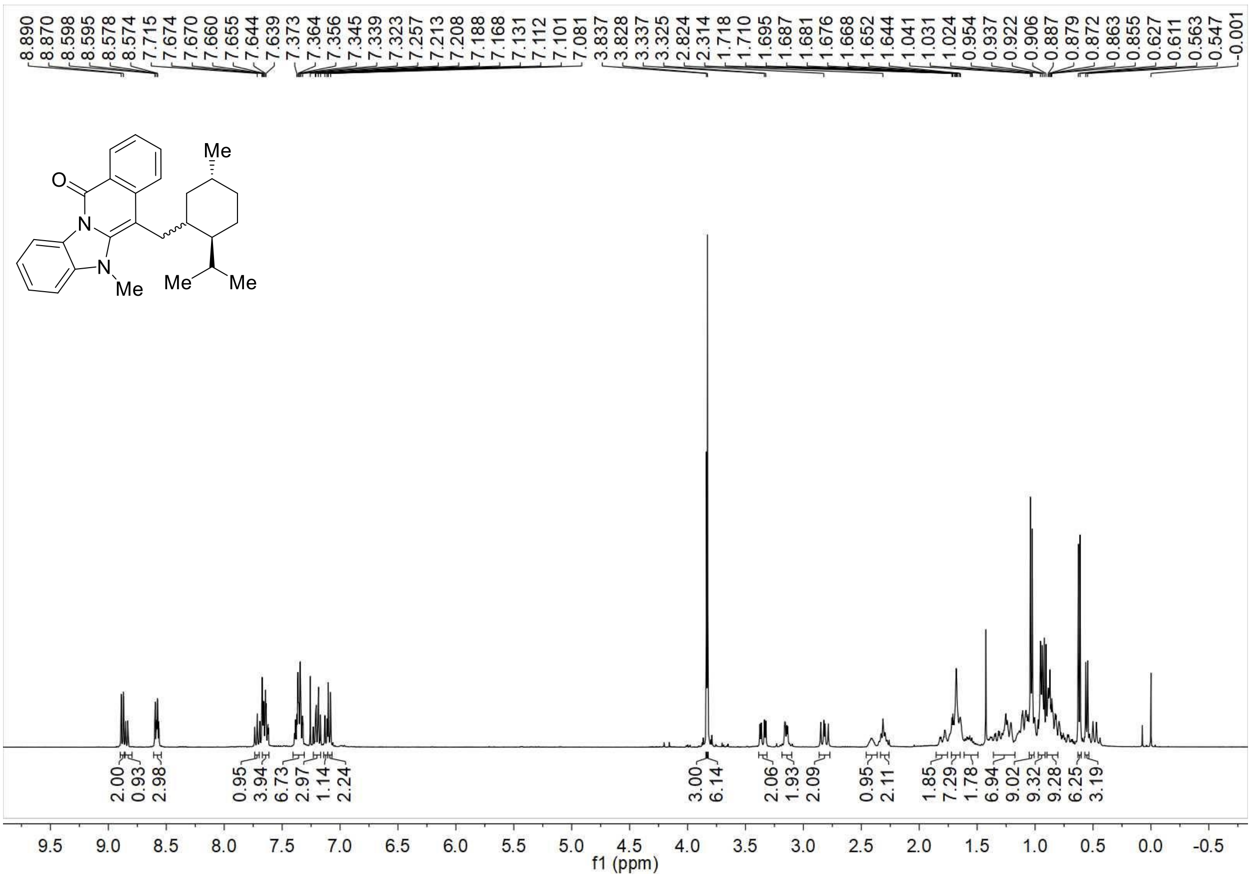

Supplementary Figure 226. ^{1}H-NMR of compound **67**, recorded at 400 MHz and 25 °C in $CDCl_3$.

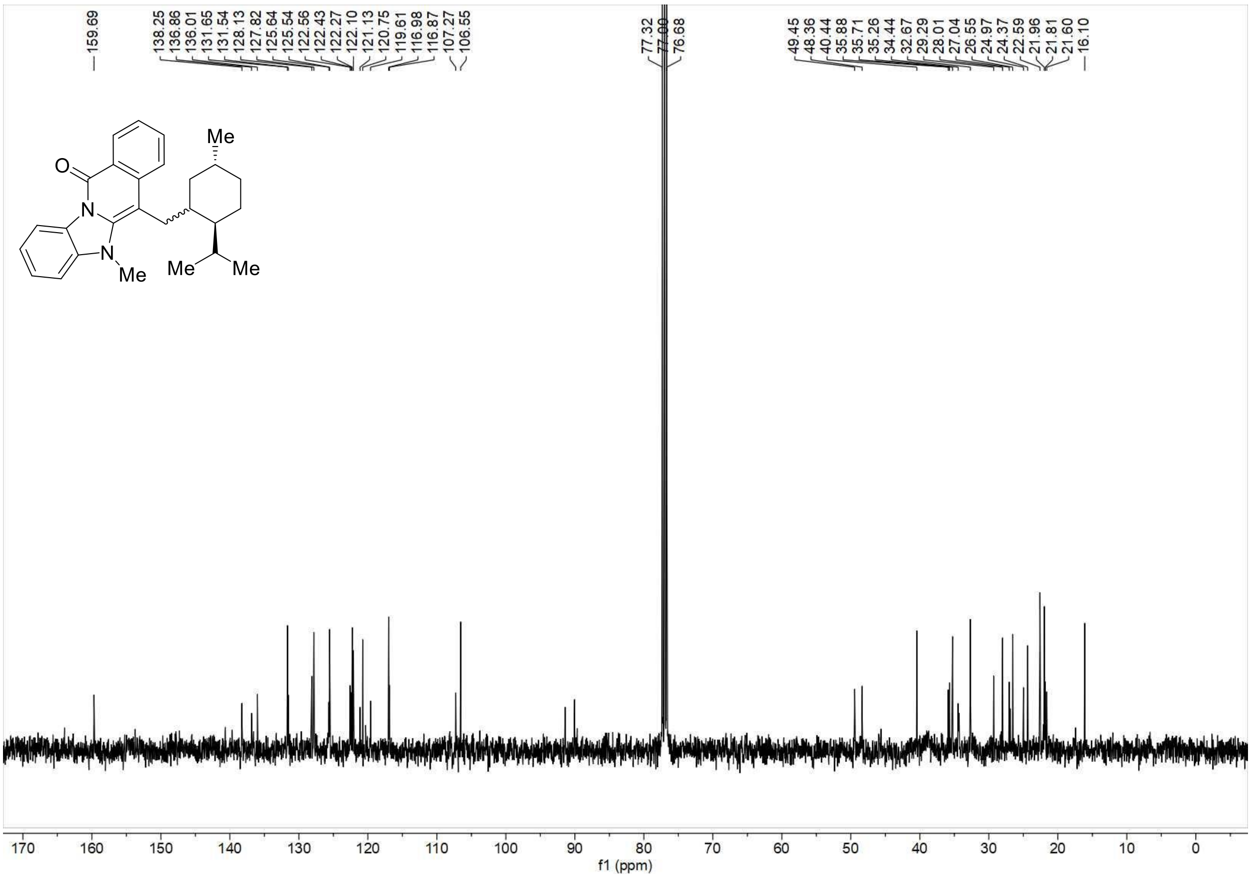

Supplementary Figure 227. ^{13}C-NMR of compound **67**, recorded at 100 MHz and 25 °C in $CDCl_3$.

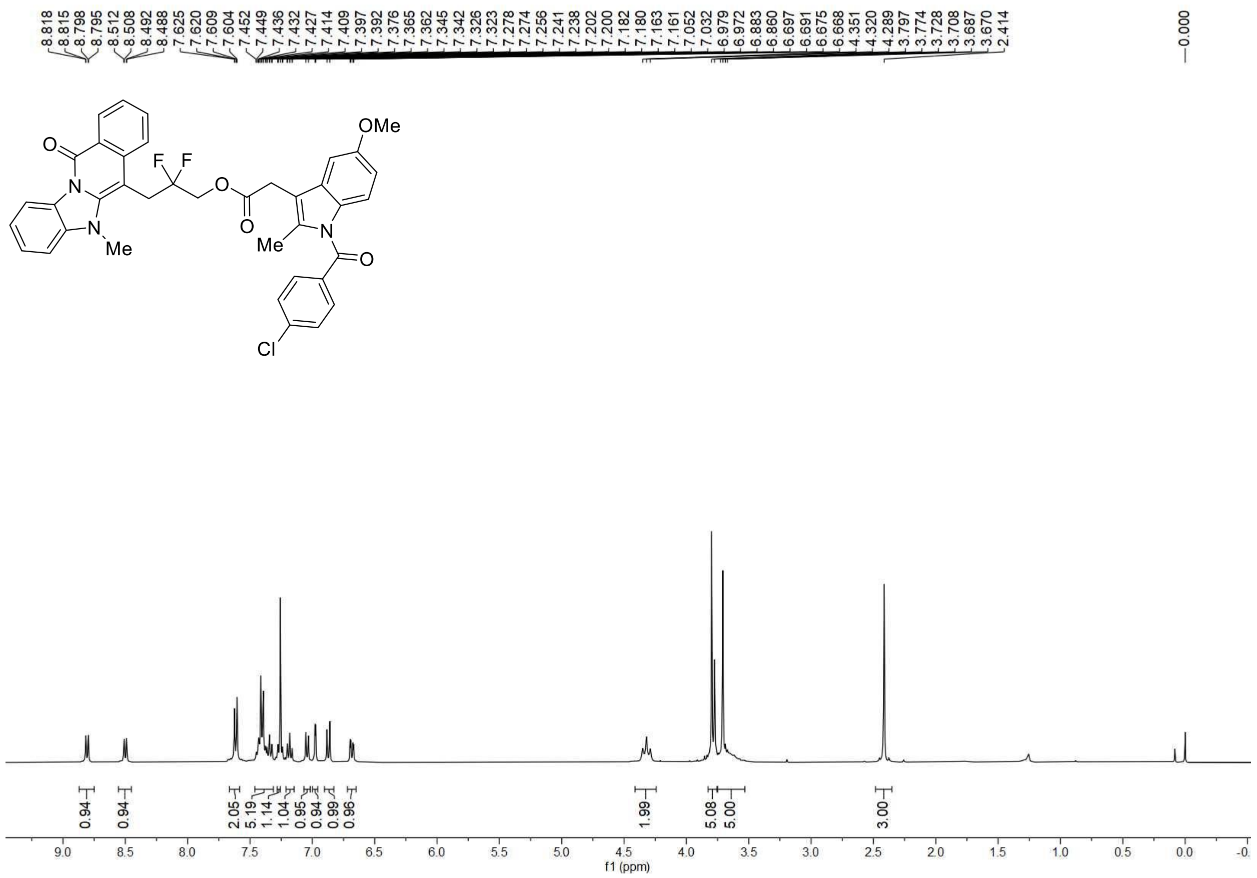

Supplementary Figure 228. ^{1}H-NMR of compound **68**, recorded at 400 MHz and 25 °C in $CDCl_3$.

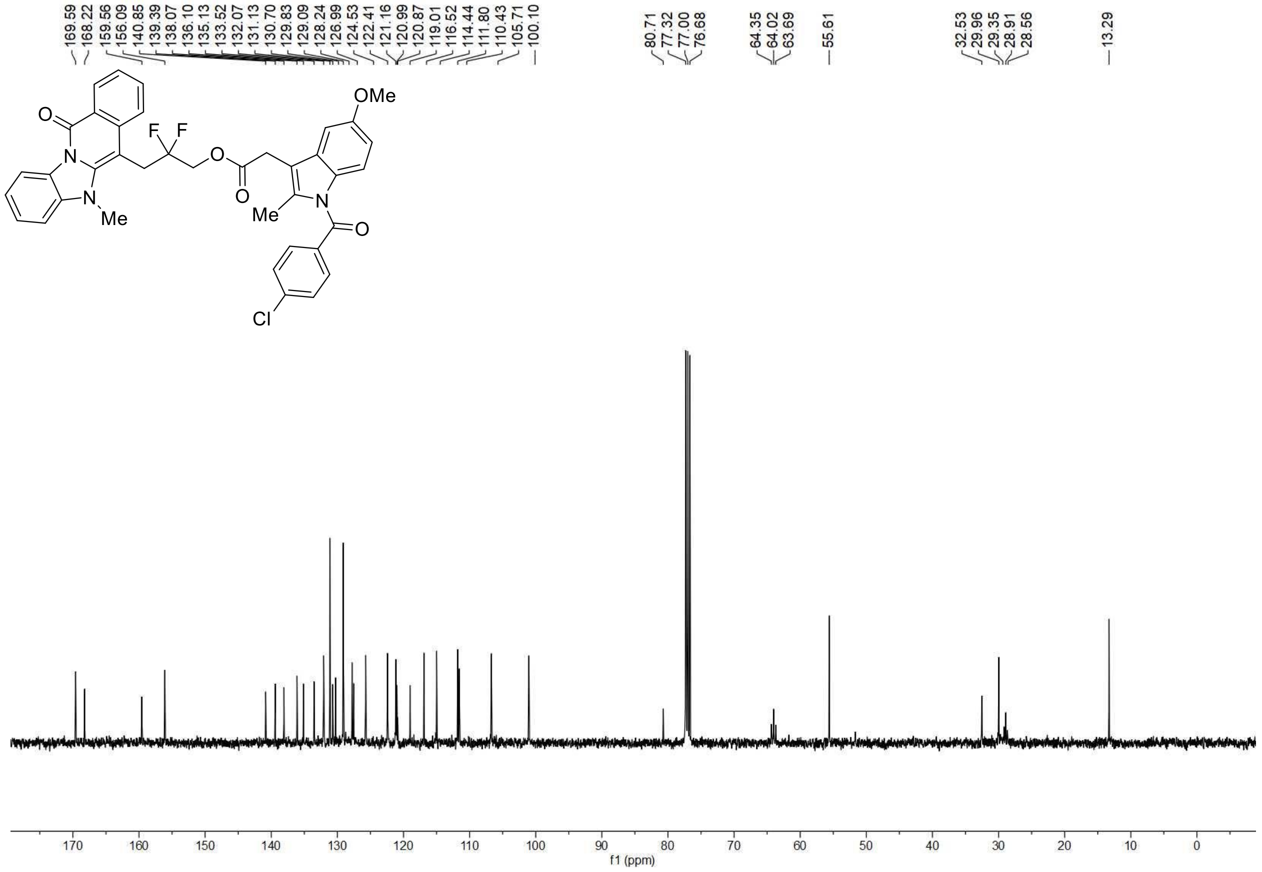

Supplementary Figure 229. ^{13}C-NMR of compound **68**, recorded at 100 MHz and 25 °C in $CDCl_3$.

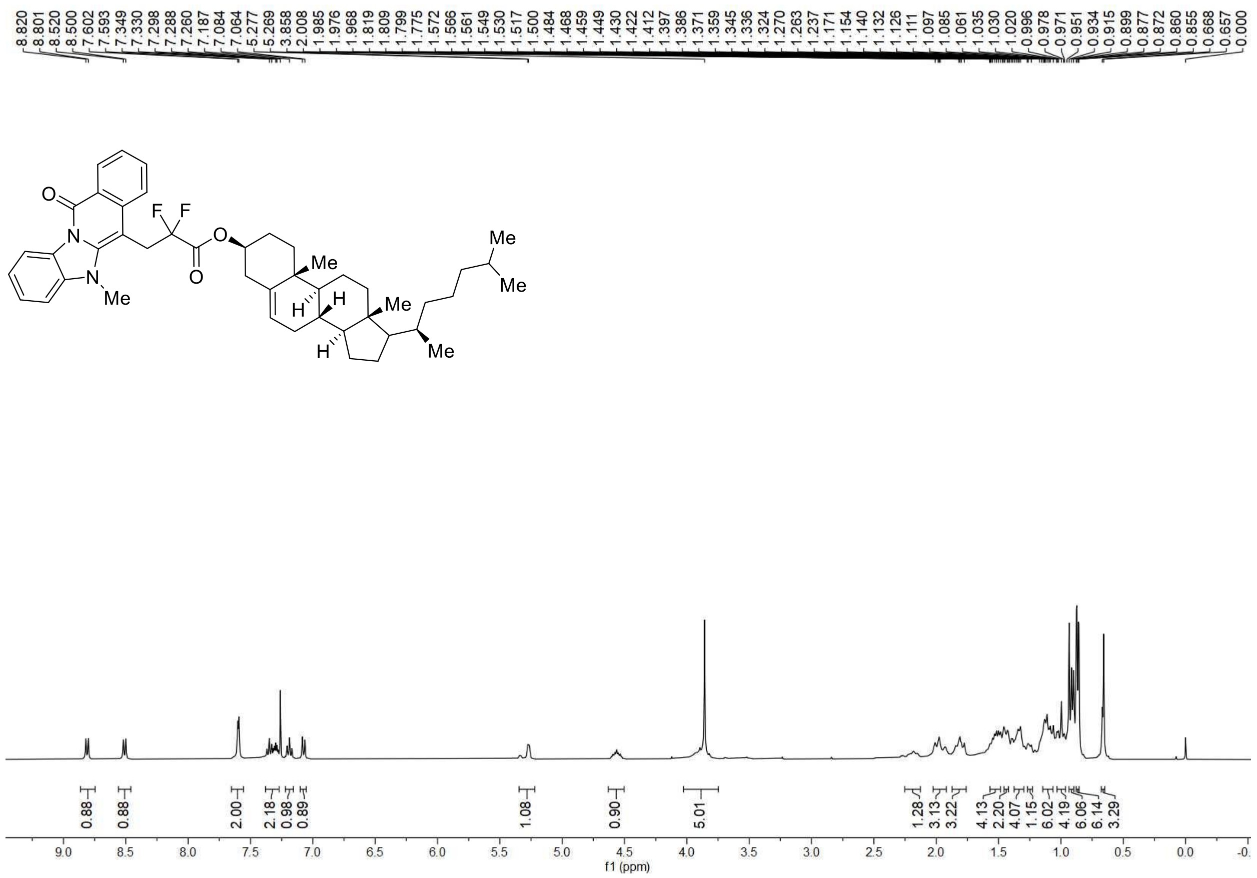

Supplementary Figure 230. ^{1}H-NMR of compound **69**, recorded at 400 MHz and 25 °C in $CDCl_3$.

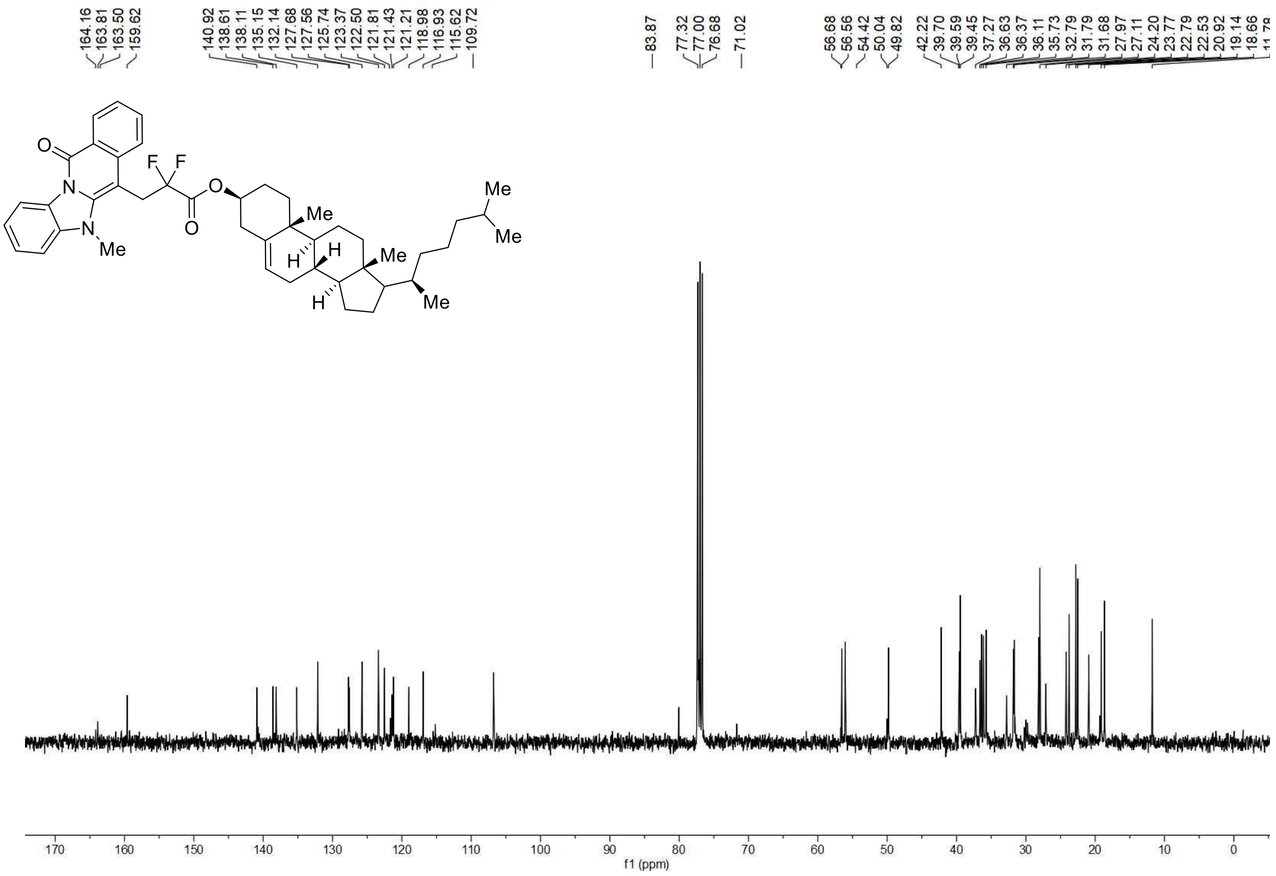

Supplementary Figure 231. ^{13}C-NMR of compound **69**, recorded at 100 MHz and 25 °C in $CDCl_3$.

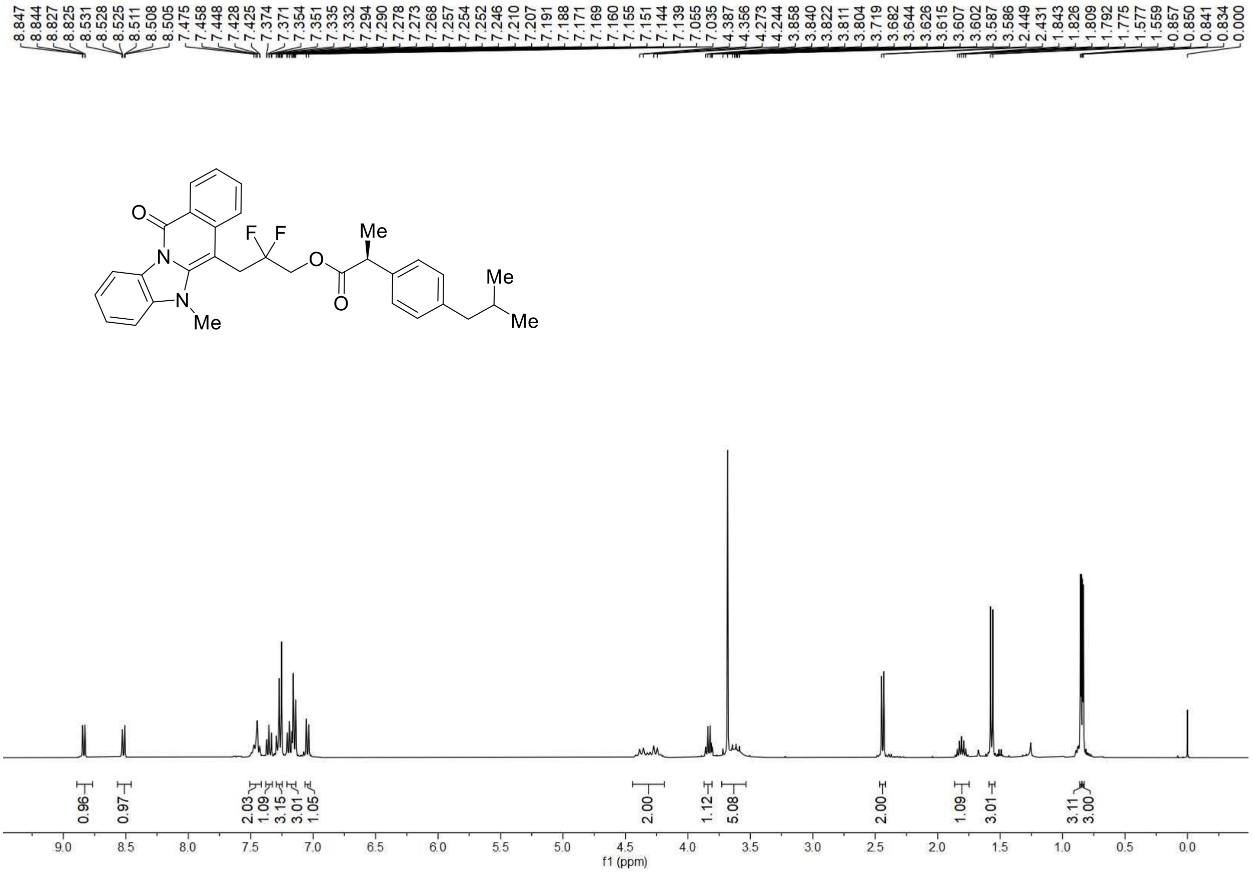

Supplementary Figure 232. ^{1}H-NMR of compound **70**, recorded at 400 MHz and 25 °C in $CDCl_3$.

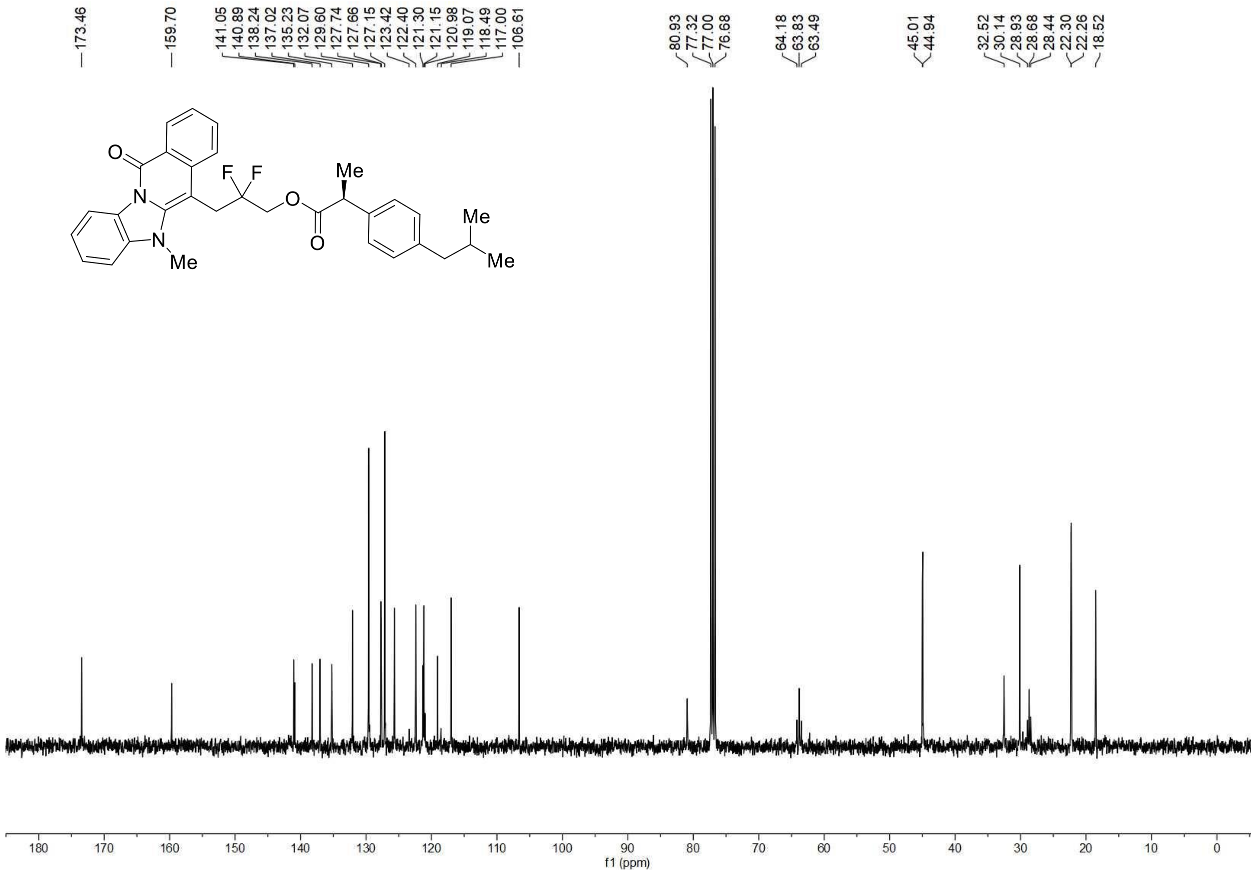

Supplementary Figure 233. ^{13}C-NMR of compound **70**, recorded at 100 MHz and 25 °C in $CDCl_3$.

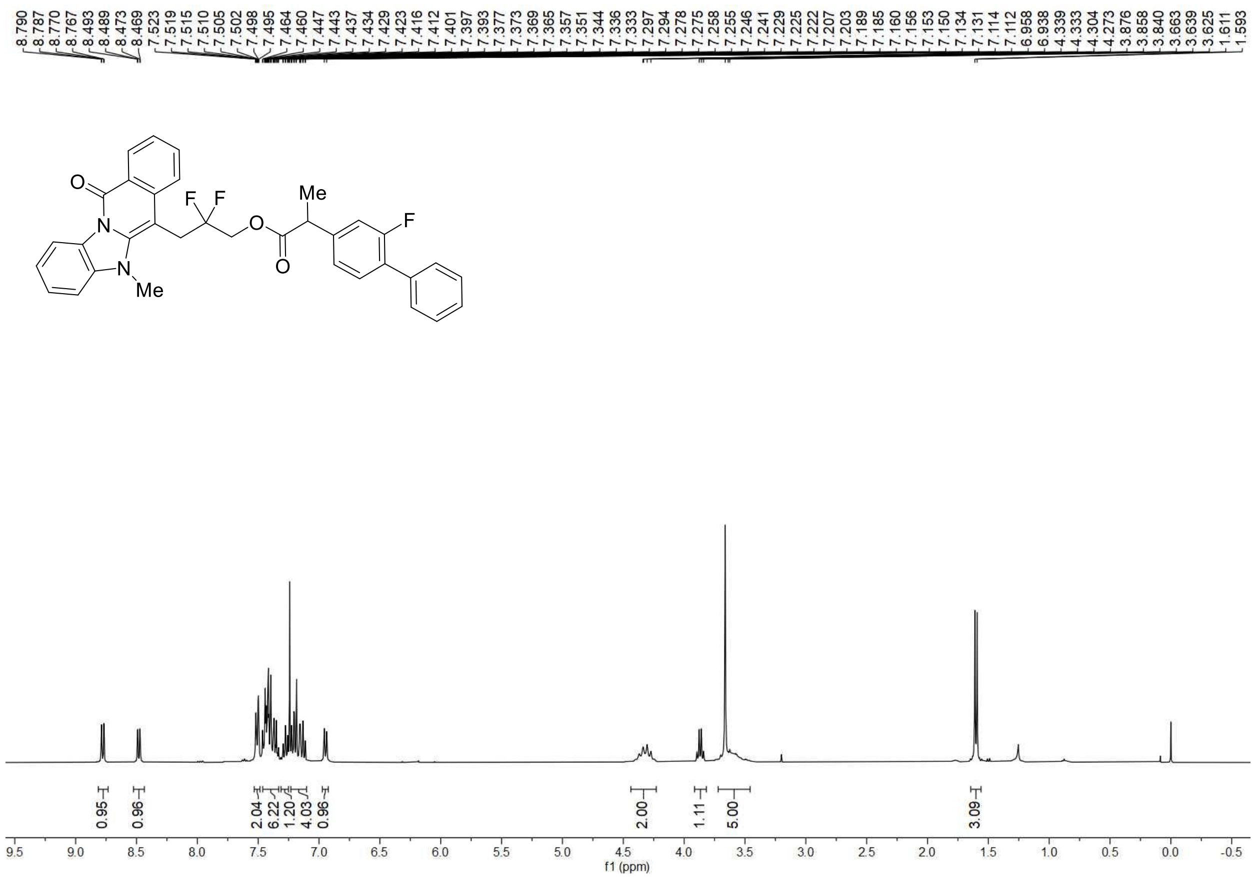

Supplementary Figure 234. ^{1}H-NMR of compound **71**, recorded at 400 MHz and 25 °C in $CDCl_3$.

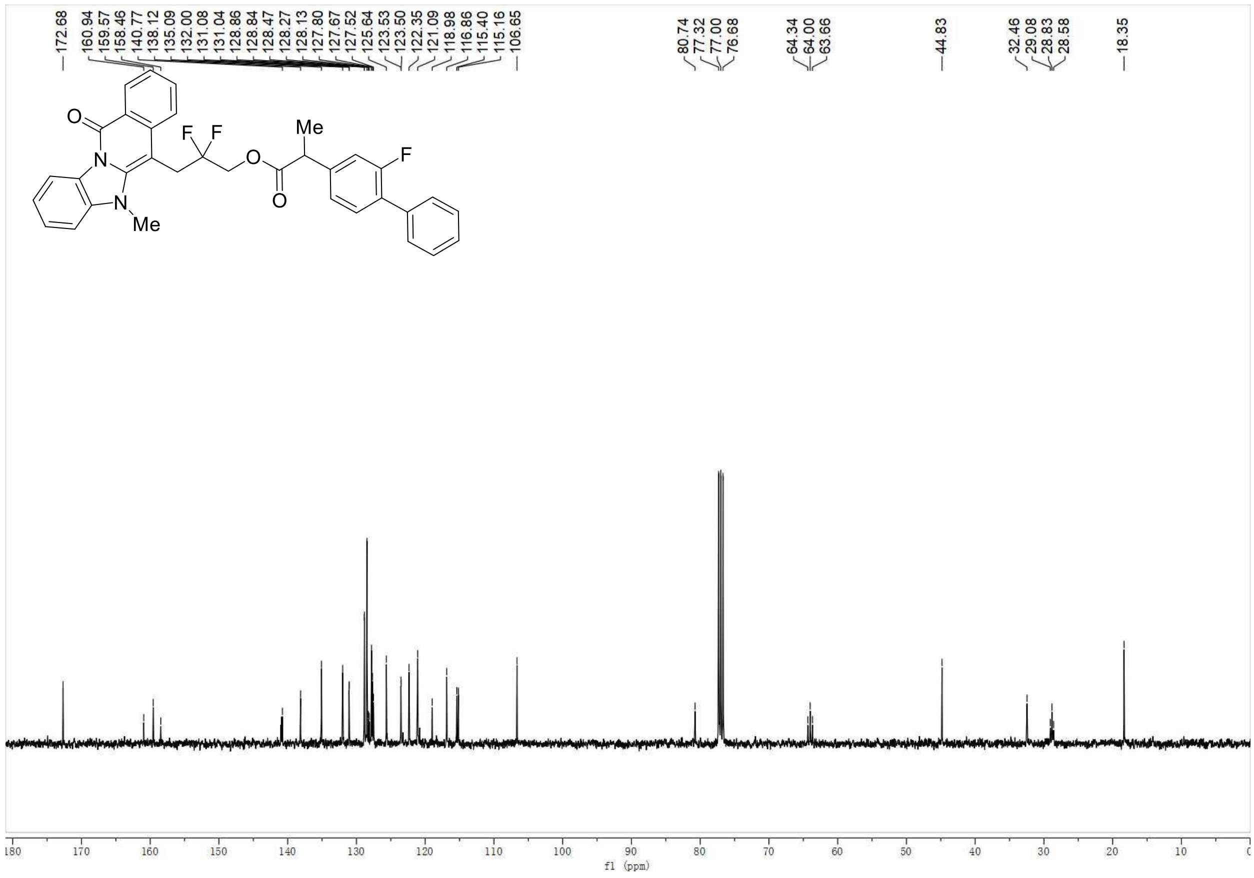

Supplementary Figure 235. ^{13}C-NMR of compound **71**, recorded at 100 MHz and 25 °C in $CDCl_3$.

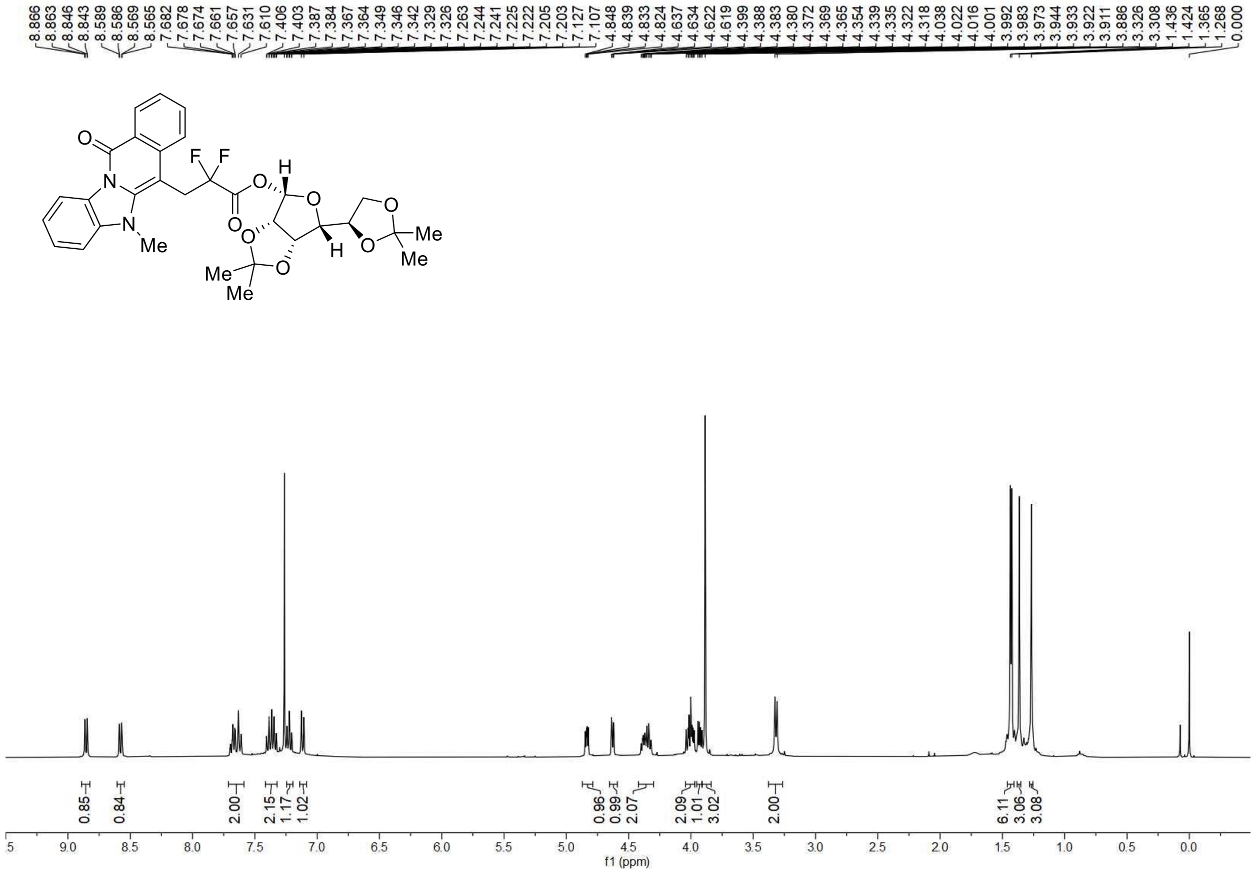

Supplementary Figure 236. ^{1}H-NMR of compound **72**, recorded at 400 MHz and 25 °C in $CDCl_3$.

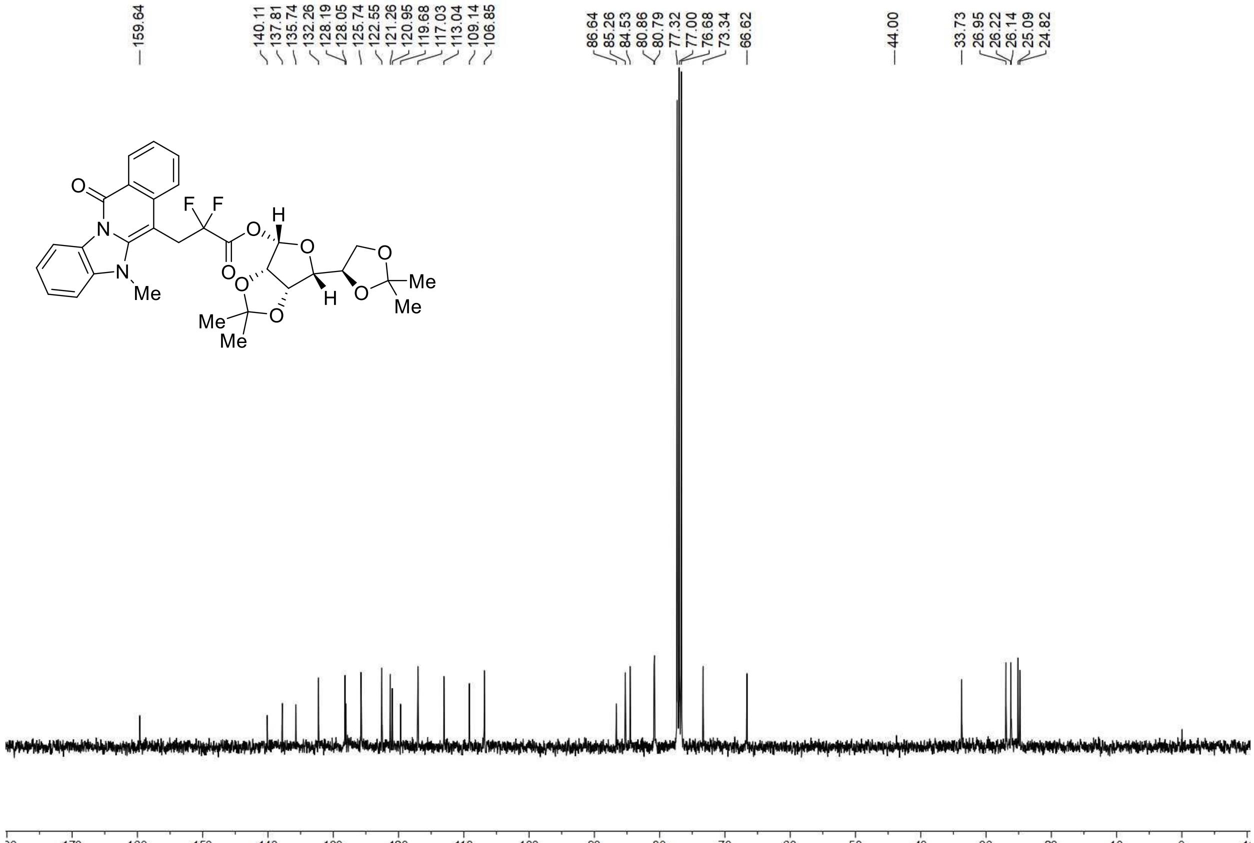

Supplementary Figure 237. ^{13}C-NMR of compound **72**, recorded at 100 MHz and 25 °C in $CDCl_3$.

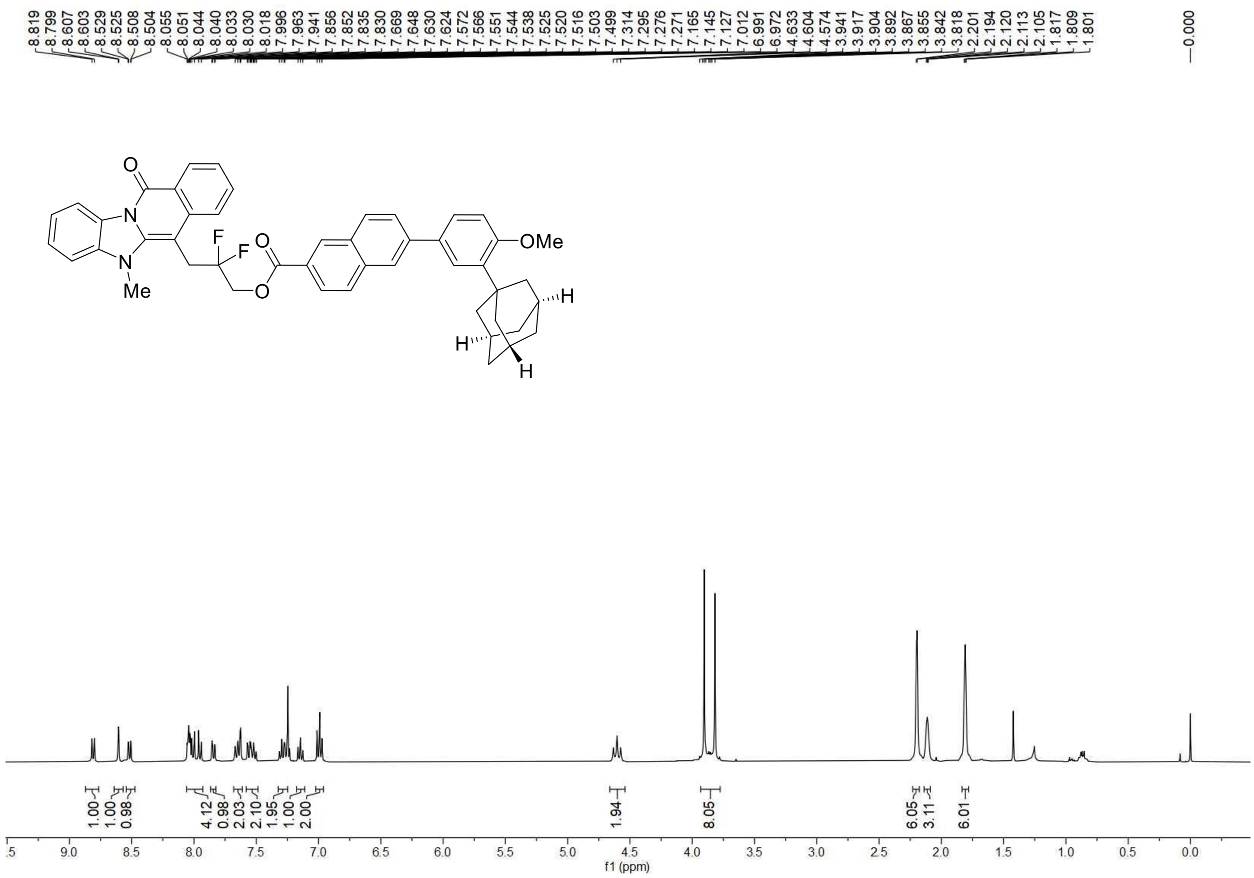

Supplementary Figure 238. ^{1}H-NMR of compound **73**, recorded at 400 MHz and 25 °C in $CDCl_3$.

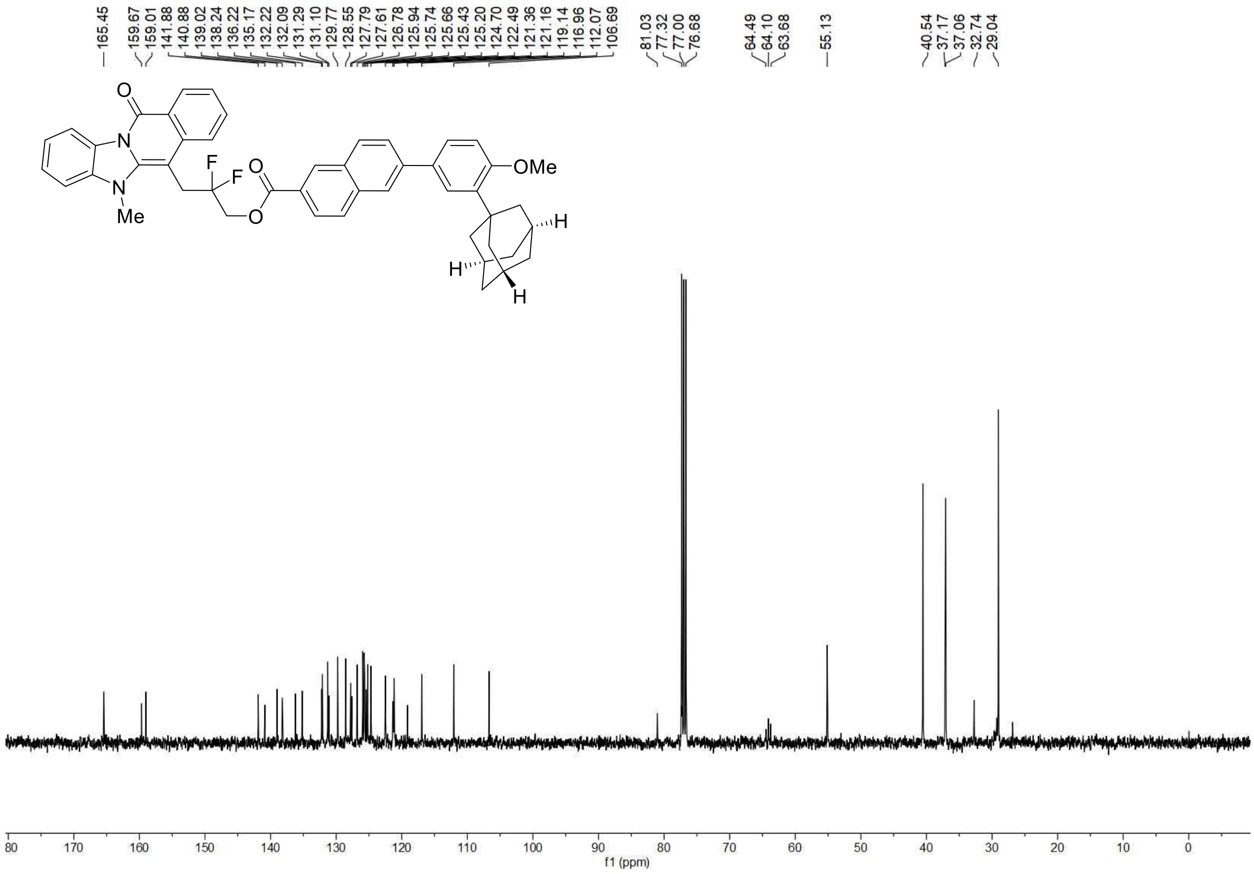

Supplementary Figure 239. ^{13}C-NMR of compound **73**, recorded at 100 MHz and 25 °C in $CDCl_3$.

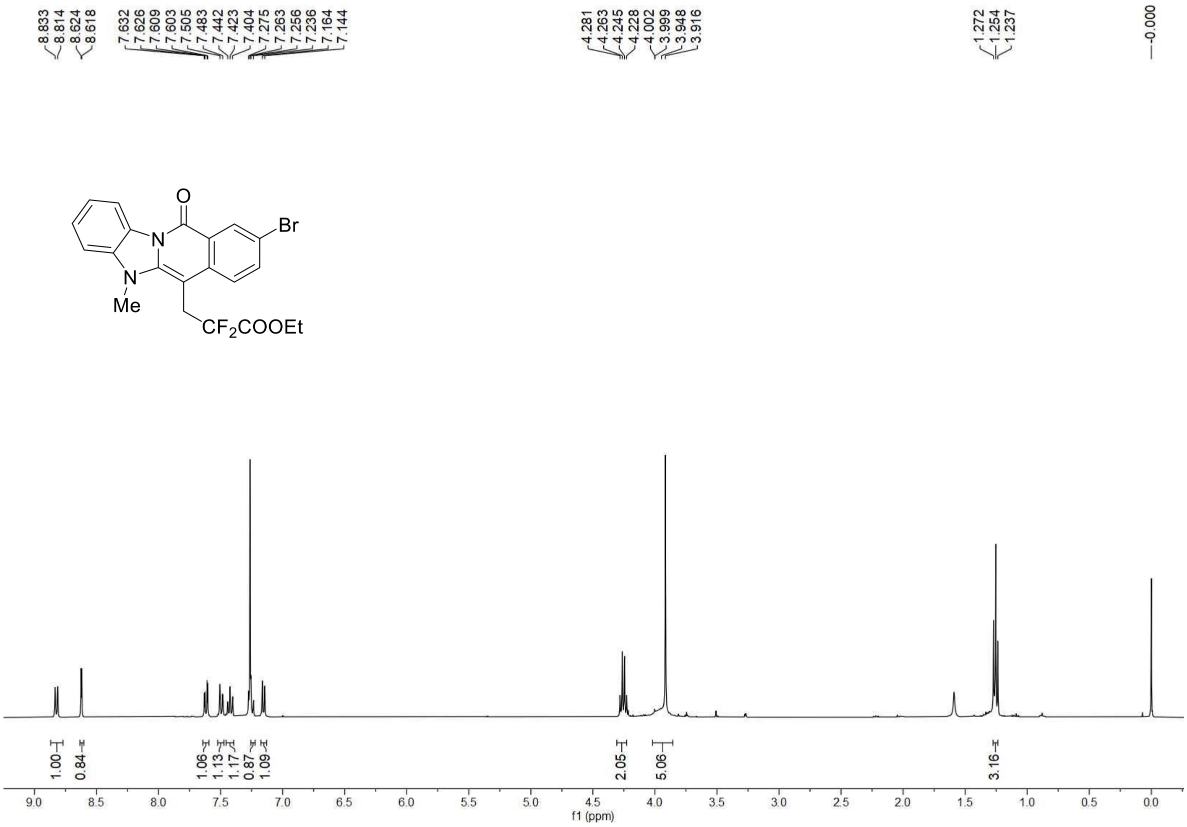

Supplementary Figure 240. ^{1}H-NMR of compound **74**, recorded at 400 MHz and 25 °C in $CDCl_3$.

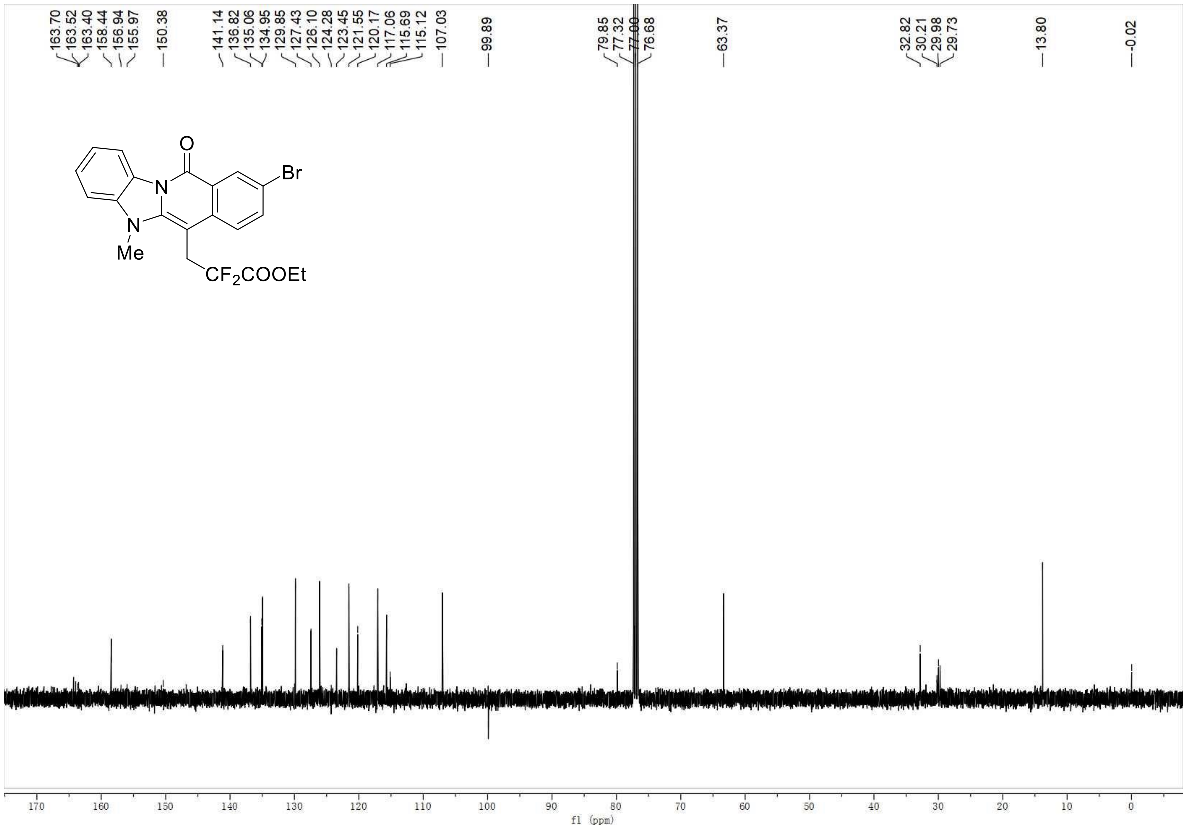

Supplementary Figure 241. ^{13}C-NMR of compound **74**, recorded at 100 MHz and 25 °C in $CDCl_3$.

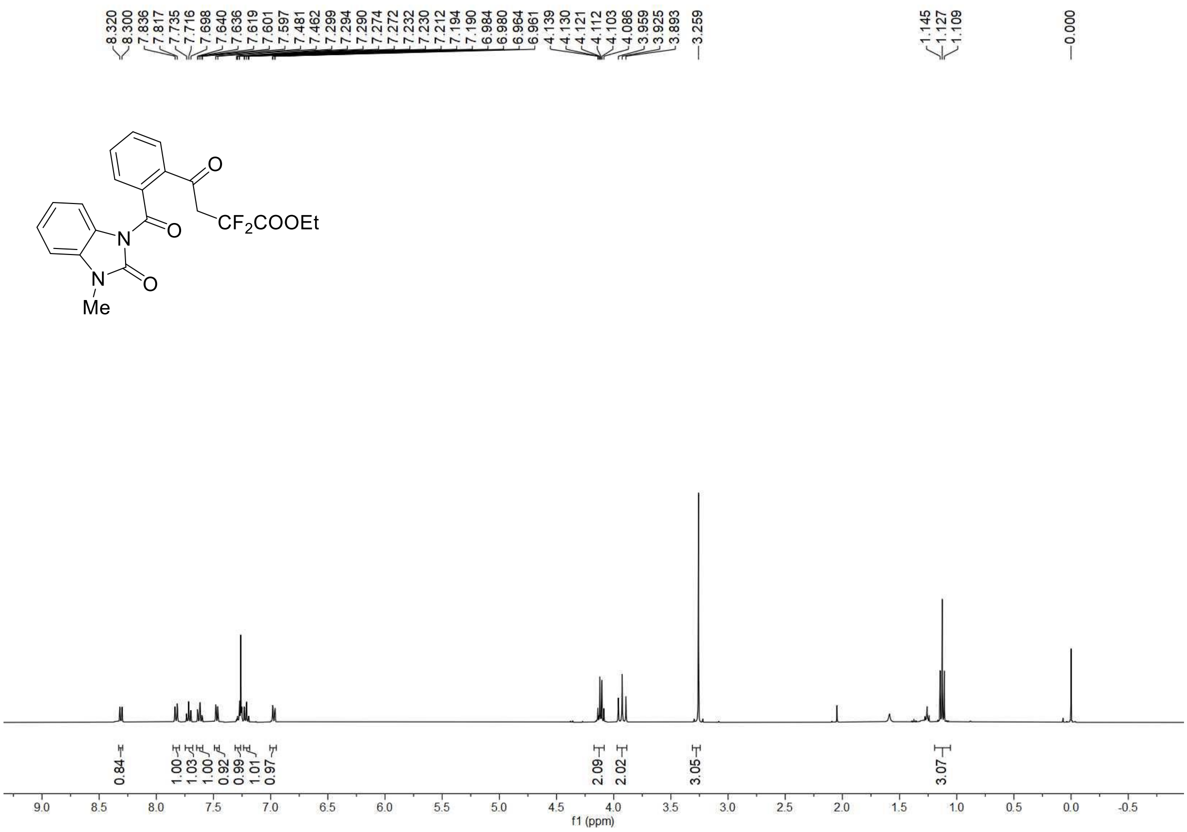

Supplementary Figure 242. ^{1}H-NMR of compound **75**, recorded at 400 MHz and 25 °C in $CDCl_3$.

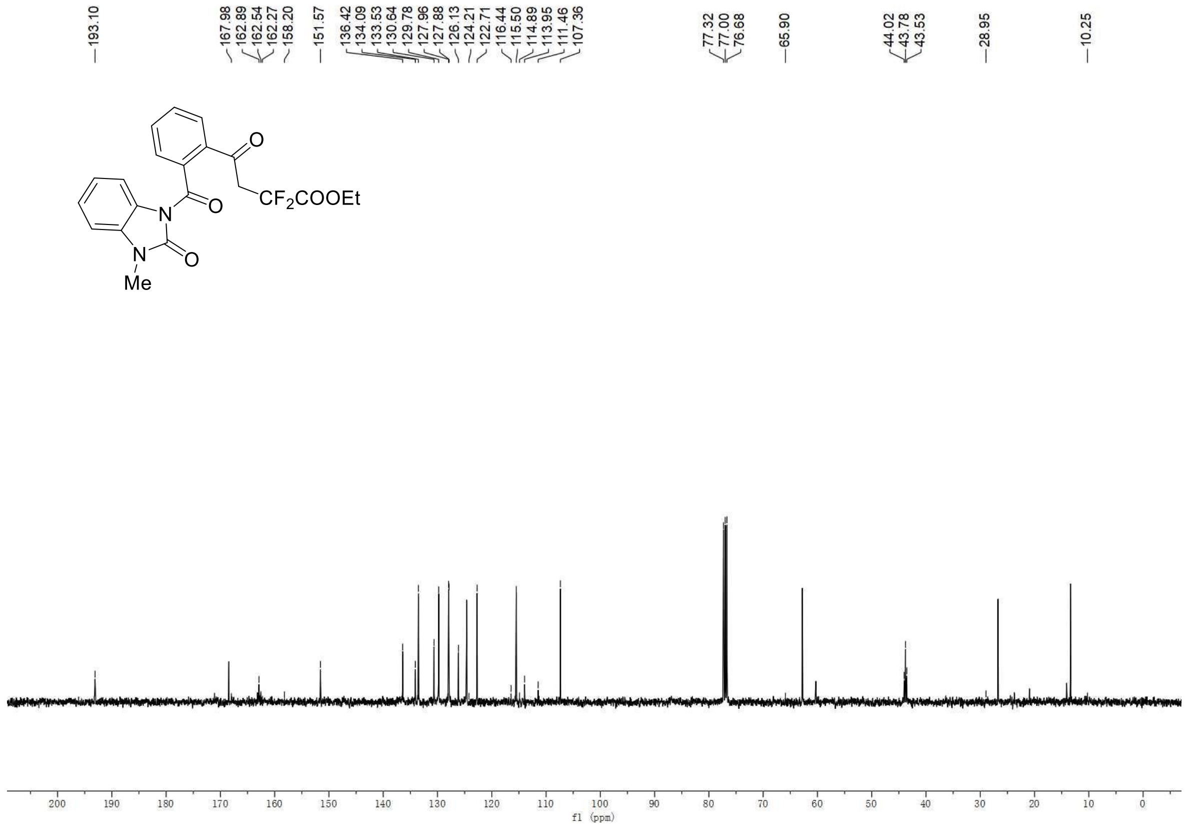

Supplementary Figure 243. ^{13}C-NMR of compound **75**, recorded at 100 MHz and 25 °C in $CDCl_3$.

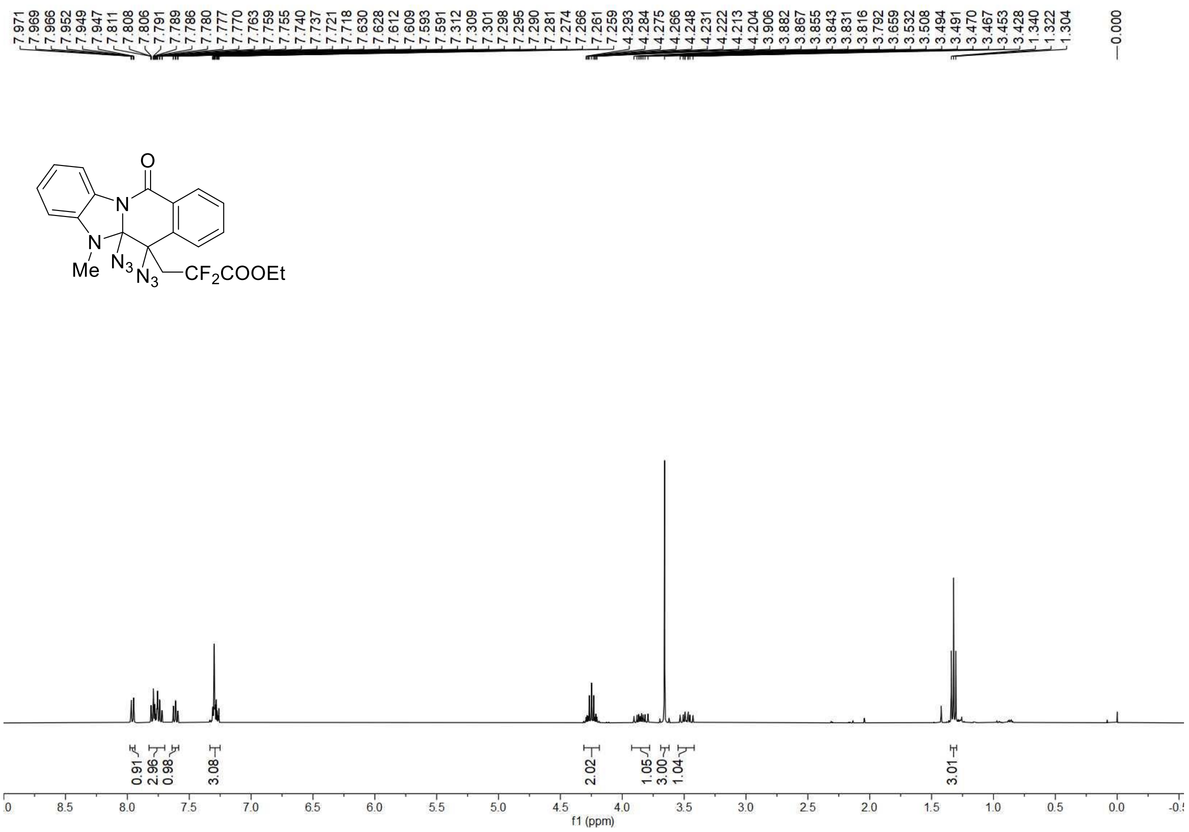

Supplementary Figure 244. ^{1}H-NMR of compound **76**, recorded at 400 MHz and 25 °C in $CDCl_3$.

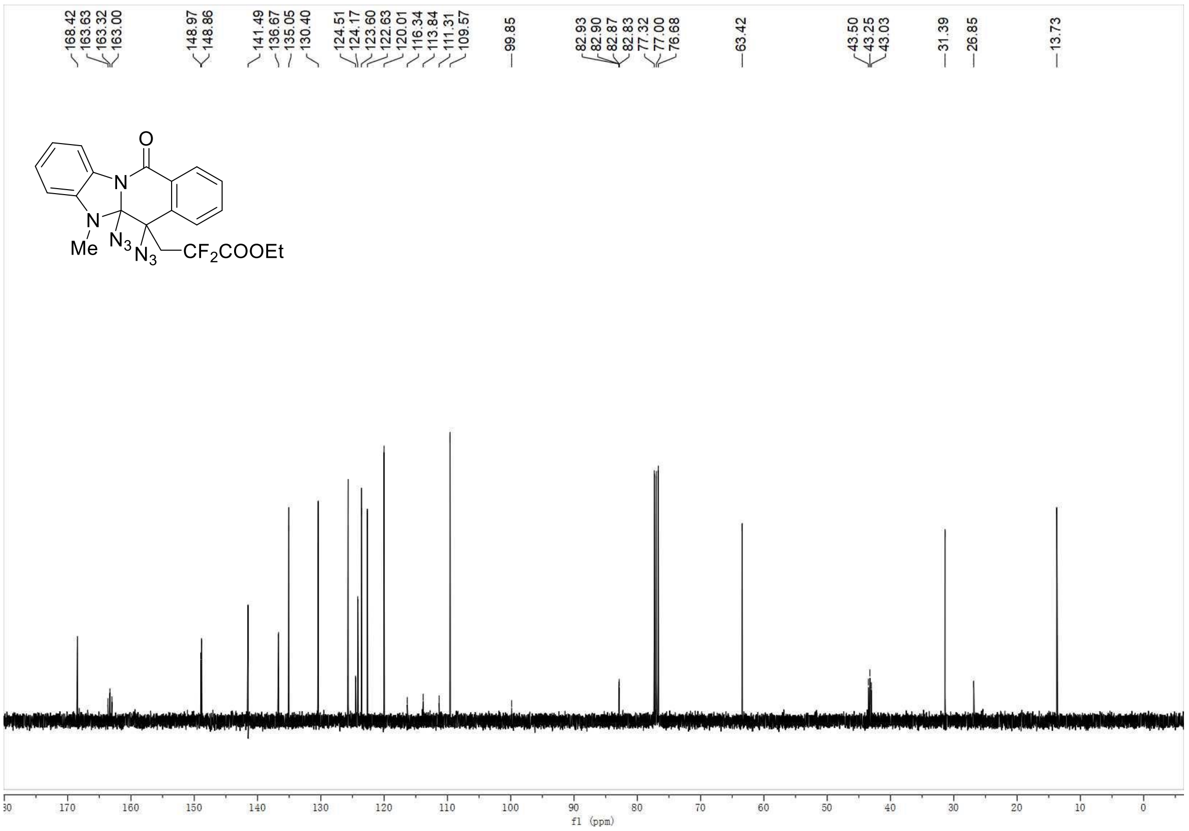

Supplementary Figure 245. ^{13}C-NMR of compound **76**, recorded at 100 MHz and 25 °C in $CDCl_3$.

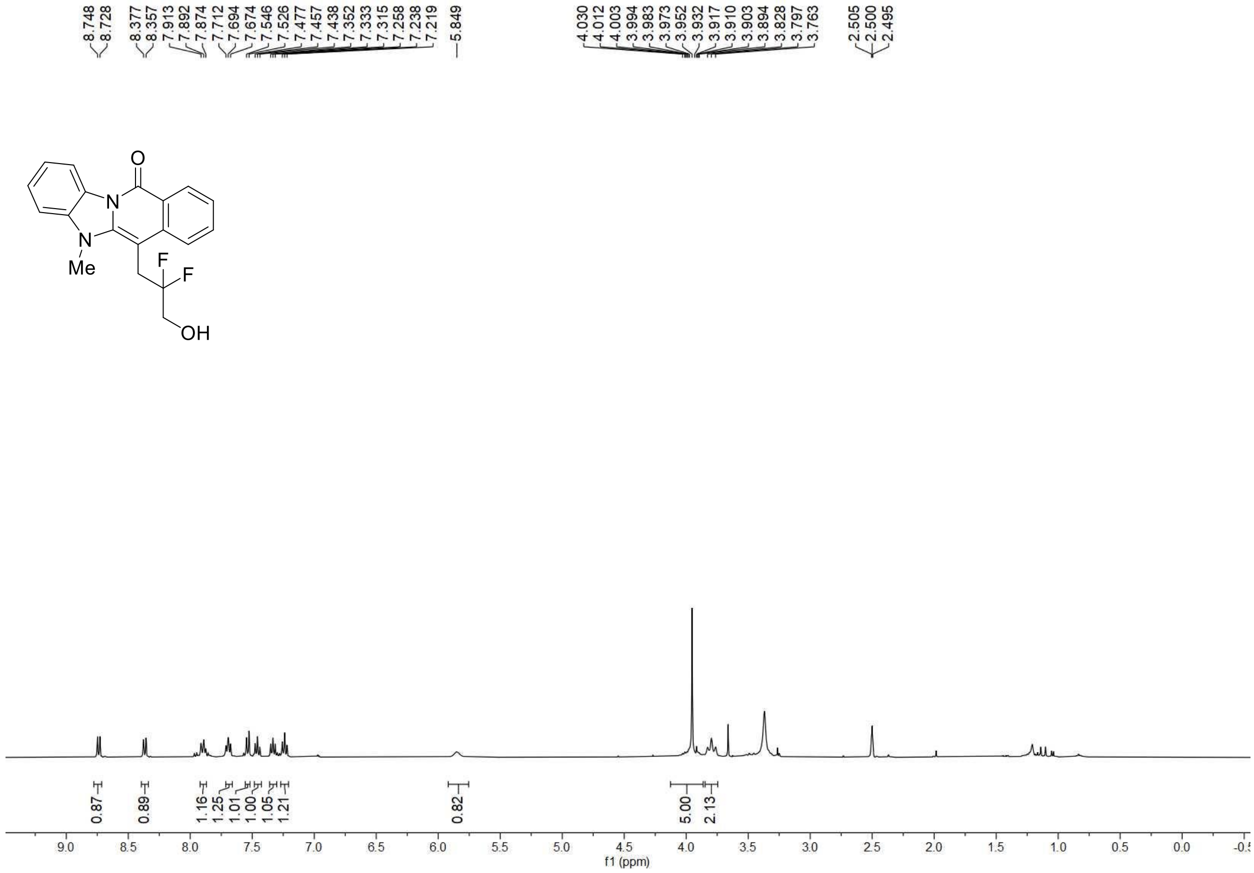

Supplementary Figure 246. ^{1}H-NMR of compound **77**, recorded at 400 MHz and 25 °C in DMSO-d_6.

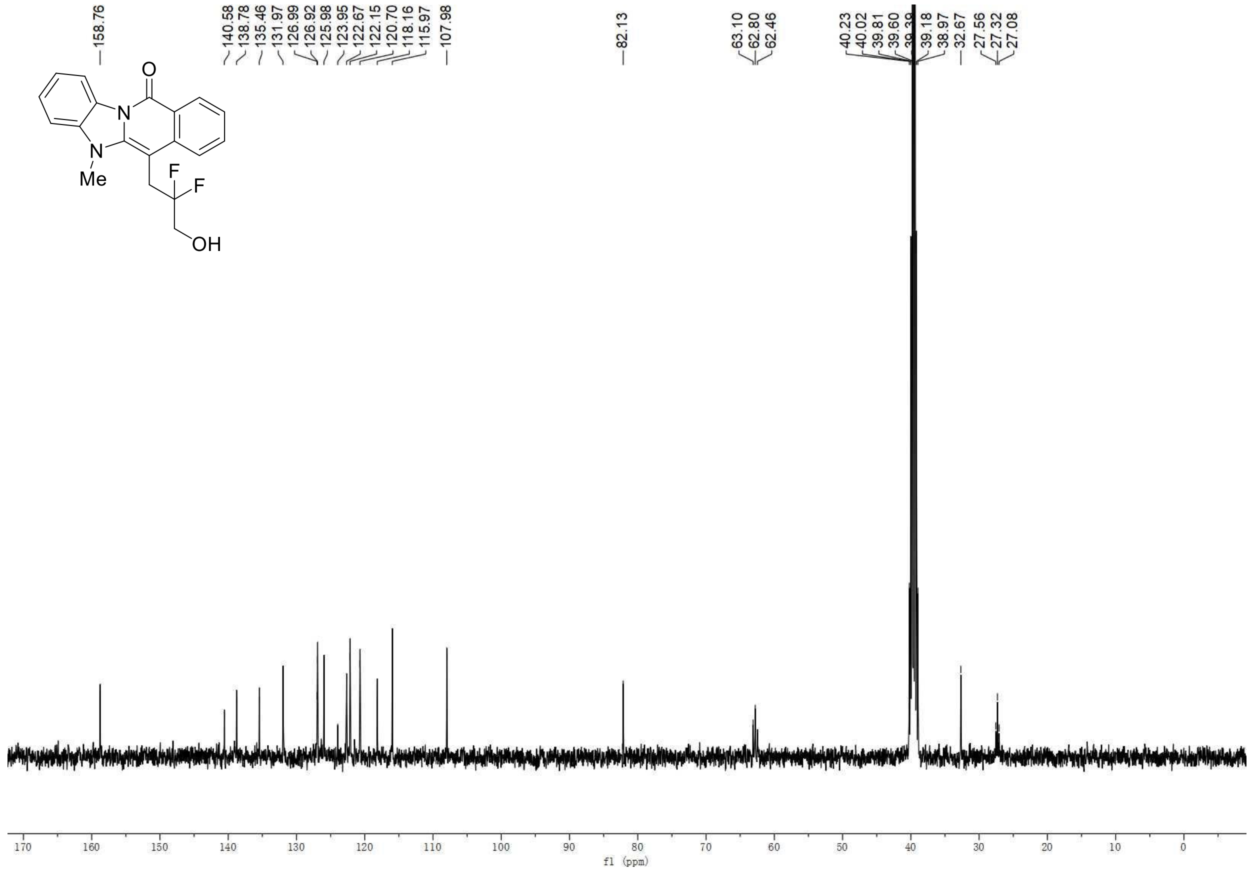

Supplementary Figure 247. ^{13}C-NMR of compound **77**, recorded at 100 MHz and 25 °C in DMSO-d_6.

3.4.2 Copies of ^{2}H spectra

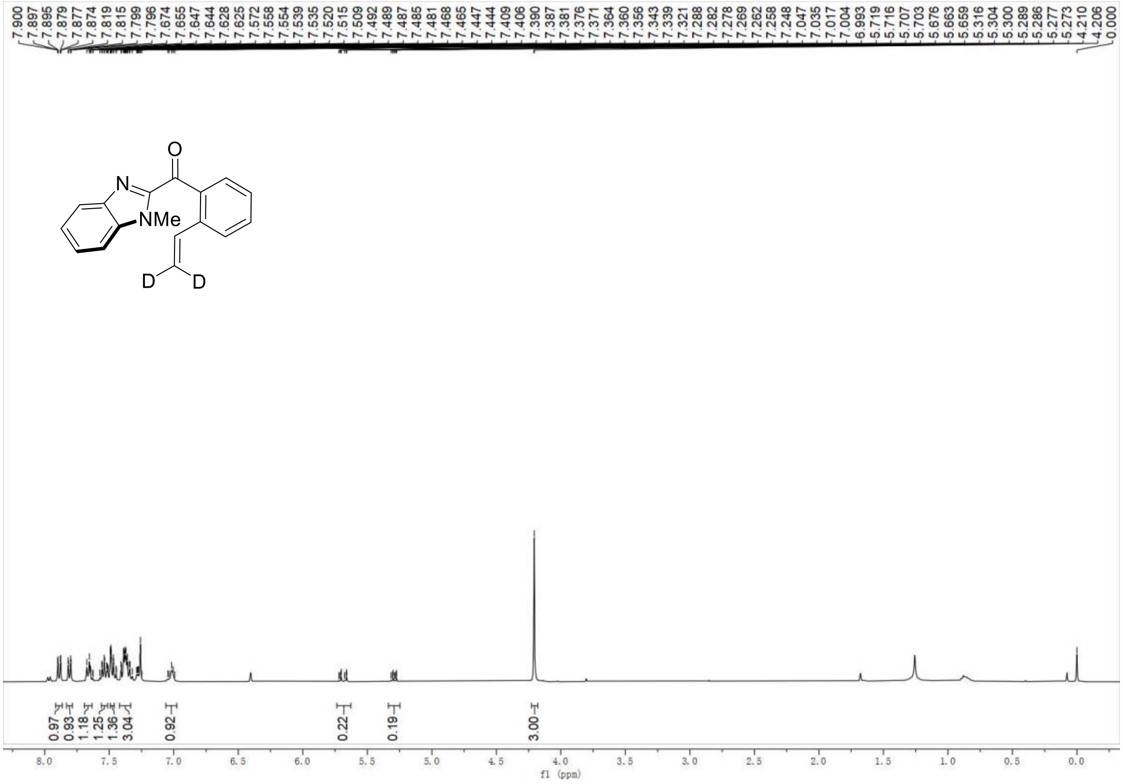

Supplementary Figure 248. ^{2}H-NMR of compound **D-1**, recorded at 400 MHz and 25 °C in $CDCl_3$.

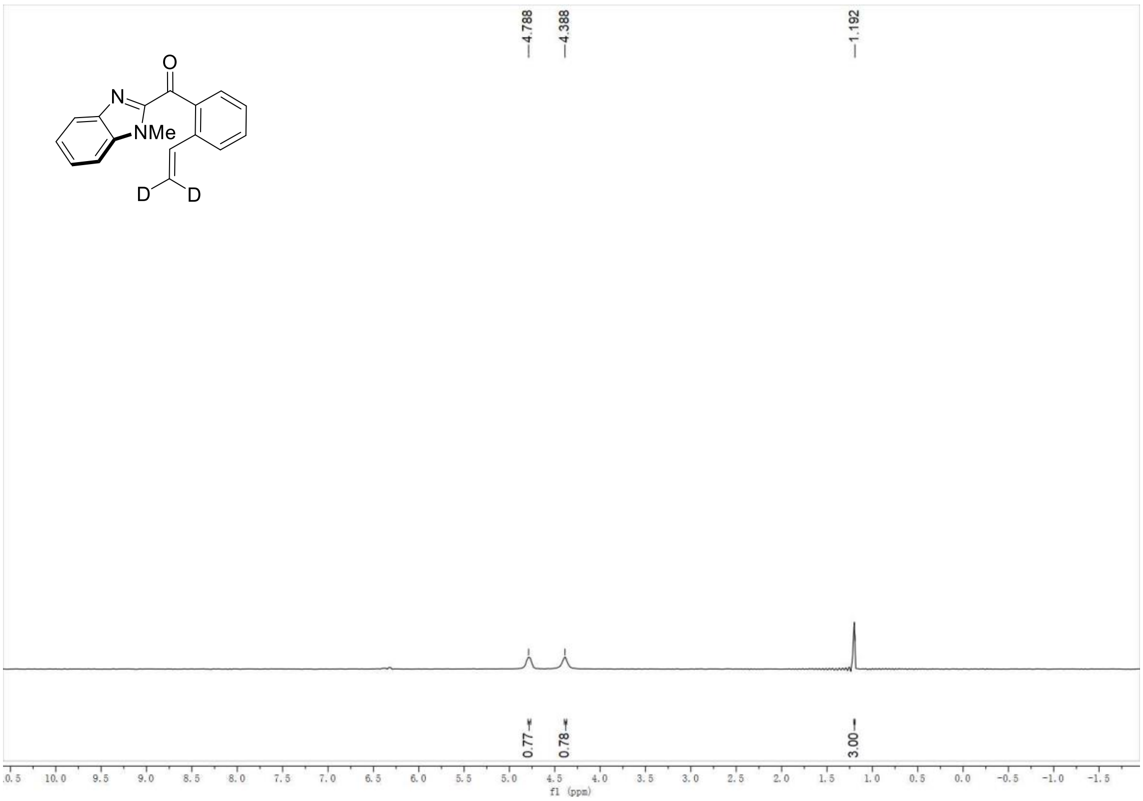

Supplementary Figure 249. ^{2}H-NMR of compound **D-1**, recorded at 400 MHz and 25 °C in $CHCl_3$. The ^{2}H NMR spectroscopy was measured with methyl acetone-d6 as an internal standard (0.5 equiv) as an internal standard.

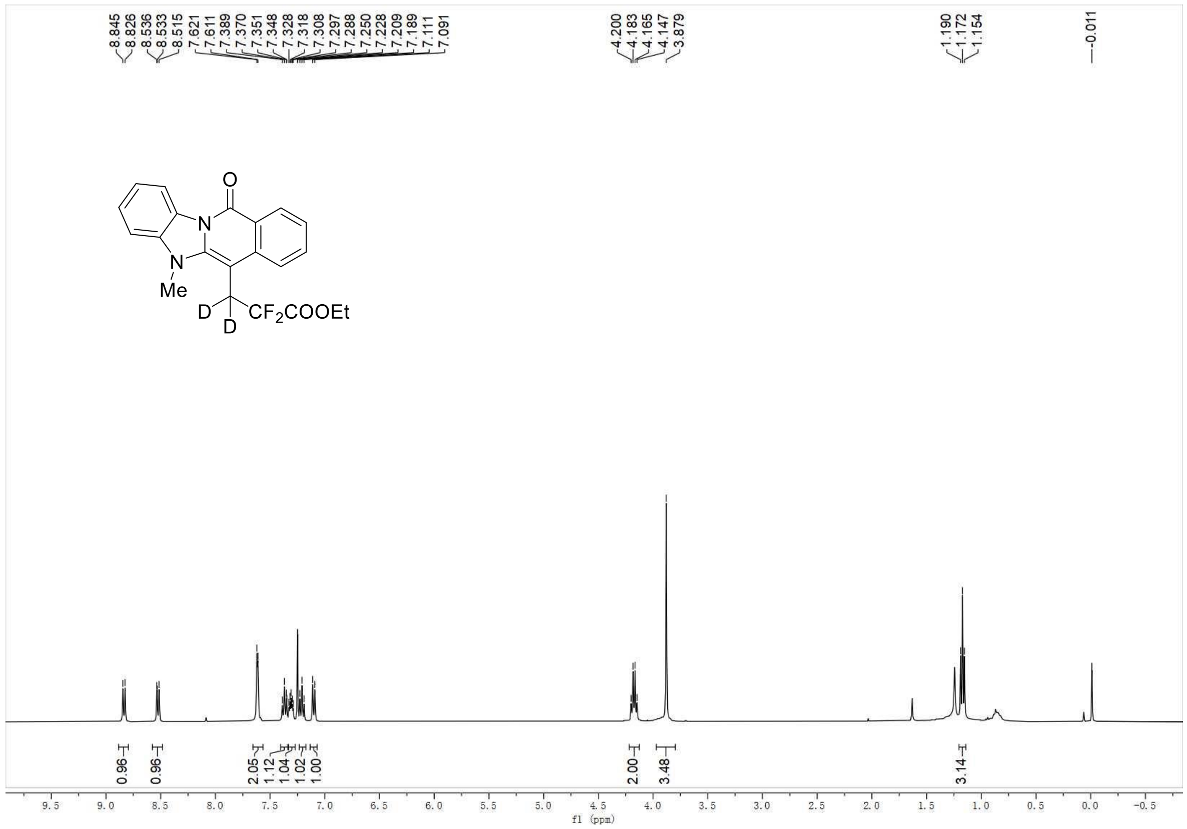

Supplementary Figure 250. ^{2}H-NMR of compound **D-2**, recorded at 400 MHz and 25 °C in $CDCl_3$.

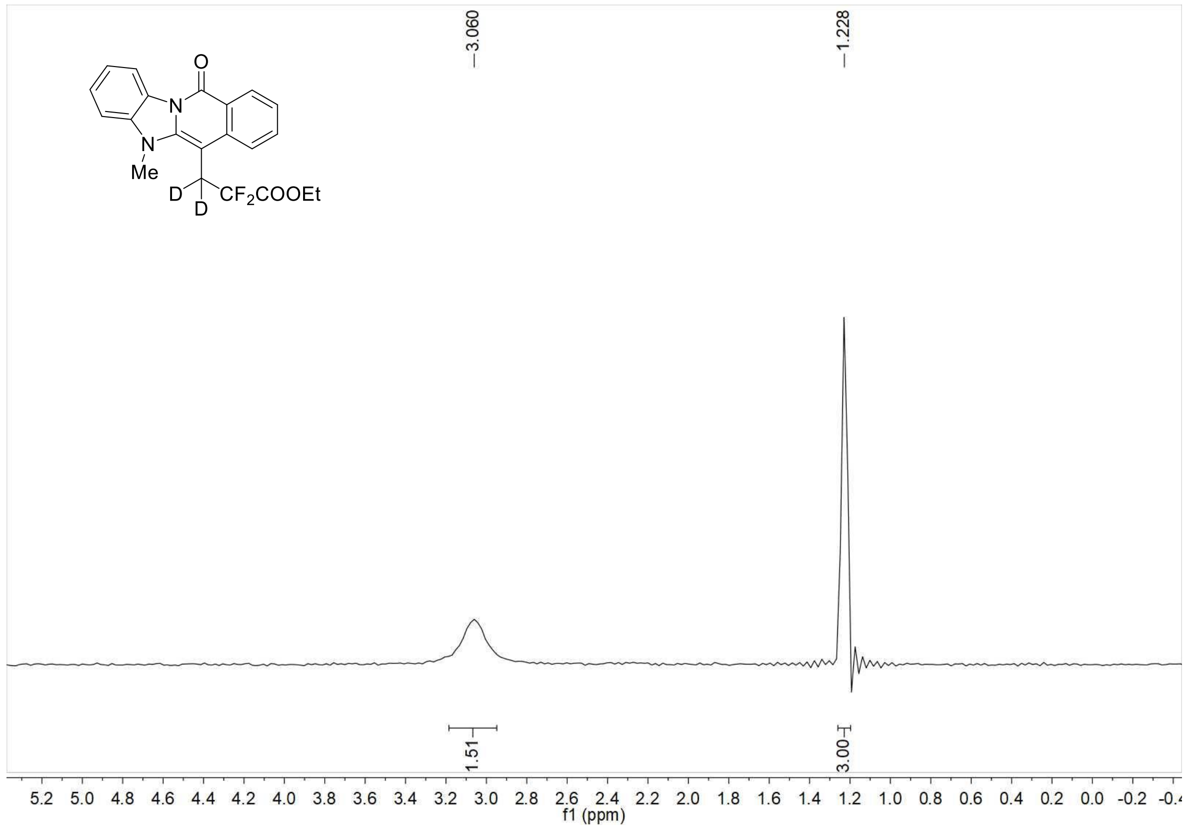

Supplementary Figure 251. ^{2}H-NMR of compound **D-2**, recorded at 400 MHz and 25 °C in $CHCl_3$. The ^{2}H NMR spectroscopy was measured with methyl acetone-d6 as an internal standard (0.5 equiv) as an internal standard.

3.4.3 Copies of ^{19}F spectra

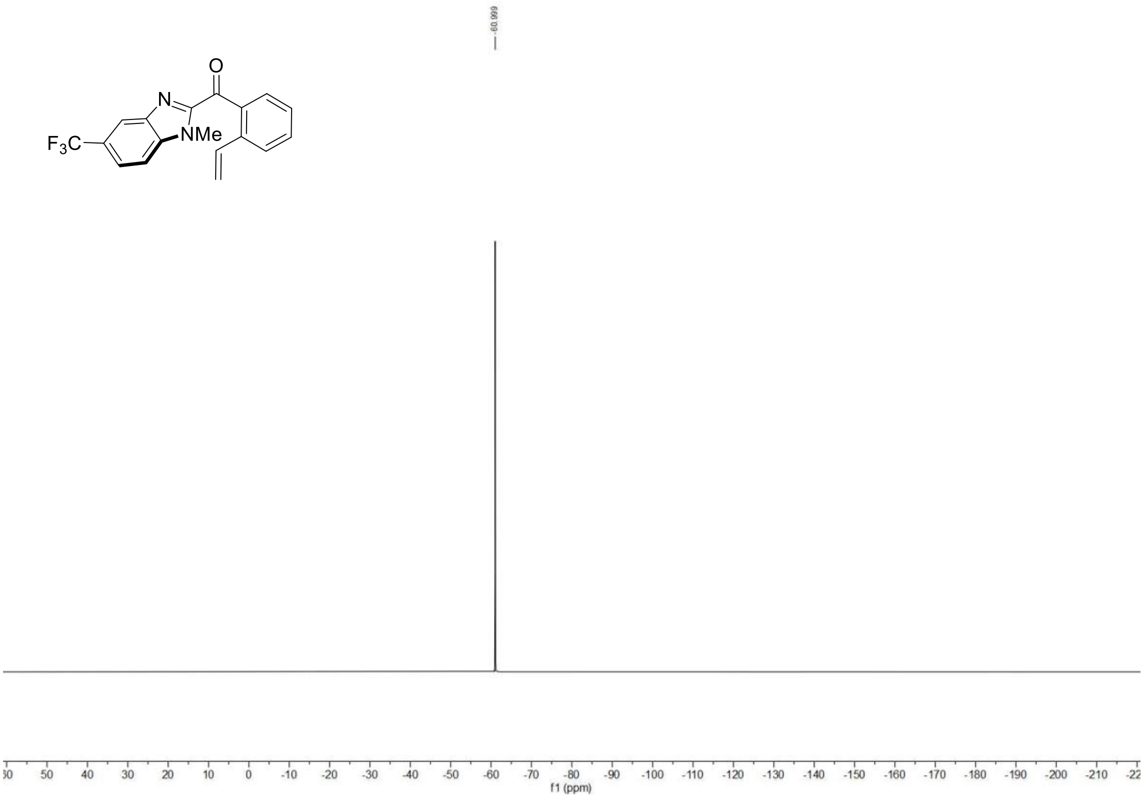

Supplementary Figure 252. ^{19}F-NMR of compound **(1-methyl-5-(trifluoromethyl)-1H-benzo[d]imidazol-2-yl)(2-vinylphenyl)methanone** , recorded at 376 MHz and 25 °C in $CDCl_3$.

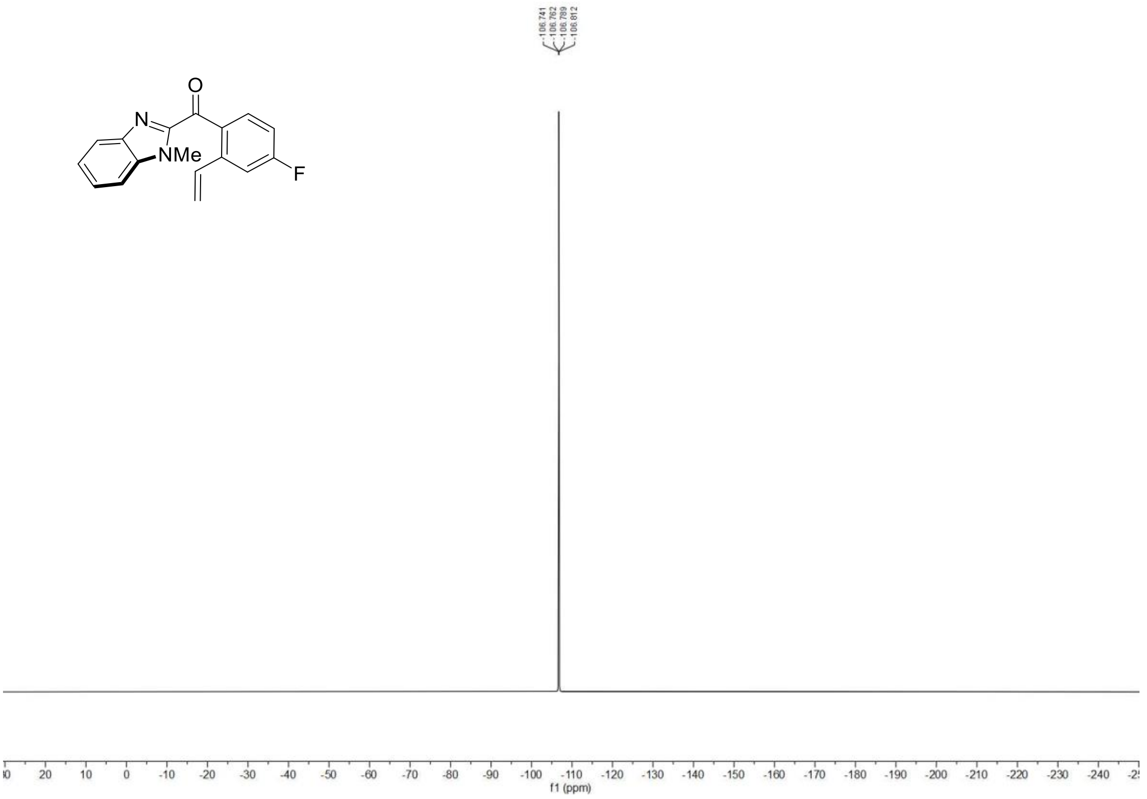

Supplementary Figure 253. ^{19}F-NMR of compound **(4-fluoro-2-vinylphenyl)(1-methyl-1*H*-benzo[*d*]imidazol-2-yl)methanone**, recorded at 376 MHz and 25 °C in $CDCl_3$.

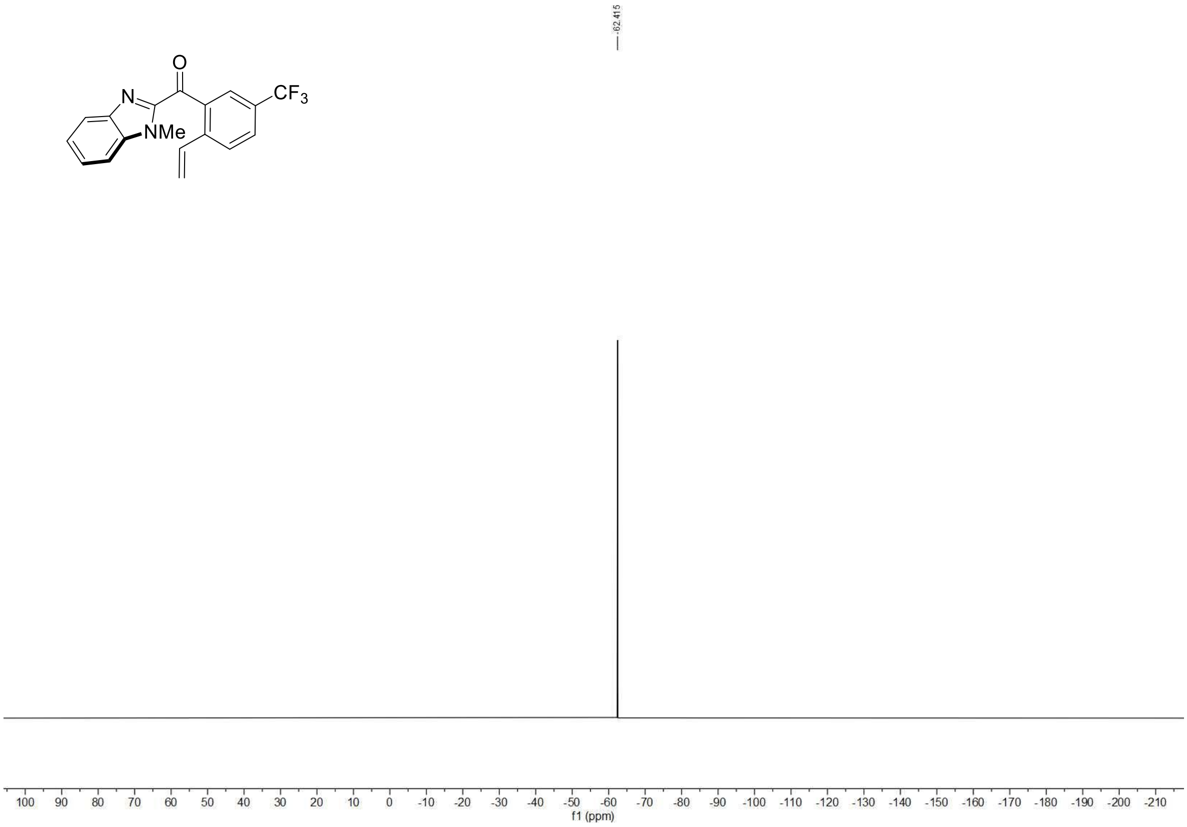

Supplementary Figure 254. ^{19}F-NMR of compound **(1-methyl-1*H*-benzo[*d*]imidazol-2-yl)(5-(trifluoromethyl)-2-vinylphenyl)methanone**, recorded at 376 MHz and 25 °C in $CDCl_3$.

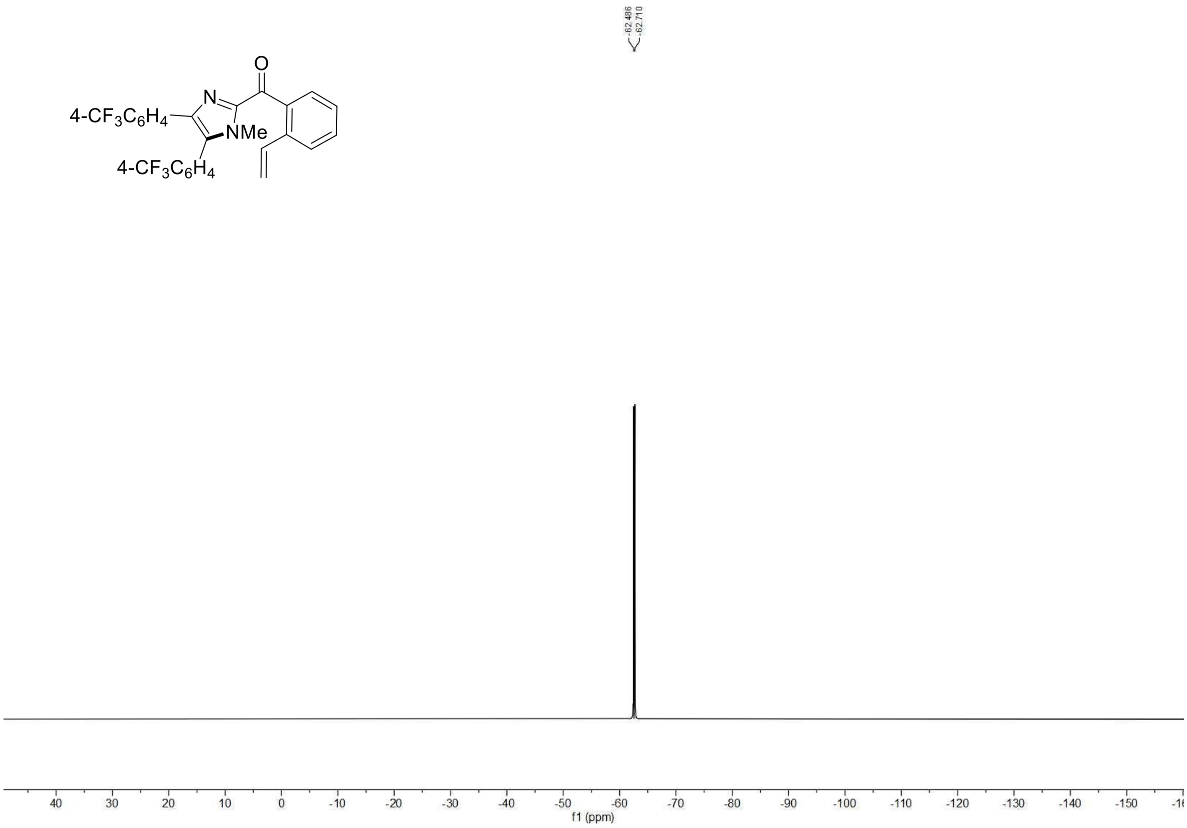

Supplementary Figure 255. ^{19}F-NMR of compound **(1-methyl-4,5-bis(4-(trifluoromethyl)phenyl)-1*H*-imidazol-2-yl)(2-vinylphenyl)methanone**, recorded at 376 MHz and 25 °C in $CDCl_3$.

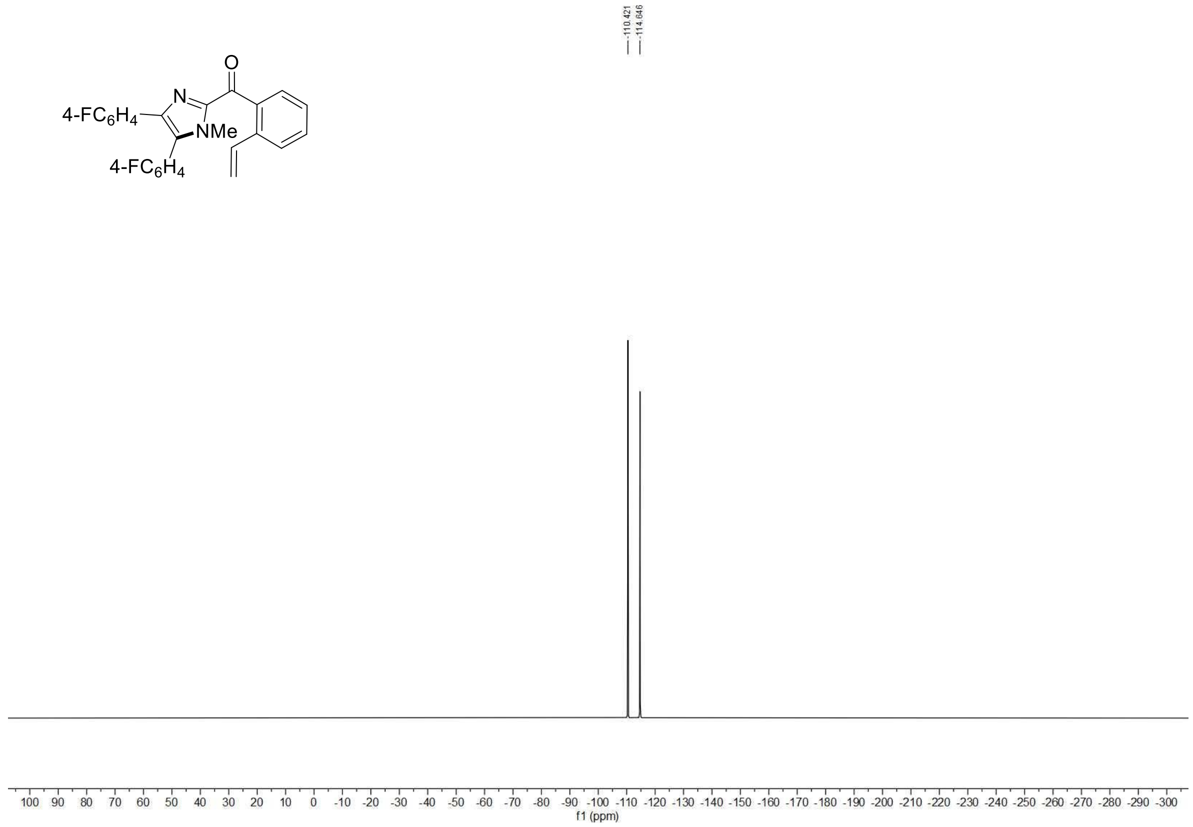

Supplementary Figure 256. ^{19}F-NMR of compound **(4,5-bis(4-fluorophenyl)-1-methyl-1*H*-imidazol-2-yl)(2-vinylphenyl)methanone**, recorded at 376 MHz and 25 °C in $CDCl_3$.

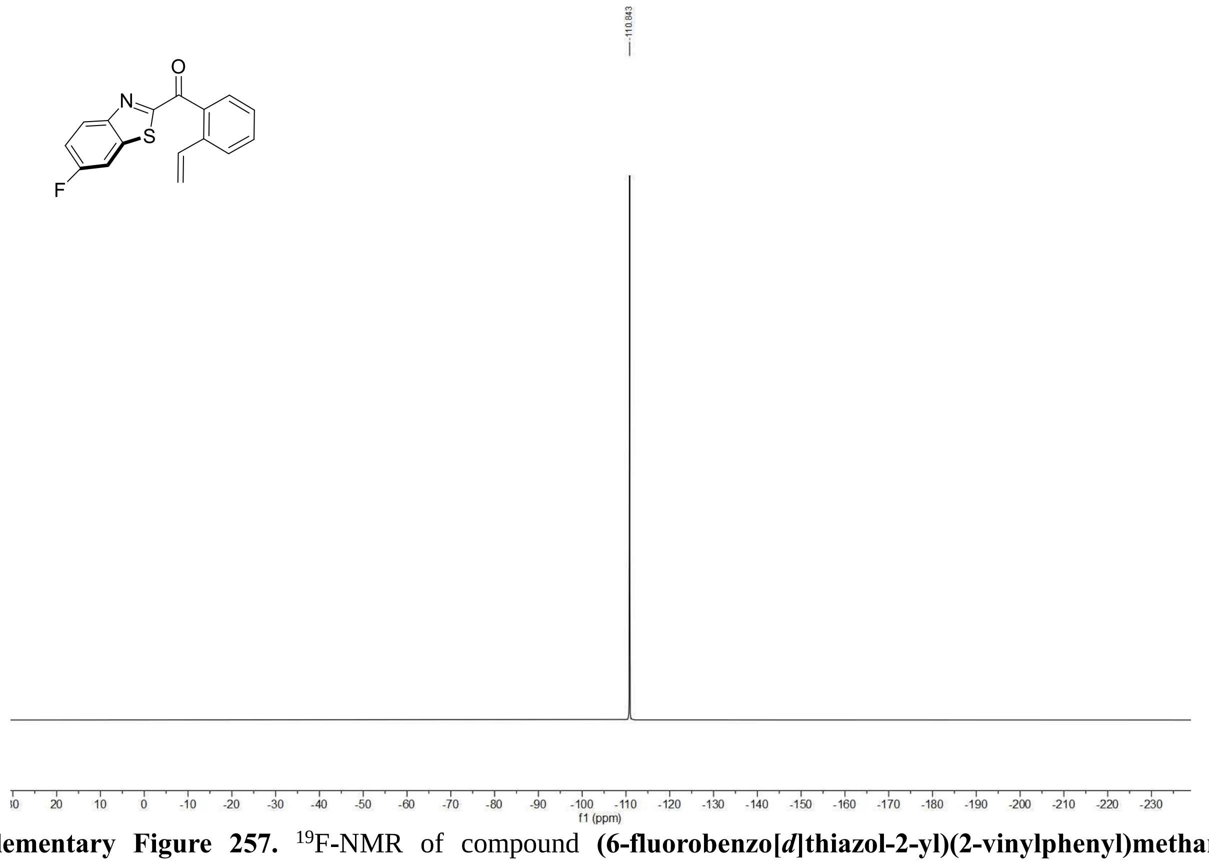

Supplementary Figure 257. ^{19}F-NMR of compound **(6-fluorobenzo[*d*]thiazol-2-yl)(2-vinylphenyl)methanone**, recorded at 376 MHz and 25 °C in $CDCl_3$.

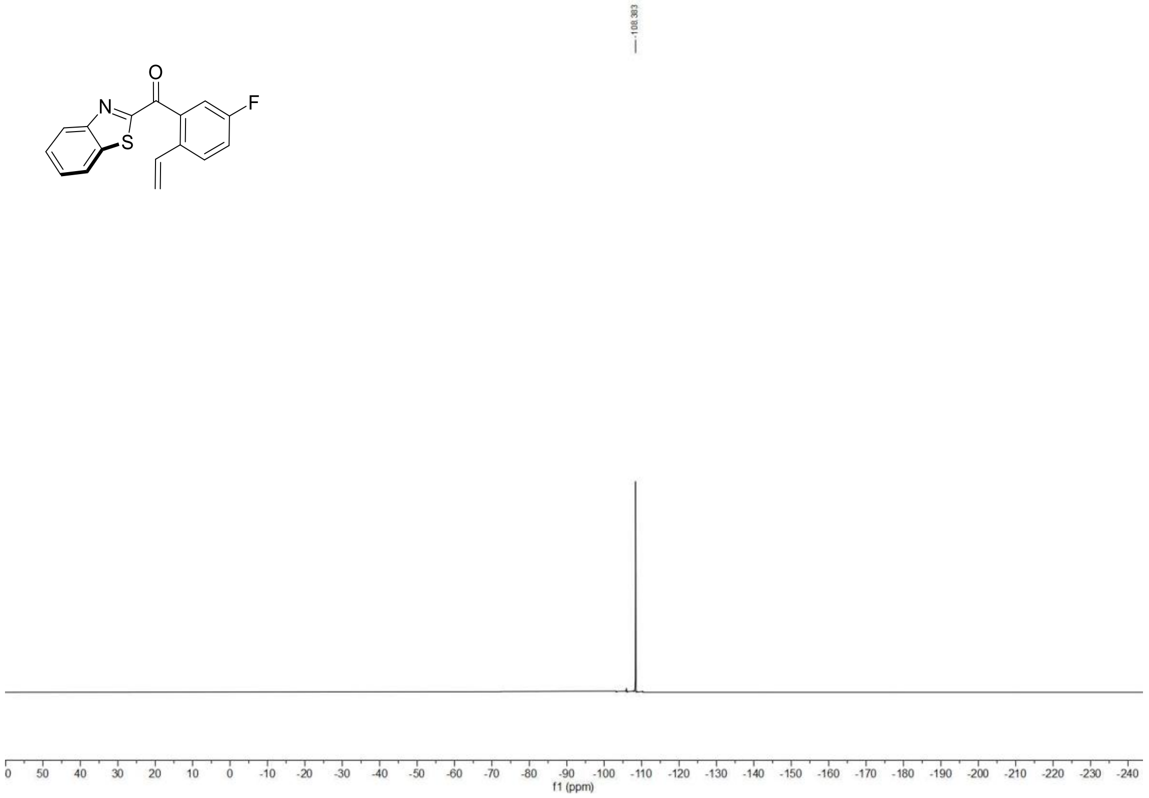

Supplementary Figure 258. ^{19}F-NMR of compound **benzo[*d*]thiazol-2-yl(5-fluoro-2-vinylphenyl)methanone**, recorded at 376 MHz and 25 °C in $CDCl_3$.

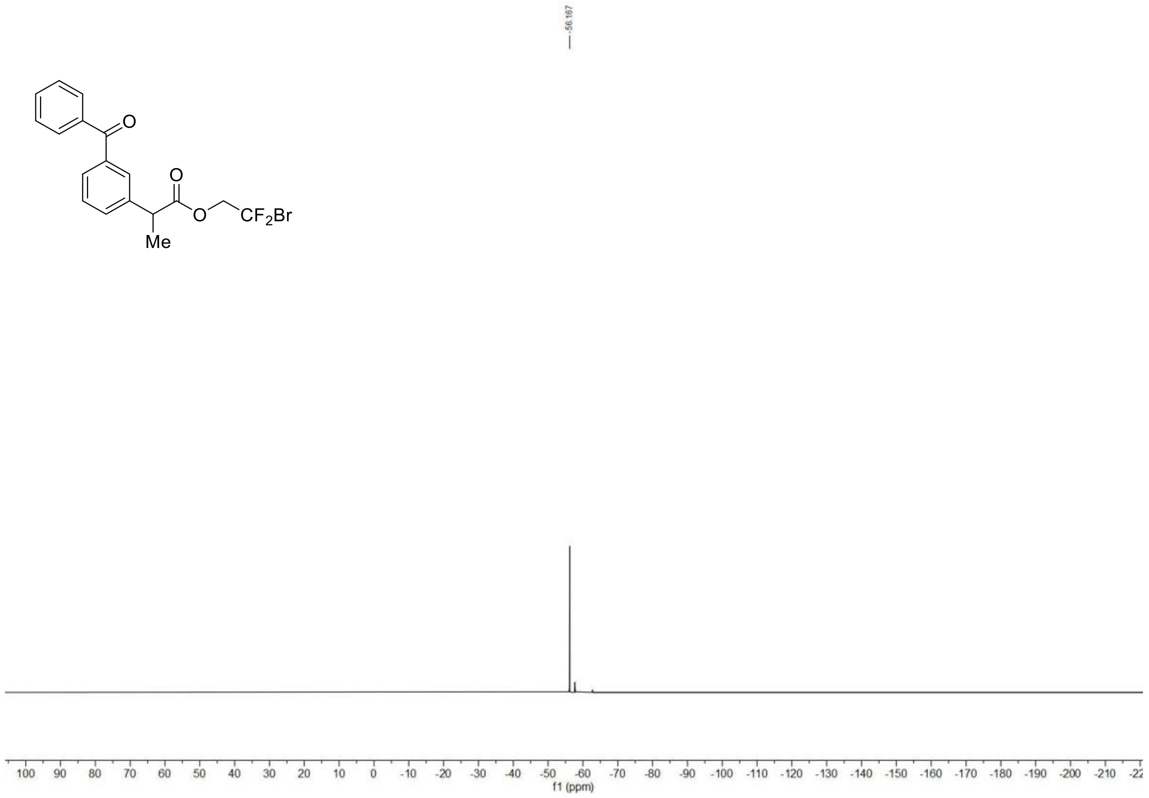

Supplementary Figure 259. ^{19}F-NMR of compound **Difluoroalkyl bromide derived from Ketoprofen**, recorded at 376 MHz and 25 °C in $CDCl_3$.

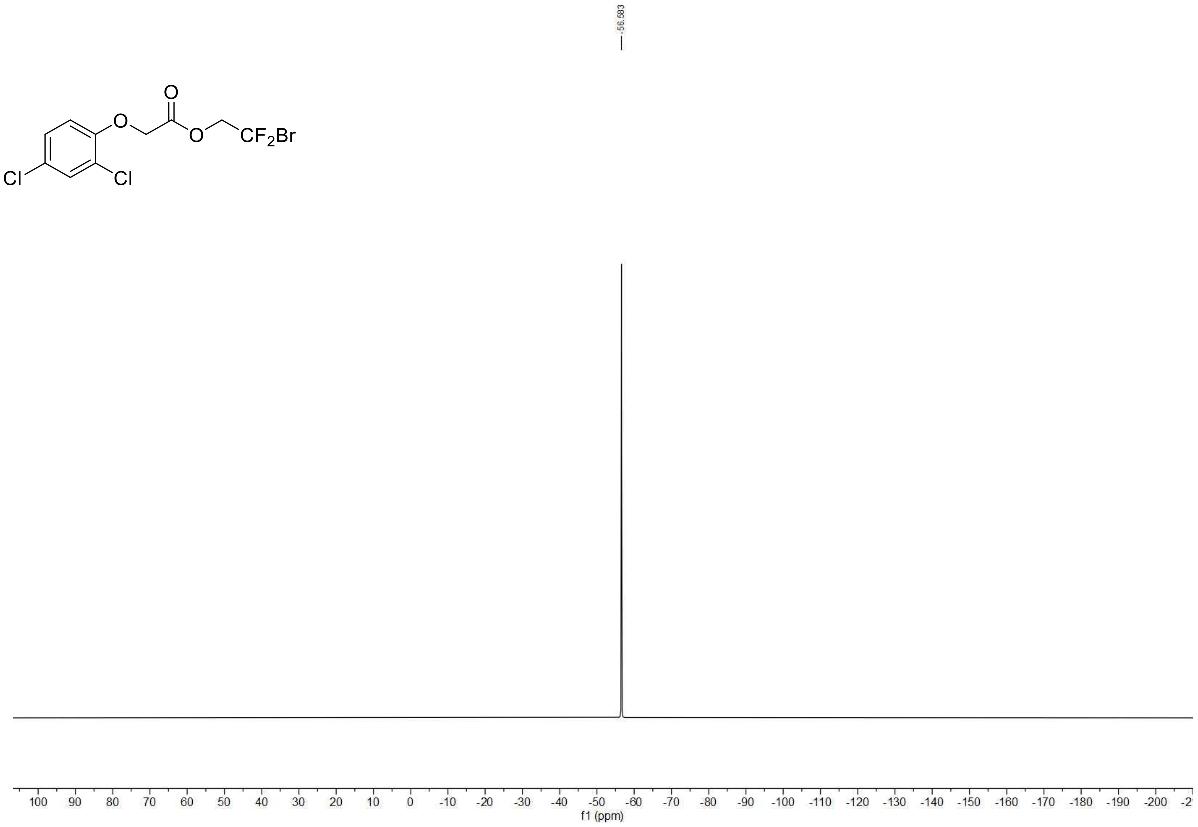

Supplementary Figure 260. ^{19}F-NMR of compound **Difluoroalkyl bromide derived from Fernoxone**, recorded at 376 MHz and 25 °C in $CDCl_3$.

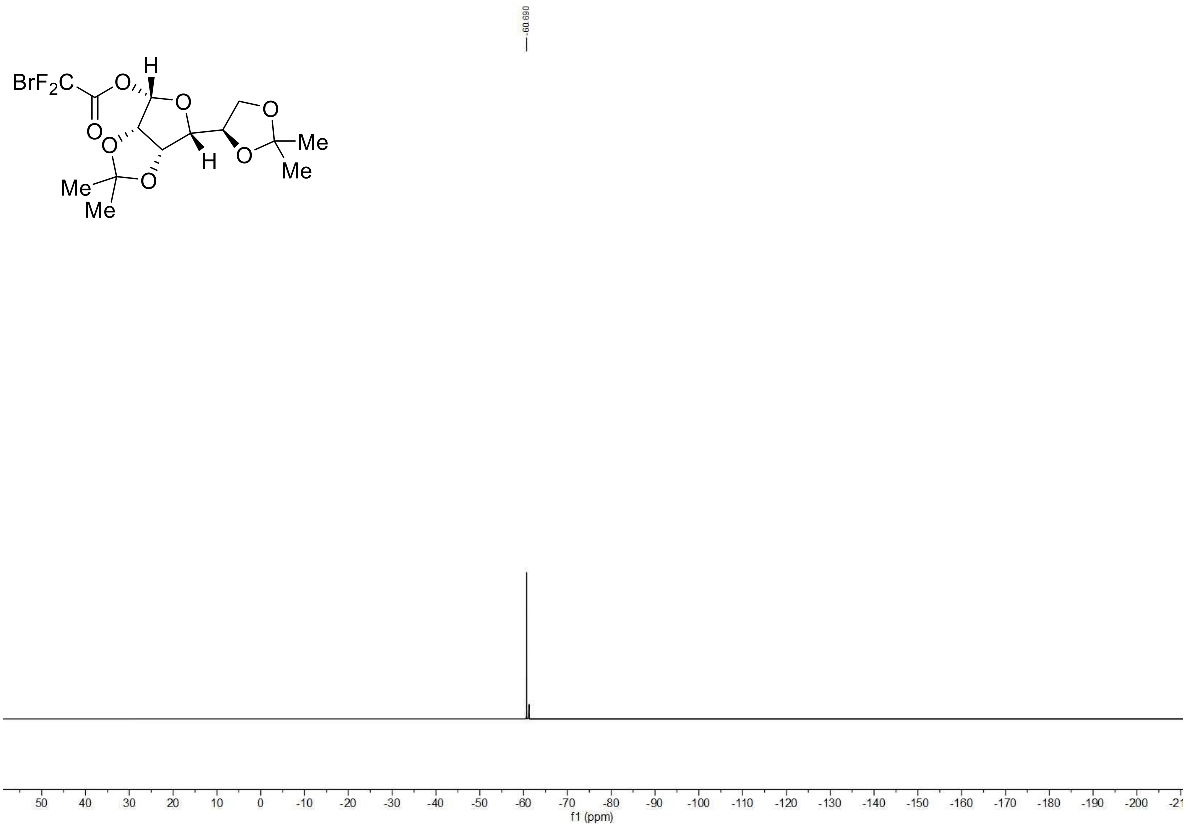

Supplementary Figure 261. ^{19}F-NMR of compound **Difluoroalkyl bromide derived from D-Mannofuranose**, recorded at 376 MHz and 25 °C in $CDCl_3$.

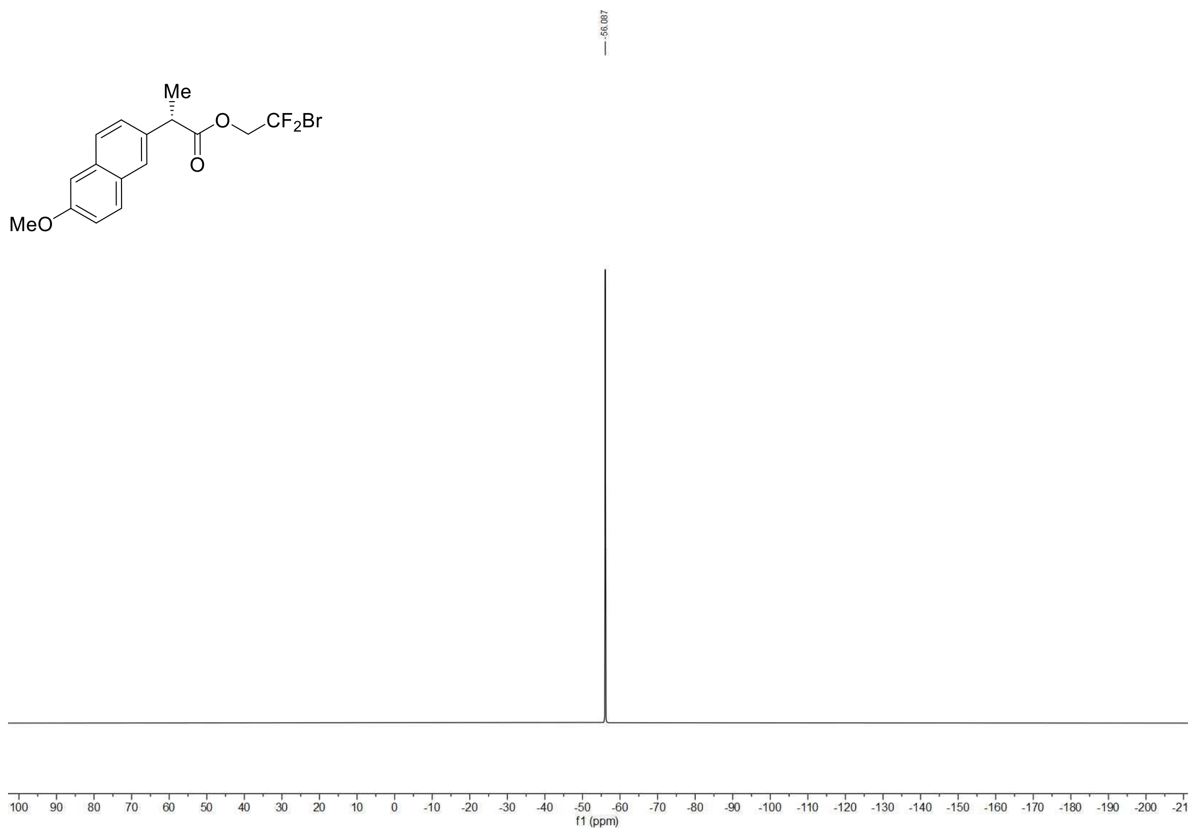

Supplementary Figure 262. ^{19}F-NMR of compound **Difluoroalkyl bromide derived from Naproxen**, recorded at 376 MHz and 25 °C in $CDCl_3$.

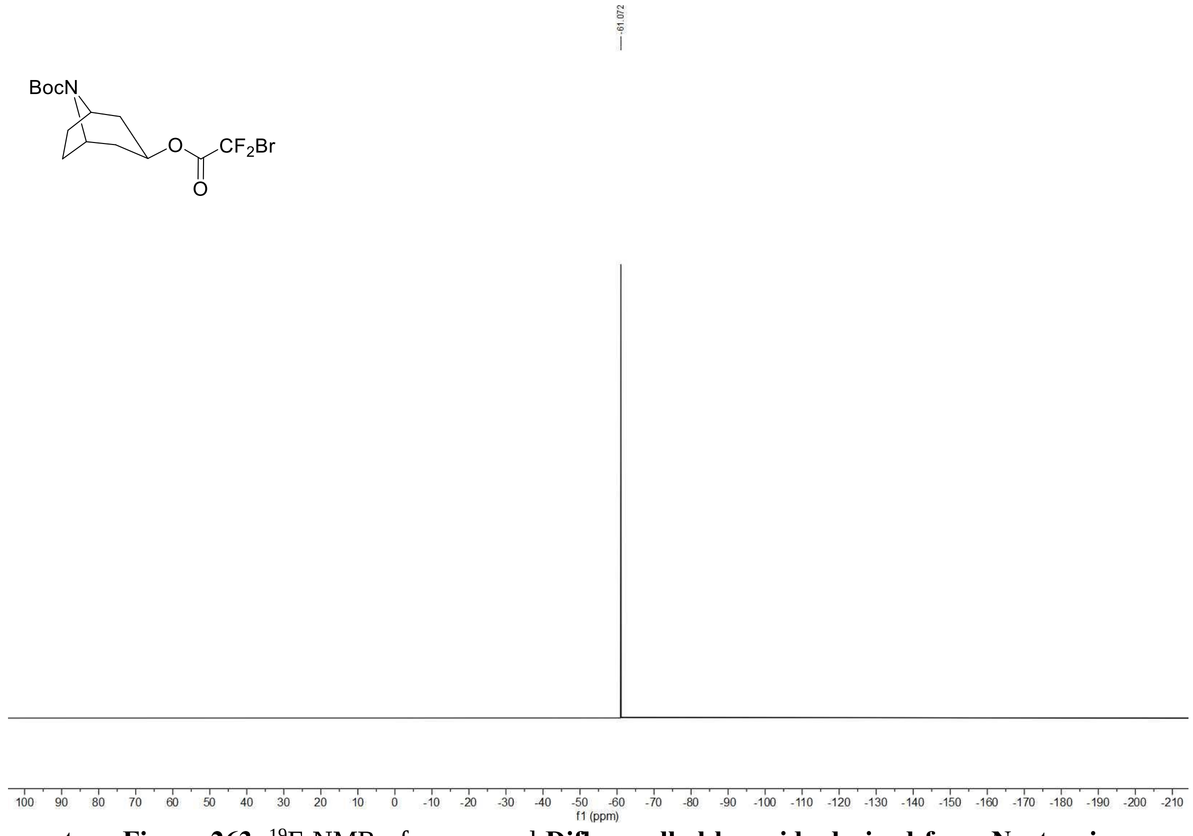

Supplementary Figure 263. ^{19}F-NMR of compound **Difluoroalkyl bromide derived from Nortropine**, recorded at 376 MHz and 25 °C in $CDCl_3$.

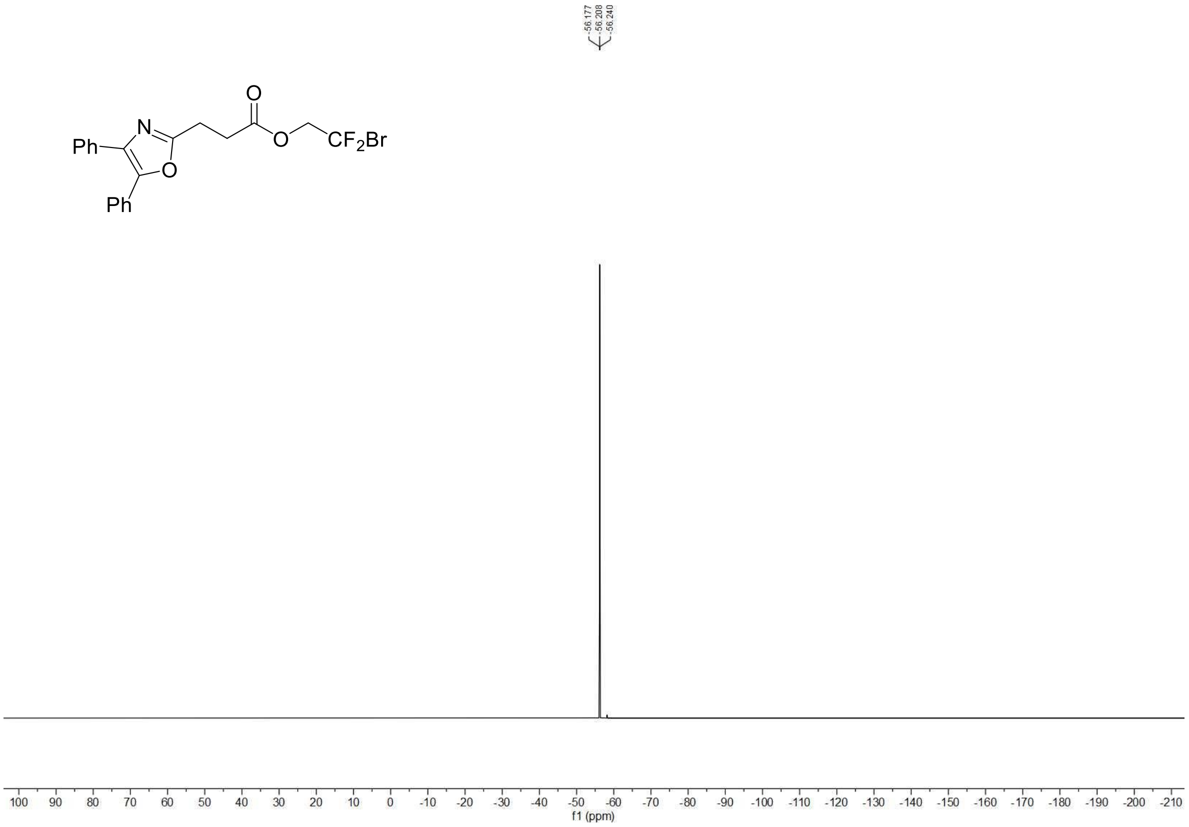

Supplementary Figure 264. ^{19}F-NMR of compound **Difluoroalkyl bromide derived from Oxaprozin**, recorded at 376 MHz and 25 °C in $CDCl_3$.

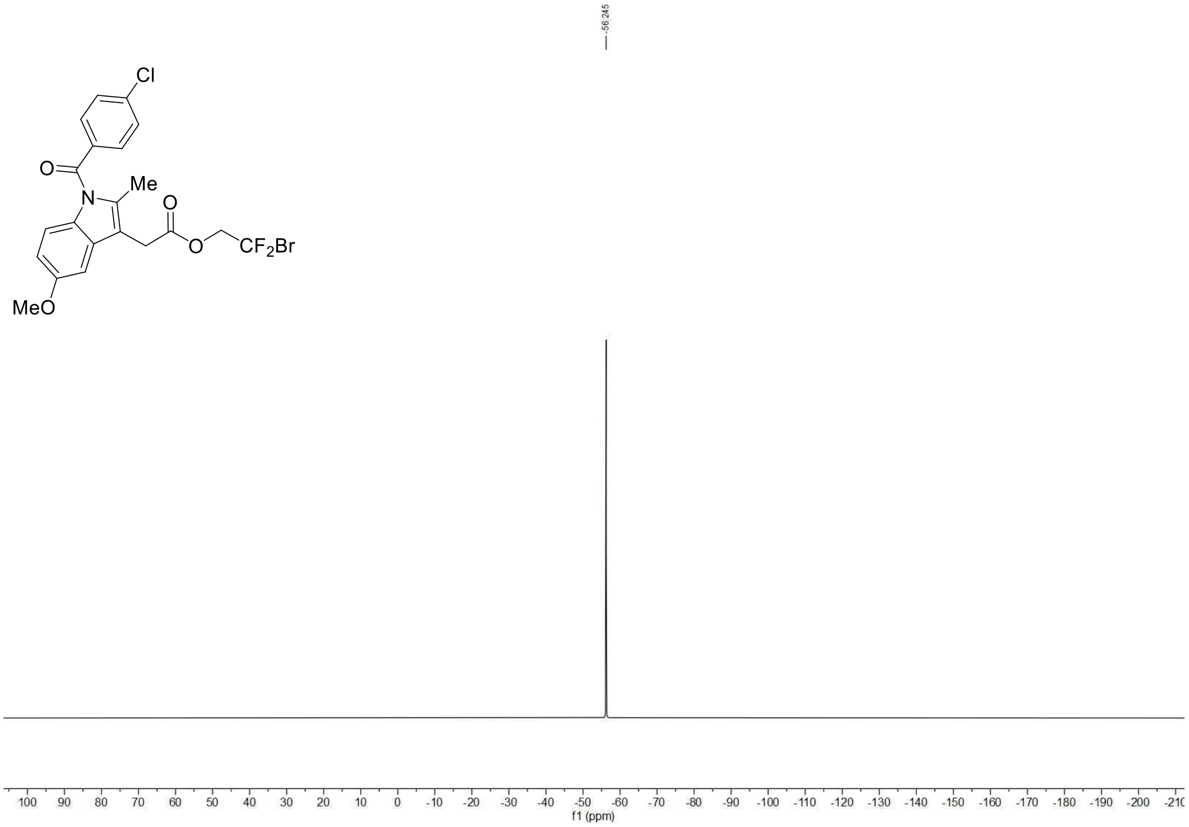

Supplementary Figure 265. ^{19}F-NMR of compound **Difluoroalkyl bromide derived from Indometacin**, recorded at 376 MHz and 25 °C in $CDCl_3$.

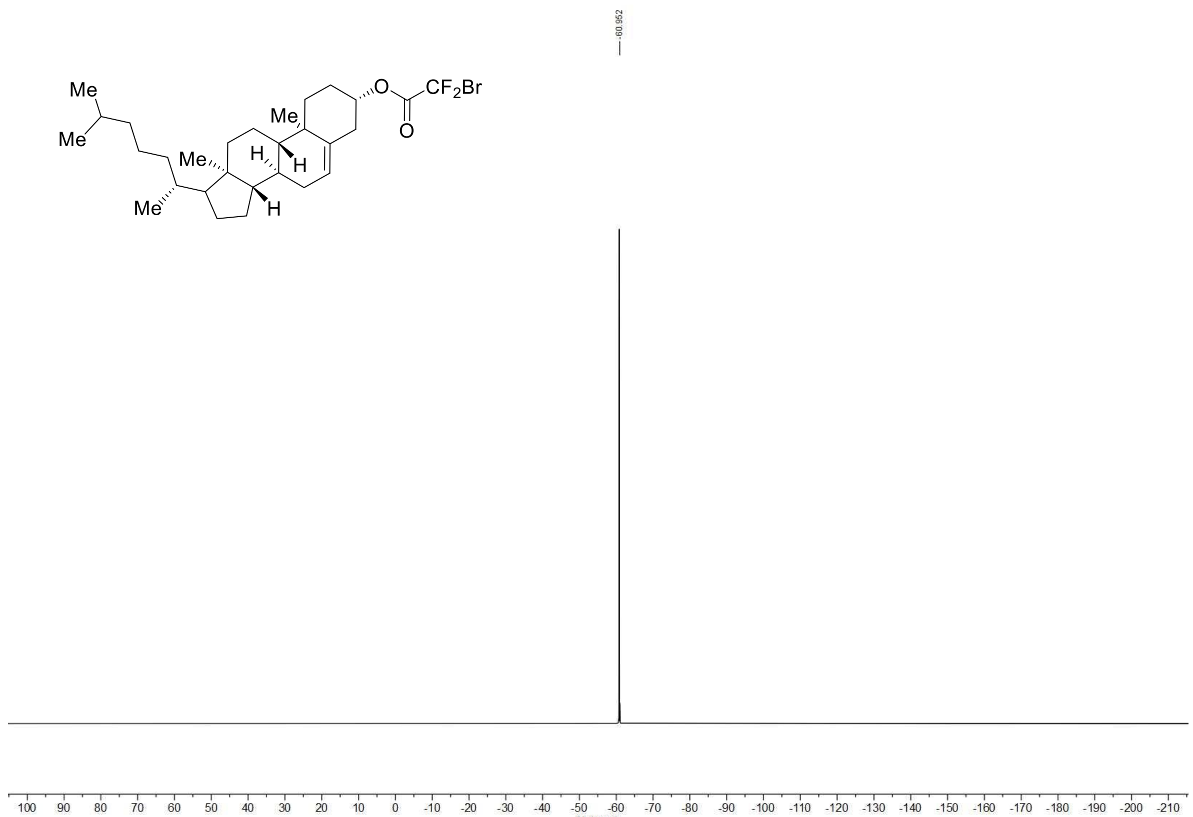

Supplementary Figure 266. ^{19}F-NMR of compound **Difluoroalkyl bromide derived from Cholesterol**, recorded at 376 MHz and 25 °C in $CDCl_3$.

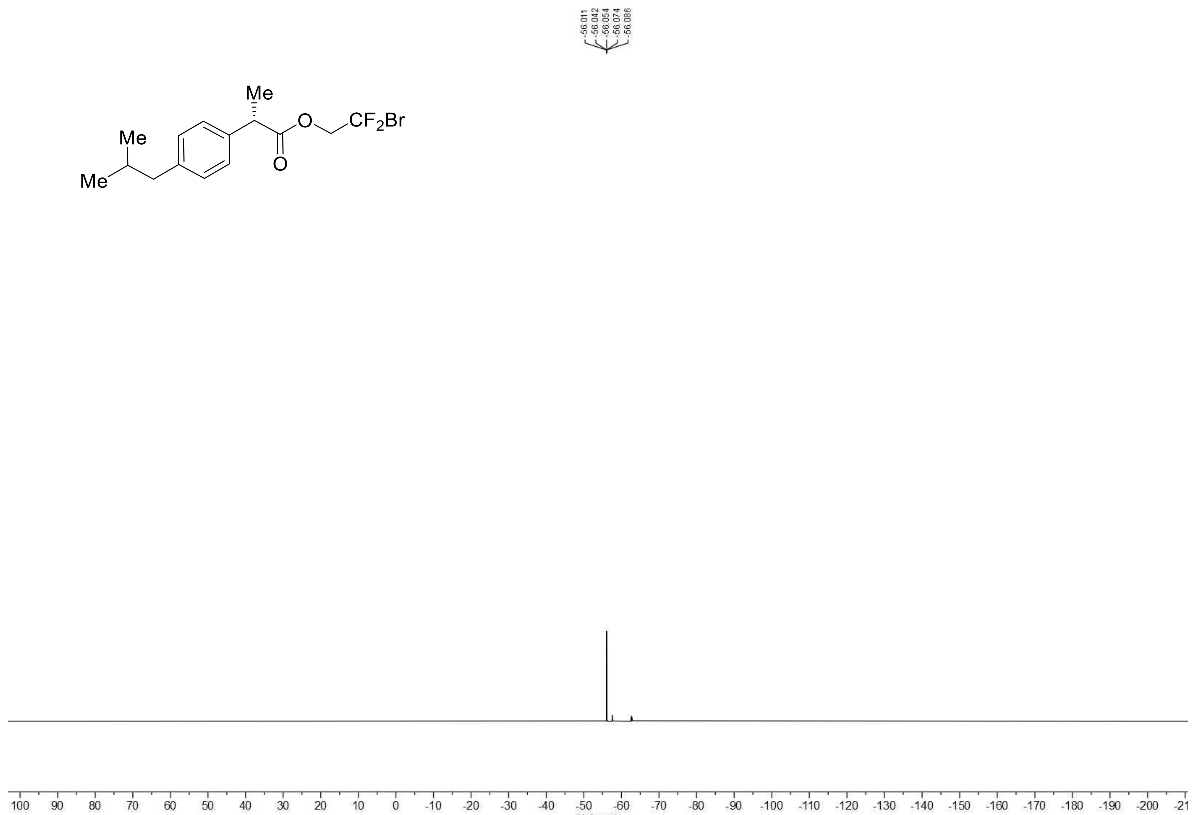

Supplementary Figure 267. ^{19}F-NMR of compound **Difluoroalkyl bromide derived from Ibuprofen**, recorded at 376 MHz and 25 °C in $CDCl_3$.

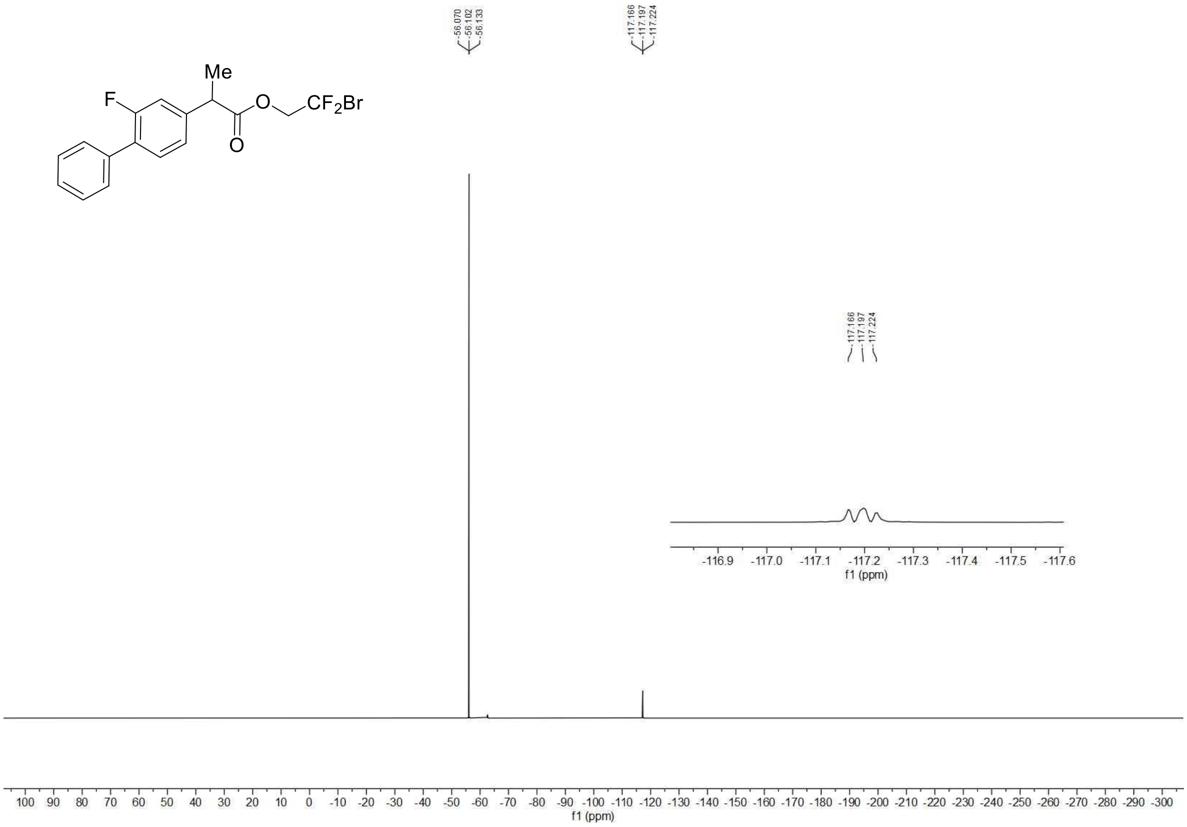

Supplementary Figure 268. ^{19}F-NMR of compound **Difluoroalkyl bromide derived from Flurbiprofen**, recorded at 376 MHz and 25 °C in $CDCl_3$.

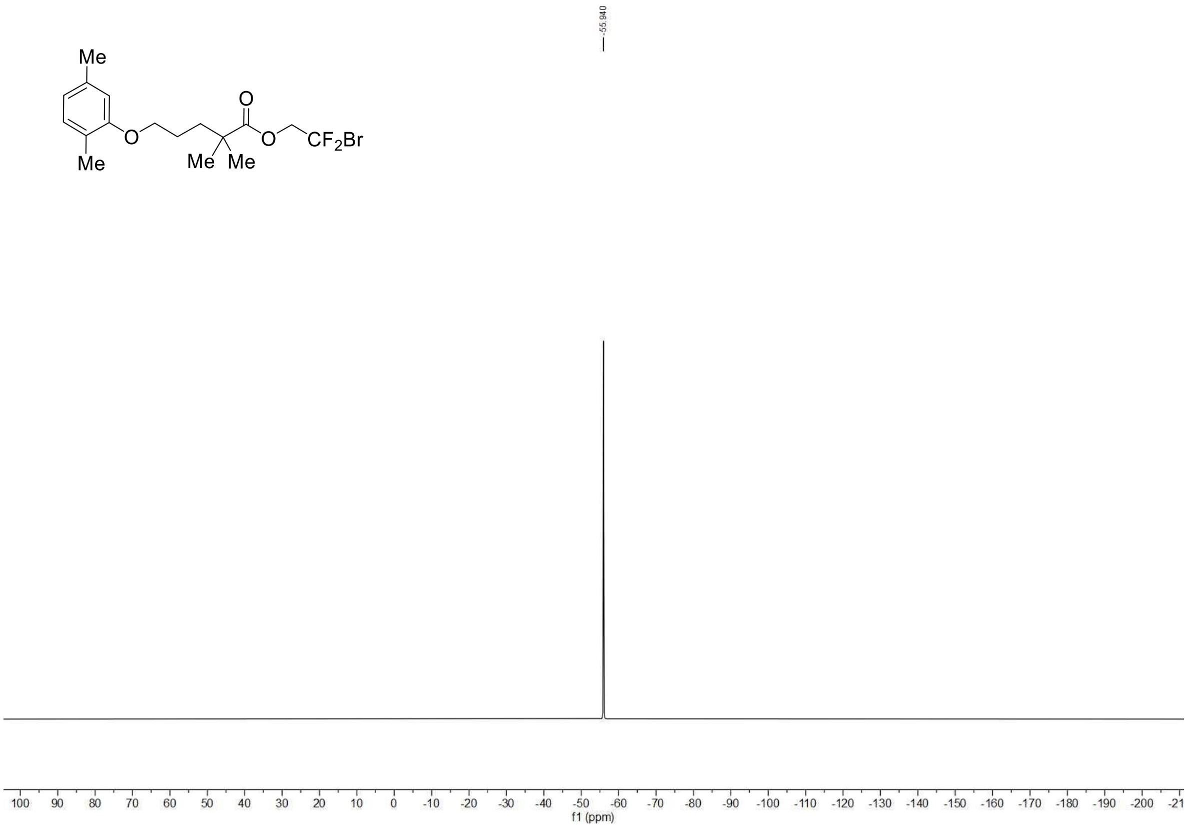

Supplementary Figure 269. ^{19}F-NMR of compound **Difluoroalkyl bromide derived from Gemfibrozil**, recorded at 376 MHz and 25 °C in $CDCl_3$.

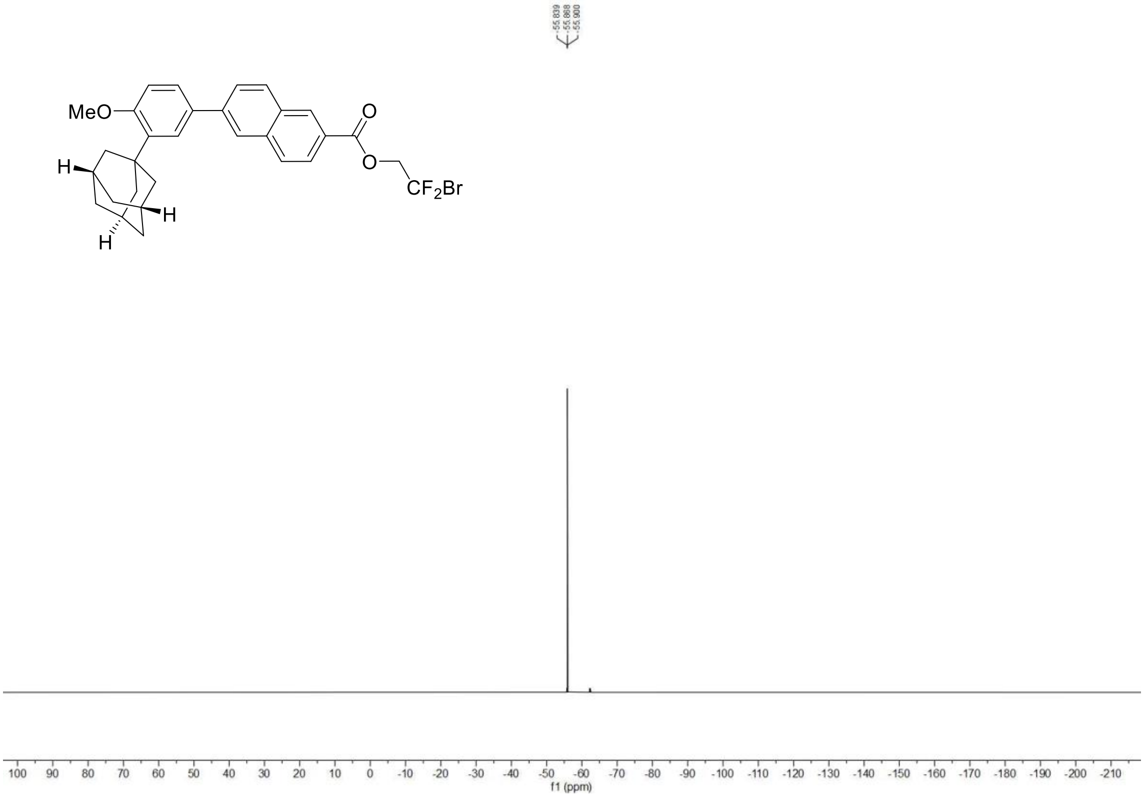

Supplementary Figure 270. ^{19}F-NMR of compound **Difluoroalkyl bromide derived from Adapalene**, recorded at 376 MHz and 25 °C in $CDCl_3$.

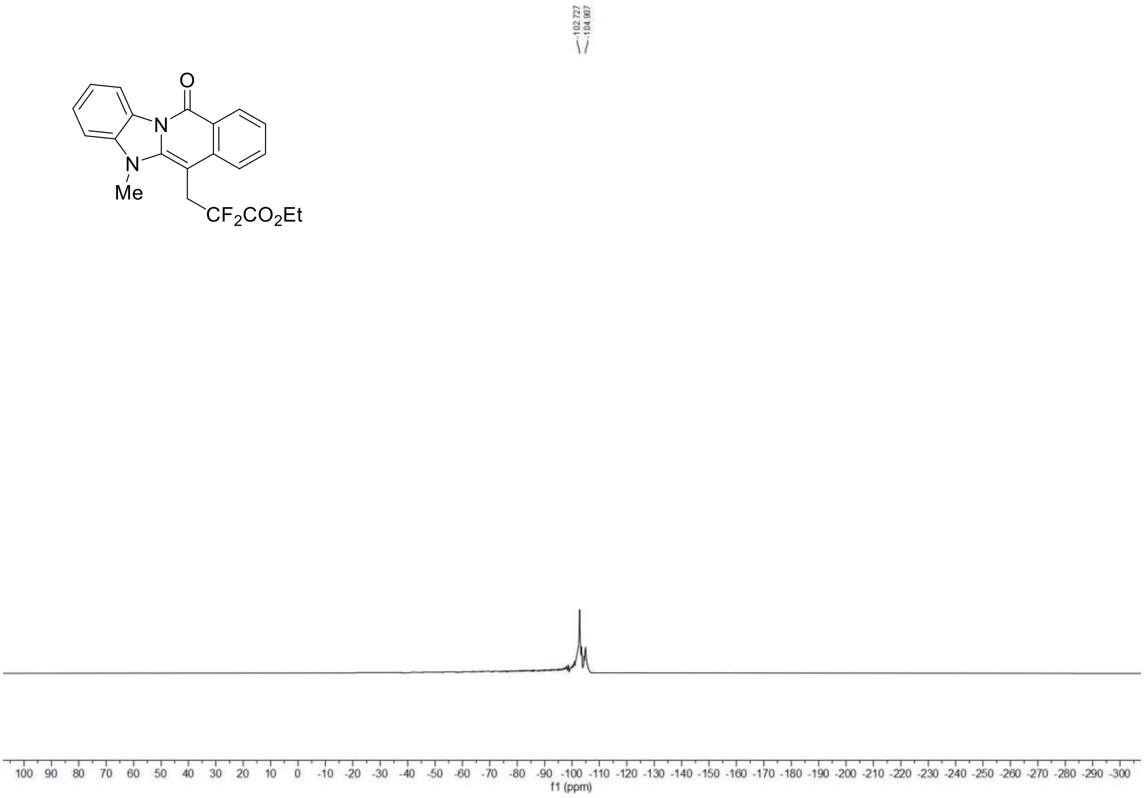

Supplementary Figure 271. ^{19}F-NMR of compound **2**, recorded at 376 MHz and 50 °C in $CDCl_3$.

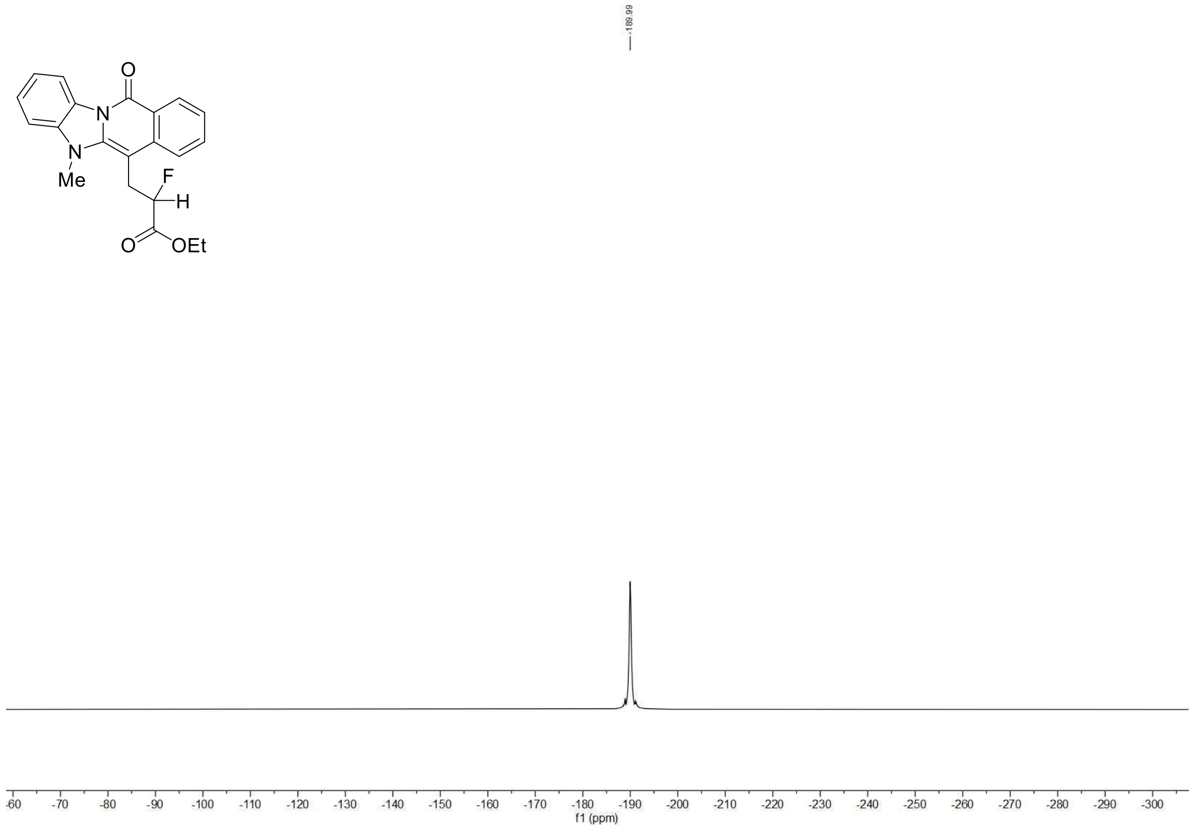

Supplementary Figure 272. ^{19}F-NMR of compound **13**, recorded at 376 MHz and 50 °C in $CDCl_3$.

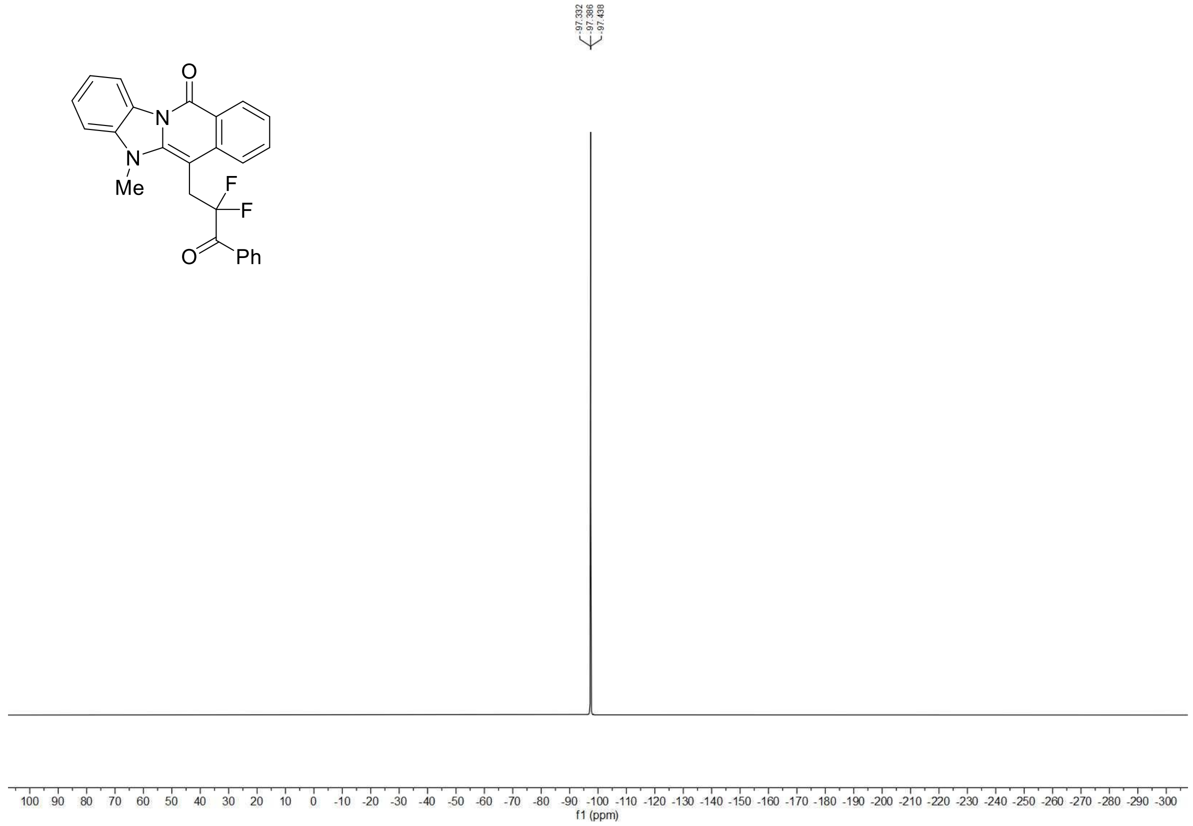

Supplementary Figure 273. ^{19}F-NMR of compound **14**, recorded at 376 MHz and 50 °C in $CDCl_3$.

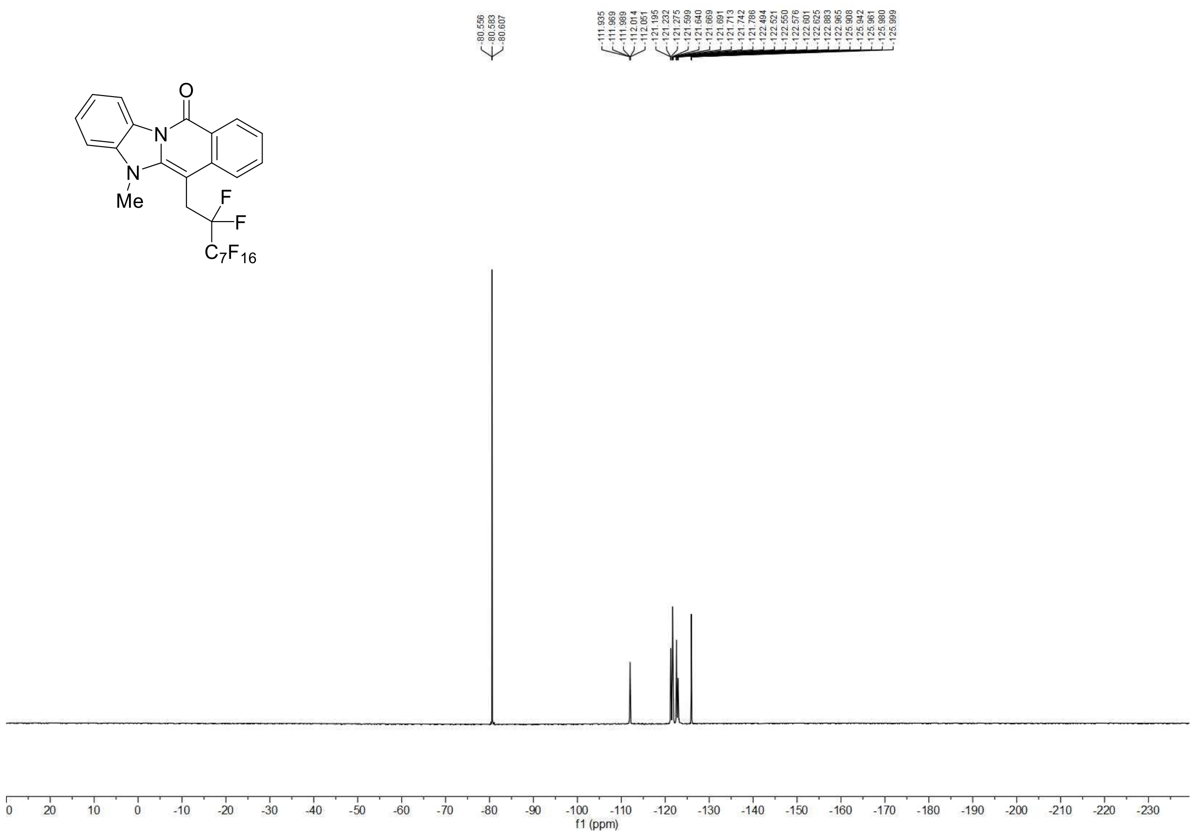

Supplementary Figure 274. ^{19}F-NMR of compound **15**, recorded at 376 MHz and 50 °C in $CDCl_3$.

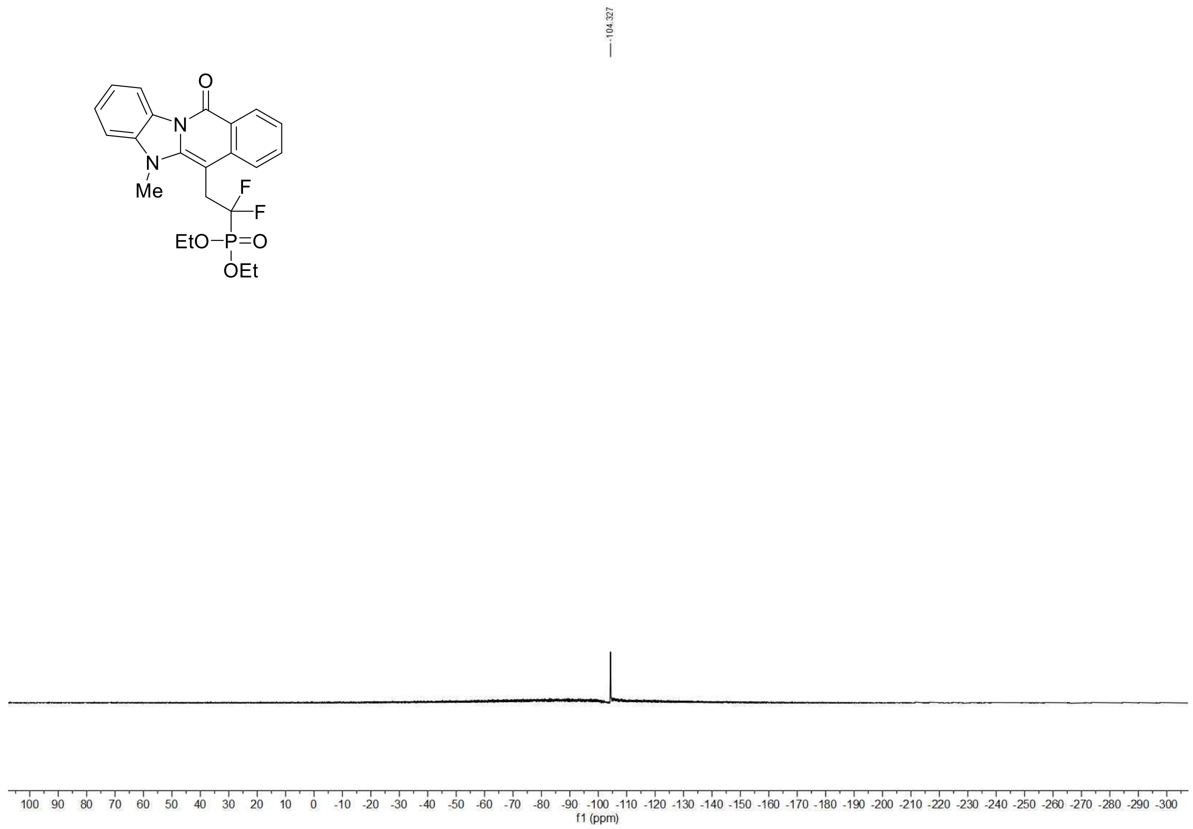

Supplementary Figure 275. ^{19}F-NMR of compound **16**, recorded at 376 MHz and 50 °C in $CDCl_3$.

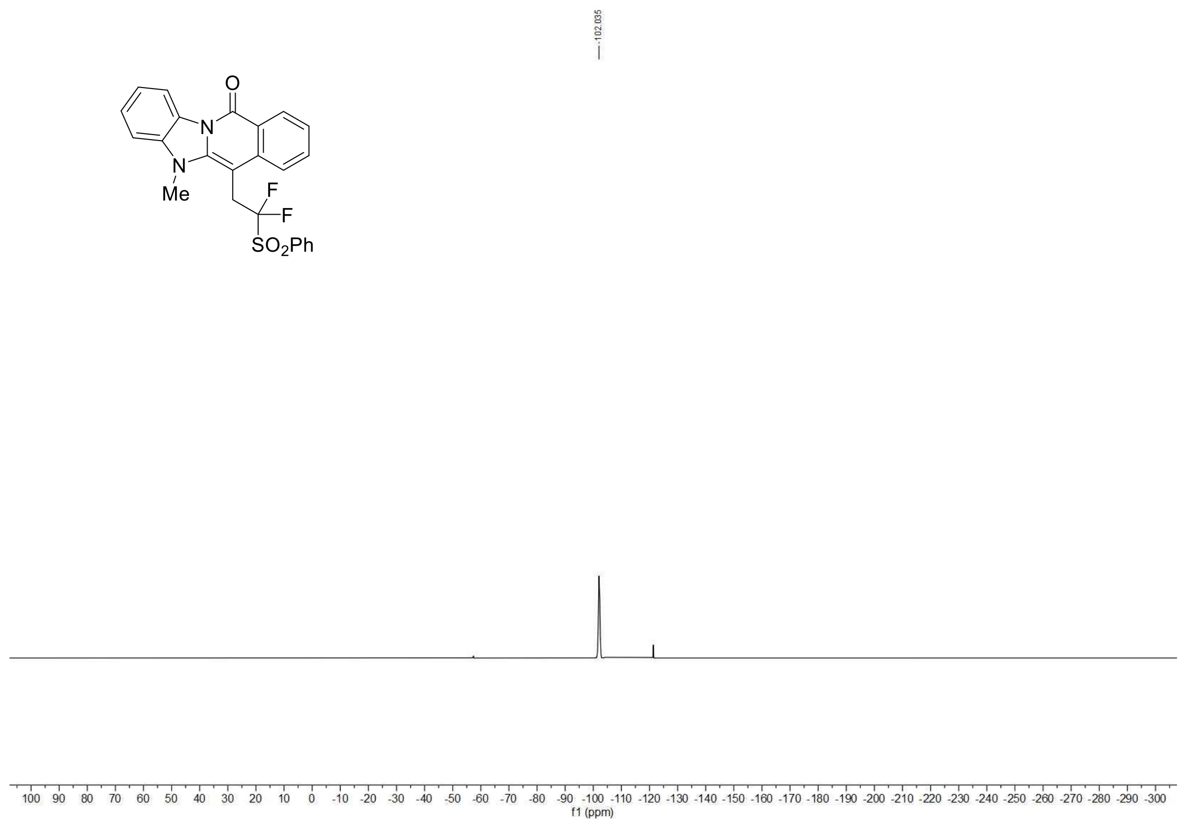

Supplementary Figure 276. ^{19}F-NMR of compound **17**, recorded at 376 MHz and 50 °C in $CDCl_3$.

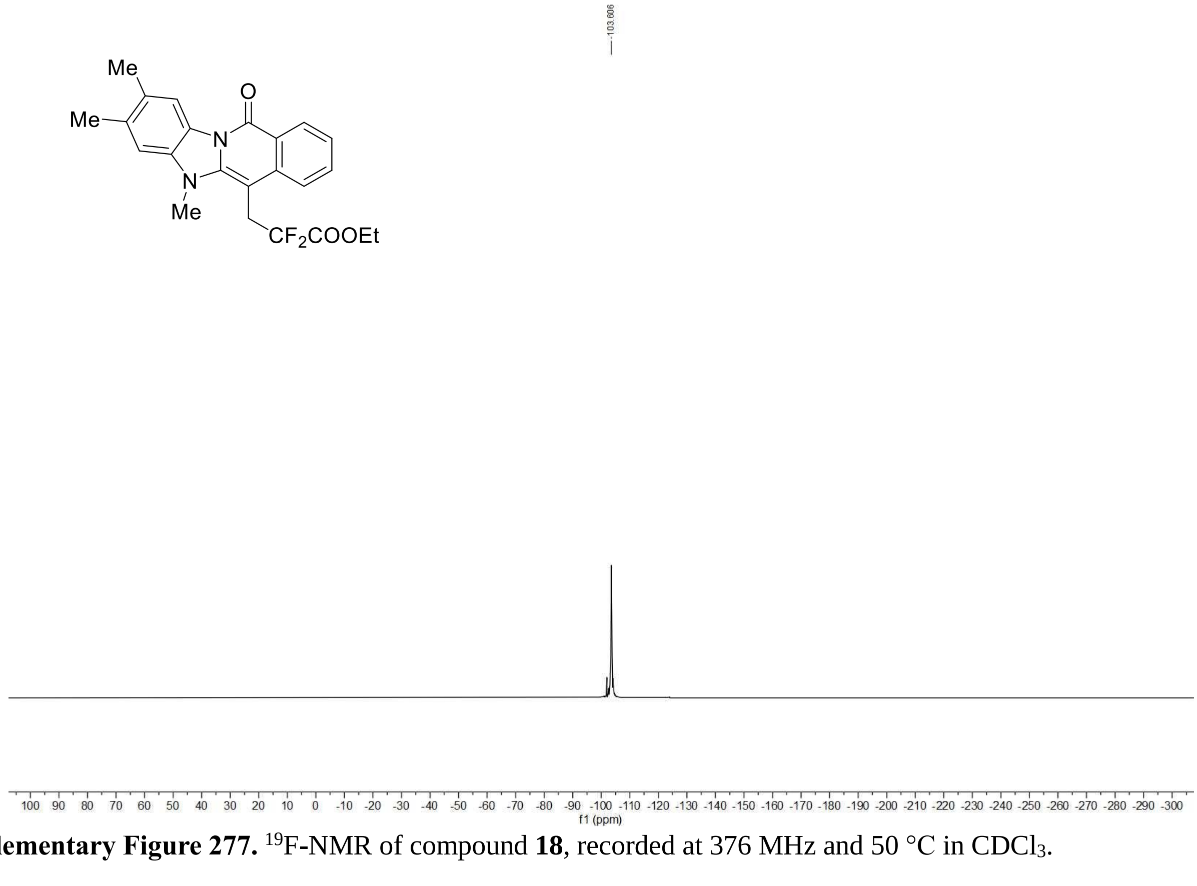

Supplementary Figure 277. ^{19}F-NMR of compound **18**, recorded at 376 MHz and 50 °C in $CDCl_3$.

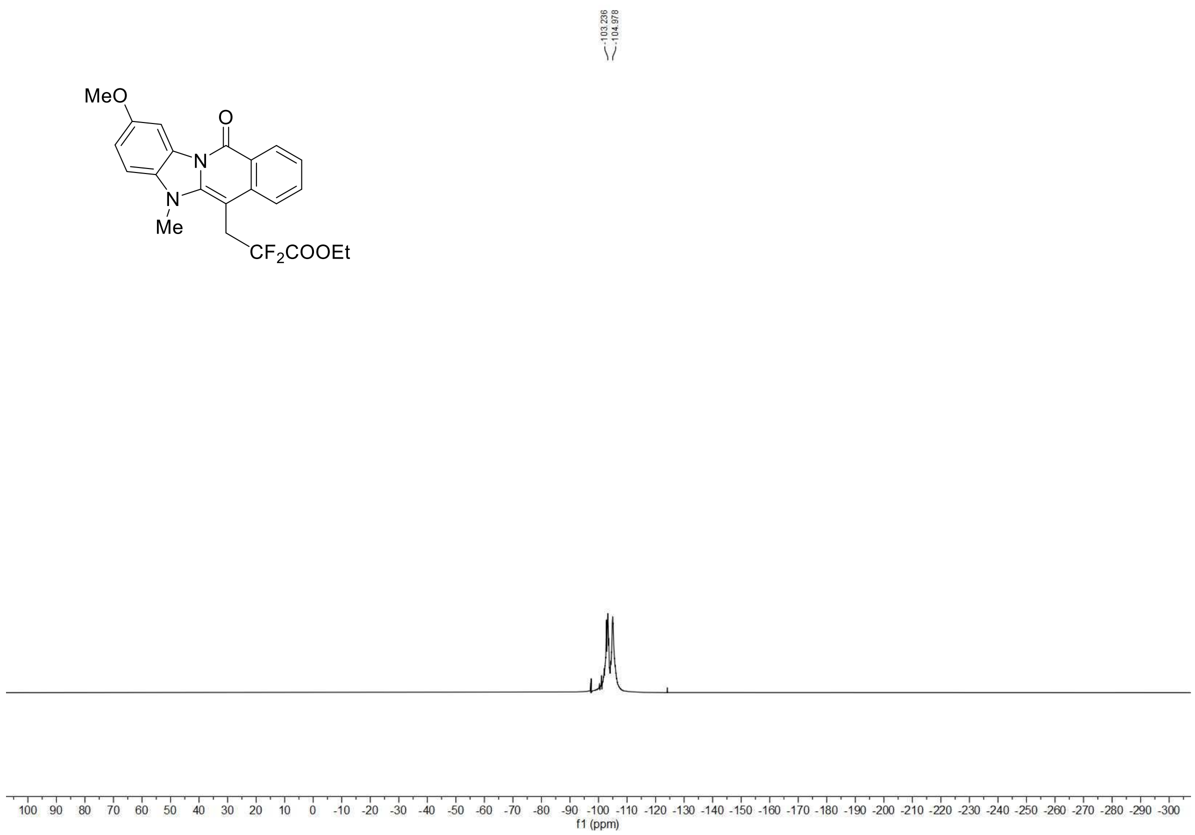

Supplementary Figure 278. ^{19}F-NMR of compound **19**, recorded at 376 MHz and 50 °C in $CDCl_3$.

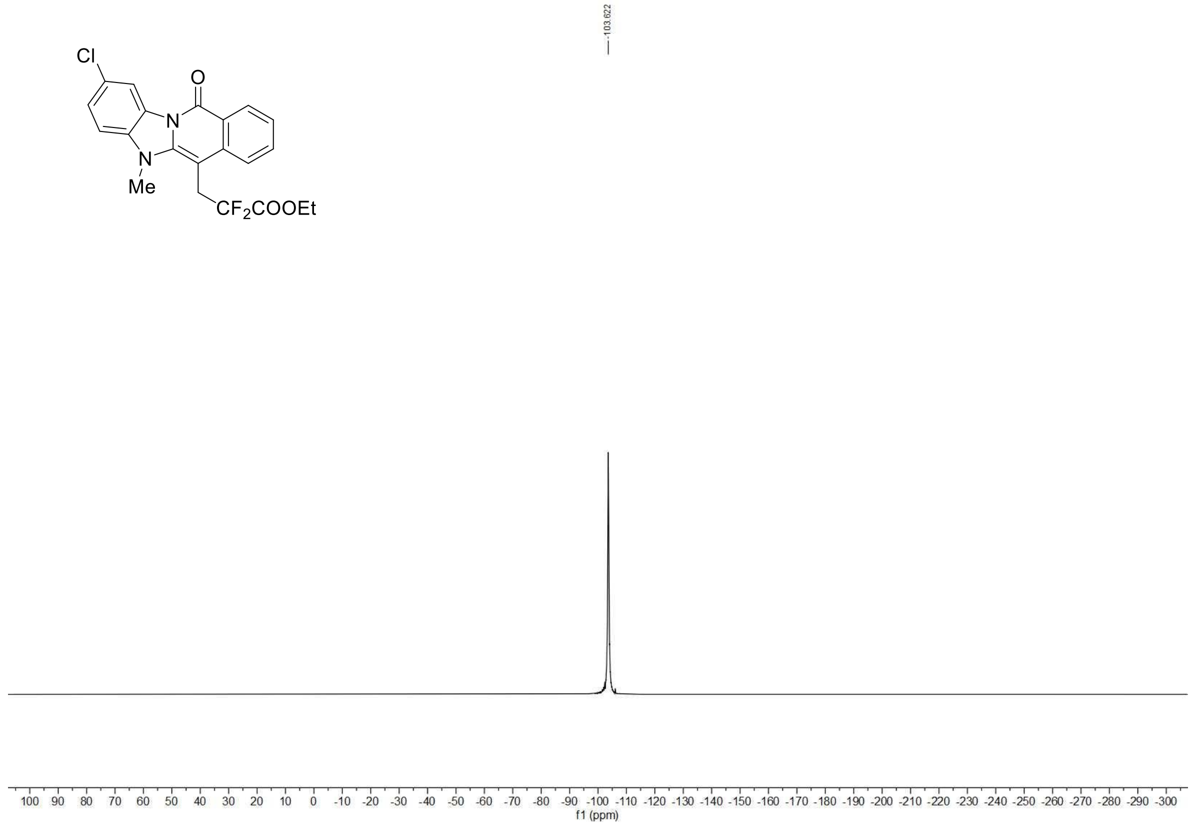

Supplementary Figure 279. ^{19}F-NMR of compound **20**, recorded at 376 MHz and 50 °C in $CDCl_3$.

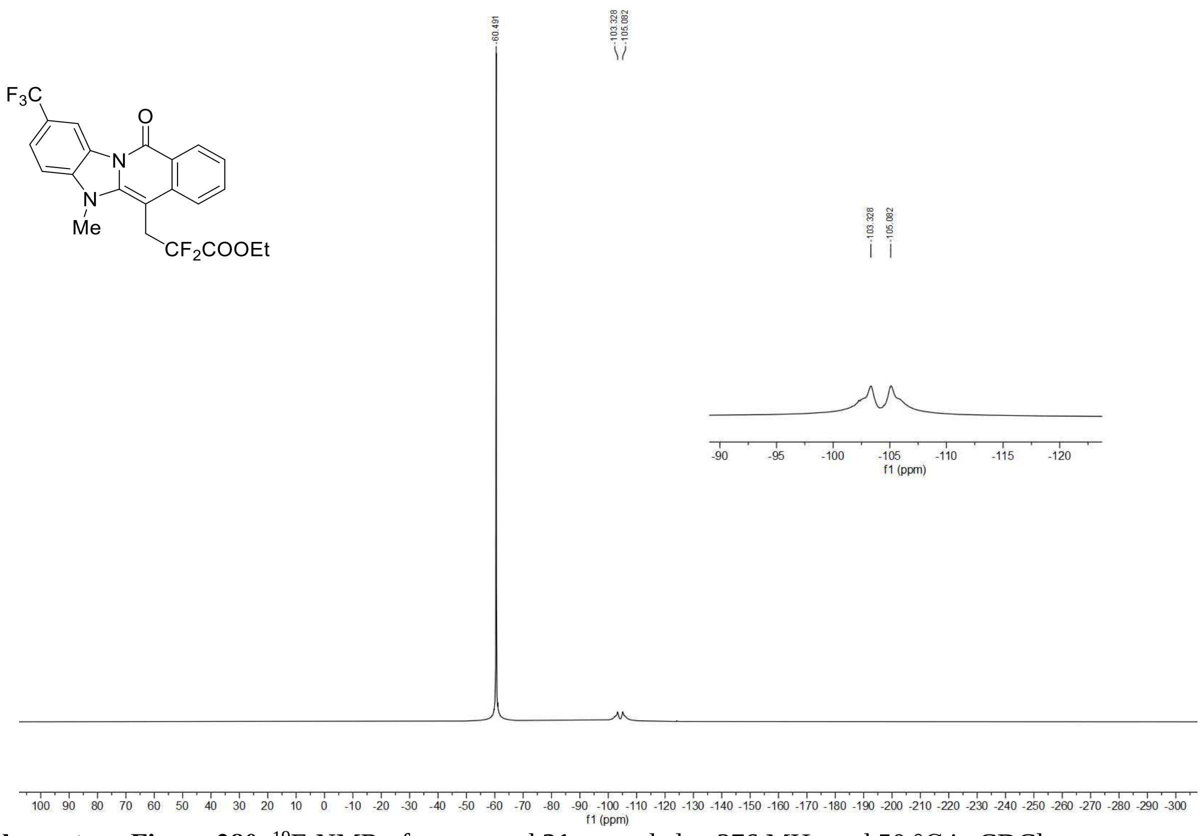

Supplementary Figure 280. ^{19}F-NMR of compound **21**, recorded at 376 MHz and 50 °C in $CDCl_3$.

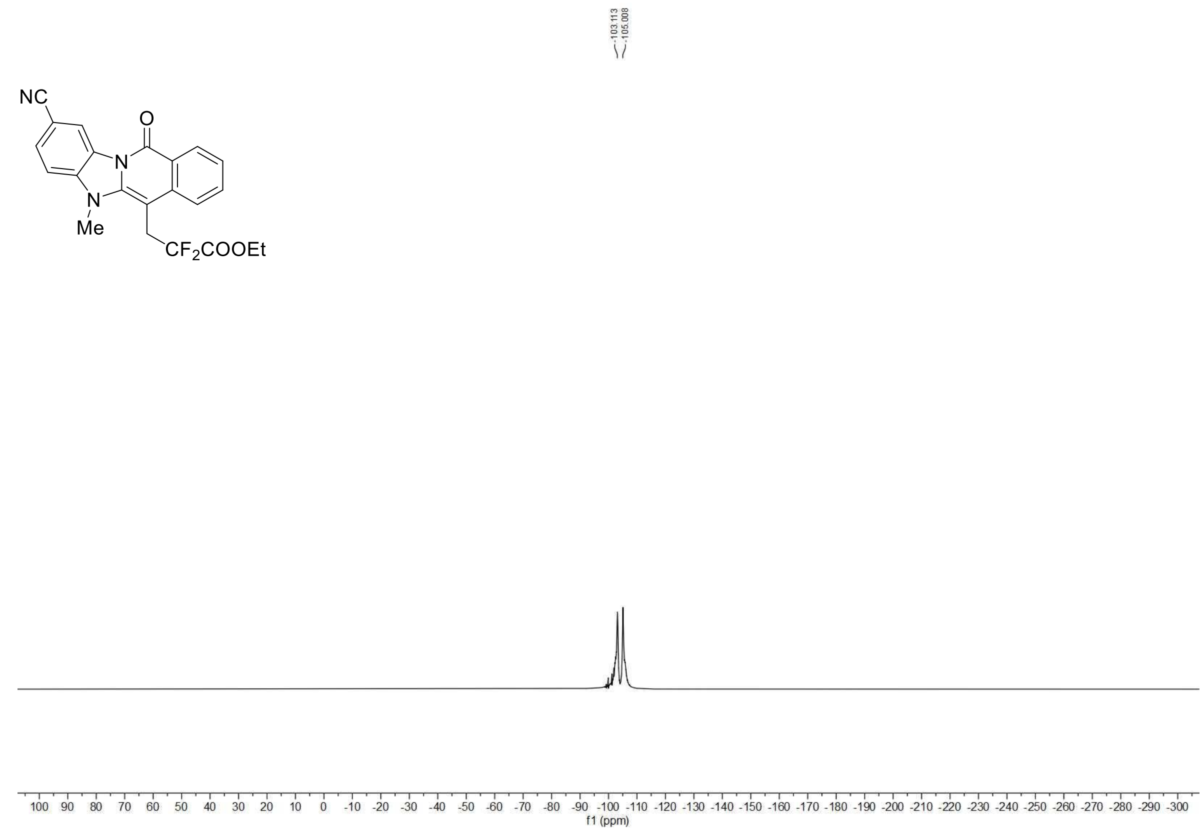

Supplementary Figure 281. ^{19}F-NMR of compound **22**, recorded at 376 MHz and 50 °C in $CDCl_3$.

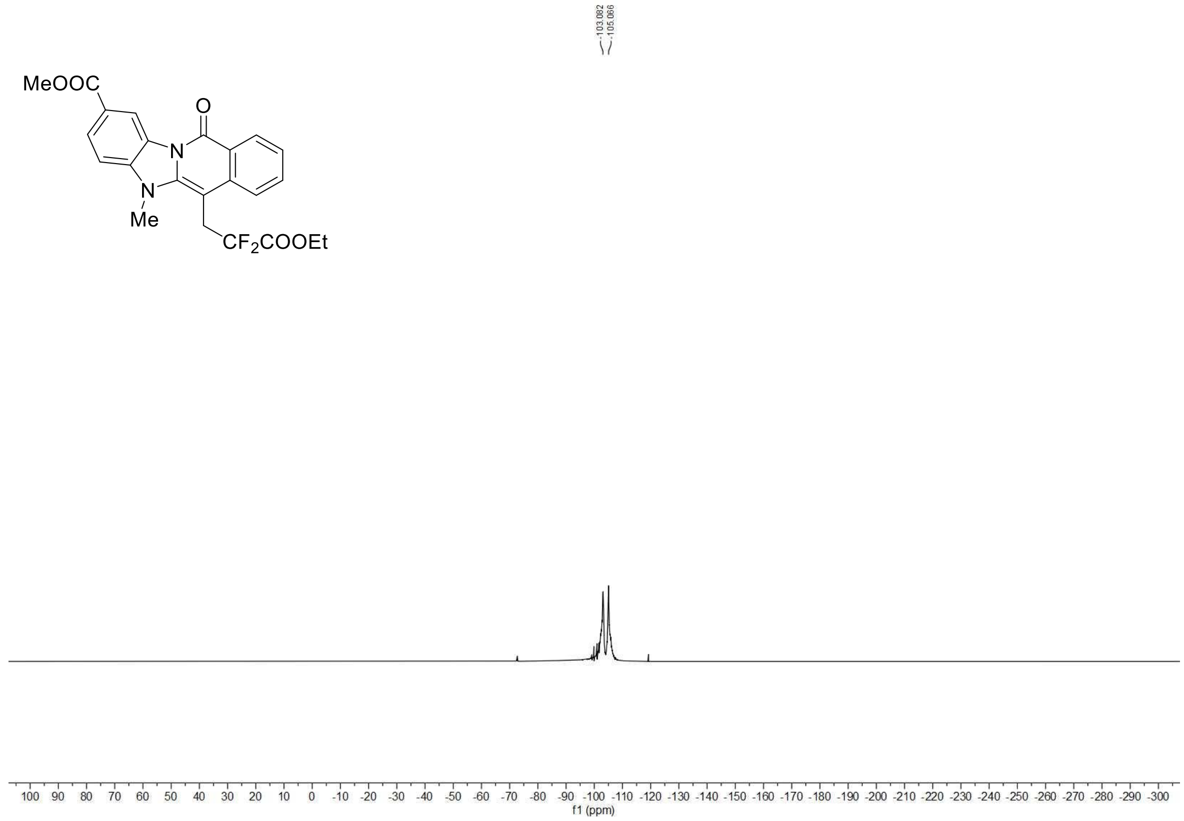

Supplementary Figure 282. ^{19}F-NMR of compound **23**, recorded at 376 MHz and 50 °C in $CDCl_3$.

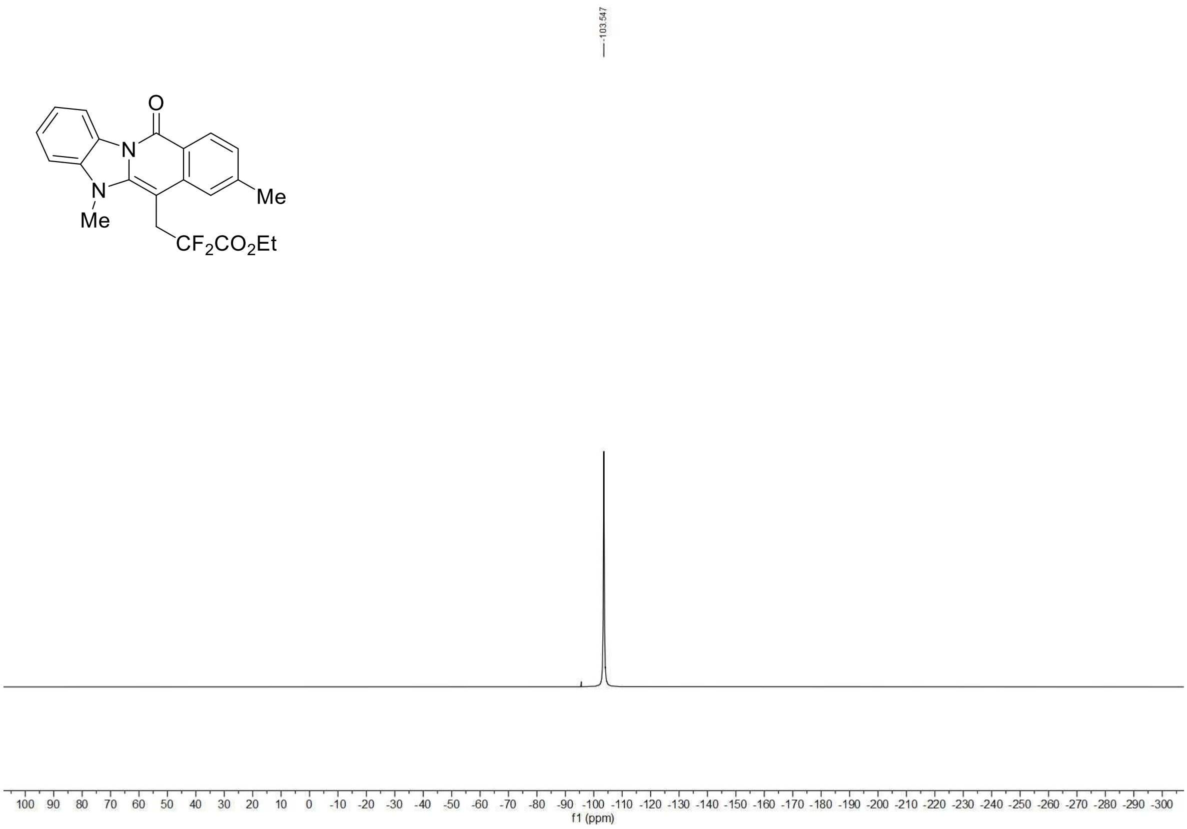

Supplementary Figure 283. ^{19}F-NMR of compound **24**, recorded at 376 MHz and 50 °C in $CDCl_3$.

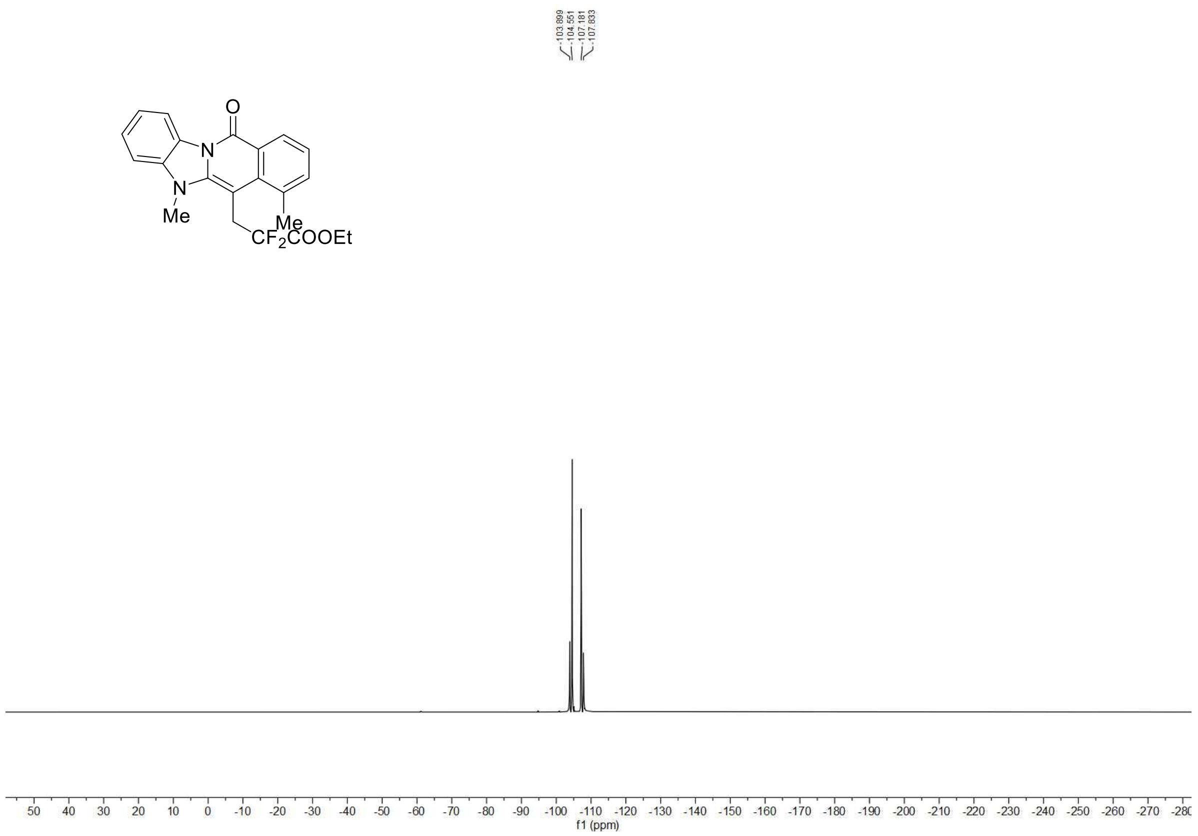

Supplementary Figure 284. ^{19}F-NMR of compound **25**, recorded at 376 MHz and 50 °C in $CDCl_3$.

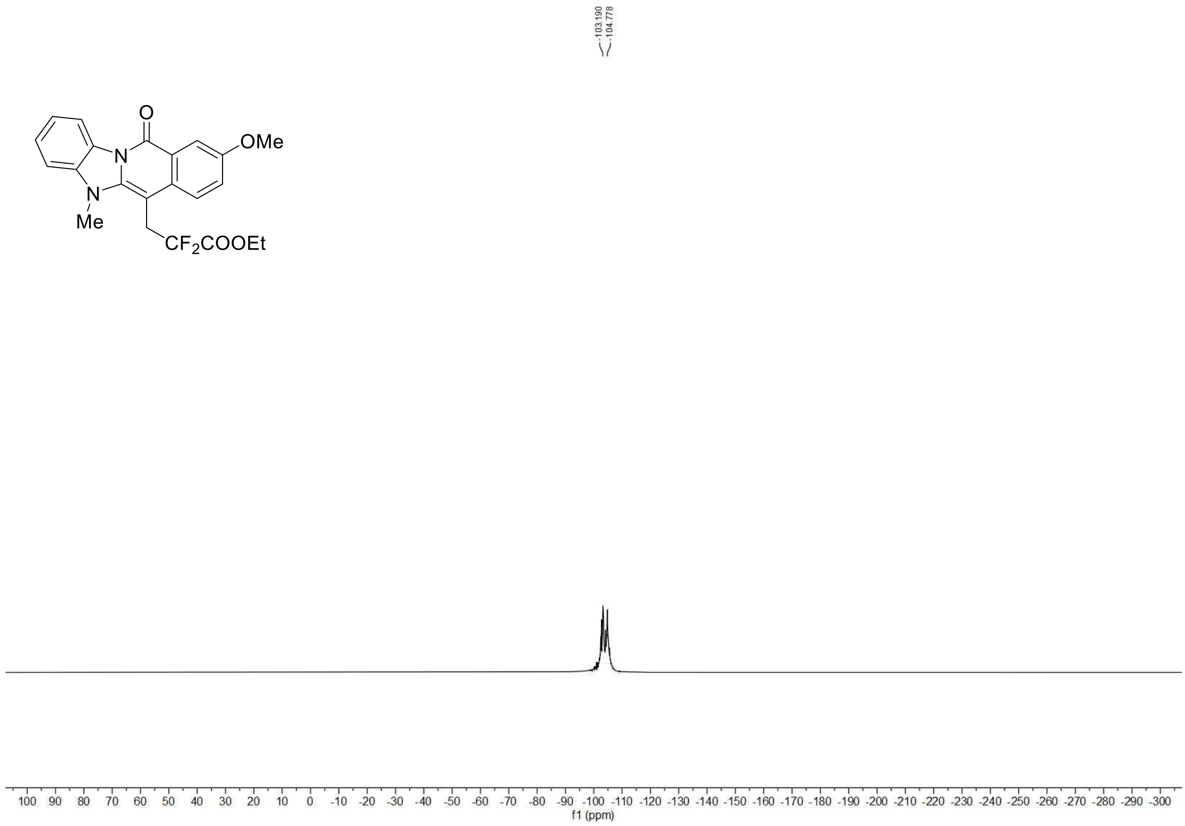

Supplementary Figure 285. ^{19}F-NMR of compound **26**, recorded at 376 MHz and 50 °C in $CDCl_3$.

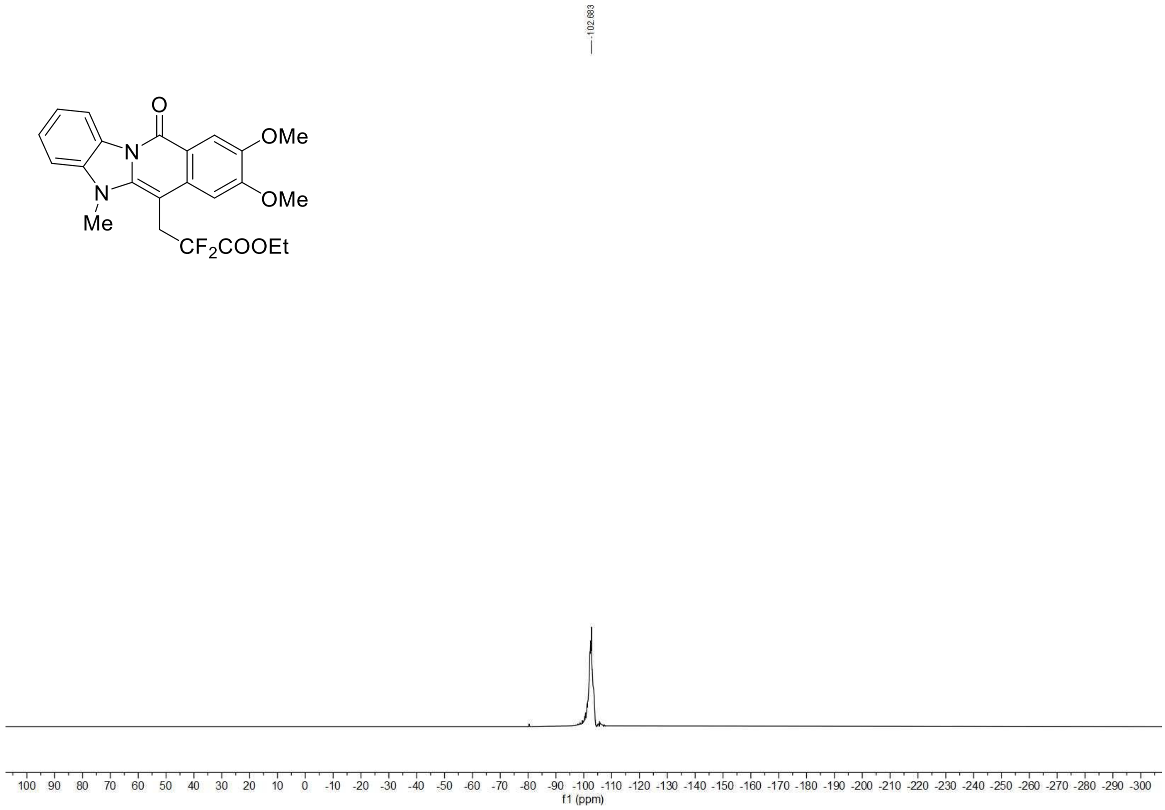

Supplementary Figure 286. ^{19}F-NMR of compound **27**, recorded at 376 MHz and 50 °C in $CDCl_3$.

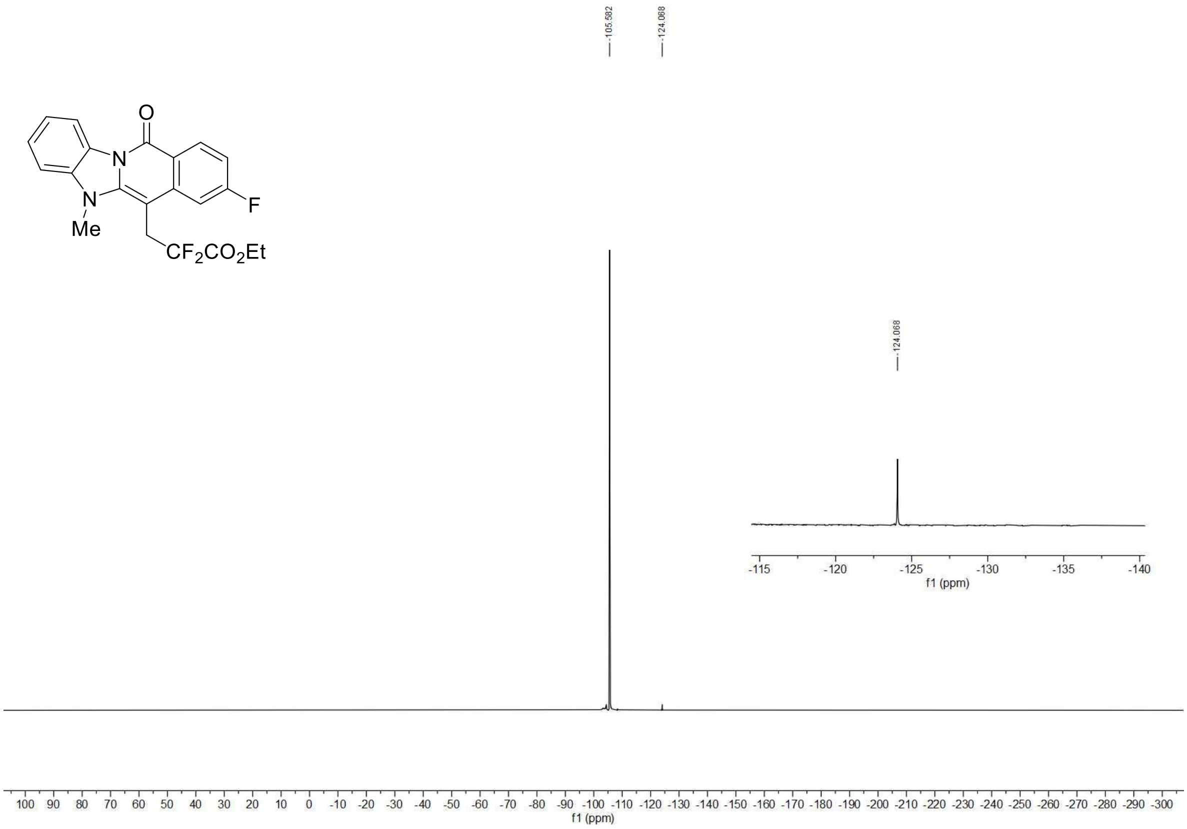

Supplementary Figure 287. ^{19}F-NMR of compound **28**, recorded at 376 MHz and 50 °C in $CDCl_3$.

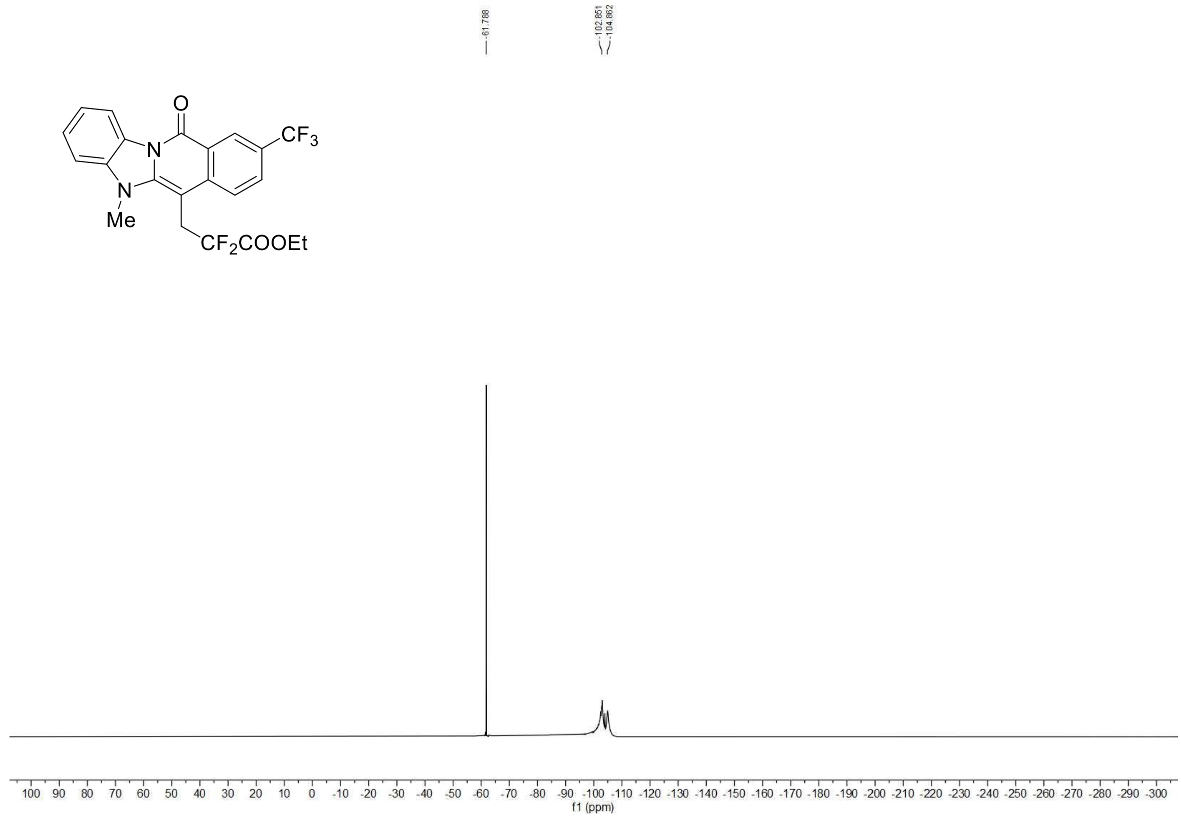

Supplementary Figure 288. ^{19}F-NMR of compound **29**, recorded at 376 MHz and 50 °C in $CDCl_3$.

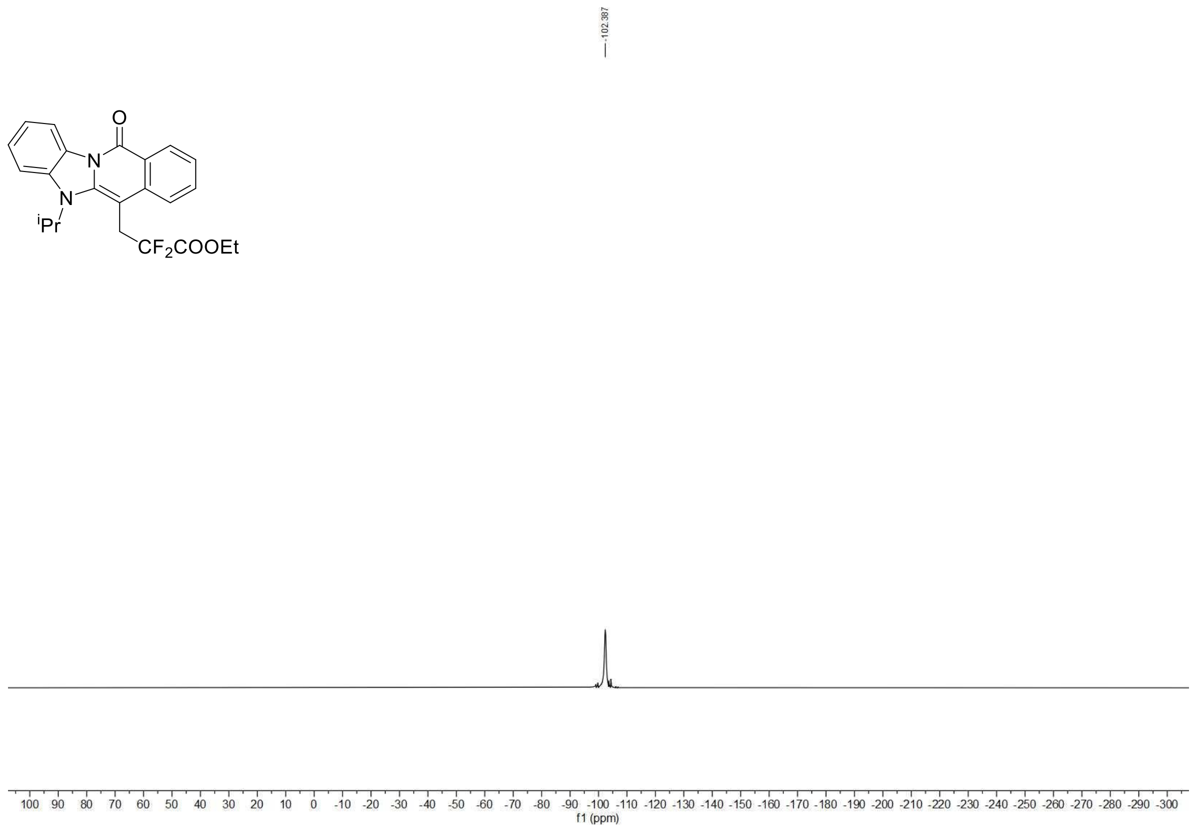

Supplementary Figure 289. ^{19}F-NMR of compound **30**, recorded at 376 MHz and 50 °C in $CDCl_3$.

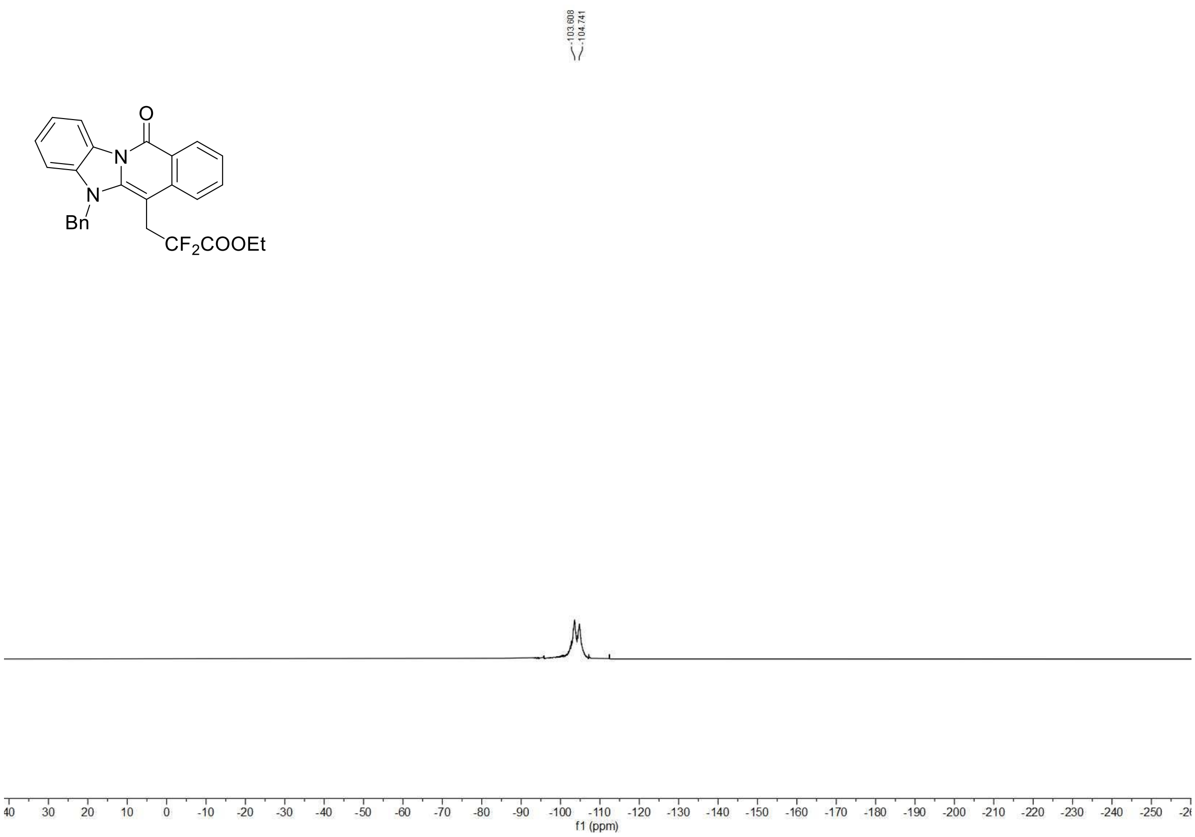

Supplementary Figure 290. ^{19}F-NMR of compound **31**, recorded at 376 MHz and 50 °C in $CDCl_3$.

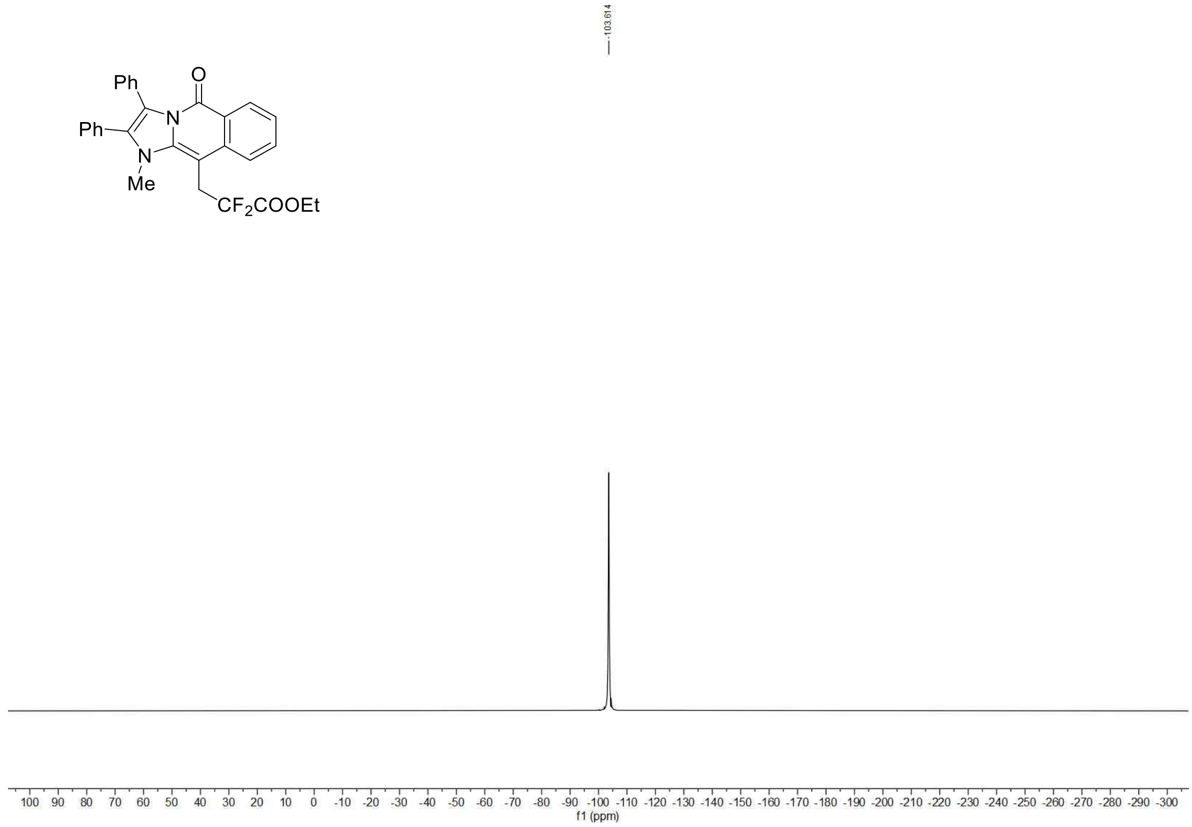

Supplementary Figure 291. ^{19}F-NMR of compound **32**, recorded at 376 MHz and 50 °C in $CDCl_3$.

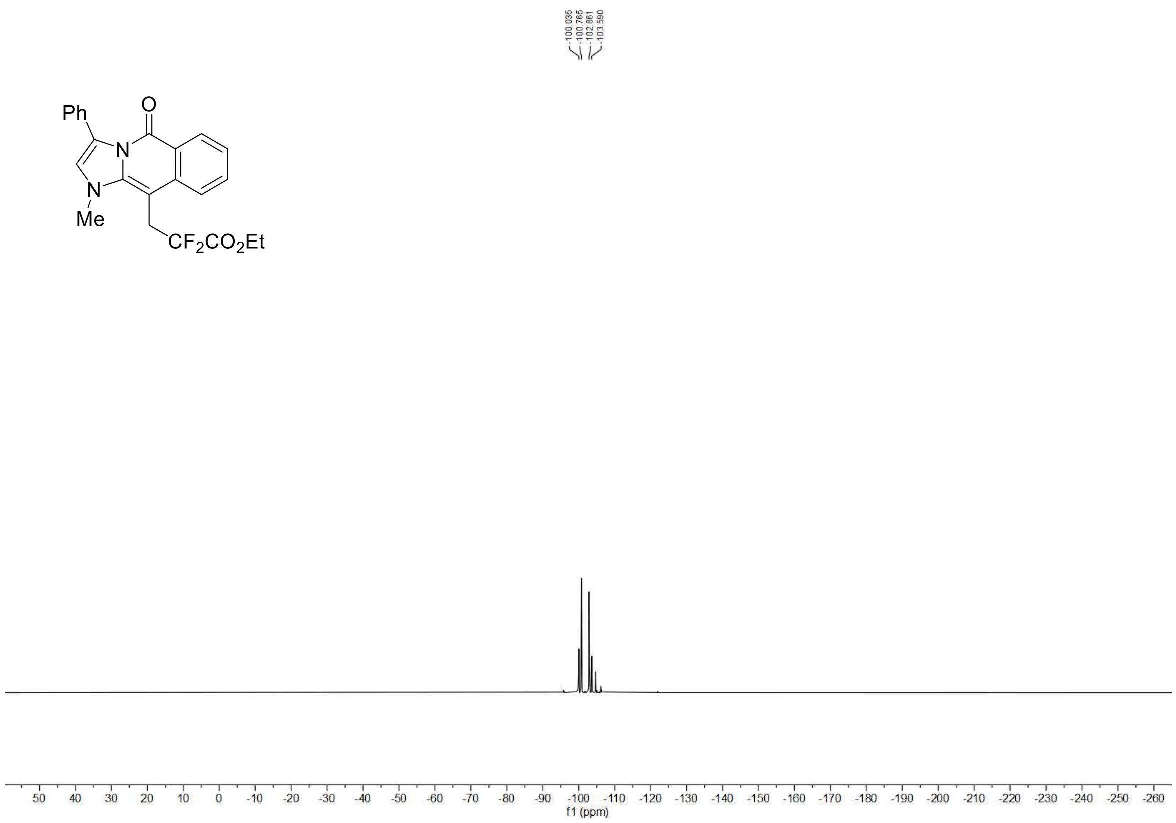

Supplementary Figure 292. ^{19}F-NMR of compound **33**, recorded at 376 MHz and 50 °C in $CDCl_3$.

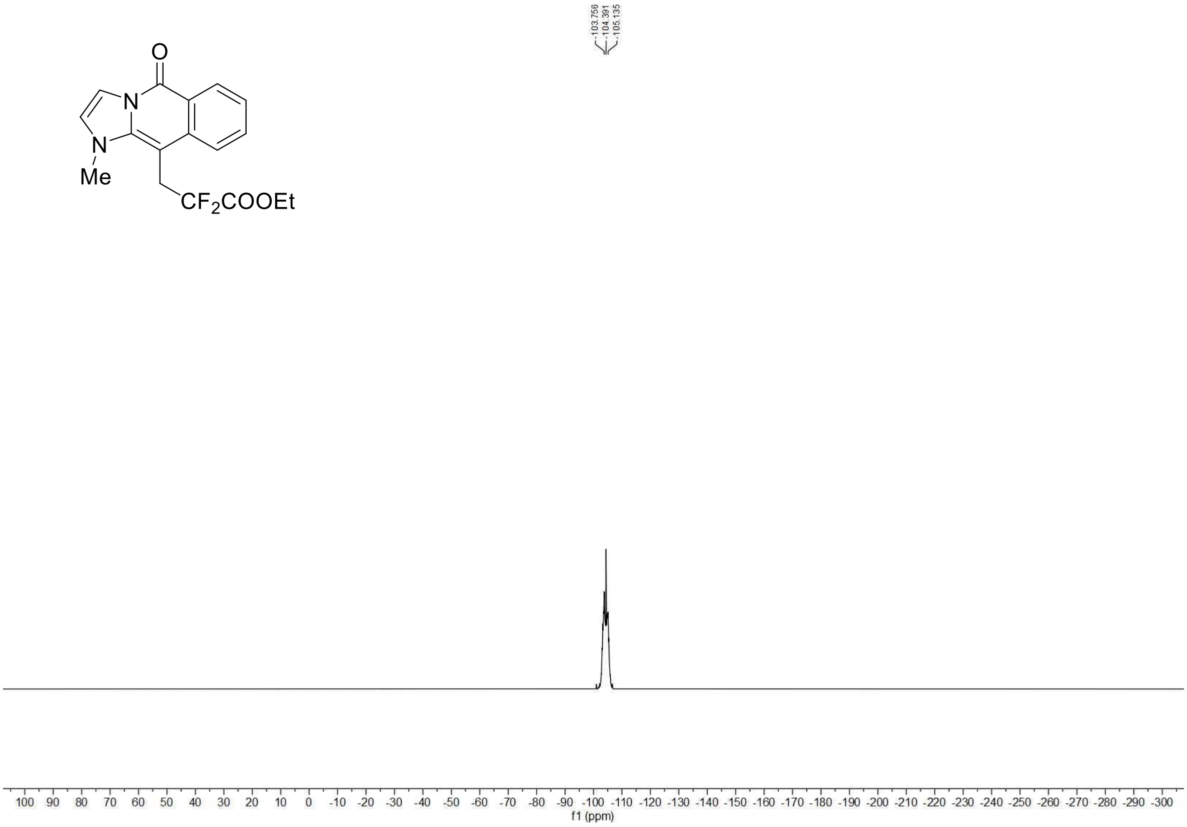

Supplementary Figure 293. ^{19}F-NMR of compound **34**, recorded at 376 MHz and 50 °C in $CDCl_3$.

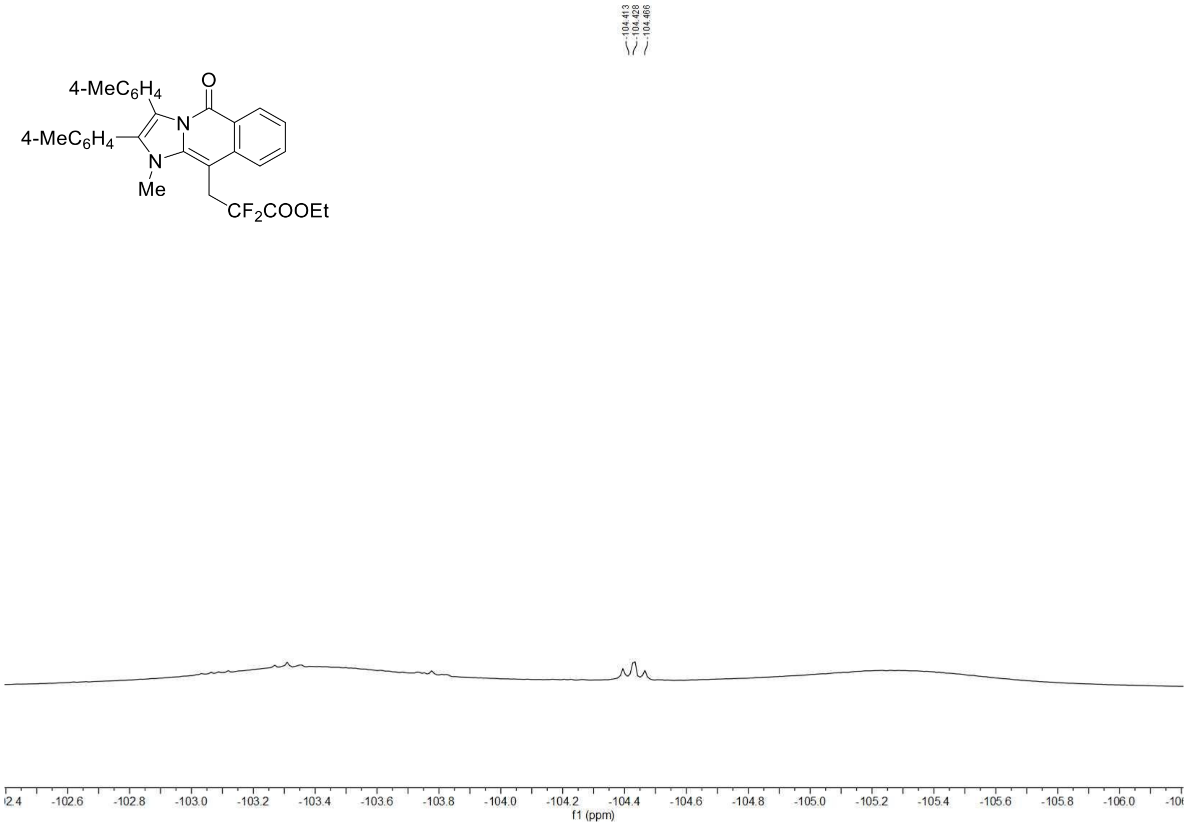

Supplementary Figure 294. ^{19}F-NMR of compound **35**, recorded at 376 MHz and 50 °C in $CDCl_3$.

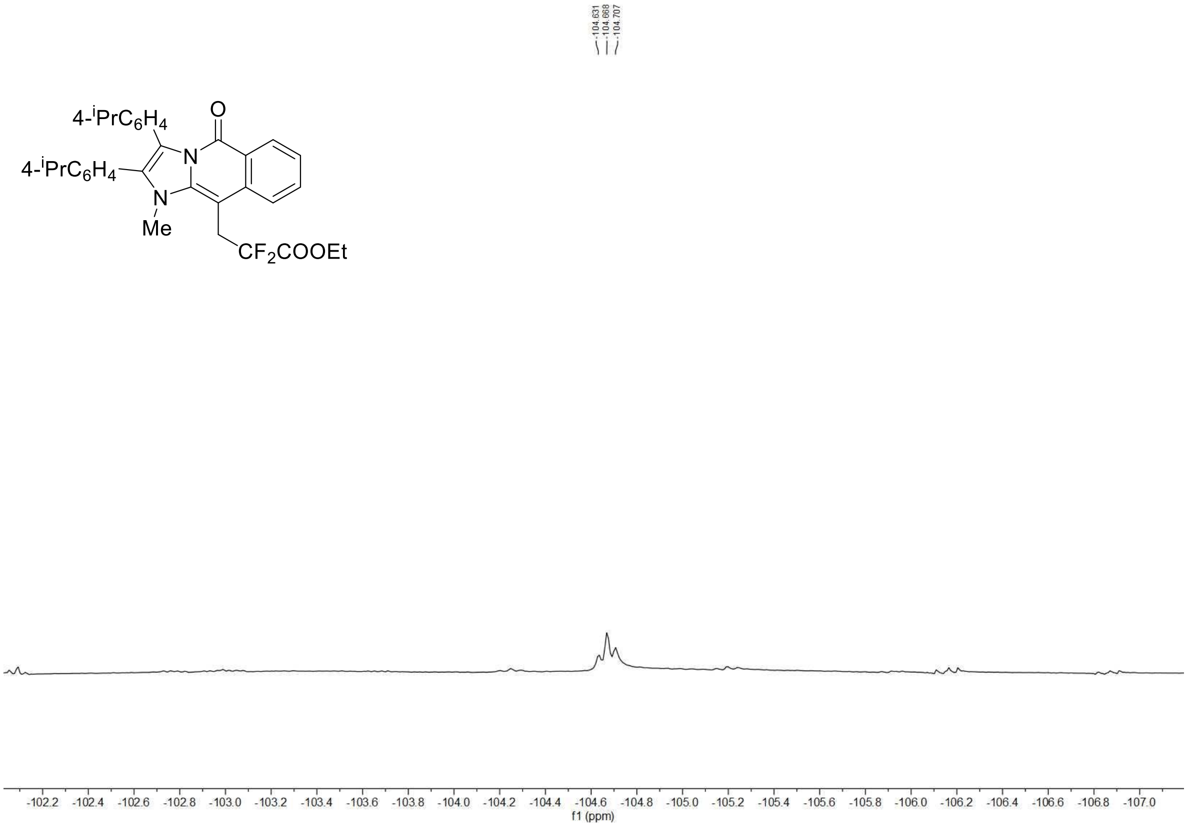

Supplementary Figure 295. ^{19}F-NMR of compound **36**, recorded at 376 MHz and 50 °C in $CDCl_3$.

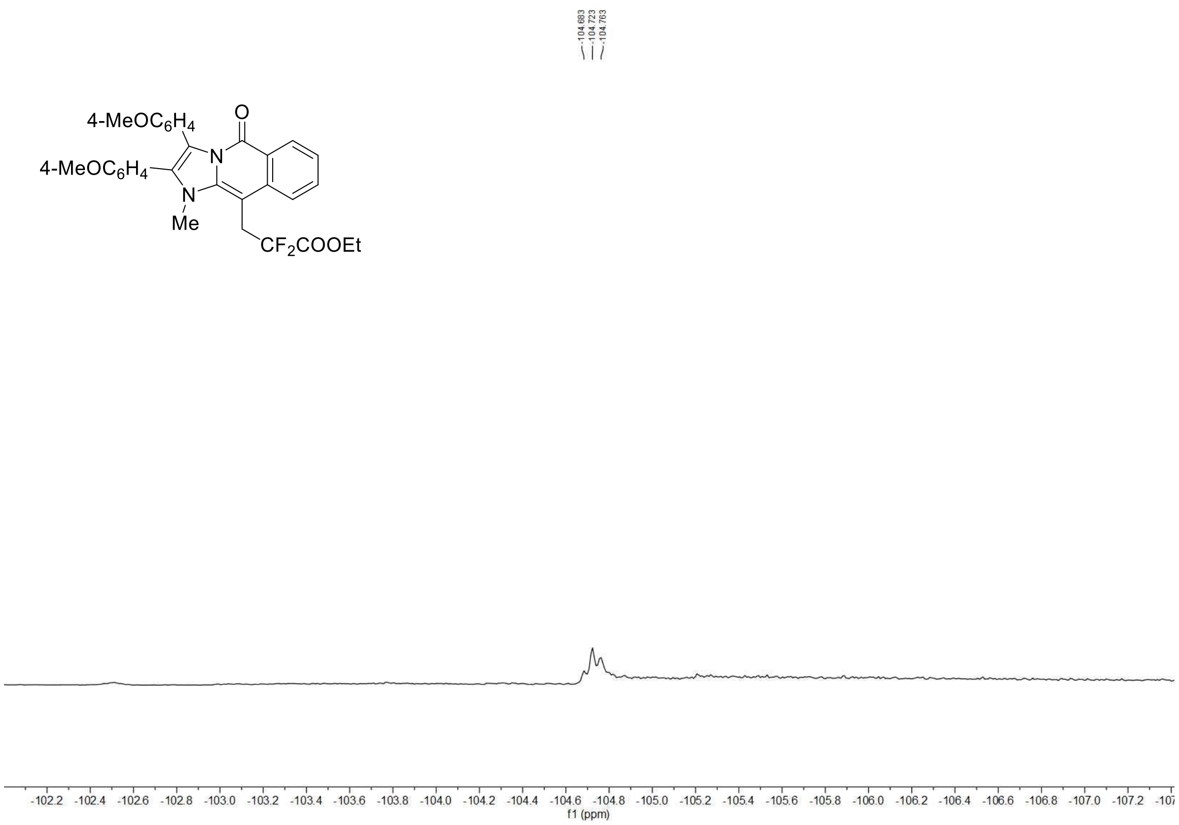

Supplementary Figure 296. ^{19}F-NMR of compound **37**, recorded at 376 MHz and 50 °C in $CDCl_3$.

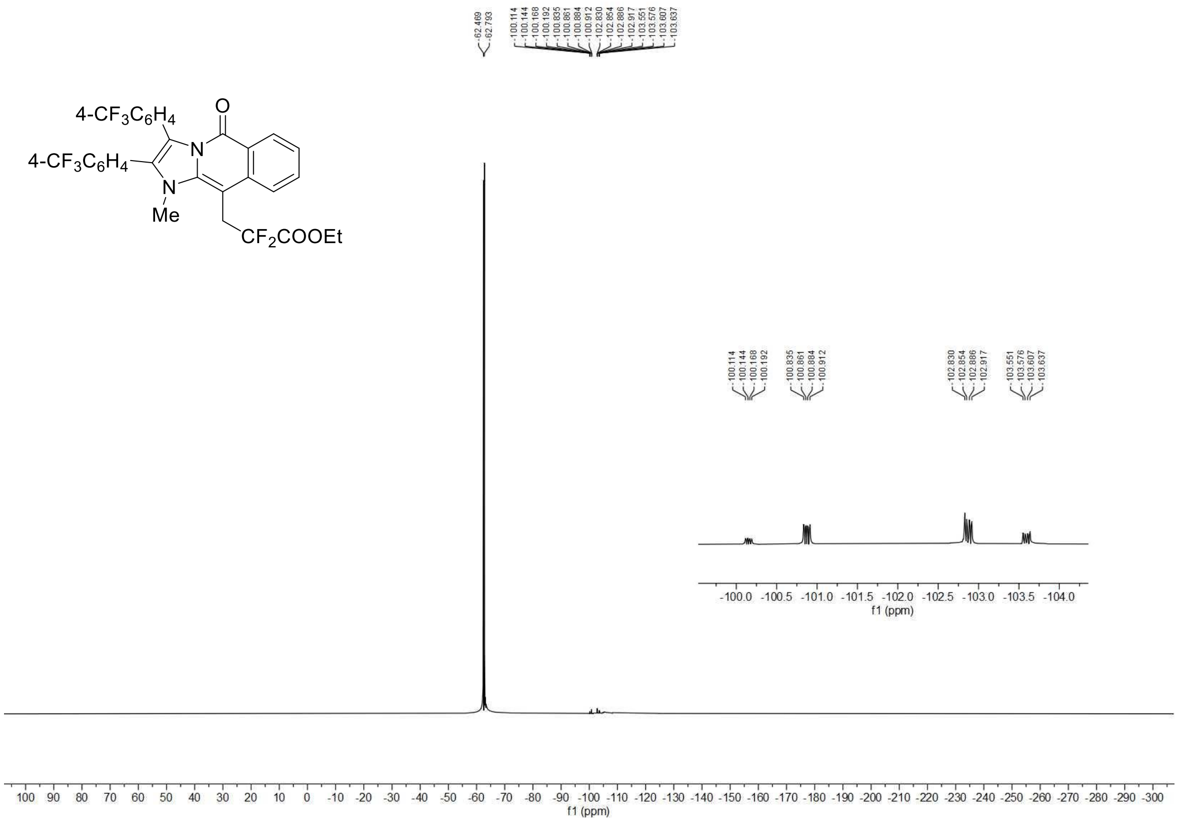

Supplementary Figure 297. ^{19}F-NMR of compound **38**, recorded at 376 MHz and 50 °C in $CDCl_3$.

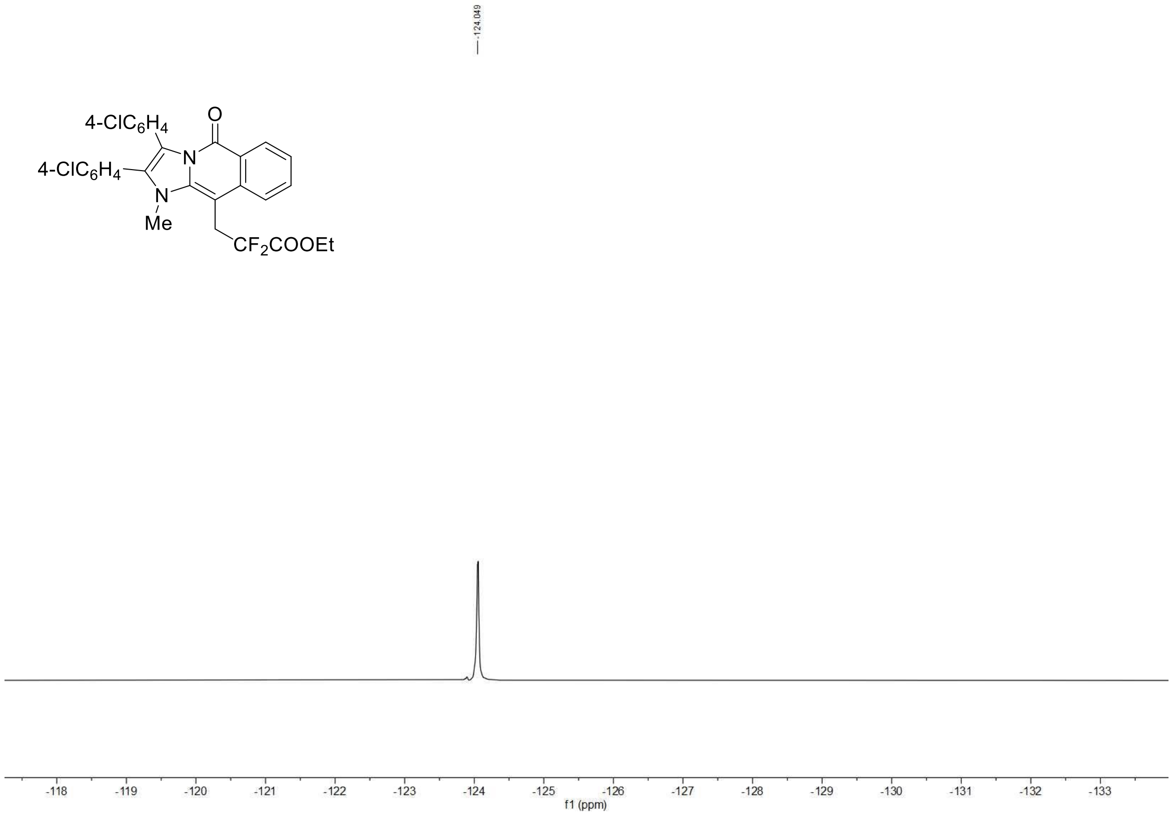

Supplementary Figure 298. ^{19}F-NMR of compound **39**, recorded at 376 MHz and 50 °C in $CDCl_3$.

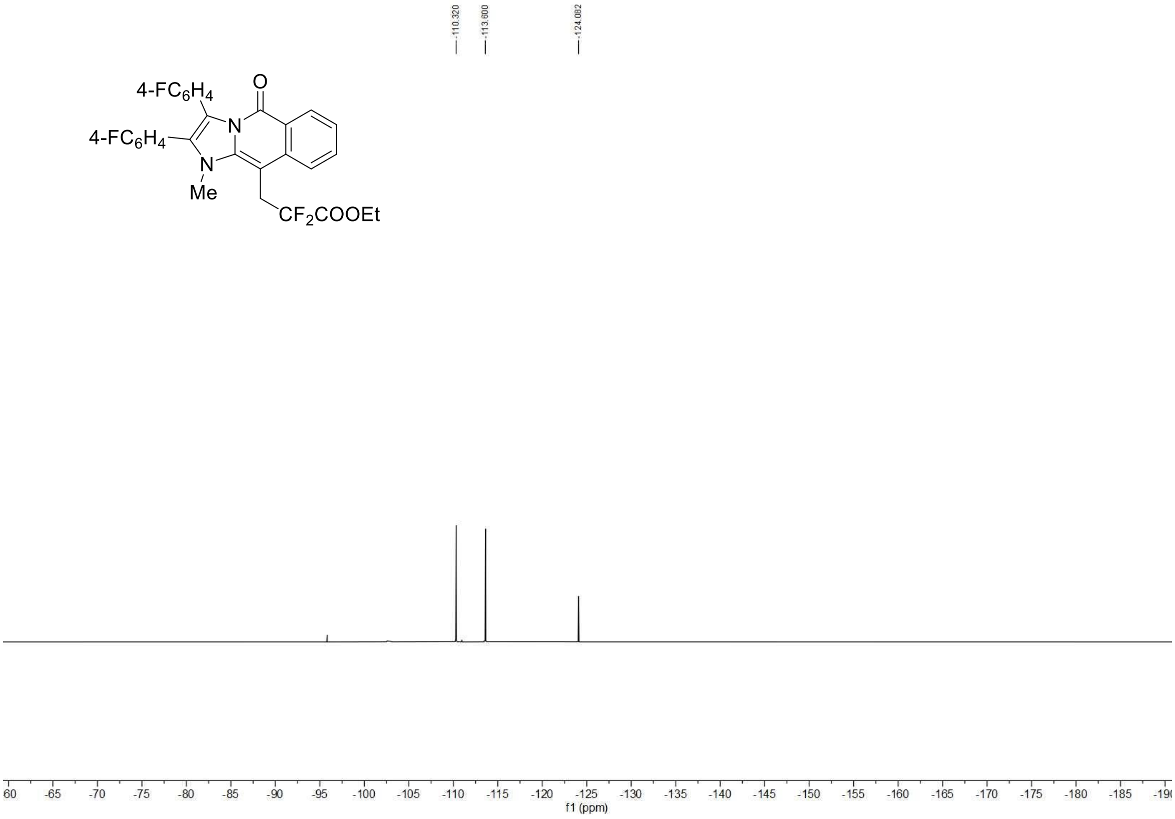

Supplementary Figure 299. ^{19}F-NMR of compound **40**, recorded at 376 MHz and 50 °C in $CDCl_3$.

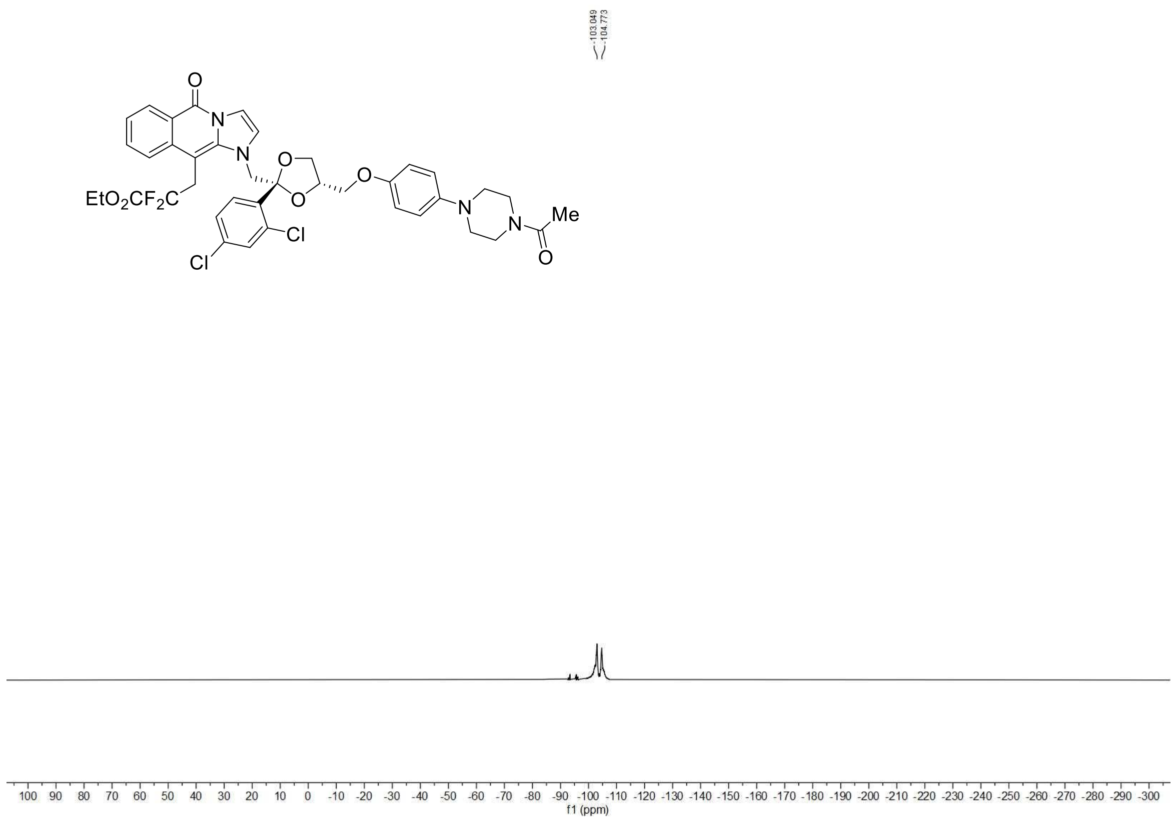

Supplementary Figure 300. ^{19}F-NMR of compound **41**, recorded at 376 MHz and 50 °C in $CDCl_3$.

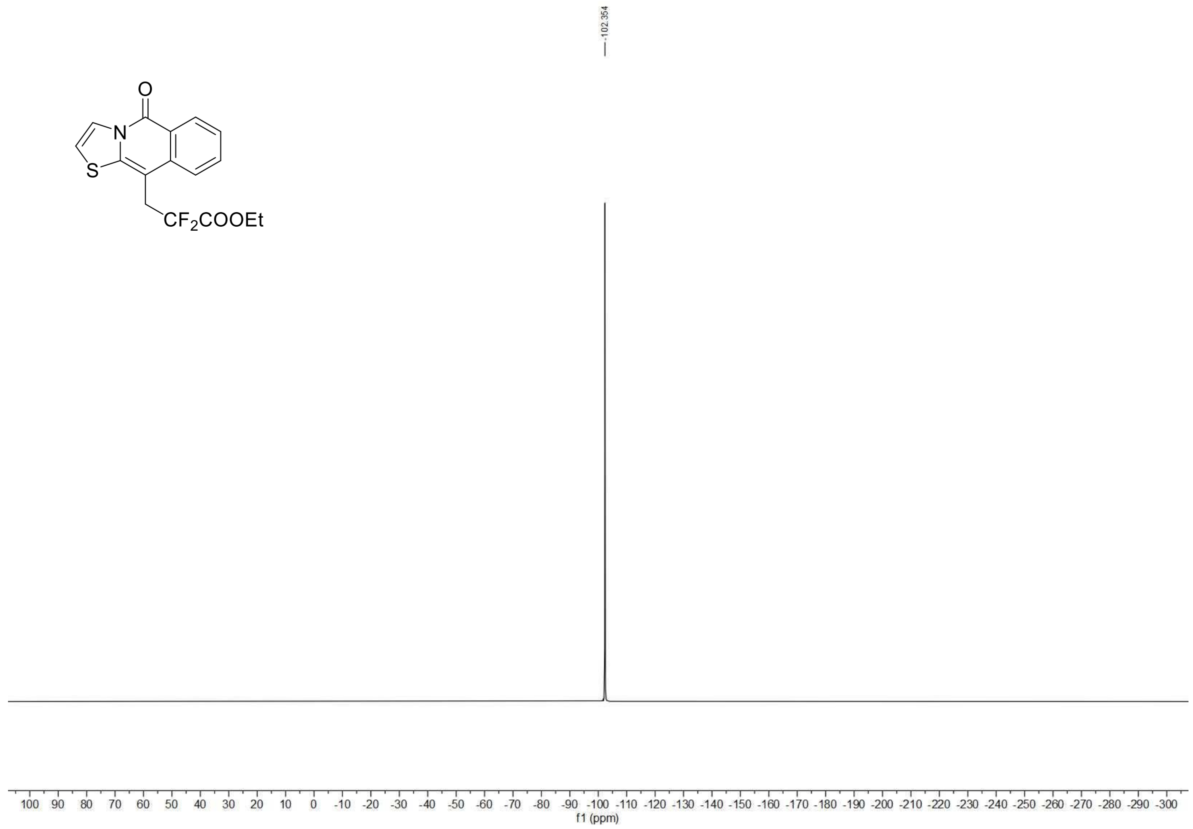

Supplementary Figure 301. ^{19}F-NMR of compound **42**, recorded at 376 MHz and 25 °C in $CDCl_3$.

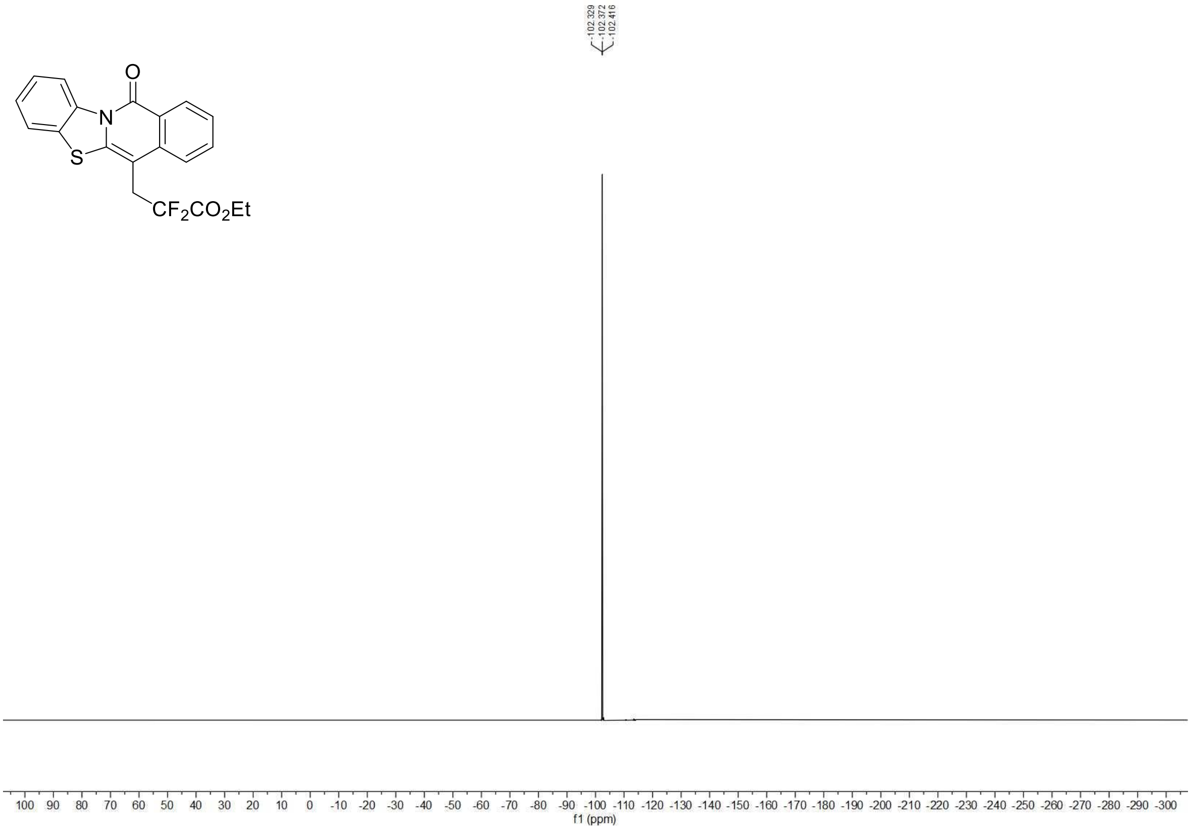

Supplementary Figure 302. ^{19}F-NMR of compound **43**, recorded at 376 MHz and 25 °C in $CDCl_3$.

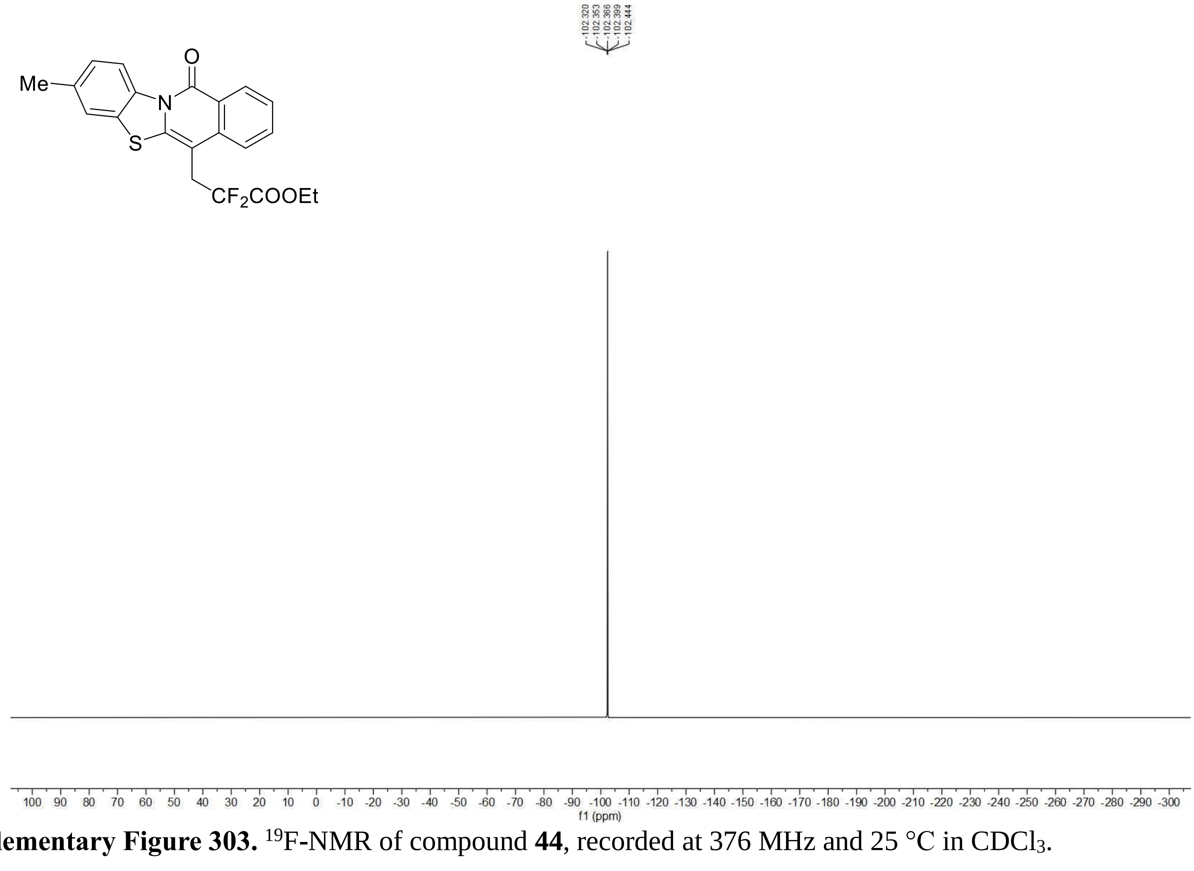

Supplementary Figure 303. ^{19}F-NMR of compound **44**, recorded at 376 MHz and 25 °C in $CDCl_3$.

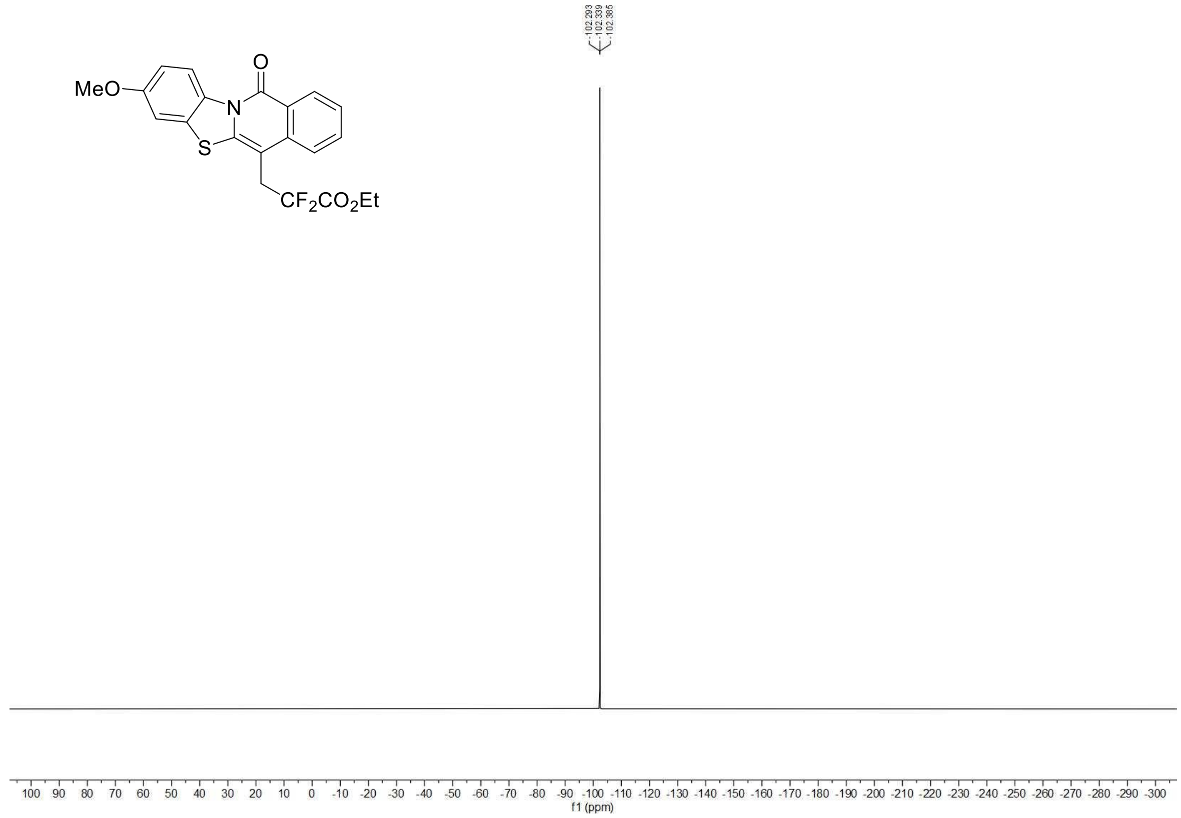

Supplementary Figure 304. ^{19}F-NMR of compound **45**, recorded at 376 MHz and 25 °C in $CDCl_3$.

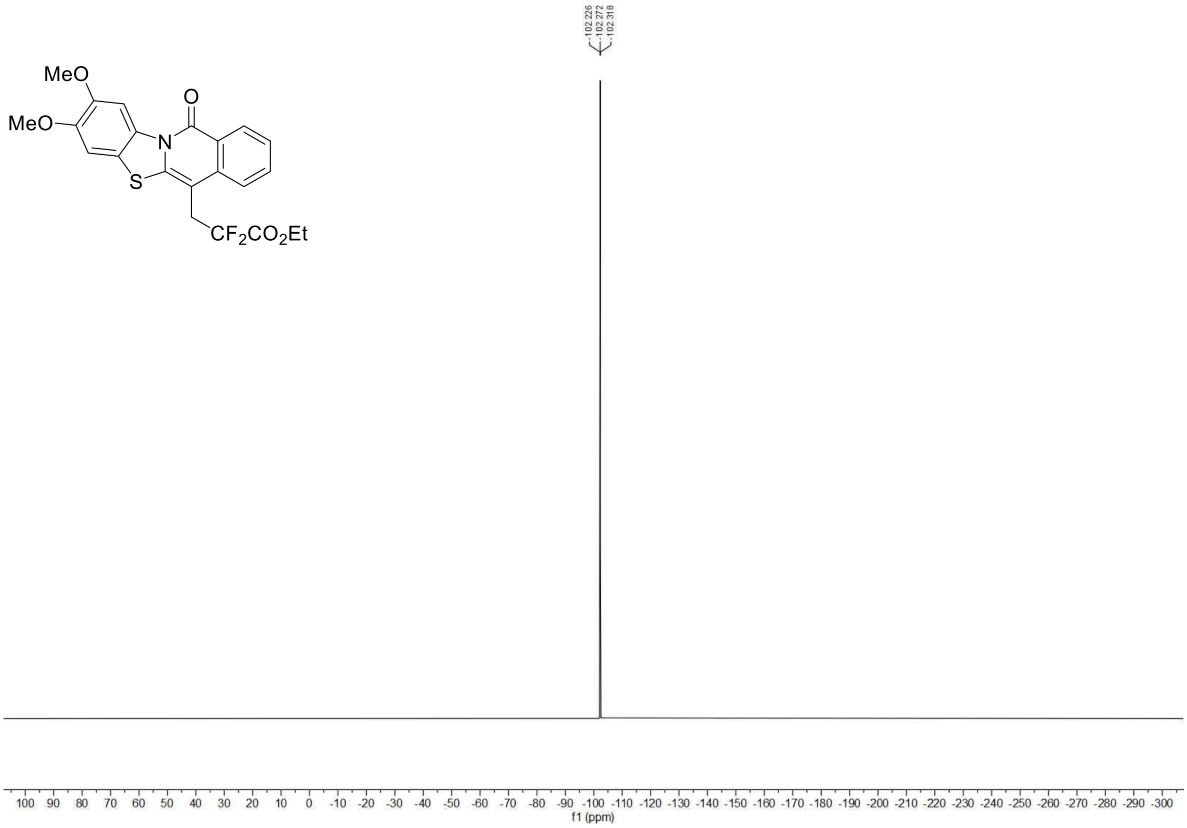

Supplementary Figure 305. ^{19}F-NMR of compound **46**, recorded at 376 MHz and 25 °C in $CDCl_3$.

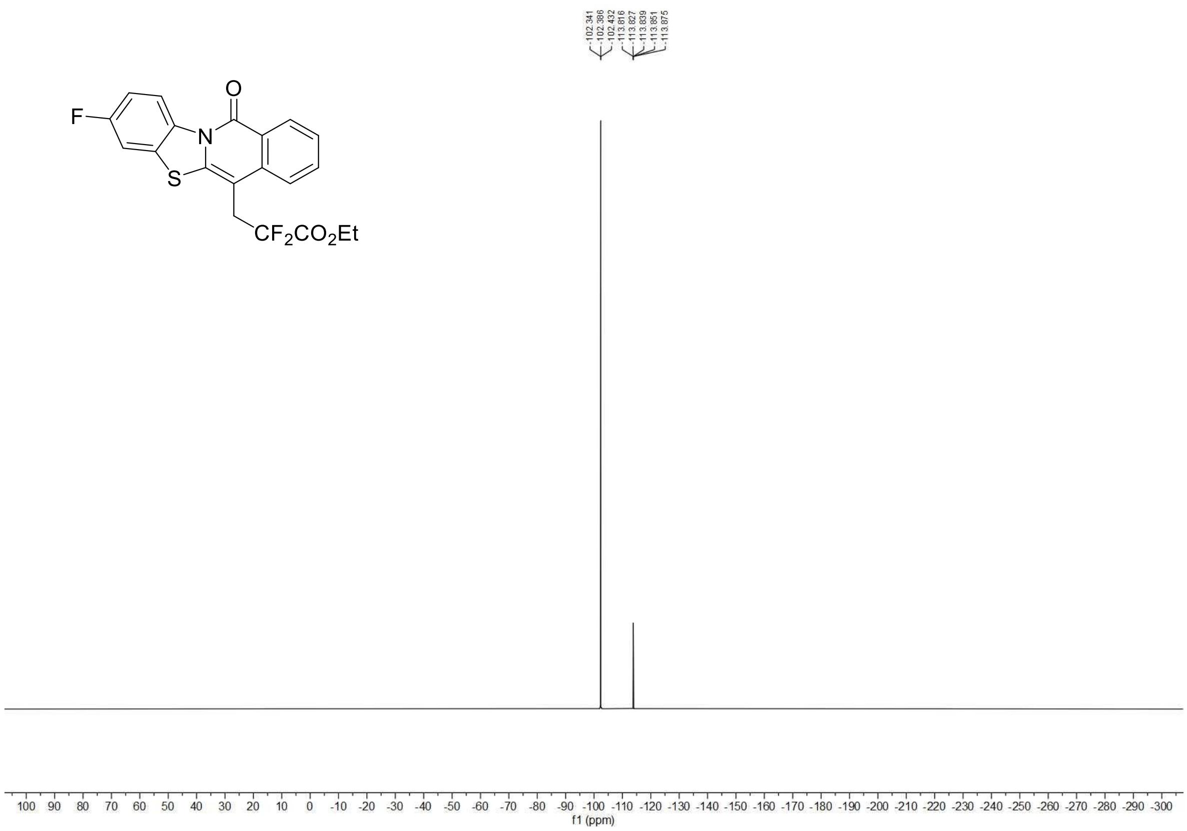

Supplementary Figure 306. ^{19}F-NMR of compound **47**, recorded at 376 MHz and 25 °C in $CDCl_3$.

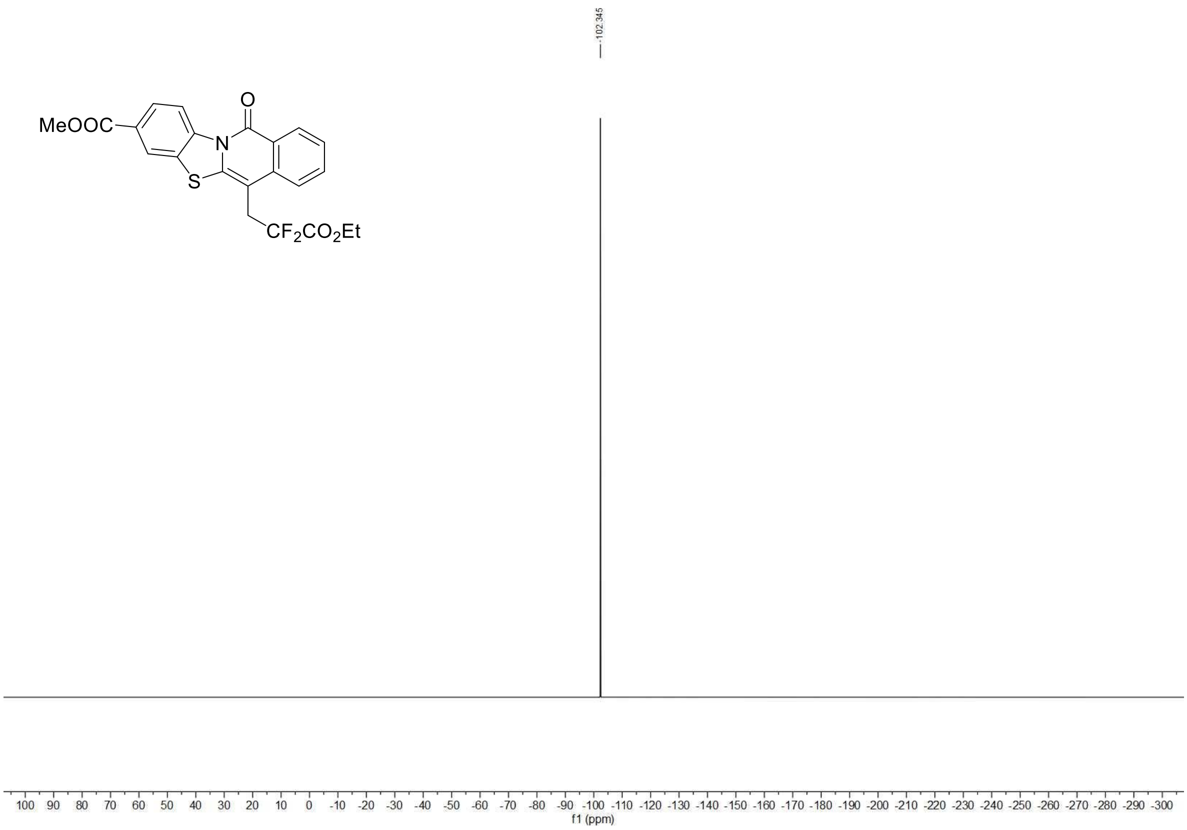

Supplementary Figure 307. ^{19}F-NMR of compound **48**, recorded at 376 MHz and 25 °C in $CDCl_3$.

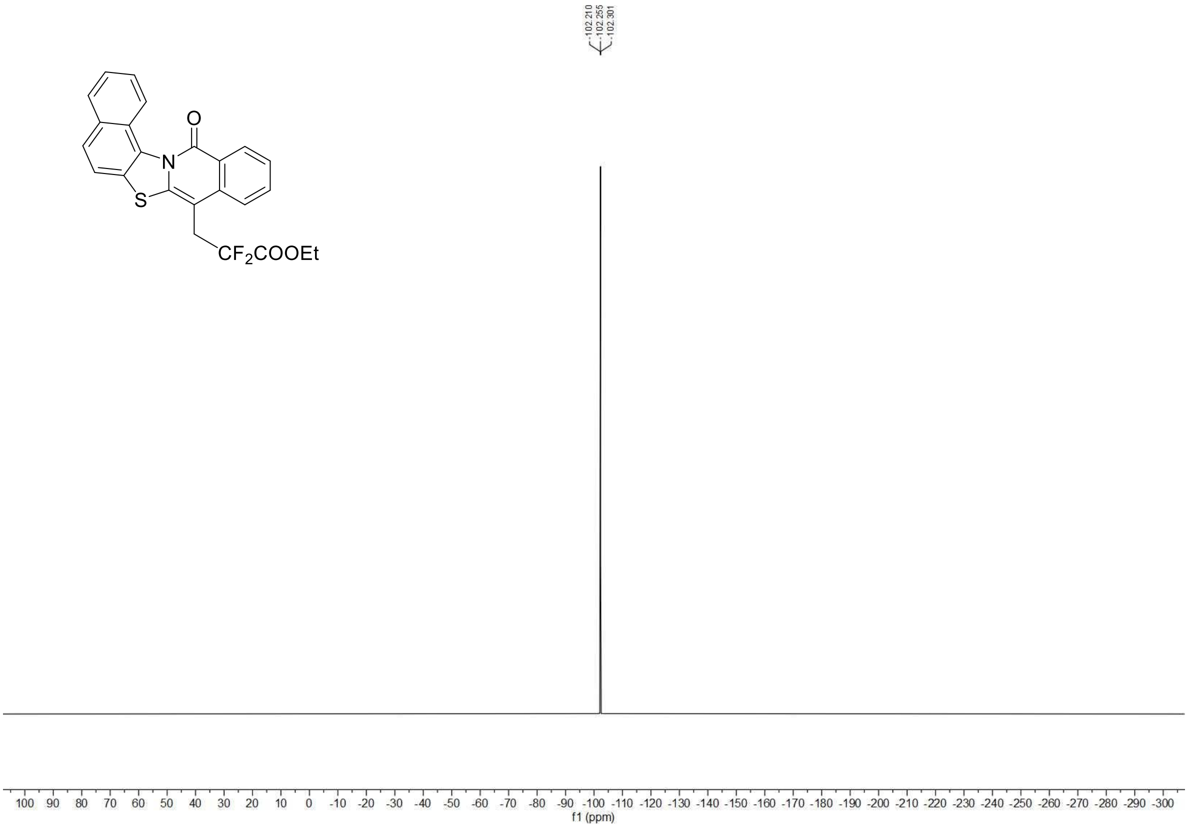

Supplementary Figure 308. ^{19}F-NMR of compound **49**, recorded at 376 MHz and 25 °C in $CDCl_3$.

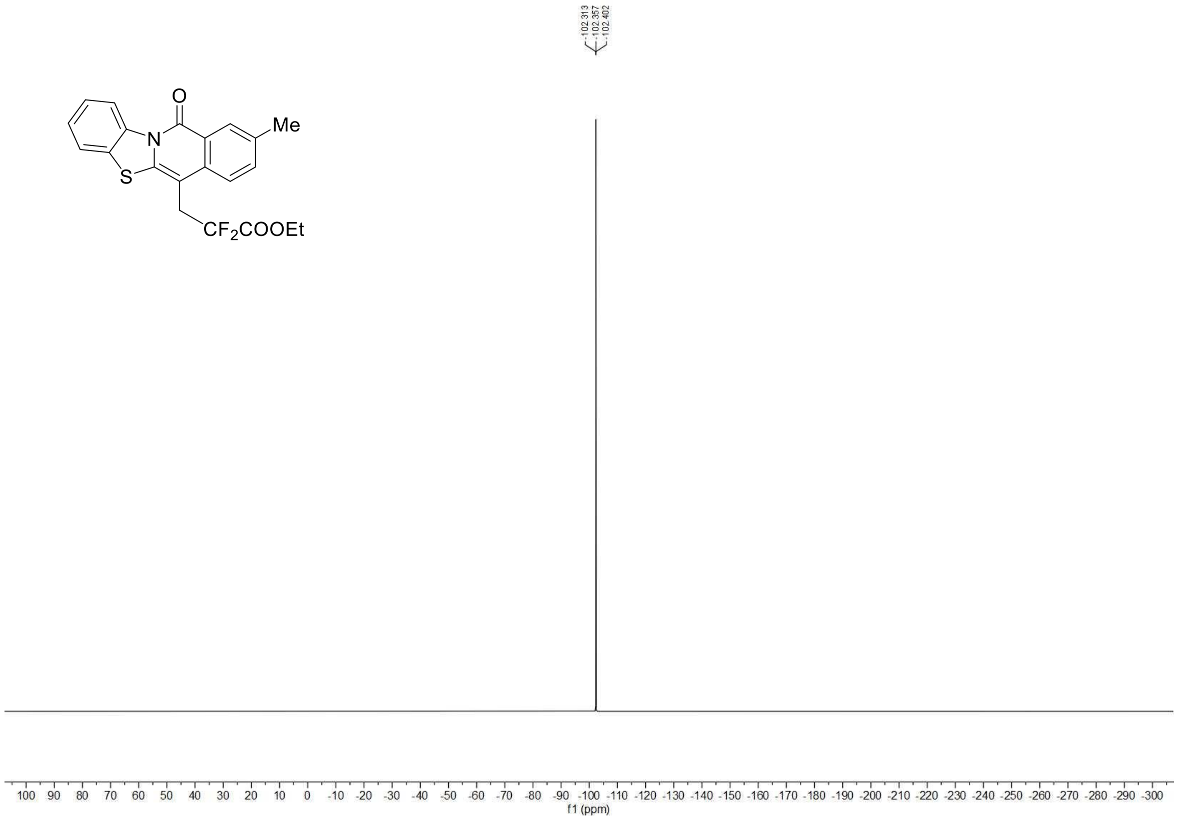

Supplementary Figure 309. ^{19}F-NMR of compound **50**, recorded at 376 MHz and 25 °C in $CDCl_3$.

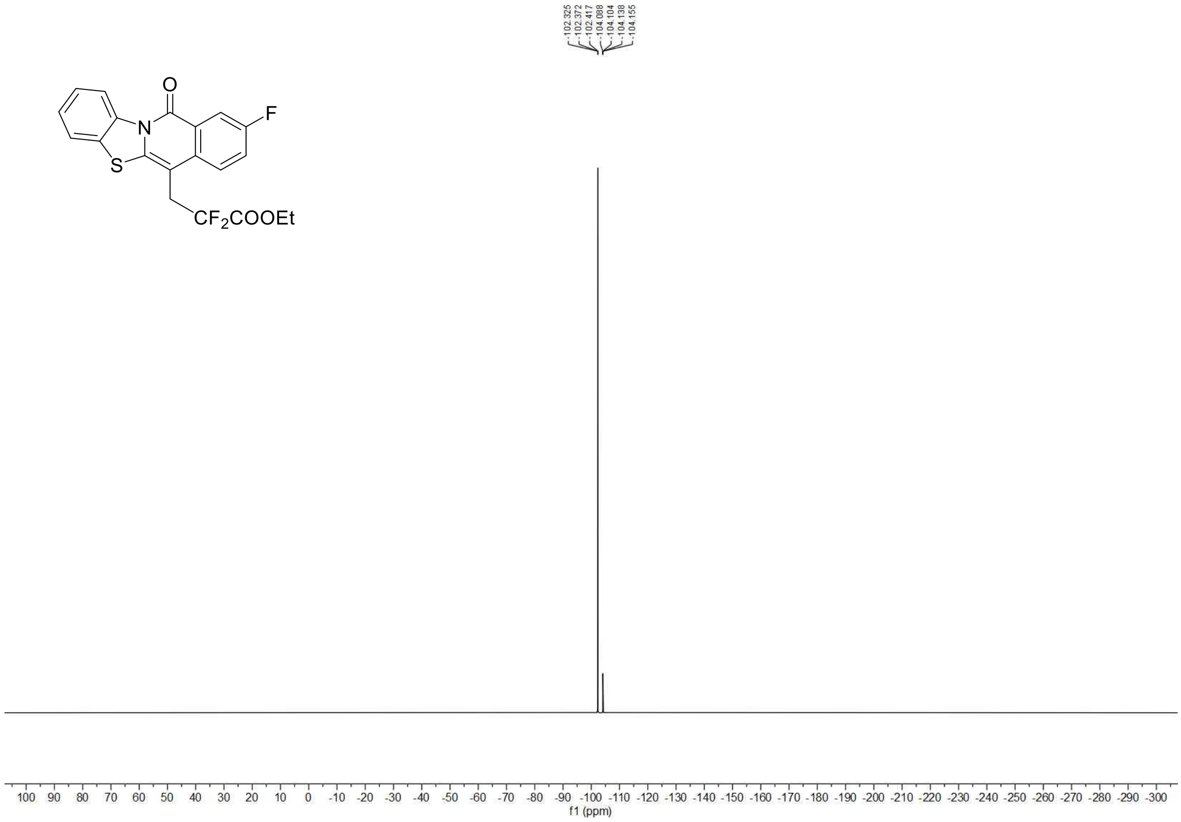

Supplementary Figure 310. ^{19}F-NMR of compound **51**, recorded at 376 MHz and 25 °C in $CDCl_3$.

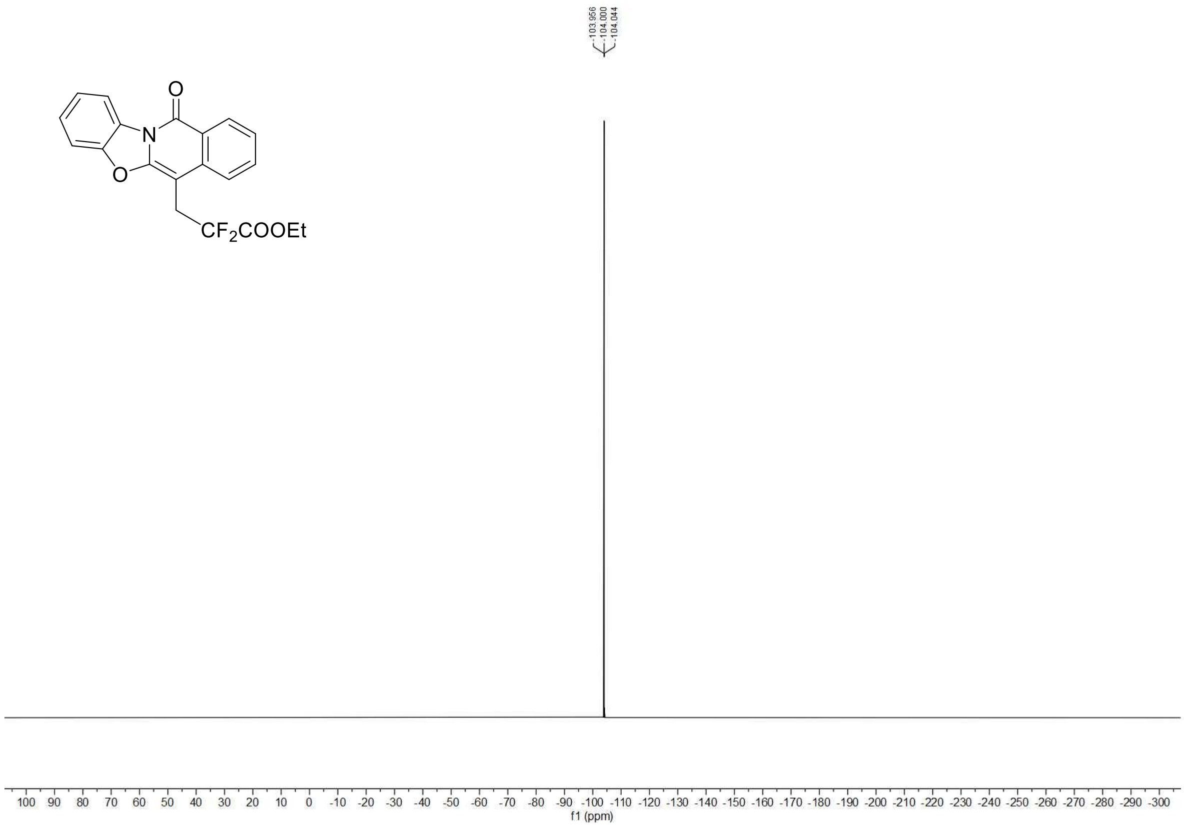

Supplementary Figure 311. ^{19}F-NMR of compound **52**, recorded at 376 MHz and 25 °C in $CDCl_3$.

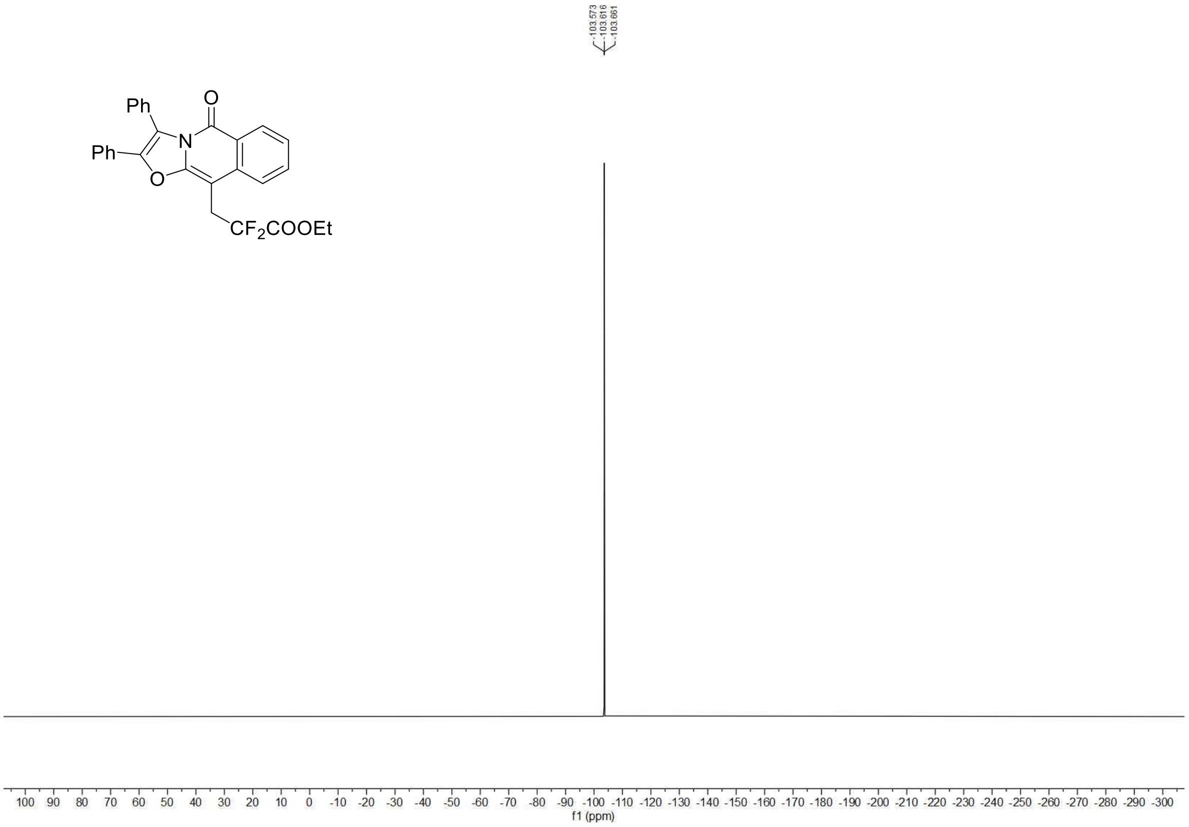

Supplementary Figure 312. ^{19}F-NMR of compound **53**, recorded at 376 MHz and 25 °C in $CDCl_3$.

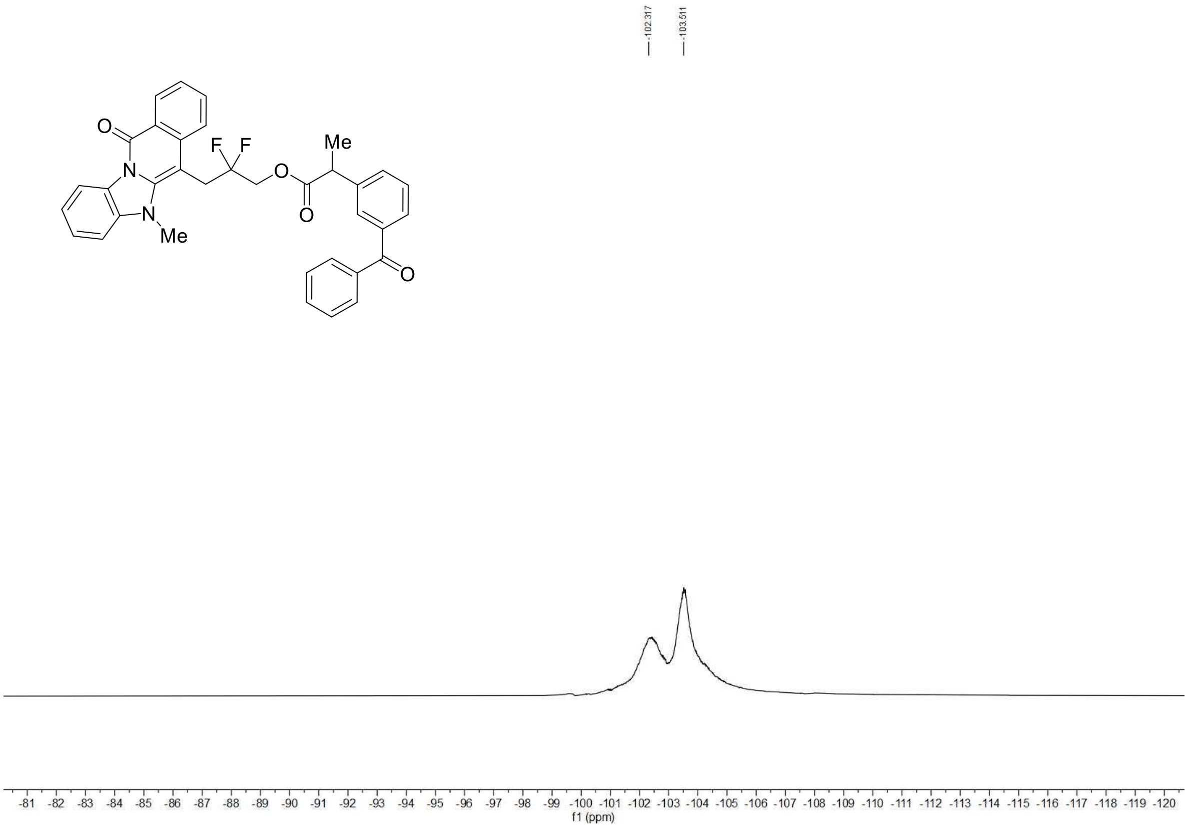

Supplementary Figure 313. ^{19}F-NMR of compound **60**, recorded at 376 MHz and 50 °C in $CDCl_3$.

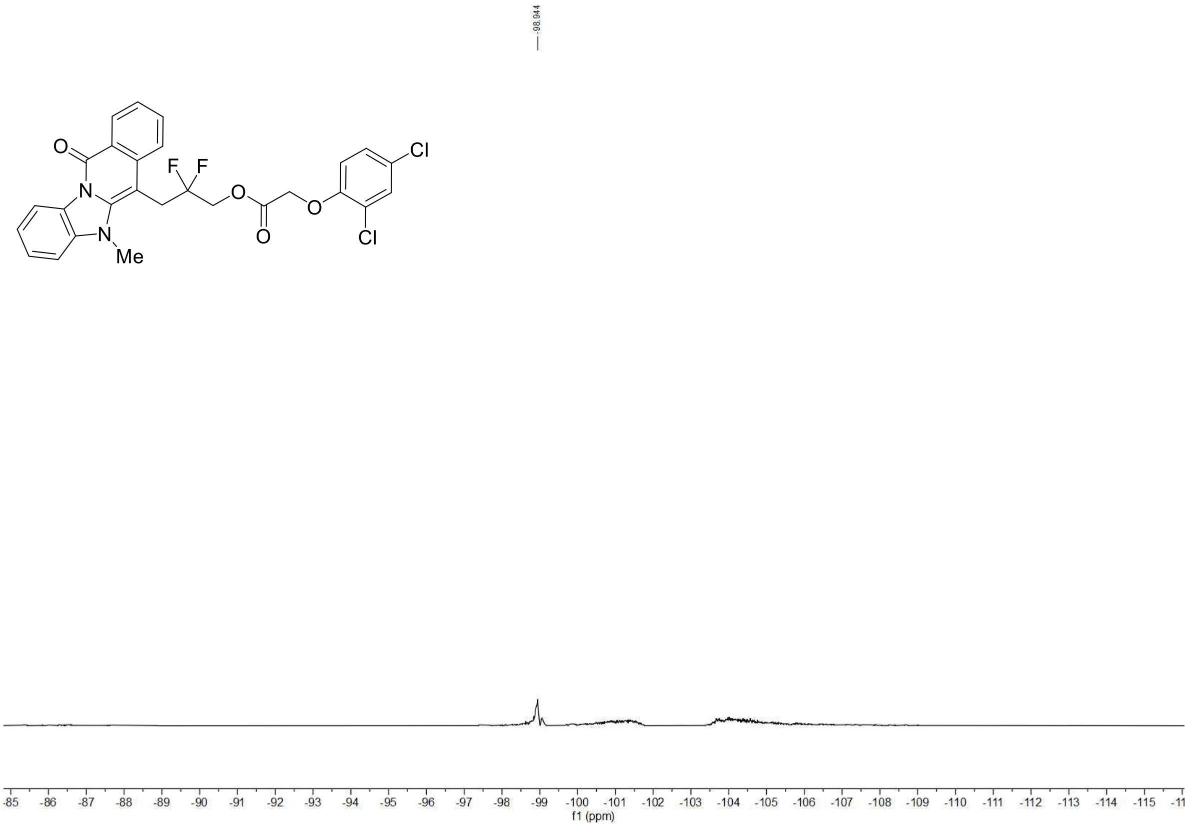

Supplementary Figure 314. ^{19}F-NMR of compound **61**, recorded at 376 MHz and 50 °C in $CDCl_3$.

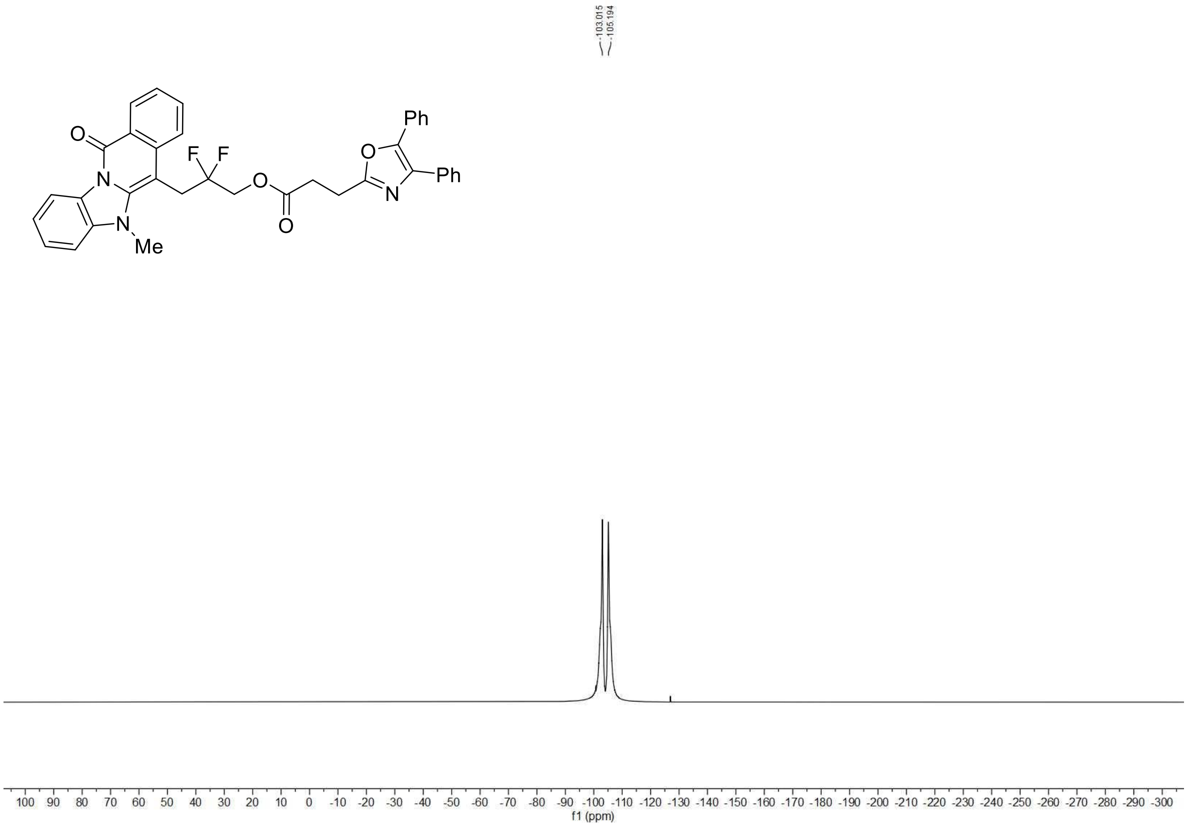

Supplementary Figure 315. ^{19}F-NMR of compound **63**, recorded at 376 MHz and 50 °C in $CDCl_3$.

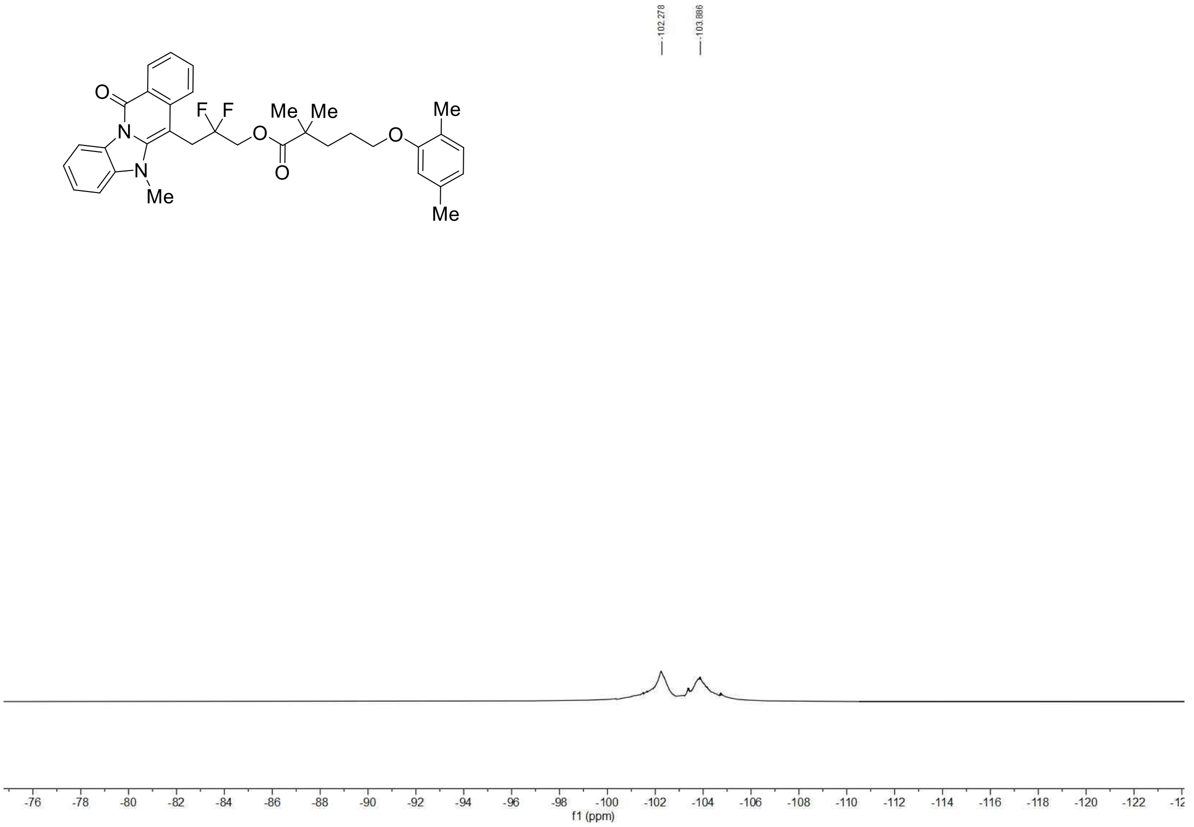

Supplementary Figure 316. ^{19}F-NMR of compound **64**, recorded at 376 MHz and 50 °C in $CDCl_3$.

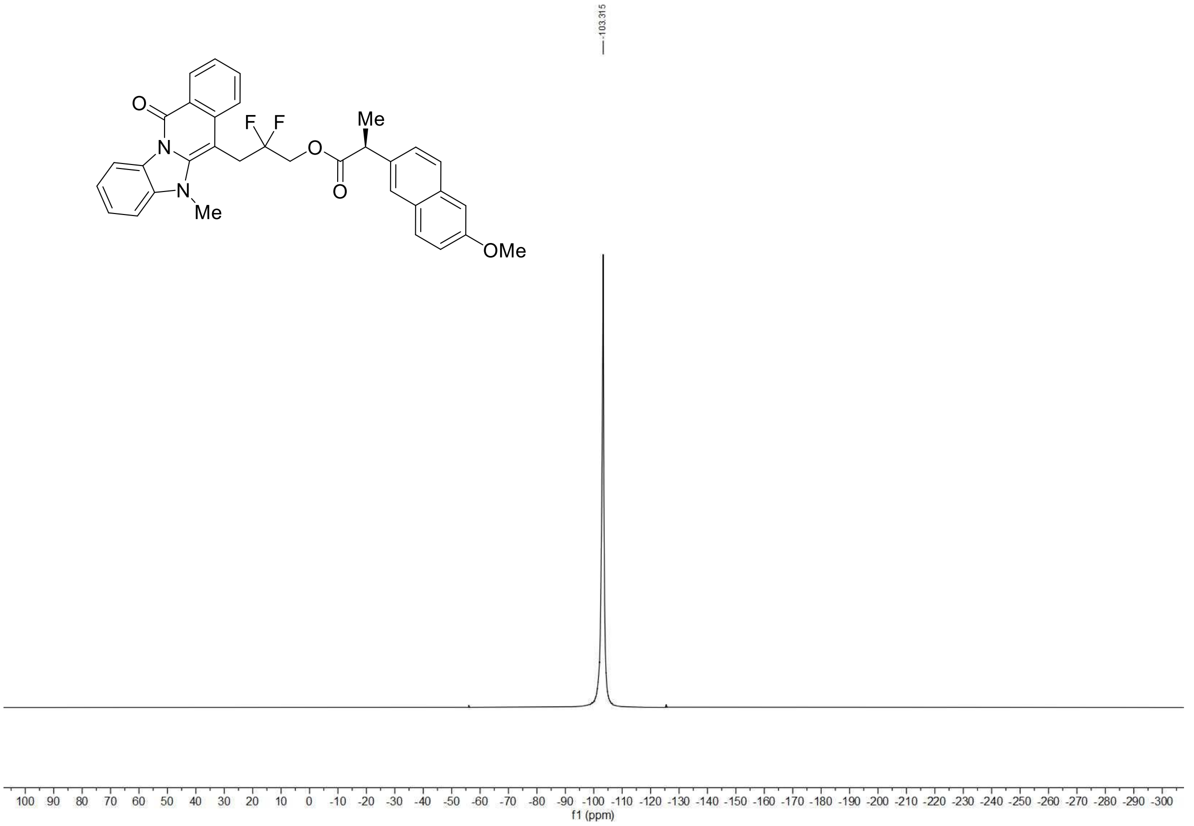

Supplementary Figure 317. ^{19}F-NMR of compound **65**, recorded at 376 MHz and 50 °C in $CDCl_3$.

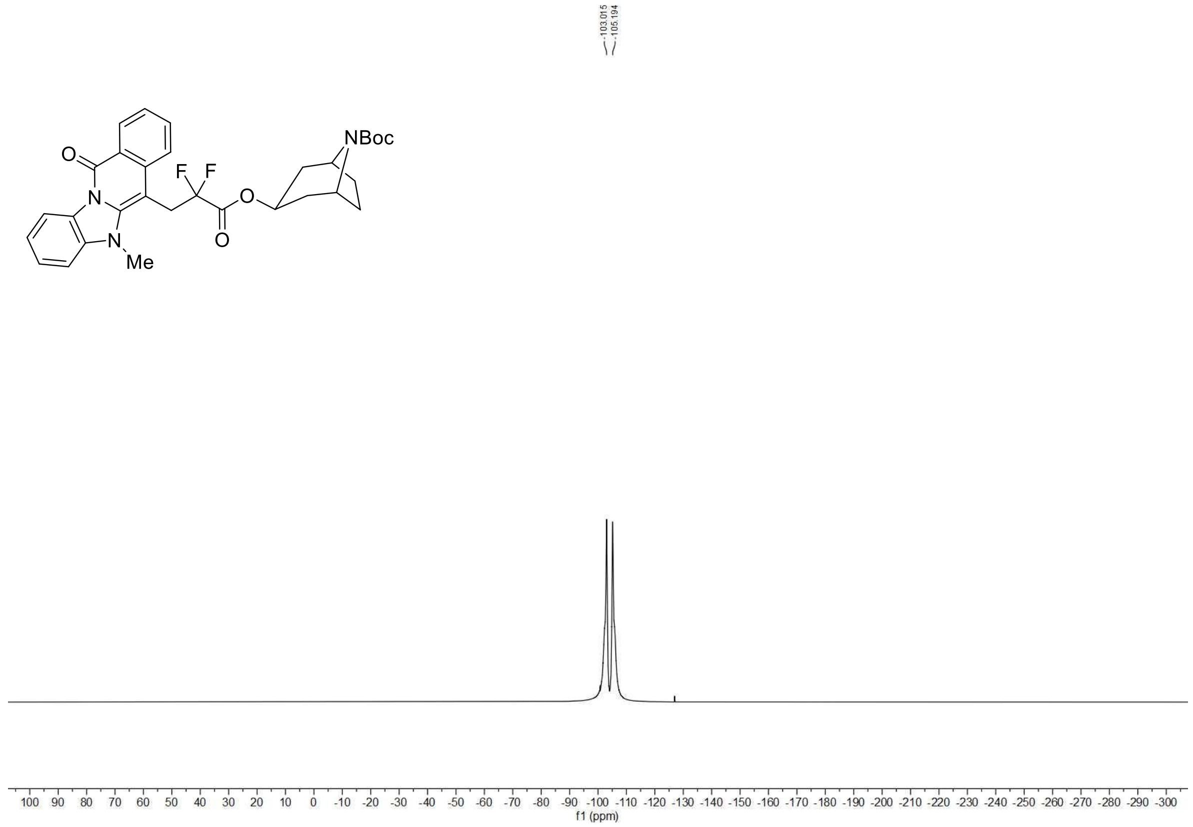

Supplementary Figure 318. ^{19}F-NMR of compound **66**, recorded at 376 MHz and 50 °C in $CDCl_3$.

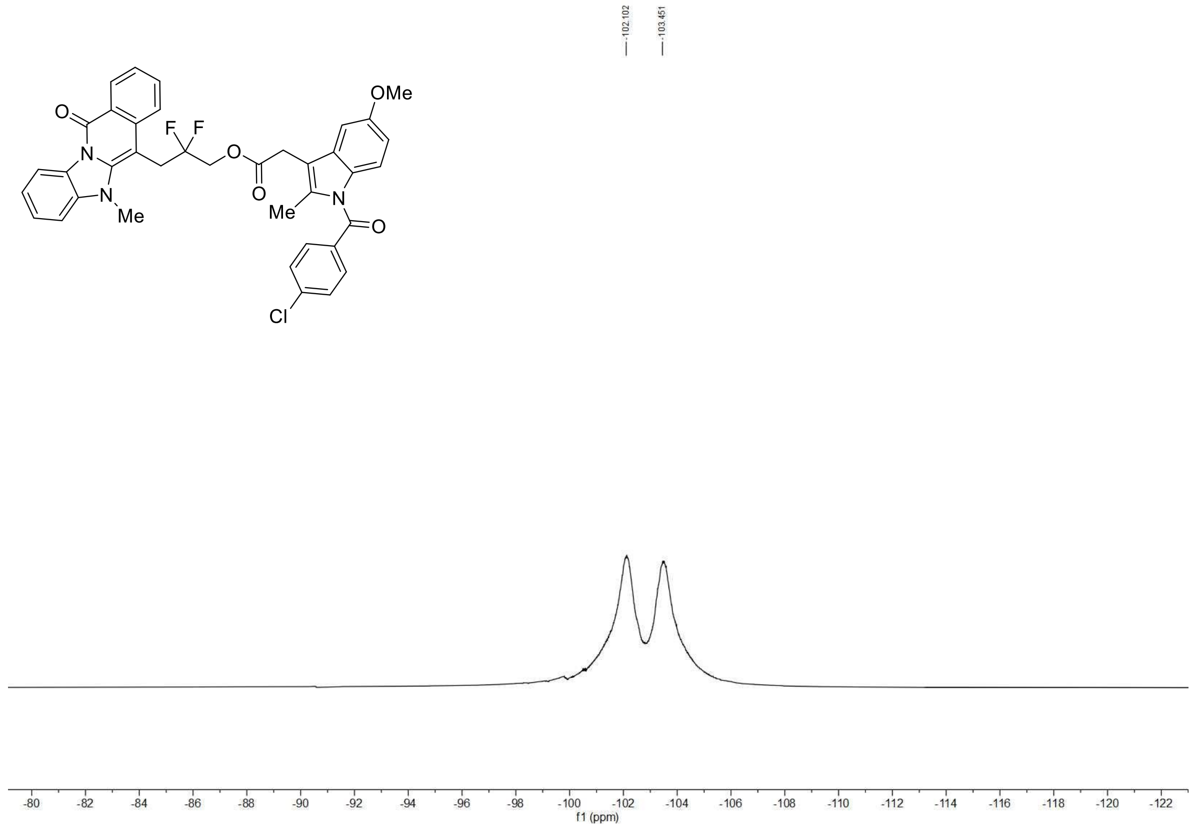

Supplementary Figure 319. ^{19}F-NMR of compound **68**, recorded at 376 MHz and 50 °C in $CDCl_3$.

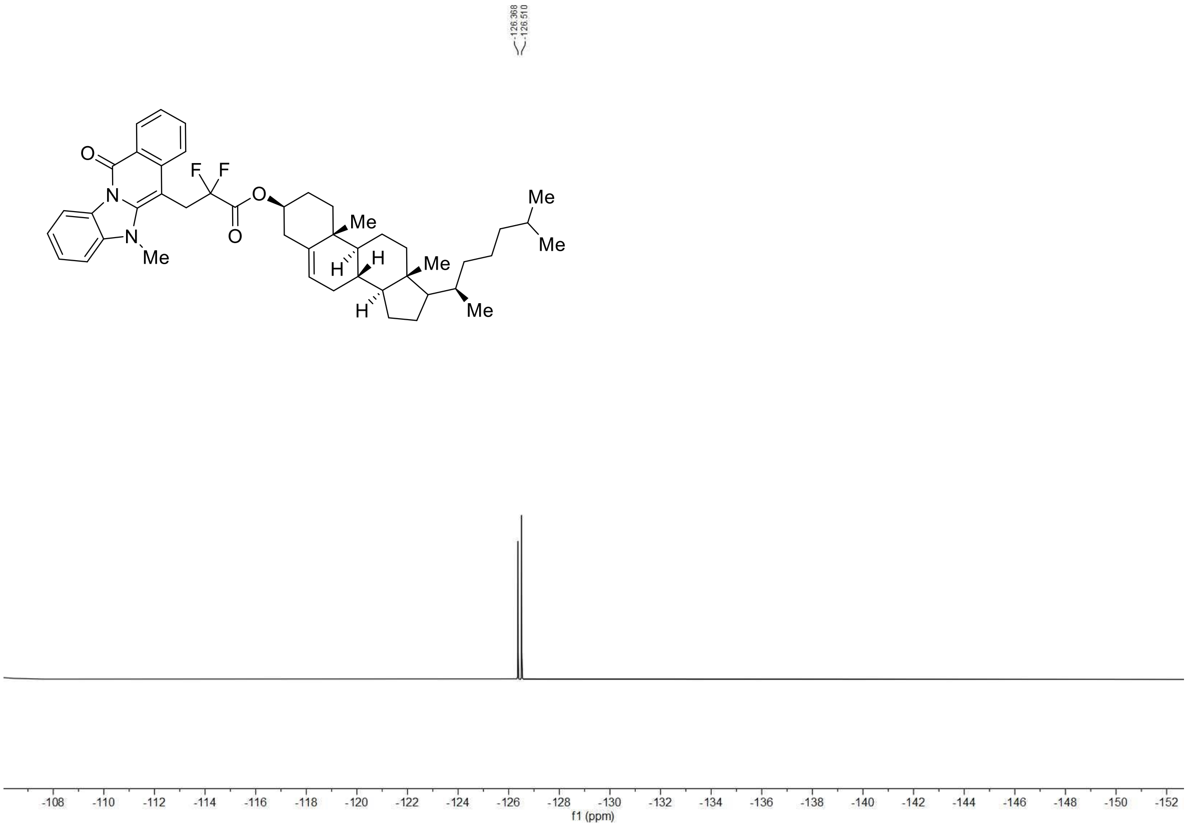

Supplementary Figure 320. ^{19}F-NMR of compound **69**, recorded at 376 MHz and 50 °C in $CDCl_3$.

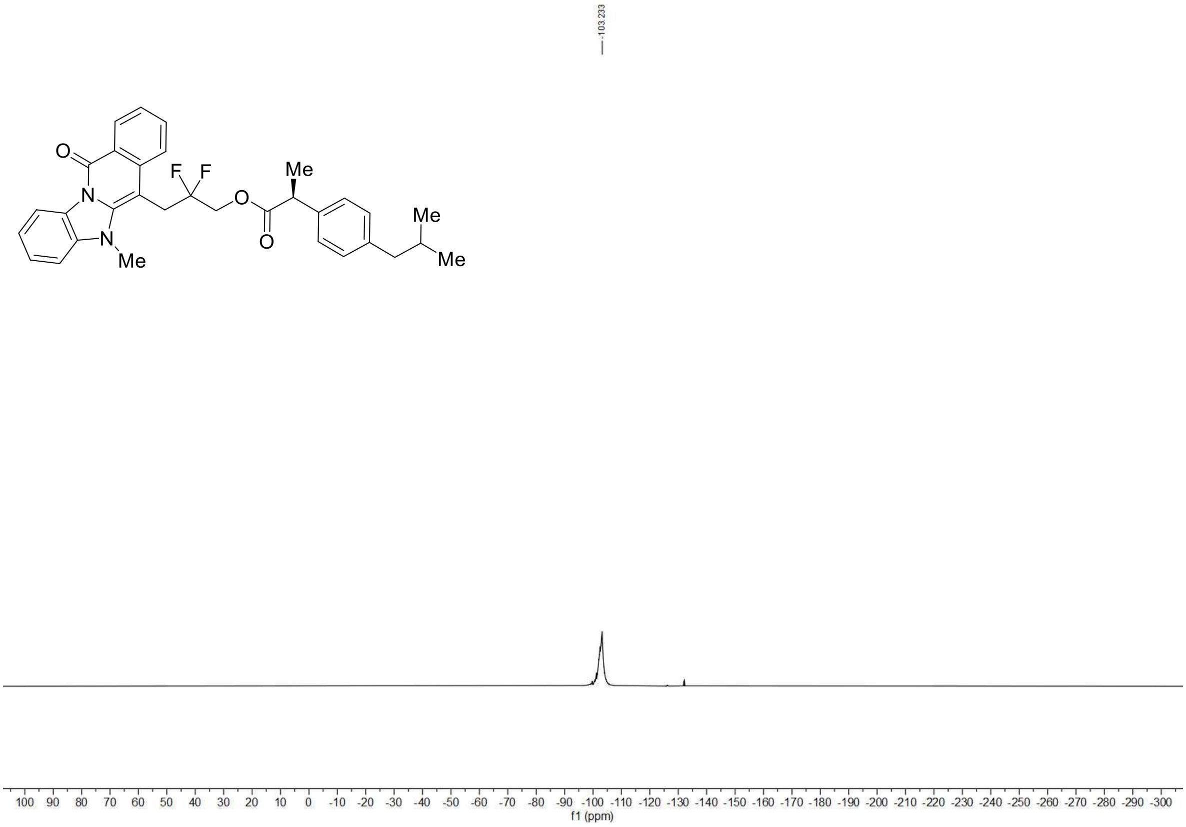

Supplementary Figure 321. ^{19}F-NMR of compound **70**, recorded at 376 MHz and 50 °C in $CDCl_3$.

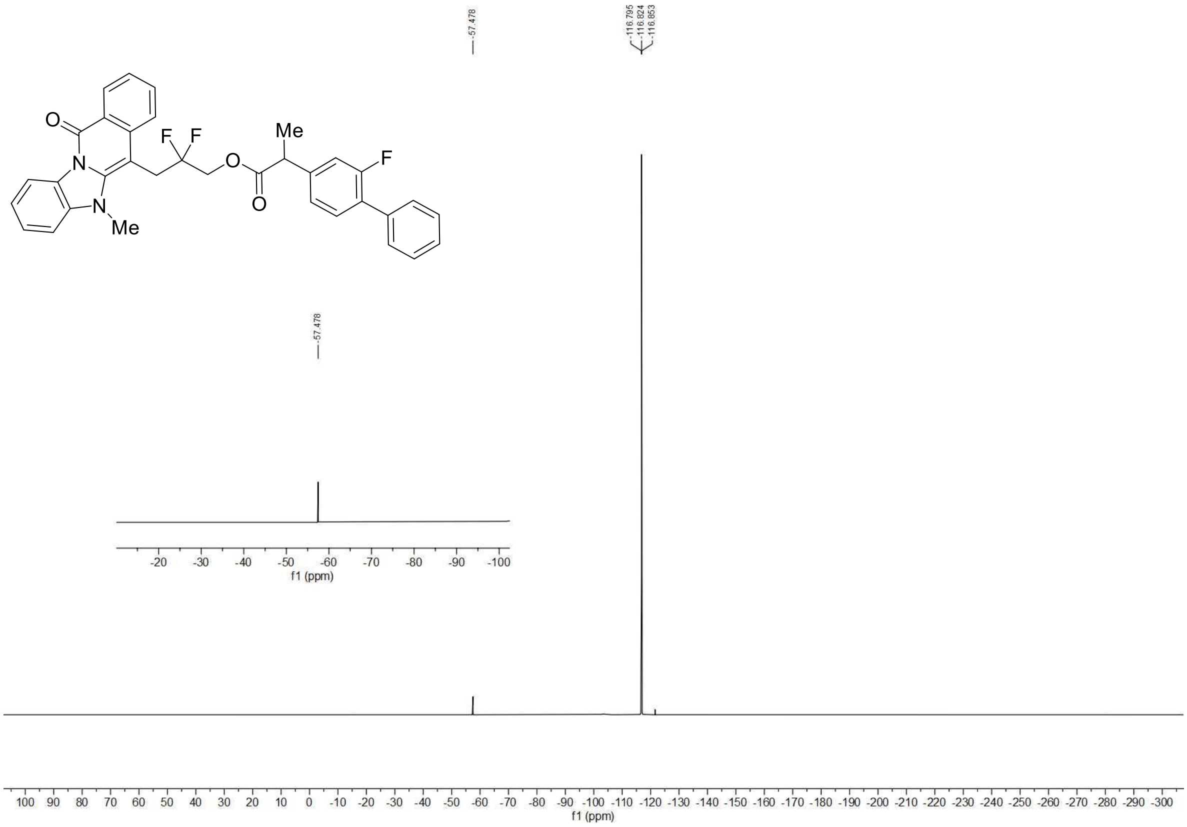

Supplementary Figure 322. ^{19}F-NMR of compound **71**, recorded at 376 MHz and 50 °C in $CDCl_3$.

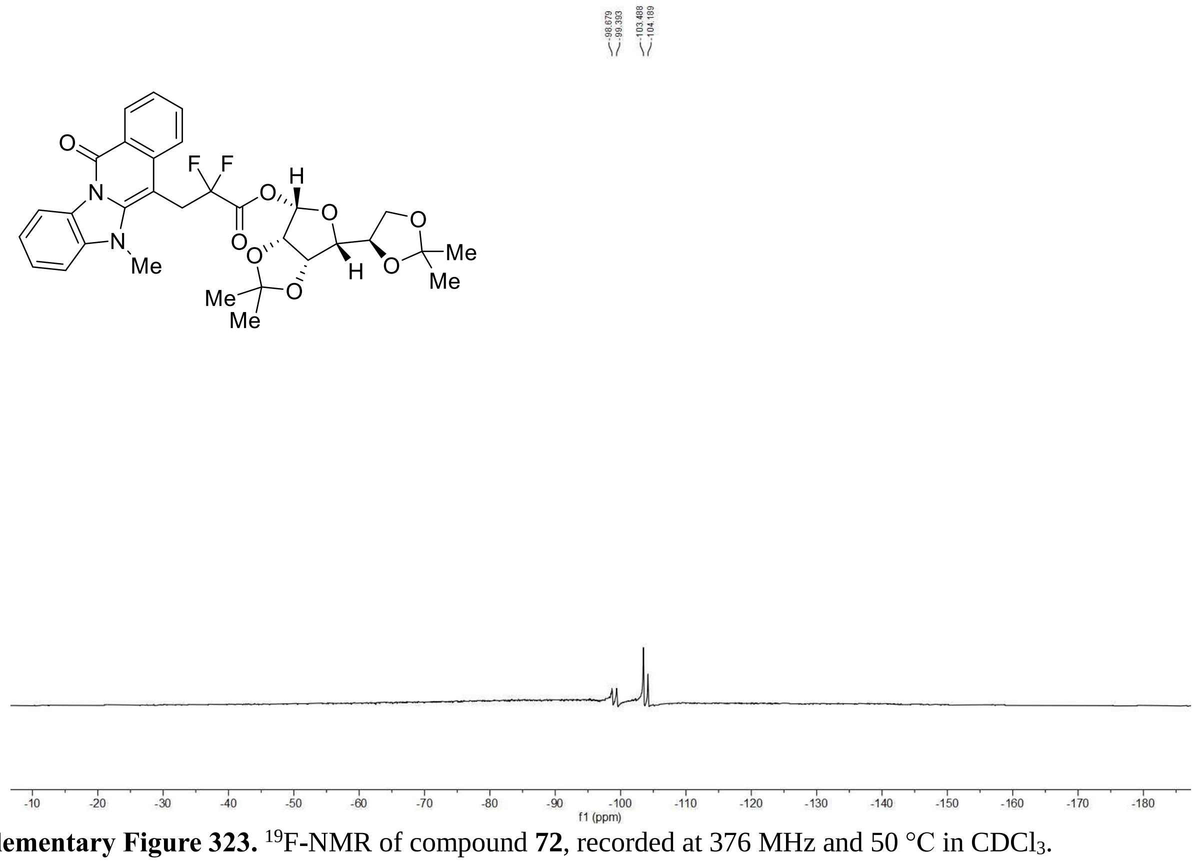

Supplementary Figure 323. ^{19}F-NMR of compound **72**, recorded at 376 MHz and 50 °C in $CDCl_3$.

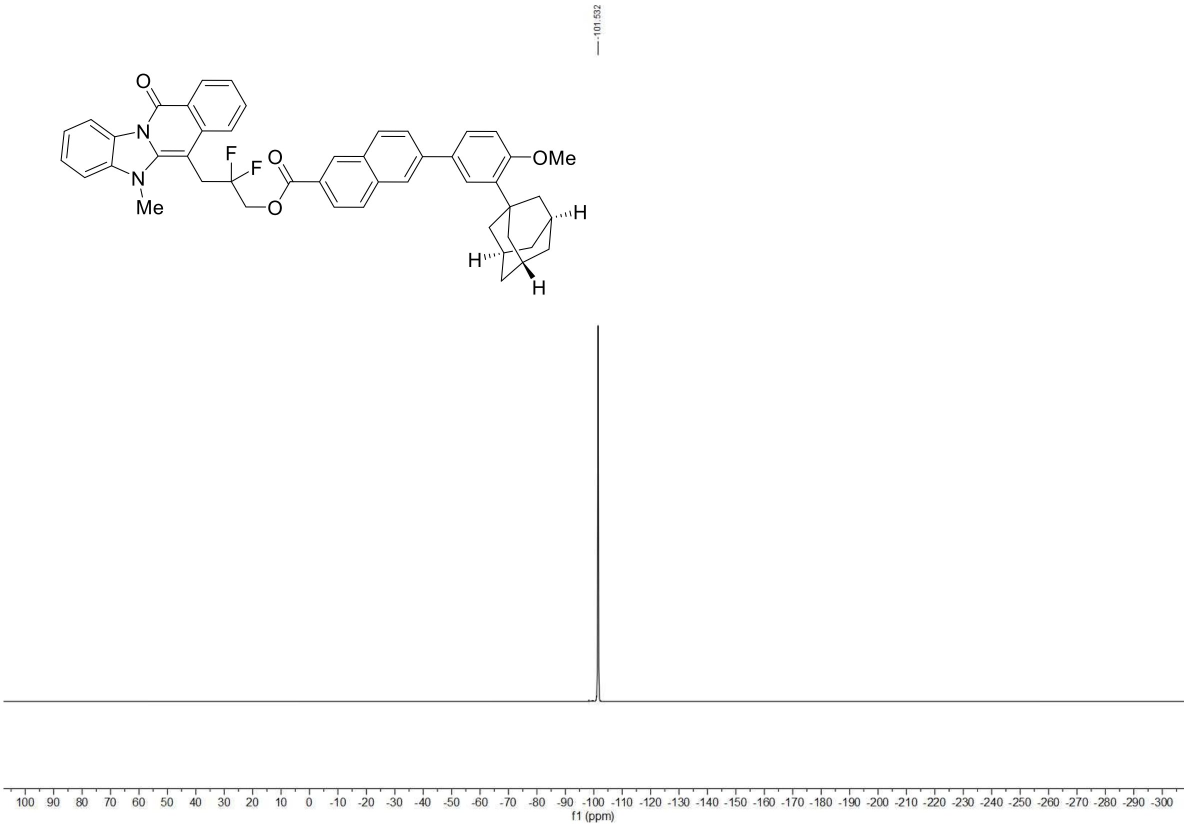

Supplementary Figure 324. ^{19}F-NMR of compound **73**, recorded at 376 MHz and 50 °C in $CDCl_3$.

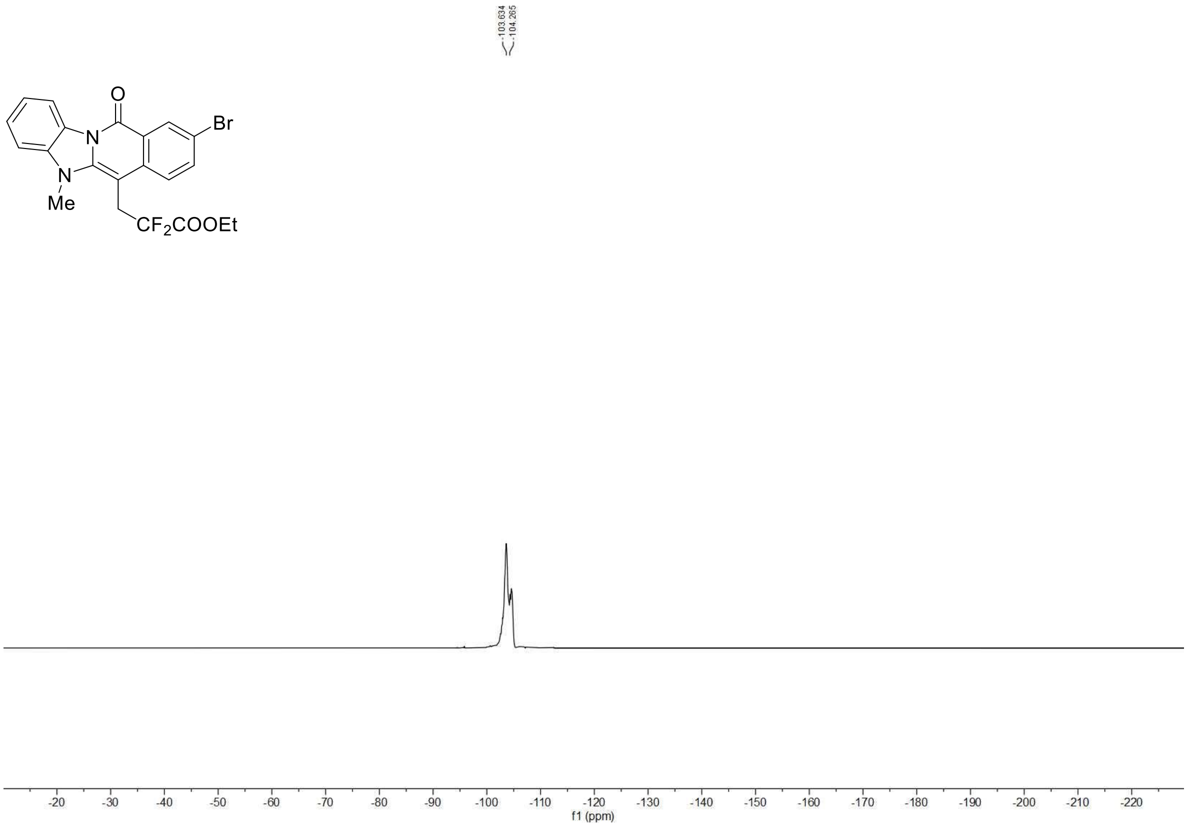

Supplementary Figure 325. ^{19}F-NMR of compound **74**, recorded at 376 MHz and 50 °C in $CDCl_3$.

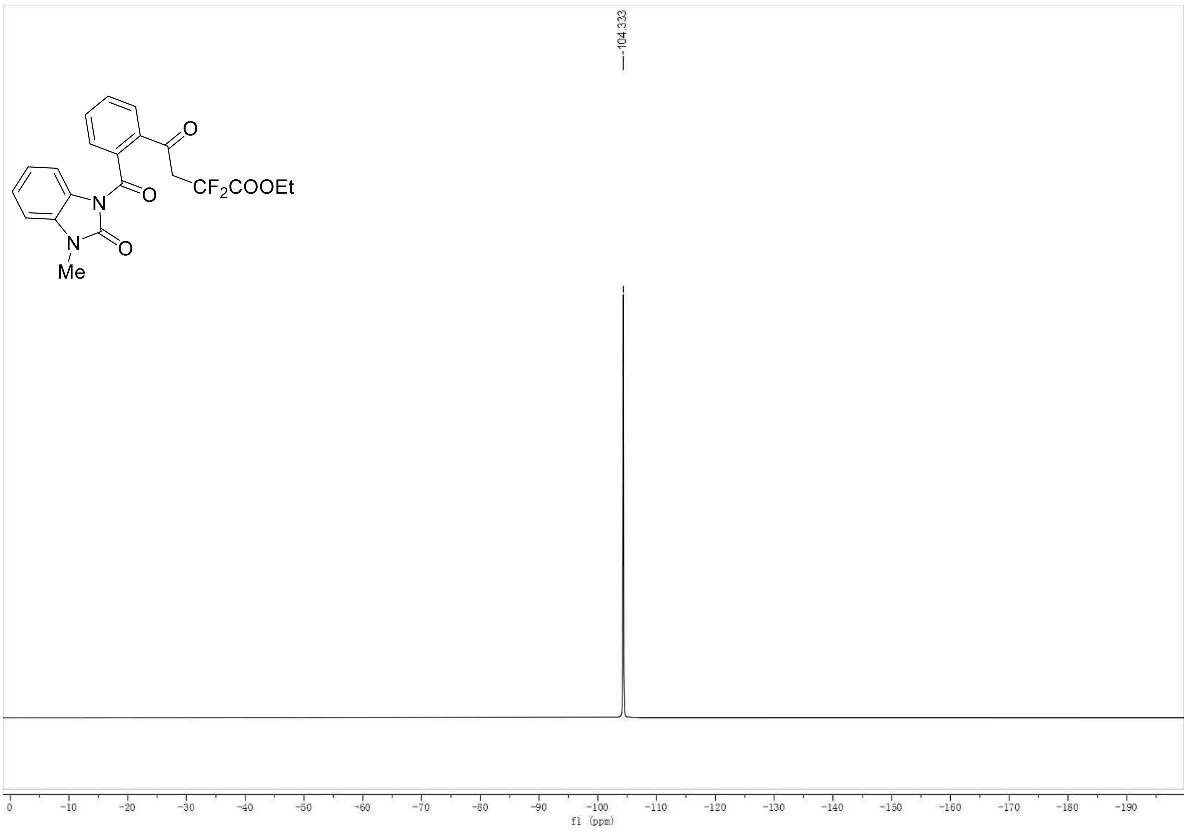

Supplementary Figure 326. ^{19}F-NMR of compound **75**, recorded at 376 MHz and 50 °C in $CDCl_3$.

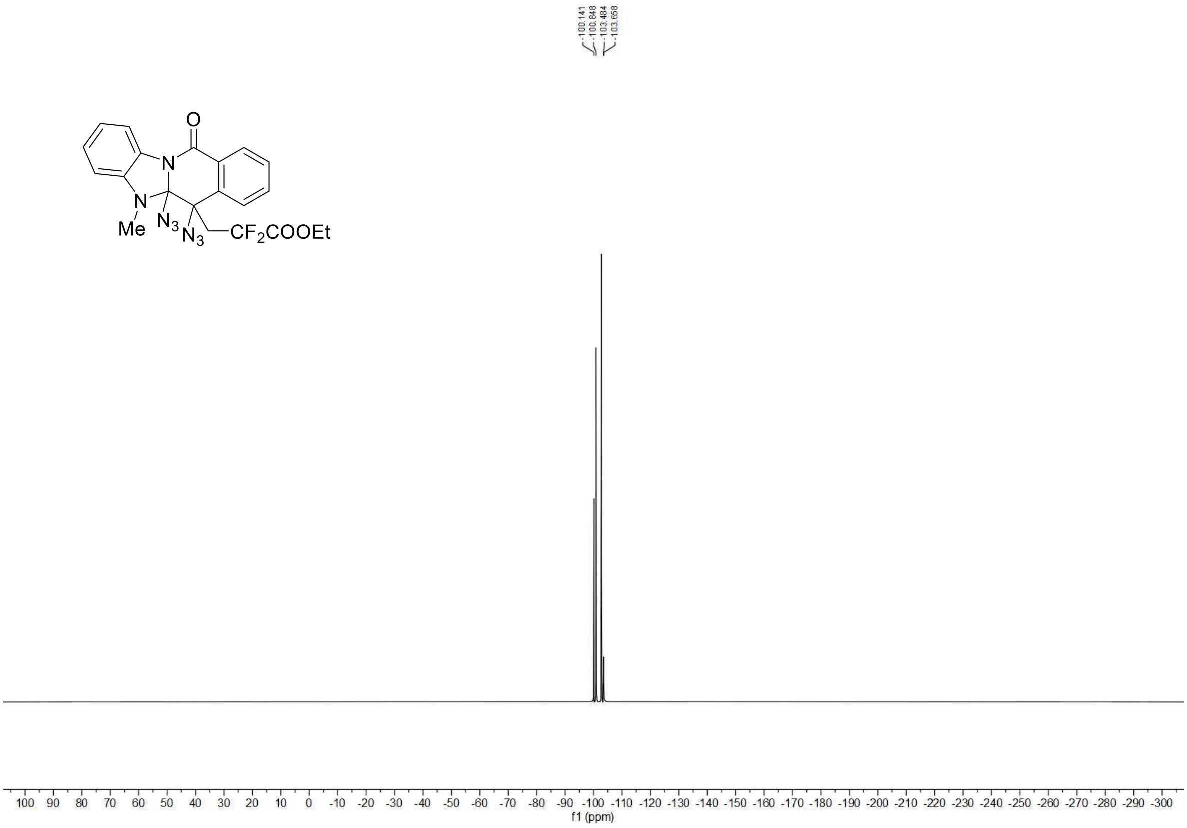

Supplementary Figure 327. ^{19}F-NMR of compound **76**, recorded at 376 MHz and 50 °C in $CDCl_3$.

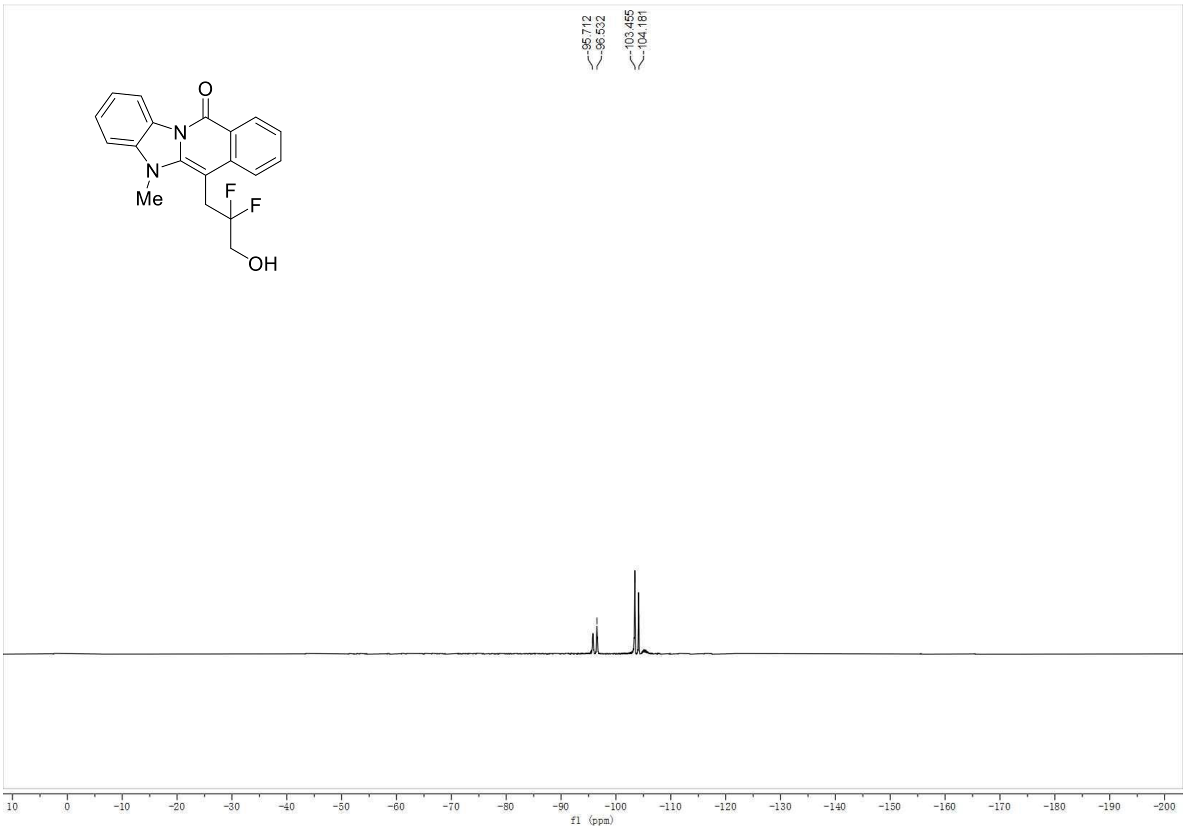

Supplementary Figure 328. ^{19}F-NMR of compound **77**, recorded at 376 MHz and 50 °C in DMSO-d_6.

4. Supplementary References

[1] Andersen, T. L. et al. Efficient 11C-carbonylation of isolated aryl palladium complexes for PET: application to challenging radiopharmaceutical synthesis *J. Am. Chem. Soc.*, **137**, 1548–1555 (2015).

[2] Huang, H.-M. et al. Catalytic radical generation of π-allylpalladium complexes. *Nat. Catal.* **3**, 393–400 (2020).

[3] Hossain, P. A. et al. Organic electroluminescence Device. *PCT Int. Appl.* WO2012163465, 06 Dec 2012.

[4] Tang, C., Jiao, N. Copper-catalyzed C–H azidation of anilines under mild conditions *J. Am. Chem. Soc.* **134**, 18924–18927 (2012).

[5] Li, W.-S., Morrison, H. Long-Range Through-bond photoactivated σ bond cleavage in steroids. Intramolecular sensitized debromination. *Org. Lett*. **2**, 15–18 (2000).

[6] Schonecker, B. et al. Conformational design for 13a-steroids. *J. Org. Chem.* **65**, 5487–5497 (2000).

[7] Li, T., Zhu, F., Zhong, Q. Method for synthesizing menthyl halide with high stereoselectivity. *Faming Zhuanli Shenqing*, CN 103012049 A, Apr 03, 2013.
